# Aboriginal Bacterial Flora in the Uricase-Deficient Rat Gut is Not the Main Factor Affecting Serum Uric Acid

**DOI:** 10.1155/2021/5587642

**Published:** 2021-05-18

**Authors:** Nan Fan, Lvyu Li, Heng Xia, Yinfang Gao, Yongkun Li, Huan Chen, Yu Yun, Weigang Duan

**Affiliations:** ^1^The Department of Pharmacology, School of Basic Medicine, Kunming Medical University, Kunming 650500, Yunnan, China; ^2^Yunnan Provincial Key Laboratory of Molecular Biology for Sinomedicine, School of Basic Medicine, Yunnan University of Traditional Chinese Medicine, Kunming 650500, Yunnan, China; ^3^The Third Affiliated Hospital, Yunnan University of Traditional Chinese Medicine, 25, Eastern Dongfeng Rd., Kunming 650051, Yunnan, China

## Abstract

The relationship between intestinal bacteria and hyperuricemia is a hot research topic. To better understand this relationship, uricase-deficient Sprague–Dawley rats (Kunming-DY rats) were used. The wild-type rats and Kunming-DY rats were used as controls. Kunming-DY rats were treated with ampicillin (90 mg/kg) and ciprofloxacin (150 mg/kg) for 5 days. Bacterial 16S rDNA in the fresh stool was sequenced, and the abundance was calculated. The rats' serum uric acid (SUA) level was assayed, and the rats' intake and output in 24 h were recorded. The bacterial diversity in three groups' fresh stool was analyzed. The gut bacterial diversity and abundance changed in the Kunming-DY rats. More than 99% of bacteria were inhibited or killed by the combination of antibiotics. In contrast to each of the antibiotics alone, the combination of antibiotics lowered the Kunming-DY rats' SUA level; it also caused mild diarrhea, which increased uric acid excretion through stool. These results suggested that the aboriginal gut bacteria in uricase-deficient rats play a minor role in determining the SUA levels. It is too early to conclude that aboriginal gut bacteria are a tempting target for lowering SUA levels.

## 1. Introduction

Hyperuricemia and associated disorders are a common threat to human health in modern society. Approaches to effectively controlling these disorders are a hot topic in medical science fields. The critical solution is to keep the serum uric acid (SUA) level below 70 *μ*g/ml [1]. Such a strategy has been supported by the results of epidemiologic studies at a large scale, considering that the lower SUA levels are highly correlated with less frequent attacks of gout or related diseases [1].

Uric acid is synthesized by xanthine dehydrogenase (Xdh) and can be degraded by uricase (urate oxidase, Uox). Therefore, in humans and animals that do not express uricase, uric acid is an end product of purine metabolism, namely, in humans, a gene encoding uricase is a pseudogene that can be transcribed without translation. The strategies for lowering SUA levels can be grouped into two classes; one strategy is to inhibit uric acid synthesis and the other is to increase uric acid excretion through urine or the intestinal tract. The intestinal tract is a new effective target to lower the SUA level because the amount of uric acid distributed in intestinal fluids is about two or three times higher than that in blood [2]. The effectiveness of targeting the intestinal tract to lower the SUA level has been shown after treatment with oral uricase [2, 3], oral urate sorbents, such as montmorillonite [4], and laxatives [5].

The intestinal tract is a dominant place where microbes reside. Since uricase is almost universally expressed in microorganisms [6], it is taken for granted that microbes play a vital role in lowering the SUA levels. The SUA levels can be lowered by altering gut microbiota [7] or inoculating engineered bacteria that highly express uricase [8]. However, microorganisms are highly metabolically active in the gut; they also synthesize purines, the substrates for uric acid, and express uricase to degrade uric acid. Furthermore, most experiments on lowering the SUA levels by affecting bacteria were carried out in animals who expressed uricase [2–4, 7, 8]. In order to exclude the tangling factor of the host's uricase, uricase-deficient (Uox^−/−^) rats were generated by our team on the background of Sprague–Dawley (SD) rats; they were named “Kunming-DY rats” [9], a type of closed colony animals rather than inbred animals, such as C57BL/6J [10, 11]. Different from Uox^−/−^ mice [10, 11], the SUA level in the male Uox^−/−^ rats was about 48.3 ± 19.1 *μ*g/ml [9], which significantly increased but still much lower than that in Uox^−/−^ mice. Since the level of SUA in the Uox^−/−^ rats was similar to that in men, it has been suggested that the rats could be one of the optimal animals to study gout and associated diseases.

The present study investigated the aboriginal bacteria in the gut of Uox^−/−^ rats and evaluated their relationship with SUA levels.

## 2. Materials and Methods

### 2.1. Materials

Wild-type SD rats of 45 days old were obtained from Chengdu Dossy Experimental Animals Co., Ltd., Chengdu, China (certification no. SCXK (Chuan) 2008–24). Uox^−/−^ (Kunming-DY) and wild-type rats of the same age (45 days old) were generated in our laboratory as previously described [9].

Uric acid assay kits of the phosphotungstic acid method (lot: C012-1-1) were purchased from Nanjing Jiancheng Bioengineering Institute (Nanjing, China). The Mag-MK Soil and Stool Genome DNA Extraction Kit (B618763) was purchased from Sangon Biotech (Shanghai) Co., Ltd. (Shanghai, China).

The fluorescent quantitative PCR apparatus (StepOne Plus) was manufactured by ABI (Foster, CA, USA). Ultrapure water was produced by a Milli *Q* water purification system manufactured by EMD Millipore Group (Darmstadt, Germany). The NanoDrop ND-1000 spectrophotometer used for experiments was manufactured by PeqLab (Erlangen, Germany). The multiple microplate reader Infinite 200pro (Tecan Group, Mannedorf, Switzerland) was also used. Other instruments or reagents used in the present study were made in China.

### 2.2. Animal Breeding

Uox^−/−^ rats were generated, identified, and raised in our laboratory as previously described [9]. Briefly, the male Uox^−/−^ rats were mated with the female Uox^−/−^ rats for three weeks to generate their offspring. Their offspring were breastfed by their mothers to the age of three weeks; then, the mothers, the male offspring, and the female offspring were separated and put in three individual cages. When the male offspring reached 45 days old, they were used in this study.

Five nests of male Uox^−/−^ rats and two nests of male wild-type rats were enriched. The rats were kept at 22°C, humidity of 45%–55%, under natural light, and with an accessible approach to food and water. All animal experiments were approved by the Animal Care and Use Committee of Kunming Medical University (approval no. KMMU-2020196) and performed under the Guidelines for Ethical Review of Laboratory Animal Welfare of China.

After the experiment, the living rats were intraperitoneally anesthetized with urethane (1.0 g/kg). Under deep anesthesia, their necks were dislocated for euthanasia. The rat bodies had been collected in yellow plastic bags and kept in a refrigerator at −20°C until they were taken away by a green company for cremation.

### 2.3. SUA Levels in Uox^−/−^ Rats Treated with Ampicillin or Ciprofloxacin

Eighteen Uox^−/−^ rats were randomized into three groups: ampicillin group (Am), ciprofloxacin group (Cip), and combination group (Am + Cip). Every group included six rats. The rats in the Am group were intragastrically administered with ampicillin (90 mg/kg) for 5 days, the Cip group was treated with ciprofloxacin (150 mg/kg), and the Am + Cip group was treated with ampicillin (90 mg/kg) in the morning and ciprofloxacin (150 mg/kg) in the afternoon. Twelve Uox^−/−^ rats were randomized into the control group and treated with normal saline of the same volume (10 ml/kg).

Before they were administered with drugs, the rats had been kept in metabolic cages to obtain their intake of food and water and output of urine and stool in 24 h. In addition, a 200-*μ*l blood sample without anticoagulation was drawn from their tail vein using a tiny needle. During blood sampling, the animals were awake at a local atmosphere of 28°C–32°C. Serum was obtained by spinning at 3,000 g and 4°C for 5 min as soon as the blood coagulated. At the end of the five-day experimental period, the animals were individually kept in metabolic cages. We recorded the amount of food and water they consumed in the last 24 h. Their stool and excreted urine were also collected and recorded; they were collected on ice in a cold insulation box for analysis.

The urine was soon stirred to a homogeneous state, and 1.2 ml was sampled. The urine sample was quickly diluted 20 times to obtain the final sample for a uric acid assay. The stool was weighed and then mixed with 3-fold weight of Tris solution (100 mmol/L), and the mixture was stirred at a shaker at 100 rpm for 4 h. The mixture was spun at 5,000 g and 4°C for 5 min, and the supernatant was collected for the final uric acid assay.

The samples for uric acid assay were kept at −20°C or determined as soon as possible to prevent the false elevation of uric acid levels by xanthine dehydrogenase in rat samples [12]. Uric acid in the final samples was assayed using the uric acid assay kits. The assay kits had a quantification range of uric acid from 3.91 *μ*g/ml to 125 *μ*g/ml. If the uric acid levels in the samples had exceeded the kits' quantification range, the samples were diluted. The protocol of uric acid assay kit is available at the following website: http://www.njjcbio.com/uploadfile/product/big/20190612093216738.pdf.

### 2.4. Fresh Stool Prepared from Rats

Twelve Uox^−/−^ rats were randomized into the normal Uox^−/−^ group (Uox^−/−^−1) and the combination group (Uox^−/−^−2). Each group contained six rats, as mentioned above. Another six wild-type SD rats were randomized into the WT group. The rats in the Uox^−/−^−2 group were intragastrically given ampicillin (90 mg/kg) in the morning and ciprofloxacin (150 mg/kg) in the afternoon for 5 days. The rats in Uox^−/−^-1 or the WT group were treated with normal saline of the same volume (10 ml/kg). After the rats had been administered for 5 days, their fresh stool was collected. The stool was divided into two aliquots: one was used for microculture and the other for microbiome assay.

### 2.5. Microculture of Fresh Stool

Their stool was diluted with sterilized normal saline solution to 1 : 10^7^. The mixture was adequately stirred, and 100 *μ*l was piped to the LB solid medium surface (containing 10 g peptone, 5 g yeast extract, 10 g sodium chloride, and 8 g agar-agar in 1,000 ml and autoclaved) in a culture dish of 30 square inches. The mixture was evenly dispersed to cover the surface of the dish. The culture dishes were kept at 37°C for 24 h. The dishes were photographed, and the clones on the surface of the solid medium were counted.

### 2.6. Microbiome Assay of Fresh Stool

The other aliquot of the fresh stool, about 200 mg, was used to extract DNA using the Mag-MK Soil and Stool Genome DNA Extraction Kit. The DNA quality and quantity were measured by the NanoDrop ND-1000 spectrophotometer.

The DNA encoding bacterial 16S rRNA was amplified by 25 cycles with primers Bakt_341F (CCTACGGGNGGCWGCAG) and Bakt_805R (GACTACHVGGGTATCTAATCC) [13]. The 5′ end of the primers was designed as adapters for purification, which were CCCTACACGACGCTCTTCCGATCTG and GACTGGAGTTCCTTGGCACCCGAGAATTCCAy [13]. The purified 16S rDNA (about 466 bp) was assessed by a standard agarose gel electrophoresis and was sequenced by Sangon Biotech (Shanghai, China). The quality and quantity of the amplified DNA were also measured by the NanoDrop ND-1000 spectrophotometer. The amplified sequences were purified and sequenced on the Illumina MiSeq platform (Sangon Biotech). The sequence of the reads were BLASTed with gene banks (http://rdp.cme.msu.edu/misc/resources.jsp, http://www.arb-silva.de/, and http://ncbi.nlm.nih.gov/), and the positive sequences (species annotation) were recognized by ≥ 97% sequence similarity. The reads were clustered into operational taxonomic units (OTUs) by referring to the 16S rDNA database. The flora's alpha diversity in every group was measured using the Shannon index (https://mothur.org/wiki/shannon/). Principal component analysis (PCA) was performed to evaluate their beta-diversity.

The exact copies of rDNA in the 50 mg stool sample were also assayed using the quantitative PCR method by the fluorescent quantitative PCR apparatus. Briefly, the rDNA in the samples was extracted, diluted to 50 or 10-fold, and amplified with the primers mentioned above. The anticipated PCR products were about 466 bp. The reaction solution of 20 *μ*l contained SybrGreen qPCR Master Mix (10 *μ*l), primers (10 *μ*M, 0.4 + 0.4 *μ*l), ultrapurified water (7.2 *μ*l), and templates (2 *μ*l). PCR was carried out with predenaturation at 95°C for 3 min, followed by 45 cycles of denaturation step at 95°C for 5 s, annealing at 60°C for 30 s, and extension at 72°C for 30 s. Finally, an additional extension at 72°C for 8 min was performed, and the melting curve of the product was determined. The green fluorescence at every cycle of the reaction was recorded, and the quality of the PCR product was evaluated by the melting curve. The cycle threshold value (CT), indicating the templates' relative abundance, was calculated by the amplification curve. By comparison with the standard amplified PCR products, the exact copies in every rDNA sample were calculated.

### 2.7. Statistical Analyses

Values were expressed as mean + standard deviation (SD) or mean + standard error (SE). If a normal distribution of the original values (or logarithmically transformed) was verified by the normality test (Shapiro–Wilk test), Student's *t*-test or one-way analysis of variance (ANOVA) was performed to compare the means between different groups. If there was a significant difference in ANOVA, the post hoc statistical tests between each two groups were performed with the S-N-K method (equal variances assumed) or with Tamhane's T2 method (equal variances not assumed). Otherwise, a nonparametric test for two independent samples in the Mann–Whitney *U* model (two-tailed) was performed. The correlation between the Uox^−/−^ rat SUA levels and other factors was tested using Pearson's correlation (two-tailed). Statistical significance was accepted at *P* < 0.05.

## 3. Results

### 3.1. SUA Levels in Uox^−/−^ Rats Treated with Antibiotics

**Figure 1:**
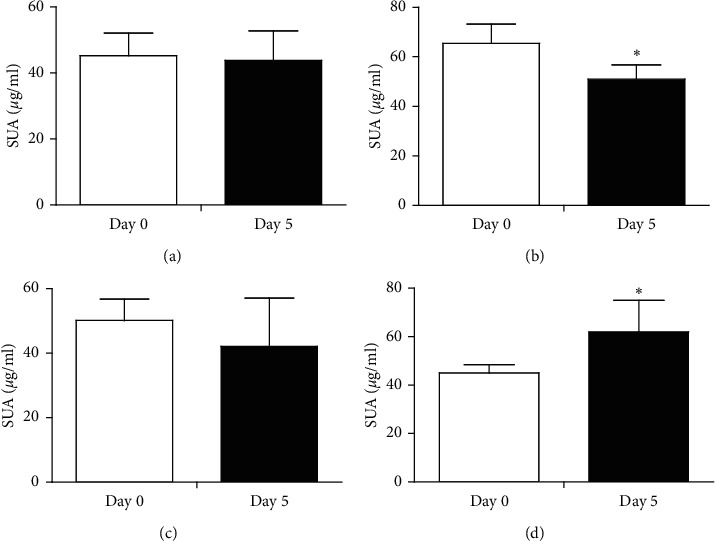
Serum uric acid (SUA) levels in male Uox^−/−^ rats treated with antibiotics (mean + SD, *n* = 12 (control) or 6 (antibiotics)). (a) control group, treated with normal saline; (b) Am + Cip group, treated with ampicillin (90 mg/kg) and ciprofloxacin (150 mg/kg); (c) Am group, treated with ampicillin (90 mg/kg); (d) Cip group, treated with ciprofloxacin (150 mg/kg). Day 0, the day before treatment; day 5, treated with drugs for 5 days. ^*∗*^*P* < 0.05 vs. day 0, paired-sample *t*-test.

The SUA levels in Uox^−/−^ rats were relatively stable during 5 days (Figure 1(a)). However, when the rats were treated with the combination of antibiotics (ampicillin, 90 mg/kg, and ciprofloxacin, 150 mg/kg), the SUA levels decreased rather than increased (Figure 1(b)). Ampicillin (90 mg/kg) had almost no significant effects on the rat SUA levels (Figure 1(c)). To our surprise, ciprofloxacin 1 (50 mg/kg) increased the rat SUA level (Figure 1(d)).

### 3.2. Intake and Output of Uox^−/−^ Rats Treated with Ampicillin and Ciprofloxacin

**Figure 2:**
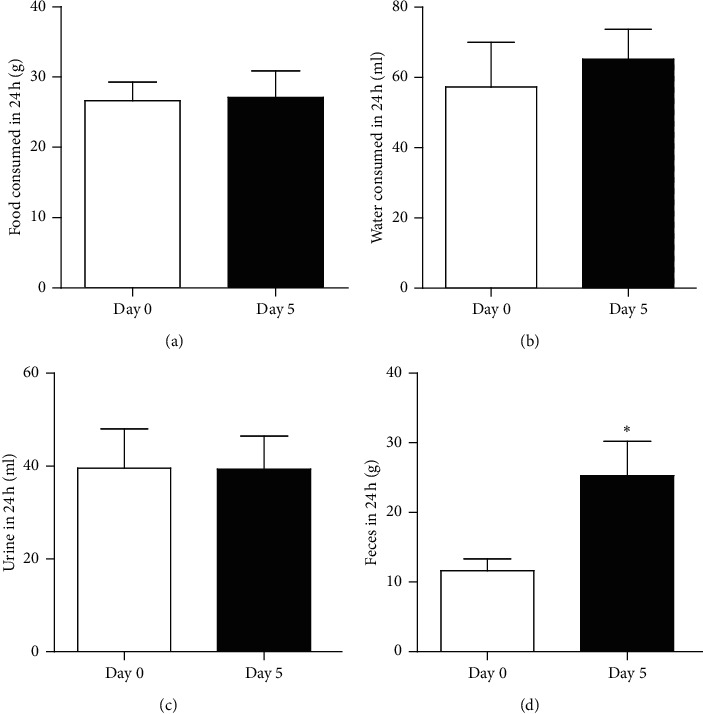
Food and water intake and urine and stool output of the male Uox^−/−^ rats treated with antibiotics for 5 days (mean + SD, *n* = 6). There were no significant differences between day 0 and day 5 in food (a) and water (b) consumption in 24 h. There was no difference between day 0 and day 5 in the volume of 24 h urine (c) either. However, in day 5, the rats excreted more amount of stool (d). Day 0, the day before treatment; day 5, treated with ampicillin (90 mg/kg) and ciprofloxacin (150 mg/kg) for 5 days. ^*∗*^*P* < 0.05 vs. day 0, paired-sample *t*-test.

Uox^−/−^ rats consumed the same quantity of food (Figure 2(a)) after treatment with the combination of antibiotics. The consumed water increased after the rats had been treated with the antibiotics, but without reaching significance (Figure 2(b)). Simultaneously, the rats excreted urine of the same volume (Figure 2(c)), but they excreted more stool (Figure 2(d)). Since there was no difference and tendency in food consumed between day 0 and day 5, there was an increasing tendency in water intake and the increased stool contained more water, suggesting that the rats had mild diarrhea.

### 3.3. Uric Acid in Uox^−/−^ Rat Urine and Stool Treated with Antibiotics

**Figure 3:**
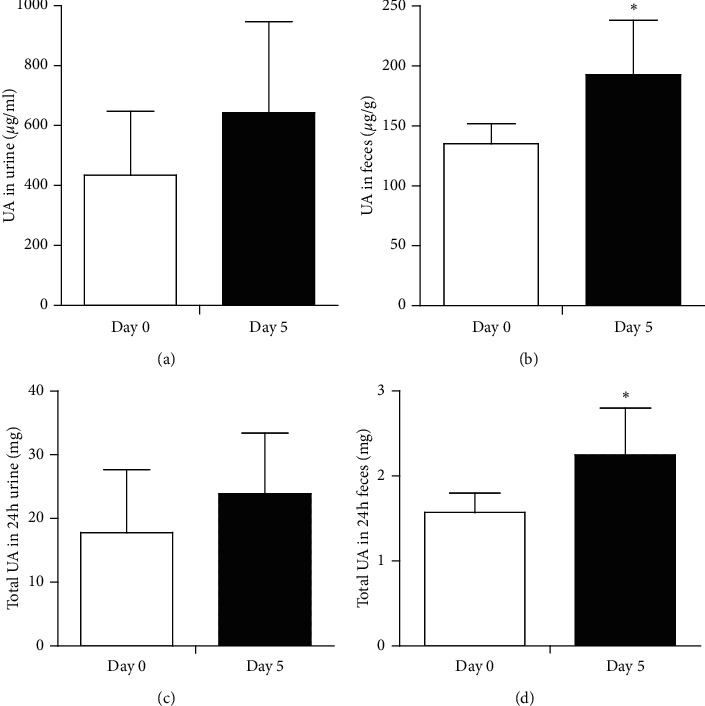
Uric acid in urine and stool excreted by the male Uox^−/−^ rats treated with antibiotics for 5 days (mean + SD, *n* = 6). When treated with the combination of antibiotics for 5 days, the rats excreted more uric acid in their urine (a, c), but without reaching statistical significance; however, they significantly excreted more uric acid in stool at both concentration (b) and amount (d) levels in 24 h. Day 0, the day before treatment; day 5, treated with ampicillin (90 mg/kg) and ciprofloxacin (150 mg/kg). ^*∗*^*P* < 0.05 vs. day 0, paired-samples *t*-test.

Since the SUA level in Uox^−/−^ rats was lowered by the combination of antibiotics, it should be explained whether this was associated with the increase in uric acid excretion. Unsurprisingly, there was a tendency toward increased uric acid in urine at concentration (Figure 3(a)) and amount (Figure 3(c)) levels, although neither of the levels reached statistical significance. However, the uric acid excreted through stool significantly increased at both concentration (Figure 3(b)) and amount (Figure 3(d)) levels. The increased uric acid in urine and stool could be the logical cause of the lowered SUA level (Figure 1(b)).

### 3.4. Bacterial Abundance in Uox^−/−^ Rat Stool Measured by Culturing

**Figure 4:**
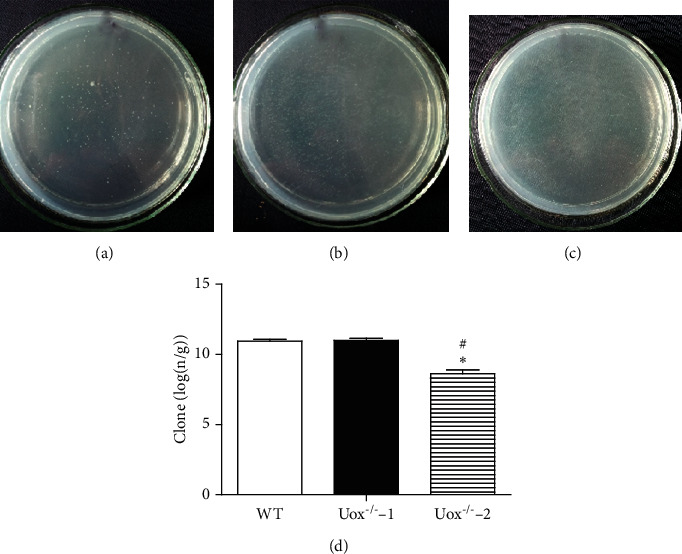
Bacterial abundance in the Uox^−/−^ rats measured by culturing clones on the solid LB medium. The fresh stool was diluted to 10^7^ folds, and a 100 *μ*l sample was cultured on the surface. The aerobic bacteria grew on the surface and developed clones. The data of clones in the three groups were logarithmically transformed before statistical analysis. (a) Clones from the wild-type rat stool, (b) clones from the Uox^−/−^ rat stool, (c) clones from the stool of Uox^−/−^ rat treated with ampicillin (90 mg/kg) and ciprofloxacin (150 mg/kg) for 5 days, (d) summary of (a, b, c) (mean + SD, *n* = 6). WT, stool from the wild-type rats; Uox^−/−^−1, stool from the normal Uox^−/−^ rats; Uox^−/−^−2, stool from the Uox^−/−^ rats treated with antibiotics for 5 days. ^#^*P* < 0.05 vs. WT; ^*∗*^*P* < 0.05 vs. Uox^−/−^−1, one-way ANOVA.

The fresh stool was diluted to 10^7^ folds and cultured on the surface of the solid LB medium. Aerobic bacteria grew on the surface and developed clones. The data of clones in the three groups were logarithmically transformed before statistical analysis.  Figure 4 showed that there were more bacteria in Uox^−/−^ rat stool (Figures 4(b) and 4(d)) than in that of wild-type rats (Figures 4(a) and 4(d)), although the difference did not reach significance. Bacteria in Uox^−/−^ rat stool significantly decreased if their hosts were treated with the combination of antibiotics for 5 days (Figures 4(c) and 4(d)). The number of aerobic bacteria in the stool of Uox^−/−^ rats treated with the combination of antibiotics was about 0.43% of that in normal Uox^−/−^ rat stool, suggesting that the bacteria were dramatically killed or inhibited by the combination of antibiotics.

### 3.5. Bacterial Abundance in Uox^−/−^ Rat Stool Measured by DNA

**Figure 5:**
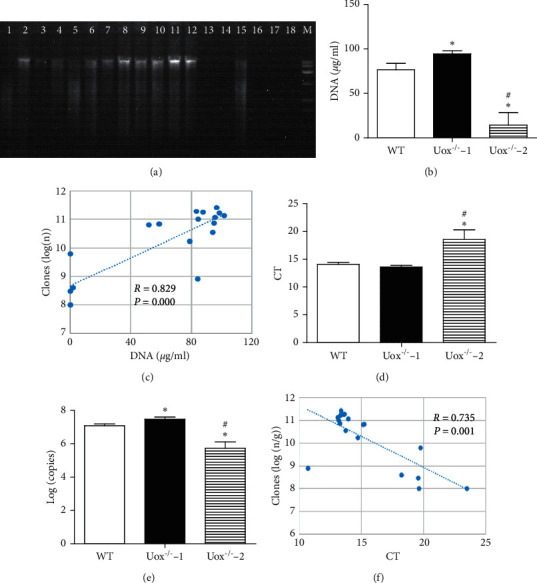
Bacterial abundance in Uox^−/−^ rat stool measured by DNA (mean + SD, *n* = 6). (a) Total DNA extracted from rat stool (nos. 1–6 were from WT rat stool, nos. 7–12 were from normal Uox^−/−^ rats', nos. 13–18 were from Uox^−/−^ rats treated with the combination of antibiotics, and “M” was the marker with the brightest band of 5,000 bp); (b) DNA content in samples (mean + SD, *n* = 6); (c) the correlation between the amount of bacterial DNA and the quantity of living bacteria (*n* = 18), *P*=0.000, Pearson's correlation (two-tailed); (d) cycle threshold (CT) values of 16S rDNA in samples (mean + SD, *n* = 6); (e) copies of 16S rDAN in samples (mean + SD, *n* = 6); (f) the correlation between CT values and the quantity of living bacteria (*n* = 18), *P*=0.001, Pearson's correlation (two-tailed). WT, stool from the wild-type rats; Uox^−/−^-1, stool from the normal Uox^−/−^ rats; Uox^−/−^−2, stool from the Uox^−/−^ rats treated with ampicillin (90 mg/kg) and ciprofloxacin (150 mg/kg) for 5 days. ^#^*P* < 0.05 vs. WT; ^*∗*^*P* < 0.05 vs. Uox^−/−^−1, one-way ANOVA.

Bacteria contain DNA. More DNA was extracted from Uox^−/−^ rat stool than from wild-type rat stool of the same weight (Figures 5(a) and 5(b)). However, less DNA was extracted from the stool of the Uox^−/−^ rats treated with the combination of antibiotics for 5 days (Figures 5(a) and 5(b)). There was a good positive correlation between the amount of bacterial DNA and the quantity of living bacteria (Figure 5(c)), suggesting that the amount of bacterial DNA can be a macroscopic evaluation index in evaluating the relative abundance of bacteria.

The sample containing 16S rDNA was amplified in a quantitative model by the fluorescent quantitative PCR apparatus. The CT value was obtained from the amplification curve. The CT value of the sample containing 16S rDNA from Uox^−/−^ rat stool was lower than that of wild-type rats, while the CT value from stool of Uox^−/−^ rats treated with the combination of antibiotics was the highest (Figure 5(d)). Further results showed that Uox^−/−^ rat stool had the highest number of copies of 16S rDNA, and stool from Uox^−/−^ rats treated with the combination of antibiotics had the lowest (Figure 5(e)), about 1.03% of that in stool of normal Uox^−/−^ rats. Because the exact copies were calculated from the CT values, a low CT value also meant a high abundance of 16S rDNA. There was a strong negative correlation between the CT values and the number of living bacteria (Figure 5(f)), suggesting that the CT value can be used to evaluate the relative abundance of bacteria.

### 3.6. Bacterial Flora in Uox^−/−^ Rat Stool

**Figure 6:**
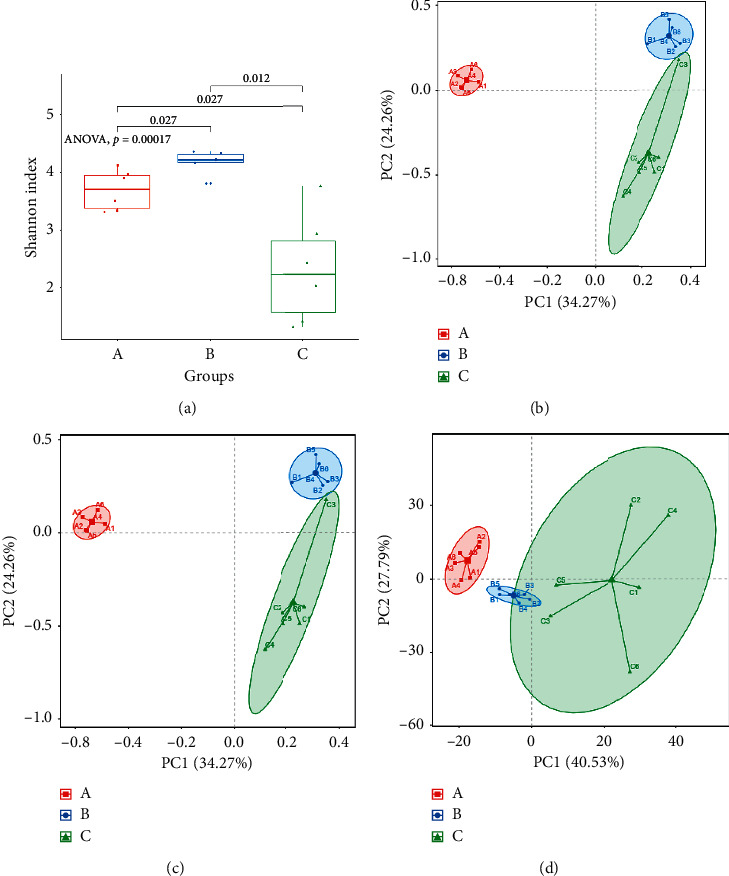
The floral diversity of bacteria in the Uox^−/−^ rat stool (*n* = 6). (a) Alpha diversity (Shannon index) (*n* = 6); ^*∗*^*P* < 0.05, Student's-*t* test; (b) beta-diversity based on the PCA at the OTU level; (c) beta-diversity at the genus level; (d) beta-diversity at the species level. Group A, wild-type rat stool; group B, normal Uox^−/−^ rats' stool; group C, stool from the Uox^−/−^ rats treated with antibiotics (ampicillin, 90 mg/kg, and ciprofloxacin, 150 mg/kg) for 5 days.

The stool rDNA was amplified, sequenced, and BLASTed. Finally, 937 OTUs were obtained in all the samples, more than 99.9% of which clustered in the bacteria domain. Further annotation results showed that they were clustered in 33 phyla, 41 classes, 83 orders, 157 families, 348 genera, and 461 species. The results of the alpha diversity analysis, as shown in Figure 6(a), elucidated that the Uox^−/−^ rat stool had more species measured by OTUs. The results suggested that uricase-deficiency increased the bacterial diversity, and the combination of antibiotics significantly decreased the bacterial diversity.

**Figure 7:**
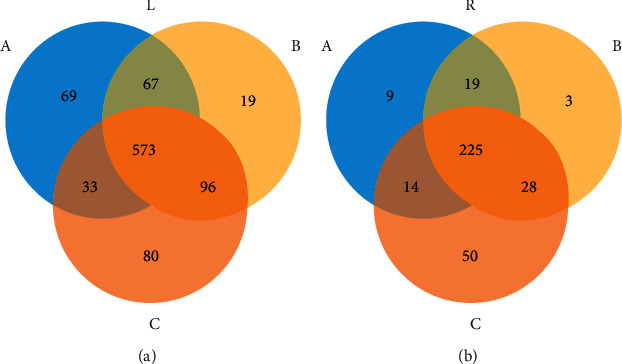
Venn distribution of the bacteria in the Uox^−/−^ rat stool at OTU (a) and genus (b) levels (*n* = 6). Group A (blue), wild-type rat stool; group B (yellow), Uox^−/−^ rat stool; group C (orange), stool from Uox^−/−^ rats treated with antibiotics (ampicillin, 90 mg/kg, and ciprofloxacin, 150 mg/kg) for 5 days.

Beta-diversity was determined based on the PCA, and the results are shown at OTU (Figure 6(b)), genus (Figure 6(c)), and species (Figure 6(d)) levels. The results clarified that the bacterial pattern was significantly differed between the groups. However, according to the results shown in Venn distribution of the bacteria in Uox^−/−^ rat stool at OTU (Figure 7(a)) and genus (Figure 7(b)) levels, about 60% of the bacteria were still shared among the three groups, at both the OTU level and genus level.

### 3.7. Bacterial Abundance in Uox^−/−^ Rat Stool

**Figure 8:**
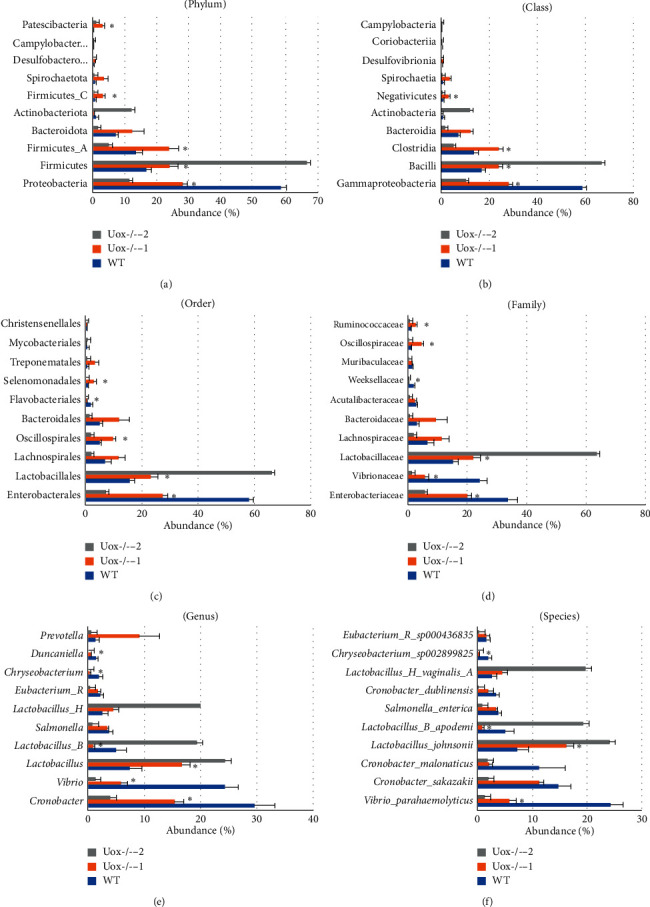
Bacterial abundance of the wild-type (WT) rats and Uox^−/−^ rats' stool at phylum (a), class (b), order (c), family (d), genus (e), and species (f) levels (mean ± SE, *n* = 6). The top 10 bacteria in the WT rats' stool were listed in order, and those in the normal Uox^−/−^ rats (Uox^−/−^−1) and the Uox^−/−^ rats treated with the combination of antibiotics (Uox^−/−^−2, ampicillin (90 mg/kg) and ciprofloxacin (150 mg/kg) for 5 days) were also listed. The top 10 in the Uox^−/−^−2 group were listed without analysis because more than 99% of bacteria were inhibited or killed by antibiotics. (a) The top 10 bacteria at the phylum level covered 99.75% of all bacteria in the WT rats and 99.65% in the normal Uox^−/−^ rats; (b) the top 10 at the class level covered 99.50% in the WT rats and 96.30% in the normal Uox^−/−^ rats; (c) the top 10 at the order level covered 96.29% in the WT rats and 91.61% in the normal Uox^−/−^ rats; (d) the top 10 at the family level covered 92.21% in the WT rats and 80.91% in the normal Uox^−/−^ rats; (e) the top 10 at the genus level covered 80.14% in the WT rats and 58.72% in the normal Uox^−/−^ rats; and (e) the top 10 at the species level covered 76.51% in the WT rats and 48.44% in the normal Uox^−/−^ rats. ^*∗*^*P* < 0.05 vs. WT, Student's *t-*test.

Since the bacterial abundance in stool of Uox^−/−^ rats treated with antibiotics for 5 days dramatically decreased (Figures 4 and 5), about 1% even less than those in the normal Uox^−/−^ rat stool, their bacterial abundance was listed without being analyzed with other two groups. The top 10 bacteria in the wild-type rat stool were listed at phylum (Figure 8(a)), class (Figure 8(b)), order (Figure 8(c)), family (Figure 8(d)), genus (Figure 8(e)), and species (Figure 8(f)) levels, and those in Uox^−/−^ rats were compared at the same levels in the same thumbnails (Figures 8(a)–8(f)). Most of the main bacteria in Uox^−/−^ rat stool were significantly different from those in the wild-type rats.

The top 10 bacteria in the three groups' stool were listed at the genus (Table 1) and species (Table 2) levels. Since the top 10 bacteria accounted for more than 50% of abundance, bacteria could be an important factor maintaining the microecological balance in the intestinal tract.

## 4. Discussion

Bacteria are an essential part of microecology in the gut. They are also regarded as a concerned factor affecting multiple aspects of health, including diabetes mellitus, hypertension, and hyperlipidemia [14]. Bacteria might contribute to hyperuricemia because it has been shown that inoculated engineered bacteria lowered SUA levels [8]. However, most experimental animals with hyperuricemia were established by administered oral uricase inhibitor or high purine diet. The administration could be an additional factor disturbing the aboriginal bacterial flora. The Uox^−/−^ rats naturally develop high SUA levels. The animals could be one of the optimal choices to study the relationship between the SUA level and the aboriginal gut microbiome. The gut microbiome in Uox^−/−^ rodents has not been explored before, and the bacterial flora in the Uox^−/−^ rats was first investigated in this study.

### 4.1. Uricase-Deficiency Alters the Bacterial Flora in Rats

The abundance of bacteria in Uox^−/−^ rat stool was higher than that in the wild-type rats, and the alpha-bacterial diversity increased. The pattern of bacterial flora in the Uox^−/−^ rats was significantly different from that in the wild-type rats. However, the bacterial pattern was further altered if the combination of antibiotics was administered for 5 days. Since the bacteria in fresh stool originated in their host's intestinal tract, the different bacterial patterns in stool largely reflected the bacteria residing in the host's intestinal tract. The difference in bacteria between the stool of Uox^−/−^ rats and that of wild-type rats could originally result from uricase-deficiency and directly from the increase of uric acid in the intestinal tract. Namely, the uricase-deficiency caused the high level of uric acid in the Uox^−/−^ rat gut and then affected the bacteria survival and growth. For instance, uric acid is a possible “food” for some bacteria; hence, uric acid would facilitate their survival and growth. The bacteria in the Uox^−/−^ rat gut could be more likely “uratophilic” bacteria, but they need further research. Several genera in the top 10 bacteria (Table 1) of the Uox^−/−^ rat stool were shown to generate uricase (Table 3), which would facilitate their utilization of uric acid. It should be noted that other bacteria could also generate uricase though needed verification.

### 4.2. Aboriginal Bacteria in Uox^−/−^ Rat Gut Are Not the Main Factor Affecting SUA Levels

High uric acid distributed in the rat intestinal tract was proved to be an important way to lower the SUA level by oral uricase and laxatives [2, 3]. The aboriginal bacteria in the Uox^−/−^ rat intestinal tract can be killed or inhibited by antibiotics such as ampicillin and ciprofloxacin. Since most bacteria can degrade uric acid, it is expected that dramatic killing or inhibition of these bacteria would elevate the SUA levels in Uox^−/−^ rats. However, the expected results were not obtained in the present study, suggesting that the aboriginal bacteria might not be the main factor affecting the SUA level [2]. In contrast, the combination of antibiotics lowered the Uox^−/−^ rat SUA level, which could explained by the mild antibiotic diarrhea [15] because the rats treated with the combination of antibiotics excreted more stool than the normal Uox^−/−^ rats. The mild diarrhea was recovered if the antibiotics were withdrawn for 3 or more days. Interestingly, ampicillin or ciprofloxacin alone did not cause diarrhea in this study.

Ampicillin can lower SUA in humans because, like probenecid, the drug can competitively inhibit uric acid reabsorption in the kidney [16]. However, the effect was not observed in the present study. Indeed, human urate transporter SLC22A11 (OAT4) is the transporter associated with uric acid reclamation and can be inhibited by probenecid and penicillin [17]. However, the transporter is not expressed in the rat' kidney, and probenecid showed a poor effect on the lowering SUA levels in rats [18]. As a quinolone, ciprofloxacin is a substrate of ABCG2 [19], an important transporter facilitating uric acid excretion [20, 21]. ABCG2 is a transporter participating in urate excretion. By hydrolyzing ATP, ABCG2 can actively pump urate to renal tubular lumen [22] or intestinal fluids [23]. ABCG2 deficiency was proved to be a key factor to increase SUA in both human [24] and mice [25]. The two substrates (ciprofloxacin and urate) could compete for the function of ABCG2 and increase the SUA level (Figure 1(d)).

Since fungi normally distributed in the rat intestinal tract have very low abundance [2], the bacteria could be the main microbe to maintain the microecological balance. Though the bacteria may play a crucial role in maintaining human health, the effect of the aboriginal bacteria on the SUA level may be negligible. Exogenous factors such as antibiotics [7], probiotics [15], and prebiotics [26] have been reported to affect SUA levels by inhibiting, supplementing, or promoting bacteria growth, but their effectiveness needs to be further proved in clinical trials. It should be noted that the chemicals and the bacteria would cause aboriginal microecological disturbance. Bacteria are tiny organisms with a high metabolic state in the gut; they synthesize purines besides degrading uric acid, and the purines are released from bacterial bodies. The released purines can be absorbed by their host and transformed into uric acid. Theoretically, uric acid could not be the favorite “food” of microorganisms if nutrients, such as proteins, carbohydrates, and fats are available in the gut, unless the microorganism was an engineered one. It is difficult for us to expect that the aboriginal bacteria can degrade uric acid more powerfully than they synthesize purines, except for the engineered bacteria [8]. However, the engineered microorganisms would theoretically cause an unexpected microecological disturbance.

It should be noted that the bacteria in the wild-type rats' gut were different from those reported recently [7]. This could be due to the regional difference. The experiment of the report [7] was performed in north China, and the present experiment was conducted in southwest China. Nevertheless, similar to the present study, the report [7] stated that the combination of antibiotics (ampicillin, neomycin, and metronidazole) decreased the SUA level, with a weak but a significant effect in “hyperuricemia” rats. Undoubtedly, the results also agreed with the present study in which the aboriginal gut bacteria were not the main factors to lower the SUA level, though most of them expressed uricase to degrade uric acid.

## 5. Conclusions

The bacterial flora in the Uox^−/−^ rat gut was altered, and the gut aboriginal bacteria are not the main factor affecting the SUA level.

## Figures and Tables

**Table 1:** The top 10 genera in rat stool.

No.	WT		Uox^−/−^−1		Uox^−/−^−2^∗^	
	Genus	Abundance (%)	Genus	Abundance (%)	Genus	Abundance (%)
1	*Cronobacter*	29.60	*Lactobacillus*	16.72	*Lactobacillus*	24.39
2	*Vibrio*	24.40	*Cronobacter*	15.44	*Lactobacillus_H*	19.91
3	*Lactobacillus*	7.51	*Prevotella*	9.13	*Lactobacillus_B*	19.37
4	*Lactobacillus_B*	5.08	*Vibrio*	5.85	*Rothia*	10.65
5	*Salmonella*	3.82	*Lactobacillus_H*	4.55	*Cronobacter*	4.00
6	*Lactobacillus_H*	2.68	*Kineothrix*	4.01	*Pseudomonas_E*	2.03
7	*Eubacterium_R*	2.18	*Treponema_D*	3.52	*Vibrio*	1.33
8	*Chryseobacterium*	2.01	*Salmonella*	3.43	*Enterococcus*	1.26
9	*Duncaniella*	1.53	*Schwartzia*	2.96	*Streptococcus*	1.11
10	*Prevotella*	1.35	*Saccharimonas*	2.93	*Saccharimonas*	1.04
Total (267)		80.20	Total (275)	68.50	Total (317)	67.55

^*∗*^More than 99% of bacteria in stool were inhibited or killed in the Uox^−/−^−2 group treated by the combination of antibiotics. WT rats, wild-type rats; Uox^−/−^−1, normal Uox^−/−^ rats; Uox^−/−^−2, Uox^−/−^ rats treated with combinatorial antibiotics (ampicillin (90 mg/kg) and ciprofloxacin (150 mg/kg) for 5 days).

**Table 2:** The top 10 species identified in rat stool.

No.	WT		Uox^−/−^ −1		Uox^−/−^ −2^∗^	
	Species	Abundance (%)	Species	Abundance (%)	Species	Abundance (%)
1	*Vibrio_parahaemolyticus*	24.40	*Lactobacillus_johnsonii*	16.25	*Lactobacillus_johnsonii*	24.15
2	*Cronobacter_sakazakii*	14.84	*Cronobacter_sakazakii*	11.31	*Lactobacillus_H_vaginalis_A*	19.87
3	*Cronobacter_malonaticus*	11.32	*Vibrio_parahaemolyticus*	5.85	*Lactobacillus_B_apodemi*	19.37
4	*Lactobacillus_johnsonii*	7.28	*Prevotella_conceptionensis*	5.40	*Rothia_nasimurium*	10.65
5	*Lactobacillus_B_apodemi*	5.08	*Lactobacillus_H_vaginalis_A*	4.55	*Pseudomonas_E_sihuiensis*	2.00
6	*Salmonella_enterica*	3.82	*Kineothrix_alysoides*	4.00	*Cronobacter_sakazakii*	1.95
7	*Cronobacter_dublinensis*	3.45	*Salmonella_enterica*	3.43	*Cronobacter_malonaticus*	1.85
8	*Lactobacillus_H_vaginalis_A*	2.68	*Schwartzia_succinivorans*	2.96	*Vibrio_parahaemolyticus*	1.335
9	*Chryseobacterium_sp002899825*	2.00	*Saccharimonas_aalborgensis*	2.93	*Enterococcus_rotai*	1.24
10	*Eubacterium_R_sp000436835*	1.66	*Prevotella_copri*	2.73	*Saccharimonas_aalborgensis*	1.04
Total (349)		76.50	Total (361)	59.40	Total (415)	83.45

^*∗*^More than 99% of bacteria in stool were inhibited or killed in the Uox^−/−^−2 group treated by the combination of antibiotics. WT rats, wild-type rats; Uox^−/−^−1, normal Uox^−/−^ rats; Uox^−/−^−2, Uox^−/−^ rats treated with combinatorial antibiotics (ampicillin (90 mg/kg) and ciprofloxacin (150 mg/kg) for 5 days).

**Table 3:** Genera can generate uricase of the top 10 in WT and Uox-/-−1 groups.

No.	Genus	Rank			Uox nucleotide	Uricase protein	Note
		WT	Uox^−/−^−1	Uox^−/−^−2			
1	*Chryseobacterium*	8	—	—	N	N	—
2	*Cronobacter*	1	2	5	Y	N	—
3	*Duncaniella*	9	—		N	N	—
4	*Enterococcus*	—	—	8	N	N	—
5	*Eubacterium_R*	7	—	—	N	N	—
6	*Lactobacillus*	3	1	1	N	Y	Partial aa sequence
7	*Lactobacillus_B*	4		3	N	Y	Partial aa sequence
8	*Lactobacillus_H*	6	5	2	N	Y	Partial aa sequence
9	*Kineothrix*		6		N	N	—
10	*Prevotella*	10	3		N	N	—
11	*Pseudomonas_E*			6	Y	Y	—
12	*Rothia*			4	N	Y	—
13	*Saccharimonas*		10	10	N	N	—
14	*Salmonella*	5	8	—	Y	N	—
15	*Schwartzia*		9	—	N	N	—
16	*Streptococcus*			9	N	Y	—
17	*Treponema_D*		7		N	N	—
18	*Vibrio*		4	7	Y	Y	—

The top 10 genera cited from Table 1. WT, wild-type rats; Uox^−/−^-1, normal Uox^-/--^ rats; Uox^−/-^−2, Uox^−/-^−2 treated with combinatorial antibiotics (ampicillin, 90 mg/kg, and ciprofloxacin, 150 mg/kg, for 5 days), and more than 99% bacteria in the group were inhibited or killed. Note: N, the nucleotide of Uox and uricase amino acid (aa) sequence cannot be presently found in the database (https://www.ncbi.nlm.nih.gov/nucleotide/for Uox and https://www.ncbi.nlm.nih.gov/protein/for uricase); Y, the nucleotide of Uox or uricase amino acid sequence can be found in the database.

## Data Availability

The data used to support the findings of this study are included within the article and Supplementary Materials.

## Supplementary Materials

Supplementary Materials The supplement.docx is the supplementary dataset in table format supporting Figures 1–8.
