# Supplementary material for: Aboriginal Bacterial Flora in the Uricase-Deficient Rat Gut is Not the Main Factor Affecting Serum Uric Acid
Source: Evid Based Complement Alternat Med. 2021 May 18;2021:5587642. doi: 10.1155/2021/5587642 (PMC8154307; doi:10.1155/2021/5587642)
Supplement: Supplementary Materials — The supplement.docx is the supplementary dataset in table format supporting Figures 1–8. [file 5587642.f1.docx]

**Supplement**

**List**

目录

[S1 2](#_Toc66957737)

[Fig 1 A 2](#_Toc66957738)

[Fig 1B 2](#_Toc66957739)

[Fig 1C 3](#_Toc66957740)

[Fig 1D 3](#_Toc66957741)

[S2 3](#_Toc66957742)

[Fig 2A 3](#_Toc66957743)

[Fig 2B 4](#_Toc66957744)

[Fig 2C 4](#_Toc66957745)

[Fig 2D 5](#_Toc66957746)

[S3 5](#_Toc66957747)

[Fig 3A 5](#_Toc66957748)

[Fig 3B 5](#_Toc66957749)

[Fig 3C 6](#_Toc66957750)

[Fig 3D 6](#_Toc66957751)

[S4 6](#_Toc66957752)

[S5 7](#_Toc66957753)

[Fig 5B 7](#_Toc66957754)

[Fig 5C 8](#_Toc66957755)

[Fig 5D 8](#_Toc66957756)

[Fig 5E 9](#_Toc66957757)

[Fig 5F 9](#_Toc66957758)

[S6 11](#_Toc66957759)

[OTUs count for Fig 6A and B 11](#_Toc66957760)

[genus count for Fig 6C 49](#_Toc66957761)

[Species count for Fig 6D 63](#_Toc66957762)

[S7 82](#_Toc66957763)

[OTUs count for Fig 7L 82](#_Toc66957764)

[genus count for Fig 7R 120](#_Toc66957765)

[S8 134](#_Toc66957766)

[S8.1（Fig8A） 134](#_Toc66957767)

[S8.2（Fig 8B） 136](#_Toc66957768)

[S8.3 （Fig 8C） 138](#_Toc66957769)

[S8.4 （Fig 8D） 142](#_Toc66957770)

[S8.5 （Fig 8E） 149](#_Toc66957771)

[S8.6（Fig 8F） 163](#_Toc66957772)

# S1

## Fig 1 A

| ug/ml | Day0 | Day5 |
| --- | --- | --- |
| 1 | 56.44 | 30.95652 |
| 2 | 51.24 | 45.30435 |
| 3 | 54.44 | 53.56522 |
| 4 | 46.44 | 43.56522 |
| 5 | 40.84 | 41.6087 |
| 6 | 44.04 | 34.21739 |
| 7 | 39.83 | 35.95652 |
| 8 | 38.96 | 39.86957 |
| 9 | 36.76 | 58.47368 |
| 10 | 38.21 | 36.36842 |
| 11 | 42.54 | 54.78947 |
| 12 | 52.66 | 51.10526 |
|  |  |  |
| mean | 45.20 | 43.82 |
| SD | 6.880044 | 8.948882 |
| P |  | 0.680005 |

## Fig 1B

| ug/ml | Day0 | Day5 |
| --- | --- | --- |
| 1 | 59.29 | 52.62 |
| 2 | 58.21 | 50.12 |
| 3 | 58.08 | 50.69 |
| 4 | 72.94 | 48.00 |
| 5 | 71.38 | 43.38 |
| 6 | 73.18 | 60.88 |
|  |  |  |
| mean | 65.51 | 50.95 |
| SD | 7.689915 | 5.799493 |
| P |  | 0.013082 |

## Fig 1C

| ug/ml | Day0 | Day5 |
| --- | --- | --- |
| 1 | 47.95 | 50.72222 |
| 2 | 53.41 | 32.94444 |
| 3 | 58.18 | 34.61111 |
| 4 | 54.09 | 24.88889 |
| 5 | 37.05 | 38.77778 |
| 6 | 50 | 70.72222 |
|  |  |  |
| mean | 50.11 | 42.11 |
| SD | 7.308851 | 16.37523 |
| P |  | 0.35881 |

## Fig 1D

| ug/ml | Day0 | Day5 |
| --- | --- | --- |
| 1 | 49.09 | 55.72222 |
| 2 | 47.73 | 70.16667 |
| 3 | 44.73 | 83.22222 |
| 4 | 47.81 | 66.27778 |
| 5 | 40.5 | 54.05556 |
| 6 | 40.88 | 42.38889 |
|  |  |  |
| mean | 45.12 | 61.97 |
| SD | 3.722449 | 14.3003 |
| P |  | 0.025092 |

# S2

## Fig 2A

| g | Day0 | Day5 |
| --- | --- | --- |
| 1 | 29.5 | 27.1 |
| 2 | 29.5 | 31.8 |
| 3 | 26.0 | 29.9 |
| 4 | 22.8 | 20.7 |
| 5 | 27.2 | 27.0 |
| 6 | 25.0 | 26.0 |
|  |  |  |
| mean | 26.67 | 27.08 |
| SD | 2.627293 | 3.797587 |
| P |  | 0.697338 |

## Fig 2B

| ml | Day0 | Day5 |
| --- | --- | --- |
| 1 | 69.4 | 76.9 |
| 2 | 61.8 | 74.4 |
| 3 | 53.5 | 62.1 |
| 4 | 35.7 | 58.3 |
| 5 | 53.8 | 56.8 |
| 6 | 69.6 | 63.1 |
|  |  |  |
| mean | 57.30 | 65.27 |
| SD | 12.73735 | 8.410628 |
| P |  | 0.100342 |

## Fig 2C

| ml | Day0 | Day5 |
| --- | --- | --- |
| 1 | 49.10 | 46.00 |
| 2 | 41.60 | 45.60 |
| 3 | 36.70 | 41.50 |
| 4 | 28.80 | 33.70 |
| 5 | 32.40 | 28.10 |
| 6 | 48.80 | 41.40 |
|  |  |  |
| mean | 39.57 | 39.38 |
| SD | 8.435323 | 7.079383 |
| P |  | 0.93693 |

## Fig 2D

| g | Day0 | Day5 |
| --- | --- | --- |
| 1 | 13.00 | 27.40 |
| 2 | 12.60 | 31.00 |
| 3 | 13.00 | 29.60 |
| 4 | 9.30 | 18.00 |
| 5 | 9.90 | 23.60 |
| 6 | 12.20 | 22.10 |
|  |  |  |
| mean | 11.67 | 25.28 |
| SD | 1.639105 | 4.935754 |
| P |  | 0.0003 |

# S3

## Fig 3A

| ug/ml | Day0 | Day5 |
| --- | --- | --- |
| 1 | 397.50 | 125.38 |
| 2 | 580.83 | 544.62 |
| 3 | 193.33 | 621.54 |
| 4 | 172.50 | 990.77 |
| 5 | 639.17 | 879.23 |
| 6 | 622.50 | 698.46 |
|  |  |  |
| mean | 434.31 | 643.33 |
| SD | 212.9883 | 302.6184 |
| P |  | 0.237785 |

## Fig 3B

| ug/g | Day0 | Day5 |
| --- | --- | --- |
| 1 | 149.50 | 182.77 |
| 2 | 131.17 | 227.38 |
| 3 | 117.83 | 174.31 |
| 4 | 133.67 | 138.92 |
| 5 | 160.33 | 264.31 |
| 6 | 119.50 | 169.69 |
|  |  |  |
| mean | 135.33 | 192.90 |
| SD | 16.74979 | 45.14124 |
| P |  | 0.013152 |

## Fig 3C

| mg | Day0 | Day5 |
| --- | --- | --- |
| 1 | 19.52 | 5.77 |
| 2 | 24.16 | 24.83 |
| 3 | 7.10 | 25.79 |
| 4 | 4.97 | 33.39 |
| 5 | 20.71 | 24.71 |
| 6 | 30.38 | 28.92 |
|  |  |  |
| mean | 17.81 | 23.90 |
| SD | 9.892935 | 9.477275 |
| P |  | 0.368492 |

## Fig 3D

| mg | Day0 | Day5 |
| --- | --- | --- |
| 1 | 1.94 | 2.38 |
| 2 | 1.65 | 2.87 |
| 3 | 1.53 | 2.27 |
| 4 | 1.24 | 1.29 |
| 5 | 1.59 | 2.62 |
| 6 | 1.46 | 2.07 |
|  |  |  |
| mean | 1.57 | 2.25 |
| SD | 0.231312 | 0.543859 |
| P |  | 0.01056 |

# S4

Original clones

| clones/dish | WT | Uox-/--1 | Uox-/--2 |
| --- | --- | --- | --- |
| 1 | 684 | 171 | 63 |
| 2 | 1789 | 1329 | 3 |
| 3 | 643 | 749 | 8 |
| 4 | 1915 | 2680 | 4 |
| 5 | 1013 | 1671 | 1 |
| 6 | 351 | 1172 | 1 |

| clones/(g stool) | WT | Uox-/--1 | Uox-/--2 |
| --- | --- | --- | --- |
| 1 | 68400000000 | 1.71E+10 | 6300000000 |
| 2 | 1.789E+11 | 1.33E+11 | 300000000 |
| 3 | 64300000000 | 7.49E+10 | 800000000 |
| 4 | 1.915E+11 | 2.68E+11 | 400000000 |
| 5 | 1.013E+11 | 1.67E+11 | 100000000 |
| 6 | 35100000000 | 1.17E+11 | 100000000 |

Fig 4D

| Log (n/g) | WT | Uox-/--1 | Uox-/--2 |
| --- | --- | --- | --- |
| 1 | 10.8350561 | 10.233 | 9.79934055 |
| 2 | 11.25261034 | 11.12352 | 8.47712125 |
| 3 | 10.80821097 | 10.87448 | 8.90308999 |
| 4 | 11.28216878 | 11.42813 | 8.60205999 |
| 5 | 11.00560945 | 11.22298 | 8 |
| 6 | 10.54530712 | 11.06893 | 8 |
| mean | 10.95482713 | 10.99184 | 8.63026863 |
| SD | 0.283532812 | 0.413927 | 0.67229273 |
| P vs A |  | 0.86021 | 1.4625E-05 |
| P vs B |  |  | 1.4625E-05 |

# S5

## Fig 5B

| ng/ul | WT | Uox-/--1 | Uox-/--2 |
| --- | --- | --- | --- |
| 1 | 58.62 | 78.8 | 0.05 |
| 2 | 87.65 | 101.62 | 0.05 |
| 3 | 51.88 | 94.92 | 84.37 |
| 4 | 83.23 | 96.7 | 1.81 |
| 5 | 84.61 | 98.6 | 0.05 |
| 6 | 94.24 | 95.86 | 0.05 |
| mean | 76.705 | 94.41667 | 14.39667 |
| SD | 17.17953 | 8.010228 | 34.28702 |
| vs WT |  | 0.045106 | 0.002602 |
| VsUox-/--1 | |  | 0.000238 |

## Fig 5C

| rat | DNA(ng/ul) | Log clones |
| --- | --- | --- |
| WT1 | 58.62 | 10.84 |
| WT2 | 87.65 | 11.25 |
| WT3 | 51.88 | 10.81 |
| WT4 | 83.23 | 11.28 |
| WT5 | 84.61 | 11.01 |
| WT6 | 94.24 | 10.54531 |
| Uox-/--1-1 | 78.8 | 10.233 |
| Uox-/--1-2 | 101.62 | 11.12352 |
| Uox-/--1-3 | 94.92 | 10.87448 |
| Uox-/--1-4 | 96.7 | 11.42813 |
| Uox-/--1-5 | 98.6 | 11.22298 |
| Uox-/--1-6 | 95.86 | 11.06893 |
| Uox-/--2-1 | 0.05 | 9.799341 |
| Uox-/--2-2 | 0.05 | 8.477121 |
| Uox-/--2-3 | 84.37 | 8.90 |
| Uox-/--2-4 | 1.81 | 8.60206 |
| Uox-/--2-5 | 0.05 | 8 |
| Uox-/--2-6 | 0.05 | 8 |

## Fig 5D

| CT | WT | Uox-/--1 | Uox-/--2 |
| --- | --- | --- | --- |
| 1 | 15.26039 | 14.72699 | 19.75924 |
| 2 | 13.60014 | 13.11007 | 19.57993 |
| 3 | 15.15205 | 13.29529 | 10.73658 |
| 4 | 13.64643 | 13.37888 | 18.24263 |
| 5 | 13.19018 | 13.32275 | 23.51648 |
| 6 | 13.75198 | 13.97357 | 19.6609 |
| mean | 14.10019 | 13.63459 | 18.58263 |
| SD | 0.878384 | 0.610104 | 4.232272 |
| vs WT |  | 0.311313 | 0.029356 |
| VsUox-/--1 | |  | 0.017716 |

## Fig 5E

| log(copies) | WT | Uox-/--1 | Uox-/--2 |
| --- | --- | --- | --- |
| 1 | 9.515011 | 9.666204 | 7.540822 |
| 2 | 9.985616 | 10.12453 | 7.591648 |
| 3 | 9.54572 | 10.07203 | 10.09834 |
| 4 | 9.972496 | 10.04833 | 8.66968 |
| 5 | 10.10182 | 10.06424 | 6.475812 |
| 6 | 9.942578 | 9.879767 | 7.568696 |
| mean | 9.84 | 9.98 | 7.99 |
| SD | 0.248982 | 0.172937 | 1.244006 |
| vs WT |  | 0.311313 | 0.005031 |
| VsUox-/--1 | |  | 0.003102 |

## Fig 5F

| rat | CT | Log clones |
| --- | --- | --- |
| WT1 | 15.26039 | 10.84 |
| WT2 | 13.60014 | 11.25 |
| WT3 | 15.15205 | 10.81 |
| WT4 | 13.64643 | 11.28 |
| WT5 | 13.19018 | 11.01 |
| WT6 | 13.75198 | 10.55 |
| Uox-/--1-1 | 14.72699 | 10.233 |
| Uox-/--1-2 | 13.11007 | 11.12352 |
| Uox-/--1-3 | 13.29529 | 10.87448 |
| Uox-/--1-4 | 13.37888 | 11.42813 |
| Uox-/--1-5 | 13.32275 | 11.22298 |
| Uox-/--1-6 | 13.97357 | 11.06893 |
| Uox-/--2-1 | 19.75924 | 9.799341 |
| Uox-/--2-2 | 19.57993 | 8.477121 |
| Uox-/--2-3 | 10.73658 | 8.90309 |
| Uox-/--2-4 | 18.24263 | 8.60 |
| Uox-/--2-5 | 23.51648 | 8 |
| Uox-/--2-6 | 19.6609 | 8 |

# S6

## OTUs count for Fig 6A and B

| otu | WT1 | WT2 | WT3 | WT4 | WT5 | WT6 | Uox-/--1-1 | Uox-/--1-2 | Uox-/--1-3 | Uox-/--1-4 | Uox-/--1-5 | Uox-/--1-6 | Uox-/--2-1 | Uox-/--2-2 | Uox-/--2-3 | Uox-/--2-4 | Uox-/--2-5 | Uox-/--2-6 |
| --- | --- | --- | --- | --- | --- | --- | --- | --- | --- | --- | --- | --- | --- | --- | --- | --- | --- | --- |
| OTU1 | 5167 | 1361 | 2374 | 4451 | 1528 | 1256 | 6036 | 6655 | 5114 | 8144 | 4276 | 5343 | 10474 | 1837 | 9951 | 7392 | 846 | 34899 |
| OTU10 | 1864 | 610 | 720 | 546 | 1298 | 1372 | 91 | 128 | 205 | 101 | 85 | 114 | 13 | 46 | 155 | 0 | 11 | 10 |
| OTU100 | 0 | 6 | 8 | 0 | 1 | 5 | 0 | 50 | 138 | 17 | 89 | 195 | 15 | 20 | 86 | 0 | 8 | 1 |
| OTU101 | 3 | 32 | 0 | 20 | 599 | 7 | 3 | 46 | 16 | 128 | 32 | 5 | 1 | 2 | 17 | 0 | 3 | 2 |
| OTU102 | 0 | 1 | 1 | 0 | 0 | 0 | 1 | 27 | 93 | 1 | 0 | 205 | 20 | 117 | 62 | 0 | 1 | 0 |
| OTU103 | 166 | 102 | 283 | 215 | 83 | 230 | 88 | 114 | 47 | 92 | 42 | 92 | 10 | 26 | 44 | 0 | 9 | 5 |
| OTU104 | 1 | 0 | 1 | 2 | 0 | 1 | 163 | 45 | 77 | 59 | 131 | 79 | 8 | 22 | 111 | 0 | 2 | 1 |
| OTU105 | 6 | 1 | 369 | 0 | 1 | 1 | 8 | 0 | 3 | 7 | 1 | 19 | 1 | 3 | 11 | 1 | 0 | 0 |
| OTU106 | 0 | 0 | 1 | 0 | 2 | 1 | 32 | 30 | 105 | 57 | 37 | 100 | 9 | 29 | 35 | 1 | 2 | 1 |
| OTU107 | 92 | 18 | 105 | 46 | 22 | 283 | 0 | 1 | 0 | 1 | 0 | 0 | 0 | 0 | 1 | 0 | 0 | 2 |
| OTU108 | 1 | 2 | 0 | 0 | 0 | 2 | 352 | 13 | 85 | 4 | 4 | 162 | 13 | 80 | 17 | 0 | 0 | 2 |
| OTU109 | 74 | 33 | 33 | 83 | 31 | 129 | 14 | 8 | 14 | 13 | 16 | 9 | 4 | 11 | 19 | 0 | 0 | 1 |
| OTU11 | 1 | 1 | 1 | 0 | 0 | 2 | 51 | 607 | 2248 | 1188 | 531 | 793 | 5 | 18 | 892 | 0 | 16 | 5 |
| OTU110 | 0 | 0 | 2 | 0 | 3 | 0 | 41 | 17 | 46 | 112 | 51 | 108 | 6 | 17 | 39 | 0 | 2 | 1 |
| OTU111 | 0 | 1 | 1 | 0 | 0 | 0 | 2 | 46 | 76 | 51 | 16 | 64 | 0 | 0 | 126 | 0 | 4 | 2 |
| OTU112 | 24 | 40 | 47 | 14 | 34 | 41 | 20 | 47 | 52 | 45 | 60 | 44 | 34 | 24 | 34 | 17 | 77 | 23 |
| OTU113 | 75 | 26 | 0 | 8 | 1 | 407 | 0 | 2 | 3 | 1 | 1 | 2 | 0 | 0 | 0 | 0 | 2 | 0 |
| OTU114 | 19 | 78 | 196 | 70 | 91 | 18 | 0 | 0 | 0 | 1 | 0 | 1 | 0 | 1 | 0 | 0 | 0 | 0 |
| OTU115 | 1 | 1 | 1 | 1 | 2 | 1 | 0 | 2 | 3 | 2 | 0 | 0 | 26 | 19 | 2 | 6 | 293 | 159 |
| OTU116 | 35 | 3 | 8 | 67 | 1 | 30 | 44 | 82 | 107 | 3 | 2 | 46 | 6 | 24 | 94 | 0 | 13 | 2 |
| OTU117 | 8 | 159 | 58 | 0 | 0 | 1 | 163 | 42 | 108 | 63 | 328 | 73 | 26 | 53 | 79 | 0 | 9 | 8 |
| OTU118 | 3 | 18 | 9 | 20 | 12 | 215 | 8 | 51 | 15 | 123 | 60 | 15 | 0 | 2 | 14 | 0 | 4 | 2 |
| OTU119 | 2 | 1 | 1 | 0 | 0 | 1 | 80 | 13 | 1 | 1 | 35 | 203 | 2 | 1 | 0 | 0 | 1 | 0 |
| OTU12 | 1 | 4 | 0 | 1 | 2 | 4 | 341 | 2197 | 617 | 1461 | 1392 | 565 | 76 | 234 | 316 | 3 | 54 | 20 |
| OTU120 | 75 | 118 | 21 | 111 | 162 | 210 | 3 | 1 | 2 | 1 | 2 | 2 | 1 | 1 | 0 | 0 | 0 | 0 |
| OTU121 | 0 | 0 | 0 | 0 | 0 | 0 | 0 | 0 | 0 | 0 | 0 | 0 | 0 | 0 | 0 | 0 | 297 | 0 |
| OTU122 | 34 | 26 | 69 | 69 | 158 | 192 | 1 | 4 | 0 | 25 | 0 | 1 | 0 | 2 | 0 | 0 | 0 | 1 |
| OTU123 | 1 | 0 | 2 | 1 | 3 | 0 | 78 | 20 | 36 | 40 | 29 | 87 | 6 | 10 | 53 | 0 | 0 | 0 |
| OTU124 | 1 | 2 | 1 | 3 | 1 | 5 | 40 | 103 | 25 | 58 | 83 | 80 | 7 | 30 | 26 | 0 | 2 | 6 |
| OTU125 | 9 | 14 | 4 | 5 | 348 | 9 | 1 | 0 | 0 | 0 | 0 | 1 | 0 | 0 | 0 | 0 | 0 | 0 |
| OTU126 | 0 | 0 | 2 | 0 | 1 | 1 | 133 | 25 | 13 | 86 | 32 | 32 | 9 | 13 | 8 | 0 | 1 | 1 |
| OTU127 | 2 | 3 | 73 | 14 | 4 | 75 | 8 | 61 | 20 | 50 | 384 | 15 | 8 | 4 | 18 | 1 | 4 | 6 |
| OTU128 | 1 | 99 | 6 | 28 | 0 | 180 | 31 | 28 | 36 | 7 | 14 | 13 | 3 | 20 | 46 | 0 | 4 | 1 |
| OTU129 | 1 | 2 | 6 | 3 | 0 | 22 | 3 | 31 | 12 | 265 | 35 | 34 | 1 | 3 | 55 | 0 | 1 | 2 |
| OTU13 | 2 | 14 | 13 | 3 | 10 | 137 | 5 | 505 | 1538 | 23 | 961 | 862 | 23 | 53 | 939 | 2 | 49 | 31 |
| OTU130 | 0 | 2 | 0 | 0 | 0 | 1 | 42 | 78 | 22 | 25 | 136 | 84 | 3 | 6 | 27 | 0 | 8 | 4 |
| OTU131 | 0 | 2 | 2 | 1 | 5 | 9 | 46 | 221 | 457 | 396 | 203 | 141 | 6 | 12 | 362 | 0 | 19 | 10 |
| OTU132 | 1 | 2 | 2 | 3 | 0 | 1 | 0 | 1 | 3 | 2 | 1 | 2 | 111 | 154 | 1 | 6 | 461 | 242 |
| OTU133 | 7 | 31 | 7 | 10 | 22 | 2 | 7 | 75 | 56 | 21 | 52 | 68 | 2 | 11 | 56 | 0 | 6 | 1 |
| OTU134 | 108 | 63 | 72 | 107 | 113 | 203 | 4 | 1 | 1 | 1 | 1 | 3 | 0 | 0 | 0 | 0 | 1 | 0 |
| OTU135 | 2 | 0 | 0 | 1 | 2 | 1 | 766 | 15 | 10 | 49 | 100 | 50 | 1 | 7 | 10 | 0 | 0 | 0 |
| OTU136 | 0 | 0 | 0 | 0 | 0 | 0 | 19 | 3 | 17 | 0 | 204 | 0 | 1 | 0 | 19 | 0 | 0 | 0 |
| OTU137 | 1 | 1 | 2 | 1 | 0 | 0 | 63 | 21 | 48 | 30 | 34 | 76 | 1 | 9 | 59 | 0 | 0 | 0 |
| OTU138 | 29 | 168 | 39 | 7 | 13 | 28 | 51 | 59 | 110 | 44 | 137 | 51 | 11 | 16 | 192 | 0 | 15 | 15 |
| OTU139 | 45 | 21 | 229 | 19 | 42 | 16 | 0 | 0 | 5 | 5 | 10 | 4 | 19 | 10 | 3 | 3 | 78 | 9 |
| OTU14 | 9 | 3 | 7 | 8 | 13 | 13 | 1736 | 826 | 463 | 895 | 651 | 1178 | 73 | 294 | 302 | 1 | 62 | 33 |
| OTU140 | 12 | 32 | 23 | 9 | 100 | 27 | 11 | 105 | 29 | 57 | 7 | 13 | 2 | 3 | 17 | 0 | 3 | 0 |
| OTU141 | 14 | 22 | 16 | 6 | 233 | 105 | 3 | 59 | 15 | 31 | 29 | 14 | 0 | 9 | 5 | 1 | 1 | 1 |
| OTU142 | 0 | 160 | 39 | 0 | 73 | 10 | 7 | 0 | 0 | 1 | 11 | 1 | 3 | 0 | 1 | 0 | 0 | 0 |
| OTU143 | 73 | 0 | 3 | 0 | 1 | 2 | 171 | 24 | 15 | 2 | 1 | 111 | 5 | 51 | 34 | 0 | 0 | 0 |
| OTU144 | 0 | 1 | 1 | 0 | 1 | 0 | 92 | 56 | 36 | 36 | 63 | 47 | 7 | 16 | 41 | 0 | 1 | 0 |
| OTU145 | 10 | 13 | 23 | 21 | 28 | 62 | 11 | 162 | 96 | 164 | 145 | 55 | 5 | 6 | 94 | 0 | 9 | 2 |
| OTU146 | 246 | 16 | 83 | 100 | 65 | 132 | 0 | 0 | 0 | 1 | 2 | 1 | 0 | 0 | 2 | 0 | 2 | 2 |
| OTU147 | 1 | 74 | 51 | 19 | 7 | 10 | 6 | 100 | 31 | 197 | 60 | 11 | 2 | 2 | 31 | 0 | 3 | 2 |
| OTU148 | 28 | 2 | 74 | 55 | 48 | 365 | 4 | 58 | 29 | 55 | 132 | 6 | 7 | 2 | 25 | 1 | 4 | 4 |
| OTU149 | 0 | 2 | 1 | 0 | 1 | 1 | 7 | 54 | 71 | 12 | 26 | 15 | 0 | 0 | 56 | 0 | 5 | 3 |
| OTU15 | 0 | 2 | 0 | 2 | 3 | 1 | 1 | 10 | 7 | 1 | 13 | 6 | 1 | 2 | 4 | 2 | 3834 | 2 |
| OTU150 | 0 | 0 | 1 | 0 | 0 | 0 | 494 | 0 | 1 | 0 | 0 | 0 | 0 | 0 | 0 | 0 | 0 | 0 |
| OTU151 | 41 | 41 | 85 | 35 | 21 | 47 | 3 | 17 | 8 | 4 | 7 | 5 | 1 | 3 | 4 | 0 | 2 | 5 |
| OTU152 | 5 | 26 | 34 | 38 | 59 | 43 | 3 | 75 | 27 | 74 | 87 | 44 | 5 | 8 | 10 | 0 | 5 | 0 |
| OTU153 | 0 | 0 | 0 | 0 | 0 | 0 | 0 | 0 | 0 | 0 | 0 | 0 | 34 | 18 | 0 | 5 | 95 | 127 |
| OTU154 | 0 | 0 | 0 | 0 | 1 | 0 | 0 | 0 | 0 | 0 | 0 | 1 | 1 | 0 | 0 | 0 | 236 | 1 |
| OTU155 | 45 | 49 | 53 | 26 | 20 | 76 | 0 | 0 | 0 | 0 | 0 | 0 | 0 | 0 | 0 | 0 | 0 | 0 |
| OTU156 | 0 | 1 | 0 | 0 | 1 | 0 | 63 | 24 | 5 | 8 | 24 | 24 | 1 | 17 | 19 | 0 | 0 | 0 |
| OTU157 | 1 | 1 | 284 | 0 | 2 | 5 | 6 | 1 | 2 | 1 | 6 | 0 | 0 | 0 | 2 | 0 | 1 | 0 |
| OTU158 | 0 | 0 | 1 | 1 | 2 | 0 | 33 | 30 | 34 | 1 | 2 | 86 | 9 | 34 | 33 | 0 | 5 | 1 |
| OTU159 | 3 | 4 | 19 | 5 | 647 | 55 | 1 | 46 | 23 | 48 | 43 | 22 | 0 | 3 | 24 | 0 | 4 | 5 |
| OTU16 | 0 | 2 | 4 | 2 | 3 | 9 | 141 | 413 | 755 | 684 | 654 | 1045 | 50 | 204 | 530 | 2 | 75 | 28 |
| OTU160 | 1 | 6 | 1 | 0 | 1 | 2 | 75 | 195 | 114 | 396 | 465 | 101 | 18 | 19 | 99 | 1 | 16 | 10 |
| OTU161 | 118 | 2 | 1 | 1 | 0 | 3 | 173 | 31 | 10 | 49 | 29 | 50 | 4 | 22 | 22 | 0 | 2 | 4 |
| OTU162 | 18 | 20 | 19 | 6 | 16 | 20 | 13 | 28 | 26 | 26 | 42 | 32 | 8 | 10 | 19 | 1 | 28 | 7 |
| OTU163 | 0 | 0 | 0 | 0 | 0 | 0 | 30 | 16 | 41 | 7 | 16 | 76 | 0 | 2 | 36 | 0 | 4 | 0 |
| OTU164 | 88 | 1 | 30 | 0 | 0 | 0 | 56 | 95 | 31 | 12 | 2 | 5 | 0 | 2 | 23 | 0 | 1 | 2 |
| OTU165 | 457 | 35 | 75 | 25 | 194 | 21 | 208 | 81 | 61 | 103 | 51 | 92 | 12 | 64 | 72 | 0 | 10 | 9 |
| OTU166 | 0 | 0 | 2 | 0 | 0 | 2 | 197 | 6 | 43 | 21 | 26 | 40 | 0 | 4 | 48 | 0 | 2 | 1 |
| OTU167 | 0 | 0 | 6 | 0 | 0 | 1 | 0 | 6 | 1 | 189 | 84 | 0 | 0 | 0 | 1 | 0 | 6 | 1 |
| OTU168 | 96 | 13 | 32 | 42 | 107 | 66 | 0 | 1 | 0 | 1 | 1 | 1 | 0 | 0 | 1 | 0 | 0 | 0 |
| OTU169 | 29 | 67 | 88 | 31 | 58 | 78 | 40 | 83 | 49 | 74 | 90 | 67 | 40 | 55 | 52 | 35 | 157 | 42 |
| OTU17 | 76 | 2145 | 1778 | 108 | 38 | 1061 | 1201 | 190 | 131 | 8 | 6 | 538 | 20 | 80 | 139 | 1 | 21 | 5 |
| OTU170 | 1 | 1 | 0 | 0 | 1 | 1 | 266 | 7 | 7 | 5 | 57 | 5 | 0 | 2 | 5 | 0 | 0 | 1 |
| OTU171 | 0 | 0 | 0 | 0 | 0 | 0 | 0 | 2 | 16 | 43 | 119 | 25 | 0 | 0 | 14 | 0 | 7 | 0 |
| OTU172 | 50 | 16 | 28 | 33 | 89 | 77 | 1 | 0 | 0 | 6 | 0 | 1 | 0 | 0 | 0 | 0 | 2 | 1 |
| OTU173 | 1 | 6 | 2 | 3 | 1 | 1 | 5 | 53 | 19 | 32 | 67 | 13 | 0 | 1 | 15 | 0 | 3 | 1 |
| OTU174 | 1 | 3 | 1 | 2 | 9 | 9 | 4 | 63 | 32 | 23 | 27 | 21 | 5 | 7 | 25 | 0 | 2 | 2 |
| OTU175 | 3 | 0 | 3 | 1 | 1 | 4 | 63 | 26 | 39 | 52 | 32 | 36 | 2 | 11 | 36 | 0 | 1 | 0 |
| OTU176 | 1 | 1 | 6 | 0 | 1 | 5 | 1 | 4 | 3 | 154 | 119 | 30 | 0 | 6 | 4 | 0 | 1 | 0 |
| OTU177 | 11 | 9 | 3 | 10 | 7 | 8 | 8 | 18 | 15 | 7 | 9 | 9 | 1 | 18 | 16 | 0 | 0 | 0 |
| OTU178 | 8 | 77 | 37 | 0 | 7 | 1 | 0 | 0 | 0 | 0 | 0 | 0 | 0 | 0 | 0 | 0 | 0 | 1 |
| OTU179 | 104 | 12 | 82 | 2 | 1 | 17 | 194 | 26 | 44 | 6 | 1 | 51 | 4 | 29 | 49 | 0 | 1 | 1 |
| OTU18 | 4 | 8 | 21 | 16 | 596 | 1155 | 19 | 39 | 506 | 100 | 55 | 368 | 8 | 23 | 209 | 1 | 3 | 2 |
| OTU180 | 0 | 0 | 0 | 0 | 0 | 0 | 24 | 32 | 40 | 30 | 73 | 29 | 9 | 22 | 60 | 0 | 4 | 1 |
| OTU181 | 0 | 0 | 0 | 0 | 0 | 0 | 1 | 3 | 11 | 30 | 2 | 57 | 4 | 6 | 20 | 0 | 0 | 0 |
| OTU182 | 3 | 6 | 6 | 2 | 29 | 2 | 13 | 87 | 30 | 87 | 84 | 29 | 2 | 6 | 18 | 0 | 3 | 2 |
| OTU183 | 25 | 63 | 64 | 53 | 115 | 41 | 2 | 0 | 0 | 0 | 1 | 1 | 0 | 1 | 0 | 0 | 3 | 0 |
| OTU184 | 159 | 2 | 47 | 0 | 10 | 7 | 0 | 0 | 0 | 0 | 0 | 0 | 0 | 0 | 0 | 0 | 0 | 0 |
| OTU185 | 15 | 53 | 34 | 22 | 71 | 73 | 18 | 63 | 62 | 81 | 72 | 35 | 5 | 13 | 39 | 0 | 1 | 0 |
| OTU186 | 1 | 0 | 3 | 0 | 1 | 1 | 23 | 122 | 3 | 1 | 23 | 5 | 1 | 0 | 1 | 0 | 1 | 0 |
| OTU187 | 24 | 30 | 16 | 84 | 69 | 53 | 51 | 371 | 208 | 208 | 168 | 252 | 7 | 13 | 117 | 1 | 11 | 7 |
| OTU188 | 9 | 1 | 14 | 0 | 0 | 9 | 0 | 12 | 52 | 2 | 7 | 217 | 1 | 12 | 42 | 0 | 2 | 0 |
| OTU189 | 4 | 9 | 8 | 1 | 71 | 72 | 2 | 1 | 8 | 9 | 14 | 14 | 0 | 2 | 5 | 0 | 3 | 0 |
| OTU19 | 29 | 277 | 124 | 17 | 380 | 177 | 14 | 50 | 46 | 27 | 6 | 12 | 561 | 1157 | 59 | 1524 | 11 | 2 |
| OTU190 | 86 | 25 | 85 | 53 | 31 | 65 | 14 | 2 | 1 | 8 | 3 | 13 | 0 | 4 | 6 | 0 | 1 | 1 |
| OTU191 | 0 | 3 | 0 | 2 | 410 | 5 | 1 | 5 | 3 | 4 | 3 | 1 | 0 | 0 | 4 | 0 | 1 | 1 |
| OTU192 | 2 | 1 | 0 | 1 | 1 | 1 | 164 | 3 | 12 | 60 | 56 | 1 | 1 | 2 | 13 | 0 | 7 | 4 |
| OTU193 | 11 | 8 | 58 | 0 | 0 | 11 | 116 | 89 | 22 | 102 | 131 | 88 | 17 | 30 | 26 | 1 | 5 | 7 |
| OTU194 | 9 | 13 | 146 | 4 | 2 | 20 | 0 | 0 | 0 | 0 | 0 | 1 | 0 | 0 | 0 | 0 | 0 | 1 |
| OTU195 | 125 | 5 | 24 | 105 | 0 | 74 | 36 | 21 | 14 | 10 | 8 | 4 | 2 | 6 | 20 | 0 | 3 | 1 |
| OTU196 | 42 | 9 | 37 | 22 | 40 | 56 | 1 | 6 | 6 | 13 | 4 | 0 | 0 | 0 | 3 | 0 | 0 | 0 |
| OTU197 | 0 | 0 | 1 | 1 | 1 | 2 | 20 | 2 | 21 | 20 | 26 | 35 | 8 | 19 | 9 | 0 | 1 | 1 |
| OTU198 | 2 | 3 | 5 | 6 | 6 | 4 | 56 | 25 | 80 | 54 | 6 | 33 | 2 | 13 | 121 | 0 | 6 | 1 |
| OTU199 | 1 | 0 | 1 | 2 | 0 | 1 | 22 | 9 | 12 | 52 | 29 | 23 | 1 | 5 | 19 | 0 | 0 | 0 |
| OTU2 | 381 | 3229 | 1307 | 215 | 4385 | 2353 | 184 | 675 | 492 | 406 | 103 | 144 | 6828 | 16355 | 677 | 20497 | 208 | 107 |
| OTU20 | 3 | 2 | 1 | 1 | 1 | 3 | 53 | 149 | 730 | 349 | 177 | 328 | 9 | 37 | 378 | 0 | 21 | 12 |
| OTU200 | 2 | 0 | 0 | 3 | 2 | 0 | 125 | 23 | 65 | 41 | 34 | 46 | 5 | 10 | 49 | 0 | 3 | 3 |
| OTU201 | 0 | 0 | 1 | 1 | 0 | 0 | 116 | 45 | 34 | 61 | 29 | 85 | 5 | 10 | 21 | 0 | 7 | 3 |
| OTU202 | 0 | 0 | 1 | 0 | 0 | 0 | 25 | 11 | 20 | 85 | 57 | 27 | 1 | 3 | 10 | 0 | 2 | 2 |
| OTU203 | 0 | 0 | 0 | 0 | 0 | 0 | 6 | 27 | 13 | 21 | 7 | 42 | 2 | 8 | 6 | 0 | 1 | 1 |
| OTU204 | 0 | 0 | 0 | 0 | 0 | 1 | 0 | 0 | 0 | 0 | 1 | 1 | 24 | 13 | 1 | 0 | 202 | 11 |
| OTU205 | 26 | 23 | 29 | 62 | 52 | 99 | 2 | 0 | 1 | 0 | 1 | 0 | 0 | 0 | 0 | 0 | 0 | 0 |
| OTU206 | 133 | 1 | 7 | 1 | 3 | 9 | 1 | 0 | 0 | 0 | 0 | 0 | 0 | 0 | 1 | 0 | 2 | 0 |
| OTU207 | 33 | 14 | 61 | 0 | 0 | 77 | 12 | 1 | 2 | 0 | 0 | 4 | 0 | 1 | 1 | 0 | 1 | 2 |
| OTU208 | 8 | 17 | 15 | 31 | 7 | 72 | 0 | 0 | 0 | 0 | 0 | 0 | 0 | 0 | 0 | 0 | 0 | 1 |
| OTU209 | 1 | 1 | 3 | 0 | 0 | 233 | 0 | 0 | 1 | 1 | 1 | 1 | 0 | 0 | 0 | 0 | 0 | 0 |
| OTU21 | 178 | 206 | 163 | 161 | 247 | 231 | 192 | 377 | 279 | 335 | 225 | 254 | 580 | 596 | 340 | 749 | 219 | 385 |
| OTU210 | 2 | 0 | 2 | 0 | 0 | 1 | 59 | 8 | 33 | 80 | 32 | 33 | 2 | 8 | 30 | 0 | 3 | 3 |
| OTU211 | 1 | 0 | 0 | 0 | 0 | 0 | 0 | 0 | 1 | 0 | 0 | 1 | 17 | 7 | 0 | 2 | 121 | 53 |
| OTU212 | 41 | 89 | 0 | 0 | 2 | 0 | 9 | 1 | 0 | 0 | 0 | 0 | 1 | 0 | 1 | 0 | 0 | 0 |
| OTU213 | 75 | 19 | 59 | 51 | 2 | 209 | 6 | 6 | 10 | 6 | 2 | 4 | 0 | 1 | 3 | 0 | 2 | 1 |
| OTU214 | 1 | 0 | 0 | 0 | 0 | 0 | 10 | 122 | 1 | 5 | 3 | 21 | 1 | 0 | 2 | 0 | 0 | 1 |
| OTU215 | 1 | 4 | 6 | 1 | 21 | 170 | 1 | 21 | 8 | 8 | 6 | 15 | 5 | 11 | 4 | 0 | 2 | 1 |
| OTU216 | 7 | 7 | 13 | 1 | 14 | 8 | 1 | 154 | 13 | 58 | 108 | 25 | 9 | 2 | 21 | 0 | 1 | 2 |
| OTU217 | 0 | 0 | 2 | 1 | 0 | 0 | 1 | 1 | 0 | 0 | 1 | 0 | 15 | 17 | 0 | 3 | 63 | 34 |
| OTU218 | 0 | 0 | 0 | 0 | 0 | 0 | 0 | 0 | 0 | 0 | 1 | 0 | 0 | 0 | 0 | 0 | 151 | 0 |
| OTU219 | 12 | 12 | 19 | 7 | 26 | 29 | 16 | 7 | 8 | 11 | 15 | 13 | 1 | 4 | 6 | 0 | 4 | 3 |
| OTU22 | 2304 | 4 | 126 | 0 | 36 | 25 | 4 | 1 | 1 | 12 | 66 | 12 | 8 | 2 | 1 | 0 | 1 | 1 |
| OTU220 | 65 | 0 | 111 | 0 | 0 | 22 | 12 | 10 | 2 | 0 | 6 | 10 | 2 | 5 | 0 | 0 | 1 | 0 |
| OTU221 | 48 | 14 | 27 | 51 | 11 | 99 | 0 | 0 | 0 | 1 | 0 | 0 | 0 | 0 | 0 | 0 | 0 | 0 |
| OTU222 | 3 | 6 | 16 | 12 | 82 | 290 | 3 | 23 | 22 | 33 | 22 | 18 | 1 | 3 | 11 | 0 | 3 | 3 |
| OTU223 | 0 | 2 | 0 | 0 | 1 | 2 | 0 | 0 | 1 | 22 | 104 | 0 | 3 | 2 | 0 | 0 | 3 | 2 |
| OTU224 | 1 | 9 | 2 | 2 | 10 | 15 | 0 | 16 | 57 | 10 | 32 | 21 | 2 | 3 | 27 | 0 | 3 | 2 |
| OTU225 | 0 | 0 | 0 | 0 | 1 | 1 | 13 | 11 | 36 | 13 | 15 | 28 | 3 | 4 | 24 | 0 | 2 | 0 |
| OTU226 | 0 | 0 | 0 | 0 | 0 | 0 | 28 | 11 | 19 | 2 | 9 | 20 | 0 | 0 | 20 | 0 | 0 | 1 |
| OTU227 | 0 | 0 | 0 | 0 | 0 | 0 | 19 | 14 | 18 | 5 | 7 | 20 | 0 | 9 | 13 | 0 | 3 | 0 |
| OTU228 | 0 | 0 | 82 | 0 | 0 | 1 | 80 | 6 | 16 | 0 | 0 | 18 | 0 | 4 | 16 | 0 | 0 | 0 |
| OTU229 | 17 | 27 | 16 | 6 | 31 | 59 | 48 | 26 | 12 | 31 | 8 | 26 | 1 | 5 | 10 | 0 | 2 | 2 |
| OTU23 | 21 | 359 | 1290 | 620 | 202 | 679 | 72 | 284 | 60 | 41 | 47 | 111 | 9 | 16 | 88 | 0 | 5 | 8 |
| OTU230 | 0 | 0 | 1 | 0 | 0 | 0 | 92 | 16 | 15 | 63 | 44 | 24 | 3 | 5 | 25 | 0 | 1 | 0 |
| OTU231 | 12 | 48 | 23 | 3 | 1 | 30 | 6 | 26 | 9 | 34 | 10 | 18 | 1 | 3 | 9 | 0 | 2 | 1 |
| OTU232 | 8 | 0 | 15 | 2 | 3 | 83 | 86 | 42 | 14 | 5 | 0 | 28 | 3 | 20 | 13 | 0 | 2 | 1 |
| OTU233 | 16 | 8 | 6 | 11 | 49 | 22 | 0 | 0 | 0 | 1 | 0 | 0 | 0 | 0 | 0 | 0 | 0 | 0 |
| OTU234 | 6 | 44 | 27 | 19 | 10 | 34 | 3 | 88 | 45 | 44 | 50 | 35 | 2 | 1 | 30 | 0 | 7 | 1 |
| OTU235 | 1 | 314 | 18 | 329 | 48 | 10 | 0 | 12 | 9 | 5 | 6 | 5 | 0 | 0 | 3 | 0 | 1 | 1 |
| OTU236 | 3 | 18 | 8 | 45 | 43 | 71 | 0 | 1 | 1 | 0 | 1 | 0 | 0 | 0 | 0 | 0 | 0 | 0 |
| OTU237 | 0 | 1 | 0 | 0 | 0 | 1 | 18 | 15 | 35 | 6 | 5 | 19 | 1 | 2 | 26 | 0 | 2 | 0 |
| OTU238 | 1 | 9 | 10 | 1 | 23 | 18 | 0 | 120 | 56 | 7 | 36 | 11 | 0 | 0 | 64 | 0 | 11 | 7 |
| OTU239 | 4 | 10 | 15 | 13 | 64 | 13 | 6 | 49 | 12 | 44 | 69 | 32 | 2 | 3 | 4 | 0 | 0 | 0 |
| OTU24 | 139 | 62 | 34 | 44 | 1395 | 1369 | 140 | 406 | 145 | 250 | 677 | 242 | 14 | 14 | 115 | 1 | 15 | 8 |
| OTU240 | 12 | 2 | 31 | 6 | 37 | 33 | 0 | 0 | 0 | 0 | 0 | 0 | 0 | 0 | 1 | 0 | 1 | 0 |
| OTU241 | 23 | 38 | 21 | 25 | 114 | 26 | 0 | 0 | 0 | 1 | 0 | 0 | 0 | 0 | 1 | 0 | 0 | 0 |
| OTU242 | 23 | 14 | 17 | 6 | 8 | 3 | 3 | 2 | 2 | 1 | 0 | 5 | 3 | 0 | 3 | 1 | 0 | 0 |
| OTU243 | 35 | 2 | 3 | 13 | 1 | 68 | 0 | 0 | 0 | 0 | 0 | 0 | 0 | 0 | 1 | 0 | 0 | 0 |
| OTU244 | 2 | 3 | 3 | 8 | 6 | 9 | 1 | 18 | 16 | 11 | 14 | 8 | 0 | 0 | 10 | 0 | 1 | 0 |
| OTU245 | 0 | 0 | 0 | 0 | 0 | 0 | 46 | 4 | 17 | 14 | 0 | 62 | 13 | 27 | 10 | 0 | 2 | 0 |
| OTU246 | 0 | 0 | 0 | 0 | 0 | 0 | 3 | 1 | 44 | 0 | 0 | 12 | 0 | 1 | 39 | 0 | 2 | 0 |
| OTU247 | 0 | 0 | 0 | 0 | 0 | 0 | 7 | 5 | 23 | 8 | 12 | 11 | 6 | 7 | 17 | 0 | 2 | 1 |
| OTU248 | 0 | 0 | 0 | 0 | 1 | 0 | 8 | 1 | 0 | 2 | 0 | 0 | 2 | 14 | 0 | 3 | 30 | 21 |
| OTU249 | 1 | 1 | 1 | 3 | 1 | 0 | 147 | 24 | 25 | 53 | 70 | 50 | 6 | 13 | 50 | 0 | 2 | 2 |
| OTU25 | 38 | 8 | 6 | 27 | 48 | 17 | 115 | 151 | 301 | 342 | 441 | 392 | 48 | 170 | 378 | 0 | 42 | 31 |
| OTU250 | 0 | 1 | 0 | 0 | 0 | 0 | 2 | 7 | 22 | 46 | 43 | 34 | 0 | 1 | 18 | 0 | 0 | 1 |
| OTU251 | 1 | 0 | 0 | 1 | 0 | 3 | 0 | 0 | 0 | 0 | 0 | 0 | 38 | 5 | 0 | 1 | 88 | 10 |
| OTU252 | 4 | 2 | 26 | 17 | 40 | 57 | 2 | 0 | 0 | 1 | 1 | 0 | 0 | 0 | 0 | 0 | 1 | 0 |
| OTU253 | 0 | 0 | 29 | 1 | 3 | 153 | 1 | 84 | 6 | 28 | 15 | 12 | 0 | 2 | 6 | 0 | 0 | 0 |
| OTU254 | 31 | 5 | 5 | 9 | 0 | 18 | 106 | 68 | 75 | 14 | 30 | 88 | 9 | 22 | 56 | 0 | 1 | 2 |
| OTU255 | 0 | 0 | 1 | 0 | 0 | 0 | 23 | 5 | 16 | 5 | 6 | 38 | 10 | 19 | 16 | 0 | 0 | 0 |
| OTU256 | 5 | 0 | 6 | 2 | 5 | 2 | 0 | 57 | 60 | 22 | 9 | 21 | 0 | 0 | 45 | 0 | 5 | 2 |
| OTU257 | 12 | 36 | 20 | 86 | 91 | 85 | 12 | 76 | 47 | 201 | 111 | 58 | 1 | 4 | 18 | 0 | 4 | 0 |
| OTU258 | 2 | 4 | 3 | 16 | 34 | 11 | 12 | 36 | 20 | 105 | 41 | 25 | 2 | 0 | 20 | 0 | 1 | 0 |
| OTU259 | 0 | 0 | 0 | 0 | 0 | 0 | 0 | 0 | 0 | 0 | 0 | 0 | 6 | 0 | 0 | 1 | 35 | 63 |
| OTU26 | 2 | 4 | 10 | 3 | 6 | 8 | 723 | 108 | 211 | 142 | 179 | 966 | 50 | 173 | 237 | 0 | 7 | 13 |
| OTU260 | 37 | 28 | 39 | 3 | 3 | 44 | 10 | 26 | 3 | 5 | 7 | 9 | 0 | 1 | 8 | 0 | 0 | 0 |
| OTU261 | 38 | 19 | 18 | 28 | 25 | 43 | 8 | 2 | 11 | 16 | 30 | 6 | 0 | 3 | 11 | 0 | 2 | 0 |
| OTU262 | 0 | 172 | 2 | 0 | 0 | 0 | 0 | 7 | 6 | 1 | 3 | 4 | 1 | 1 | 8 | 0 | 0 | 0 |
| OTU263 | 4 | 10 | 4 | 2 | 7 | 3 | 10 | 7 | 5 | 12 | 6 | 9 | 21 | 15 | 8 | 22 | 4 | 19 |
| OTU264 | 7 | 20 | 25 | 19 | 26 | 26 | 39 | 29 | 29 | 46 | 67 | 36 | 5 | 10 | 22 | 0 | 0 | 3 |
| OTU265 | 1 | 1 | 2 | 0 | 1 | 1 | 17 | 12 | 18 | 11 | 14 | 22 | 2 | 3 | 7 | 0 | 0 | 1 |
| OTU266 | 27 | 11 | 22 | 73 | 25 | 106 | 2 | 1 | 3 | 5 | 5 | 0 | 0 | 0 | 4 | 0 | 1 | 2 |
| OTU267 | 1 | 5 | 3 | 2 | 0 | 25 | 2 | 16 | 10 | 12 | 17 | 11 | 0 | 3 | 3 | 0 | 3 | 2 |
| OTU268 | 1 | 1 | 6 | 0 | 8 | 8 | 0 | 44 | 11 | 0 | 3 | 15 | 0 | 1 | 13 | 0 | 3 | 0 |
| OTU269 | 1 | 0 | 1 | 1 | 1 | 0 | 35 | 11 | 18 | 15 | 18 | 3 | 3 | 9 | 10 | 0 | 0 | 0 |
| OTU27 | 7 | 6 | 4 | 7 | 12 | 6 | 710 | 139 | 227 | 378 | 1995 | 850 | 41 | 79 | 39 | 0 | 4 | 5 |
| OTU270 | 0 | 0 | 2 | 0 | 0 | 0 | 58 | 4 | 6 | 13 | 20 | 20 | 2 | 5 | 12 | 0 | 0 | 0 |
| OTU271 | 8 | 19 | 5 | 1 | 8 | 13 | 13 | 21 | 18 | 10 | 11 | 18 | 2 | 7 | 21 | 0 | 1 | 2 |
| OTU272 | 0 | 0 | 0 | 0 | 0 | 0 | 47 | 7 | 22 | 22 | 10 | 50 | 2 | 7 | 22 | 0 | 0 | 0 |
| OTU273 | 0 | 0 | 0 | 0 | 0 | 0 | 0 | 0 | 0 | 0 | 0 | 0 | 5 | 0 | 0 | 0 | 101 | 5 |
| OTU274 | 0 | 0 | 0 | 0 | 0 | 0 | 1 | 0 | 0 | 0 | 0 | 0 | 1 | 0 | 0 | 0 | 178 | 0 |
| OTU275 | 1 | 0 | 0 | 0 | 0 | 0 | 0 | 0 | 0 | 0 | 0 | 0 | 1 | 1 | 0 | 1 | 346 | 0 |
| OTU276 | 75 | 1 | 4 | 3 | 1 | 22 | 0 | 1 | 0 | 0 | 1 | 41 | 0 | 1 | 0 | 0 | 0 | 0 |
| OTU277 | 22 | 12 | 53 | 15 | 2 | 14 | 4 | 2 | 6 | 2 | 0 | 3 | 0 | 3 | 4 | 0 | 0 | 1 |
| OTU278 | 5 | 16 | 35 | 0 | 5 | 33 | 0 | 0 | 0 | 0 | 0 | 0 | 0 | 0 | 0 | 0 | 0 | 0 |
| OTU279 | 14 | 4 | 19 | 8 | 12 | 2 | 9 | 6 | 0 | 26 | 16 | 17 | 0 | 0 | 0 | 0 | 1 | 0 |
| OTU28 | 40 | 131 | 204 | 10 | 883 | 654 | 2 | 0 | 1 | 1 | 1 | 1 | 3 | 1 | 1 | 3 | 0 | 2 |
| OTU280 | 0 | 2 | 2 | 1 | 5 | 2 | 4 | 4 | 5 | 3 | 5 | 6 | 0 | 3 | 3 | 5 | 2 | 2 |
| OTU281 | 3 | 81 | 0 | 0 | 0 | 1 | 11 | 31 | 4 | 0 | 2 | 3 | 0 | 0 | 4 | 0 | 0 | 0 |
| OTU282 | 2 | 12 | 4 | 1 | 105 | 60 | 2 | 16 | 21 | 37 | 30 | 12 | 0 | 1 | 12 | 0 | 2 | 2 |
| OTU283 | 1 | 0 | 5 | 14 | 32 | 28 | 1 | 6 | 8 | 8 | 6 | 5 | 0 | 0 | 12 | 0 | 1 | 0 |
| OTU284 | 0 | 0 | 0 | 0 | 1 | 0 | 6 | 9 | 11 | 2 | 3 | 30 | 2 | 2 | 18 | 0 | 0 | 0 |
| OTU285 | 0 | 0 | 1 | 1 | 0 | 0 | 11 | 25 | 16 | 17 | 5 | 45 | 0 | 9 | 20 | 0 | 3 | 0 |
| OTU286 | 0 | 0 | 0 | 0 | 0 | 0 | 2 | 13 | 12 | 15 | 5 | 7 | 4 | 15 | 8 | 0 | 1 | 0 |
| OTU287 | 0 | 3 | 0 | 3 | 14 | 0 | 2 | 39 | 11 | 2 | 21 | 67 | 1 | 11 | 10 | 0 | 0 | 0 |
| OTU288 | 0 | 0 | 0 | 0 | 0 | 0 | 0 | 0 | 0 | 0 | 0 | 0 | 8 | 26 | 0 | 1 | 65 | 30 |
| OTU289 | 0 | 1 | 0 | 0 | 0 | 0 | 0 | 0 | 0 | 0 | 0 | 0 | 11 | 5 | 0 | 0 | 64 | 17 |
| OTU29 | 171 | 22 | 81 | 38 | 37 | 78 | 720 | 1100 | 1508 | 625 | 1043 | 1290 | 155 | 543 | 1300 | 9 | 67 | 72 |
| OTU290 | 35 | 43 | 12 | 0 | 2 | 19 | 5 | 5 | 1 | 5 | 4 | 16 | 0 | 4 | 0 | 0 | 2 | 0 |
| OTU291 | 10 | 13 | 16 | 2 | 30 | 1 | 1 | 0 | 1 | 0 | 0 | 0 | 0 | 0 | 0 | 0 | 0 | 0 |
| OTU292 | 41 | 0 | 15 | 13 | 11 | 26 | 0 | 0 | 0 | 0 | 0 | 0 | 0 | 0 | 0 | 0 | 0 | 1 |
| OTU293 | 53 | 33 | 70 | 15 | 27 | 20 | 0 | 1 | 0 | 0 | 2 | 0 | 0 | 0 | 0 | 0 | 0 | 0 |
| OTU294 | 19 | 0 | 11 | 21 | 5 | 18 | 2 | 0 | 0 | 0 | 0 | 0 | 0 | 0 | 0 | 0 | 0 | 0 |
| OTU295 | 0 | 28 | 2 | 0 | 0 | 22 | 11 | 3 | 10 | 0 | 0 | 22 | 1 | 2 | 6 | 0 | 2 | 1 |
| OTU296 | 0 | 21 | 5 | 7 | 1 | 95 | 1 | 36 | 11 | 14 | 6 | 8 | 0 | 1 | 7 | 0 | 1 | 2 |
| OTU297 | 0 | 0 | 0 | 2 | 0 | 0 | 14 | 2 | 5 | 42 | 7 | 11 | 3 | 3 | 6 | 0 | 0 | 2 |
| OTU298 | 8 | 9 | 15 | 10 | 19 | 9 | 10 | 21 | 12 | 13 | 11 | 18 | 30 | 24 | 15 | 38 | 16 | 26 |
| OTU299 | 0 | 0 | 0 | 1 | 56 | 16 | 3 | 12 | 5 | 13 | 5 | 0 | 1 | 1 | 4 | 0 | 1 | 0 |
| OTU3 | 2388 | 130 | 660 | 1393 | 693 | 779 | 1117 | 2742 | 2408 | 1755 | 731 | 979 | 11350 | 7912 | 3215 | 9927 | 4024 | 11377 |
| OTU30 | 14 | 7 | 22 | 14 | 13 | 18 | 959 | 279 | 387 | 349 | 682 | 344 | 27 | 93 | 564 | 0 | 2 | 5 |
| OTU300 | 1 | 3 | 1 | 0 | 0 | 1 | 62 | 12 | 19 | 51 | 104 | 12 | 2 | 5 | 40 | 0 | 0 | 3 |
| OTU301 | 1 | 0 | 0 | 0 | 1 | 7 | 11 | 26 | 3 | 3 | 35 | 5 | 0 | 3 | 2 | 0 | 0 | 0 |
| OTU302 | 0 | 0 | 4 | 0 | 8 | 0 | 2 | 8 | 5 | 11 | 32 | 2 | 0 | 1 | 2 | 0 | 1 | 1 |
| OTU303 | 0 | 18 | 2 | 0 | 2 | 85 | 6 | 21 | 67 | 9 | 59 | 66 | 0 | 1 | 71 | 0 | 2 | 3 |
| OTU304 | 0 | 2 | 5 | 0 | 1 | 2 | 10 | 105 | 61 | 18 | 7 | 30 | 2 | 4 | 42 | 0 | 3 | 5 |
| OTU305 | 0 | 0 | 0 | 0 | 0 | 0 | 2 | 39 | 35 | 0 | 2 | 75 | 6 | 10 | 32 | 0 | 1 | 0 |
| OTU306 | 0 | 0 | 0 | 0 | 0 | 0 | 0 | 0 | 0 | 0 | 0 | 0 | 6 | 10 | 0 | 4 | 27 | 151 |
| OTU307 | 0 | 0 | 0 | 0 | 0 | 0 | 0 | 0 | 0 | 0 | 0 | 0 | 8 | 40 | 0 | 1 | 56 | 35 |
| OTU308 | 0 | 1 | 0 | 0 | 0 | 0 | 0 | 0 | 0 | 0 | 0 | 0 | 0 | 0 | 0 | 0 | 94 | 0 |
| OTU309 | 1 | 8 | 11 | 0 | 0 | 28 | 1 | 30 | 4 | 15 | 25 | 6 | 3 | 4 | 4 | 0 | 1 | 0 |
| OTU31 | 72 | 178 | 95 | 664 | 499 | 1012 | 79 | 22 | 15 | 74 | 32 | 11 | 4 | 11 | 10 | 0 | 7 | 5 |
| OTU310 | 9 | 1 | 4 | 0 | 0 | 0 | 24 | 27 | 21 | 4 | 6 | 18 | 1 | 10 | 23 | 0 | 2 | 1 |
| OTU311 | 8 | 0 | 1 | 0 | 0 | 0 | 3 | 1 | 0 | 45 | 0 | 0 | 0 | 0 | 0 | 1 | 0 | 0 |
| OTU312 | 8 | 4 | 7 | 2 | 6 | 3 | 2 | 8 | 11 | 5 | 3 | 5 | 1 | 3 | 11 | 0 | 1 | 0 |
| OTU313 | 15 | 4 | 6 | 1 | 8 | 5 | 0 | 1 | 0 | 0 | 0 | 0 | 0 | 0 | 0 | 0 | 0 | 0 |
| OTU314 | 2 | 18 | 28 | 13 | 21 | 101 | 7 | 101 | 60 | 56 | 62 | 36 | 4 | 24 | 25 | 0 | 8 | 9 |
| OTU315 | 0 | 1 | 1 | 0 | 0 | 0 | 27 | 10 | 12 | 12 | 24 | 33 | 2 | 4 | 20 | 0 | 0 | 0 |
| OTU316 | 8 | 10 | 7 | 1 | 0 | 2 | 3 | 13 | 9 | 11 | 7 | 8 | 1 | 8 | 14 | 0 | 2 | 0 |
| OTU317 | 114 | 26 | 34 | 0 | 128 | 14 | 38 | 11 | 14 | 153 | 24 | 51 | 7 | 24 | 18 | 0 | 7 | 8 |
| OTU318 | 15 | 1 | 10 | 36 | 10 | 41 | 0 | 0 | 0 | 0 | 0 | 0 | 0 | 0 | 0 | 0 | 0 | 0 |
| OTU319 | 1 | 0 | 0 | 0 | 0 | 1 | 0 | 71 | 42 | 1 | 2 | 29 | 0 | 1 | 25 | 0 | 3 | 7 |
| OTU32 | 244 | 156 | 271 | 339 | 240 | 641 | 3 | 3 | 0 | 1 | 1 | 1 | 1 | 0 | 1 | 1 | 0 | 1 |
| OTU320 | 7 | 6 | 12 | 5 | 5 | 14 | 6 | 11 | 12 | 11 | 11 | 10 | 2 | 8 | 4 | 2 | 23 | 7 |
| OTU321 | 4 | 12 | 18 | 3 | 10 | 11 | 5 | 11 | 10 | 11 | 12 | 13 | 6 | 15 | 12 | 4 | 20 | 4 |
| OTU322 | 9 | 148 | 23 | 1 | 19 | 0 | 0 | 0 | 0 | 0 | 0 | 0 | 0 | 0 | 0 | 0 | 0 | 0 |
| OTU323 | 7 | 12 | 18 | 2 | 1 | 2 | 7 | 92 | 19 | 37 | 20 | 24 | 0 | 3 | 29 | 0 | 2 | 1 |
| OTU324 | 0 | 91 | 4 | 0 | 1 | 4 | 0 | 0 | 0 | 1 | 0 | 0 | 1 | 0 | 0 | 0 | 0 | 0 |
| OTU325 | 1 | 34 | 8 | 11 | 40 | 3 | 0 | 0 | 0 | 0 | 1 | 0 | 0 | 0 | 1 | 0 | 0 | 0 |
| OTU326 | 38 | 15 | 11 | 0 | 0 | 2 | 130 | 35 | 10 | 1 | 0 | 5 | 0 | 3 | 7 | 1 | 1 | 1 |
| OTU327 | 0 | 0 | 1 | 0 | 1 | 5 | 0 | 0 | 0 | 3 | 0 | 0 | 8 | 3 | 0 | 1 | 50 | 4 |
| OTU328 | 0 | 0 | 0 | 0 | 0 | 0 | 7 | 0 | 3 | 0 | 9 | 9 | 0 | 3 | 0 | 0 | 0 | 0 |
| OTU329 | 6 | 6 | 4 | 5 | 3 | 2 | 6 | 5 | 9 | 10 | 3 | 9 | 19 | 13 | 6 | 18 | 12 | 8 |
| OTU33 | 4 | 4 | 276 | 1 | 2 | 175 | 0 | 68 | 7 | 441 | 1063 | 12 | 3 | 7 | 7 | 0 | 3 | 0 |
| OTU330 | 1 | 1 | 0 | 0 | 0 | 0 | 0 | 2 | 0 | 38 | 17 | 0 | 0 | 0 | 0 | 0 | 2 | 0 |
| OTU331 | 0 | 1 | 0 | 0 | 0 | 0 | 0 | 0 | 0 | 0 | 0 | 0 | 0 | 0 | 1 | 0 | 58 | 0 |
| OTU332 | 0 | 0 | 0 | 0 | 0 | 0 | 0 | 0 | 0 | 0 | 0 | 0 | 3 | 13 | 0 | 0 | 108 | 28 |
| OTU333 | 6 | 9 | 8 | 10 | 28 | 1 | 1 | 0 | 0 | 1 | 0 | 0 | 0 | 1 | 0 | 0 | 0 | 0 |
| OTU334 | 1 | 0 | 2 | 5 | 2 | 1 | 8 | 1 | 7 | 17 | 2 | 11 | 2 | 2 | 4 | 0 | 0 | 2 |
| OTU335 | 4 | 3 | 11 | 0 | 3 | 9 | 0 | 4 | 3 | 3 | 7 | 5 | 0 | 0 | 0 | 0 | 0 | 0 |
| OTU336 | 33 | 11 | 26 | 6 | 2 | 24 | 6 | 0 | 1 | 1 | 4 | 0 | 0 | 0 | 2 | 0 | 0 | 1 |
| OTU337 | 23 | 28 | 21 | 11 | 22 | 30 | 10 | 3 | 2 | 7 | 6 | 3 | 0 | 0 | 6 | 0 | 0 | 1 |
| OTU338 | 0 | 6 | 0 | 4 | 29 | 2 | 0 | 0 | 0 | 0 | 1 | 0 | 0 | 0 | 0 | 0 | 0 | 0 |
| OTU339 | 3 | 3 | 5 | 2 | 5 | 5 | 2 | 0 | 4 | 2 | 3 | 2 | 1 | 1 | 1 | 1 | 0 | 0 |
| OTU34 | 226 | 34 | 258 | 109 | 885 | 30 | 6 | 0 | 3 | 4 | 5 | 0 | 0 | 1 | 0 | 0 | 0 | 3 |
| OTU340 | 2 | 1 | 12 | 2 | 0 | 4 | 0 | 2 | 14 | 4 | 0 | 10 | 1 | 6 | 10 | 0 | 0 | 0 |
| OTU341 | 0 | 12 | 0 | 18 | 0 | 35 | 0 | 0 | 1 | 0 | 1 | 0 | 0 | 0 | 0 | 0 | 0 | 0 |
| OTU342 | 1 | 2 | 3 | 3 | 3 | 59 | 1 | 9 | 5 | 26 | 4 | 9 | 0 | 0 | 2 | 0 | 1 | 0 |
| OTU343 | 5 | 8 | 10 | 0 | 9 | 3 | 0 | 7 | 0 | 3 | 0 | 2 | 2 | 2 | 1 | 0 | 0 | 0 |
| OTU344 | 6 | 5 | 8 | 3 | 7 | 17 | 5 | 5 | 4 | 4 | 8 | 10 | 1 | 5 | 14 | 0 | 2 | 0 |
| OTU345 | 15 | 4 | 5 | 25 | 7 | 15 | 0 | 0 | 0 | 0 | 0 | 0 | 0 | 0 | 0 | 0 | 0 | 0 |
| OTU346 | 0 | 0 | 0 | 1 | 1 | 0 | 0 | 0 | 0 | 0 | 1 | 0 | 4 | 4 | 0 | 1 | 33 | 10 |
| OTU347 | 4 | 4 | 7 | 1 | 8 | 23 | 33 | 23 | 8 | 32 | 33 | 15 | 2 | 8 | 7 | 0 | 3 | 1 |
| OTU348 | 0 | 2 | 5 | 0 | 1 | 80 | 0 | 11 | 3 | 19 | 3 | 6 | 1 | 0 | 3 | 0 | 2 | 1 |
| OTU349 | 0 | 0 | 2 | 0 | 0 | 2 | 8 | 16 | 2 | 25 | 2 | 10 | 2 | 9 | 14 | 0 | 1 | 0 |
| OTU35 | 286 | 339 | 403 | 427 | 496 | 226 | 6 | 2 | 4 | 8 | 5 | 7 | 2 | 0 | 1 | 0 | 2 | 3 |
| OTU350 | 0 | 0 | 5 | 1 | 3 | 0 | 13 | 11 | 3 | 4 | 3 | 13 | 2 | 4 | 4 | 0 | 1 | 0 |
| OTU351 | 0 | 1 | 0 | 1 | 0 | 0 | 14 | 0 | 5 | 17 | 8 | 9 | 0 | 4 | 5 | 0 | 0 | 0 |
| OTU352 | 0 | 0 | 0 | 0 | 0 | 0 | 15 | 0 | 12 | 8 | 4 | 21 | 1 | 2 | 16 | 0 | 0 | 0 |
| OTU353 | 2 | 15 | 1 | 4 | 0 | 0 | 6 | 8 | 1 | 18 | 13 | 2 | 1 | 1 | 2 | 0 | 1 | 0 |
| OTU354 | 0 | 0 | 0 | 0 | 0 | 0 | 9 | 6 | 2 | 24 | 2 | 5 | 0 | 0 | 6 | 0 | 2 | 0 |
| OTU355 | 4 | 0 | 16 | 0 | 0 | 0 | 2 | 13 | 1 | 23 | 39 | 0 | 0 | 0 | 5 | 0 | 0 | 0 |
| OTU356 | 0 | 0 | 0 | 0 | 0 | 0 | 0 | 0 | 0 | 4 | 1 | 3 | 5 | 7 | 0 | 6 | 60 | 9 |
| OTU357 | 0 | 0 | 0 | 0 | 0 | 0 | 0 | 3 | 13 | 1 | 8 | 21 | 0 | 3 | 8 | 0 | 2 | 0 |
| OTU358 | 0 | 0 | 1 | 0 | 0 | 0 | 0 | 0 | 0 | 0 | 0 | 0 | 2 | 19 | 0 | 2 | 46 | 66 |
| OTU359 | 0 | 0 | 0 | 0 | 0 | 0 | 0 | 1 | 0 | 0 | 0 | 0 | 11 | 1 | 0 | 8 | 134 | 204 |
| OTU36 | 0 | 3 | 8 | 1 | 32 | 58 | 46 | 506 | 16 | 308 | 358 | 369 | 30 | 6 | 270 | 1 | 26 | 19 |
| OTU360 | 3 | 0 | 2 | 14 | 0 | 41 | 0 | 5 | 6 | 3 | 1 | 3 | 0 | 0 | 8 | 0 | 1 | 0 |
| OTU361 | 5 | 2 | 2 | 6 | 13 | 12 | 2 | 7 | 9 | 8 | 10 | 6 | 0 | 1 | 4 | 0 | 0 | 0 |
| OTU362 | 18 | 6 | 7 | 9 | 13 | 14 | 1 | 5 | 8 | 5 | 1 | 3 | 1 | 2 | 12 | 0 | 0 | 0 |
| OTU363 | 2 | 6 | 8 | 2 | 11 | 57 | 0 | 2 | 2 | 15 | 4 | 1 | 0 | 0 | 2 | 0 | 0 | 1 |
| OTU364 | 60 | 22 | 12 | 5 | 17 | 20 | 1 | 0 | 1 | 10 | 8 | 4 | 2 | 1 | 0 | 0 | 0 | 0 |
| OTU365 | 5 | 19 | 5 | 3 | 21 | 2 | 0 | 0 | 0 | 0 | 0 | 0 | 0 | 0 | 0 | 0 | 0 | 0 |
| OTU366 | 3 | 1 | 0 | 7 | 5 | 1 | 0 | 2 | 2 | 3 | 1 | 7 | 1 | 8 | 2 | 0 | 2 | 2 |
| OTU367 | 7 | 0 | 1 | 1 | 3 | 5 | 1 | 8 | 2 | 0 | 2 | 2 | 0 | 0 | 0 | 0 | 0 | 0 |
| OTU368 | 52 | 9 | 1 | 3 | 6 | 2 | 37 | 0 | 6 | 0 | 2 | 25 | 4 | 8 | 7 | 0 | 0 | 0 |
| OTU369 | 58 | 1 | 8 | 0 | 0 | 1 | 0 | 0 | 0 | 0 | 0 | 0 | 1 | 0 | 0 | 0 | 0 | 0 |
| OTU37 | 8 | 6 | 9 | 12 | 7 | 8 | 991 | 313 | 637 | 286 | 608 | 76 | 12 | 15 | 754 | 0 | 11 | 12 |
| OTU370 | 16 | 0 | 2 | 0 | 15 | 0 | 0 | 0 | 0 | 0 | 0 | 0 | 0 | 0 | 0 | 0 | 0 | 0 |
| OTU371 | 2 | 31 | 0 | 1 | 13 | 12 | 0 | 0 | 1 | 1 | 0 | 2 | 0 | 0 | 0 | 0 | 0 | 0 |
| OTU372 | 0 | 8 | 3 | 0 | 0 | 1 | 5 | 10 | 8 | 4 | 0 | 10 | 0 | 1 | 9 | 1 | 2 | 0 |
| OTU373 | 3 | 16 | 3 | 18 | 15 | 1 | 1 | 1 | 0 | 5 | 20 | 4 | 0 | 0 | 1 | 0 | 1 | 0 |
| OTU374 | 4 | 7 | 10 | 2 | 3 | 13 | 5 | 4 | 7 | 7 | 16 | 12 | 2 | 4 | 7 | 5 | 6 | 1 |
| OTU375 | 0 | 87 | 0 | 0 | 1 | 1 | 0 | 0 | 0 | 0 | 0 | 0 | 0 | 0 | 0 | 0 | 0 | 0 |
| OTU376 | 5 | 16 | 48 | 0 | 1 | 3 | 0 | 0 | 0 | 0 | 0 | 0 | 0 | 0 | 0 | 0 | 0 | 0 |
| OTU377 | 0 | 2 | 0 | 0 | 31 | 1 | 2 | 16 | 8 | 9 | 11 | 2 | 1 | 0 | 7 | 0 | 2 | 1 |
| OTU378 | 0 | 1 | 4 | 2 | 68 | 22 | 0 | 0 | 0 | 0 | 0 | 6 | 0 | 0 | 2 | 0 | 0 | 0 |
| OTU379 | 4 | 1 | 8 | 1 | 15 | 4 | 9 | 2 | 29 | 13 | 18 | 38 | 2 | 7 | 55 | 0 | 4 | 0 |
| OTU38 | 3 | 95 | 6 | 3 | 4 | 160 | 1 | 273 | 324 | 1008 | 45 | 70 | 4 | 11 | 198 | 0 | 13 | 13 |
| OTU380 | 0 | 1 | 3 | 4 | 6 | 4 | 1 | 93 | 27 | 30 | 6 | 23 | 0 | 1 | 9 | 0 | 3 | 1 |
| OTU381 | 0 | 2 | 1 | 67 | 0 | 1 | 0 | 0 | 0 | 0 | 0 | 1 | 0 | 0 | 0 | 0 | 1 | 0 |
| OTU382 | 2 | 2 | 2 | 0 | 2 | 6 | 4 | 11 | 19 | 6 | 9 | 16 | 11 | 13 | 9 | 0 | 2 | 3 |
| OTU383 | 1 | 4 | 0 | 0 | 2 | 0 | 5 | 1 | 1 | 1 | 10 | 2 | 1 | 2 | 0 | 0 | 0 | 1 |
| OTU384 | 32 | 1 | 2 | 0 | 1 | 0 | 19 | 2 | 4 | 1 | 1 | 9 | 0 | 12 | 5 | 0 | 0 | 0 |
| OTU385 | 0 | 0 | 0 | 0 | 0 | 0 | 2 | 10 | 2 | 31 | 13 | 7 | 0 | 0 | 5 | 0 | 0 | 0 |
| OTU386 | 0 | 0 | 1 | 0 | 0 | 0 | 2 | 30 | 11 | 2 | 4 | 11 | 0 | 3 | 0 | 0 | 0 | 0 |
| OTU387 | 1 | 1 | 0 | 7 | 9 | 6 | 2 | 6 | 13 | 18 | 8 | 32 | 0 | 1 | 1 | 0 | 0 | 0 |
| OTU388 | 0 | 0 | 0 | 0 | 0 | 0 | 3 | 1 | 2 | 2 | 1 | 0 | 4 | 21 | 1 | 0 | 22 | 62 |
| OTU389 | 0 | 8 | 3 | 4 | 3 | 7 | 23 | 13 | 14 | 22 | 18 | 55 | 7 | 22 | 17 | 0 | 3 | 1 |
| OTU39 | 115 | 924 | 270 | 0 | 0 | 9 | 19 | 80 | 2 | 1 | 3 | 4 | 2 | 0 | 1 | 1 | 0 | 0 |
| OTU390 | 0 | 0 | 0 | 0 | 0 | 0 | 0 | 0 | 0 | 0 | 0 | 0 | 2 | 5 | 0 | 0 | 35 | 6 |
| OTU391 | 0 | 1 | 0 | 0 | 0 | 0 | 0 | 0 | 0 | 0 | 1 | 0 | 2 | 1 | 0 | 1 | 42 | 16 |
| OTU392 | 9 | 7 | 6 | 2 | 0 | 1 | 2 | 2 | 2 | 0 | 0 | 1 | 0 | 3 | 2 | 0 | 0 | 1 |
| OTU393 | 5 | 14 | 2 | 3 | 7 | 1 | 0 | 0 | 0 | 1 | 0 | 0 | 0 | 0 | 0 | 0 | 0 | 0 |
| OTU394 | 11 | 11 | 17 | 1 | 48 | 2 | 0 | 47 | 54 | 11 | 15 | 28 | 2 | 3 | 29 | 0 | 3 | 1 |
| OTU395 | 13 | 10 | 10 | 12 | 9 | 0 | 1 | 0 | 0 | 0 | 0 | 0 | 0 | 0 | 0 | 0 | 0 | 0 |
| OTU396 | 10 | 4 | 1 | 5 | 6 | 11 | 3 | 4 | 5 | 2 | 7 | 3 | 0 | 0 | 12 | 0 | 0 | 1 |
| OTU397 | 20 | 9 | 25 | 18 | 3 | 22 | 1 | 0 | 0 | 1 | 0 | 0 | 0 | 0 | 0 | 0 | 0 | 0 |
| OTU398 | 3 | 3 | 2 | 1 | 0 | 2 | 1 | 1 | 2 | 5 | 4 | 3 | 1 | 1 | 2 | 1 | 3 | 0 |
| OTU399 | 6 | 1 | 4 | 1 | 12 | 2 | 0 | 13 | 10 | 11 | 4 | 1 | 0 | 0 | 5 | 0 | 0 | 0 |
| OTU4 | 28 | 16 | 39 | 15 | 34 | 24 | 3 | 31 | 14 | 20 | 16 | 21 | 2503 | 1992 | 20 | 273 | 12142 | 7471 |
| OTU40 | 0 | 2 | 2 | 0 | 4 | 2 | 2 | 750 | 338 | 582 | 1203 | 765 | 6 | 6 | 66 | 1 | 8 | 2 |
| OTU400 | 5 | 2 | 2 | 8 | 14 | 8 | 1 | 0 | 1 | 11 | 2 | 0 | 0 | 0 | 5 | 0 | 0 | 0 |
| OTU401 | 10 | 8 | 9 | 1 | 9 | 12 | 0 | 0 | 0 | 0 | 0 | 0 | 1 | 0 | 0 | 0 | 0 | 0 |
| OTU402 | 9 | 9 | 1 | 0 | 21 | 28 | 0 | 1 | 0 | 2 | 5 | 0 | 1 | 0 | 1 | 0 | 0 | 0 |
| OTU403 | 10 | 31 | 12 | 3 | 7 | 12 | 2 | 1 | 1 | 0 | 0 | 0 | 1 | 0 | 0 | 0 | 0 | 0 |
| OTU404 | 4 | 14 | 2 | 18 | 70 | 47 | 0 | 22 | 15 | 8 | 9 | 13 | 1 | 5 | 6 | 0 | 1 | 1 |
| OTU405 | 14 | 16 | 10 | 19 | 8 | 3 | 6 | 0 | 0 | 0 | 0 | 1 | 0 | 0 | 0 | 0 | 0 | 0 |
| OTU406 | 0 | 1 | 1 | 8 | 7 | 73 | 0 | 1 | 0 | 1 | 0 | 0 | 0 | 0 | 1 | 0 | 2 | 0 |
| OTU407 | 0 | 1 | 4 | 0 | 14 | 17 | 0 | 0 | 0 | 0 | 0 | 0 | 0 | 0 | 0 | 0 | 0 | 0 |
| OTU408 | 1 | 2 | 3 | 13 | 7 | 5 | 1 | 5 | 17 | 17 | 7 | 2 | 0 | 0 | 20 | 0 | 0 | 0 |
| OTU409 | 4 | 1 | 8 | 14 | 0 | 4 | 2 | 1 | 1 | 0 | 3 | 2 | 0 | 0 | 2 | 0 | 0 | 0 |
| OTU41 | 14 | 17 | 27 | 20 | 55 | 85 | 6 | 120 | 64 | 1089 | 1067 | 165 | 9 | 22 | 46 | 1 | 13 | 3 |
| OTU410 | 13 | 12 | 11 | 1 | 50 | 69 | 5 | 5 | 7 | 0 | 1 | 6 | 0 | 2 | 4 | 0 | 0 | 0 |
| OTU411 | 1 | 4 | 4 | 2 | 3 | 12 | 2 | 4 | 6 | 4 | 7 | 2 | 0 | 1 | 1 | 0 | 0 | 0 |
| OTU412 | 9 | 2 | 9 | 23 | 17 | 28 | 0 | 0 | 0 | 0 | 0 | 1 | 0 | 0 | 0 | 0 | 1 | 0 |
| OTU413 | 0 | 9 | 1 | 3 | 19 | 10 | 0 | 7 | 6 | 6 | 1 | 7 | 0 | 6 | 0 | 0 | 0 | 0 |
| OTU414 | 0 | 0 | 14 | 0 | 42 | 0 | 54 | 2 | 34 | 0 | 0 | 57 | 0 | 11 | 31 | 0 | 4 | 0 |
| OTU415 | 0 | 0 | 0 | 0 | 0 | 0 | 5 | 4 | 2 | 10 | 5 | 3 | 0 | 0 | 0 | 0 | 0 | 0 |
| OTU416 | 0 | 0 | 0 | 1 | 0 | 1 | 30 | 2 | 2 | 12 | 2 | 6 | 2 | 4 | 0 | 0 | 1 | 0 |
| OTU417 | 1 | 4 | 12 | 11 | 33 | 9 | 9 | 0 | 13 | 3 | 2 | 0 | 0 | 0 | 9 | 0 | 0 | 2 |
| OTU418 | 0 | 1 | 0 | 0 | 0 | 0 | 10 | 3 | 1 | 4 | 5 | 11 | 4 | 4 | 1 | 0 | 0 | 0 |
| OTU419 | 2 | 5 | 9 | 1 | 8 | 4 | 34 | 21 | 22 | 31 | 51 | 17 | 0 | 7 | 18 | 0 | 3 | 0 |
| OTU42 | 194 | 58 | 86 | 2 | 66 | 125 | 307 | 114 | 228 | 247 | 74 | 48 | 236 | 13 | 142 | 14 | 28 | 38 |
| OTU420 | 3 | 4 | 2 | 2 | 6 | 2 | 8 | 6 | 8 | 5 | 4 | 3 | 7 | 2 | 2 | 11 | 4 | 4 |
| OTU421 | 0 | 0 | 0 | 0 | 0 | 0 | 2 | 8 | 15 | 19 | 32 | 5 | 2 | 4 | 8 | 0 | 1 | 1 |
| OTU422 | 0 | 3 | 0 | 0 | 1 | 16 | 20 | 13 | 0 | 5 | 13 | 0 | 0 | 0 | 1 | 0 | 0 | 0 |
| OTU423 | 0 | 0 | 0 | 0 | 0 | 0 | 24 | 7 | 2 | 13 | 11 | 11 | 0 | 2 | 10 | 0 | 0 | 1 |
| OTU424 | 26 | 22 | 10 | 11 | 20 | 37 | 170 | 100 | 126 | 0 | 7 | 157 | 17 | 58 | 105 | 0 | 7 | 1 |
| OTU425 | 0 | 7 | 4 | 3 | 9 | 5 | 7 | 49 | 24 | 49 | 57 | 26 | 1 | 4 | 10 | 0 | 2 | 2 |
| OTU426 | 2 | 1 | 4 | 1 | 2 | 0 | 1 | 6 | 1 | 1 | 1 | 5 | 0 | 10 | 4 | 0 | 0 | 2 |
| OTU427 | 0 | 0 | 0 | 0 | 0 | 0 | 4 | 0 | 3 | 3 | 0 | 14 | 1 | 7 | 6 | 0 | 0 | 1 |
| OTU428 | 0 | 0 | 0 | 0 | 0 | 0 | 0 | 1 | 0 | 1 | 0 | 0 | 10 | 10 | 0 | 0 | 46 | 16 |
| OTU429 | 0 | 0 | 0 | 0 | 0 | 0 | 0 | 0 | 0 | 0 | 0 | 0 | 4 | 7 | 0 | 0 | 37 | 29 |
| OTU43 | 286 | 86 | 465 | 156 | 317 | 225 | 2 | 0 | 0 | 2 | 2 | 1 | 1 | 1 | 3 | 0 | 1 | 0 |
| OTU430 | 0 | 0 | 0 | 0 | 0 | 0 | 0 | 0 | 0 | 0 | 1 | 0 | 0 | 0 | 0 | 0 | 17 | 0 |
| OTU431 | 0 | 0 | 0 | 0 | 1 | 1 | 0 | 0 | 0 | 0 | 0 | 0 | 2 | 4 | 0 | 1 | 63 | 19 |
| OTU432 | 71 | 7 | 4 | 76 | 1 | 3 | 49 | 25 | 24 | 7 | 5 | 15 | 1 | 6 | 20 | 0 | 4 | 0 |
| OTU433 | 7 | 6 | 10 | 5 | 8 | 5 | 7 | 10 | 11 | 13 | 9 | 10 | 4 | 2 | 9 | 5 | 14 | 6 |
| OTU434 | 6 | 13 | 3 | 3 | 9 | 11 | 0 | 0 | 0 | 0 | 0 | 0 | 0 | 0 | 1 | 0 | 0 | 0 |
| OTU435 | 6 | 1 | 2 | 3 | 4 | 4 | 1 | 4 | 0 | 0 | 0 | 1 | 0 | 1 | 1 | 0 | 0 | 0 |
| OTU436 | 6 | 35 | 10 | 0 | 0 | 7 | 0 | 1 | 0 | 0 | 0 | 0 | 0 | 0 | 0 | 0 | 0 | 0 |
| OTU437 | 28 | 1 | 1 | 36 | 7 | 63 | 4 | 28 | 6 | 8 | 6 | 6 | 1 | 0 | 5 | 0 | 0 | 0 |
| OTU438 | 2 | 8 | 6 | 2 | 8 | 6 | 0 | 0 | 0 | 1 | 1 | 0 | 0 | 0 | 0 | 0 | 0 | 0 |
| OTU439 | 7 | 3 | 4 | 0 | 1 | 8 | 7 | 7 | 0 | 0 | 0 | 6 | 0 | 0 | 2 | 0 | 0 | 0 |
| OTU44 | 0 | 1 | 1 | 0 | 1 | 0 | 1 | 2 | 3 | 2 | 4 | 2 | 3 | 4 | 1 | 1 | 1037 | 4 |
| OTU440 | 0 | 105 | 1 | 0 | 0 | 0 | 0 | 0 | 0 | 1 | 0 | 1 | 0 | 0 | 0 | 0 | 0 | 0 |
| OTU441 | 0 | 12 | 1 | 4 | 0 | 2 | 1 | 34 | 9 | 16 | 13 | 6 | 0 | 0 | 9 | 0 | 1 | 2 |
| OTU442 | 1 | 1 | 13 | 7 | 14 | 6 | 0 | 0 | 0 | 1 | 0 | 0 | 0 | 0 | 0 | 0 | 0 | 0 |
| OTU443 | 10 | 28 | 21 | 3 | 4 | 6 | 1 | 0 | 0 | 0 | 0 | 0 | 0 | 0 | 0 | 0 | 1 | 0 |
| OTU444 | 0 | 0 | 12 | 0 | 0 | 0 | 2 | 5 | 7 | 0 | 0 | 2 | 0 | 0 | 4 | 0 | 0 | 0 |
| OTU445 | 4 | 0 | 1 | 3 | 0 | 20 | 0 | 0 | 0 | 1 | 2 | 3 | 0 | 0 | 0 | 0 | 0 | 0 |
| OTU446 | 0 | 2 | 16 | 1 | 0 | 1 | 7 | 0 | 2 | 0 | 0 | 1 | 0 | 0 | 1 | 0 | 0 | 0 |
| OTU447 | 1 | 0 | 34 | 0 | 0 | 2 | 0 | 0 | 0 | 0 | 0 | 0 | 0 | 0 | 0 | 0 | 1 | 0 |
| OTU448 | 1 | 0 | 6 | 0 | 0 | 3 | 1 | 0 | 1 | 0 | 0 | 4 | 0 | 0 | 1 | 0 | 0 | 0 |
| OTU449 | 0 | 0 | 0 | 3 | 32 | 4 | 1 | 0 | 2 | 10 | 8 | 10 | 0 | 1 | 1 | 0 | 1 | 0 |
| OTU45 | 329 | 69 | 397 | 926 | 682 | 1049 | 94 | 18 | 14 | 29 | 66 | 25 | 2 | 25 | 17 | 0 | 9 | 7 |
| OTU450 | 2 | 6 | 3 | 2 | 2 | 5 | 4 | 3 | 1 | 4 | 6 | 5 | 2 | 5 | 2 | 2 | 10 | 1 |
| OTU451 | 9 | 12 | 9 | 8 | 8 | 21 | 8 | 21 | 11 | 23 | 23 | 22 | 4 | 12 | 13 | 3 | 30 | 8 |
| OTU452 | 0 | 4 | 0 | 4 | 5 | 11 | 1 | 0 | 0 | 1 | 2 | 9 | 0 | 3 | 0 | 0 | 0 | 0 |
| OTU453 | 4 | 1 | 0 | 3 | 11 | 12 | 0 | 14 | 11 | 22 | 19 | 12 | 1 | 0 | 7 | 0 | 1 | 0 |
| OTU454 | 1 | 12 | 0 | 0 | 11 | 3 | 4 | 1 | 8 | 4 | 5 | 5 | 1 | 1 | 5 | 0 | 0 | 0 |
| OTU455 | 1 | 6 | 5 | 0 | 4 | 7 | 0 | 0 | 2 | 5 | 3 | 2 | 1 | 0 | 1 | 0 | 0 | 0 |
| OTU456 | 0 | 0 | 0 | 0 | 1 | 1 | 22 | 5 | 32 | 4 | 8 | 10 | 0 | 0 | 21 | 0 | 1 | 0 |
| OTU457 | 0 | 0 | 0 | 0 | 14 | 0 | 0 | 0 | 0 | 0 | 0 | 0 | 0 | 0 | 0 | 0 | 0 | 0 |
| OTU458 | 0 | 2 | 0 | 0 | 0 | 5 | 3 | 15 | 4 | 14 | 7 | 0 | 0 | 0 | 3 | 0 | 0 | 1 |
| OTU459 | 4 | 7 | 4 | 2 | 6 | 7 | 5 | 5 | 7 | 9 | 10 | 4 | 1 | 9 | 3 | 8 | 5 | 4 |
| OTU46 | 3 | 82 | 2 | 118 | 1 | 223 | 10 | 33 | 265 | 9 | 240 | 169 | 8 | 10 | 198 | 0 | 7 | 14 |
| OTU460 | 0 | 0 | 0 | 1 | 0 | 1 | 3 | 62 | 1 | 0 | 5 | 4 | 0 | 6 | 2 | 0 | 0 | 0 |
| OTU461 | 1 | 20 | 12 | 0 | 1 | 5 | 11 | 11 | 7 | 13 | 8 | 16 | 0 | 5 | 6 | 0 | 1 | 1 |
| OTU462 | 0 | 0 | 0 | 0 | 0 | 0 | 3 | 3 | 3 | 12 | 8 | 2 | 2 | 2 | 2 | 0 | 1 | 1 |
| OTU463 | 2 | 0 | 0 | 1 | 2 | 3 | 4 | 3 | 11 | 0 | 4 | 3 | 1 | 1 | 6 | 0 | 0 | 0 |
| OTU464 | 2 | 0 | 0 | 0 | 0 | 0 | 3 | 6 | 0 | 33 | 58 | 0 | 0 | 0 | 0 | 0 | 0 | 1 |
| OTU465 | 0 | 0 | 0 | 0 | 0 | 1 | 6 | 18 | 8 | 25 | 12 | 2 | 1 | 1 | 15 | 0 | 1 | 0 |
| OTU466 | 0 | 0 | 0 | 0 | 0 | 0 | 0 | 2 | 0 | 1 | 6 | 26 | 0 | 0 | 0 | 0 | 0 | 1 |
| OTU467 | 0 | 3 | 2 | 1 | 4 | 6 | 0 | 10 | 3 | 19 | 14 | 0 | 0 | 0 | 1 | 0 | 0 | 0 |
| OTU468 | 0 | 0 | 0 | 0 | 0 | 0 | 6 | 7 | 13 | 19 | 13 | 30 | 5 | 5 | 8 | 0 | 0 | 2 |
| OTU469 | 0 | 0 | 0 | 1 | 0 | 2 | 0 | 29 | 0 | 2 | 1 | 1 | 1 | 0 | 1 | 0 | 1 | 0 |
| OTU47 | 215 | 145 | 255 | 444 | 289 | 418 | 168 | 58 | 65 | 153 | 124 | 145 | 13 | 36 | 63 | 0 | 5 | 7 |
| OTU470 | 0 | 0 | 4 | 1 | 0 | 2 | 2 | 10 | 7 | 4 | 6 | 4 | 1 | 1 | 8 | 0 | 0 | 0 |
| OTU471 | 0 | 5 | 5 | 0 | 1 | 0 | 0 | 6 | 5 | 2 | 0 | 5 | 3 | 11 | 1 | 0 | 1 | 1 |
| OTU472 | 1 | 0 | 0 | 0 | 0 | 0 | 2 | 4 | 4 | 1 | 0 | 12 | 3 | 7 | 8 | 0 | 0 | 1 |
| OTU473 | 0 | 1 | 0 | 1 | 1 | 0 | 2 | 6 | 0 | 3 | 4 | 4 | 0 | 1 | 1 | 0 | 1 | 0 |
| OTU474 | 0 | 0 | 0 | 0 | 0 | 0 | 0 | 1 | 2 | 7 | 2 | 8 | 0 | 0 | 5 | 0 | 0 | 0 |
| OTU475 | 1 | 7 | 5 | 3 | 3 | 2 | 1 | 4 | 6 | 4 | 5 | 4 | 3 | 6 | 1 | 6 | 4 | 2 |
| OTU476 | 11 | 28 | 48 | 37 | 106 | 103 | 26 | 517 | 360 | 490 | 338 | 576 | 13 | 47 | 216 | 0 | 30 | 15 |
| OTU477 | 0 | 0 | 0 | 0 | 0 | 0 | 5 | 3 | 6 | 7 | 15 | 15 | 3 | 8 | 13 | 0 | 2 | 0 |
| OTU478 | 0 | 0 | 0 | 0 | 0 | 0 | 0 | 0 | 0 | 0 | 0 | 0 | 8 | 1 | 0 | 0 | 1 | 6 |
| OTU479 | 0 | 0 | 0 | 0 | 0 | 0 | 0 | 0 | 0 | 0 | 0 | 0 | 10 | 2 | 0 | 0 | 29 | 8 |
| OTU48 | 164 | 271 | 263 | 458 | 326 | 131 | 3 | 0 | 1 | 3 | 2 | 2 | 0 | 1 | 1 | 0 | 2 | 1 |
| OTU480 | 0 | 0 | 0 | 0 | 0 | 0 | 0 | 0 | 0 | 0 | 0 | 0 | 15 | 2 | 0 | 0 | 17 | 7 |
| OTU481 | 0 | 0 | 0 | 0 | 0 | 0 | 0 | 0 | 1 | 1 | 0 | 0 | 1 | 16 | 0 | 0 | 21 | 2 |
| OTU482 | 0 | 0 | 0 | 0 | 0 | 0 | 0 | 1 | 1 | 0 | 0 | 0 | 0 | 1 | 0 | 0 | 80 | 0 |
| OTU483 | 0 | 0 | 0 | 0 | 0 | 0 | 0 | 0 | 0 | 0 | 0 | 0 | 0 | 0 | 0 | 0 | 86 | 0 |
| OTU484 | 0 | 0 | 1 | 0 | 0 | 0 | 0 | 0 | 0 | 0 | 0 | 0 | 0 | 0 | 0 | 0 | 42 | 0 |
| OTU485 | 2 | 0 | 0 | 0 | 0 | 0 | 1 | 0 | 0 | 0 | 0 | 0 | 0 | 0 | 0 | 0 | 58 | 0 |
| OTU486 | 0 | 0 | 0 | 0 | 0 | 0 | 0 | 0 | 0 | 0 | 0 | 0 | 0 | 0 | 0 | 1 | 66 | 2 |
| OTU487 | 1 | 0 | 0 | 0 | 0 | 1 | 0 | 0 | 1 | 1 | 0 | 0 | 3 | 4 | 3 | 2 | 27 | 10 |
| OTU488 | 11 | 0 | 6 | 11 | 0 | 20 | 0 | 0 | 0 | 0 | 0 | 0 | 0 | 0 | 0 | 0 | 0 | 0 |
| OTU489 | 11 | 4 | 5 | 4 | 4 | 4 | 0 | 1 | 5 | 1 | 2 | 6 | 0 | 1 | 4 | 0 | 0 | 0 |
| OTU49 | 296 | 359 | 474 | 438 | 377 | 249 | 72 | 45 | 20 | 77 | 20 | 35 | 3 | 7 | 10 | 0 | 6 | 4 |
| OTU490 | 10 | 13 | 23 | 6 | 2 | 16 | 2 | 26 | 5 | 12 | 13 | 12 | 0 | 1 | 6 | 0 | 4 | 0 |
| OTU491 | 4 | 4 | 6 | 4 | 10 | 40 | 1 | 0 | 4 | 1 | 1 | 5 | 0 | 0 | 1 | 0 | 0 | 0 |
| OTU492 | 11 | 4 | 0 | 1 | 1 | 28 | 0 | 0 | 1 | 0 | 1 | 0 | 0 | 0 | 0 | 0 | 0 | 0 |
| OTU493 | 3 | 3 | 5 | 1 | 3 | 1 | 2 | 2 | 1 | 0 | 1 | 3 | 0 | 1 | 0 | 0 | 0 | 0 |
| OTU494 | 7 | 2 | 2 | 4 | 9 | 0 | 3 | 3 | 2 | 4 | 6 | 5 | 1 | 2 | 8 | 0 | 0 | 0 |
| OTU495 | 6 | 12 | 10 | 0 | 0 | 0 | 0 | 0 | 0 | 0 | 0 | 0 | 0 | 0 | 0 | 0 | 0 | 0 |
| OTU496 | 9 | 0 | 14 | 9 | 7 | 7 | 0 | 0 | 0 | 0 | 0 | 0 | 0 | 0 | 0 | 0 | 0 | 0 |
| OTU497 | 3 | 3 | 0 | 3 | 11 | 3 | 0 | 0 | 0 | 0 | 0 | 0 | 0 | 0 | 0 | 0 | 0 | 0 |
| OTU498 | 3 | 4 | 3 | 2 | 1 | 4 | 0 | 4 | 3 | 1 | 7 | 6 | 4 | 7 | 3 | 3 | 6 | 3 |
| OTU499 | 3 | 1 | 6 | 0 | 0 | 2 | 0 | 0 | 0 | 0 | 0 | 0 | 0 | 0 | 0 | 0 | 3 | 1 |
| OTU5 | 3663 | 12707 | 1740 | 1318 | 1441 | 1321 | 0 | 7 | 4 | 7 | 7 | 10 | 6 | 6 | 1 | 3 | 14 | 3 |
| OTU50 | 6 | 55 | 1515 | 8 | 7 | 10 | 1 | 1 | 0 | 0 | 3 | 1 | 0 | 0 | 1 | 0 | 2 | 0 |
| OTU500 | 11 | 1 | 6 | 0 | 1 | 6 | 2 | 1 | 5 | 0 | 0 | 4 | 0 | 0 | 0 | 0 | 3 | 1 |
| OTU501 | 9 | 11 | 4 | 0 | 2 | 1 | 1 | 0 | 1 | 1 | 1 | 3 | 0 | 0 | 0 | 0 | 0 | 0 |
| OTU502 | 38 | 1 | 0 | 0 | 0 | 8 | 0 | 0 | 0 | 0 | 0 | 0 | 0 | 0 | 0 | 0 | 0 | 0 |
| OTU503 | 44 | 1 | 4 | 3 | 0 | 4 | 3 | 0 | 3 | 0 | 5 | 1 | 0 | 1 | 5 | 0 | 1 | 0 |
| OTU504 | 7 | 1 | 3 | 9 | 2 | 14 | 0 | 0 | 0 | 0 | 0 | 0 | 0 | 0 | 0 | 0 | 0 | 0 |
| OTU505 | 4 | 11 | 8 | 7 | 3 | 10 | 1 | 0 | 0 | 0 | 0 | 0 | 0 | 0 | 0 | 0 | 0 | 0 |
| OTU506 | 1 | 4 | 0 | 3 | 7 | 1 | 2 | 2 | 1 | 0 | 1 | 0 | 0 | 0 | 1 | 0 | 0 | 1 |
| OTU507 | 0 | 1 | 0 | 0 | 5 | 2 | 0 | 2 | 0 | 2 | 3 | 0 | 0 | 0 | 3 | 0 | 0 | 0 |
| OTU508 | 2 | 3 | 23 | 3 | 1 | 0 | 5 | 0 | 0 | 0 | 0 | 0 | 0 | 0 | 0 | 0 | 0 | 0 |
| OTU509 | 7 | 7 | 7 | 1 | 0 | 5 | 0 | 0 | 0 | 0 | 0 | 0 | 0 | 0 | 0 | 0 | 0 | 0 |
| OTU51 | 1 | 1 | 126 | 847 | 37 | 4 | 63 | 27 | 1 | 26 | 96 | 52 | 8 | 11 | 0 | 1 | 9 | 5 |
| OTU510 | 0 | 1 | 2 | 3 | 1 | 2 | 0 | 12 | 1 | 1 | 1 | 0 | 0 | 0 | 0 | 0 | 0 | 0 |
| OTU511 | 3 | 5 | 4 | 0 | 5 | 4 | 0 | 0 | 0 | 0 | 1 | 0 | 0 | 0 | 0 | 0 | 0 | 0 |
| OTU512 | 0 | 48 | 0 | 0 | 0 | 0 | 0 | 0 | 0 | 0 | 0 | 0 | 0 | 0 | 0 | 0 | 0 | 0 |
| OTU513 | 1 | 4 | 5 | 3 | 10 | 16 | 1 | 4 | 3 | 12 | 6 | 2 | 0 | 0 | 3 | 0 | 0 | 0 |
| OTU514 | 0 | 2 | 54 | 0 | 0 | 1 | 4 | 19 | 12 | 19 | 6 | 15 | 0 | 1 | 8 | 0 | 1 | 0 |
| OTU515 | 0 | 2 | 2 | 0 | 16 | 0 | 1 | 5 | 5 | 3 | 2 | 2 | 0 | 1 | 2 | 0 | 1 | 1 |
| OTU516 | 0 | 2 | 1 | 0 | 0 | 0 | 1 | 3 | 0 | 9 | 5 | 1 | 0 | 0 | 0 | 0 | 0 | 0 |
| OTU517 | 7 | 1 | 0 | 17 | 5 | 12 | 3 | 4 | 1 | 1 | 1 | 0 | 0 | 1 | 3 | 0 | 0 | 0 |
| OTU518 | 1 | 0 | 1 | 5 | 0 | 8 | 2 | 0 | 0 | 0 | 0 | 1 | 1 | 0 | 0 | 0 | 2 | 0 |
| OTU519 | 5 | 1 | 2 | 4 | 2 | 3 | 1 | 0 | 0 | 0 | 0 | 2 | 0 | 0 | 0 | 0 | 0 | 0 |
| OTU52 | 3 | 0 | 2 | 3 | 1 | 4 | 682 | 172 | 5 | 179 | 154 | 89 | 10 | 17 | 22 | 1 | 7 | 6 |
| OTU520 | 2 | 6 | 2 | 7 | 11 | 6 | 0 | 0 | 0 | 0 | 0 | 0 | 0 | 0 | 0 | 0 | 0 | 0 |
| OTU521 | 1 | 0 | 0 | 0 | 2 | 2 | 1 | 1 | 1 | 1 | 3 | 2 | 1 | 1 | 1 | 0 | 1 | 0 |
| OTU522 | 3 | 1 | 0 | 1 | 1 | 1 | 0 | 2 | 3 | 1 | 5 | 4 | 1 | 0 | 2 | 2 | 3 | 2 |
| OTU523 | 1 | 0 | 0 | 1 | 1 | 1 | 1 | 0 | 0 | 3 | 1 | 0 | 1 | 1 | 1 | 1 | 0 | 1 |
| OTU524 | 1 | 0 | 2 | 1 | 29 | 1 | 0 | 1 | 2 | 1 | 13 | 1 | 1 | 0 | 1 | 0 | 1 | 0 |
| OTU525 | 0 | 0 | 0 | 0 | 0 | 5 | 0 | 0 | 5 | 0 | 0 | 0 | 0 | 0 | 0 | 0 | 0 | 0 |
| OTU526 | 1 | 0 | 0 | 0 | 0 | 0 | 10 | 1 | 5 | 21 | 4 | 1 | 0 | 0 | 3 | 0 | 0 | 0 |
| OTU527 | 8 | 0 | 3 | 0 | 2 | 2 | 18 | 4 | 12 | 3 | 4 | 7 | 2 | 1 | 7 | 0 | 0 | 0 |
| OTU528 | 0 | 0 | 0 | 0 | 0 | 0 | 8 | 0 | 17 | 10 | 1 | 3 | 0 | 0 | 7 | 0 | 0 | 1 |
| OTU529 | 0 | 0 | 0 | 0 | 0 | 0 | 2 | 6 | 3 | 3 | 0 | 5 | 0 | 4 | 3 | 0 | 0 | 2 |
| OTU53 | 3 | 8 | 71 | 2 | 49 | 96 | 72 | 190 | 249 | 52 | 912 | 110 | 3 | 4 | 219 | 0 | 21 | 5 |
| OTU530 | 0 | 1 | 0 | 1 | 1 | 1 | 18 | 9 | 2 | 12 | 6 | 8 | 2 | 2 | 1 | 0 | 2 | 0 |
| OTU531 | 0 | 0 | 0 | 0 | 0 | 0 | 2 | 1 | 1 | 1 | 3 | 2 | 1 | 1 | 5 | 0 | 0 | 0 |
| OTU532 | 0 | 0 | 0 | 0 | 0 | 0 | 9 | 0 | 0 | 0 | 0 | 0 | 0 | 0 | 0 | 0 | 0 | 0 |
| OTU533 | 2 | 0 | 0 | 2 | 7 | 0 | 14 | 2 | 2 | 2 | 1 | 0 | 0 | 0 | 1 | 0 | 0 | 0 |
| OTU534 | 0 | 0 | 0 | 0 | 0 | 0 | 4 | 4 | 3 | 30 | 4 | 4 | 0 | 2 | 0 | 0 | 0 | 0 |
| OTU535 | 1 | 0 | 0 | 0 | 0 | 0 | 1 | 28 | 4 | 2 | 0 | 0 | 0 | 0 | 3 | 0 | 1 | 1 |
| OTU536 | 0 | 1 | 0 | 0 | 0 | 0 | 0 | 7 | 2 | 7 | 3 | 1 | 0 | 0 | 2 | 0 | 0 | 0 |
| OTU537 | 0 | 0 | 0 | 0 | 0 | 0 | 0 | 5 | 1 | 0 | 1 | 4 | 0 | 0 | 1 | 0 | 1 | 0 |
| OTU538 | 16 | 3 | 4 | 1 | 0 | 14 | 7 | 5 | 9 | 0 | 0 | 5 | 0 | 1 | 4 | 0 | 1 | 0 |
| OTU539 | 1 | 0 | 2 | 0 | 2 | 4 | 2 | 7 | 0 | 11 | 3 | 1 | 0 | 0 | 1 | 0 | 0 | 0 |
| OTU54 | 222 | 160 | 234 | 282 | 524 | 501 | 4 | 1 | 1 | 5 | 4 | 0 | 0 | 3 | 5 | 0 | 2 | 2 |
| OTU540 | 0 | 0 | 0 | 0 | 0 | 0 | 0 | 9 | 8 | 0 | 0 | 6 | 1 | 0 | 10 | 0 | 0 | 0 |
| OTU541 | 0 | 0 | 0 | 0 | 0 | 0 | 1 | 6 | 11 | 1 | 2 | 3 | 0 | 0 | 2 | 0 | 0 | 0 |
| OTU542 | 1 | 0 | 0 | 0 | 0 | 2 | 1 | 4 | 4 | 3 | 5 | 4 | 0 | 1 | 10 | 0 | 0 | 0 |
| OTU543 | 0 | 1 | 0 | 0 | 0 | 2 | 1 | 29 | 0 | 50 | 5 | 3 | 0 | 0 | 1 | 0 | 0 | 1 |
| OTU544 | 0 | 5 | 2 | 1 | 0 | 14 | 0 | 15 | 1 | 24 | 24 | 5 | 2 | 2 | 5 | 0 | 1 | 0 |
| OTU545 | 10 | 4 | 5 | 7 | 1 | 2 | 4 | 13 | 3 | 2 | 3 | 6 | 0 | 0 | 2 | 0 | 0 | 0 |
| OTU546 | 0 | 0 | 0 | 0 | 0 | 0 | 0 | 1 | 8 | 0 | 0 | 24 | 0 | 1 | 9 | 0 | 0 | 0 |
| OTU547 | 0 | 0 | 0 | 0 | 0 | 4 | 13 | 31 | 11 | 9 | 16 | 31 | 2 | 5 | 14 | 0 | 1 | 0 |
| OTU548 | 1 | 0 | 0 | 0 | 0 | 1 | 2 | 2 | 5 | 25 | 1 | 3 | 0 | 1 | 1 | 0 | 2 | 2 |
| OTU549 | 0 | 0 | 0 | 0 | 0 | 0 | 6 | 0 | 0 | 15 | 2 | 1 | 0 | 12 | 2 | 0 | 0 | 2 |
| OTU55 | 80 | 144 | 212 | 241 | 232 | 218 | 47 | 16 | 127 | 1896 | 1661 | 29 | 47 | 63 | 101 | 1 | 26 | 37 |
| OTU550 | 0 | 0 | 0 | 0 | 0 | 0 | 4 | 0 | 1 | 2 | 0 | 4 | 0 | 1 | 0 | 0 | 0 | 0 |
| OTU551 | 0 | 1 | 0 | 0 | 0 | 0 | 3 | 1 | 0 | 8 | 14 | 4 | 1 | 1 | 3 | 0 | 0 | 0 |
| OTU552 | 1 | 4 | 6 | 2 | 2 | 3 | 1 | 5 | 4 | 2 | 4 | 8 | 0 | 2 | 2 | 0 | 0 | 0 |
| OTU553 | 0 | 0 | 0 | 0 | 0 | 1 | 1 | 0 | 6 | 0 | 20 | 0 | 0 | 1 | 3 | 0 | 1 | 0 |
| OTU554 | 0 | 0 | 0 | 0 | 0 | 0 | 0 | 1 | 0 | 0 | 0 | 8 | 0 | 2 | 0 | 0 | 0 | 0 |
| OTU555 | 0 | 0 | 0 | 0 | 0 | 0 | 0 | 0 | 0 | 0 | 0 | 0 | 2 | 5 | 0 | 1 | 10 | 6 |
| OTU556 | 0 | 0 | 0 | 0 | 0 | 0 | 0 | 0 | 0 | 0 | 0 | 0 | 9 | 1 | 0 | 0 | 25 | 12 |
| OTU557 | 8 | 3 | 3 | 0 | 5 | 0 | 0 | 0 | 0 | 0 | 0 | 0 | 5 | 0 | 0 | 0 | 15 | 1 |
| OTU558 | 0 | 0 | 0 | 0 | 0 | 0 | 0 | 0 | 0 | 0 | 0 | 0 | 0 | 2 | 0 | 1 | 18 | 2 |
| OTU559 | 0 | 0 | 0 | 0 | 0 | 0 | 1 | 3 | 1 | 1 | 1 | 1 | 1 | 3 | 0 | 4 | 19 | 3 |
| OTU56 | 0 | 10 | 5 | 17 | 3 | 31 | 11 | 528 | 154 | 331 | 83 | 123 | 4 | 6 | 142 | 0 | 11 | 3 |
| OTU560 | 0 | 0 | 0 | 0 | 0 | 0 | 0 | 0 | 0 | 0 | 0 | 0 | 1 | 1 | 0 | 1 | 19 | 3 |
| OTU561 | 0 | 0 | 1 | 0 | 2 | 0 | 0 | 0 | 0 | 0 | 0 | 0 | 1 | 0 | 1 | 0 | 62 | 1 |
| OTU562 | 0 | 1 | 0 | 1 | 0 | 0 | 0 | 0 | 0 | 0 | 0 | 0 | 7 | 5 | 0 | 0 | 71 | 19 |
| OTU563 | 0 | 0 | 0 | 0 | 0 | 0 | 0 | 0 | 0 | 0 | 0 | 0 | 0 | 0 | 0 | 0 | 35 | 0 |
| OTU564 | 0 | 0 | 0 | 0 | 0 | 0 | 0 | 0 | 0 | 0 | 0 | 0 | 0 | 0 | 0 | 0 | 152 | 1 |
| OTU565 | 0 | 0 | 0 | 0 | 0 | 0 | 0 | 0 | 0 | 0 | 0 | 0 | 1 | 1 | 0 | 1 | 14 | 17 |
| OTU566 | 2 | 1 | 1 | 1 | 0 | 0 | 0 | 0 | 0 | 0 | 0 | 0 | 0 | 0 | 0 | 0 | 1 | 0 |
| OTU567 | 10 | 0 | 0 | 0 | 3 | 0 | 0 | 0 | 0 | 0 | 0 | 1 | 1 | 1 | 0 | 0 | 0 | 0 |
| OTU568 | 2 | 2 | 18 | 0 | 0 | 0 | 0 | 0 | 0 | 0 | 0 | 0 | 0 | 0 | 0 | 0 | 0 | 0 |
| OTU569 | 5 | 1 | 1 | 1 | 0 | 0 | 0 | 0 | 1 | 1 | 1 | 1 | 3 | 1 | 0 | 4 | 4 | 3 |
| OTU57 | 0 | 0 | 0 | 0 | 2 | 0 | 39 | 193 | 218 | 228 | 67 | 195 | 2 | 4 | 99 | 0 | 3 | 1 |
| OTU570 | 3 | 3 | 0 | 0 | 6 | 2 | 0 | 4 | 1 | 2 | 0 | 1 | 0 | 2 | 3 | 0 | 0 | 2 |
| OTU571 | 8 | 4 | 8 | 1 | 5 | 3 | 1 | 3 | 2 | 1 | 1 | 2 | 0 | 4 | 2 | 0 | 1 | 1 |
| OTU572 | 9 | 4 | 2 | 0 | 7 | 0 | 0 | 0 | 0 | 0 | 0 | 0 | 0 | 0 | 0 | 0 | 0 | 0 |
| OTU573 | 5 | 1 | 1 | 1 | 1 | 4 | 0 | 0 | 0 | 1 | 1 | 0 | 0 | 0 | 0 | 0 | 0 | 0 |
| OTU574 | 12 | 0 | 30 | 0 | 1 | 0 | 1 | 0 | 0 | 1 | 0 | 2 | 0 | 0 | 0 | 0 | 0 | 0 |
| OTU575 | 1 | 1 | 0 | 2 | 1 | 2 | 0 | 0 | 0 | 0 | 0 | 0 | 0 | 0 | 0 | 0 | 0 | 0 |
| OTU576 | 4 | 2 | 1 | 0 | 1 | 3 | 0 | 7 | 3 | 0 | 0 | 3 | 0 | 1 | 6 | 0 | 1 | 0 |
| OTU577 | 7 | 1 | 1 | 8 | 1 | 0 | 8 | 6 | 0 | 3 | 0 | 4 | 1 | 2 | 3 | 0 | 0 | 0 |
| OTU578 | 1 | 2 | 0 | 4 | 1 | 4 | 1 | 1 | 1 | 1 | 0 | 2 | 0 | 1 | 1 | 0 | 0 | 0 |
| OTU579 | 7 | 1 | 0 | 0 | 4 | 1 | 4 | 1 | 2 | 0 | 1 | 1 | 5 | 3 | 1 | 6 | 3 | 7 |
| OTU58 | 513 | 16 | 343 | 13 | 33 | 109 | 570 | 54 | 69 | 75 | 40 | 34 | 1 | 19 | 62 | 0 | 10 | 2 |
| OTU580 | 11 | 0 | 0 | 0 | 0 | 0 | 2 | 0 | 0 | 6 | 0 | 0 | 0 | 0 | 0 | 0 | 0 | 0 |
| OTU581 | 12 | 0 | 0 | 0 | 1 | 0 | 0 | 0 | 0 | 0 | 0 | 0 | 0 | 0 | 0 | 0 | 0 | 0 |
| OTU582 | 17 | 0 | 0 | 0 | 0 | 0 | 0 | 0 | 0 | 0 | 0 | 0 | 0 | 0 | 0 | 0 | 0 | 0 |
| OTU583 | 1 | 0 | 6 | 1 | 2 | 4 | 0 | 0 | 0 | 0 | 0 | 0 | 0 | 0 | 0 | 0 | 0 | 0 |
| OTU584 | 5 | 11 | 0 | 8 | 4 | 0 | 0 | 0 | 0 | 0 | 0 | 0 | 0 | 0 | 0 | 0 | 1 | 0 |
| OTU585 | 0 | 20 | 20 | 0 | 0 | 0 | 0 | 0 | 0 | 7 | 1 | 0 | 0 | 0 | 2 | 0 | 0 | 0 |
| OTU586 | 0 | 3 | 3 | 3 | 2 | 0 | 1 | 3 | 0 | 0 | 1 | 0 | 2 | 5 | 4 | 2 | 0 | 0 |
| OTU587 | 1 | 4 | 4 | 3 | 7 | 5 | 2 | 5 | 1 | 1 | 1 | 2 | 0 | 3 | 2 | 0 | 1 | 0 |
| OTU588 | 2 | 25 | 6 | 5 | 9 | 7 | 0 | 9 | 5 | 9 | 10 | 2 | 0 | 0 | 4 | 0 | 1 | 0 |
| OTU589 | 1 | 1 | 2 | 4 | 0 | 2 | 2 | 0 | 6 | 0 | 0 | 0 | 0 | 0 | 2 | 0 | 0 | 0 |
| OTU59 | 227 | 16 | 96 | 401 | 74 | 204 | 4 | 2 | 3 | 0 | 0 | 1 | 0 | 1 | 1 | 0 | 4 | 1 |
| OTU590 | 0 | 7 | 1 | 0 | 6 | 0 | 0 | 0 | 0 | 1 | 3 | 2 | 0 | 0 | 0 | 0 | 0 | 0 |
| OTU591 | 2 | 5 | 0 | 3 | 0 | 6 | 4 | 0 | 0 | 1 | 0 | 2 | 1 | 2 | 0 | 0 | 0 | 0 |
| OTU592 | 8 | 2 | 0 | 0 | 6 | 15 | 0 | 0 | 0 | 0 | 0 | 0 | 0 | 0 | 0 | 0 | 0 | 0 |
| OTU593 | 6 | 15 | 0 | 0 | 0 | 3 | 0 | 0 | 0 | 0 | 0 | 0 | 0 | 0 | 0 | 0 | 0 | 0 |
| OTU594 | 0 | 0 | 3 | 0 | 0 | 0 | 0 | 0 | 0 | 0 | 1 | 0 | 0 | 0 | 0 | 0 | 0 | 0 |
| OTU595 | 1 | 0 | 1 | 0 | 1 | 1 | 0 | 7 | 7 | 4 | 5 | 2 | 1 | 1 | 4 | 0 | 0 | 0 |
| OTU596 | 4 | 0 | 3 | 1 | 0 | 5 | 0 | 1 | 5 | 13 | 12 | 4 | 1 | 0 | 3 | 0 | 1 | 0 |
| OTU597 | 0 | 0 | 2 | 0 | 2 | 0 | 2 | 0 | 2 | 1 | 2 | 0 | 0 | 1 | 0 | 2 | 5 | 1 |
| OTU598 | 0 | 1 | 4 | 7 | 1 | 2 | 0 | 1 | 0 | 0 | 1 | 0 | 0 | 1 | 0 | 0 | 0 | 0 |
| OTU599 | 0 | 1 | 0 | 3 | 7 | 8 | 1 | 0 | 4 | 8 | 3 | 3 | 0 | 0 | 3 | 0 | 0 | 0 |
| OTU6 | 5913 | 2348 | 6104 | 7558 | 6115 | 9119 | 94 | 32 | 41 | 74 | 74 | 59 | 8 | 14 | 39 | 1 | 30 | 12 |
| OTU60 | 3 | 4 | 7 | 7 | 4 | 9 | 233 | 108 | 425 | 204 | 70 | 284 | 38 | 111 | 445 | 0 | 7 | 13 |
| OTU600 | 0 | 0 | 1 | 1 | 1 | 2 | 0 | 0 | 0 | 0 | 0 | 0 | 0 | 0 | 0 | 0 | 0 | 0 |
| OTU601 | 1 | 1 | 2 | 3 | 3 | 3 | 1 | 4 | 1 | 3 | 0 | 4 | 0 | 1 | 4 | 0 | 0 | 0 |
| OTU602 | 0 | 0 | 0 | 2 | 1 | 1 | 1 | 2 | 2 | 2 | 1 | 0 | 2 | 0 | 0 | 0 | 0 | 0 |
| OTU603 | 0 | 0 | 1 | 2 | 2 | 0 | 1 | 2 | 0 | 0 | 0 | 1 | 0 | 2 | 0 | 0 | 0 | 0 |
| OTU604 | 2 | 3 | 2 | 6 | 8 | 18 | 1 | 0 | 2 | 0 | 0 | 4 | 0 | 0 | 0 | 0 | 0 | 0 |
| OTU605 | 2 | 0 | 0 | 3 | 7 | 0 | 0 | 0 | 0 | 1 | 0 | 2 | 0 | 0 | 0 | 0 | 0 | 0 |
| OTU606 | 1 | 0 | 0 | 7 | 2 | 3 | 0 | 0 | 3 | 6 | 2 | 2 | 0 | 0 | 3 | 0 | 0 | 0 |
| OTU607 | 6 | 2 | 8 | 3 | 23 | 5 | 10 | 24 | 18 | 45 | 50 | 7 | 3 | 2 | 17 | 0 | 4 | 3 |
| OTU608 | 2 | 0 | 2 | 18 | 4 | 0 | 0 | 0 | 0 | 0 | 0 | 0 | 0 | 0 | 0 | 0 | 0 | 0 |
| OTU609 | 0 | 0 | 5 | 6 | 0 | 0 | 0 | 1 | 1 | 0 | 0 | 0 | 0 | 0 | 1 | 0 | 0 | 0 |
| OTU61 | 117 | 78 | 122 | 193 | 398 | 603 | 100 | 59 | 79 | 49 | 58 | 101 | 9 | 25 | 36 | 0 | 5 | 2 |
| OTU610 | 1 | 0 | 0 | 37 | 0 | 3 | 0 | 1 | 1 | 0 | 1 | 0 | 0 | 0 | 0 | 0 | 0 | 0 |
| OTU611 | 2 | 1 | 3 | 4 | 0 | 12 | 1 | 2 | 1 | 1 | 0 | 3 | 0 | 2 | 0 | 0 | 0 | 0 |
| OTU612 | 0 | 0 | 0 | 0 | 4 | 10 | 0 | 0 | 0 | 3 | 0 | 0 | 0 | 0 | 0 | 0 | 0 | 0 |
| OTU613 | 0 | 0 | 0 | 0 | 2 | 16 | 0 | 0 | 0 | 0 | 0 | 1 | 0 | 0 | 0 | 0 | 0 | 0 |
| OTU614 | 1 | 0 | 0 | 0 | 4 | 2 | 1 | 2 | 1 | 0 | 0 | 2 | 0 | 0 | 1 | 0 | 0 | 0 |
| OTU615 | 1 | 0 | 1 | 2 | 5 | 2 | 0 | 0 | 0 | 0 | 0 | 0 | 0 | 0 | 0 | 0 | 0 | 0 |
| OTU616 | 0 | 1 | 3 | 0 | 3 | 0 | 1 | 0 | 1 | 1 | 1 | 1 | 0 | 0 | 1 | 0 | 0 | 0 |
| OTU617 | 0 | 2 | 0 | 1 | 10 | 8 | 0 | 4 | 4 | 1 | 0 | 3 | 0 | 0 | 3 | 0 | 1 | 1 |
| OTU618 | 0 | 0 | 2 | 1 | 0 | 10 | 0 | 1 | 0 | 0 | 0 | 0 | 0 | 0 | 0 | 0 | 0 | 0 |
| OTU619 | 0 | 0 | 1 | 0 | 2 | 11 | 3 | 8 | 3 | 7 | 5 | 4 | 0 | 3 | 2 | 0 | 0 | 0 |
| OTU62 | 1 | 1 | 1 | 0 | 0 | 1 | 0 | 50 | 199 | 3 | 4 | 425 | 22 | 58 | 129 | 0 | 8 | 1 |
| OTU620 | 0 | 0 | 1 | 3 | 1 | 4 | 0 | 11 | 7 | 4 | 3 | 2 | 0 | 0 | 1 | 0 | 0 | 0 |
| OTU621 | 0 | 1 | 1 | 0 | 4 | 8 | 0 | 5 | 10 | 4 | 4 | 8 | 0 | 1 | 3 | 0 | 4 | 0 |
| OTU622 | 4 | 4 | 1 | 2 | 9 | 8 | 1 | 3 | 8 | 4 | 8 | 7 | 2 | 6 | 3 | 3 | 4 | 1 |
| OTU623 | 9 | 5 | 4 | 3 | 3 | 8 | 1 | 11 | 7 | 4 | 11 | 7 | 0 | 2 | 7 | 0 | 3 | 0 |
| OTU624 | 1 | 3 | 1 | 2 | 2 | 2 | 1 | 3 | 1 | 2 | 2 | 2 | 0 | 0 | 2 | 0 | 0 | 0 |
| OTU625 | 0 | 0 | 0 | 0 | 0 | 27 | 0 | 0 | 0 | 1 | 0 | 0 | 0 | 0 | 0 | 0 | 0 | 0 |
| OTU626 | 15 | 0 | 7 | 0 | 0 | 0 | 48 | 0 | 0 | 0 | 0 | 0 | 0 | 0 | 2 | 0 | 0 | 0 |
| OTU627 | 0 | 0 | 0 | 0 | 0 | 0 | 8 | 1 | 0 | 1 | 0 | 0 | 1 | 0 | 1 | 0 | 0 | 0 |
| OTU628 | 0 | 0 | 0 | 0 | 0 | 1 | 14 | 0 | 2 | 3 | 1 | 4 | 0 | 2 | 1 | 0 | 0 | 0 |
| OTU629 | 0 | 0 | 0 | 0 | 0 | 0 | 2 | 3 | 5 | 0 | 0 | 1 | 1 | 1 | 3 | 0 | 0 | 0 |
| OTU63 | 2 | 2 | 4 | 5 | 2 | 6 | 133 | 96 | 156 | 122 | 229 | 62 | 12 | 41 | 225 | 0 | 4 | 10 |
| OTU630 | 0 | 0 | 1 | 0 | 0 | 0 | 3 | 0 | 2 | 0 | 0 | 2 | 0 | 0 | 1 | 0 | 0 | 0 |
| OTU631 | 0 | 0 | 0 | 1 | 0 | 2 | 2 | 7 | 2 | 16 | 13 | 3 | 0 | 0 | 2 | 0 | 0 | 0 |
| OTU632 | 1 | 0 | 0 | 0 | 0 | 0 | 4 | 3 | 2 | 2 | 14 | 2 | 0 | 2 | 3 | 0 | 0 | 0 |
| OTU633 | 0 | 1 | 0 | 0 | 0 | 2 | 2 | 14 | 6 | 7 | 9 | 4 | 0 | 1 | 7 | 0 | 1 | 0 |
| OTU634 | 0 | 0 | 0 | 0 | 0 | 0 | 3 | 4 | 0 | 0 | 0 | 4 | 0 | 0 | 0 | 0 | 0 | 0 |
| OTU635 | 0 | 2 | 0 | 0 | 0 | 0 | 2 | 0 | 1 | 1 | 0 | 2 | 0 | 0 | 3 | 0 | 0 | 0 |
| OTU636 | 2 | 0 | 0 | 0 | 0 | 0 | 8 | 2 | 4 | 3 | 5 | 2 | 0 | 1 | 2 | 0 | 0 | 0 |
| OTU637 | 0 | 3 | 1 | 3 | 1 | 2 | 2 | 4 | 1 | 2 | 1 | 2 | 0 | 0 | 1 | 2 | 3 | 1 |
| OTU638 | 5 | 0 | 3 | 2 | 0 | 1 | 1 | 3 | 0 | 0 | 5 | 0 | 0 | 0 | 1 | 0 | 0 | 0 |
| OTU639 | 0 | 0 | 4 | 0 | 1 | 0 | 13 | 3 | 0 | 0 | 1 | 5 | 0 | 1 | 1 | 0 | 0 | 0 |
| OTU64 | 0 | 1 | 1 | 0 | 0 | 1 | 3 | 3 | 0 | 2 | 1 | 2 | 12 | 39 | 1 | 38 | 552 | 152 |
| OTU640 | 1 | 2 | 1 | 0 | 0 | 4 | 1 | 4 | 1 | 0 | 1 | 2 | 1 | 1 | 0 | 1 | 7 | 1 |
| OTU641 | 0 | 0 | 1 | 0 | 0 | 0 | 2 | 6 | 0 | 0 | 1 | 1 | 0 | 1 | 1 | 0 | 1 | 0 |
| OTU642 | 0 | 5 | 1 | 0 | 0 | 2 | 0 | 3 | 1 | 0 | 0 | 0 | 0 | 0 | 2 | 0 | 0 | 0 |
| OTU643 | 0 | 0 | 0 | 2 | 0 | 2 | 0 | 7 | 4 | 15 | 3 | 3 | 0 | 0 | 6 | 0 | 0 | 0 |
| OTU644 | 0 | 0 | 0 | 0 | 0 | 0 | 6 | 3 | 3 | 5 | 0 | 0 | 0 | 0 | 1 | 0 | 0 | 0 |
| OTU645 | 1 | 2 | 0 | 0 | 0 | 0 | 2 | 5 | 0 | 3 | 4 | 2 | 1 | 0 | 1 | 1 | 1 | 0 |
| OTU646 | 0 | 0 | 0 | 0 | 0 | 0 | 1 | 11 | 32 | 1 | 0 | 1 | 0 | 0 | 9 | 0 | 0 | 0 |
| OTU647 | 0 | 1 | 1 | 0 | 2 | 0 | 1 | 10 | 1 | 2 | 14 | 3 | 0 | 0 | 1 | 0 | 0 | 0 |
| OTU648 | 0 | 0 | 0 | 0 | 0 | 0 | 0 | 3 | 1 | 3 | 0 | 4 | 1 | 3 | 0 | 0 | 0 | 0 |
| OTU649 | 2 | 2 | 9 | 3 | 0 | 5 | 1 | 3 | 2 | 1 | 1 | 2 | 1 | 2 | 2 | 0 | 0 | 0 |
| OTU65 | 0 | 0 | 1 | 0 | 0 | 0 | 167 | 37 | 99 | 55 | 57 | 91 | 379 | 26 | 169 | 1 | 14 | 7 |
| OTU650 | 7 | 9 | 8 | 1 | 0 | 14 | 0 | 17 | 6 | 8 | 9 | 2 | 0 | 1 | 2 | 0 | 0 | 0 |
| OTU651 | 5 | 0 | 4 | 0 | 0 | 1 | 0 | 16 | 1 | 0 | 0 | 0 | 0 | 0 | 2 | 0 | 0 | 0 |
| OTU652 | 0 | 0 | 0 | 0 | 0 | 0 | 0 | 1 | 2 | 3 | 0 | 3 | 0 | 0 | 5 | 0 | 0 | 0 |
| OTU653 | 0 | 0 | 0 | 0 | 0 | 0 | 0 | 1 | 9 | 12 | 2 | 1 | 0 | 1 | 11 | 0 | 0 | 1 |
| OTU654 | 0 | 0 | 0 | 0 | 0 | 0 | 0 | 0 | 5 | 3 | 3 | 0 | 0 | 0 | 1 | 0 | 0 | 0 |
| OTU655 | 0 | 2 | 0 | 1 | 2 | 1 | 1 | 0 | 1 | 1 | 4 | 3 | 1 | 0 | 2 | 0 | 0 | 0 |
| OTU656 | 0 | 0 | 0 | 1 | 0 | 0 | 0 | 0 | 6 | 5 | 0 | 0 | 0 | 0 | 4 | 0 | 1 | 0 |
| OTU657 | 0 | 1 | 0 | 0 | 1 | 0 | 0 | 0 | 1 | 2 | 0 | 0 | 0 | 0 | 1 | 0 | 0 | 0 |
| OTU658 | 0 | 0 | 0 | 0 | 0 | 1 | 5 | 11 | 1 | 12 | 3 | 0 | 0 | 1 | 0 | 0 | 0 | 0 |
| OTU659 | 0 | 0 | 0 | 0 | 0 | 0 | 0 | 0 | 3 | 2 | 2 | 0 | 1 | 0 | 4 | 0 | 0 | 0 |
| OTU66 | 2 | 1015 | 160 | 6 | 77 | 26 | 5 | 10 | 10 | 36 | 45 | 22 | 1 | 3 | 14 | 0 | 1 | 1 |
| OTU660 | 0 | 0 | 0 | 0 | 0 | 0 | 1 | 1 | 1 | 1 | 2 | 15 | 0 | 0 | 1 | 0 | 0 | 0 |
| OTU661 | 0 | 0 | 0 | 0 | 0 | 0 | 0 | 0 | 0 | 2 | 17 | 2 | 0 | 0 | 0 | 0 | 0 | 0 |
| OTU662 | 0 | 0 | 0 | 0 | 0 | 1 | 3 | 1 | 1 | 2 | 24 | 1 | 1 | 0 | 0 | 0 | 0 | 0 |
| OTU663 | 0 | 0 | 0 | 0 | 0 | 0 | 0 | 3 | 6 | 1 | 4 | 0 | 0 | 1 | 5 | 0 | 0 | 0 |
| OTU664 | 11 | 5 | 11 | 0 | 2 | 1 | 1 | 0 | 1 | 0 | 0 | 4 | 0 | 2 | 0 | 0 | 0 | 0 |
| OTU665 | 0 | 0 | 0 | 0 | 0 | 0 | 0 | 0 | 0 | 0 | 0 | 0 | 1 | 7 | 0 | 0 | 1 | 6 |
| OTU666 | 1 | 0 | 0 | 0 | 0 | 0 | 0 | 1 | 2 | 1 | 1 | 0 | 1 | 0 | 0 | 4 | 0 | 0 |
| OTU667 | 1 | 1 | 3 | 1 | 1 | 1 | 2 | 6 | 3 | 1 | 2 | 0 | 2 | 5 | 2 | 2 | 2 | 0 |
| OTU668 | 0 | 0 | 0 | 0 | 0 | 0 | 0 | 0 | 0 | 0 | 0 | 0 | 2 | 4 | 0 | 0 | 3 | 6 |
| OTU669 | 0 | 0 | 0 | 0 | 0 | 0 | 0 | 0 | 0 | 0 | 0 | 0 | 0 | 6 | 0 | 0 | 9 | 14 |
| OTU67 | 1 | 157 | 70 | 1 | 176 | 302 | 0 | 1 | 0 | 0 | 0 | 0 | 0 | 0 | 0 | 0 | 1 | 0 |
| OTU670 | 0 | 1 | 1 | 0 | 0 | 0 | 0 | 0 | 1 | 0 | 2 | 0 | 4 | 3 | 0 | 0 | 13 | 8 |
| OTU671 | 0 | 0 | 0 | 0 | 0 | 0 | 0 | 0 | 0 | 0 | 0 | 0 | 1 | 0 | 0 | 0 | 2 | 3 |
| OTU672 | 0 | 0 | 0 | 0 | 0 | 0 | 0 | 0 | 0 | 0 | 0 | 0 | 0 | 0 | 0 | 0 | 19 | 5 |
| OTU673 | 0 | 0 | 0 | 0 | 0 | 0 | 0 | 0 | 0 | 0 | 0 | 1 | 3 | 0 | 0 | 0 | 17 | 11 |
| OTU674 | 0 | 0 | 0 | 0 | 1 | 1 | 0 | 0 | 0 | 0 | 1 | 0 | 0 | 0 | 0 | 0 | 15 | 0 |
| OTU675 | 0 | 0 | 0 | 0 | 0 | 0 | 0 | 0 | 0 | 0 | 0 | 0 | 0 | 0 | 0 | 0 | 5 | 2 |
| OTU676 | 0 | 0 | 0 | 0 | 0 | 0 | 0 | 0 | 0 | 0 | 0 | 0 | 0 | 0 | 0 | 0 | 24 | 0 |
| OTU677 | 0 | 0 | 0 | 0 | 0 | 0 | 1 | 0 | 0 | 0 | 0 | 0 | 1 | 0 | 0 | 1 | 11 | 4 |
| OTU678 | 3 | 0 | 0 | 0 | 0 | 0 | 0 | 0 | 0 | 0 | 0 | 0 | 0 | 0 | 0 | 0 | 41 | 0 |
| OTU679 | 0 | 1 | 0 | 0 | 0 | 0 | 0 | 0 | 0 | 0 | 0 | 0 | 0 | 0 | 0 | 0 | 27 | 0 |
| OTU68 | 0 | 0 | 0 | 0 | 0 | 0 | 2 | 2 | 2 | 2 | 1 | 5 | 52 | 126 | 4 | 38 | 387 | 270 |
| OTU680 | 0 | 0 | 0 | 0 | 0 | 0 | 0 | 0 | 0 | 0 | 0 | 0 | 0 | 0 | 0 | 0 | 52 | 0 |
| OTU681 | 0 | 0 | 0 | 0 | 0 | 0 | 0 | 1 | 0 | 0 | 0 | 0 | 0 | 0 | 0 | 0 | 26 | 0 |
| OTU682 | 0 | 0 | 0 | 0 | 0 | 0 | 0 | 0 | 0 | 0 | 0 | 0 | 2 | 2 | 0 | 2 | 22 | 8 |
| OTU683 | 0 | 1 | 1 | 2 | 0 | 1 | 1 | 0 | 0 | 1 | 0 | 1 | 2 | 1 | 0 | 0 | 23 | 2 |
| OTU684 | 7 | 0 | 0 | 0 | 0 | 0 | 0 | 11 | 2 | 6 | 15 | 0 | 0 | 0 | 0 | 0 | 0 | 0 |
| OTU685 | 2 | 0 | 1 | 0 | 0 | 2 | 0 | 0 | 0 | 0 | 0 | 0 | 0 | 0 | 0 | 0 | 0 | 0 |
| OTU686 | 3 | 3 | 11 | 0 | 0 | 1 | 0 | 0 | 0 | 0 | 0 | 0 | 0 | 0 | 0 | 0 | 0 | 0 |
| OTU687 | 2 | 0 | 0 | 0 | 0 | 0 | 0 | 0 | 0 | 0 | 0 | 0 | 0 | 0 | 0 | 0 | 0 | 0 |
| OTU688 | 2 | 0 | 1 | 0 | 0 | 0 | 0 | 0 | 4 | 0 | 0 | 0 | 0 | 0 | 1 | 0 | 2 | 0 |
| OTU689 | 13 | 0 | 0 | 0 | 0 | 0 | 0 | 0 | 0 | 0 | 0 | 0 | 0 | 0 | 0 | 0 | 0 | 0 |
| OTU69 | 35 | 564 | 56 | 12 | 176 | 135 | 77 | 1 | 101 | 11 | 8 | 3 | 0 | 2 | 170 | 0 | 3 | 1 |
| OTU690 | 1 | 1 | 1 | 0 | 1 | 2 | 1 | 0 | 2 | 4 | 1 | 0 | 1 | 1 | 0 | 0 | 0 | 0 |
| OTU691 | 76 | 112 | 13 | 0 | 0 | 1 | 16 | 3 | 4 | 0 | 3 | 89 | 8 | 18 | 7 | 0 | 0 | 0 |
| OTU692 | 2 | 0 | 0 | 0 | 0 | 0 | 0 | 0 | 0 | 0 | 0 | 0 | 0 | 0 | 0 | 0 | 0 | 0 |
| OTU693 | 1 | 1 | 2 | 0 | 2 | 1 | 0 | 1 | 0 | 0 | 1 | 0 | 1 | 0 | 0 | 0 | 0 | 1 |
| OTU694 | 2 | 1 | 2 | 0 | 3 | 3 | 0 | 0 | 0 | 0 | 0 | 0 | 0 | 0 | 0 | 0 | 0 | 0 |
| OTU695 | 2 | 0 | 0 | 0 | 3 | 8 | 0 | 1 | 0 | 1 | 2 | 2 | 0 | 0 | 0 | 0 | 0 | 0 |
| OTU696 | 1 | 0 | 1 | 0 | 0 | 1 | 4 | 4 | 1 | 4 | 2 | 0 | 0 | 0 | 1 | 0 | 0 | 0 |
| OTU697 | 5 | 3 | 1 | 1 | 3 | 2 | 1 | 6 | 1 | 0 | 1 | 1 | 0 | 0 | 0 | 0 | 0 | 0 |
| OTU698 | 4 | 0 | 4 | 0 | 0 | 0 | 0 | 1 | 0 | 1 | 0 | 0 | 0 | 0 | 1 | 0 | 0 | 0 |
| OTU699 | 4 | 0 | 3 | 3 | 0 | 1 | 0 | 1 | 0 | 0 | 0 | 0 | 0 | 0 | 0 | 0 | 0 | 0 |
| OTU7 | 4083 | 1198 | 3816 | 4193 | 2975 | 524 | 37 | 3 | 10 | 15 | 17 | 23 | 0 | 6 | 7 | 0 | 6 | 6 |
| OTU70 | 1 | 3 | 2 | 1 | 3 | 4 | 108 | 227 | 80 | 211 | 445 | 34 | 1 | 3 | 41 | 0 | 23 | 5 |
| OTU700 | 1 | 1 | 0 | 0 | 0 | 1 | 0 | 2 | 1 | 0 | 1 | 0 | 1 | 2 | 0 | 3 | 1 | 0 |
| OTU701 | 2 | 1 | 0 | 0 | 3 | 2 | 0 | 0 | 0 | 0 | 0 | 0 | 0 | 0 | 0 | 0 | 0 | 0 |
| OTU702 | 0 | 1 | 0 | 0 | 0 | 0 | 0 | 0 | 0 | 0 | 0 | 0 | 0 | 0 | 1 | 0 | 0 | 0 |
| OTU703 | 0 | 1 | 0 | 0 | 0 | 1 | 1 | 0 | 0 | 0 | 2 | 0 | 0 | 0 | 0 | 0 | 1 | 0 |
| OTU704 | 0 | 2 | 0 | 0 | 0 | 0 | 0 | 0 | 0 | 0 | 0 | 0 | 0 | 0 | 0 | 0 | 0 | 0 |
| OTU705 | 0 | 6 | 0 | 0 | 0 | 0 | 0 | 0 | 0 | 0 | 0 | 0 | 0 | 0 | 0 | 0 | 0 | 0 |
| OTU706 | 2 | 3 | 1 | 2 | 4 | 1 | 0 | 0 | 0 | 0 | 0 | 0 | 0 | 0 | 0 | 0 | 0 | 0 |
| OTU707 | 2 | 2 | 0 | 1 | 0 | 1 | 0 | 0 | 0 | 0 | 0 | 0 | 0 | 0 | 0 | 0 | 0 | 0 |
| OTU708 | 3 | 47 | 3 | 47 | 0 | 1 | 0 | 17 | 58 | 1 | 0 | 6 | 2 | 1 | 33 | 0 | 7 | 2 |
| OTU709 | 0 | 2 | 2 | 0 | 0 | 2 | 0 | 0 | 2 | 5 | 2 | 0 | 0 | 1 | 0 | 1 | 1 | 0 |
| OTU71 | 73 | 106 | 126 | 35 | 71 | 299 | 3 | 0 | 0 | 0 | 0 | 0 | 0 | 0 | 0 | 0 | 0 | 1 |
| OTU710 | 0 | 1 | 1 | 2 | 4 | 6 | 2 | 7 | 4 | 2 | 4 | 2 | 0 | 0 | 1 | 2 | 3 | 0 |
| OTU711 | 0 | 1 | 0 | 0 | 0 | 0 | 0 | 0 | 0 | 0 | 0 | 0 | 0 | 0 | 0 | 3 | 0 | 0 |
| OTU712 | 0 | 4 | 1 | 0 | 0 | 0 | 0 | 0 | 0 | 0 | 0 | 2 | 0 | 1 | 0 | 0 | 0 | 0 |
| OTU713 | 4 | 1 | 0 | 1 | 0 | 0 | 0 | 2 | 0 | 4 | 1 | 0 | 1 | 1 | 1 | 0 | 4 | 2 |
| OTU714 | 1 | 2 | 2 | 2 | 1 | 0 | 0 | 2 | 0 | 0 | 1 | 3 | 0 | 0 | 1 | 0 | 0 | 0 |
| OTU715 | 2 | 6 | 3 | 1 | 3 | 6 | 2 | 3 | 1 | 5 | 10 | 6 | 1 | 0 | 5 | 0 | 7 | 2 |
| OTU716 | 3 | 7 | 1 | 3 | 4 | 4 | 0 | 0 | 0 | 0 | 0 | 0 | 0 | 0 | 0 | 0 | 0 | 0 |
| OTU717 | 1 | 4 | 9 | 0 | 0 | 5 | 2 | 1 | 1 | 3 | 5 | 1 | 1 | 1 | 2 | 0 | 0 | 0 |
| OTU718 | 1 | 21 | 1 | 0 | 0 | 11 | 0 | 0 | 1 | 1 | 1 | 0 | 0 | 0 | 0 | 0 | 0 | 0 |
| OTU719 | 0 | 6 | 0 | 0 | 5 | 15 | 0 | 0 | 3 | 0 | 0 | 0 | 0 | 0 | 3 | 0 | 0 | 1 |
| OTU72 | 6 | 4 | 1 | 4 | 3 | 7 | 437 | 129 | 243 | 108 | 301 | 2 | 16 | 24 | 219 | 1 | 5 | 10 |
| OTU720 | 0 | 2 | 1 | 0 | 0 | 1 | 0 | 0 | 0 | 0 | 0 | 0 | 0 | 0 | 0 | 0 | 0 | 0 |
| OTU721 | 0 | 1 | 1 | 1 | 3 | 1 | 0 | 0 | 0 | 0 | 0 | 0 | 0 | 0 | 0 | 0 | 0 | 0 |
| OTU722 | 0 | 2 | 1 | 2 | 4 | 0 | 4 | 0 | 0 | 2 | 1 | 0 | 0 | 1 | 2 | 0 | 1 | 1 |
| OTU723 | 0 | 7 | 0 | 1 | 0 | 0 | 0 | 0 | 0 | 0 | 0 | 0 | 0 | 0 | 0 | 0 | 0 | 0 |
| OTU724 | 0 | 2 | 7 | 7 | 8 | 6 | 0 | 0 | 0 | 0 | 0 | 0 | 0 | 0 | 0 | 0 | 0 | 0 |
| OTU725 | 4 | 0 | 13 | 0 | 0 | 0 | 0 | 0 | 1 | 0 | 0 | 1 | 0 | 0 | 2 | 0 | 0 | 0 |
| OTU726 | 0 | 1 | 12 | 0 | 8 | 1 | 0 | 0 | 0 | 0 | 0 | 0 | 0 | 0 | 0 | 1 | 1 | 1 |
| OTU727 | 1 | 0 | 5 | 3 | 3 | 0 | 1 | 2 | 0 | 3 | 1 | 1 | 0 | 1 | 0 | 0 | 1 | 1 |
| OTU728 | 0 | 0 | 2 | 0 | 0 | 0 | 1 | 1 | 0 | 0 | 0 | 9 | 0 | 3 | 0 | 0 | 0 | 0 |
| OTU729 | 0 | 0 | 3 | 1 | 2 | 2 | 1 | 0 | 0 | 1 | 2 | 0 | 0 | 0 | 0 | 0 | 0 | 0 |
| OTU73 | 6 | 36 | 65 | 4 | 306 | 17 | 2 | 194 | 449 | 4 | 170 | 138 | 1 | 3 | 462 | 0 | 23 | 10 |
| OTU730 | 0 | 0 | 4 | 2 | 14 | 0 | 2 | 0 | 0 | 0 | 0 | 0 | 0 | 0 | 0 | 0 | 0 | 0 |
| OTU731 | 0 | 0 | 14 | 0 | 3 | 0 | 0 | 4 | 5 | 1 | 2 | 0 | 1 | 0 | 2 | 0 | 1 | 0 |
| OTU732 | 5 | 3 | 5 | 3 | 4 | 0 | 0 | 1 | 1 | 1 | 1 | 4 | 1 | 3 | 2 | 0 | 0 | 0 |
| OTU733 | 0 | 0 | 2 | 0 | 0 | 0 | 0 | 0 | 0 | 0 | 0 | 0 | 0 | 0 | 0 | 0 | 0 | 0 |
| OTU734 | 0 | 6 | 7 | 0 | 0 | 0 | 0 | 0 | 1 | 1 | 0 | 0 | 0 | 0 | 0 | 0 | 0 | 0 |
| OTU735 | 2 | 0 | 1 | 4 | 1 | 4 | 4 | 0 | 1 | 0 | 0 | 5 | 1 | 6 | 1 | 0 | 0 | 1 |
| OTU736 | 1 | 1 | 5 | 1 | 0 | 0 | 0 | 0 | 4 | 0 | 0 | 1 | 0 | 0 | 1 | 0 | 1 | 0 |
| OTU737 | 0 | 0 | 3 | 0 | 0 | 3 | 0 | 0 | 0 | 0 | 0 | 0 | 0 | 0 | 0 | 0 | 0 | 0 |
| OTU738 | 24 | 11 | 32 | 42 | 27 | 45 | 2 | 0 | 1 | 1 | 0 | 1 | 0 | 0 | 0 | 0 | 0 | 0 |
| OTU739 | 0 | 0 | 1 | 0 | 1 | 0 | 0 | 0 | 0 | 0 | 0 | 0 | 0 | 0 | 1 | 0 | 0 | 0 |
| OTU74 | 65 | 20 | 638 | 0 | 3 | 25 | 3 | 0 | 0 | 0 | 0 | 0 | 0 | 0 | 0 | 0 | 0 | 0 |
| OTU740 | 1 | 0 | 3 | 3 | 0 | 2 | 0 | 0 | 0 | 0 | 0 | 0 | 0 | 0 | 0 | 0 | 0 | 0 |
| OTU741 | 0 | 0 | 1 | 0 | 2 | 0 | 0 | 0 | 0 | 0 | 0 | 0 | 0 | 0 | 0 | 0 | 0 | 0 |
| OTU742 | 3 | 2 | 8 | 7 | 3 | 8 | 0 | 0 | 0 | 0 | 0 | 0 | 0 | 0 | 0 | 0 | 0 | 0 |
| OTU743 | 1 | 0 | 2 | 0 | 0 | 0 | 1 | 5 | 1 | 1 | 8 | 3 | 1 | 2 | 2 | 0 | 0 | 1 |
| OTU744 | 1 | 0 | 7 | 0 | 0 | 2 | 0 | 0 | 0 | 0 | 0 | 0 | 0 | 0 | 0 | 0 | 0 | 0 |
| OTU745 | 0 | 0 | 1 | 4 | 1 | 1 | 0 | 0 | 0 | 1 | 0 | 1 | 0 | 0 | 0 | 0 | 0 | 0 |
| OTU746 | 7 | 2 | 6 | 6 | 17 | 16 | 3 | 38 | 13 | 23 | 29 | 17 | 1 | 1 | 11 | 0 | 0 | 0 |
| OTU747 | 0 | 0 | 1 | 7 | 2 | 1 | 0 | 0 | 0 | 0 | 0 | 0 | 0 | 0 | 0 | 0 | 0 | 0 |
| OTU748 | 15 | 29 | 66 | 58 | 51 | 41 | 2 | 0 | 0 | 17 | 18 | 0 | 0 | 1 | 0 | 0 | 0 | 1 |
| OTU749 | 3 | 1 | 4 | 3 | 0 | 8 | 0 | 0 | 0 | 0 | 0 | 0 | 0 | 0 | 0 | 0 | 0 | 0 |
| OTU75 | 7 | 2 | 11 | 0 | 1 | 2 | 4 | 0 | 1 | 1 | 2 | 2 | 2 | 4 | 2 | 22 | 639 | 300 |
| OTU750 | 1 | 2 | 6 | 6 | 3 | 7 | 0 | 0 | 0 | 0 | 0 | 0 | 0 | 0 | 0 | 0 | 0 | 0 |
| OTU751 | 0 | 0 | 0 | 1 | 0 | 0 | 0 | 1 | 0 | 2 | 0 | 2 | 0 | 1 | 0 | 0 | 0 | 0 |
| OTU752 | 1 | 5 | 5 | 1 | 0 | 0 | 1 | 0 | 0 | 1 | 0 | 0 | 0 | 0 | 1 | 0 | 0 | 0 |
| OTU753 | 0 | 0 | 1 | 0 | 8 | 12 | 0 | 0 | 0 | 0 | 0 | 0 | 0 | 0 | 0 | 0 | 0 | 0 |
| OTU754 | 1 | 0 | 0 | 0 | 3 | 0 | 1 | 0 | 0 | 0 | 0 | 0 | 0 | 0 | 0 | 0 | 0 | 0 |
| OTU755 | 0 | 0 | 0 | 2 | 3 | 16 | 0 | 1 | 0 | 1 | 2 | 0 | 0 | 0 | 0 | 0 | 0 | 0 |
| OTU756 | 0 | 1 | 2 | 0 | 2 | 2 | 5 | 0 | 1 | 0 | 0 | 3 | 0 | 0 | 1 | 0 | 6 | 1 |
| OTU757 | 0 | 0 | 0 | 0 | 13 | 2 | 0 | 0 | 0 | 0 | 0 | 0 | 0 | 0 | 0 | 0 | 0 | 0 |
| OTU758 | 0 | 0 | 2 | 0 | 1 | 4 | 0 | 0 | 0 | 0 | 0 | 0 | 0 | 0 | 0 | 0 | 0 | 0 |
| OTU759 | 0 | 0 | 4 | 1 | 11 | 2 | 0 | 0 | 0 | 0 | 0 | 0 | 0 | 0 | 0 | 0 | 0 | 0 |
| OTU76 | 0 | 0 | 0 | 0 | 0 | 0 | 141 | 138 | 84 | 23 | 121 | 148 | 18 | 80 | 66 | 1 | 3 | 4 |
| OTU760 | 1 | 0 | 0 | 0 | 4 | 1 | 0 | 0 | 0 | 0 | 0 | 0 | 0 | 0 | 0 | 0 | 0 | 0 |
| OTU761 | 0 | 0 | 2 | 0 | 1 | 1 | 3 | 4 | 0 | 2 | 3 | 3 | 1 | 1 | 2 | 3 | 4 | 1 |
| OTU762 | 0 | 0 | 0 | 0 | 2 | 0 | 0 | 0 | 0 | 2 | 0 | 0 | 0 | 0 | 0 | 0 | 0 | 0 |
| OTU763 | 0 | 0 | 1 | 1 | 2 | 0 | 0 | 0 | 3 | 0 | 2 | 2 | 0 | 0 | 0 | 0 | 1 | 1 |
| OTU764 | 2 | 0 | 0 | 0 | 3 | 6 | 0 | 0 | 0 | 0 | 0 | 0 | 0 | 0 | 0 | 0 | 0 | 0 |
| OTU765 | 1 | 0 | 1 | 0 | 24 | 0 | 0 | 0 | 0 | 0 | 0 | 0 | 0 | 0 | 0 | 0 | 0 | 0 |
| OTU766 | 4 | 21 | 0 | 12 | 17 | 1 | 0 | 32 | 6 | 29 | 24 | 6 | 2 | 6 | 6 | 0 | 0 | 3 |
| OTU767 | 2 | 2 | 1 | 1 | 2 | 2 | 2 | 3 | 1 | 1 | 2 | 2 | 8 | 2 | 0 | 1 | 7 | 2 |
| OTU768 | 1 | 0 | 0 | 1 | 1 | 0 | 1 | 1 | 1 | 1 | 0 | 0 | 0 | 0 | 2 | 0 | 1 | 0 |
| OTU769 | 0 | 0 | 0 | 0 | 1 | 0 | 0 | 0 | 0 | 0 | 0 | 0 | 0 | 1 | 0 | 0 | 0 | 1 |
| OTU77 | 150 | 198 | 263 | 30 | 284 | 79 | 9 | 3 | 2 | 1 | 3 | 17 | 1 | 2 | 10 | 0 | 1 | 1 |
| OTU770 | 1 | 0 | 0 | 0 | 1 | 1 | 1 | 0 | 0 | 6 | 4 | 1 | 0 | 0 | 3 | 0 | 1 | 0 |
| OTU771 | 0 | 0 | 1 | 0 | 3 | 2 | 0 | 0 | 0 | 0 | 0 | 0 | 0 | 0 | 0 | 0 | 0 | 0 |
| OTU772 | 0 | 0 | 0 | 0 | 1 | 0 | 0 | 0 | 0 | 0 | 0 | 0 | 0 | 2 | 0 | 0 | 0 | 0 |
| OTU773 | 0 | 1 | 1 | 0 | 1 | 2 | 0 | 0 | 0 | 0 | 1 | 0 | 1 | 1 | 1 | 0 | 1 | 0 |
| OTU774 | 1 | 1 | 1 | 1 | 1 | 0 | 0 | 1 | 2 | 0 | 0 | 0 | 2 | 0 | 1 | 0 | 0 | 0 |
| OTU775 | 1 | 1 | 0 | 0 | 0 | 5 | 0 | 0 | 0 | 0 | 0 | 0 | 0 | 0 | 0 | 0 | 0 | 0 |
| OTU776 | 1 | 0 | 0 | 0 | 0 | 6 | 0 | 3 | 0 | 0 | 1 | 0 | 0 | 0 | 0 | 0 | 0 | 0 |
| OTU777 | 1 | 0 | 0 | 0 | 0 | 2 | 0 | 0 | 0 | 0 | 0 | 0 | 0 | 0 | 0 | 0 | 0 | 0 |
| OTU778 | 0 | 1 | 1 | 0 | 2 | 3 | 0 | 0 | 0 | 0 | 0 | 0 | 0 | 0 | 0 | 0 | 0 | 0 |
| OTU779 | 0 | 0 | 0 | 0 | 0 | 10 | 0 | 3 | 1 | 1 | 2 | 0 | 0 | 0 | 0 | 0 | 1 | 0 |
| OTU78 | 92 | 296 | 277 | 121 | 69 | 173 | 5 | 0 | 1 | 10 | 4 | 2 | 0 | 1 | 2 | 0 | 0 | 2 |
| OTU780 | 0 | 3 | 0 | 0 | 2 | 19 | 0 | 0 | 0 | 0 | 0 | 0 | 0 | 0 | 0 | 0 | 0 | 0 |
| OTU781 | 0 | 0 | 1 | 1 | 0 | 8 | 0 | 0 | 2 | 1 | 0 | 0 | 0 | 0 | 0 | 0 | 0 | 0 |
| OTU782 | 5 | 1 | 3 | 1 | 17 | 8 | 2 | 24 | 12 | 14 | 15 | 7 | 0 | 1 | 7 | 0 | 0 | 0 |
| OTU783 | 0 | 3 | 0 | 1 | 0 | 1 | 0 | 0 | 1 | 2 | 0 | 0 | 0 | 0 | 0 | 0 | 0 | 0 |
| OTU784 | 0 | 1 | 0 | 0 | 0 | 1 | 0 | 0 | 1 | 0 | 2 | 2 | 0 | 1 | 0 | 0 | 0 | 0 |
| OTU785 | 0 | 0 | 1 | 2 | 0 | 2 | 1 | 0 | 0 | 1 | 4 | 1 | 0 | 1 | 1 | 0 | 0 | 0 |
| OTU786 | 2 | 0 | 0 | 0 | 1 | 2 | 0 | 1 | 2 | 0 | 3 | 1 | 0 | 0 | 1 | 0 | 2 | 0 |
| OTU787 | 0 | 0 | 0 | 0 | 0 | 1 | 0 | 0 | 0 | 2 | 2 | 1 | 0 | 0 | 1 | 0 | 0 | 0 |
| OTU788 | 0 | 0 | 0 | 0 | 0 | 1 | 0 | 0 | 0 | 1 | 0 | 0 | 0 | 0 | 0 | 0 | 0 | 0 |
| OTU789 | 0 | 0 | 1 | 1 | 1 | 4 | 3 | 3 | 0 | 1 | 2 | 0 | 2 | 0 | 0 | 0 | 0 | 0 |
| OTU79 | 112 | 72 | 293 | 163 | 553 | 161 | 31 | 17 | 18 | 53 | 12 | 18 | 8 | 29 | 11 | 0 | 2 | 1 |
| OTU790 | 0 | 0 | 0 | 0 | 0 | 0 | 7 | 2 | 3 | 2 | 1 | 2 | 0 | 0 | 8 | 0 | 0 | 0 |
| OTU791 | 1 | 0 | 0 | 0 | 0 | 0 | 86 | 0 | 0 | 44 | 13 | 0 | 0 | 0 | 0 | 0 | 2 | 3 |
| OTU792 | 0 | 0 | 0 | 0 | 0 | 1 | 3 | 1 | 0 | 0 | 0 | 1 | 0 | 0 | 0 | 0 | 0 | 1 |
| OTU793 | 0 | 0 | 0 | 0 | 1 | 0 | 1 | 0 | 0 | 0 | 0 | 2 | 0 | 0 | 0 | 0 | 0 | 0 |
| OTU794 | 6 | 0 | 1 | 0 | 0 | 0 | 4 | 8 | 1 | 0 | 0 | 2 | 0 | 1 | 0 | 0 | 0 | 0 |
| OTU795 | 2 | 1 | 2 | 2 | 1 | 2 | 5 | 5 | 3 | 7 | 3 | 2 | 11 | 6 | 1 | 11 | 4 | 5 |
| OTU796 | 0 | 0 | 0 | 0 | 0 | 0 | 2 | 4 | 0 | 9 | 8 | 3 | 1 | 2 | 1 | 0 | 0 | 0 |
| OTU797 | 0 | 0 | 2 | 0 | 0 | 0 | 3 | 0 | 3 | 3 | 1 | 0 | 0 | 0 | 2 | 0 | 2 | 0 |
| OTU798 | 0 | 0 | 0 | 0 | 0 | 0 | 1 | 2 | 1 | 3 | 0 | 1 | 0 | 2 | 2 | 0 | 0 | 0 |
| OTU799 | 0 | 0 | 0 | 0 | 0 | 0 | 4 | 1 | 0 | 0 | 0 | 0 | 0 | 0 | 0 | 0 | 0 | 0 |
| OTU8 | 1737 | 42 | 416 | 325 | 379 | 66 | 6336 | 313 | 109 | 1460 | 770 | 2766 | 70 | 358 | 255 | 0 | 9 | 43 |
| OTU80 | 0 | 26 | 3 | 1 | 0 | 719 | 2 | 19 | 18 | 50 | 24 | 51 | 2 | 8 | 11 | 0 | 2 | 3 |
| OTU800 | 0 | 0 | 0 | 0 | 0 | 0 | 17 | 0 | 0 | 0 | 5 | 0 | 0 | 0 | 1 | 0 | 0 | 0 |
| OTU801 | 2 | 1 | 1 | 0 | 1 | 2 | 6 | 3 | 2 | 6 | 9 | 3 | 0 | 1 | 5 | 0 | 0 | 0 |
| OTU802 | 0 | 0 | 0 | 0 | 0 | 0 | 1 | 0 | 1 | 0 | 0 | 0 | 0 | 0 | 0 | 0 | 0 | 0 |
| OTU803 | 0 | 0 | 0 | 0 | 0 | 0 | 4 | 3 | 1 | 9 | 7 | 2 | 3 | 0 | 8 | 0 | 0 | 0 |
| OTU804 | 0 | 0 | 0 | 0 | 0 | 0 | 5 | 0 | 0 | 1 | 1 | 2 | 0 | 0 | 1 | 0 | 3 | 0 |
| OTU805 | 0 | 0 | 0 | 0 | 0 | 0 | 3 | 0 | 1 | 0 | 1 | 6 | 0 | 1 | 0 | 0 | 0 | 0 |
| OTU806 | 0 | 0 | 0 | 0 | 0 | 0 | 0 | 1 | 1 | 1 | 1 | 0 | 0 | 0 | 0 | 0 | 6 | 0 |
| OTU807 | 0 | 0 | 0 | 1 | 1 | 0 | 2 | 1 | 1 | 1 | 1 | 1 | 1 | 0 | 2 | 1 | 0 | 0 |
| OTU808 | 0 | 0 | 0 | 0 | 0 | 0 | 0 | 4 | 1 | 1 | 0 | 0 | 0 | 0 | 0 | 0 | 0 | 0 |
| OTU809 | 0 | 0 | 0 | 0 | 0 | 0 | 0 | 6 | 2 | 3 | 0 | 2 | 0 | 0 | 0 | 0 | 0 | 0 |
| OTU81 | 70 | 223 | 83 | 5 | 1 | 282 | 60 | 10 | 25 | 4 | 6 | 191 | 9 | 69 | 38 | 1 | 6 | 0 |
| OTU810 | 0 | 1 | 0 | 0 | 0 | 0 | 0 | 2 | 0 | 0 | 0 | 1 | 1 | 0 | 0 | 1 | 0 | 0 |
| OTU811 | 0 | 0 | 0 | 0 | 0 | 0 | 0 | 1 | 0 | 0 | 0 | 2 | 0 | 0 | 0 | 0 | 0 | 0 |
| OTU812 | 0 | 0 | 0 | 0 | 0 | 0 | 0 | 2 | 0 | 0 | 0 | 0 | 0 | 0 | 0 | 0 | 0 | 0 |
| OTU813 | 0 | 0 | 0 | 0 | 0 | 0 | 0 | 1 | 2 | 5 | 1 | 2 | 0 | 0 | 0 | 0 | 0 | 0 |
| OTU814 | 0 | 1 | 2 | 0 | 0 | 0 | 1 | 8 | 0 | 0 | 15 | 1 | 0 | 0 | 2 | 0 | 1 | 1 |
| OTU815 | 0 | 0 | 0 | 0 | 0 | 0 | 0 | 1 | 1 | 1 | 1 | 2 | 0 | 0 | 1 | 0 | 1 | 0 |
| OTU816 | 0 | 0 | 0 | 0 | 0 | 0 | 2 | 7 | 1 | 3 | 0 | 0 | 0 | 1 | 2 | 0 | 0 | 0 |
| OTU817 | 0 | 0 | 0 | 0 | 0 | 0 | 0 | 2 | 0 | 0 | 0 | 0 | 0 | 0 | 0 | 0 | 0 | 0 |
| OTU818 | 0 | 0 | 0 | 0 | 0 | 0 | 0 | 3 | 0 | 0 | 14 | 1 | 1 | 0 | 0 | 0 | 0 | 0 |
| OTU819 | 0 | 0 | 0 | 0 | 0 | 0 | 0 | 3 | 6 | 2 | 0 | 1 | 0 | 0 | 9 | 0 | 0 | 0 |
| OTU82 | 117 | 80 | 183 | 0 | 41 | 104 | 26 | 3 | 1 | 0 | 2 | 19 | 6 | 14 | 0 | 0 | 1 | 1 |
| OTU820 | 0 | 0 | 0 | 0 | 0 | 1 | 0 | 1 | 0 | 1 | 1 | 1 | 0 | 0 | 0 | 0 | 2 | 0 |
| OTU821 | 0 | 0 | 0 | 0 | 0 | 0 | 0 | 3 | 2 | 0 | 1 | 0 | 0 | 0 | 0 | 0 | 0 | 0 |
| OTU822 | 0 | 0 | 0 | 0 | 0 | 1 | 1 | 2 | 1 | 3 | 4 | 1 | 0 | 2 | 3 | 0 | 0 | 0 |
| OTU823 | 2 | 1 | 3 | 0 | 0 | 6 | 20 | 3 | 3 | 4 | 3 | 7 | 1 | 2 | 3 | 0 | 1 | 0 |
| OTU824 | 0 | 1 | 1 | 0 | 1 | 0 | 1 | 2 | 0 | 1 | 1 | 2 | 0 | 0 | 0 | 0 | 0 | 0 |
| OTU825 | 0 | 0 | 0 | 0 | 0 | 0 | 0 | 4 | 0 | 2 | 3 | 5 | 0 | 0 | 1 | 0 | 1 | 0 |
| OTU826 | 1 | 0 | 0 | 0 | 0 | 0 | 0 | 1 | 0 | 0 | 1 | 0 | 2 | 0 | 1 | 0 | 1 | 1 |
| OTU827 | 1 | 0 | 9 | 3 | 0 | 4 | 12 | 136 | 38 | 42 | 82 | 58 | 2 | 5 | 20 | 0 | 0 | 0 |
| OTU828 | 0 | 2 | 0 | 0 | 0 | 0 | 1 | 3 | 9 | 0 | 0 | 1 | 0 | 0 | 3 | 0 | 2 | 0 |
| OTU829 | 1 | 3 | 6 | 2 | 5 | 46 | 7 | 95 | 20 | 105 | 72 | 65 | 1 | 2 | 20 | 0 | 1 | 2 |
| OTU83 | 14 | 10 | 3 | 13 | 11 | 14 | 474 | 83 | 159 | 128 | 608 | 382 | 18 | 83 | 70 | 0 | 1 | 2 |
| OTU830 | 2 | 0 | 1 | 0 | 0 | 0 | 0 | 3 | 3 | 0 | 0 | 9 | 0 | 0 | 4 | 0 | 0 | 0 |
| OTU831 | 0 | 0 | 0 | 0 | 0 | 2 | 0 | 4 | 2 | 9 | 9 | 1 | 0 | 0 | 1 | 0 | 1 | 0 |
| OTU832 | 0 | 0 | 0 | 0 | 0 | 0 | 0 | 0 | 1 | 1 | 0 | 1 | 0 | 0 | 0 | 0 | 0 | 0 |
| OTU833 | 0 | 1 | 1 | 2 | 0 | 0 | 0 | 0 | 4 | 1 | 0 | 2 | 0 | 0 | 0 | 0 | 0 | 0 |
| OTU834 | 0 | 0 | 2 | 0 | 0 | 0 | 2 | 0 | 27 | 1 | 0 | 20 | 0 | 2 | 20 | 0 | 0 | 0 |
| OTU835 | 0 | 0 | 0 | 0 | 0 | 1 | 0 | 0 | 5 | 1 | 0 | 0 | 0 | 1 | 3 | 0 | 0 | 0 |
| OTU836 | 0 | 0 | 0 | 0 | 0 | 0 | 0 | 0 | 9 | 4 | 0 | 0 | 0 | 0 | 2 | 0 | 0 | 0 |
| OTU837 | 0 | 0 | 0 | 0 | 1 | 3 | 0 | 1 | 2 | 2 | 0 | 1 | 1 | 0 | 1 | 0 | 1 | 0 |
| OTU838 | 3 | 3 | 1 | 2 | 0 | 2 | 4 | 20 | 77 | 109 | 121 | 46 | 2 | 9 | 87 | 1 | 7 | 5 |
| OTU839 | 0 | 1 | 1 | 1 | 1 | 2 | 0 | 20 | 14 | 0 | 3 | 6 | 1 | 0 | 22 | 0 | 0 | 0 |
| OTU84 | 12 | 3 | 30 | 0 | 4 | 4 | 20 | 48 | 140 | 38 | 307 | 132 | 22 | 70 | 153 | 0 | 8 | 3 |
| OTU840 | 1 | 0 | 1 | 1 | 0 | 1 | 1 | 1 | 3 | 0 | 2 | 2 | 0 | 1 | 0 | 0 | 2 | 0 |
| OTU841 | 0 | 0 | 0 | 0 | 0 | 0 | 1 | 0 | 6 | 0 | 0 | 2 | 1 | 1 | 3 | 1 | 0 | 1 |
| OTU842 | 1 | 0 | 0 | 1 | 1 | 0 | 0 | 3 | 1 | 0 | 0 | 0 | 0 | 5 | 1 | 1 | 2 | 2 |
| OTU843 | 1 | 2 | 2 | 0 | 3 | 5 | 2 | 3 | 2 | 5 | 2 | 3 | 1 | 1 | 2 | 1 | 1 | 0 |
| OTU844 | 0 | 0 | 0 | 1 | 1 | 0 | 0 | 10 | 4 | 11 | 1 | 0 | 0 | 0 | 3 | 0 | 1 | 0 |
| OTU845 | 0 | 0 | 0 | 0 | 0 | 0 | 0 | 1 | 2 | 0 | 0 | 1 | 0 | 0 | 0 | 0 | 1 | 0 |
| OTU846 | 0 | 0 | 0 | 0 | 0 | 0 | 0 | 4 | 7 | 3 | 2 | 1 | 0 | 1 | 3 | 0 | 0 | 0 |
| OTU847 | 0 | 0 | 0 | 2 | 0 | 0 | 1 | 0 | 7 | 0 | 0 | 0 | 0 | 0 | 6 | 0 | 0 | 0 |
| OTU848 | 1 | 0 | 0 | 0 | 1 | 2 | 0 | 0 | 3 | 2 | 1 | 0 | 0 | 0 | 2 | 0 | 0 | 0 |
| OTU849 | 0 | 0 | 0 | 0 | 0 | 0 | 4 | 0 | 0 | 4 | 2 | 0 | 0 | 0 | 0 | 0 | 0 | 0 |
| OTU85 | 0 | 2 | 6 | 0 | 1 | 8 | 66 | 263 | 75 | 35 | 11 | 202 | 5 | 7 | 54 | 0 | 5 | 4 |
| OTU850 | 3 | 4 | 2 | 10 | 4 | 12 | 34 | 22 | 43 | 135 | 12 | 6 | 1 | 1 | 18 | 0 | 0 | 1 |
| OTU851 | 0 | 0 | 0 | 0 | 0 | 0 | 0 | 2 | 1 | 3 | 0 | 3 | 0 | 0 | 0 | 0 | 0 | 0 |
| OTU852 | 1 | 0 | 3 | 0 | 2 | 3 | 73 | 69 | 52 | 88 | 54 | 45 | 11 | 26 | 18 | 0 | 7 | 4 |
| OTU853 | 0 | 0 | 0 | 0 | 0 | 0 | 0 | 2 | 0 | 1 | 1 | 0 | 0 | 1 | 0 | 0 | 0 | 0 |
| OTU854 | 0 | 0 | 0 | 0 | 0 | 0 | 0 | 4 | 0 | 9 | 1 | 0 | 0 | 0 | 0 | 0 | 0 | 0 |
| OTU855 | 0 | 0 | 0 | 0 | 2 | 2 | 1 | 2 | 0 | 1 | 1 | 1 | 0 | 0 | 1 | 0 | 0 | 0 |
| OTU856 | 0 | 0 | 0 | 0 | 1 | 0 | 8 | 38 | 3 | 42 | 8 | 1 | 0 | 0 | 2 | 0 | 3 | 1 |
| OTU857 | 0 | 0 | 0 | 0 | 0 | 0 | 0 | 0 | 0 | 2 | 1 | 2 | 1 | 0 | 1 | 0 | 0 | 0 |
| OTU858 | 0 | 0 | 0 | 0 | 0 | 0 | 1 | 3 | 2 | 1 | 3 | 2 | 0 | 0 | 2 | 0 | 0 | 0 |
| OTU859 | 0 | 0 | 0 | 0 | 0 | 0 | 0 | 0 | 0 | 1 | 0 | 1 | 0 | 0 | 0 | 0 | 0 | 0 |
| OTU86 | 0 | 1 | 0 | 0 | 1 | 0 | 17 | 87 | 187 | 37 | 45 | 356 | 16 | 18 | 138 | 0 | 6 | 5 |
| OTU860 | 0 | 0 | 1 | 0 | 0 | 0 | 1 | 2 | 2 | 3 | 1 | 0 | 0 | 2 | 0 | 0 | 0 | 1 |
| OTU861 | 1 | 2 | 3 | 0 | 8 | 11 | 1 | 4 | 8 | 18 | 4 | 6 | 0 | 11 | 2 | 0 | 0 | 3 |
| OTU862 | 0 | 0 | 1 | 0 | 0 | 0 | 1 | 0 | 2 | 3 | 0 | 2 | 0 | 1 | 2 | 0 | 0 | 0 |
| OTU863 | 0 | 0 | 0 | 0 | 0 | 0 | 0 | 0 | 0 | 1 | 2 | 1 | 0 | 0 | 1 | 0 | 0 | 0 |
| OTU864 | 0 | 0 | 0 | 0 | 0 | 0 | 1 | 1 | 1 | 1 | 3 | 2 | 0 | 2 | 0 | 0 | 0 | 0 |
| OTU865 | 0 | 0 | 8 | 0 | 0 | 25 | 3 | 6 | 1 | 34 | 104 | 2 | 0 | 0 | 5 | 0 | 1 | 1 |
| OTU866 | 0 | 0 | 0 | 0 | 0 | 0 | 0 | 0 | 0 | 1 | 2 | 0 | 0 | 0 | 4 | 0 | 0 | 0 |
| OTU867 | 0 | 0 | 0 | 0 | 1 | 1 | 1 | 0 | 3 | 0 | 8 | 1 | 0 | 2 | 0 | 0 | 0 | 1 |
| OTU868 | 0 | 0 | 0 | 0 | 0 | 0 | 0 | 0 | 0 | 0 | 1 | 3 | 1 | 1 | 0 | 0 | 0 | 0 |
| OTU869 | 0 | 0 | 0 | 0 | 1 | 1 | 0 | 1 | 0 | 0 | 1 | 0 | 2 | 0 | 0 | 0 | 0 | 1 |
| OTU87 | 7 | 3 | 8 | 11 | 2 | 11 | 1 | 19 | 2 | 2 | 8 | 359 | 32 | 134 | 0 | 0 | 0 | 0 |
| OTU870 | 0 | 0 | 1 | 1 | 0 | 0 | 1 | 0 | 1 | 0 | 2 | 3 | 1 | 1 | 1 | 0 | 0 | 0 |
| OTU871 | 1 | 0 | 0 | 1 | 1 | 0 | 0 | 0 | 0 | 0 | 0 | 2 | 0 | 0 | 1 | 0 | 0 | 2 |
| OTU872 | 0 | 0 | 0 | 0 | 0 | 0 | 2 | 0 | 1 | 0 | 1 | 4 | 0 | 0 | 0 | 0 | 0 | 0 |
| OTU873 | 0 | 1 | 0 | 0 | 0 | 1 | 1 | 1 | 1 | 0 | 0 | 1 | 0 | 1 | 1 | 1 | 0 | 0 |
| OTU874 | 0 | 1 | 2 | 0 | 2 | 0 | 0 | 1 | 0 | 0 | 1 | 3 | 0 | 2 | 1 | 0 | 0 | 0 |
| OTU875 | 0 | 0 | 0 | 0 | 0 | 0 | 0 | 0 | 0 | 0 | 0 | 4 | 0 | 0 | 1 | 0 | 0 | 0 |
| OTU876 | 0 | 0 | 0 | 0 | 0 | 0 | 0 | 3 | 5 | 0 | 0 | 1 | 1 | 2 | 4 | 0 | 1 | 0 |
| OTU877 | 1 | 0 | 1 | 0 | 0 | 1 | 0 | 0 | 0 | 2 | 1 | 3 | 1 | 1 | 0 | 1 | 0 | 0 |
| OTU878 | 0 | 1 | 0 | 0 | 0 | 0 | 1 | 0 | 0 | 1 | 0 | 1 | 2 | 0 | 0 | 0 | 0 | 0 |
| OTU879 | 0 | 0 | 0 | 0 | 0 | 0 | 0 | 0 | 0 | 0 | 0 | 0 | 1 | 0 | 1 | 0 | 0 | 0 |
| OTU88 | 103 | 24 | 39 | 302 | 127 | 4 | 0 | 0 | 0 | 0 | 0 | 0 | 0 | 0 | 0 | 0 | 0 | 1 |
| OTU880 | 0 | 0 | 0 | 0 | 0 | 0 | 0 | 0 | 0 | 0 | 0 | 0 | 2 | 1 | 0 | 0 | 3 | 1 |
| OTU881 | 0 | 0 | 0 | 0 | 0 | 0 | 0 | 0 | 0 | 0 | 0 | 0 | 1 | 0 | 0 | 0 | 2 | 2 |
| OTU882 | 0 | 0 | 0 | 0 | 0 | 0 | 0 | 0 | 0 | 0 | 0 | 0 | 0 | 1 | 0 | 0 | 18 | 5 |
| OTU883 | 0 | 0 | 0 | 0 | 0 | 0 | 0 | 0 | 0 | 0 | 0 | 0 | 0 | 1 | 0 | 0 | 5 | 3 |
| OTU884 | 1 | 0 | 0 | 0 | 0 | 0 | 0 | 0 | 1 | 0 | 0 | 0 | 1 | 5 | 0 | 0 | 11 | 7 |
| OTU885 | 0 | 0 | 0 | 0 | 0 | 0 | 0 | 0 | 0 | 0 | 1 | 0 | 0 | 6 | 0 | 0 | 9 | 3 |
| OTU886 | 1 | 0 | 1 | 0 | 1 | 0 | 0 | 0 | 2 | 1 | 1 | 0 | 1 | 2 | 0 | 0 | 5 | 0 |
| OTU887 | 0 | 0 | 0 | 0 | 0 | 0 | 0 | 0 | 0 | 0 | 0 | 0 | 0 | 0 | 1 | 0 | 3 | 0 |
| OTU888 | 0 | 1 | 0 | 0 | 0 | 0 | 0 | 0 | 0 | 0 | 0 | 0 | 0 | 0 | 0 | 1 | 0 | 2 |
| OTU889 | 0 | 0 | 0 | 0 | 0 | 0 | 0 | 0 | 0 | 0 | 0 | 0 | 0 | 0 | 0 | 1 | 1 | 0 |
| OTU89 | 7 | 5 | 11 | 14 | 49 | 63 | 5 | 55 | 12 | 302 | 297 | 15 | 1 | 7 | 9 | 0 | 2 | 1 |
| OTU890 | 0 | 0 | 0 | 0 | 0 | 0 | 0 | 0 | 0 | 0 | 0 | 0 | 0 | 1 | 0 | 0 | 6 | 0 |
| OTU891 | 0 | 0 | 0 | 0 | 0 | 0 | 0 | 0 | 0 | 0 | 0 | 0 | 0 | 0 | 0 | 0 | 20 | 0 |
| OTU892 | 0 | 0 | 0 | 0 | 0 | 0 | 0 | 0 | 0 | 0 | 0 | 0 | 0 | 0 | 1 | 0 | 40 | 0 |
| OTU893 | 0 | 0 | 0 | 0 | 0 | 0 | 0 | 0 | 1 | 0 | 0 | 0 | 0 | 0 | 0 | 0 | 9 | 0 |
| OTU894 | 0 | 0 | 0 | 0 | 0 | 0 | 0 | 0 | 0 | 0 | 0 | 0 | 0 | 0 | 0 | 0 | 3 | 0 |
| OTU895 | 0 | 0 | 0 | 0 | 0 | 0 | 0 | 0 | 0 | 0 | 0 | 0 | 0 | 0 | 0 | 0 | 7 | 0 |
| OTU896 | 0 | 0 | 0 | 0 | 0 | 0 | 0 | 0 | 0 | 0 | 0 | 0 | 0 | 0 | 0 | 0 | 13 | 0 |
| OTU897 | 0 | 0 | 0 | 0 | 0 | 0 | 0 | 0 | 0 | 0 | 0 | 0 | 4 | 0 | 1 | 0 | 7 | 4 |
| OTU898 | 0 | 0 | 0 | 0 | 0 | 0 | 0 | 0 | 0 | 0 | 0 | 0 | 0 | 0 | 0 | 0 | 5 | 1 |
| OTU899 | 0 | 0 | 0 | 0 | 0 | 0 | 0 | 0 | 0 | 0 | 0 | 0 | 0 | 0 | 0 | 0 | 7 | 0 |
| OTU9 | 234 | 271 | 14 | 17 | 26 | 800 | 9 | 1991 | 610 | 1599 | 3160 | 1098 | 73 | 313 | 372 | 0 | 64 | 35 |
| OTU90 | 1 | 1 | 1 | 3 | 2 | 8 | 8 | 28 | 103 | 172 | 24 | 556 | 21 | 66 | 80 | 0 | 11 | 4 |
| OTU900 | 0 | 0 | 0 | 0 | 0 | 0 | 0 | 0 | 1 | 0 | 0 | 0 | 2 | 2 | 0 | 0 | 6 | 1 |
| OTU901 | 0 | 0 | 0 | 0 | 0 | 0 | 0 | 0 | 0 | 0 | 0 | 0 | 0 | 0 | 0 | 0 | 7 | 0 |
| OTU902 | 0 | 0 | 0 | 0 | 0 | 0 | 0 | 0 | 0 | 0 | 0 | 0 | 0 | 0 | 0 | 0 | 11 | 1 |
| OTU903 | 0 | 0 | 0 | 0 | 0 | 0 | 0 | 0 | 0 | 0 | 0 | 0 | 0 | 0 | 0 | 0 | 2 | 0 |
| OTU904 | 0 | 0 | 0 | 0 | 0 | 0 | 0 | 0 | 0 | 0 | 0 | 0 | 0 | 0 | 0 | 0 | 9 | 0 |
| OTU905 | 1 | 0 | 0 | 0 | 1 | 0 | 0 | 0 | 0 | 0 | 1 | 0 | 2 | 3 | 0 | 0 | 12 | 0 |
| OTU906 | 0 | 0 | 0 | 0 | 0 | 0 | 0 | 0 | 0 | 0 | 0 | 0 | 0 | 0 | 0 | 0 | 23 | 0 |
| OTU907 | 0 | 0 | 0 | 0 | 0 | 0 | 0 | 1 | 0 | 0 | 0 | 0 | 0 | 0 | 0 | 0 | 15 | 0 |
| OTU908 | 0 | 0 | 0 | 0 | 0 | 0 | 0 | 0 | 0 | 0 | 0 | 0 | 0 | 0 | 0 | 0 | 8 | 0 |
| OTU909 | 0 | 0 | 0 | 0 | 0 | 0 | 0 | 0 | 0 | 0 | 0 | 0 | 0 | 0 | 0 | 0 | 12 | 0 |
| OTU91 | 0 | 0 | 1 | 0 | 1 | 0 | 0 | 2 | 1 | 1 | 1 | 2 | 41 | 131 | 0 | 8 | 212 | 197 |
| OTU910 | 0 | 0 | 0 | 0 | 0 | 0 | 0 | 0 | 0 | 0 | 0 | 0 | 0 | 0 | 0 | 0 | 16 | 0 |
| OTU911 | 0 | 0 | 0 | 0 | 0 | 0 | 0 | 0 | 0 | 0 | 0 | 0 | 0 | 0 | 0 | 0 | 5 | 0 |
| OTU912 | 0 | 0 | 0 | 0 | 0 | 0 | 0 | 0 | 0 | 0 | 0 | 0 | 0 | 0 | 0 | 0 | 21 | 0 |
| OTU913 | 0 | 0 | 0 | 0 | 0 | 0 | 0 | 0 | 0 | 0 | 0 | 0 | 0 | 0 | 0 | 0 | 3 | 0 |
| OTU914 | 0 | 0 | 0 | 0 | 0 | 0 | 0 | 0 | 0 | 0 | 0 | 0 | 0 | 0 | 0 | 0 | 17 | 0 |
| OTU915 | 0 | 0 | 0 | 0 | 0 | 0 | 0 | 0 | 0 | 0 | 0 | 0 | 0 | 0 | 0 | 0 | 20 | 0 |
| OTU916 | 0 | 0 | 0 | 0 | 0 | 0 | 0 | 0 | 0 | 0 | 0 | 0 | 0 | 1 | 0 | 0 | 4 | 1 |
| OTU917 | 0 | 0 | 0 | 0 | 0 | 0 | 0 | 0 | 0 | 0 | 0 | 0 | 0 | 0 | 0 | 0 | 24 | 0 |
| OTU918 | 0 | 0 | 0 | 0 | 0 | 0 | 0 | 0 | 0 | 0 | 0 | 0 | 0 | 0 | 0 | 0 | 2 | 0 |
| OTU919 | 0 | 0 | 0 | 0 | 0 | 0 | 0 | 0 | 0 | 0 | 0 | 0 | 0 | 0 | 0 | 0 | 3 | 0 |
| OTU92 | 411 | 113 | 135 | 0 | 0 | 3 | 1 | 0 | 0 | 2 | 4 | 2 | 0 | 1 | 2 | 0 | 0 | 0 |
| OTU920 | 0 | 0 | 0 | 0 | 0 | 0 | 0 | 0 | 0 | 0 | 0 | 0 | 0 | 0 | 0 | 0 | 10 | 0 |
| OTU921 | 0 | 0 | 0 | 0 | 0 | 0 | 0 | 0 | 0 | 0 | 0 | 0 | 0 | 0 | 0 | 0 | 3 | 0 |
| OTU922 | 0 | 0 | 0 | 0 | 0 | 0 | 0 | 0 | 0 | 0 | 0 | 0 | 1 | 2 | 0 | 0 | 10 | 1 |
| OTU923 | 0 | 0 | 0 | 0 | 0 | 0 | 0 | 0 | 0 | 0 | 0 | 0 | 0 | 0 | 0 | 0 | 5 | 0 |
| OTU924 | 0 | 0 | 0 | 0 | 0 | 0 | 0 | 0 | 0 | 0 | 0 | 0 | 0 | 0 | 0 | 0 | 6 | 0 |
| OTU925 | 0 | 0 | 0 | 0 | 0 | 0 | 0 | 0 | 0 | 0 | 0 | 0 | 3 | 0 | 0 | 0 | 6 | 3 |
| OTU926 | 0 | 0 | 0 | 0 | 0 | 0 | 0 | 0 | 0 | 0 | 0 | 0 | 0 | 0 | 0 | 0 | 1 | 8 |
| OTU927 | 0 | 0 | 0 | 0 | 0 | 0 | 0 | 0 | 0 | 0 | 0 | 0 | 0 | 0 | 0 | 0 | 19 | 0 |
| OTU928 | 0 | 0 | 0 | 0 | 0 | 0 | 0 | 0 | 0 | 0 | 0 | 0 | 0 | 0 | 0 | 0 | 3 | 0 |
| OTU929 | 0 | 0 | 0 | 0 | 0 | 0 | 0 | 0 | 0 | 0 | 0 | 0 | 0 | 0 | 0 | 0 | 2 | 0 |
| OTU93 | 277 | 11 | 412 | 5 | 456 | 64 | 4 | 1 | 1 | 5 | 0 | 0 | 0 | 1 | 4 | 0 | 0 | 0 |
| OTU930 | 0 | 0 | 0 | 0 | 0 | 0 | 0 | 0 | 0 | 0 | 0 | 0 | 0 | 0 | 0 | 0 | 13 | 0 |
| OTU931 | 3 | 4 | 2 | 5 | 2 | 5 | 2 | 6 | 4 | 2 | 5 | 3 | 1 | 3 | 2 | 3 | 4 | 1 |
| OTU932 | 0 | 0 | 0 | 0 | 0 | 0 | 0 | 0 | 0 | 0 | 0 | 0 | 1 | 0 | 0 | 0 | 2 | 0 |
| OTU933 | 0 | 0 | 0 | 0 | 0 | 0 | 0 | 0 | 0 | 0 | 0 | 0 | 0 | 0 | 0 | 0 | 5 | 0 |
| OTU934 | 0 | 0 | 0 | 0 | 0 | 0 | 0 | 1 | 0 | 0 | 0 | 0 | 1 | 0 | 1 | 0 | 9 | 0 |
| OTU935 | 0 | 0 | 0 | 0 | 0 | 0 | 0 | 0 | 0 | 0 | 0 | 0 | 0 | 0 | 0 | 0 | 2 | 0 |
| OTU936 | 0 | 0 | 0 | 0 | 0 | 0 | 0 | 0 | 0 | 0 | 0 | 0 | 0 | 0 | 0 | 0 | 8 | 0 |
| OTU937 | 0 | 0 | 0 | 0 | 0 | 0 | 0 | 0 | 0 | 0 | 0 | 0 | 0 | 0 | 0 | 0 | 0 | 2 |
| OTU94 | 24 | 114 | 175 | 129 | 62 | 18 | 0 | 0 | 0 | 1 | 0 | 0 | 1 | 0 | 0 | 0 | 0 | 0 |
| OTU95 | 32 | 12 | 179 | 2 | 8 | 361 | 12 | 582 | 171 | 631 | 473 | 205 | 3 | 3 | 116 | 0 | 8 | 6 |
| OTU96 | 56 | 846 | 303 | 33 | 119 | 57 | 5 | 6 | 8 | 5 | 6 | 5 | 1 | 1 | 10 | 0 | 1 | 0 |
| OTU97 | 1 | 0 | 0 | 4 | 4 | 4 | 75 | 23 | 84 | 336 | 90 | 16 | 8 | 10 | 53 | 0 | 3 | 14 |
| OTU98 | 0 | 0 | 0 | 0 | 0 | 0 | 0 | 0 | 0 | 0 | 0 | 1 | 15 | 87 | 0 | 9 | 223 | 249 |
| OTU99 | 3 | 2 | 7 | 0 | 10 | 2 | 19 | 12 | 18 | 92 | 148 | 225 | 1 | 1 | 18 | 0 | 6 | 3 |

## genus count for Fig 6C

| genus | WT1 | WT2 | WT3 | WT4 | WT5 | WT6 | Uox^-/-^-  1-1 | Uox^-/-^-  1-2 | Uox^-/-^-  1-3 | Uox^-/-^-  1-4 | Uox^-/-^-  1-5 | Uox^-/-^-  1-6 | Uox^-/-^-  2-1 | Uox^-/-^-  2-2 | Uox^-/-^-  2-3 | Uox^-/-^-  2-4 | Uox^-/-^-2-5 | Uox-/--2-6 |
| --- | --- | --- | --- | --- | --- | --- | --- | --- | --- | --- | --- | --- | --- | --- | --- | --- | --- | --- |
| g__14-2 | 13 | 30 | 30 | 10 | 413 | 29 | 5 | 64 | 21 | 172 | 156 | 44 | 1 | 8 | 19 | 0 | 10 | 1 |
| g__21-14-0-10-47-8-A | 0 | 0 | 0 | 0 | 0 | 0 | 0 | 0 | 0 | 0 | 0 | 0 | 0 | 0 | 0 | 0 | 6 | 0 |
| g__33-17 | 0 | 0 | 0 | 0 | 0 | 0 | 0 | 0 | 0 | 0 | 1 | 3 | 1 | 1 | 0 | 0 | 0 | 0 |
| g__49-20 | 4 | 7 | 4 | 2 | 6 | 7 | 5 | 5 | 7 | 9 | 10 | 4 | 1 | 9 | 3 | 8 | 5 | 4 |
| g__992a | 140 | 62 | 34 | 44 | 1396 | 1370 | 141 | 406 | 145 | 256 | 681 | 243 | 14 | 14 | 118 | 1 | 16 | 8 |
| g__ASF356 | 0 | 6 | 0 | 0 | 5 | 15 | 0 | 0 | 3 | 0 | 0 | 0 | 0 | 0 | 3 | 0 | 0 | 1 |
| g__Absiella | 68 | 1 | 8 | 0 | 3 | 1 | 7 | 5 | 23 | 8 | 12 | 12 | 8 | 8 | 17 | 0 | 2 | 1 |
| g__Acetatifactor | 0 | 0 | 0 | 0 | 0 | 0 | 0 | 4 | 1 | 1 | 0 | 0 | 0 | 0 | 0 | 0 | 0 | 0 |
| g__Acetitomaculum | 0 | 3 | 0 | 3 | 14 | 0 | 2 | 39 | 11 | 2 | 21 | 67 | 1 | 11 | 10 | 0 | 0 | 0 |
| g__Acetivibrio | 7 | 14 | 10 | 4 | 1 | 4 | 1 | 1 | 1 | 1 | 0 | 2 | 0 | 1 | 1 | 0 | 0 | 0 |
| g__Acetoanaerobium | 0 | 3 | 0 | 1 | 0 | 1 | 0 | 0 | 1 | 2 | 0 | 0 | 0 | 0 | 0 | 0 | 0 | 0 |
| g__Acetobacter | 0 | 0 | 0 | 0 | 0 | 0 | 0 | 0 | 0 | 0 | 0 | 0 | 0 | 0 | 0 | 0 | 16 | 0 |
| g__Achromobacter | 0 | 0 | 0 | 0 | 0 | 0 | 0 | 0 | 0 | 0 | 0 | 0 | 0 | 0 | 0 | 0 | 13 | 0 |
| g__Acinetobacter | 0 | 1 | 3 | 0 | 4 | 3 | 5 | 0 | 1 | 0 | 1 | 4 | 25 | 13 | 3 | 0 | 270 | 13 |
| g__Actinomadura_B | 1 | 1 | 1 | 1 | 1 | 0 | 0 | 1 | 2 | 0 | 0 | 0 | 2 | 0 | 1 | 0 | 0 | 0 |
| g__Actinomyces | 0 | 1 | 1 | 0 | 0 | 1 | 3 | 3 | 0 | 2 | 1 | 2 | 46 | 57 | 1 | 43 | 647 | 279 |
| g__Actinomyces_I | 1 | 2 | 1 | 0 | 0 | 4 | 1 | 4 | 1 | 0 | 1 | 2 | 1 | 1 | 0 | 1 | 7 | 1 |
| g__Acutalibacter | 30 | 20 | 45 | 41 | 70 | 94 | 25 | 243 | 129 | 234 | 232 | 438 | 41 | 143 | 122 | 0 | 13 | 5 |
| g__Adlercreutzia | 53 | 76 | 117 | 43 | 27 | 60 | 6 | 28 | 16 | 5 | 8 | 9 | 3 | 9 | 12 | 0 | 4 | 5 |
| g__Advenella | 0 | 0 | 0 | 0 | 0 | 0 | 0 | 0 | 0 | 0 | 0 | 0 | 0 | 0 | 0 | 0 | 297 | 0 |
| g__Agathobacter | 32 | 12 | 179 | 2 | 8 | 361 | 12 | 582 | 171 | 631 | 473 | 205 | 3 | 3 | 116 | 0 | 8 | 6 |
| g__Agathobaculum | 1 | 4 | 4 | 2 | 3 | 12 | 2 | 4 | 6 | 4 | 7 | 2 | 0 | 1 | 1 | 0 | 0 | 0 |
| g__Agrobacterium | 0 | 1 | 1 | 0 | 1 | 0 | 1 | 2 | 3 | 2 | 4 | 2 | 3 | 4 | 1 | 1 | 1037 | 4 |
| g__Alicyclobacillus_B | 0 | 1 | 0 | 0 | 0 | 1 | 1 | 1 | 1 | 0 | 0 | 1 | 0 | 1 | 1 | 1 | 0 | 0 |
| g__Alicyclobacillus_H | 0 | 1 | 0 | 0 | 0 | 0 | 0 | 2 | 0 | 0 | 0 | 1 | 1 | 0 | 0 | 1 | 0 | 0 |
| g__Alistipes | 1 | 4 | 19 | 13 | 47 | 12 | 11 | 0 | 16 | 5 | 4 | 0 | 1 | 0 | 13 | 0 | 0 | 2 |
| g__Alistipes_A | 0 | 6 | 0 | 4 | 29 | 2 | 0 | 0 | 0 | 0 | 1 | 0 | 0 | 0 | 0 | 0 | 0 | 0 |
| g__Allobaculum | 7 | 18 | 66 | 0 | 1 | 3 | 0 | 0 | 0 | 0 | 0 | 0 | 0 | 0 | 0 | 0 | 0 | 0 |
| g__Aminicenans | 0 | 1 | 0 | 0 | 0 | 1 | 0 | 0 | 1 | 0 | 2 | 2 | 0 | 1 | 0 | 0 | 0 | 0 |
| g__An181 | 9 | 148 | 23 | 1 | 19 | 0 | 0 | 0 | 0 | 0 | 0 | 0 | 0 | 0 | 0 | 0 | 0 | 0 |
| g__Anaerobiospirillum_A | 0 | 2 | 2 | 0 | 4 | 2 | 2 | 750 | 338 | 582 | 1203 | 765 | 6 | 6 | 66 | 1 | 8 | 2 |
| g__Anaerocolumna | 0 | 0 | 0 | 1 | 0 | 0 | 0 | 0 | 6 | 5 | 0 | 0 | 0 | 0 | 4 | 0 | 1 | 0 |
| g__Anaerofustis | 5 | 0 | 3 | 2 | 0 | 1 | 1 | 3 | 0 | 0 | 5 | 0 | 0 | 0 | 1 | 0 | 0 | 0 |
| g__Anaeromassilibacillus | 45 | 49 | 53 | 26 | 20 | 76 | 0 | 0 | 0 | 0 | 0 | 0 | 0 | 0 | 0 | 0 | 0 | 0 |
| g__Anaerostipes | 74 | 231 | 371 | 7 | 6 | 326 | 157 | 106 | 83 | 91 | 153 | 235 | 12 | 78 | 70 | 1 | 18 | 14 |
| g__Anaerotignum | 1 | 7 | 2 | 3 | 2 | 1 | 5 | 53 | 20 | 34 | 67 | 13 | 0 | 1 | 16 | 0 | 3 | 1 |
| g__Anaerotruncus | 5 | 26 | 13 | 52 | 120 | 96 | 3 | 6 | 3 | 2 | 4 | 8 | 0 | 0 | 5 | 0 | 0 | 1 |
| g__Anaerovibrio | 0 | 0 | 0 | 0 | 0 | 0 | 1 | 11 | 32 | 1 | 0 | 1 | 0 | 0 | 9 | 0 | 0 | 0 |
| g__Angelakisella | 5 | 2 | 2 | 6 | 13 | 12 | 2 | 7 | 9 | 8 | 10 | 6 | 0 | 1 | 4 | 0 | 0 | 0 |
| g__Aquamicrobium_A | 0 | 0 | 2 | 1 | 0 | 0 | 1 | 1 | 0 | 0 | 1 | 0 | 15 | 17 | 0 | 3 | 63 | 34 |
| g__Arboricoccus | 0 | 0 | 0 | 0 | 0 | 0 | 0 | 1 | 2 | 0 | 0 | 1 | 0 | 0 | 0 | 0 | 1 | 0 |
| g__Atopostipes | 0 | 0 | 0 | 0 | 0 | 0 | 0 | 0 | 0 | 0 | 0 | 0 | 0 | 0 | 0 | 0 | 11 | 1 |
| g__Bacillus | 0 | 1 | 0 | 1 | 0 | 0 | 0 | 0 | 0 | 0 | 0 | 0 | 7 | 5 | 0 | 0 | 71 | 19 |
| g__Bacillus_A | 0 | 0 | 0 | 0 | 0 | 0 | 0 | 0 | 0 | 0 | 0 | 0 | 0 | 0 | 0 | 1 | 66 | 2 |
| g__Bacillus_C | 1 | 0 | 0 | 0 | 0 | 1 | 0 | 0 | 1 | 1 | 0 | 0 | 3 | 4 | 3 | 2 | 27 | 10 |
| g__Bacillus_J | 0 | 0 | 0 | 0 | 0 | 0 | 0 | 0 | 0 | 0 | 0 | 0 | 1 | 4 | 0 | 1 | 28 | 3 |
| g__Bacillus_W | 5 | 1 | 1 | 1 | 0 | 0 | 0 | 0 | 1 | 1 | 1 | 1 | 3 | 1 | 0 | 4 | 4 | 3 |
| g__Bact-08 | 0 | 0 | 0 | 0 | 0 | 0 | 7 | 0 | 3 | 0 | 9 | 9 | 0 | 3 | 0 | 0 | 0 | 0 |
| g__Bacteroides | 177 | 333 | 286 | 485 | 420 | 139 | 166 | 49 | 52 | 80 | 34 | 149 | 18 | 39 | 37 | 0 | 11 | 4 |
| g__Bacteroides_B | 159 | 212 | 269 | 35 | 632 | 88 | 68 | 7 | 8 | 14 | 23 | 38 | 3 | 7 | 22 | 0 | 1 | 1 |
| g__Bacteroides_F | 76 | 27 | 1 | 8 | 1 | 408 | 0 | 52 | 202 | 4 | 5 | 427 | 22 | 58 | 129 | 0 | 10 | 1 |
| g__Beduini | 7 | 1 | 1 | 8 | 1 | 0 | 12 | 6 | 3 | 6 | 0 | 18 | 2 | 9 | 9 | 0 | 0 | 1 |
| g__Beta-01 | 0 | 0 | 0 | 0 | 0 | 0 | 0 | 0 | 0 | 0 | 0 | 0 | 1 | 7 | 0 | 0 | 1 | 6 |
| g__Bifidobacterium | 8 | 10 | 27 | 10 | 27 | 10 | 10 | 21 | 12 | 13 | 11 | 18 | 30 | 24 | 15 | 39 | 17 | 27 |
| g__Bilophila | 0 | 0 | 0 | 3 | 32 | 4 | 1 | 0 | 2 | 10 | 8 | 10 | 0 | 1 | 1 | 0 | 1 | 0 |
| g__Bittarella | 6 | 13 | 3 | 3 | 9 | 11 | 0 | 0 | 0 | 0 | 0 | 0 | 0 | 0 | 1 | 0 | 0 | 0 |
| g__Blastococcus | 6 | 55 | 1515 | 8 | 7 | 10 | 1 | 1 | 0 | 0 | 3 | 1 | 0 | 0 | 1 | 0 | 2 | 0 |
| g__Blautia_A | 56 | 851 | 313 | 39 | 120 | 57 | 5 | 13 | 14 | 7 | 6 | 10 | 4 | 12 | 12 | 0 | 2 | 1 |
| g__Bog-159 | 0 | 1 | 0 | 0 | 0 | 0 | 0 | 0 | 0 | 0 | 0 | 0 | 0 | 0 | 1 | 0 | 0 | 0 |
| g__Bosea | 0 | 0 | 0 | 0 | 0 | 0 | 0 | 0 | 0 | 0 | 0 | 0 | 0 | 1 | 0 | 0 | 18 | 5 |
| g__Brachybacterium | 0 | 0 | 0 | 0 | 0 | 0 | 0 | 1 | 0 | 1 | 0 | 0 | 10 | 10 | 0 | 0 | 46 | 16 |
| g__Brachyspira | 0 | 0 | 0 | 0 | 0 | 0 | 0 | 0 | 0 | 0 | 0 | 0 | 0 | 0 | 0 | 0 | 10 | 0 |
| g__Bradyrhizobium | 0 | 1 | 1 | 0 | 0 | 0 | 0 | 0 | 1 | 0 | 2 | 0 | 4 | 3 | 0 | 0 | 13 | 8 |
| g__Brevibacillus | 0 | 0 | 0 | 0 | 0 | 0 | 0 | 0 | 1 | 0 | 0 | 0 | 2 | 2 | 0 | 0 | 6 | 1 |
| g__Brevibacterium | 0 | 1 | 0 | 0 | 0 | 0 | 0 | 0 | 0 | 0 | 0 | 0 | 11 | 5 | 0 | 0 | 64 | 17 |
| g__Brevundimonas | 0 | 0 | 0 | 0 | 0 | 0 | 0 | 0 | 0 | 0 | 0 | 0 | 15 | 2 | 0 | 0 | 17 | 7 |
| g__Butyricicoccus | 0 | 5 | 2 | 1 | 0 | 14 | 0 | 15 | 1 | 24 | 24 | 5 | 2 | 2 | 5 | 0 | 1 | 0 |
| g__Butyricicoccus_A | 0 | 0 | 1 | 1 | 0 | 0 | 1 | 0 | 1 | 0 | 2 | 3 | 1 | 1 | 1 | 0 | 0 | 0 |
| g__Butyricimonas | 2 | 8 | 6 | 2 | 8 | 6 | 0 | 0 | 0 | 1 | 1 | 0 | 0 | 0 | 0 | 0 | 0 | 0 |
| g__Butyrivibrio | 1 | 0 | 0 | 0 | 0 | 6 | 0 | 3 | 0 | 0 | 1 | 0 | 0 | 0 | 0 | 0 | 0 | 0 |
| g__Butyrivibrio_A | 2 | 14 | 13 | 3 | 10 | 137 | 5 | 505 | 1538 | 23 | 961 | 862 | 23 | 53 | 939 | 2 | 49 | 31 |
| g__CAG-1031 | 50 | 21 | 49 | 34 | 62 | 76 | 8 | 2 | 11 | 16 | 30 | 6 | 0 | 3 | 12 | 0 | 3 | 0 |
| g__CAG-110 | 34 | 15 | 35 | 6 | 2 | 29 | 8 | 1 | 2 | 4 | 9 | 1 | 1 | 1 | 4 | 0 | 0 | 1 |
| g__CAG-194 | 0 | 4 | 2 | 0 | 9 | 13 | 10 | 65 | 95 | 31 | 58 | 21 | 2 | 4 | 67 | 0 | 8 | 4 |
| g__CAG-217 | 35 | 564 | 56 | 12 | 176 | 135 | 77 | 1 | 101 | 11 | 8 | 3 | 0 | 2 | 170 | 0 | 3 | 1 |
| g__CAG-302 | 1 | 2 | 16 | 1 | 0 | 3 | 7 | 0 | 2 | 0 | 0 | 1 | 0 | 0 | 1 | 0 | 0 | 0 |
| g__CAG-306 | 3 | 1 | 1 | 9 | 5 | 3 | 1 | 2 | 2 | 4 | 5 | 8 | 1 | 9 | 3 | 0 | 2 | 2 |
| g__CAG-353 | 5 | 0 | 22 | 0 | 0 | 1 | 12 | 141 | 3 | 217 | 126 | 21 | 1 | 0 | 8 | 0 | 6 | 2 |
| g__CAG-41 | 51 | 112 | 42 | 55 | 52 | 201 | 168 | 229 | 494 | 431 | 881 | 564 | 73 | 260 | 594 | 0 | 61 | 35 |
| g__CAG-45 | 0 | 105 | 1 | 0 | 0 | 0 | 0 | 0 | 0 | 1 | 0 | 1 | 0 | 0 | 0 | 0 | 0 | 0 |
| g__CAG-484 | 3 | 5 | 0 | 3 | 0 | 6 | 8 | 3 | 2 | 3 | 14 | 4 | 1 | 4 | 3 | 0 | 0 | 0 |
| g__CAG-495 | 2 | 4 | 1 | 2 | 7 | 0 | 26 | 4 | 2 | 3 | 1 | 2 | 1 | 1 | 2 | 0 | 0 | 0 |
| g__CAG-791 | 1 | 0 | 3 | 0 | 0 | 0 | 3 | 35 | 12 | 3 | 12 | 14 | 1 | 5 | 2 | 0 | 0 | 1 |
| g__CAG-81 | 1 | 4 | 6 | 1 | 21 | 170 | 1 | 22 | 17 | 20 | 8 | 16 | 5 | 12 | 15 | 0 | 2 | 2 |
| g__CAG-822 | 1 | 0 | 0 | 0 | 0 | 0 | 5 | 5 | 48 | 1 | 0 | 24 | 3 | 8 | 47 | 0 | 2 | 1 |
| g__CAG-878 | 21 | 0 | 17 | 0 | 1 | 1 | 0 | 16 | 2 | 0 | 0 | 1 | 0 | 0 | 4 | 0 | 0 | 0 |
| g__CAG-95 | 2 | 9 | 48 | 2 | 6 | 53 | 7 | 145 | 158 | 122 | 161 | 260 | 16 | 22 | 106 | 0 | 10 | 3 |
| g__CAG-988 | 0 | 0 | 0 | 0 | 0 | 1 | 0 | 0 | 5 | 1 | 0 | 0 | 0 | 1 | 3 | 0 | 0 | 0 |
| g__CDF | 0 | 0 | 0 | 0 | 0 | 0 | 0 | 0 | 0 | 0 | 0 | 0 | 1 | 1 | 0 | 1 | 19 | 3 |
| g__CG2-30-66-27 | 1 | 2 | 2 | 0 | 3 | 5 | 2 | 3 | 2 | 5 | 2 | 3 | 1 | 1 | 2 | 1 | 1 | 0 |
| g__CHKCI006 | 0 | 87 | 0 | 0 | 1 | 1 | 3 | 0 | 1 | 0 | 1 | 6 | 0 | 1 | 0 | 0 | 0 | 0 |
| g__COE1 | 28 | 1 | 1 | 36 | 7 | 65 | 4 | 34 | 8 | 17 | 15 | 7 | 1 | 0 | 6 | 0 | 1 | 0 |
| g__Caldilinea | 0 | 0 | 0 | 0 | 0 | 0 | 0 | 0 | 0 | 0 | 0 | 0 | 0 | 0 | 0 | 0 | 2 | 0 |
| g__Christensenella | 27 | 13 | 17 | 15 | 24 | 18 | 6 | 12 | 3 | 10 | 10 | 14 | 2 | 3 | 9 | 0 | 1 | 0 |
| g__Christensenella_A | 2 | 0 | 1 | 4 | 1 | 4 | 4 | 0 | 6 | 3 | 3 | 5 | 1 | 6 | 2 | 0 | 0 | 1 |
| g__Chryseobacterium | 141 | 1285 | 1562 | 622 | 209 | 697 | 108 | 367 | 78 | 50 | 56 | 140 | 12 | 19 | 106 | 1 | 7 | 8 |
| g__Citrobacter | 0 | 0 | 0 | 0 | 1 | 1 | 0 | 0 | 0 | 0 | 0 | 0 | 2 | 4 | 0 | 1 | 63 | 19 |
| g__Clostridium | 0 | 0 | 1 | 0 | 1 | 5 | 0 | 0 | 0 | 3 | 0 | 0 | 8 | 3 | 0 | 1 | 50 | 4 |
| g__Clostridium_A | 0 | 0 | 0 | 0 | 0 | 0 | 1 | 3 | 2 | 1 | 3 | 2 | 0 | 0 | 2 | 0 | 0 | 0 |
| g__Clostridium_AM | 0 | 0 | 0 | 0 | 0 | 0 | 0 | 1 | 0 | 0 | 0 | 0 | 0 | 0 | 0 | 0 | 26 | 0 |
| g__Clostridium_B | 0 | 0 | 0 | 0 | 0 | 0 | 0 | 0 | 0 | 0 | 0 | 0 | 0 | 0 | 0 | 0 | 86 | 0 |
| g__Clostridium_C | 0 | 1 | 0 | 0 | 0 | 0 | 0 | 0 | 0 | 0 | 0 | 0 | 0 | 0 | 0 | 0 | 27 | 0 |
| g__Clostridium_H | 0 | 0 | 0 | 0 | 0 | 0 | 0 | 0 | 0 | 0 | 0 | 0 | 0 | 0 | 0 | 0 | 5 | 0 |
| g__Clostridium_N | 3 | 47 | 3 | 47 | 0 | 1 | 0 | 17 | 58 | 1 | 0 | 6 | 2 | 1 | 33 | 0 | 7 | 2 |
| g__Clostridium_Q | 18 | 23 | 55 | 18 | 779 | 351 | 7 | 126 | 99 | 112 | 88 | 70 | 3 | 9 | 65 | 0 | 10 | 9 |
| g__Clostridium_S | 0 | 0 | 0 | 0 | 0 | 0 | 1 | 0 | 0 | 0 | 0 | 0 | 1 | 0 | 0 | 0 | 178 | 0 |
| g__Collinsella | 8 | 10 | 7 | 1 | 0 | 2 | 3 | 13 | 9 | 11 | 7 | 8 | 1 | 8 | 14 | 0 | 2 | 0 |
| g__Comamonas_D | 0 | 0 | 0 | 0 | 0 | 0 | 0 | 0 | 0 | 0 | 0 | 0 | 0 | 0 | 0 | 0 | 7 | 0 |
| g__Coprobacter | 1 | 0 | 3 | 3 | 0 | 2 | 0 | 0 | 0 | 0 | 0 | 0 | 0 | 0 | 0 | 0 | 0 | 0 |
| g__Coprococcus_B | 1 | 8 | 11 | 0 | 0 | 28 | 1 | 30 | 4 | 15 | 25 | 6 | 3 | 4 | 4 | 0 | 1 | 0 |
| g__Corynebacterium | 2 | 4 | 3 | 3 | 2 | 1 | 5 | 9 | 6 | 8 | 7 | 6 | 157 | 308 | 3 | 15 | 699 | 501 |
| g__Cronobacter | 10870 | 17262 | 10095 | 10552 | 9337 | 8871 | 5817 | 4539 | 3421 | 8329 | 7306 | 4970 | 973 | 2355 | 2984 | 1554 | 526 | 280 |
| g__Cryobacterium_A | 1 | 0 | 0 | 0 | 0 | 0 | 0 | 0 | 1 | 0 | 0 | 0 | 1 | 5 | 0 | 0 | 11 | 7 |
| g__DNF00809 | 10 | 6 | 17 | 9 | 8 | 5 | 5 | 17 | 4 | 3 | 7 | 10 | 0 | 18 | 7 | 0 | 2 | 3 |
| g__DTU053 | 9 | 4 | 2 | 0 | 7 | 0 | 0 | 0 | 0 | 0 | 0 | 0 | 0 | 0 | 0 | 0 | 0 | 0 |
| g__Dakarella | 0 | 0 | 0 | 0 | 0 | 0 | 0 | 0 | 0 | 0 | 0 | 0 | 0 | 0 | 0 | 0 | 152 | 1 |
| g__Defluviitalea | 1 | 0 | 6 | 0 | 0 | 3 | 1 | 0 | 1 | 0 | 0 | 4 | 0 | 0 | 1 | 0 | 0 | 0 |
| g__Dehalobacterium | 9 | 11 | 4 | 0 | 2 | 1 | 1 | 0 | 1 | 1 | 1 | 3 | 0 | 0 | 0 | 0 | 0 | 0 |
| g__Denitrobacterium | 0 | 0 | 0 | 0 | 0 | 0 | 19 | 14 | 18 | 5 | 7 | 20 | 0 | 9 | 13 | 0 | 3 | 0 |
| g__Desulfonispora | 2 | 3 | 23 | 3 | 1 | 0 | 5 | 0 | 0 | 0 | 0 | 0 | 0 | 0 | 0 | 0 | 0 | 0 |
| g__Desulfovibrio | 1 | 160 | 78 | 2 | 208 | 360 | 46 | 507 | 16 | 308 | 358 | 369 | 30 | 6 | 270 | 1 | 27 | 19 |
| g__Desulfuromonas_B | 0 | 0 | 0 | 0 | 0 | 0 | 0 | 0 | 0 | 0 | 0 | 0 | 0 | 1 | 0 | 0 | 4 | 1 |
| g__Devosia | 0 | 0 | 0 | 0 | 0 | 0 | 0 | 0 | 0 | 0 | 0 | 0 | 10 | 13 | 1 | 0 | 121 | 35 |
| g__Dialister | 0 | 0 | 0 | 0 | 0 | 0 | 0 | 0 | 0 | 0 | 0 | 0 | 0 | 0 | 0 | 0 | 24 | 0 |
| g__Dietzia | 0 | 0 | 0 | 0 | 0 | 0 | 0 | 0 | 0 | 0 | 0 | 0 | 8 | 40 | 0 | 1 | 56 | 35 |
| g__Dorea | 12 | 63 | 55 | 23 | 77 | 130 | 26 | 204 | 206 | 249 | 266 | 138 | 10 | 45 | 153 | 2 | 23 | 18 |
| g__Duncaniella | 540 | 366 | 528 | 877 | 600 | 491 | 158 | 144 | 126 | 443 | 190 | 130 | 23 | 48 | 84 | 0 | 15 | 26 |
| g__ER4 | 2 | 0 | 0 | 0 | 3 | 8 | 0 | 1 | 0 | 1 | 2 | 2 | 0 | 0 | 0 | 0 | 0 | 0 |
| g__Effusibacillus | 0 | 3 | 3 | 3 | 2 | 0 | 1 | 3 | 0 | 0 | 1 | 0 | 2 | 5 | 4 | 2 | 0 | 0 |
| g__Eggerthella | 0 | 0 | 0 | 0 | 0 | 0 | 2 | 13 | 12 | 15 | 5 | 7 | 4 | 15 | 8 | 0 | 1 | 0 |
| g__Eisenbergiella | 13 | 3 | 3 | 1 | 23 | 23 | 2 | 24 | 12 | 14 | 15 | 7 | 0 | 1 | 7 | 0 | 0 | 0 |
| g__Emergencia | 46 | 42 | 38 | 17 | 30 | 33 | 13 | 5 | 4 | 8 | 6 | 8 | 3 | 0 | 9 | 1 | 0 | 1 |
| g__Enterococcus | 180 | 207 | 165 | 163 | 248 | 233 | 197 | 382 | 282 | 342 | 228 | 256 | 591 | 602 | 341 | 760 | 223 | 390 |
| g__Enteroscipio | 1 | 4 | 20 | 2 | 5 | 3 | 4 | 12 | 12 | 15 | 14 | 10 | 3 | 4 | 6 | 0 | 2 | 1 |
| g__Erwinia | 4 | 0 | 1 | 3 | 0 | 20 | 0 | 0 | 0 | 1 | 2 | 3 | 0 | 0 | 0 | 0 | 0 | 0 |
| g__Erysipelatoclostridium | 0 | 28 | 2 | 0 | 0 | 22 | 11 | 3 | 10 | 0 | 0 | 22 | 1 | 2 | 6 | 0 | 2 | 1 |
| g__Escherichia | 3 | 2 | 3 | 1 | 1 | 3 | 53 | 149 | 730 | 349 | 177 | 328 | 9 | 37 | 378 | 0 | 21 | 12 |
| g__Eubacterium_A | 0 | 0 | 0 | 0 | 0 | 0 | 2 | 3 | 5 | 0 | 0 | 1 | 1 | 1 | 3 | 0 | 2 | 0 |
| g__Eubacterium_C | 0 | 1 | 0 | 0 | 0 | 2 | 2 | 14 | 6 | 7 | 9 | 4 | 0 | 1 | 7 | 0 | 1 | 0 |
| g__Eubacterium_F | 0 | 0 | 0 | 0 | 0 | 0 | 2 | 39 | 35 | 0 | 2 | 75 | 6 | 10 | 32 | 0 | 1 | 0 |
| g__Eubacterium_G | 14 | 4 | 19 | 8 | 12 | 2 | 9 | 6 | 0 | 26 | 16 | 17 | 0 | 0 | 0 | 0 | 1 | 0 |
| g__Eubacterium_I | 12 | 0 | 30 | 0 | 1 | 0 | 1 | 0 | 0 | 1 | 0 | 2 | 0 | 0 | 0 | 0 | 0 | 0 |
| g__Eubacterium_J | 0 | 8 | 9 | 68 | 0 | 9 | 0 | 4 | 10 | 5 | 2 | 2 | 0 | 1 | 3 | 0 | 1 | 0 |
| g__Eubacterium_Q | 3 | 82 | 2 | 118 | 1 | 223 | 10 | 33 | 265 | 9 | 240 | 169 | 8 | 10 | 198 | 0 | 7 | 14 |
| g__Eubacterium_R | 1836 | 245 | 885 | 1141 | 444 | 309 | 1552 | 486 | 437 | 435 | 311 | 553 | 56 | 246 | 428 | 2 | 66 | 32 |
| g__Exiguobacterium | 3 | 4 | 2 | 2 | 6 | 2 | 8 | 6 | 8 | 5 | 4 | 3 | 7 | 2 | 2 | 11 | 4 | 4 |
| g__Exiguobacterium_A | 4 | 10 | 4 | 2 | 7 | 3 | 10 | 7 | 5 | 12 | 6 | 9 | 21 | 15 | 8 | 22 | 4 | 19 |
| g__F0428 | 0 | 0 | 0 | 0 | 0 | 0 | 0 | 0 | 0 | 0 | 0 | 0 | 0 | 1 | 0 | 0 | 5 | 3 |
| g__Facklamia | 0 | 0 | 0 | 0 | 0 | 0 | 1 | 0 | 0 | 0 | 0 | 0 | 1 | 0 | 0 | 1 | 14 | 4 |
| g__Faecalibacterium | 0 | 0 | 0 | 0 | 2 | 2 | 1 | 2 | 0 | 1 | 1 | 1 | 0 | 0 | 1 | 0 | 20 | 0 |
| g__Faecalibaculum | 9 | 13 | 146 | 4 | 2 | 20 | 0 | 0 | 0 | 0 | 0 | 1 | 0 | 0 | 0 | 0 | 0 | 1 |
| g__Faecalicatena | 38 | 15 | 93 | 58 | 91 | 375 | 18 | 301 | 72 | 200 | 324 | 60 | 18 | 10 | 64 | 1 | 8 | 8 |
| g__Fermentibacter | 1 | 1 | 0 | 0 | 0 | 1 | 0 | 2 | 1 | 0 | 1 | 0 | 1 | 2 | 0 | 3 | 1 | 0 |
| g__Flavisolibacter | 0 | 0 | 0 | 0 | 2 | 0 | 0 | 0 | 0 | 2 | 0 | 0 | 0 | 0 | 0 | 0 | 0 | 0 |
| g__Flavobacterium | 0 | 0 | 0 | 0 | 0 | 0 | 0 | 0 | 0 | 0 | 0 | 0 | 0 | 0 | 0 | 0 | 7 | 0 |
| g__Flavobacterium_A | 0 | 0 | 0 | 0 | 0 | 0 | 0 | 0 | 0 | 0 | 0 | 0 | 0 | 0 | 0 | 0 | 5 | 1 |
| g__Flavonifractor | 50 | 91 | 108 | 106 | 140 | 137 | 185 | 523 | 292 | 391 | 371 | 375 | 29 | 56 | 182 | 2 | 17 | 14 |
| g__Fournierella | 30 | 43 | 33 | 14 | 107 | 38 | 13 | 115 | 30 | 57 | 8 | 15 | 2 | 4 | 18 | 0 | 3 | 0 |
| g__GCA-900066575 | 15 | 51 | 32 | 22 | 13 | 43 | 4 | 99 | 52 | 48 | 61 | 42 | 2 | 3 | 37 | 0 | 10 | 1 |
| g__GCA-900066905 | 0 | 0 | 1 | 0 | 1 | 0 | 0 | 0 | 0 | 0 | 0 | 0 | 0 | 0 | 1 | 0 | 0 | 0 |
| g__GW-Nitrospira-1 | 0 | 1 | 1 | 0 | 1 | 0 | 1 | 2 | 0 | 1 | 1 | 2 | 0 | 0 | 0 | 0 | 0 | 0 |
| g__Gemmata | 0 | 0 | 0 | 0 | 0 | 0 | 0 | 0 | 0 | 0 | 0 | 0 | 0 | 0 | 0 | 0 | 3 | 0 |
| g__Globicatella | 0 | 0 | 0 | 0 | 0 | 0 | 2 | 0 | 1 | 0 | 1 | 4 | 0 | 0 | 0 | 0 | 0 | 0 |
| g__Gordonibacter | 2 | 6 | 2 | 7 | 11 | 6 | 0 | 3 | 1 | 3 | 0 | 4 | 1 | 3 | 0 | 0 | 0 | 0 |
| g__Gracilibacter | 0 | 0 | 0 | 0 | 1 | 0 | 1 | 0 | 0 | 0 | 0 | 2 | 0 | 0 | 0 | 0 | 0 | 0 |
| g__Halomonas | 0 | 0 | 0 | 0 | 0 | 0 | 0 | 0 | 0 | 0 | 0 | 0 | 2 | 2 | 0 | 2 | 22 | 8 |
| g__Halomonas_D | 0 | 1 | 0 | 0 | 0 | 0 | 1 | 0 | 1 | 0 | 0 | 0 | 0 | 0 | 0 | 1 | 0 | 2 |
| g__Helicobacter_C | 92 | 18 | 105 | 46 | 22 | 283 | 1 | 4 | 11 | 31 | 2 | 57 | 4 | 6 | 21 | 0 | 0 | 2 |
| g__Helicobacter_D | 1 | 2 | 6 | 3 | 0 | 22 | 3 | 31 | 12 | 265 | 35 | 34 | 1 | 3 | 55 | 0 | 1 | 2 |
| g__Holdemania | 4 | 3 | 11 | 0 | 3 | 9 | 0 | 4 | 3 | 3 | 7 | 5 | 0 | 0 | 0 | 0 | 0 | 0 |
| g__Hydrogenoanaero  bacterium | 3 | 3 | 5 | 1 | 3 | 1 | 2 | 2 | 1 | 0 | 1 | 3 | 0 | 1 | 0 | 0 | 0 | 0 |
| g__Hydrogenophaga | 0 | 0 | 0 | 0 | 0 | 0 | 0 | 0 | 0 | 0 | 1 | 0 | 0 | 0 | 0 | 0 | 151 | 0 |
| g__Hyphomicrobium | 2 | 0 | 0 | 0 | 1 | 2 | 0 | 1 | 2 | 0 | 3 | 1 | 0 | 0 | 1 | 0 | 2 | 0 |
| g__Intestinibacillus | 0 | 0 | 0 | 0 | 0 | 0 | 0 | 1 | 1 | 1 | 1 | 2 | 0 | 0 | 1 | 0 | 1 | 0 |
| g__Intestinimonas | 15 | 177 | 71 | 18 | 18 | 7 | 175 | 63 | 114 | 80 | 374 | 83 | 26 | 54 | 87 | 0 | 10 | 8 |
| g__JJ008 | 0 | 0 | 0 | 0 | 0 | 0 | 0 | 0 | 0 | 0 | 0 | 0 | 0 | 0 | 0 | 0 | 7 | 0 |
| g__Jeotgalicoccus | 0 | 0 | 0 | 0 | 0 | 0 | 0 | 0 | 0 | 0 | 0 | 0 | 10 | 2 | 0 | 1 | 30 | 8 |
| g__Kineothrix | 251 | 338 | 38 | 103 | 111 | 1842 | 18 | 2170 | 697 | 1723 | 3232 | 1200 | 77 | 330 | 406 | 0 | 76 | 40 |
| g__Klebsiella | 31 | 68 | 89 | 32 | 60 | 79 | 40 | 85 | 53 | 76 | 90 | 68 | 83 | 82 | 54 | 43 | 577 | 254 |
| g__LD21 | 12 | 14 | 20 | 9 | 30 | 29 | 20 | 7 | 8 | 13 | 16 | 13 | 1 | 5 | 8 | 0 | 5 | 4 |
| g__Lachnoclostridium_A | 6 | 12 | 7 | 6 | 24 | 11 | 0 | 15 | 14 | 11 | 7 | 13 | 2 | 10 | 6 | 0 | 0 | 0 |
| g__Lachnospira | 0 | 12 | 0 | 18 | 0 | 35 | 0 | 0 | 1 | 0 | 1 | 0 | 0 | 0 | 0 | 0 | 0 | 0 |
| g__Lachnotalea | 0 | 0 | 2 | 0 | 1 | 4 | 0 | 0 | 0 | 0 | 0 | 0 | 0 | 0 | 0 | 0 | 0 | 0 |
| g__Lactobacillus | 5364 | 1419 | 2460 | 4453 | 1594 | 1381 | 6343 | 6769 | 5342 | 8391 | 4350 | 5391 | 10710 | 1850 | 10093 | 7406 | 915 | 34937 |
| g__Lactobacillus_B | 381 | 3230 | 1307 | 215 | 4385 | 2353 | 184 | 675 | 492 | 406 | 103 | 144 | 6828 | 16355 | 677 | 20500 | 208 | 107 |
| g__Lactobacillus_E | 0 | 0 | 0 | 0 | 0 | 0 | 0 | 0 | 0 | 0 | 0 | 0 | 0 | 0 | 0 | 0 | 19 | 0 |
| g__Lactobacillus_F | 1 | 0 | 0 | 1 | 1 | 0 | 0 | 3 | 1 | 0 | 0 | 0 | 0 | 5 | 1 | 1 | 2 | 2 |
| g__Lactobacillus_G | 0 | 0 | 0 | 0 | 0 | 0 | 0 | 0 | 0 | 0 | 0 | 0 | 0 | 0 | 0 | 0 | 8 | 0 |
| g__Lactobacillus_H | 2388 | 130 | 660 | 1393 | 693 | 779 | 1117 | 2743 | 2409 | 1755 | 731 | 979 | 11350 | 7913 | 3215 | 9927 | 4104 | 11377 |
| g__Lawsonibacter | 44 | 89 | 104 | 154 | 265 | 309 | 112 | 758 | 525 | 1947 | 1569 | 821 | 26 | 84 | 305 | 1 | 50 | 21 |
| g__Lentimicrobium | 0 | 1 | 0 | 0 | 0 | 0 | 2 | 7 | 22 | 46 | 43 | 34 | 0 | 1 | 18 | 0 | 0 | 1 |
| g__Listeria | 0 | 0 | 0 | 0 | 0 | 0 | 0 | 0 | 0 | 0 | 0 | 0 | 0 | 0 | 0 | 0 | 1 | 8 |
| g__Longilinea | 1 | 0 | 1 | 0 | 1 | 0 | 0 | 0 | 2 | 1 | 1 | 0 | 1 | 2 | 0 | 0 | 5 | 0 |
| g__Luteimonas | 0 | 0 | 0 | 0 | 0 | 0 | 0 | 0 | 0 | 0 | 0 | 0 | 3 | 5 | 0 | 0 | 37 | 6 |
| g__Lysinibacillus_A | 0 | 0 | 0 | 0 | 0 | 0 | 0 | 0 | 1 | 1 | 0 | 0 | 1 | 16 | 0 | 0 | 21 | 2 |
| g__Lysinibacillus_D | 0 | 0 | 0 | 0 | 0 | 0 | 0 | 0 | 0 | 0 | 0 | 0 | 5 | 0 | 0 | 0 | 101 | 5 |
| g__MS4 | 0 | 2 | 0 | 1 | 2 | 1 | 7 | 27 | 14 | 22 | 11 | 45 | 3 | 8 | 8 | 0 | 1 | 1 |
| g__MYbin3 | 18 | 20 | 19 | 6 | 16 | 20 | 13 | 28 | 26 | 26 | 42 | 32 | 8 | 10 | 19 | 1 | 28 | 7 |
| g__Marseille-P3106 | 2 | 13 | 12 | 5 | 11 | 11 | 57 | 34 | 36 | 53 | 69 | 72 | 7 | 29 | 35 | 0 | 6 | 1 |
| g__Marvinbryantia | 10 | 32 | 14 | 3 | 7 | 12 | 3 | 9 | 1 | 0 | 15 | 1 | 1 | 0 | 2 | 0 | 1 | 1 |
| g__Massilia | 0 | 0 | 0 | 0 | 1 | 1 | 0 | 0 | 0 | 0 | 1 | 0 | 0 | 0 | 0 | 0 | 15 | 0 |
| g__Massilimaliae | 12 | 5 | 11 | 4 | 12 | 12 | 0 | 45 | 16 | 1 | 5 | 21 | 0 | 2 | 17 | 0 | 3 | 0 |
| g__Massilioclostridium | 5 | 0 | 10 | 2 | 13 | 2 | 4 | 71 | 68 | 36 | 41 | 28 | 0 | 5 | 50 | 0 | 6 | 5 |
| g__Megamonas | 40 | 131 | 204 | 10 | 883 | 654 | 2 | 0 | 1 | 1 | 1 | 1 | 3 | 1 | 1 | 3 | 0 | 2 |
| g__Megasphaera | 1 | 0 | 0 | 0 | 0 | 0 | 0 | 0 | 0 | 0 | 0 | 0 | 1 | 1 | 0 | 1 | 386 | 0 |
| g__Meiothermus_B | 7 | 6 | 10 | 5 | 8 | 5 | 7 | 10 | 11 | 13 | 9 | 10 | 4 | 2 | 9 | 5 | 14 | 6 |
| g__Mesotoga | 0 | 1 | 2 | 3 | 1 | 2 | 0 | 12 | 1 | 1 | 1 | 0 | 0 | 0 | 0 | 0 | 0 | 0 |
| g__Methanosphaera | 5 | 16 | 35 | 0 | 5 | 33 | 0 | 2 | 0 | 1 | 6 | 26 | 0 | 0 | 0 | 0 | 0 | 1 |
| g__Methanothrix | 0 | 0 | 0 | 0 | 1 | 1 | 0 | 1 | 0 | 0 | 1 | 0 | 2 | 0 | 0 | 0 | 0 | 1 |
| g__Methylocystis | 0 | 0 | 0 | 0 | 0 | 0 | 0 | 1 | 1 | 1 | 1 | 0 | 0 | 0 | 0 | 0 | 6 | 0 |
| g__Microcystis | 1 | 1 | 3 | 1 | 1 | 1 | 2 | 6 | 3 | 1 | 2 | 0 | 2 | 5 | 2 | 2 | 2 | 0 |
| g__Monoglobus | 1 | 1 | 2 | 5 | 2 | 1 | 33 | 33 | 47 | 48 | 75 | 41 | 13 | 24 | 64 | 0 | 4 | 3 |
| g__Morganella | 7 | 6 | 12 | 5 | 5 | 14 | 6 | 11 | 12 | 11 | 11 | 10 | 2 | 8 | 4 | 2 | 23 | 7 |
| g__Muribaculum | 3 | 4 | 8 | 7 | 4 | 9 | 234 | 108 | 427 | 207 | 70 | 286 | 38 | 112 | 447 | 0 | 7 | 13 |
| g__NAK82 | 4 | 4 | 1 | 2 | 9 | 8 | 1 | 3 | 8 | 4 | 8 | 7 | 2 | 6 | 3 | 3 | 4 | 1 |
| g__Negativibacillus | 6 | 35 | 10 | 0 | 0 | 7 | 0 | 4 | 6 | 2 | 0 | 1 | 0 | 0 | 9 | 0 | 0 | 0 |
| g__Nioella | 0 | 0 | 0 | 0 | 0 | 0 | 0 | 0 | 0 | 0 | 0 | 0 | 0 | 0 | 0 | 0 | 0 | 2 |
| g__Nocardiopsis | 0 | 0 | 0 | 0 | 0 | 0 | 0 | 0 | 0 | 0 | 0 | 0 | 0 | 0 | 0 | 0 | 5 | 2 |
| g__Nosocomiicoccus | 0 | 0 | 0 | 0 | 0 | 0 | 0 | 0 | 0 | 0 | 0 | 0 | 9 | 1 | 0 | 0 | 25 | 12 |
| g__OEMS01 | 134 | 439 | 134 | 42 | 229 | 220 | 86 | 90 | 151 | 80 | 201 | 121 | 18 | 40 | 246 | 0 | 23 | 17 |
| g__Oceanisphaera | 0 | 0 | 0 | 0 | 0 | 0 | 0 | 0 | 0 | 0 | 0 | 0 | 6 | 10 | 0 | 4 | 27 | 151 |
| g__Odoribacter | 3 | 5 | 4 | 0 | 5 | 4 | 7 | 13 | 3 | 19 | 15 | 10 | 1 | 2 | 2 | 0 | 1 | 0 |
| g__Oligella | 0 | 0 | 0 | 0 | 0 | 0 | 0 | 0 | 0 | 0 | 0 | 1 | 15 | 87 | 0 | 9 | 223 | 249 |
| g__Olsenella | 3 | 1 | 6 | 0 | 0 | 2 | 0 | 0 | 0 | 0 | 0 | 0 | 0 | 0 | 0 | 0 | 3 | 1 |
| g__Oscillibacter | 10 | 20 | 21 | 30 | 76 | 177 | 14 | 76 | 44 | 200 | 90 | 49 | 3 | 0 | 35 | 0 | 3 | 1 |
| g__Paenalcaligenes | 0 | 0 | 0 | 0 | 0 | 0 | 0 | 1 | 0 | 0 | 0 | 0 | 17 | 1 | 0 | 9 | 169 | 267 |
| g__Pannonibacter | 0 | 1 | 0 | 0 | 0 | 0 | 0 | 0 | 0 | 0 | 0 | 0 | 0 | 0 | 1 | 0 | 58 | 0 |
| g__Papillibacter | 16 | 12 | 16 | 1 | 8 | 11 | 22 | 5 | 8 | 7 | 12 | 11 | 2 | 4 | 2 | 0 | 3 | 2 |
| g__Parabacteroides | 42 | 76 | 80 | 69 | 137 | 48 | 9 | 4 | 7 | 8 | 18 | 31 | 3 | 9 | 15 | 0 | 58 | 0 |
| g__Paracoccus | 0 | 0 | 0 | 0 | 0 | 0 | 0 | 0 | 0 | 0 | 0 | 0 | 4 | 7 | 0 | 0 | 37 | 29 |
| g__Paramuribaculum | 2 | 3 | 5 | 6 | 6 | 4 | 60 | 28 | 81 | 63 | 13 | 35 | 5 | 13 | 129 | 0 | 6 | 1 |
| g__Paraprevotella | 0 | 0 | 1 | 7 | 2 | 1 | 0 | 0 | 0 | 0 | 0 | 0 | 0 | 0 | 0 | 0 | 0 | 0 |
| g__Parasutterella | 43 | 192 | 371 | 199 | 153 | 36 | 0 | 0 | 0 | 2 | 0 | 1 | 1 | 1 | 0 | 0 | 0 | 0 |
| g__Pectobacterium | 0 | 2 | 2 | 0 | 0 | 2 | 0 | 0 | 2 | 5 | 2 | 0 | 0 | 1 | 0 | 1 | 1 | 0 |
| g__Pediococcus | 0 | 0 | 0 | 0 | 0 | 0 | 0 | 0 | 0 | 0 | 0 | 0 | 0 | 0 | 0 | 0 | 19 | 5 |
| g__Pedobacter | 0 | 0 | 0 | 0 | 0 | 0 | 2 | 10 | 2 | 31 | 13 | 7 | 0 | 0 | 5 | 0 | 0 | 0 |
| g__Pelagibacterium | 0 | 0 | 0 | 0 | 0 | 0 | 0 | 0 | 0 | 0 | 0 | 0 | 2 | 1 | 0 | 0 | 3 | 1 |
| g__Phascolarctobacterium | 10 | 5 | 15 | 2 | 9 | 23 | 7 | 76 | 89 | 33 | 48 | 242 | 7 | 20 | 71 | 0 | 7 | 2 |
| g__Phycicoccus | 0 | 0 | 0 | 0 | 0 | 0 | 0 | 1 | 0 | 0 | 0 | 0 | 1 | 0 | 1 | 0 | 9 | 0 |
| g__Phyllobacterium | 0 | 1 | 1 | 2 | 0 | 1 | 1 | 0 | 0 | 1 | 0 | 1 | 2 | 1 | 0 | 0 | 23 | 2 |
| g__Prevotella | 1765 | 61 | 427 | 346 | 406 | 91 | 8932 | 699 | 603 | 2028 | 3558 | 4224 | 144 | 611 | 397 | 0 | 149 | 54 |
| g__Prevotellamassilia | 103 | 24 | 41 | 302 | 130 | 4 | 45 | 17 | 46 | 116 | 53 | 108 | 6 | 17 | 39 | 0 | 2 | 2 |
| g__Propionicicella | 0 | 0 | 0 | 0 | 0 | 1 | 0 | 1 | 0 | 1 | 1 | 1 | 0 | 0 | 0 | 0 | 2 | 0 |
| g__Proteus | 4 | 12 | 18 | 3 | 10 | 11 | 5 | 11 | 10 | 11 | 12 | 13 | 6 | 15 | 12 | 4 | 20 | 4 |
| g__Provencibacterium | 19 | 51 | 20 | 49 | 101 | 73 | 8 | 16 | 32 | 28 | 26 | 15 | 1 | 0 | 36 | 0 | 3 | 1 |
| g__Providencia | 24 | 40 | 47 | 14 | 34 | 41 | 20 | 47 | 52 | 45 | 60 | 44 | 34 | 24 | 34 | 17 | 77 | 23 |
| g__Pseudochrobactrum | 0 | 0 | 0 | 0 | 0 | 0 | 0 | 0 | 0 | 0 | 0 | 0 | 0 | 0 | 0 | 0 | 3 | 0 |
| g__Pseudoflavonifractor | 4 | 4 | 6 | 4 | 10 | 40 | 1 | 0 | 4 | 1 | 1 | 5 | 0 | 0 | 1 | 0 | 0 | 0 |
| g__Pseudomonas_A | 0 | 0 | 0 | 0 | 0 | 0 | 0 | 0 | 0 | 0 | 0 | 0 | 0 | 6 | 0 | 0 | 9 | 14 |
| g__Pseudomonas_E | 0 | 2 | 1 | 2 | 3 | 1 | 1 | 10 | 7 | 1 | 13 | 6 | 1 | 2 | 4 | 2 | 3876 | 2 |
| g__Psychrobacter | 0 | 0 | 0 | 0 | 1 | 0 | 8 | 1 | 0 | 2 | 0 | 0 | 2 | 14 | 0 | 3 | 30 | 21 |
| g__Pusillimonas | 0 | 0 | 0 | 0 | 0 | 0 | 0 | 0 | 0 | 0 | 0 | 0 | 3 | 6 | 0 | 2 | 24 | 23 |
| g__QAMH01 | 5 | 8 | 10 | 0 | 9 | 3 | 0 | 7 | 0 | 3 | 0 | 2 | 2 | 2 | 1 | 0 | 0 | 0 |
| g__QKVK01 | 0 | 0 | 1 | 1 | 2 | 0 | 0 | 0 | 3 | 0 | 2 | 2 | 0 | 0 | 0 | 0 | 1 | 1 |
| g__RC9 | 0 | 1 | 0 | 0 | 1 | 1 | 138 | 64 | 130 | 63 | 45 | 200 | 5 | 13 | 128 | 0 | 8 | 1 |
| g__RUG754 | 6 | 1 | 4 | 1 | 12 | 2 | 0 | 13 | 10 | 11 | 4 | 1 | 0 | 0 | 5 | 0 | 0 | 0 |
| g__Ralstonia | 0 | 0 | 0 | 0 | 0 | 0 | 0 | 0 | 0 | 0 | 1 | 0 | 0 | 6 | 0 | 0 | 9 | 3 |
| g__Raoultibacter | 11 | 9 | 3 | 10 | 7 | 8 | 8 | 18 | 15 | 7 | 9 | 9 | 1 | 18 | 16 | 0 | 0 | 0 |
| g__Reyranella | 1 | 0 | 0 | 0 | 1 | 0 | 0 | 0 | 0 | 0 | 1 | 0 | 2 | 3 | 0 | 0 | 12 | 0 |
| g__Rhizorhabdus | 3 | 4 | 2 | 5 | 2 | 5 | 2 | 6 | 4 | 2 | 5 | 3 | 1 | 3 | 2 | 3 | 4 | 1 |
| g__Rikenella | 15 | 5 | 5 | 25 | 8 | 15 | 17 | 87 | 187 | 37 | 45 | 356 | 16 | 18 | 138 | 0 | 6 | 5 |
| g__Robinsoniella | 0 | 0 | 0 | 1 | 56 | 16 | 3 | 12 | 5 | 13 | 5 | 0 | 1 | 1 | 4 | 0 | 1 | 0 |
| g__Rodentibacter | 0 | 0 | 5 | 1 | 3 | 0 | 13 | 11 | 3 | 4 | 3 | 13 | 2 | 4 | 4 | 0 | 1 | 0 |
| g__Romboutsia | 45 | 21 | 229 | 19 | 42 | 16 | 0 | 0 | 5 | 5 | 10 | 4 | 19 | 10 | 3 | 3 | 78 | 9 |
| g__Roseburia | 0 | 1 | 8 | 0 | 0 | 25 | 3 | 20 | 13 | 44 | 107 | 29 | 0 | 1 | 16 | 0 | 1 | 1 |
| g__Rothia | 28 | 16 | 39 | 15 | 34 | 24 | 3 | 31 | 14 | 20 | 16 | 21 | 2503 | 1992 | 20 | 273 | 12142 | 7471 |
| g__Ruminiclostridium | 1 | 1 | 5 | 1 | 0 | 0 | 0 | 0 | 4 | 0 | 0 | 1 | 0 | 0 | 1 | 0 | 1 | 0 |
| g__Ruminiclostridium_A | 111 | 1 | 4 | 0 | 1 | 10 | 665 | 24 | 16 | 2 | 1 | 111 | 5 | 51 | 34 | 0 | 0 | 0 |
| g__Ruminiclostridium_C | 7 | 20 | 25 | 19 | 26 | 26 | 39 | 29 | 29 | 46 | 67 | 36 | 5 | 10 | 22 | 0 | 0 | 3 |
| g__Ruminiclostridium_D | 0 | 0 | 0 | 0 | 0 | 0 | 0 | 0 | 0 | 0 | 0 | 0 | 0 | 0 | 0 | 0 | 10 | 0 |
| g__Ruminiclostridium_E | 25 | 44 | 138 | 6 | 370 | 113 | 74 | 384 | 698 | 56 | 1082 | 248 | 4 | 7 | 681 | 0 | 44 | 15 |
| g__Ruminococcus | 4 | 2 | 7 | 0 | 14 | 3 | 38 | 15 | 35 | 92 | 352 | 225 | 2 | 1 | 37 | 0 | 6 | 3 |
| g__Ruminococcus_A | 2 | 1015 | 160 | 6 | 77 | 26 | 5 | 10 | 10 | 36 | 45 | 22 | 1 | 3 | 14 | 0 | 1 | 1 |
| g__Ruminococcus_C | 170 | 35 | 57 | 10 | 35 | 13 | 87 | 88 | 57 | 22 | 87 | 271 | 4 | 12 | 56 | 0 | 7 | 1 |
| g__Ruminococcus_D | 75 | 19 | 6 | 3 | 3 | 107 | 6 | 22 | 67 | 11 | 77 | 109 | 0 | 2 | 71 | 0 | 2 | 3 |
| g__Ruthenibacterium | 11 | 23 | 9 | 9 | 5 | 113 | 2 | 52 | 16 | 14 | 8 | 13 | 0 | 2 | 13 | 0 | 2 | 2 |
| g__SK-Y3 | 3 | 0 | 1 | 2 | 5 | 7 | 5 | 4 | 12 | 1 | 7 | 5 | 2 | 2 | 7 | 0 | 1 | 0 |
| g__SR-FBR-E99 | 3 | 3 | 5 | 2 | 5 | 5 | 2 | 0 | 4 | 2 | 3 | 2 | 1 | 1 | 1 | 1 | 0 | 0 |
| g__SZUA-55 | 0 | 0 | 0 | 0 | 0 | 0 | 0 | 0 | 0 | 0 | 0 | 0 | 8 | 1 | 0 | 0 | 1 | 6 |
| g__Saccharimonas | 171 | 22 | 81 | 38 | 37 | 78 | 720 | 1100 | 1508 | 625 | 1043 | 1290 | 155 | 543 | 1300 | 9 | 87 | 72 |
| g__Salmonella | 2307 | 765 | 1294 | 663 | 1872 | 2016 | 944 | 1109 | 1448 | 1331 | 1271 | 1378 | 96 | 317 | 1143 | 9 | 181 | 82 |
| g__Savagella | 4 | 0 | 4 | 0 | 0 | 0 | 0 | 1 | 0 | 1 | 0 | 0 | 0 | 0 | 1 | 0 | 0 | 0 |
| g__Schwartzia | 1 | 4 | 0 | 1 | 2 | 4 | 341 | 2197 | 617 | 1461 | 1392 | 565 | 76 | 234 | 316 | 3 | 54 | 20 |
| g__Serinibacter | 3 | 1 | 0 | 1 | 1 | 1 | 0 | 2 | 3 | 1 | 5 | 4 | 1 | 0 | 2 | 2 | 3 | 2 |
| g__Solobacterium | 0 | 0 | 0 | 0 | 0 | 0 | 0 | 0 | 0 | 0 | 0 | 0 | 0 | 0 | 0 | 0 | 8 | 0 |
| g__Sphingobacterium | 1 | 4 | 5 | 3 | 8 | 6 | 24 | 10 | 33 | 5 | 9 | 12 | 2 | 22 | 23 | 2 | 48 | 66 |
| g__Sphingomonas_A | 9 | 12 | 9 | 8 | 8 | 21 | 8 | 21 | 11 | 23 | 23 | 22 | 4 | 12 | 13 | 3 | 30 | 8 |
| g__Sphingomonas_B | 3 | 3 | 2 | 1 | 0 | 2 | 1 | 1 | 2 | 5 | 4 | 3 | 1 | 1 | 2 | 1 | 3 | 0 |
| g__Sphingopyxis | 0 | 1 | 0 | 0 | 0 | 0 | 0 | 0 | 0 | 0 | 0 | 0 | 0 | 0 | 0 | 0 | 94 | 0 |
| g__Sporobacter | 9 | 22 | 10 | 3 | 12 | 13 | 14 | 23 | 19 | 11 | 13 | 22 | 2 | 7 | 23 | 0 | 1 | 2 |
| g__Sporolactobacillus | 0 | 0 | 0 | 0 | 0 | 0 | 0 | 0 | 0 | 0 | 1 | 0 | 0 | 0 | 0 | 0 | 17 | 0 |
| g__Staphylococcus | 8 | 3 | 3 | 0 | 5 | 0 | 0 | 0 | 0 | 0 | 0 | 0 | 5 | 0 | 0 | 0 | 15 | 1 |
| g__Staphylococcus_A | 1 | 0 | 0 | 1 | 0 | 3 | 0 | 0 | 0 | 0 | 0 | 0 | 38 | 5 | 0 | 1 | 88 | 10 |
| g__Steroidobacter | 0 | 1 | 2 | 0 | 2 | 0 | 0 | 1 | 0 | 0 | 1 | 3 | 0 | 2 | 1 | 0 | 0 | 0 |
| g__Stoquefichus | 15 | 7 | 6 | 1 | 10 | 24 | 0 | 1 | 0 | 0 | 0 | 0 | 0 | 0 | 0 | 0 | 0 | 0 |
| g__Streptococcus | 7 | 2 | 12 | 0 | 1 | 2 | 174 | 42 | 103 | 59 | 61 | 99 | 434 | 159 | 175 | 65 | 1059 | 580 |
| g__Sutterella | 37 | 30 | 45 | 3 | 4 | 52 | 76 | 289 | 78 | 40 | 18 | 211 | 5 | 8 | 62 | 0 | 5 | 4 |
| g__Symbiobacterium | 0 | 3 | 1 | 3 | 1 | 2 | 2 | 4 | 1 | 2 | 1 | 2 | 0 | 0 | 1 | 2 | 3 | 1 |
| g__Syntrophobacter | 2 | 6 | 3 | 2 | 2 | 5 | 4 | 3 | 1 | 4 | 6 | 5 | 2 | 5 | 2 | 2 | 10 | 1 |
| g__T78 | 4 | 1 | 0 | 1 | 0 | 0 | 0 | 2 | 0 | 4 | 1 | 0 | 1 | 1 | 1 | 0 | 4 | 2 |
| g__TF01-11 | 1 | 9 | 2 | 3 | 10 | 18 | 5 | 24 | 60 | 28 | 69 | 25 | 3 | 3 | 29 | 0 | 3 | 2 |
| g__TWA4 | 2 | 25 | 6 | 5 | 9 | 7 | 0 | 9 | 5 | 9 | 10 | 2 | 0 | 0 | 4 | 0 | 1 | 0 |
| g__Tatlockia | 0 | 0 | 0 | 0 | 1 | 0 | 0 | 0 | 0 | 0 | 0 | 1 | 1 | 0 | 0 | 0 | 236 | 1 |
| g__Thermoflavifilum | 1 | 0 | 5 | 3 | 3 | 0 | 1 | 2 | 0 | 3 | 1 | 1 | 0 | 1 | 0 | 0 | 1 | 1 |
| g__Thioalkalivibrio_A | 0 | 1 | 1 | 2 | 4 | 6 | 2 | 7 | 4 | 2 | 4 | 2 | 0 | 0 | 1 | 2 | 3 | 0 |
| g__Thiobacillus | 1 | 0 | 0 | 1 | 1 | 0 | 0 | 0 | 0 | 0 | 0 | 2 | 0 | 0 | 1 | 0 | 0 | 2 |
| g__Tissierella_A | 0 | 0 | 0 | 0 | 1 | 0 | 0 | 0 | 0 | 0 | 0 | 0 | 0 | 1 | 0 | 0 | 0 | 1 |
| g__Tolypothrix_B | 0 | 0 | 0 | 0 | 1 | 3 | 0 | 1 | 2 | 2 | 0 | 1 | 1 | 0 | 1 | 0 | 1 | 0 |
| g__Treponema_D | 5 | 9 | 22 | 16 | 598 | 1157 | 110 | 845 | 2983 | 1517 | 655 | 1359 | 15 | 45 | 1202 | 1 | 22 | 8 |
| g__Tyzzerella | 4 | 1 | 8 | 1 | 15 | 4 | 9 | 2 | 29 | 13 | 18 | 38 | 2 | 7 | 55 | 0 | 4 | 0 |
| g__UBA1033 | 2 | 3 | 1 | 2 | 4 | 1 | 0 | 0 | 0 | 0 | 0 | 0 | 0 | 0 | 0 | 0 | 0 | 0 |
| g__UBA1174 | 0 | 0 | 1 | 4 | 1 | 1 | 0 | 0 | 0 | 1 | 0 | 1 | 0 | 0 | 0 | 0 | 0 | 0 |
| g__UBA1191 | 3 | 9 | 8 | 0 | 8 | 16 | 4 | 11 | 21 | 11 | 12 | 18 | 12 | 13 | 10 | 0 | 2 | 3 |
| g__UBA12465 | 0 | 0 | 0 | 0 | 0 | 1 | 3 | 1 | 0 | 0 | 0 | 1 | 0 | 0 | 0 | 0 | 0 | 1 |
| g__UBA1394 | 41 | 90 | 1 | 0 | 2 | 0 | 11 | 47 | 76 | 51 | 16 | 64 | 1 | 0 | 127 | 0 | 4 | 2 |
| g__UBA1436 | 1 | 0 | 1 | 5 | 0 | 8 | 2 | 0 | 0 | 0 | 0 | 5 | 1 | 0 | 1 | 0 | 2 | 0 |
| g__UBA1547 | 0 | 0 | 0 | 0 | 0 | 0 | 141 | 138 | 84 | 23 | 121 | 148 | 19 | 80 | 66 | 1 | 5 | 6 |
| g__UBA1711 | 0 | 0 | 1 | 0 | 0 | 0 | 92 | 16 | 15 | 63 | 44 | 24 | 3 | 5 | 25 | 0 | 1 | 0 |
| g__UBA1777 | 0 | 1 | 1 | 0 | 1 | 0 | 96 | 61 | 41 | 69 | 67 | 54 | 7 | 18 | 46 | 0 | 1 | 0 |
| g__UBA2212 | 22 | 12 | 53 | 15 | 2 | 14 | 4 | 2 | 6 | 2 | 0 | 3 | 0 | 3 | 4 | 0 | 0 | 1 |
| g__UBA2365 | 0 | 0 | 0 | 0 | 0 | 0 | 0 | 0 | 0 | 0 | 0 | 0 | 0 | 0 | 0 | 0 | 12 | 0 |
| g__UBA2730 | 53 | 30 | 78 | 4 | 3 | 163 | 113 | 78 | 117 | 6 | 0 | 239 | 23 | 139 | 82 | 0 | 4 | 3 |
| g__UBA3006 | 2 | 2 | 1 | 1 | 2 | 2 | 2 | 3 | 1 | 1 | 2 | 2 | 8 | 2 | 0 | 1 | 7 | 2 |
| g__UBA4782 | 1 | 7 | 5 | 3 | 3 | 2 | 1 | 4 | 6 | 4 | 5 | 4 | 3 | 6 | 1 | 6 | 4 | 2 |
| g__UBA6382 | 0 | 0 | 0 | 0 | 0 | 0 | 0 | 0 | 0 | 0 | 0 | 0 | 0 | 0 | 0 | 0 | 24 | 0 |
| g__UBA6398 | 0 | 0 | 0 | 0 | 13 | 2 | 0 | 0 | 0 | 0 | 0 | 0 | 0 | 0 | 0 | 0 | 0 | 0 |
| g__UBA6659 | 1 | 1 | 2 | 0 | 2 | 1 | 0 | 1 | 0 | 0 | 1 | 0 | 1 | 0 | 0 | 0 | 0 | 1 |
| g__UBA6985 | 44 | 0 | 101 | 0 | 2 | 3 | 115 | 33 | 56 | 58 | 10 | 55 | 4 | 11 | 46 | 2 | 0 | 1 |
| g__UBA7182 | 0 | 0 | 0 | 0 | 0 | 0 | 0 | 0 | 1 | 1 | 0 | 1 | 0 | 0 | 0 | 0 | 0 | 0 |
| g__UBA8950 | 1 | 0 | 1 | 0 | 0 | 1 | 0 | 0 | 0 | 2 | 1 | 3 | 1 | 1 | 0 | 1 | 0 | 0 |
| g__UBA945 | 0 | 0 | 0 | 0 | 0 | 0 | 0 | 3 | 13 | 1 | 8 | 21 | 0 | 3 | 8 | 0 | 2 | 0 |
| g__UC5-1-2E3 | 3 | 15 | 8 | 9 | 120 | 144 | 3 | 21 | 29 | 56 | 34 | 18 | 0 | 12 | 15 | 0 | 4 | 5 |
| g__URHD0088 | 0 | 0 | 0 | 1 | 0 | 0 | 0 | 1 | 0 | 2 | 0 | 2 | 0 | 1 | 0 | 0 | 0 | 0 |
| g__UTCFX2 | 3 | 4 | 3 | 2 | 1 | 4 | 0 | 4 | 3 | 1 | 7 | 6 | 4 | 7 | 3 | 3 | 6 | 3 |
| g__Vagococcus | 7 | 1 | 0 | 0 | 4 | 1 | 4 | 1 | 2 | 0 | 1 | 1 | 5 | 3 | 1 | 6 | 3 | 7 |
| g__Vallitalea | 127 | 93 | 199 | 2 | 71 | 105 | 27 | 3 | 2 | 0 | 2 | 19 | 6 | 14 | 0 | 0 | 1 | 1 |
| g__Vallitalea_A | 5 | 14 | 2 | 3 | 7 | 1 | 0 | 0 | 0 | 1 | 0 | 0 | 0 | 0 | 0 | 0 | 0 | 0 |
| g__Veillonella | 0 | 0 | 0 | 0 | 0 | 0 | 4 | 0 | 1 | 2 | 0 | 4 | 0 | 1 | 0 | 0 | 0 | 0 |
| g__Vibrio | 10022 | 5347 | 10250 | 8790 | 8905 | 12466 | 4103 | 1338 | 1759 | 1563 | 2173 | 1783 | 167 | 432 | 1955 | 3 | 99 | 65 |
| g__Virgibacillus | 0 | 0 | 0 | 0 | 0 | 0 | 0 | 0 | 0 | 0 | 0 | 0 | 8 | 26 | 0 | 1 | 65 | 30 |
| g__Virgibacillus_G | 3 | 33 | 33 | 21 | 616 | 177 | 4 | 130 | 22 | 156 | 47 | 17 | 1 | 4 | 23 | 0 | 3 | 2 |
| g__W-Firmicutes-11 | 7 | 27 | 17 | 13 | 17 | 20 | 14 | 39 | 27 | 31 | 23 | 29 | 2 | 8 | 23 | 0 | 2 | 3 |
| g__Weissella | 6 | 6 | 4 | 5 | 3 | 2 | 6 | 5 | 9 | 10 | 3 | 9 | 19 | 13 | 6 | 18 | 12 | 8 |
| g__Yaniella | 0 | 0 | 0 | 0 | 0 | 0 | 0 | 0 | 0 | 0 | 0 | 1 | 3 | 0 | 0 | 0 | 17 | 11 |
| g__ZCTH02-B6 | 0 | 0 | 0 | 0 | 0 | 0 | 0 | 0 | 0 | 0 | 0 | 0 | 1 | 0 | 1 | 0 | 0 | 0 |
| g__Zag1 | 0 | 1 | 0 | 0 | 1 | 0 | 68 | 24 | 5 | 9 | 25 | 26 | 1 | 17 | 20 | 0 | 3 | 0 |
| g__Zag111 | 0 | 0 | 0 | 0 | 0 | 1 | 1 | 2 | 1 | 3 | 4 | 1 | 0 | 2 | 3 | 0 | 0 | 0 |
| g__unclassified | 2 | 0 | 0 | 0 | 14 | 0 | 0 | 2 | 2 | 2 | 2 | 1 | 4 | 0 | 1 | 4 | 7 | 4 |

## Species count for Fig 6D

| species | WT1 | WT2 | WT3 | WT4 | WT5 | WT6 | Uox-/- -1-1 | Uox-/- -1-2 | Uox-/- -1-3 | Uox-/- -1-4 | Uox-/- -1-5 | Uox-/- -1-6 | Uox-/- -2-1 | Uox-/- -2-2 | Uox-/- -2-3 | Uox-/- -2-4 | Uox-/- -2-5 | Uox-/- -2-6 |
| --- | --- | --- | --- | --- | --- | --- | --- | --- | --- | --- | --- | --- | --- | --- | --- | --- | --- | --- |
| s__14-2_sp000403255 | 3 | 11 | 7 | 3 | 411 | 10 | 2 | 9 | 10 | 158 | 122 | 31 | 0 | 6 | 9 | 0 | 4 | 1 |
| s__14-2_sp000403315 | 0 | 6 | 0 | 1 | 0 | 2 | 0 | 29 | 0 | 2 | 1 | 1 | 1 | 0 | 1 | 0 | 1 | 0 |
| s__14-2_sp000403845 | 0 | 0 | 0 | 0 | 0 | 1 | 1 | 0 | 6 | 0 | 20 | 0 | 0 | 1 | 3 | 0 | 1 | 0 |
| s__14-2_sp001940225 | 10 | 13 | 23 | 6 | 2 | 16 | 2 | 26 | 5 | 12 | 13 | 12 | 0 | 1 | 6 | 0 | 4 | 0 |
| s__21-14-0-10-47-8-A_sp002793885 | 0 | 0 | 0 | 0 | 0 | 0 | 0 | 0 | 0 | 0 | 0 | 0 | 0 | 0 | 0 | 0 | 6 | 0 |
| s__33-17_sp001897445 | 0 | 0 | 0 | 0 | 0 | 0 | 0 | 0 | 0 | 0 | 1 | 3 | 1 | 1 | 0 | 0 | 0 | 0 |
| s__49-20_sp002436085 | 4 | 7 | 4 | 2 | 6 | 7 | 5 | 5 | 7 | 9 | 10 | 4 | 1 | 9 | 3 | 8 | 5 | 4 |
| s__992a_sp001940245 | 140 | 62 | 34 | 44 | 1396 | 1370 | 141 | 406 | 145 | 256 | 681 | 243 | 14 | 14 | 118 | 1 | 16 | 8 |
| s__ASF356_sp000364165 | 0 | 6 | 0 | 0 | 5 | 15 | 0 | 0 | 3 | 0 | 0 | 0 | 0 | 0 | 3 | 0 | 0 | 1 |
| s__Absiella_dolichum | 68 | 1 | 8 | 0 | 3 | 1 | 7 | 5 | 23 | 8 | 12 | 12 | 8 | 8 | 17 | 0 | 2 | 1 |
| s__Acetatifactor_muris | 0 | 0 | 0 | 0 | 0 | 0 | 0 | 4 | 1 | 1 | 0 | 0 | 0 | 0 | 0 | 0 | 0 | 0 |
| s__Acetitomaculum_ruminis | 0 | 3 | 0 | 3 | 14 | 0 | 2 | 39 | 11 | 2 | 21 | 67 | 1 | 11 | 10 | 0 | 0 | 0 |
| s__Acetivibrio_cellulolyticus | 7 | 14 | 10 | 4 | 1 | 4 | 1 | 1 | 1 | 1 | 0 | 2 | 0 | 1 | 1 | 0 | 0 | 0 |
| s__Acetoanaerobium_sticklandii | 0 | 3 | 0 | 1 | 0 | 1 | 0 | 0 | 1 | 2 | 0 | 0 | 0 | 0 | 0 | 0 | 0 | 0 |
| s__Acetobacter_papayae | 0 | 0 | 0 | 0 | 0 | 0 | 0 | 0 | 0 | 0 | 0 | 0 | 0 | 0 | 0 | 0 | 16 | 0 |
| s__Achromobacter_sp000757485 | 0 | 0 | 0 | 0 | 0 | 0 | 0 | 0 | 0 | 0 | 0 | 0 | 0 | 0 | 0 | 0 | 13 | 0 |
| s__Acinetobacter_kookii | 0 | 0 | 1 | 0 | 2 | 0 | 0 | 0 | 0 | 0 | 0 | 0 | 1 | 0 | 1 | 0 | 62 | 1 |
| s__Acinetobacter_radioresistens | 0 | 0 | 0 | 0 | 0 | 1 | 0 | 0 | 0 | 0 | 1 | 1 | 24 | 13 | 1 | 0 | 202 | 11 |
| s__Acinetobacter_sp000313935 | 0 | 1 | 2 | 0 | 2 | 2 | 5 | 0 | 1 | 0 | 0 | 3 | 0 | 0 | 1 | 0 | 6 | 1 |
| s__Actinomadura_B_rubrobrunea | 1 | 1 | 1 | 1 | 1 | 0 | 0 | 1 | 2 | 0 | 0 | 0 | 2 | 0 | 1 | 0 | 0 | 0 |
| s__Actinomyces_I_minihominis | 1 | 2 | 1 | 0 | 0 | 4 | 1 | 4 | 1 | 0 | 1 | 2 | 1 | 1 | 0 | 1 | 7 | 1 |
| s__Actinomyces_bovis | 0 | 1 | 1 | 0 | 0 | 1 | 3 | 3 | 0 | 2 | 1 | 2 | 12 | 39 | 1 | 38 | 552 | 152 |
| s__Actinomyces_urogenitalis | 0 | 0 | 0 | 0 | 0 | 0 | 0 | 0 | 0 | 0 | 0 | 0 | 34 | 18 | 0 | 5 | 95 | 127 |
| s__Acutalibacter_muris | 13 | 4 | 14 | 9 | 40 | 21 | 13 | 62 | 31 | 68 | 79 | 24 | 4 | 3 | 28 | 0 | 4 | 3 |
| s__Acutalibacter_timonensis | 17 | 16 | 31 | 32 | 30 | 73 | 12 | 181 | 98 | 166 | 153 | 414 | 37 | 140 | 94 | 0 | 9 | 2 |
| s__Adlercreutzia_caecimuris | 10 | 28 | 22 | 5 | 6 | 6 | 2 | 5 | 5 | 0 | 0 | 2 | 1 | 4 | 4 | 0 | 2 | 0 |
| s__Adlercreutzia_equolifaciens | 43 | 48 | 95 | 38 | 21 | 54 | 4 | 23 | 11 | 5 | 8 | 7 | 2 | 5 | 8 | 0 | 2 | 5 |
| s__Advenella_kashmirensis | 0 | 0 | 0 | 0 | 0 | 0 | 0 | 0 | 0 | 0 | 0 | 0 | 0 | 0 | 0 | 0 | 297 | 0 |
| s__Agathobacter_ruminis | 32 | 12 | 179 | 2 | 8 | 361 | 12 | 582 | 171 | 631 | 473 | 205 | 3 | 3 | 116 | 0 | 8 | 6 |
| s__Agathobaculum_sp900291975 | 1 | 4 | 4 | 2 | 3 | 12 | 2 | 4 | 6 | 4 | 7 | 2 | 0 | 1 | 1 | 0 | 0 | 0 |
| s__Agrobacterium_nepotum | 0 | 1 | 1 | 0 | 1 | 0 | 1 | 2 | 3 | 2 | 4 | 2 | 3 | 4 | 1 | 1 | 1037 | 4 |
| s__Alicyclobacillus_B_ferrooxydans | 0 | 1 | 0 | 0 | 0 | 1 | 1 | 1 | 1 | 0 | 0 | 1 | 0 | 1 | 1 | 1 | 0 | 0 |
| s__Alicyclobacillus_H_macrosporangiidus | 0 | 1 | 0 | 0 | 0 | 0 | 0 | 2 | 0 | 0 | 0 | 1 | 1 | 0 | 0 | 1 | 0 | 0 |
| s__Alistipes_A_sp900240235 | 0 | 6 | 0 | 4 | 29 | 2 | 0 | 0 | 0 | 0 | 1 | 0 | 0 | 0 | 0 | 0 | 0 | 0 |
| s__Alistipes_putredinis | 1 | 4 | 12 | 11 | 33 | 9 | 9 | 0 | 16 | 5 | 4 | 0 | 1 | 0 | 13 | 0 | 0 | 2 |
| s__Alistipes_shahii | 0 | 0 | 3 | 0 | 0 | 3 | 0 | 0 | 0 | 0 | 0 | 0 | 0 | 0 | 0 | 0 | 0 | 0 |
| s__Alistipes_timonensis | 0 | 0 | 4 | 2 | 14 | 0 | 2 | 0 | 0 | 0 | 0 | 0 | 0 | 0 | 0 | 0 | 0 | 0 |
| s__Allobaculum_stercoricanis | 7 | 18 | 66 | 0 | 1 | 3 | 0 | 0 | 0 | 0 | 0 | 0 | 0 | 0 | 0 | 0 | 0 | 0 |
| s__Aminicenans_sakinawicola | 0 | 1 | 0 | 0 | 0 | 1 | 0 | 0 | 1 | 0 | 2 | 2 | 0 | 1 | 0 | 0 | 0 | 0 |
| s__An181_sp002160325 | 9 | 148 | 23 | 1 | 19 | 0 | 0 | 0 | 0 | 0 | 0 | 0 | 0 | 0 | 0 | 0 | 0 | 0 |
| s__Anaerobiospirillum_A_thomasii | 0 | 2 | 2 | 0 | 4 | 2 | 2 | 750 | 338 | 582 | 1203 | 765 | 6 | 6 | 66 | 1 | 8 | 2 |
| s__Anaerocolumna_jejuensis | 0 | 0 | 0 | 1 | 0 | 0 | 0 | 0 | 6 | 5 | 0 | 0 | 0 | 0 | 4 | 0 | 1 | 0 |
| s__Anaerofustis_stercorihominis | 5 | 0 | 3 | 2 | 0 | 1 | 1 | 3 | 0 | 0 | 5 | 0 | 0 | 0 | 1 | 0 | 0 | 0 |
| s__Anaeromassilibacillus_senegalensis | 45 | 49 | 53 | 26 | 20 | 76 | 0 | 0 | 0 | 0 | 0 | 0 | 0 | 0 | 0 | 0 | 0 | 0 |
| s__Anaerostipes_hadrus | 72 | 229 | 370 | 7 | 3 | 312 | 68 | 27 | 37 | 17 | 29 | 202 | 9 | 72 | 43 | 1 | 10 | 2 |
| s__Anaerostipes_sp000508985 | 1 | 2 | 1 | 0 | 3 | 14 | 3 | 79 | 46 | 30 | 111 | 33 | 3 | 6 | 27 | 0 | 6 | 9 |
| s__Anaerostipes_sp001940315 | 1 | 0 | 0 | 0 | 0 | 0 | 86 | 0 | 0 | 44 | 13 | 0 | 0 | 0 | 0 | 0 | 2 | 3 |
| s__Anaerotignum_lactatifermentans | 1 | 7 | 2 | 3 | 2 | 1 | 5 | 53 | 20 | 34 | 67 | 13 | 0 | 1 | 16 | 0 | 3 | 1 |
| s__Anaerotruncus_colihominis | 1 | 5 | 4 | 5 | 75 | 23 | 2 | 2 | 1 | 0 | 1 | 6 | 0 | 0 | 3 | 0 | 0 | 1 |
| s__Anaerotruncus_rubiinfantis | 1 | 3 | 1 | 2 | 2 | 2 | 1 | 3 | 1 | 2 | 2 | 2 | 0 | 0 | 2 | 0 | 0 | 0 |
| s__Anaerotruncus_sp000403395 | 3 | 18 | 8 | 45 | 43 | 71 | 0 | 1 | 1 | 0 | 1 | 0 | 0 | 0 | 0 | 0 | 0 | 0 |
| s__Anaerovibrio_sp002100115 | 0 | 0 | 0 | 0 | 0 | 0 | 1 | 11 | 32 | 1 | 0 | 1 | 0 | 0 | 9 | 0 | 0 | 0 |
| s__Angelakisella_massiliensis | 5 | 2 | 2 | 6 | 13 | 12 | 2 | 7 | 9 | 8 | 10 | 6 | 0 | 1 | 4 | 0 | 0 | 0 |
| s__Aquamicrobium_A_aerolatum | 0 | 0 | 2 | 1 | 0 | 0 | 1 | 1 | 0 | 0 | 1 | 0 | 15 | 17 | 0 | 3 | 63 | 34 |
| s__Arboricoccus_pini | 0 | 0 | 0 | 0 | 0 | 0 | 0 | 1 | 2 | 0 | 0 | 1 | 0 | 0 | 0 | 0 | 1 | 0 |
| s__Atopostipes_suicloacalis | 0 | 0 | 0 | 0 | 0 | 0 | 0 | 0 | 0 | 0 | 0 | 0 | 0 | 0 | 0 | 0 | 11 | 1 |
| s__Bacillus_A_sp002571225 | 0 | 0 | 0 | 0 | 0 | 0 | 0 | 0 | 0 | 0 | 0 | 0 | 0 | 0 | 0 | 1 | 66 | 2 |
| s__Bacillus_C_aryabhattai | 1 | 0 | 0 | 0 | 0 | 1 | 0 | 0 | 1 | 1 | 0 | 0 | 3 | 4 | 3 | 2 | 27 | 10 |
| s__Bacillus_J_thermoamylovorans | 0 | 0 | 0 | 0 | 0 | 0 | 0 | 0 | 0 | 0 | 0 | 0 | 1 | 4 | 0 | 1 | 28 | 3 |
| s__Bacillus_W_sp002559145 | 5 | 1 | 1 | 1 | 0 | 0 | 0 | 0 | 1 | 1 | 1 | 1 | 3 | 1 | 0 | 4 | 4 | 3 |
| s__Bacillus_haynesii | 0 | 1 | 0 | 1 | 0 | 0 | 0 | 0 | 0 | 0 | 0 | 0 | 7 | 5 | 0 | 0 | 71 | 19 |
| s__Bact-08_sp001897515 | 0 | 0 | 0 | 0 | 0 | 0 | 7 | 0 | 3 | 0 | 9 | 9 | 0 | 3 | 0 | 0 | 0 | 0 |
| s__Bacteroides_B_dorei | 9 | 14 | 4 | 5 | 348 | 9 | 1 | 0 | 0 | 0 | 0 | 1 | 0 | 0 | 0 | 0 | 0 | 0 |
| s__Bacteroides_B_sartorii | 150 | 198 | 265 | 30 | 284 | 79 | 67 | 7 | 8 | 14 | 23 | 37 | 3 | 7 | 22 | 0 | 1 | 1 |
| s__Bacteroides_F_pectinophilus | 76 | 27 | 1 | 8 | 1 | 408 | 0 | 52 | 202 | 4 | 5 | 427 | 22 | 58 | 129 | 0 | 10 | 1 |
| s__Bacteroides_acidifaciens | 164 | 271 | 263 | 458 | 326 | 131 | 3 | 0 | 1 | 3 | 2 | 2 | 0 | 1 | 1 | 0 | 2 | 1 |
| s__Bacteroides_cutis | 0 | 0 | 0 | 0 | 0 | 0 | 46 | 4 | 17 | 14 | 0 | 62 | 13 | 27 | 10 | 0 | 2 | 0 |
| s__Bacteroides_eggerthii | 1 | 34 | 8 | 11 | 40 | 3 | 0 | 0 | 0 | 0 | 1 | 0 | 0 | 0 | 1 | 0 | 0 | 0 |
| s__Bacteroides_gallinarum | 0 | 0 | 0 | 0 | 0 | 0 | 0 | 0 | 0 | 1 | 2 | 0 | 0 | 0 | 4 | 0 | 0 | 0 |
| s__Bacteroides_intestinalis | 5 | 19 | 6 | 4 | 21 | 2 | 116 | 45 | 34 | 61 | 29 | 85 | 5 | 10 | 21 | 0 | 7 | 3 |
| s__Bacteroides_oleiciplenus | 1 | 0 | 1 | 2 | 5 | 2 | 0 | 0 | 0 | 0 | 0 | 0 | 0 | 0 | 0 | 0 | 0 | 0 |
| s__Bacteroides_uniformis | 6 | 9 | 8 | 10 | 28 | 1 | 1 | 0 | 0 | 1 | 0 | 0 | 0 | 1 | 0 | 0 | 0 | 0 |
| s__Beduini_massiliensis | 7 | 1 | 1 | 8 | 1 | 0 | 12 | 6 | 3 | 6 | 0 | 18 | 2 | 9 | 9 | 0 | 0 | 1 |
| s__Beta-01_sp001603715 | 0 | 0 | 0 | 0 | 0 | 0 | 0 | 0 | 0 | 0 | 0 | 0 | 1 | 7 | 0 | 0 | 1 | 6 |
| s__Bifidobacterium_animalis | 0 | 1 | 12 | 0 | 8 | 1 | 0 | 0 | 0 | 0 | 0 | 0 | 0 | 0 | 0 | 1 | 1 | 1 |
| s__Bifidobacterium_asteroides | 8 | 9 | 15 | 10 | 19 | 9 | 10 | 21 | 12 | 13 | 11 | 18 | 30 | 24 | 15 | 38 | 16 | 26 |
| s__Bilophila_wadsworthia | 0 | 0 | 0 | 3 | 32 | 4 | 1 | 0 | 2 | 10 | 8 | 10 | 0 | 1 | 1 | 0 | 1 | 0 |
| s__Bittarella_massiliensis | 6 | 13 | 3 | 3 | 9 | 11 | 0 | 0 | 0 | 0 | 0 | 0 | 0 | 0 | 1 | 0 | 0 | 0 |
| s__Blastococcus_sp003075095 | 6 | 55 | 1515 | 8 | 7 | 10 | 1 | 1 | 0 | 0 | 3 | 1 | 0 | 0 | 1 | 0 | 2 | 0 |
| s__Blautia_A_sp900066165 | 56 | 846 | 303 | 33 | 119 | 57 | 5 | 6 | 8 | 5 | 6 | 5 | 1 | 1 | 10 | 0 | 1 | 0 |
| s__Blautia_A_sp900066205 | 0 | 5 | 5 | 0 | 1 | 0 | 0 | 6 | 5 | 2 | 0 | 5 | 3 | 11 | 1 | 0 | 1 | 1 |
| s__Blautia_A_sp900120195 | 0 | 0 | 5 | 6 | 0 | 0 | 0 | 1 | 1 | 0 | 0 | 0 | 0 | 0 | 1 | 0 | 0 | 0 |
| s__Bog-159_sp003166475 | 0 | 1 | 0 | 0 | 0 | 0 | 0 | 0 | 0 | 0 | 0 | 0 | 0 | 0 | 1 | 0 | 0 | 0 |
| s__Bosea_sp001748145 | 0 | 0 | 0 | 0 | 0 | 0 | 0 | 0 | 0 | 0 | 0 | 0 | 0 | 1 | 0 | 0 | 18 | 5 |
| s__Brachybacterium_massiliense | 0 | 0 | 0 | 0 | 0 | 0 | 0 | 1 | 0 | 1 | 0 | 0 | 10 | 10 | 0 | 0 | 46 | 16 |
| s__Brachyspira_hampsonii_B | 0 | 0 | 0 | 0 | 0 | 0 | 0 | 0 | 0 | 0 | 0 | 0 | 0 | 0 | 0 | 0 | 10 | 0 |
| s__Bradyrhizobium_sp000617845 | 0 | 1 | 1 | 0 | 0 | 0 | 0 | 0 | 1 | 0 | 2 | 0 | 4 | 3 | 0 | 0 | 13 | 8 |
| s__Brevibacillus_panacihumi | 0 | 0 | 0 | 0 | 0 | 0 | 0 | 0 | 1 | 0 | 0 | 0 | 2 | 2 | 0 | 0 | 6 | 1 |
| s__Brevibacterium_senegalense | 0 | 1 | 0 | 0 | 0 | 0 | 0 | 0 | 0 | 0 | 0 | 0 | 11 | 5 | 0 | 0 | 64 | 17 |
| s__Brevundimonas_diminuta_B | 0 | 0 | 0 | 0 | 0 | 0 | 0 | 0 | 0 | 0 | 0 | 0 | 15 | 2 | 0 | 0 | 17 | 7 |
| s__Butyricicoccus_A_porcorum | 0 | 0 | 1 | 1 | 0 | 0 | 1 | 0 | 1 | 0 | 2 | 3 | 1 | 1 | 1 | 0 | 0 | 0 |
| s__Butyricicoccus_pullicaecorum | 0 | 5 | 2 | 1 | 0 | 14 | 0 | 15 | 1 | 24 | 24 | 5 | 2 | 2 | 5 | 0 | 1 | 0 |
| s__Butyricimonas_synergistica | 2 | 8 | 6 | 2 | 8 | 6 | 0 | 0 | 0 | 1 | 1 | 0 | 0 | 0 | 0 | 0 | 0 | 0 |
| s__Butyrivibrio_A_crossotus | 2 | 14 | 13 | 3 | 10 | 137 | 5 | 505 | 1538 | 23 | 961 | 862 | 23 | 53 | 939 | 2 | 49 | 31 |
| s__Butyrivibrio_sp900112195 | 1 | 0 | 0 | 0 | 0 | 6 | 0 | 3 | 0 | 0 | 1 | 0 | 0 | 0 | 0 | 0 | 0 | 0 |
| s__CAG-1031_sp000431215 | 50 | 21 | 49 | 34 | 62 | 76 | 8 | 2 | 11 | 16 | 30 | 6 | 0 | 3 | 12 | 0 | 3 | 0 |
| s__CAG-110_sp900315595 | 34 | 15 | 35 | 6 | 2 | 29 | 8 | 1 | 2 | 4 | 9 | 1 | 1 | 1 | 4 | 0 | 0 | 1 |
| s__CAG-194_sp000432915 | 0 | 4 | 2 | 0 | 9 | 13 | 10 | 65 | 95 | 31 | 58 | 21 | 2 | 4 | 67 | 0 | 8 | 4 |
| s__CAG-217_sp000436335 | 35 | 564 | 56 | 12 | 176 | 135 | 77 | 1 | 101 | 11 | 8 | 3 | 0 | 2 | 170 | 0 | 3 | 1 |
| s__CAG-302_sp002375205 | 1 | 2 | 16 | 1 | 0 | 3 | 7 | 0 | 2 | 0 | 0 | 1 | 0 | 0 | 1 | 0 | 0 | 0 |
| s__CAG-306_sp000980375 | 3 | 1 | 1 | 9 | 5 | 3 | 1 | 2 | 2 | 4 | 5 | 8 | 1 | 9 | 3 | 0 | 2 | 2 |
| s__CAG-353_sp900066885 | 5 | 0 | 22 | 0 | 0 | 1 | 12 | 141 | 3 | 217 | 126 | 21 | 1 | 0 | 8 | 0 | 6 | 2 |
| s__CAG-41_sp001941225 | 1 | 99 | 6 | 28 | 0 | 180 | 31 | 28 | 36 | 7 | 14 | 13 | 3 | 20 | 46 | 0 | 4 | 1 |
| s__CAG-41_sp900066215 | 50 | 13 | 36 | 27 | 52 | 21 | 137 | 201 | 458 | 424 | 867 | 551 | 70 | 240 | 548 | 0 | 57 | 34 |
| s__CAG-45_sp900066395 | 0 | 105 | 1 | 0 | 0 | 0 | 0 | 0 | 0 | 1 | 0 | 1 | 0 | 0 | 0 | 0 | 0 | 0 |
| s__CAG-484_sp000431315 | 3 | 5 | 0 | 3 | 0 | 6 | 8 | 3 | 2 | 3 | 14 | 4 | 1 | 4 | 3 | 0 | 0 | 0 |
| s__CAG-495_sp001917125 | 2 | 4 | 1 | 2 | 7 | 0 | 26 | 4 | 2 | 3 | 1 | 2 | 1 | 1 | 2 | 0 | 0 | 0 |
| s__CAG-791_sp900317475 | 1 | 0 | 3 | 0 | 0 | 0 | 3 | 35 | 12 | 3 | 12 | 14 | 1 | 5 | 2 | 0 | 0 | 1 |
| s__CAG-81_sp900066785 | 1 | 4 | 6 | 1 | 21 | 170 | 1 | 22 | 17 | 20 | 8 | 16 | 5 | 12 | 15 | 0 | 2 | 2 |
| s__CAG-822_sp000432855 | 1 | 0 | 0 | 0 | 0 | 0 | 5 | 5 | 48 | 1 | 0 | 24 | 3 | 8 | 47 | 0 | 2 | 1 |
| s__CAG-878_sp000432255 | 21 | 0 | 17 | 0 | 1 | 1 | 0 | 16 | 2 | 0 | 0 | 1 | 0 | 0 | 4 | 0 | 0 | 0 |
| s__CAG-95_sp000403495 | 2 | 9 | 48 | 2 | 6 | 53 | 7 | 145 | 158 | 122 | 161 | 260 | 16 | 22 | 106 | 0 | 10 | 3 |
| s__CAG-988_sp003149915 | 0 | 0 | 0 | 0 | 0 | 1 | 0 | 0 | 5 | 1 | 0 | 0 | 0 | 1 | 3 | 0 | 0 | 0 |
| s__CDF_sp001187615 | 0 | 0 | 0 | 0 | 0 | 0 | 0 | 0 | 0 | 0 | 0 | 0 | 1 | 1 | 0 | 1 | 19 | 3 |
| s__CG2-30-66-27_sp001873935 | 1 | 2 | 2 | 0 | 3 | 5 | 2 | 3 | 2 | 5 | 2 | 3 | 1 | 1 | 2 | 1 | 1 | 0 |
| s__CHKCI006_sp900018345 | 0 | 87 | 0 | 0 | 1 | 1 | 3 | 0 | 1 | 0 | 1 | 6 | 0 | 1 | 0 | 0 | 0 | 0 |
| s__COE1_sp000403215 | 0 | 0 | 0 | 0 | 0 | 2 | 0 | 6 | 2 | 9 | 9 | 1 | 0 | 0 | 1 | 0 | 1 | 0 |
| s__COE1_sp000403335 | 28 | 1 | 1 | 36 | 7 | 63 | 4 | 28 | 6 | 8 | 6 | 6 | 1 | 0 | 5 | 0 | 0 | 0 |
| s__Caldilinea_aerophila | 0 | 0 | 0 | 0 | 0 | 0 | 0 | 0 | 0 | 0 | 0 | 0 | 0 | 0 | 0 | 0 | 2 | 0 |
| s__Christensenella_A_timonensis | 2 | 0 | 1 | 4 | 1 | 4 | 4 | 0 | 6 | 3 | 3 | 5 | 1 | 6 | 2 | 0 | 0 | 1 |
| s__Christensenella_massiliensis | 18 | 11 | 12 | 7 | 21 | 14 | 3 | 5 | 3 | 7 | 6 | 8 | 2 | 2 | 8 | 0 | 0 | 0 |
| s__Christensenella_minuta | 9 | 2 | 5 | 8 | 3 | 4 | 3 | 7 | 0 | 3 | 4 | 6 | 0 | 1 | 1 | 0 | 1 | 0 |
| s__Chryseobacterium_geocarposphaerae | 1 | 0 | 1 | 1 | 0 | 1 | 1 | 1 | 3 | 0 | 2 | 2 | 0 | 1 | 0 | 0 | 2 | 0 |
| s__Chryseobacterium_oncorhynchi | 2 | 1 | 0 | 0 | 4 | 7 | 1 | 2 | 1 | 0 | 0 | 2 | 0 | 0 | 1 | 0 | 0 | 0 |
| s__Chryseobacterium_sp002899825 | 138 | 1283 | 1560 | 620 | 202 | 688 | 91 | 364 | 62 | 42 | 50 | 115 | 11 | 16 | 89 | 1 | 5 | 8 |
| s__Chryseobacterium_taihuense | 0 | 1 | 1 | 1 | 3 | 1 | 0 | 0 | 0 | 0 | 0 | 0 | 0 | 0 | 0 | 0 | 0 | 0 |
| s__Chryseobacterium_viscerum | 0 | 0 | 0 | 0 | 0 | 0 | 15 | 0 | 12 | 8 | 4 | 21 | 1 | 2 | 16 | 0 | 0 | 0 |
| s__Citrobacter_portucalensis | 0 | 0 | 0 | 0 | 1 | 1 | 0 | 0 | 0 | 0 | 0 | 0 | 2 | 4 | 0 | 1 | 63 | 19 |
| s__Clostridium_AM_magnum | 0 | 0 | 0 | 0 | 0 | 0 | 0 | 1 | 0 | 0 | 0 | 0 | 0 | 0 | 0 | 0 | 26 | 0 |
| s__Clostridium_A_leptum | 0 | 0 | 0 | 0 | 0 | 0 | 1 | 3 | 2 | 1 | 3 | 2 | 0 | 0 | 2 | 0 | 0 | 0 |
| s__Clostridium_B_tyrobutyricum | 0 | 0 | 0 | 0 | 0 | 0 | 0 | 0 | 0 | 0 | 0 | 0 | 0 | 0 | 0 | 0 | 86 | 0 |
| s__Clostridium_C_acetireducens | 0 | 1 | 0 | 0 | 0 | 0 | 0 | 0 | 0 | 0 | 0 | 0 | 0 | 0 | 0 | 0 | 27 | 0 |
| s__Clostridium_H_homopropionicum | 0 | 0 | 0 | 0 | 0 | 0 | 0 | 0 | 0 | 0 | 0 | 0 | 0 | 0 | 0 | 0 | 5 | 0 |
| s__Clostridium_N_fimetarium | 3 | 47 | 3 | 47 | 0 | 1 | 0 | 17 | 58 | 1 | 0 | 6 | 2 | 1 | 33 | 0 | 7 | 2 |
| s__Clostridium_Q_saccharolyticum | 17 | 21 | 52 | 18 | 777 | 347 | 4 | 116 | 99 | 92 | 80 | 68 | 3 | 9 | 64 | 0 | 10 | 9 |
| s__Clostridium_Q_sp003024715 | 0 | 2 | 1 | 0 | 0 | 0 | 1 | 3 | 0 | 9 | 5 | 1 | 0 | 0 | 0 | 0 | 0 | 0 |
| s__Clostridium_Q_symbiosum | 1 | 0 | 2 | 0 | 2 | 4 | 2 | 7 | 0 | 11 | 3 | 1 | 0 | 0 | 1 | 0 | 0 | 0 |
| s__Clostridium_S_pasteurianum | 0 | 0 | 0 | 0 | 0 | 0 | 1 | 0 | 0 | 0 | 0 | 0 | 1 | 0 | 0 | 0 | 178 | 0 |
| s__Clostridium_saudiense | 0 | 0 | 1 | 0 | 1 | 5 | 0 | 0 | 0 | 3 | 0 | 0 | 8 | 3 | 0 | 1 | 50 | 4 |
| s__Collinsella_provencensis | 8 | 10 | 7 | 1 | 0 | 2 | 3 | 13 | 9 | 11 | 7 | 8 | 1 | 8 | 14 | 0 | 2 | 0 |
| s__Comamonas_D_granuli | 0 | 0 | 0 | 0 | 0 | 0 | 0 | 0 | 0 | 0 | 0 | 0 | 0 | 0 | 0 | 0 | 7 | 0 |
| s__Coprobacter_fastidiosus | 1 | 0 | 3 | 3 | 0 | 2 | 0 | 0 | 0 | 0 | 0 | 0 | 0 | 0 | 0 | 0 | 0 | 0 |
| s__Coprococcus_B_comes | 1 | 8 | 11 | 0 | 0 | 28 | 1 | 30 | 4 | 15 | 25 | 6 | 3 | 4 | 4 | 0 | 1 | 0 |
| s__Corynebacterium_capitovis | 0 | 0 | 0 | 0 | 0 | 0 | 0 | 0 | 0 | 0 | 0 | 0 | 0 | 0 | 0 | 0 | 3 | 0 |
| s__Corynebacterium_freneyi | 0 | 0 | 1 | 0 | 1 | 0 | 0 | 2 | 1 | 1 | 1 | 2 | 41 | 131 | 0 | 8 | 212 | 197 |
| s__Corynebacterium_glutamicum | 0 | 0 | 0 | 0 | 0 | 0 | 3 | 1 | 2 | 2 | 1 | 0 | 4 | 21 | 1 | 0 | 22 | 62 |
| s__Corynebacterium_nuruki | 1 | 2 | 0 | 0 | 0 | 0 | 2 | 5 | 0 | 3 | 4 | 2 | 1 | 0 | 1 | 1 | 1 | 0 |
| s__Corynebacterium_stationis | 1 | 2 | 2 | 3 | 0 | 1 | 0 | 1 | 3 | 2 | 1 | 2 | 111 | 154 | 1 | 6 | 461 | 242 |
| s__Corynebacterium_urealyticum | 0 | 0 | 0 | 0 | 1 | 0 | 0 | 0 | 0 | 0 | 0 | 0 | 0 | 2 | 0 | 0 | 0 | 0 |
| s__Cronobacter_dublinensis | 642 | 1345 | 907 | 1716 | 1099 | 2096 | 324 | 185 | 192 | 2123 | 1784 | 69 | 56 | 100 | 165 | 2 | 50 | 59 |
| s__Cronobacter_malonaticus | 3973 | 13210 | 2167 | 1672 | 2717 | 2152 | 1627 | 454 | 387 | 473 | 532 | 1176 | 641 | 1397 | 455 | 1531 | 117 | 44 |
| s__Cronobacter_sakazakii | 6255 | 2707 | 7021 | 7164 | 5521 | 4623 | 3866 | 3900 | 2842 | 5733 | 4990 | 3725 | 276 | 858 | 2364 | 21 | 359 | 177 |
| s__Cryobacterium_A_mesophilum | 1 | 0 | 0 | 0 | 0 | 0 | 0 | 0 | 1 | 0 | 0 | 0 | 1 | 5 | 0 | 0 | 11 | 7 |
| s__DNF00809_sp000814825 | 10 | 5 | 12 | 2 | 7 | 3 | 2 | 9 | 3 | 2 | 2 | 7 | 0 | 14 | 6 | 0 | 1 | 3 |
| s__DNF00809_sp001552935 | 0 | 1 | 5 | 7 | 1 | 2 | 3 | 8 | 1 | 1 | 5 | 3 | 0 | 4 | 1 | 0 | 1 | 0 |
| s__DTU053_sp001512765 | 9 | 4 | 2 | 0 | 7 | 0 | 0 | 0 | 0 | 0 | 0 | 0 | 0 | 0 | 0 | 0 | 0 | 0 |
| s__Dakarella_massiliensis | 0 | 0 | 0 | 0 | 0 | 0 | 0 | 0 | 0 | 0 | 0 | 0 | 0 | 0 | 0 | 0 | 152 | 1 |
| s__Defluviitalea_phaphyphila | 1 | 0 | 6 | 0 | 0 | 3 | 1 | 0 | 1 | 0 | 0 | 4 | 0 | 0 | 1 | 0 | 0 | 0 |
| s__Dehalobacterium_formicoaceticum | 9 | 11 | 4 | 0 | 2 | 1 | 1 | 0 | 1 | 1 | 1 | 3 | 0 | 0 | 0 | 0 | 0 | 0 |
| s__Denitrobacterium_detoxificans | 0 | 0 | 0 | 0 | 0 | 0 | 19 | 14 | 18 | 5 | 7 | 20 | 0 | 9 | 13 | 0 | 3 | 0 |
| s__Desulfonispora_thiosulfatigenes | 2 | 3 | 23 | 3 | 1 | 0 | 5 | 0 | 0 | 0 | 0 | 0 | 0 | 0 | 0 | 0 | 0 | 0 |
| s__Desulfovibrio_fairfieldensis | 1 | 160 | 78 | 2 | 208 | 360 | 46 | 507 | 16 | 308 | 358 | 369 | 30 | 6 | 270 | 1 | 27 | 19 |
| s__Desulfuromonas_B_soudanensis | 0 | 0 | 0 | 0 | 0 | 0 | 0 | 0 | 0 | 0 | 0 | 0 | 0 | 1 | 0 | 0 | 4 | 1 |
| s__Devosia_epidermidihirudinis | 0 | 0 | 0 | 0 | 0 | 0 | 0 | 0 | 0 | 0 | 0 | 0 | 3 | 13 | 0 | 0 | 108 | 28 |
| s__Devosia_riboflavina | 0 | 0 | 0 | 0 | 0 | 0 | 0 | 0 | 0 | 0 | 0 | 0 | 4 | 0 | 1 | 0 | 7 | 4 |
| s__Devosia_sp001402915 | 0 | 0 | 0 | 0 | 0 | 0 | 0 | 0 | 0 | 0 | 0 | 0 | 3 | 0 | 0 | 0 | 6 | 3 |
| s__Dialister_sp900343095 | 0 | 0 | 0 | 0 | 0 | 0 | 0 | 0 | 0 | 0 | 0 | 0 | 0 | 0 | 0 | 0 | 24 | 0 |
| s__Dietzia_lutea | 0 | 0 | 0 | 0 | 0 | 0 | 0 | 0 | 0 | 0 | 0 | 0 | 8 | 40 | 0 | 1 | 56 | 35 |
| s__Dorea_formicigenerans | 0 | 15 | 7 | 3 | 9 | 6 | 12 | 59 | 32 | 53 | 57 | 36 | 1 | 5 | 19 | 1 | 4 | 2 |
| s__Dorea_longicatena_B | 4 | 0 | 3 | 1 | 0 | 5 | 0 | 1 | 5 | 13 | 12 | 4 | 1 | 0 | 3 | 0 | 1 | 0 |
| s__Dorea_scindens | 2 | 2 | 13 | 4 | 0 | 4 | 0 | 2 | 18 | 5 | 0 | 12 | 1 | 6 | 10 | 0 | 0 | 0 |
| s__Dorea_sp000403475 | 2 | 22 | 30 | 13 | 68 | 102 | 10 | 122 | 73 | 68 | 75 | 40 | 5 | 25 | 34 | 0 | 11 | 11 |
| s__Dorea_sp001185345 | 3 | 3 | 1 | 2 | 0 | 2 | 4 | 20 | 77 | 109 | 121 | 46 | 2 | 9 | 87 | 1 | 7 | 5 |
| s__Dorea_sp002160985 | 1 | 21 | 1 | 0 | 0 | 11 | 0 | 0 | 1 | 1 | 1 | 0 | 0 | 0 | 0 | 0 | 0 | 0 |
| s__Duncaniella_muris | 540 | 366 | 528 | 877 | 600 | 491 | 158 | 144 | 126 | 443 | 190 | 130 | 23 | 48 | 84 | 0 | 15 | 26 |
| s__ER4_sp000765235 | 2 | 0 | 0 | 0 | 3 | 8 | 0 | 1 | 0 | 1 | 2 | 2 | 0 | 0 | 0 | 0 | 0 | 0 |
| s__Effusibacillus_lacus | 0 | 3 | 3 | 3 | 2 | 0 | 1 | 3 | 0 | 0 | 1 | 0 | 2 | 5 | 4 | 2 | 0 | 0 |
| s__Eggerthella_timonensis | 0 | 0 | 0 | 0 | 0 | 0 | 2 | 13 | 12 | 15 | 5 | 7 | 4 | 15 | 8 | 0 | 1 | 0 |
| s__Eisenbergiella_tayi | 13 | 3 | 3 | 1 | 23 | 23 | 2 | 24 | 12 | 14 | 15 | 7 | 0 | 1 | 7 | 0 | 0 | 0 |
| s__Emergencia_timonensis | 46 | 42 | 38 | 17 | 30 | 33 | 13 | 5 | 4 | 8 | 6 | 8 | 3 | 0 | 9 | 1 | 0 | 1 |
| s__Enterococcus_rotai | 178 | 206 | 163 | 161 | 247 | 231 | 192 | 377 | 279 | 335 | 225 | 254 | 580 | 596 | 340 | 749 | 219 | 385 |
| s__Enterococcus_ureasiticus | 2 | 1 | 2 | 2 | 1 | 2 | 5 | 5 | 3 | 7 | 3 | 2 | 11 | 6 | 1 | 11 | 4 | 5 |
| s__Enteroscipio_rubneri | 0 | 0 | 14 | 0 | 3 | 0 | 3 | 7 | 8 | 13 | 10 | 2 | 3 | 2 | 4 | 0 | 2 | 1 |
| s__Enteroscipio_sp000270285 | 1 | 4 | 6 | 2 | 2 | 3 | 1 | 5 | 4 | 2 | 4 | 8 | 0 | 2 | 2 | 0 | 0 | 0 |
| s__Erwinia_coffeiphila | 4 | 0 | 1 | 3 | 0 | 20 | 0 | 0 | 0 | 1 | 2 | 3 | 0 | 0 | 0 | 0 | 0 | 0 |
| s__Erysipelatoclostridium_sp000752095 | 0 | 28 | 2 | 0 | 0 | 22 | 11 | 3 | 10 | 0 | 0 | 22 | 1 | 2 | 6 | 0 | 2 | 1 |
| s__Escherichia_coli_D | 0 | 0 | 2 | 0 | 0 | 0 | 0 | 0 | 0 | 0 | 0 | 0 | 0 | 0 | 0 | 0 | 0 | 0 |
| s__Escherichia_flexneri | 3 | 2 | 1 | 1 | 1 | 3 | 53 | 149 | 730 | 349 | 177 | 328 | 9 | 37 | 378 | 0 | 21 | 12 |
| s__Eubacterium_A_pyruvativorans | 0 | 0 | 0 | 0 | 0 | 0 | 2 | 3 | 5 | 0 | 0 | 1 | 1 | 1 | 3 | 0 | 2 | 0 |
| s__Eubacterium_C_oxidoreducens | 0 | 1 | 0 | 0 | 0 | 2 | 2 | 14 | 6 | 7 | 9 | 4 | 0 | 1 | 7 | 0 | 1 | 0 |
| s__Eubacterium_F_xylanophilum | 0 | 0 | 0 | 0 | 0 | 0 | 2 | 39 | 35 | 0 | 2 | 75 | 6 | 10 | 32 | 0 | 1 | 0 |
| s__Eubacterium_G_ventriosum | 14 | 4 | 19 | 8 | 12 | 2 | 9 | 6 | 0 | 26 | 16 | 17 | 0 | 0 | 0 | 0 | 1 | 0 |
| s__Eubacterium_I_sp000270305 | 12 | 0 | 30 | 0 | 1 | 0 | 1 | 0 | 0 | 1 | 0 | 2 | 0 | 0 | 0 | 0 | 0 | 0 |
| s__Eubacterium_J_plexicaudatum | 0 | 8 | 9 | 68 | 0 | 9 | 0 | 4 | 10 | 5 | 2 | 2 | 0 | 1 | 3 | 0 | 1 | 0 |
| s__Eubacterium_Q_ruminantium_A | 3 | 82 | 2 | 118 | 1 | 223 | 10 | 33 | 265 | 9 | 240 | 169 | 8 | 10 | 198 | 0 | 7 | 14 |
| s__Eubacterium_R_coprostanoligenes | 18 | 1 | 126 | 847 | 37 | 4 | 63 | 27 | 1 | 26 | 96 | 52 | 8 | 11 | 0 | 1 | 9 | 5 |
| s__Eubacterium_R_sp000436835 | 1818 | 244 | 759 | 294 | 407 | 305 | 1489 | 459 | 436 | 409 | 215 | 501 | 48 | 235 | 428 | 1 | 57 | 27 |
| s__Exiguobacterium_A_acetylicum | 4 | 10 | 4 | 2 | 7 | 3 | 10 | 7 | 5 | 12 | 6 | 9 | 21 | 15 | 8 | 22 | 4 | 19 |
| s__Exiguobacterium_mexicanum | 3 | 4 | 2 | 2 | 6 | 2 | 8 | 6 | 8 | 5 | 4 | 3 | 7 | 2 | 2 | 11 | 4 | 4 |
| s__F0428_sp003043955 | 0 | 0 | 0 | 0 | 0 | 0 | 0 | 0 | 0 | 0 | 0 | 0 | 0 | 1 | 0 | 0 | 5 | 3 |
| s__Facklamia_hominis | 0 | 0 | 0 | 0 | 0 | 0 | 0 | 0 | 0 | 0 | 0 | 0 | 0 | 0 | 0 | 0 | 3 | 0 |
| s__Facklamia_miroungae | 0 | 0 | 0 | 0 | 0 | 0 | 1 | 0 | 0 | 0 | 0 | 0 | 1 | 0 | 0 | 1 | 11 | 4 |
| s__Faecalibacterium_prausnitzii_D | 0 | 0 | 0 | 0 | 0 | 0 | 0 | 0 | 0 | 0 | 0 | 0 | 0 | 0 | 0 | 0 | 20 | 0 |
| s__Faecalibacterium_prausnitzii_E | 0 | 0 | 0 | 0 | 2 | 2 | 1 | 2 | 0 | 1 | 1 | 1 | 0 | 0 | 1 | 0 | 0 | 0 |
| s__Faecalibaculum_rodentium | 9 | 13 | 146 | 4 | 2 | 20 | 0 | 0 | 0 | 0 | 0 | 1 | 0 | 0 | 0 | 0 | 0 | 1 |
| s__Faecalicatena_orotica | 3 | 6 | 6 | 2 | 29 | 2 | 13 | 89 | 30 | 87 | 84 | 29 | 2 | 6 | 18 | 0 | 3 | 2 |
| s__Faecalicatena_sp000364245 | 7 | 7 | 13 | 1 | 14 | 8 | 1 | 154 | 13 | 58 | 108 | 25 | 9 | 2 | 21 | 0 | 1 | 2 |
| s__Faecalicatena_sp000403295 | 28 | 2 | 74 | 55 | 48 | 365 | 4 | 58 | 29 | 55 | 132 | 6 | 7 | 2 | 25 | 1 | 4 | 4 |
| s__Fermentibacter_daniensis | 1 | 1 | 0 | 0 | 0 | 1 | 0 | 2 | 1 | 0 | 1 | 0 | 1 | 2 | 0 | 3 | 1 | 0 |
| s__Flavisolibacter_ginsengisoli | 0 | 0 | 0 | 0 | 2 | 0 | 0 | 0 | 0 | 2 | 0 | 0 | 0 | 0 | 0 | 0 | 0 | 0 |
| s__Flavobacterium_A_sp002807015 | 0 | 0 | 0 | 0 | 0 | 0 | 0 | 0 | 0 | 0 | 0 | 0 | 0 | 0 | 0 | 0 | 5 | 1 |
| s__Flavobacterium_terrigena | 0 | 0 | 0 | 0 | 0 | 0 | 0 | 0 | 0 | 0 | 0 | 0 | 0 | 0 | 0 | 0 | 7 | 0 |
| s__Flavonifractor_sp002161085 | 39 | 83 | 50 | 106 | 140 | 126 | 69 | 434 | 270 | 289 | 240 | 287 | 12 | 26 | 156 | 1 | 12 | 7 |
| s__Flavonifractor_sp900199495 | 11 | 8 | 58 | 0 | 0 | 11 | 116 | 89 | 22 | 102 | 131 | 88 | 17 | 30 | 26 | 1 | 5 | 7 |
| s__Fournierella_massiliensis | 12 | 32 | 23 | 9 | 100 | 27 | 11 | 105 | 29 | 57 | 7 | 13 | 2 | 3 | 17 | 0 | 3 | 0 |
| s__Fournierella_sp002160145 | 18 | 11 | 10 | 5 | 7 | 11 | 2 | 10 | 1 | 0 | 1 | 2 | 0 | 1 | 1 | 0 | 0 | 0 |
| s__GCA-900066575_sp002160765 | 0 | 2 | 1 | 0 | 0 | 1 | 0 | 0 | 0 | 0 | 0 | 0 | 0 | 0 | 0 | 0 | 0 | 0 |
| s__GCA-900066575_sp900066385 | 15 | 49 | 31 | 22 | 13 | 42 | 4 | 99 | 52 | 48 | 61 | 42 | 2 | 3 | 37 | 0 | 10 | 1 |
| s__GCA-900066905_sp900066905 | 0 | 0 | 1 | 0 | 1 | 0 | 0 | 0 | 0 | 0 | 0 | 0 | 0 | 0 | 1 | 0 | 0 | 0 |
| s__GW-Nitrospira-1_sp002839535 | 0 | 1 | 1 | 0 | 1 | 0 | 1 | 2 | 0 | 1 | 1 | 2 | 0 | 0 | 0 | 0 | 0 | 0 |
| s__Gemmata_massiliana | 0 | 0 | 0 | 0 | 0 | 0 | 0 | 0 | 0 | 0 | 0 | 0 | 0 | 0 | 0 | 0 | 3 | 0 |
| s__Globicatella_sulfidifaciens | 0 | 0 | 0 | 0 | 0 | 0 | 2 | 0 | 1 | 0 | 1 | 4 | 0 | 0 | 0 | 0 | 0 | 0 |
| s__Gordonibacter_massiliensis | 2 | 6 | 2 | 7 | 11 | 6 | 0 | 0 | 0 | 0 | 0 | 0 | 0 | 0 | 0 | 0 | 0 | 0 |
| s__Gordonibacter_pamelaeae | 0 | 0 | 0 | 0 | 0 | 0 | 0 | 3 | 1 | 3 | 0 | 4 | 1 | 3 | 0 | 0 | 0 | 0 |
| s__Gracilibacter_sp001516055 | 0 | 0 | 0 | 0 | 1 | 0 | 1 | 0 | 0 | 0 | 0 | 2 | 0 | 0 | 0 | 0 | 0 | 0 |
| s__Halomonas_D_sp002286965 | 0 | 1 | 0 | 0 | 0 | 0 | 1 | 0 | 1 | 0 | 0 | 0 | 0 | 0 | 0 | 1 | 0 | 2 |
| s__Halomonas_sulfidaeris | 0 | 0 | 0 | 0 | 0 | 0 | 0 | 0 | 0 | 0 | 0 | 0 | 2 | 2 | 0 | 2 | 22 | 8 |
| s__Helicobacter_C_japonicus | 0 | 0 | 0 | 0 | 0 | 0 | 1 | 3 | 11 | 30 | 2 | 57 | 4 | 6 | 20 | 0 | 0 | 0 |
| s__Helicobacter_C_typhlonius | 92 | 18 | 105 | 46 | 22 | 283 | 0 | 1 | 0 | 1 | 0 | 0 | 0 | 0 | 1 | 0 | 0 | 2 |
| s__Helicobacter_D_sp000765695 | 1 | 2 | 6 | 3 | 0 | 22 | 3 | 31 | 12 | 265 | 35 | 34 | 1 | 3 | 55 | 0 | 1 | 2 |
| s__Holdemania_sp900120005 | 4 | 3 | 11 | 0 | 3 | 9 | 0 | 4 | 3 | 3 | 7 | 5 | 0 | 0 | 0 | 0 | 0 | 0 |
| s__Hydrogenoanaerobacterium_saccharovorans | 3 | 3 | 5 | 1 | 3 | 1 | 2 | 2 | 1 | 0 | 1 | 3 | 0 | 1 | 0 | 0 | 0 | 0 |
| s__Hydrogenophaga_intermedia | 0 | 0 | 0 | 0 | 0 | 0 | 0 | 0 | 0 | 0 | 1 | 0 | 0 | 0 | 0 | 0 | 151 | 0 |
| s__Hyphomicrobium_zavarzinii | 2 | 0 | 0 | 0 | 1 | 2 | 0 | 1 | 2 | 0 | 3 | 1 | 0 | 0 | 1 | 0 | 2 | 0 |
| s__Intestinibacillus_massiliensis | 0 | 0 | 0 | 0 | 0 | 0 | 0 | 1 | 1 | 1 | 1 | 2 | 0 | 0 | 1 | 0 | 1 | 0 |
| s__Intestinimonas_butyriciproducens | 14 | 177 | 70 | 18 | 18 | 6 | 171 | 59 | 113 | 76 | 372 | 83 | 26 | 54 | 86 | 0 | 10 | 8 |
| s__Intestinimonas_massiliensis | 1 | 0 | 1 | 0 | 0 | 1 | 4 | 4 | 1 | 4 | 2 | 0 | 0 | 0 | 1 | 0 | 0 | 0 |
| s__JJ008_sp002400415 | 0 | 0 | 0 | 0 | 0 | 0 | 0 | 0 | 0 | 0 | 0 | 0 | 0 | 0 | 0 | 0 | 7 | 0 |
| s__Jeotgalicoccus_halophilus | 0 | 0 | 0 | 0 | 0 | 0 | 0 | 0 | 0 | 0 | 0 | 0 | 10 | 2 | 0 | 0 | 29 | 8 |
| s__Jeotgalicoccus_marinus | 0 | 0 | 0 | 0 | 0 | 0 | 0 | 0 | 0 | 0 | 0 | 0 | 0 | 0 | 0 | 1 | 1 | 0 |
| s__Kineothrix_alysoides | 249 | 337 | 35 | 66 | 111 | 1596 | 18 | 2166 | 694 | 1721 | 3228 | 1199 | 77 | 330 | 406 | 0 | 75 | 40 |
| s__Kineothrix_sp000403275 | 2 | 1 | 3 | 37 | 0 | 246 | 0 | 4 | 3 | 2 | 4 | 1 | 0 | 0 | 0 | 0 | 1 | 0 |
| s__Klebsiella_pneumoniae | 31 | 68 | 89 | 32 | 60 | 79 | 40 | 85 | 53 | 76 | 90 | 68 | 83 | 82 | 54 | 43 | 577 | 254 |
| s__LD21_sp002428385 | 12 | 14 | 20 | 9 | 30 | 29 | 20 | 7 | 8 | 13 | 16 | 13 | 1 | 5 | 8 | 0 | 5 | 4 |
| s__Lachnoclostridium_A_edouardi | 0 | 9 | 1 | 3 | 19 | 10 | 0 | 7 | 6 | 6 | 1 | 7 | 0 | 6 | 0 | 0 | 0 | 0 |
| s__Lachnoclostridium_A_sp002160755 | 6 | 3 | 6 | 3 | 5 | 1 | 0 | 8 | 8 | 5 | 6 | 6 | 2 | 4 | 6 | 0 | 0 | 0 |
| s__Lachnospira_rogosae | 0 | 12 | 0 | 18 | 0 | 35 | 0 | 0 | 1 | 0 | 1 | 0 | 0 | 0 | 0 | 0 | 0 | 0 |
| s__Lachnotalea_glycerini | 0 | 0 | 2 | 0 | 1 | 4 | 0 | 0 | 0 | 0 | 0 | 0 | 0 | 0 | 0 | 0 | 0 | 0 |
| s__Lactobacillus_B_apodemi | 381 | 3230 | 1307 | 215 | 4385 | 2353 | 184 | 675 | 492 | 406 | 103 | 144 | 6828 | 16355 | 677 | 20500 | 208 | 107 |
| s__Lactobacillus_E_bifermentans | 0 | 0 | 0 | 0 | 0 | 0 | 0 | 0 | 0 | 0 | 0 | 0 | 0 | 0 | 0 | 0 | 19 | 0 |
| s__Lactobacillus_F_plantarum_A | 1 | 0 | 0 | 1 | 1 | 0 | 0 | 3 | 1 | 0 | 0 | 0 | 0 | 5 | 1 | 1 | 2 | 2 |
| s__Lactobacillus_G_parabuchneri | 0 | 0 | 0 | 0 | 0 | 0 | 0 | 0 | 0 | 0 | 0 | 0 | 0 | 0 | 0 | 0 | 8 | 0 |
| s__Lactobacillus_H_mucosae | 0 | 0 | 0 | 0 | 0 | 0 | 0 | 1 | 1 | 0 | 0 | 0 | 0 | 1 | 0 | 0 | 80 | 0 |
| s__Lactobacillus_H_vaginalis_A | 2388 | 130 | 660 | 1393 | 693 | 779 | 1117 | 2742 | 2408 | 1755 | 731 | 979 | 11350 | 7912 | 3215 | 9927 | 4024 | 11377 |
| s__Lactobacillus_delbrueckii | 3 | 0 | 0 | 0 | 0 | 0 | 0 | 0 | 0 | 0 | 0 | 0 | 0 | 0 | 0 | 0 | 41 | 0 |
| s__Lactobacillus_intestinalis | 194 | 58 | 86 | 2 | 66 | 125 | 307 | 114 | 228 | 247 | 74 | 48 | 236 | 13 | 142 | 14 | 28 | 38 |
| s__Lactobacillus_johnsonii | 5167 | 1361 | 2374 | 4451 | 1528 | 1256 | 6036 | 6655 | 5114 | 8144 | 4276 | 5343 | 10474 | 1837 | 9951 | 7392 | 846 | 34899 |
| s__Lawsonibacter_asaccharolyticus | 15 | 40 | 22 | 96 | 96 | 98 | 47 | 98 | 93 | 336 | 131 | 65 | 2 | 7 | 36 | 0 | 4 | 2 |
| s__Lawsonibacter_sp000492175 | 11 | 28 | 48 | 37 | 106 | 103 | 26 | 517 | 360 | 490 | 338 | 576 | 13 | 47 | 216 | 0 | 30 | 15 |
| s__Lawsonibacter_sp002160305 | 4 | 4 | 7 | 1 | 8 | 23 | 33 | 23 | 8 | 32 | 33 | 15 | 2 | 8 | 7 | 0 | 3 | 1 |
| s__Lawsonibacter_sp002161175 | 14 | 17 | 27 | 20 | 55 | 85 | 6 | 120 | 64 | 1089 | 1067 | 165 | 9 | 22 | 46 | 1 | 13 | 3 |
| s__Lentimicrobium_saccharophilum | 0 | 1 | 0 | 0 | 0 | 0 | 2 | 7 | 22 | 46 | 43 | 34 | 0 | 1 | 18 | 0 | 0 | 1 |
| s__Listeria_seeligeri | 0 | 0 | 0 | 0 | 0 | 0 | 0 | 0 | 0 | 0 | 0 | 0 | 0 | 0 | 0 | 0 | 1 | 8 |
| s__Longilinea_arvoryzae | 1 | 0 | 1 | 0 | 1 | 0 | 0 | 0 | 2 | 1 | 1 | 0 | 1 | 2 | 0 | 0 | 5 | 0 |
| s__Luteimonas_sp001014645 | 0 | 0 | 0 | 0 | 0 | 0 | 0 | 0 | 0 | 0 | 0 | 0 | 1 | 0 | 0 | 0 | 2 | 0 |
| s__Luteimonas_sp001717465 | 0 | 0 | 0 | 0 | 0 | 0 | 0 | 0 | 0 | 0 | 0 | 0 | 2 | 5 | 0 | 0 | 35 | 6 |
| s__Lysinibacillus_A_sp900291985 | 0 | 0 | 0 | 0 | 0 | 0 | 0 | 0 | 1 | 1 | 0 | 0 | 1 | 16 | 0 | 0 | 21 | 2 |
| s__Lysinibacillus_D_sphaericus | 0 | 0 | 0 | 0 | 0 | 0 | 0 | 0 | 0 | 0 | 0 | 0 | 5 | 0 | 0 | 0 | 101 | 5 |
| s__MS4_sp000752215 | 0 | 2 | 0 | 1 | 2 | 1 | 7 | 27 | 14 | 22 | 11 | 45 | 3 | 8 | 8 | 0 | 1 | 1 |
| s__MYbin3_sp002753335 | 18 | 20 | 19 | 6 | 16 | 20 | 13 | 28 | 26 | 26 | 42 | 32 | 8 | 10 | 19 | 1 | 28 | 7 |
| s__Marseille-P3106_sp900169975 | 2 | 13 | 12 | 5 | 11 | 11 | 57 | 34 | 36 | 53 | 69 | 72 | 7 | 29 | 35 | 0 | 6 | 1 |
| s__Marvinbryantia_sp900066075 | 10 | 32 | 14 | 3 | 7 | 12 | 3 | 9 | 1 | 0 | 15 | 1 | 1 | 0 | 2 | 0 | 1 | 1 |
| s__Massilia_timonae_A | 0 | 0 | 0 | 0 | 1 | 1 | 0 | 0 | 0 | 0 | 1 | 0 | 0 | 0 | 0 | 0 | 15 | 0 |
| s__Massilimaliae_massiliensis | 12 | 5 | 11 | 4 | 12 | 12 | 0 | 45 | 16 | 1 | 5 | 21 | 0 | 2 | 17 | 0 | 3 | 0 |
| s__Massilioclostridium_coli | 0 | 0 | 0 | 0 | 0 | 0 | 2 | 6 | 3 | 3 | 0 | 5 | 0 | 4 | 3 | 0 | 0 | 2 |
| s__Massilioclostridium_methylpentosum | 5 | 0 | 10 | 2 | 13 | 2 | 2 | 65 | 65 | 33 | 41 | 23 | 0 | 1 | 47 | 0 | 6 | 3 |
| s__Megamonas_hypermegale | 40 | 131 | 204 | 10 | 883 | 654 | 2 | 0 | 1 | 1 | 1 | 1 | 3 | 1 | 1 | 3 | 0 | 2 |
| s__Megasphaera_cerevisiae | 0 | 0 | 0 | 0 | 0 | 0 | 0 | 0 | 0 | 0 | 0 | 0 | 0 | 0 | 0 | 0 | 17 | 0 |
| s__Megasphaera_sp000417505 | 1 | 0 | 0 | 0 | 0 | 0 | 0 | 0 | 0 | 0 | 0 | 0 | 1 | 1 | 0 | 1 | 346 | 0 |
| s__Megasphaera_sp900066485 | 0 | 0 | 0 | 0 | 0 | 0 | 0 | 0 | 0 | 0 | 0 | 0 | 0 | 0 | 0 | 0 | 23 | 0 |
| s__Meiothermus_B_silvanus | 7 | 6 | 10 | 5 | 8 | 5 | 7 | 10 | 11 | 13 | 9 | 10 | 4 | 2 | 9 | 5 | 14 | 6 |
| s__Mesotoga_infera_B | 0 | 1 | 2 | 3 | 1 | 2 | 0 | 12 | 1 | 1 | 1 | 0 | 0 | 0 | 0 | 0 | 0 | 0 |
| s__Methanosphaera_cuniculi | 5 | 16 | 35 | 0 | 5 | 33 | 0 | 0 | 0 | 0 | 0 | 0 | 0 | 0 | 0 | 0 | 0 | 0 |
| s__Methanosphaera_sp11921u | 0 | 0 | 0 | 0 | 0 | 0 | 0 | 2 | 0 | 1 | 6 | 26 | 0 | 0 | 0 | 0 | 0 | 1 |
| s__Methanothrix_soehngenii | 0 | 0 | 0 | 0 | 1 | 1 | 0 | 1 | 0 | 0 | 1 | 0 | 2 | 0 | 0 | 0 | 0 | 1 |
| s__Methylocystis_parvus | 0 | 0 | 0 | 0 | 0 | 0 | 0 | 1 | 1 | 1 | 1 | 0 | 0 | 0 | 0 | 0 | 6 | 0 |
| s__Microcystis_wesenbergii | 1 | 1 | 3 | 1 | 1 | 1 | 2 | 6 | 3 | 1 | 2 | 0 | 2 | 5 | 2 | 2 | 2 | 0 |
| s__Monoglobus_pectinilyticus | 1 | 1 | 2 | 5 | 2 | 1 | 33 | 33 | 47 | 48 | 75 | 41 | 13 | 24 | 64 | 0 | 4 | 3 |
| s__Morganella_morganii_B | 7 | 6 | 12 | 5 | 5 | 14 | 6 | 11 | 12 | 11 | 11 | 10 | 2 | 8 | 4 | 2 | 23 | 7 |
| s__Muribaculum_intestinale | 3 | 4 | 7 | 7 | 4 | 9 | 233 | 108 | 425 | 204 | 70 | 284 | 38 | 111 | 445 | 0 | 7 | 13 |
| s__Muribaculum_sp003150235 | 0 | 0 | 1 | 0 | 0 | 0 | 1 | 0 | 2 | 3 | 0 | 2 | 0 | 1 | 2 | 0 | 0 | 0 |
| s__NAK82_sp003130875 | 4 | 4 | 1 | 2 | 9 | 8 | 1 | 3 | 8 | 4 | 8 | 7 | 2 | 6 | 3 | 3 | 4 | 1 |
| s__Negativibacillus_massiliensis | 6 | 35 | 10 | 0 | 0 | 7 | 0 | 4 | 6 | 2 | 0 | 1 | 0 | 0 | 9 | 0 | 0 | 0 |
| s__Nioella_sediminis | 0 | 0 | 0 | 0 | 0 | 0 | 0 | 0 | 0 | 0 | 0 | 0 | 0 | 0 | 0 | 0 | 0 | 2 |
| s__Nocardiopsis_listeri | 0 | 0 | 0 | 0 | 0 | 0 | 0 | 0 | 0 | 0 | 0 | 0 | 0 | 0 | 0 | 0 | 5 | 2 |
| s__Nosocomiicoccus_ampullae | 0 | 0 | 0 | 0 | 0 | 0 | 0 | 0 | 0 | 0 | 0 | 0 | 9 | 1 | 0 | 0 | 25 | 12 |
| s__OEMS01_sp900199405 | 134 | 439 | 134 | 42 | 229 | 220 | 86 | 90 | 151 | 80 | 201 | 121 | 18 | 40 | 246 | 0 | 23 | 17 |
| s__Oceanisphaera_avium | 0 | 0 | 0 | 0 | 0 | 0 | 0 | 0 | 0 | 0 | 0 | 0 | 6 | 10 | 0 | 4 | 27 | 151 |
| s__Odoribacter_massiliensis | 0 | 0 | 0 | 0 | 0 | 0 | 7 | 13 | 3 | 19 | 14 | 10 | 1 | 2 | 2 | 0 | 1 | 0 |
| s__Odoribacter_splanchnicus | 3 | 5 | 4 | 0 | 5 | 4 | 0 | 0 | 0 | 0 | 1 | 0 | 0 | 0 | 0 | 0 | 0 | 0 |
| s__Oligella_ureolytica | 0 | 0 | 0 | 0 | 0 | 0 | 0 | 0 | 0 | 0 | 0 | 1 | 15 | 87 | 0 | 9 | 223 | 249 |
| s__Olsenella_profusa | 3 | 1 | 6 | 0 | 0 | 2 | 0 | 0 | 0 | 0 | 0 | 0 | 0 | 0 | 0 | 0 | 3 | 1 |
| s__Oscillibacter_ruminantium | 1 | 4 | 5 | 3 | 10 | 16 | 1 | 4 | 3 | 12 | 6 | 2 | 0 | 0 | 3 | 0 | 0 | 0 |
| s__Oscillibacter_sp000403435 | 9 | 16 | 16 | 27 | 66 | 161 | 13 | 72 | 41 | 188 | 84 | 47 | 3 | 0 | 32 | 0 | 3 | 1 |
| s__Paenalcaligenes_hominis | 0 | 0 | 0 | 0 | 0 | 0 | 0 | 1 | 0 | 0 | 0 | 0 | 17 | 1 | 0 | 9 | 169 | 267 |
| s__Pannonibacter_indicus | 0 | 1 | 0 | 0 | 0 | 0 | 0 | 0 | 0 | 0 | 0 | 0 | 0 | 0 | 1 | 0 | 58 | 0 |
| s__Papillibacter_cinnamivorans | 16 | 12 | 16 | 1 | 8 | 11 | 22 | 5 | 8 | 7 | 12 | 11 | 2 | 4 | 2 | 0 | 3 | 2 |
| s__Parabacteroides_distasonis | 25 | 63 | 64 | 53 | 115 | 41 | 2 | 0 | 0 | 0 | 1 | 1 | 0 | 1 | 0 | 0 | 3 | 0 |
| s__Parabacteroides_goldsteinii | 3 | 3 | 0 | 3 | 11 | 3 | 1 | 1 | 1 | 1 | 2 | 15 | 0 | 0 | 1 | 0 | 0 | 0 |
| s__Parabacteroides_johnsonii | 0 | 0 | 0 | 0 | 0 | 0 | 5 | 3 | 6 | 7 | 15 | 15 | 3 | 8 | 13 | 0 | 2 | 0 |
| s__Parabacteroides_merdae | 1 | 0 | 6 | 1 | 2 | 4 | 0 | 0 | 0 | 0 | 0 | 0 | 0 | 0 | 0 | 0 | 13 | 0 |
| s__Parabacteroides_sp900155425 | 13 | 10 | 10 | 12 | 9 | 0 | 1 | 0 | 0 | 0 | 0 | 0 | 0 | 0 | 0 | 0 | 0 | 0 |
| s__Parabacteroides_timonensis | 0 | 0 | 0 | 0 | 0 | 0 | 0 | 0 | 0 | 0 | 0 | 0 | 0 | 0 | 1 | 0 | 40 | 0 |
| s__Paracoccus_alcaliphilus | 0 | 0 | 0 | 0 | 0 | 0 | 0 | 0 | 0 | 0 | 0 | 0 | 4 | 7 | 0 | 0 | 37 | 29 |
| s__Paramuribaculum_intestinale | 2 | 3 | 5 | 6 | 6 | 4 | 60 | 28 | 81 | 63 | 13 | 35 | 5 | 13 | 129 | 0 | 6 | 1 |
| s__Paraprevotella_clara | 0 | 0 | 1 | 7 | 2 | 1 | 0 | 0 | 0 | 0 | 0 | 0 | 0 | 0 | 0 | 0 | 0 | 0 |
| s__Parasutterella_excrementihominis | 19 | 78 | 196 | 70 | 91 | 18 | 0 | 0 | 0 | 1 | 0 | 1 | 0 | 1 | 0 | 0 | 0 | 0 |
| s__Parasutterella_sp000980495 | 24 | 114 | 175 | 129 | 62 | 18 | 0 | 0 | 0 | 1 | 0 | 0 | 1 | 0 | 0 | 0 | 0 | 0 |
| s__Pectobacterium_carotovorum_E | 0 | 2 | 2 | 0 | 0 | 2 | 0 | 0 | 2 | 5 | 2 | 0 | 0 | 1 | 0 | 1 | 1 | 0 |
| s__Pediococcus_acidilactici | 0 | 0 | 0 | 0 | 0 | 0 | 0 | 0 | 0 | 0 | 0 | 0 | 0 | 0 | 0 | 0 | 19 | 5 |
| s__Pedobacter_zeaxanthinifaciens | 0 | 0 | 0 | 0 | 0 | 0 | 2 | 10 | 2 | 31 | 13 | 7 | 0 | 0 | 5 | 0 | 0 | 0 |
| s__Pelagibacterium_luteolum | 0 | 0 | 0 | 0 | 0 | 0 | 0 | 0 | 0 | 0 | 0 | 0 | 2 | 1 | 0 | 0 | 3 | 1 |
| s__Phascolarctobacterium_faecium | 10 | 5 | 15 | 2 | 9 | 23 | 7 | 76 | 89 | 33 | 48 | 242 | 7 | 20 | 71 | 0 | 7 | 2 |
| s__Phycicoccus_elongata | 0 | 0 | 0 | 0 | 0 | 0 | 0 | 1 | 0 | 0 | 0 | 0 | 1 | 0 | 1 | 0 | 9 | 0 |
| s__Phyllobacterium_sp900473175 | 0 | 1 | 1 | 2 | 0 | 1 | 1 | 0 | 0 | 1 | 0 | 1 | 2 | 1 | 0 | 0 | 23 | 2 |
| s__Prevotella_conceptionensis | 1737 | 42 | 416 | 325 | 379 | 66 | 6336 | 313 | 109 | 1460 | 770 | 2766 | 70 | 358 | 255 | 0 | 9 | 43 |
| s__Prevotella_copri | 21 | 16 | 7 | 20 | 23 | 20 | 1184 | 222 | 386 | 506 | 2603 | 1232 | 59 | 162 | 109 | 0 | 5 | 7 |
| s__Prevotella_maculosa | 2 | 0 | 0 | 0 | 0 | 0 | 1 | 0 | 0 | 0 | 0 | 0 | 0 | 0 | 0 | 0 | 58 | 0 |
| s__Prevotella_multisaccharivorax | 0 | 0 | 0 | 0 | 0 | 0 | 0 | 0 | 0 | 0 | 0 | 0 | 0 | 0 | 0 | 0 | 52 | 0 |
| s__Prevotella_pleuritidis | 1 | 0 | 3 | 0 | 1 | 1 | 23 | 122 | 3 | 1 | 23 | 5 | 1 | 0 | 1 | 0 | 1 | 0 |
| s__Prevotella_sp000758925 | 0 | 0 | 0 | 0 | 0 | 0 | 0 | 0 | 1 | 0 | 0 | 0 | 0 | 0 | 0 | 0 | 9 | 0 |
| s__Prevotella_sp001275135 | 1 | 2 | 0 | 0 | 0 | 2 | 352 | 13 | 85 | 4 | 4 | 162 | 13 | 80 | 17 | 0 | 0 | 2 |
| s__Prevotella_sp002251365 | 2 | 0 | 0 | 1 | 2 | 1 | 769 | 20 | 10 | 49 | 100 | 54 | 1 | 7 | 10 | 0 | 15 | 0 |
| s__Prevotella_sp002251385 | 0 | 0 | 1 | 0 | 0 | 0 | 1 | 2 | 2 | 3 | 1 | 0 | 0 | 2 | 0 | 0 | 0 | 1 |
| s__Prevotella_sp002933775 | 1 | 1 | 0 | 0 | 1 | 1 | 266 | 7 | 7 | 5 | 57 | 5 | 0 | 2 | 5 | 0 | 0 | 1 |
| s__Prevotellamassilia_sp002933955 | 103 | 24 | 41 | 302 | 130 | 4 | 45 | 17 | 46 | 116 | 53 | 108 | 6 | 17 | 39 | 0 | 2 | 2 |
| s__Propionicicella_superfundia | 0 | 0 | 0 | 0 | 0 | 1 | 0 | 1 | 0 | 1 | 1 | 1 | 0 | 0 | 0 | 0 | 2 | 0 |
| s__Proteus_mirabilis | 4 | 12 | 18 | 3 | 10 | 11 | 5 | 11 | 10 | 11 | 12 | 13 | 6 | 15 | 12 | 4 | 20 | 4 |
| s__Provencibacterium_massiliense | 19 | 51 | 20 | 49 | 101 | 73 | 8 | 16 | 32 | 28 | 26 | 15 | 1 | 0 | 36 | 0 | 3 | 1 |
| s__Providencia_stuartii_B | 24 | 40 | 47 | 14 | 34 | 41 | 20 | 47 | 52 | 45 | 60 | 44 | 34 | 24 | 34 | 17 | 77 | 23 |
| s__Pseudochrobactrum_sp001939785 | 0 | 0 | 0 | 0 | 0 | 0 | 0 | 0 | 0 | 0 | 0 | 0 | 0 | 0 | 0 | 0 | 3 | 0 |
| s__Pseudoflavonifractor_capillosus | 4 | 4 | 6 | 4 | 10 | 40 | 1 | 0 | 4 | 1 | 1 | 5 | 0 | 0 | 1 | 0 | 0 | 0 |
| s__Pseudomonas_A_saudiphocaensis | 0 | 0 | 0 | 0 | 0 | 0 | 0 | 0 | 0 | 0 | 0 | 0 | 0 | 6 | 0 | 0 | 9 | 14 |
| s__Pseudomonas_E_anguilliseptica | 0 | 0 | 1 | 0 | 0 | 0 | 0 | 0 | 0 | 0 | 0 | 0 | 0 | 0 | 0 | 0 | 42 | 0 |
| s__Pseudomonas_E_sihuiensis | 0 | 2 | 0 | 2 | 3 | 1 | 1 | 10 | 7 | 1 | 13 | 6 | 1 | 2 | 4 | 2 | 3834 | 2 |
| s__Psychrobacter_pasteurii | 0 | 0 | 0 | 0 | 1 | 0 | 8 | 1 | 0 | 2 | 0 | 0 | 2 | 14 | 0 | 3 | 30 | 21 |
| s__Pusillimonas_noertemannii | 0 | 0 | 0 | 0 | 0 | 0 | 0 | 0 | 0 | 0 | 0 | 0 | 2 | 5 | 0 | 1 | 10 | 6 |
| s__Pusillimonas_sp002359265 | 0 | 0 | 0 | 0 | 0 | 0 | 0 | 0 | 0 | 0 | 0 | 0 | 1 | 1 | 0 | 1 | 14 | 17 |
| s__QAMH01_sp003149935 | 5 | 8 | 10 | 0 | 9 | 3 | 0 | 7 | 0 | 3 | 0 | 2 | 2 | 2 | 1 | 0 | 0 | 0 |
| s__QKVK01_sp003234965 | 0 | 0 | 1 | 1 | 2 | 0 | 0 | 0 | 3 | 0 | 2 | 2 | 0 | 0 | 0 | 0 | 1 | 1 |
| s__RC9_sp000434935 | 0 | 0 | 0 | 0 | 0 | 0 | 28 | 11 | 19 | 2 | 9 | 20 | 0 | 0 | 20 | 0 | 0 | 1 |
| s__RC9_sp002438635 | 0 | 1 | 0 | 0 | 0 | 1 | 27 | 21 | 37 | 30 | 7 | 24 | 1 | 2 | 32 | 0 | 4 | 0 |
| s__RC9_sp900167895 | 0 | 0 | 0 | 0 | 1 | 0 | 6 | 9 | 11 | 2 | 3 | 30 | 2 | 2 | 18 | 0 | 0 | 0 |
| s__RC9_sp900317925 | 0 | 0 | 0 | 0 | 0 | 0 | 77 | 23 | 63 | 29 | 26 | 126 | 2 | 9 | 58 | 0 | 4 | 0 |
| s__RUG754_sp900315895 | 6 | 1 | 4 | 1 | 12 | 2 | 0 | 13 | 10 | 11 | 4 | 1 | 0 | 0 | 5 | 0 | 0 | 0 |
| s__Ralstonia_sp001078575 | 0 | 0 | 0 | 0 | 0 | 0 | 0 | 0 | 0 | 0 | 1 | 0 | 0 | 6 | 0 | 0 | 9 | 3 |
| s__Raoultibacter_timonensis | 11 | 9 | 3 | 10 | 7 | 8 | 8 | 18 | 15 | 7 | 9 | 9 | 1 | 18 | 16 | 0 | 0 | 0 |
| s__Reyranella_sp001557035 | 1 | 0 | 0 | 0 | 1 | 0 | 0 | 0 | 0 | 0 | 1 | 0 | 2 | 3 | 0 | 0 | 12 | 0 |
| s__Rhizorhabdus_sp001015195 | 3 | 4 | 2 | 5 | 2 | 5 | 2 | 6 | 4 | 2 | 5 | 3 | 1 | 3 | 2 | 3 | 4 | 1 |
| s__Rikenella_massiliensis | 15 | 5 | 5 | 25 | 8 | 15 | 17 | 87 | 187 | 37 | 45 | 356 | 16 | 18 | 138 | 0 | 6 | 5 |
| s__Robinsoniella_peoriensis | 0 | 0 | 0 | 1 | 56 | 16 | 3 | 12 | 5 | 13 | 5 | 0 | 1 | 1 | 4 | 0 | 1 | 0 |
| s__Rodentibacter_heidelbergensis | 0 | 0 | 5 | 1 | 3 | 0 | 13 | 11 | 3 | 4 | 3 | 13 | 2 | 4 | 4 | 0 | 1 | 0 |
| s__Romboutsia_timonensis | 45 | 21 | 229 | 19 | 42 | 16 | 0 | 0 | 5 | 5 | 10 | 4 | 19 | 10 | 3 | 3 | 78 | 9 |
| s__Roseburia_hominis | 0 | 0 | 8 | 0 | 0 | 25 | 3 | 6 | 1 | 34 | 104 | 2 | 0 | 0 | 5 | 0 | 1 | 1 |
| s__Roseburia_intestinalis | 0 | 1 | 0 | 0 | 0 | 0 | 0 | 13 | 4 | 10 | 3 | 3 | 0 | 0 | 2 | 0 | 0 | 0 |
| s__Roseburia_sp001940165 | 0 | 0 | 0 | 0 | 0 | 0 | 0 | 1 | 8 | 0 | 0 | 24 | 0 | 1 | 9 | 0 | 0 | 0 |
| s__Rothia_nasimurium | 28 | 16 | 39 | 15 | 34 | 24 | 3 | 31 | 14 | 20 | 16 | 21 | 2503 | 1992 | 20 | 273 | 12142 | 7471 |
| s__Ruminiclostridium_A_cellulolyticum | 111 | 1 | 3 | 0 | 1 | 10 | 171 | 24 | 15 | 2 | 1 | 111 | 5 | 51 | 34 | 0 | 0 | 0 |
| s__Ruminiclostridium_A_sp000244875 | 0 | 0 | 1 | 0 | 0 | 0 | 494 | 0 | 1 | 0 | 0 | 0 | 0 | 0 | 0 | 0 | 0 | 0 |
| s__Ruminiclostridium_C_viride | 7 | 20 | 25 | 19 | 26 | 26 | 39 | 29 | 29 | 46 | 67 | 36 | 5 | 10 | 22 | 0 | 0 | 3 |
| s__Ruminiclostridium_D_cellulosi | 0 | 0 | 0 | 0 | 0 | 0 | 0 | 0 | 0 | 0 | 0 | 0 | 0 | 0 | 0 | 0 | 10 | 0 |
| s__Ruminiclostridium_E_siraeum | 25 | 44 | 138 | 6 | 370 | 113 | 74 | 384 | 698 | 56 | 1082 | 248 | 4 | 7 | 681 | 0 | 44 | 15 |
| s__Ruminiclostridium_thermocellum | 1 | 1 | 5 | 1 | 0 | 0 | 0 | 0 | 4 | 0 | 0 | 1 | 0 | 0 | 1 | 0 | 1 | 0 |
| s__Ruminococcus_A_sp003011855 | 2 | 1015 | 160 | 6 | 77 | 26 | 5 | 10 | 10 | 36 | 45 | 22 | 1 | 3 | 14 | 0 | 1 | 1 |
| s__Ruminococcus_C_callidus | 2 | 1 | 1 | 0 | 0 | 1 | 80 | 13 | 1 | 1 | 35 | 203 | 2 | 1 | 0 | 0 | 1 | 0 |
| s__Ruminococcus_C_sp000433635 | 168 | 34 | 56 | 10 | 35 | 12 | 7 | 75 | 56 | 21 | 52 | 68 | 2 | 11 | 56 | 0 | 6 | 1 |
| s__Ruminococcus_D_sp000686125 | 75 | 1 | 4 | 3 | 1 | 22 | 0 | 1 | 0 | 0 | 1 | 41 | 0 | 1 | 0 | 0 | 0 | 0 |
| s__Ruminococcus_D_sp900119155 | 0 | 18 | 2 | 0 | 2 | 85 | 6 | 21 | 67 | 11 | 76 | 68 | 0 | 1 | 71 | 0 | 2 | 3 |
| s__Ruminococcus_flavefaciens_E | 3 | 2 | 7 | 0 | 10 | 2 | 19 | 12 | 18 | 92 | 148 | 225 | 1 | 1 | 18 | 0 | 6 | 3 |
| s__Ruminococcus_flavefaciens_G | 1 | 0 | 0 | 0 | 4 | 1 | 19 | 3 | 17 | 0 | 204 | 0 | 1 | 0 | 19 | 0 | 0 | 0 |
| s__Ruthenibacterium_lactatiformans | 11 | 23 | 9 | 9 | 5 | 113 | 2 | 52 | 16 | 14 | 8 | 13 | 0 | 2 | 13 | 0 | 2 | 2 |
| s__SK-Y3_sp002252565 | 3 | 0 | 1 | 2 | 5 | 7 | 5 | 4 | 12 | 1 | 7 | 5 | 2 | 2 | 7 | 0 | 1 | 0 |
| s__SR-FBR-E99_sp002403305 | 3 | 3 | 5 | 2 | 5 | 5 | 2 | 0 | 4 | 2 | 3 | 2 | 1 | 1 | 1 | 1 | 0 | 0 |
| s__SZUA-55_sp003232855 | 0 | 0 | 0 | 0 | 0 | 0 | 0 | 0 | 0 | 0 | 0 | 0 | 8 | 1 | 0 | 0 | 1 | 6 |
| s__Saccharimonas_aalborgensis | 171 | 22 | 81 | 38 | 37 | 78 | 720 | 1100 | 1508 | 625 | 1043 | 1290 | 155 | 543 | 1300 | 9 | 87 | 72 |
| s__Salmonella_enterica | 2307 | 765 | 1294 | 663 | 1872 | 2016 | 944 | 1109 | 1448 | 1331 | 1271 | 1378 | 96 | 317 | 1143 | 9 | 181 | 82 |
| s__Savagella_sp000283555 | 4 | 0 | 4 | 0 | 0 | 0 | 0 | 1 | 0 | 1 | 0 | 0 | 0 | 0 | 1 | 0 | 0 | 0 |
| s__Schwartzia_succinivorans | 1 | 4 | 0 | 1 | 2 | 4 | 341 | 2197 | 617 | 1461 | 1392 | 565 | 76 | 234 | 316 | 3 | 54 | 20 |
| s__Serinibacter_sp003121705 | 3 | 1 | 0 | 1 | 1 | 1 | 0 | 2 | 3 | 1 | 5 | 4 | 1 | 0 | 2 | 2 | 3 | 2 |
| s__Solobacterium_sp900343155 | 0 | 0 | 0 | 0 | 0 | 0 | 0 | 0 | 0 | 0 | 0 | 0 | 0 | 0 | 0 | 0 | 8 | 0 |
| s__Sphingobacterium_mizutaii | 1 | 4 | 4 | 3 | 7 | 5 | 2 | 5 | 1 | 1 | 1 | 2 | 0 | 3 | 2 | 0 | 1 | 0 |
| s__Sphingobacterium_sp002980525 | 0 | 0 | 1 | 0 | 0 | 0 | 0 | 0 | 0 | 0 | 0 | 0 | 2 | 19 | 0 | 2 | 46 | 66 |
| s__Sphingobacterium_spiritivorum_A | 0 | 0 | 0 | 0 | 1 | 1 | 22 | 5 | 32 | 4 | 8 | 10 | 0 | 0 | 21 | 0 | 1 | 0 |
| s__Sphingomonas_A_sp003097155 | 9 | 12 | 9 | 8 | 8 | 21 | 8 | 21 | 11 | 23 | 23 | 22 | 4 | 12 | 13 | 3 | 30 | 8 |
| s__Sphingomonas_B_changbaiensis | 3 | 3 | 2 | 1 | 0 | 2 | 1 | 1 | 2 | 5 | 4 | 3 | 1 | 1 | 2 | 1 | 3 | 0 |
| s__Sphingopyxis_sp000756385 | 0 | 1 | 0 | 0 | 0 | 0 | 0 | 0 | 0 | 0 | 0 | 0 | 0 | 0 | 0 | 0 | 94 | 0 |
| s__Sporobacter_termitidis | 9 | 22 | 10 | 3 | 12 | 13 | 14 | 23 | 19 | 11 | 13 | 22 | 2 | 7 | 23 | 0 | 1 | 2 |
| s__Sporolactobacillus_inulinus | 0 | 0 | 0 | 0 | 0 | 0 | 0 | 0 | 0 | 0 | 1 | 0 | 0 | 0 | 0 | 0 | 17 | 0 |
| s__Staphylococcus_A_lentus | 1 | 0 | 0 | 1 | 0 | 3 | 0 | 0 | 0 | 0 | 0 | 0 | 38 | 5 | 0 | 1 | 88 | 10 |
| s__Staphylococcus_cohnii | 8 | 3 | 3 | 0 | 5 | 0 | 0 | 0 | 0 | 0 | 0 | 0 | 5 | 0 | 0 | 0 | 15 | 1 |
| s__Steroidobacter_denitrificans | 0 | 1 | 2 | 0 | 2 | 0 | 0 | 1 | 0 | 0 | 1 | 3 | 0 | 2 | 1 | 0 | 0 | 0 |
| s__Stoquefichus_sp001244545 | 15 | 7 | 6 | 1 | 10 | 24 | 0 | 1 | 0 | 0 | 0 | 0 | 0 | 0 | 0 | 0 | 0 | 0 |
| s__Streptococcus_caballi | 0 | 0 | 1 | 0 | 0 | 0 | 167 | 37 | 99 | 55 | 57 | 91 | 379 | 26 | 169 | 1 | 14 | 7 |
| s__Streptococcus_ferus | 0 | 0 | 0 | 0 | 0 | 0 | 1 | 3 | 1 | 1 | 1 | 1 | 1 | 3 | 0 | 4 | 19 | 3 |
| s__Streptococcus_parasanguinis_D | 7 | 2 | 11 | 0 | 1 | 2 | 4 | 0 | 1 | 1 | 2 | 2 | 2 | 4 | 2 | 22 | 639 | 300 |
| s__Streptococcus_ratti | 0 | 0 | 0 | 0 | 0 | 0 | 2 | 2 | 2 | 2 | 1 | 5 | 52 | 126 | 4 | 38 | 387 | 270 |
| s__Sutterella_wadsworthensis_B | 37 | 30 | 45 | 3 | 4 | 52 | 76 | 289 | 78 | 40 | 18 | 211 | 5 | 8 | 62 | 0 | 5 | 4 |
| s__Symbiobacterium_thermophilum | 0 | 3 | 1 | 3 | 1 | 2 | 2 | 4 | 1 | 2 | 1 | 2 | 0 | 0 | 1 | 2 | 3 | 1 |
| s__Syntrophobacter_fumaroxidans | 2 | 6 | 3 | 2 | 2 | 5 | 4 | 3 | 1 | 4 | 6 | 5 | 2 | 5 | 2 | 2 | 10 | 1 |
| s__T78_sp002347705 | 4 | 1 | 0 | 1 | 0 | 0 | 0 | 2 | 0 | 4 | 1 | 0 | 1 | 1 | 1 | 0 | 4 | 2 |
| s__TF01-11_sp001414325 | 1 | 9 | 2 | 3 | 10 | 18 | 5 | 24 | 60 | 28 | 69 | 25 | 3 | 3 | 29 | 0 | 3 | 2 |
| s__TWA4_sp000875945 | 2 | 25 | 6 | 5 | 9 | 7 | 0 | 9 | 5 | 9 | 10 | 2 | 0 | 0 | 4 | 0 | 1 | 0 |
| s__Tatlockia_lansingensis | 0 | 0 | 0 | 0 | 1 | 0 | 0 | 0 | 0 | 0 | 0 | 1 | 1 | 0 | 0 | 0 | 236 | 1 |
| s__Thermoflavifilum_aggregans | 1 | 0 | 5 | 3 | 3 | 0 | 1 | 2 | 0 | 3 | 1 | 1 | 0 | 1 | 0 | 0 | 1 | 1 |
| s__Thioalkalivibrio_A_denitrificans | 0 | 1 | 1 | 2 | 4 | 6 | 2 | 7 | 4 | 2 | 4 | 2 | 0 | 0 | 1 | 2 | 3 | 0 |
| s__Thiobacillus_denitrificans | 1 | 0 | 0 | 1 | 1 | 0 | 0 | 0 | 0 | 0 | 0 | 2 | 0 | 0 | 1 | 0 | 0 | 2 |
| s__Tissierella_A_creatinophila | 0 | 0 | 0 | 0 | 1 | 0 | 0 | 0 | 0 | 0 | 0 | 0 | 0 | 1 | 0 | 0 | 0 | 1 |
| s__Tolypothrix_B_campylonemoides | 0 | 0 | 0 | 0 | 1 | 3 | 0 | 1 | 2 | 2 | 0 | 1 | 1 | 0 | 1 | 0 | 1 | 0 |
| s__Treponema_D_bryantii_A | 1 | 1 | 1 | 0 | 0 | 2 | 51 | 607 | 2248 | 1188 | 531 | 793 | 5 | 18 | 892 | 0 | 16 | 5 |
| s__Treponema_D_sp002296965 | 0 | 0 | 0 | 0 | 2 | 0 | 39 | 193 | 218 | 228 | 67 | 195 | 2 | 4 | 99 | 0 | 3 | 1 |
| s__Treponema_D_sp900316905 | 4 | 8 | 21 | 16 | 596 | 1155 | 19 | 39 | 506 | 100 | 55 | 368 | 8 | 23 | 209 | 1 | 3 | 2 |
| s__Treponema_D_succinifaciens | 0 | 0 | 0 | 0 | 0 | 0 | 1 | 6 | 11 | 1 | 2 | 3 | 0 | 0 | 2 | 0 | 0 | 0 |
| s__Tyzzerella_nexilis | 4 | 1 | 8 | 1 | 15 | 4 | 9 | 2 | 29 | 13 | 18 | 38 | 2 | 7 | 55 | 0 | 4 | 0 |
| s__UBA1033_sp001695555 | 2 | 3 | 1 | 2 | 4 | 1 | 0 | 0 | 0 | 0 | 0 | 0 | 0 | 0 | 0 | 0 | 0 | 0 |
| s__UBA1174_sp002311025 | 0 | 0 | 1 | 4 | 1 | 1 | 0 | 0 | 0 | 1 | 0 | 1 | 0 | 0 | 0 | 0 | 0 | 0 |
| s__UBA1191_sp900066305 | 3 | 9 | 8 | 0 | 8 | 16 | 4 | 11 | 21 | 11 | 12 | 18 | 12 | 13 | 10 | 0 | 2 | 3 |
| s__UBA12465_sp001871165 | 0 | 0 | 0 | 0 | 0 | 1 | 3 | 1 | 0 | 0 | 0 | 1 | 0 | 0 | 0 | 0 | 0 | 1 |
| s__UBA1394_sp900066845 | 41 | 90 | 1 | 0 | 2 | 0 | 11 | 47 | 76 | 51 | 16 | 64 | 1 | 0 | 127 | 0 | 4 | 2 |
| s__UBA1436_sp002159705 | 1 | 0 | 1 | 5 | 0 | 8 | 2 | 0 | 0 | 0 | 0 | 5 | 1 | 0 | 1 | 0 | 2 | 0 |
| s__UBA1547_sp002413425 | 0 | 0 | 0 | 0 | 0 | 0 | 141 | 138 | 84 | 23 | 121 | 148 | 19 | 80 | 66 | 1 | 5 | 6 |
| s__UBA1711_sp001543385 | 0 | 0 | 1 | 0 | 0 | 0 | 92 | 16 | 15 | 63 | 44 | 24 | 3 | 5 | 25 | 0 | 1 | 0 |
| s__UBA1777_sp900319835 | 0 | 1 | 1 | 0 | 1 | 0 | 96 | 61 | 41 | 69 | 67 | 54 | 7 | 18 | 46 | 0 | 1 | 0 |
| s__UBA2212_sp002402585 | 22 | 12 | 53 | 15 | 2 | 14 | 4 | 2 | 6 | 2 | 0 | 3 | 0 | 3 | 4 | 0 | 0 | 1 |
| s__UBA2365_sp002344345 | 0 | 0 | 0 | 0 | 0 | 0 | 0 | 0 | 0 | 0 | 0 | 0 | 0 | 0 | 0 | 0 | 12 | 0 |
| s__UBA2730_sp900320505 | 53 | 30 | 78 | 4 | 3 | 163 | 113 | 78 | 117 | 6 | 0 | 239 | 23 | 139 | 82 | 0 | 4 | 3 |
| s__UBA3006_sp002367695 | 2 | 2 | 1 | 1 | 2 | 2 | 2 | 3 | 1 | 1 | 2 | 2 | 8 | 2 | 0 | 1 | 7 | 2 |
| s__UBA4782_sp002403095 | 1 | 7 | 5 | 3 | 3 | 2 | 1 | 4 | 6 | 4 | 5 | 4 | 3 | 6 | 1 | 6 | 4 | 2 |
| s__UBA6382_sp900316315 | 0 | 0 | 0 | 0 | 0 | 0 | 0 | 0 | 0 | 0 | 0 | 0 | 0 | 0 | 0 | 0 | 24 | 0 |
| s__UBA6398_sp002451695 | 0 | 0 | 0 | 0 | 13 | 2 | 0 | 0 | 0 | 0 | 0 | 0 | 0 | 0 | 0 | 0 | 0 | 0 |
| s__UBA6659_sp002344135 | 1 | 1 | 2 | 0 | 2 | 1 | 0 | 1 | 0 | 0 | 1 | 0 | 1 | 0 | 0 | 0 | 0 | 1 |
| s__UBA6985_sp900314465 | 44 | 0 | 101 | 0 | 2 | 3 | 115 | 33 | 56 | 58 | 10 | 55 | 4 | 11 | 46 | 2 | 0 | 1 |
| s__UBA7182_sp002160135 | 0 | 0 | 0 | 0 | 0 | 0 | 0 | 0 | 1 | 1 | 0 | 1 | 0 | 0 | 0 | 0 | 0 | 0 |
| s__UBA8950_sp001872455 | 1 | 0 | 1 | 0 | 0 | 1 | 0 | 0 | 0 | 2 | 1 | 3 | 1 | 1 | 0 | 1 | 0 | 0 |
| s__UBA945_sp900197595 | 0 | 0 | 0 | 0 | 0 | 0 | 0 | 3 | 13 | 1 | 8 | 21 | 0 | 3 | 8 | 0 | 2 | 0 |
| s__UC5-1-2E3_sp001304875 | 3 | 15 | 8 | 9 | 120 | 144 | 3 | 21 | 29 | 56 | 34 | 18 | 0 | 12 | 15 | 0 | 4 | 5 |
| s__URHD0088_sp000518365 | 0 | 0 | 0 | 1 | 0 | 0 | 0 | 1 | 0 | 2 | 0 | 2 | 0 | 1 | 0 | 0 | 0 | 0 |
| s__UTCFX2_sp002050125 | 3 | 4 | 3 | 2 | 1 | 4 | 0 | 4 | 3 | 1 | 7 | 6 | 4 | 7 | 3 | 3 | 6 | 3 |
| s__Vagococcus_sp002140795 | 7 | 1 | 0 | 0 | 4 | 1 | 4 | 1 | 2 | 0 | 1 | 1 | 5 | 3 | 1 | 6 | 3 | 7 |
| s__Vallitalea_A_okinawensis | 5 | 14 | 2 | 3 | 7 | 1 | 0 | 0 | 0 | 1 | 0 | 0 | 0 | 0 | 0 | 0 | 0 | 0 |
| s__Vallitalea_guaymasensis | 127 | 93 | 199 | 2 | 71 | 105 | 27 | 3 | 2 | 0 | 2 | 19 | 6 | 14 | 0 | 0 | 1 | 1 |
| s__Veillonella_rogosae | 0 | 0 | 0 | 0 | 0 | 0 | 4 | 0 | 1 | 2 | 0 | 4 | 0 | 1 | 0 | 0 | 0 | 0 |
| s__Vibrio_parahaemolyticus | 10022 | 5347 | 10250 | 8790 | 8905 | 12466 | 4103 | 1338 | 1759 | 1563 | 2173 | 1783 | 167 | 432 | 1955 | 3 | 99 | 65 |
| s__Virgibacillus_G_profundi | 3 | 33 | 33 | 21 | 616 | 177 | 4 | 130 | 22 | 156 | 47 | 17 | 1 | 4 | 23 | 0 | 3 | 2 |
| s__Virgibacillus_picturae | 0 | 0 | 0 | 0 | 0 | 0 | 0 | 0 | 0 | 0 | 0 | 0 | 8 | 26 | 0 | 1 | 65 | 30 |
| s__W-Firmicutes-11_sp002840285 | 7 | 27 | 17 | 13 | 17 | 20 | 14 | 39 | 27 | 31 | 23 | 29 | 2 | 8 | 23 | 0 | 2 | 3 |
| s__Weissella_paramesenteroides | 6 | 6 | 4 | 5 | 3 | 2 | 6 | 5 | 9 | 10 | 3 | 9 | 19 | 13 | 6 | 18 | 12 | 8 |
| s__Yaniella_halotolerans | 0 | 0 | 0 | 0 | 0 | 0 | 0 | 0 | 0 | 0 | 0 | 1 | 3 | 0 | 0 | 0 | 17 | 11 |
| s__ZCTH02-B6_sp002159155 | 0 | 0 | 0 | 0 | 0 | 0 | 0 | 0 | 0 | 0 | 0 | 0 | 1 | 0 | 1 | 0 | 0 | 0 |
| s__Zag111_sp003258735 | 0 | 0 | 0 | 0 | 0 | 1 | 1 | 2 | 1 | 3 | 4 | 1 | 0 | 2 | 3 | 0 | 0 | 0 |
| s__Zag1_sp001765415 | 0 | 1 | 0 | 0 | 1 | 0 | 68 | 24 | 5 | 9 | 25 | 26 | 1 | 17 | 20 | 0 | 3 | 0 |
| s__unclassified | 2 | 0 | 0 | 0 | 14 | 0 | 0 | 2 | 2 | 2 | 2 | 1 | 4 | 0 | 1 | 4 | 7 | 4 |

# S7

## OTUs count for Fig 7L

| otu | A1 | A2 | A3 | A4 | A5 | A6 | B1 | B2 | B3 | B4 | B5 | B6 | C1 | C2 | C3 | C4 | C5 | C6 |
| --- | --- | --- | --- | --- | --- | --- | --- | --- | --- | --- | --- | --- | --- | --- | --- | --- | --- | --- |
| OTU1 | 5167 | 1361 | 2374 | 4451 | 1528 | 1256 | 6036 | 6655 | 5114 | 8144 | 4276 | 5343 | 10474 | 1837 | 9951 | 7392 | 846 | 34899 |
| OTU10 | 1864 | 610 | 720 | 546 | 1298 | 1372 | 91 | 128 | 205 | 101 | 85 | 114 | 13 | 46 | 155 | 0 | 11 | 10 |
| OTU100 | 0 | 6 | 8 | 0 | 1 | 5 | 0 | 50 | 138 | 17 | 89 | 195 | 15 | 20 | 86 | 0 | 8 | 1 |
| OTU101 | 3 | 32 | 0 | 20 | 599 | 7 | 3 | 46 | 16 | 128 | 32 | 5 | 1 | 2 | 17 | 0 | 3 | 2 |
| OTU102 | 0 | 1 | 1 | 0 | 0 | 0 | 1 | 27 | 93 | 1 | 0 | 205 | 20 | 117 | 62 | 0 | 1 | 0 |
| OTU103 | 166 | 102 | 283 | 215 | 83 | 230 | 88 | 114 | 47 | 92 | 42 | 92 | 10 | 26 | 44 | 0 | 9 | 5 |
| OTU104 | 1 | 0 | 1 | 2 | 0 | 1 | 163 | 45 | 77 | 59 | 131 | 79 | 8 | 22 | 111 | 0 | 2 | 1 |
| OTU105 | 6 | 1 | 369 | 0 | 1 | 1 | 8 | 0 | 3 | 7 | 1 | 19 | 1 | 3 | 11 | 1 | 0 | 0 |
| OTU106 | 0 | 0 | 1 | 0 | 2 | 1 | 32 | 30 | 105 | 57 | 37 | 100 | 9 | 29 | 35 | 1 | 2 | 1 |
| OTU107 | 92 | 18 | 105 | 46 | 22 | 283 | 0 | 1 | 0 | 1 | 0 | 0 | 0 | 0 | 1 | 0 | 0 | 2 |
| OTU108 | 1 | 2 | 0 | 0 | 0 | 2 | 352 | 13 | 85 | 4 | 4 | 162 | 13 | 80 | 17 | 0 | 0 | 2 |
| OTU109 | 74 | 33 | 33 | 83 | 31 | 129 | 14 | 8 | 14 | 13 | 16 | 9 | 4 | 11 | 19 | 0 | 0 | 1 |
| OTU11 | 1 | 1 | 1 | 0 | 0 | 2 | 51 | 607 | 2248 | 1188 | 531 | 793 | 5 | 18 | 892 | 0 | 16 | 5 |
| OTU110 | 0 | 0 | 2 | 0 | 3 | 0 | 41 | 17 | 46 | 112 | 51 | 108 | 6 | 17 | 39 | 0 | 2 | 1 |
| OTU111 | 0 | 1 | 1 | 0 | 0 | 0 | 2 | 46 | 76 | 51 | 16 | 64 | 0 | 0 | 126 | 0 | 4 | 2 |
| OTU112 | 24 | 40 | 47 | 14 | 34 | 41 | 20 | 47 | 52 | 45 | 60 | 44 | 34 | 24 | 34 | 17 | 77 | 23 |
| OTU113 | 75 | 26 | 0 | 8 | 1 | 407 | 0 | 2 | 3 | 1 | 1 | 2 | 0 | 0 | 0 | 0 | 2 | 0 |
| OTU114 | 19 | 78 | 196 | 70 | 91 | 18 | 0 | 0 | 0 | 1 | 0 | 1 | 0 | 1 | 0 | 0 | 0 | 0 |
| OTU115 | 1 | 1 | 1 | 1 | 2 | 1 | 0 | 2 | 3 | 2 | 0 | 0 | 26 | 19 | 2 | 6 | 293 | 159 |
| OTU116 | 35 | 3 | 8 | 67 | 1 | 30 | 44 | 82 | 107 | 3 | 2 | 46 | 6 | 24 | 94 | 0 | 13 | 2 |
| OTU117 | 8 | 159 | 58 | 0 | 0 | 1 | 163 | 42 | 108 | 63 | 328 | 73 | 26 | 53 | 79 | 0 | 9 | 8 |
| OTU118 | 3 | 18 | 9 | 20 | 12 | 215 | 8 | 51 | 15 | 123 | 60 | 15 | 0 | 2 | 14 | 0 | 4 | 2 |
| OTU119 | 2 | 1 | 1 | 0 | 0 | 1 | 80 | 13 | 1 | 1 | 35 | 203 | 2 | 1 | 0 | 0 | 1 | 0 |
| OTU12 | 1 | 4 | 0 | 1 | 2 | 4 | 341 | 2197 | 617 | 1461 | 1392 | 565 | 76 | 234 | 316 | 3 | 54 | 20 |
| OTU120 | 75 | 118 | 21 | 111 | 162 | 210 | 3 | 1 | 2 | 1 | 2 | 2 | 1 | 1 | 0 | 0 | 0 | 0 |
| OTU121 | 0 | 0 | 0 | 0 | 0 | 0 | 0 | 0 | 0 | 0 | 0 | 0 | 0 | 0 | 0 | 0 | 297 | 0 |
| OTU122 | 34 | 26 | 69 | 69 | 158 | 192 | 1 | 4 | 0 | 25 | 0 | 1 | 0 | 2 | 0 | 0 | 0 | 1 |
| OTU123 | 1 | 0 | 2 | 1 | 3 | 0 | 78 | 20 | 36 | 40 | 29 | 87 | 6 | 10 | 53 | 0 | 0 | 0 |
| OTU124 | 1 | 2 | 1 | 3 | 1 | 5 | 40 | 103 | 25 | 58 | 83 | 80 | 7 | 30 | 26 | 0 | 2 | 6 |
| OTU125 | 9 | 14 | 4 | 5 | 348 | 9 | 1 | 0 | 0 | 0 | 0 | 1 | 0 | 0 | 0 | 0 | 0 | 0 |
| OTU126 | 0 | 0 | 2 | 0 | 1 | 1 | 133 | 25 | 13 | 86 | 32 | 32 | 9 | 13 | 8 | 0 | 1 | 1 |
| OTU127 | 2 | 3 | 73 | 14 | 4 | 75 | 8 | 61 | 20 | 50 | 384 | 15 | 8 | 4 | 18 | 1 | 4 | 6 |
| OTU128 | 1 | 99 | 6 | 28 | 0 | 180 | 31 | 28 | 36 | 7 | 14 | 13 | 3 | 20 | 46 | 0 | 4 | 1 |
| OTU129 | 1 | 2 | 6 | 3 | 0 | 22 | 3 | 31 | 12 | 265 | 35 | 34 | 1 | 3 | 55 | 0 | 1 | 2 |
| OTU13 | 2 | 14 | 13 | 3 | 10 | 137 | 5 | 505 | 1538 | 23 | 961 | 862 | 23 | 53 | 939 | 2 | 49 | 31 |
| OTU130 | 0 | 2 | 0 | 0 | 0 | 1 | 42 | 78 | 22 | 25 | 136 | 84 | 3 | 6 | 27 | 0 | 8 | 4 |
| OTU131 | 0 | 2 | 2 | 1 | 5 | 9 | 46 | 221 | 457 | 396 | 203 | 141 | 6 | 12 | 362 | 0 | 19 | 10 |
| OTU132 | 1 | 2 | 2 | 3 | 0 | 1 | 0 | 1 | 3 | 2 | 1 | 2 | 111 | 154 | 1 | 6 | 461 | 242 |
| OTU133 | 7 | 31 | 7 | 10 | 22 | 2 | 7 | 75 | 56 | 21 | 52 | 68 | 2 | 11 | 56 | 0 | 6 | 1 |
| OTU134 | 108 | 63 | 72 | 107 | 113 | 203 | 4 | 1 | 1 | 1 | 1 | 3 | 0 | 0 | 0 | 0 | 1 | 0 |
| OTU135 | 2 | 0 | 0 | 1 | 2 | 1 | 766 | 15 | 10 | 49 | 100 | 50 | 1 | 7 | 10 | 0 | 0 | 0 |
| OTU136 | 0 | 0 | 0 | 0 | 0 | 0 | 19 | 3 | 17 | 0 | 204 | 0 | 1 | 0 | 19 | 0 | 0 | 0 |
| OTU137 | 1 | 1 | 2 | 1 | 0 | 0 | 63 | 21 | 48 | 30 | 34 | 76 | 1 | 9 | 59 | 0 | 0 | 0 |
| OTU138 | 29 | 168 | 39 | 7 | 13 | 28 | 51 | 59 | 110 | 44 | 137 | 51 | 11 | 16 | 192 | 0 | 15 | 15 |
| OTU139 | 45 | 21 | 229 | 19 | 42 | 16 | 0 | 0 | 5 | 5 | 10 | 4 | 19 | 10 | 3 | 3 | 78 | 9 |
| OTU14 | 9 | 3 | 7 | 8 | 13 | 13 | 1736 | 826 | 463 | 895 | 651 | 1178 | 73 | 294 | 302 | 1 | 62 | 33 |
| OTU140 | 12 | 32 | 23 | 9 | 100 | 27 | 11 | 105 | 29 | 57 | 7 | 13 | 2 | 3 | 17 | 0 | 3 | 0 |
| OTU141 | 14 | 22 | 16 | 6 | 233 | 105 | 3 | 59 | 15 | 31 | 29 | 14 | 0 | 9 | 5 | 1 | 1 | 1 |
| OTU142 | 0 | 160 | 39 | 0 | 73 | 10 | 7 | 0 | 0 | 1 | 11 | 1 | 3 | 0 | 1 | 0 | 0 | 0 |
| OTU143 | 73 | 0 | 3 | 0 | 1 | 2 | 171 | 24 | 15 | 2 | 1 | 111 | 5 | 51 | 34 | 0 | 0 | 0 |
| OTU144 | 0 | 1 | 1 | 0 | 1 | 0 | 92 | 56 | 36 | 36 | 63 | 47 | 7 | 16 | 41 | 0 | 1 | 0 |
| OTU145 | 10 | 13 | 23 | 21 | 28 | 62 | 11 | 162 | 96 | 164 | 145 | 55 | 5 | 6 | 94 | 0 | 9 | 2 |
| OTU146 | 246 | 16 | 83 | 100 | 65 | 132 | 0 | 0 | 0 | 1 | 2 | 1 | 0 | 0 | 2 | 0 | 2 | 2 |
| OTU147 | 1 | 74 | 51 | 19 | 7 | 10 | 6 | 100 | 31 | 197 | 60 | 11 | 2 | 2 | 31 | 0 | 3 | 2 |
| OTU148 | 28 | 2 | 74 | 55 | 48 | 365 | 4 | 58 | 29 | 55 | 132 | 6 | 7 | 2 | 25 | 1 | 4 | 4 |
| OTU149 | 0 | 2 | 1 | 0 | 1 | 1 | 7 | 54 | 71 | 12 | 26 | 15 | 0 | 0 | 56 | 0 | 5 | 3 |
| OTU15 | 0 | 2 | 0 | 2 | 3 | 1 | 1 | 10 | 7 | 1 | 13 | 6 | 1 | 2 | 4 | 2 | 3834 | 2 |
| OTU150 | 0 | 0 | 1 | 0 | 0 | 0 | 494 | 0 | 1 | 0 | 0 | 0 | 0 | 0 | 0 | 0 | 0 | 0 |
| OTU151 | 41 | 41 | 85 | 35 | 21 | 47 | 3 | 17 | 8 | 4 | 7 | 5 | 1 | 3 | 4 | 0 | 2 | 5 |
| OTU152 | 5 | 26 | 34 | 38 | 59 | 43 | 3 | 75 | 27 | 74 | 87 | 44 | 5 | 8 | 10 | 0 | 5 | 0 |
| OTU153 | 0 | 0 | 0 | 0 | 0 | 0 | 0 | 0 | 0 | 0 | 0 | 0 | 34 | 18 | 0 | 5 | 95 | 127 |
| OTU154 | 0 | 0 | 0 | 0 | 1 | 0 | 0 | 0 | 0 | 0 | 0 | 1 | 1 | 0 | 0 | 0 | 236 | 1 |
| OTU155 | 45 | 49 | 53 | 26 | 20 | 76 | 0 | 0 | 0 | 0 | 0 | 0 | 0 | 0 | 0 | 0 | 0 | 0 |
| OTU156 | 0 | 1 | 0 | 0 | 1 | 0 | 63 | 24 | 5 | 8 | 24 | 24 | 1 | 17 | 19 | 0 | 0 | 0 |
| OTU157 | 1 | 1 | 284 | 0 | 2 | 5 | 6 | 1 | 2 | 1 | 6 | 0 | 0 | 0 | 2 | 0 | 1 | 0 |
| OTU158 | 0 | 0 | 1 | 1 | 2 | 0 | 33 | 30 | 34 | 1 | 2 | 86 | 9 | 34 | 33 | 0 | 5 | 1 |
| OTU159 | 3 | 4 | 19 | 5 | 647 | 55 | 1 | 46 | 23 | 48 | 43 | 22 | 0 | 3 | 24 | 0 | 4 | 5 |
| OTU16 | 0 | 2 | 4 | 2 | 3 | 9 | 141 | 413 | 755 | 684 | 654 | 1045 | 50 | 204 | 530 | 2 | 75 | 28 |
| OTU160 | 1 | 6 | 1 | 0 | 1 | 2 | 75 | 195 | 114 | 396 | 465 | 101 | 18 | 19 | 99 | 1 | 16 | 10 |
| OTU161 | 118 | 2 | 1 | 1 | 0 | 3 | 173 | 31 | 10 | 49 | 29 | 50 | 4 | 22 | 22 | 0 | 2 | 4 |
| OTU162 | 18 | 20 | 19 | 6 | 16 | 20 | 13 | 28 | 26 | 26 | 42 | 32 | 8 | 10 | 19 | 1 | 28 | 7 |
| OTU163 | 0 | 0 | 0 | 0 | 0 | 0 | 30 | 16 | 41 | 7 | 16 | 76 | 0 | 2 | 36 | 0 | 4 | 0 |
| OTU164 | 88 | 1 | 30 | 0 | 0 | 0 | 56 | 95 | 31 | 12 | 2 | 5 | 0 | 2 | 23 | 0 | 1 | 2 |
| OTU165 | 457 | 35 | 75 | 25 | 194 | 21 | 208 | 81 | 61 | 103 | 51 | 92 | 12 | 64 | 72 | 0 | 10 | 9 |
| OTU166 | 0 | 0 | 2 | 0 | 0 | 2 | 197 | 6 | 43 | 21 | 26 | 40 | 0 | 4 | 48 | 0 | 2 | 1 |
| OTU167 | 0 | 0 | 6 | 0 | 0 | 1 | 0 | 6 | 1 | 189 | 84 | 0 | 0 | 0 | 1 | 0 | 6 | 1 |
| OTU168 | 96 | 13 | 32 | 42 | 107 | 66 | 0 | 1 | 0 | 1 | 1 | 1 | 0 | 0 | 1 | 0 | 0 | 0 |
| OTU169 | 29 | 67 | 88 | 31 | 58 | 78 | 40 | 83 | 49 | 74 | 90 | 67 | 40 | 55 | 52 | 35 | 157 | 42 |
| OTU17 | 76 | 2145 | 1778 | 108 | 38 | 1061 | 1201 | 190 | 131 | 8 | 6 | 538 | 20 | 80 | 139 | 1 | 21 | 5 |
| OTU170 | 1 | 1 | 0 | 0 | 1 | 1 | 266 | 7 | 7 | 5 | 57 | 5 | 0 | 2 | 5 | 0 | 0 | 1 |
| OTU171 | 0 | 0 | 0 | 0 | 0 | 0 | 0 | 2 | 16 | 43 | 119 | 25 | 0 | 0 | 14 | 0 | 7 | 0 |
| OTU172 | 50 | 16 | 28 | 33 | 89 | 77 | 1 | 0 | 0 | 6 | 0 | 1 | 0 | 0 | 0 | 0 | 2 | 1 |
| OTU173 | 1 | 6 | 2 | 3 | 1 | 1 | 5 | 53 | 19 | 32 | 67 | 13 | 0 | 1 | 15 | 0 | 3 | 1 |
| OTU174 | 1 | 3 | 1 | 2 | 9 | 9 | 4 | 63 | 32 | 23 | 27 | 21 | 5 | 7 | 25 | 0 | 2 | 2 |
| OTU175 | 3 | 0 | 3 | 1 | 1 | 4 | 63 | 26 | 39 | 52 | 32 | 36 | 2 | 11 | 36 | 0 | 1 | 0 |
| OTU176 | 1 | 1 | 6 | 0 | 1 | 5 | 1 | 4 | 3 | 154 | 119 | 30 | 0 | 6 | 4 | 0 | 1 | 0 |
| OTU177 | 11 | 9 | 3 | 10 | 7 | 8 | 8 | 18 | 15 | 7 | 9 | 9 | 1 | 18 | 16 | 0 | 0 | 0 |
| OTU178 | 8 | 77 | 37 | 0 | 7 | 1 | 0 | 0 | 0 | 0 | 0 | 0 | 0 | 0 | 0 | 0 | 0 | 1 |
| OTU179 | 104 | 12 | 82 | 2 | 1 | 17 | 194 | 26 | 44 | 6 | 1 | 51 | 4 | 29 | 49 | 0 | 1 | 1 |
| OTU18 | 4 | 8 | 21 | 16 | 596 | 1155 | 19 | 39 | 506 | 100 | 55 | 368 | 8 | 23 | 209 | 1 | 3 | 2 |
| OTU180 | 0 | 0 | 0 | 0 | 0 | 0 | 24 | 32 | 40 | 30 | 73 | 29 | 9 | 22 | 60 | 0 | 4 | 1 |
| OTU181 | 0 | 0 | 0 | 0 | 0 | 0 | 1 | 3 | 11 | 30 | 2 | 57 | 4 | 6 | 20 | 0 | 0 | 0 |
| OTU182 | 3 | 6 | 6 | 2 | 29 | 2 | 13 | 87 | 30 | 87 | 84 | 29 | 2 | 6 | 18 | 0 | 3 | 2 |
| OTU183 | 25 | 63 | 64 | 53 | 115 | 41 | 2 | 0 | 0 | 0 | 1 | 1 | 0 | 1 | 0 | 0 | 3 | 0 |
| OTU184 | 159 | 2 | 47 | 0 | 10 | 7 | 0 | 0 | 0 | 0 | 0 | 0 | 0 | 0 | 0 | 0 | 0 | 0 |
| OTU185 | 15 | 53 | 34 | 22 | 71 | 73 | 18 | 63 | 62 | 81 | 72 | 35 | 5 | 13 | 39 | 0 | 1 | 0 |
| OTU186 | 1 | 0 | 3 | 0 | 1 | 1 | 23 | 122 | 3 | 1 | 23 | 5 | 1 | 0 | 1 | 0 | 1 | 0 |
| OTU187 | 24 | 30 | 16 | 84 | 69 | 53 | 51 | 371 | 208 | 208 | 168 | 252 | 7 | 13 | 117 | 1 | 11 | 7 |
| OTU188 | 9 | 1 | 14 | 0 | 0 | 9 | 0 | 12 | 52 | 2 | 7 | 217 | 1 | 12 | 42 | 0 | 2 | 0 |
| OTU189 | 4 | 9 | 8 | 1 | 71 | 72 | 2 | 1 | 8 | 9 | 14 | 14 | 0 | 2 | 5 | 0 | 3 | 0 |
| OTU19 | 29 | 277 | 124 | 17 | 380 | 177 | 14 | 50 | 46 | 27 | 6 | 12 | 561 | 1157 | 59 | 1524 | 11 | 2 |
| OTU190 | 86 | 25 | 85 | 53 | 31 | 65 | 14 | 2 | 1 | 8 | 3 | 13 | 0 | 4 | 6 | 0 | 1 | 1 |
| OTU191 | 0 | 3 | 0 | 2 | 410 | 5 | 1 | 5 | 3 | 4 | 3 | 1 | 0 | 0 | 4 | 0 | 1 | 1 |
| OTU192 | 2 | 1 | 0 | 1 | 1 | 1 | 164 | 3 | 12 | 60 | 56 | 1 | 1 | 2 | 13 | 0 | 7 | 4 |
| OTU193 | 11 | 8 | 58 | 0 | 0 | 11 | 116 | 89 | 22 | 102 | 131 | 88 | 17 | 30 | 26 | 1 | 5 | 7 |
| OTU194 | 9 | 13 | 146 | 4 | 2 | 20 | 0 | 0 | 0 | 0 | 0 | 1 | 0 | 0 | 0 | 0 | 0 | 1 |
| OTU195 | 125 | 5 | 24 | 105 | 0 | 74 | 36 | 21 | 14 | 10 | 8 | 4 | 2 | 6 | 20 | 0 | 3 | 1 |
| OTU196 | 42 | 9 | 37 | 22 | 40 | 56 | 1 | 6 | 6 | 13 | 4 | 0 | 0 | 0 | 3 | 0 | 0 | 0 |
| OTU197 | 0 | 0 | 1 | 1 | 1 | 2 | 20 | 2 | 21 | 20 | 26 | 35 | 8 | 19 | 9 | 0 | 1 | 1 |
| OTU198 | 2 | 3 | 5 | 6 | 6 | 4 | 56 | 25 | 80 | 54 | 6 | 33 | 2 | 13 | 121 | 0 | 6 | 1 |
| OTU199 | 1 | 0 | 1 | 2 | 0 | 1 | 22 | 9 | 12 | 52 | 29 | 23 | 1 | 5 | 19 | 0 | 0 | 0 |
| OTU2 | 381 | 3229 | 1307 | 215 | 4385 | 2353 | 184 | 675 | 492 | 406 | 103 | 144 | 6828 | 16355 | 677 | 20497 | 208 | 107 |
| OTU20 | 3 | 2 | 1 | 1 | 1 | 3 | 53 | 149 | 730 | 349 | 177 | 328 | 9 | 37 | 378 | 0 | 21 | 12 |
| OTU200 | 2 | 0 | 0 | 3 | 2 | 0 | 125 | 23 | 65 | 41 | 34 | 46 | 5 | 10 | 49 | 0 | 3 | 3 |
| OTU201 | 0 | 0 | 1 | 1 | 0 | 0 | 116 | 45 | 34 | 61 | 29 | 85 | 5 | 10 | 21 | 0 | 7 | 3 |
| OTU202 | 0 | 0 | 1 | 0 | 0 | 0 | 25 | 11 | 20 | 85 | 57 | 27 | 1 | 3 | 10 | 0 | 2 | 2 |
| OTU203 | 0 | 0 | 0 | 0 | 0 | 0 | 6 | 27 | 13 | 21 | 7 | 42 | 2 | 8 | 6 | 0 | 1 | 1 |
| OTU204 | 0 | 0 | 0 | 0 | 0 | 1 | 0 | 0 | 0 | 0 | 1 | 1 | 24 | 13 | 1 | 0 | 202 | 11 |
| OTU205 | 26 | 23 | 29 | 62 | 52 | 99 | 2 | 0 | 1 | 0 | 1 | 0 | 0 | 0 | 0 | 0 | 0 | 0 |
| OTU206 | 133 | 1 | 7 | 1 | 3 | 9 | 1 | 0 | 0 | 0 | 0 | 0 | 0 | 0 | 1 | 0 | 2 | 0 |
| OTU207 | 33 | 14 | 61 | 0 | 0 | 77 | 12 | 1 | 2 | 0 | 0 | 4 | 0 | 1 | 1 | 0 | 1 | 2 |
| OTU208 | 8 | 17 | 15 | 31 | 7 | 72 | 0 | 0 | 0 | 0 | 0 | 0 | 0 | 0 | 0 | 0 | 0 | 1 |
| OTU209 | 1 | 1 | 3 | 0 | 0 | 233 | 0 | 0 | 1 | 1 | 1 | 1 | 0 | 0 | 0 | 0 | 0 | 0 |
| OTU21 | 178 | 206 | 163 | 161 | 247 | 231 | 192 | 377 | 279 | 335 | 225 | 254 | 580 | 596 | 340 | 749 | 219 | 385 |
| OTU210 | 2 | 0 | 2 | 0 | 0 | 1 | 59 | 8 | 33 | 80 | 32 | 33 | 2 | 8 | 30 | 0 | 3 | 3 |
| OTU211 | 1 | 0 | 0 | 0 | 0 | 0 | 0 | 0 | 1 | 0 | 0 | 1 | 17 | 7 | 0 | 2 | 121 | 53 |
| OTU212 | 41 | 89 | 0 | 0 | 2 | 0 | 9 | 1 | 0 | 0 | 0 | 0 | 1 | 0 | 1 | 0 | 0 | 0 |
| OTU213 | 75 | 19 | 59 | 51 | 2 | 209 | 6 | 6 | 10 | 6 | 2 | 4 | 0 | 1 | 3 | 0 | 2 | 1 |
| OTU214 | 1 | 0 | 0 | 0 | 0 | 0 | 10 | 122 | 1 | 5 | 3 | 21 | 1 | 0 | 2 | 0 | 0 | 1 |
| OTU215 | 1 | 4 | 6 | 1 | 21 | 170 | 1 | 21 | 8 | 8 | 6 | 15 | 5 | 11 | 4 | 0 | 2 | 1 |
| OTU216 | 7 | 7 | 13 | 1 | 14 | 8 | 1 | 154 | 13 | 58 | 108 | 25 | 9 | 2 | 21 | 0 | 1 | 2 |
| OTU217 | 0 | 0 | 2 | 1 | 0 | 0 | 1 | 1 | 0 | 0 | 1 | 0 | 15 | 17 | 0 | 3 | 63 | 34 |
| OTU218 | 0 | 0 | 0 | 0 | 0 | 0 | 0 | 0 | 0 | 0 | 1 | 0 | 0 | 0 | 0 | 0 | 151 | 0 |
| OTU219 | 12 | 12 | 19 | 7 | 26 | 29 | 16 | 7 | 8 | 11 | 15 | 13 | 1 | 4 | 6 | 0 | 4 | 3 |
| OTU22 | 2304 | 4 | 126 | 0 | 36 | 25 | 4 | 1 | 1 | 12 | 66 | 12 | 8 | 2 | 1 | 0 | 1 | 1 |
| OTU220 | 65 | 0 | 111 | 0 | 0 | 22 | 12 | 10 | 2 | 0 | 6 | 10 | 2 | 5 | 0 | 0 | 1 | 0 |
| OTU221 | 48 | 14 | 27 | 51 | 11 | 99 | 0 | 0 | 0 | 1 | 0 | 0 | 0 | 0 | 0 | 0 | 0 | 0 |
| OTU222 | 3 | 6 | 16 | 12 | 82 | 290 | 3 | 23 | 22 | 33 | 22 | 18 | 1 | 3 | 11 | 0 | 3 | 3 |
| OTU223 | 0 | 2 | 0 | 0 | 1 | 2 | 0 | 0 | 1 | 22 | 104 | 0 | 3 | 2 | 0 | 0 | 3 | 2 |
| OTU224 | 1 | 9 | 2 | 2 | 10 | 15 | 0 | 16 | 57 | 10 | 32 | 21 | 2 | 3 | 27 | 0 | 3 | 2 |
| OTU225 | 0 | 0 | 0 | 0 | 1 | 1 | 13 | 11 | 36 | 13 | 15 | 28 | 3 | 4 | 24 | 0 | 2 | 0 |
| OTU226 | 0 | 0 | 0 | 0 | 0 | 0 | 28 | 11 | 19 | 2 | 9 | 20 | 0 | 0 | 20 | 0 | 0 | 1 |
| OTU227 | 0 | 0 | 0 | 0 | 0 | 0 | 19 | 14 | 18 | 5 | 7 | 20 | 0 | 9 | 13 | 0 | 3 | 0 |
| OTU228 | 0 | 0 | 82 | 0 | 0 | 1 | 80 | 6 | 16 | 0 | 0 | 18 | 0 | 4 | 16 | 0 | 0 | 0 |
| OTU229 | 17 | 27 | 16 | 6 | 31 | 59 | 48 | 26 | 12 | 31 | 8 | 26 | 1 | 5 | 10 | 0 | 2 | 2 |
| OTU23 | 21 | 359 | 1290 | 620 | 202 | 679 | 72 | 284 | 60 | 41 | 47 | 111 | 9 | 16 | 88 | 0 | 5 | 8 |
| OTU230 | 0 | 0 | 1 | 0 | 0 | 0 | 92 | 16 | 15 | 63 | 44 | 24 | 3 | 5 | 25 | 0 | 1 | 0 |
| OTU231 | 12 | 48 | 23 | 3 | 1 | 30 | 6 | 26 | 9 | 34 | 10 | 18 | 1 | 3 | 9 | 0 | 2 | 1 |
| OTU232 | 8 | 0 | 15 | 2 | 3 | 83 | 86 | 42 | 14 | 5 | 0 | 28 | 3 | 20 | 13 | 0 | 2 | 1 |
| OTU233 | 16 | 8 | 6 | 11 | 49 | 22 | 0 | 0 | 0 | 1 | 0 | 0 | 0 | 0 | 0 | 0 | 0 | 0 |
| OTU234 | 6 | 44 | 27 | 19 | 10 | 34 | 3 | 88 | 45 | 44 | 50 | 35 | 2 | 1 | 30 | 0 | 7 | 1 |
| OTU235 | 1 | 314 | 18 | 329 | 48 | 10 | 0 | 12 | 9 | 5 | 6 | 5 | 0 | 0 | 3 | 0 | 1 | 1 |
| OTU236 | 3 | 18 | 8 | 45 | 43 | 71 | 0 | 1 | 1 | 0 | 1 | 0 | 0 | 0 | 0 | 0 | 0 | 0 |
| OTU237 | 0 | 1 | 0 | 0 | 0 | 1 | 18 | 15 | 35 | 6 | 5 | 19 | 1 | 2 | 26 | 0 | 2 | 0 |
| OTU238 | 1 | 9 | 10 | 1 | 23 | 18 | 0 | 120 | 56 | 7 | 36 | 11 | 0 | 0 | 64 | 0 | 11 | 7 |
| OTU239 | 4 | 10 | 15 | 13 | 64 | 13 | 6 | 49 | 12 | 44 | 69 | 32 | 2 | 3 | 4 | 0 | 0 | 0 |
| OTU24 | 139 | 62 | 34 | 44 | 1395 | 1369 | 140 | 406 | 145 | 250 | 677 | 242 | 14 | 14 | 115 | 1 | 15 | 8 |
| OTU240 | 12 | 2 | 31 | 6 | 37 | 33 | 0 | 0 | 0 | 0 | 0 | 0 | 0 | 0 | 1 | 0 | 1 | 0 |
| OTU241 | 23 | 38 | 21 | 25 | 114 | 26 | 0 | 0 | 0 | 1 | 0 | 0 | 0 | 0 | 1 | 0 | 0 | 0 |
| OTU242 | 23 | 14 | 17 | 6 | 8 | 3 | 3 | 2 | 2 | 1 | 0 | 5 | 3 | 0 | 3 | 1 | 0 | 0 |
| OTU243 | 35 | 2 | 3 | 13 | 1 | 68 | 0 | 0 | 0 | 0 | 0 | 0 | 0 | 0 | 1 | 0 | 0 | 0 |
| OTU244 | 2 | 3 | 3 | 8 | 6 | 9 | 1 | 18 | 16 | 11 | 14 | 8 | 0 | 0 | 10 | 0 | 1 | 0 |
| OTU245 | 0 | 0 | 0 | 0 | 0 | 0 | 46 | 4 | 17 | 14 | 0 | 62 | 13 | 27 | 10 | 0 | 2 | 0 |
| OTU246 | 0 | 0 | 0 | 0 | 0 | 0 | 3 | 1 | 44 | 0 | 0 | 12 | 0 | 1 | 39 | 0 | 2 | 0 |
| OTU247 | 0 | 0 | 0 | 0 | 0 | 0 | 7 | 5 | 23 | 8 | 12 | 11 | 6 | 7 | 17 | 0 | 2 | 1 |
| OTU248 | 0 | 0 | 0 | 0 | 1 | 0 | 8 | 1 | 0 | 2 | 0 | 0 | 2 | 14 | 0 | 3 | 30 | 21 |
| OTU249 | 1 | 1 | 1 | 3 | 1 | 0 | 147 | 24 | 25 | 53 | 70 | 50 | 6 | 13 | 50 | 0 | 2 | 2 |
| OTU25 | 38 | 8 | 6 | 27 | 48 | 17 | 115 | 151 | 301 | 342 | 441 | 392 | 48 | 170 | 378 | 0 | 42 | 31 |
| OTU250 | 0 | 1 | 0 | 0 | 0 | 0 | 2 | 7 | 22 | 46 | 43 | 34 | 0 | 1 | 18 | 0 | 0 | 1 |
| OTU251 | 1 | 0 | 0 | 1 | 0 | 3 | 0 | 0 | 0 | 0 | 0 | 0 | 38 | 5 | 0 | 1 | 88 | 10 |
| OTU252 | 4 | 2 | 26 | 17 | 40 | 57 | 2 | 0 | 0 | 1 | 1 | 0 | 0 | 0 | 0 | 0 | 1 | 0 |
| OTU253 | 0 | 0 | 29 | 1 | 3 | 153 | 1 | 84 | 6 | 28 | 15 | 12 | 0 | 2 | 6 | 0 | 0 | 0 |
| OTU254 | 31 | 5 | 5 | 9 | 0 | 18 | 106 | 68 | 75 | 14 | 30 | 88 | 9 | 22 | 56 | 0 | 1 | 2 |
| OTU255 | 0 | 0 | 1 | 0 | 0 | 0 | 23 | 5 | 16 | 5 | 6 | 38 | 10 | 19 | 16 | 0 | 0 | 0 |
| OTU256 | 5 | 0 | 6 | 2 | 5 | 2 | 0 | 57 | 60 | 22 | 9 | 21 | 0 | 0 | 45 | 0 | 5 | 2 |
| OTU257 | 12 | 36 | 20 | 86 | 91 | 85 | 12 | 76 | 47 | 201 | 111 | 58 | 1 | 4 | 18 | 0 | 4 | 0 |
| OTU258 | 2 | 4 | 3 | 16 | 34 | 11 | 12 | 36 | 20 | 105 | 41 | 25 | 2 | 0 | 20 | 0 | 1 | 0 |
| OTU259 | 0 | 0 | 0 | 0 | 0 | 0 | 0 | 0 | 0 | 0 | 0 | 0 | 6 | 0 | 0 | 1 | 35 | 63 |
| OTU26 | 2 | 4 | 10 | 3 | 6 | 8 | 723 | 108 | 211 | 142 | 179 | 966 | 50 | 173 | 237 | 0 | 7 | 13 |
| OTU260 | 37 | 28 | 39 | 3 | 3 | 44 | 10 | 26 | 3 | 5 | 7 | 9 | 0 | 1 | 8 | 0 | 0 | 0 |
| OTU261 | 38 | 19 | 18 | 28 | 25 | 43 | 8 | 2 | 11 | 16 | 30 | 6 | 0 | 3 | 11 | 0 | 2 | 0 |
| OTU262 | 0 | 172 | 2 | 0 | 0 | 0 | 0 | 7 | 6 | 1 | 3 | 4 | 1 | 1 | 8 | 0 | 0 | 0 |
| OTU263 | 4 | 10 | 4 | 2 | 7 | 3 | 10 | 7 | 5 | 12 | 6 | 9 | 21 | 15 | 8 | 22 | 4 | 19 |
| OTU264 | 7 | 20 | 25 | 19 | 26 | 26 | 39 | 29 | 29 | 46 | 67 | 36 | 5 | 10 | 22 | 0 | 0 | 3 |
| OTU265 | 1 | 1 | 2 | 0 | 1 | 1 | 17 | 12 | 18 | 11 | 14 | 22 | 2 | 3 | 7 | 0 | 0 | 1 |
| OTU266 | 27 | 11 | 22 | 73 | 25 | 106 | 2 | 1 | 3 | 5 | 5 | 0 | 0 | 0 | 4 | 0 | 1 | 2 |
| OTU267 | 1 | 5 | 3 | 2 | 0 | 25 | 2 | 16 | 10 | 12 | 17 | 11 | 0 | 3 | 3 | 0 | 3 | 2 |
| OTU268 | 1 | 1 | 6 | 0 | 8 | 8 | 0 | 44 | 11 | 0 | 3 | 15 | 0 | 1 | 13 | 0 | 3 | 0 |
| OTU269 | 1 | 0 | 1 | 1 | 1 | 0 | 35 | 11 | 18 | 15 | 18 | 3 | 3 | 9 | 10 | 0 | 0 | 0 |
| OTU27 | 7 | 6 | 4 | 7 | 12 | 6 | 710 | 139 | 227 | 378 | 1995 | 850 | 41 | 79 | 39 | 0 | 4 | 5 |
| OTU270 | 0 | 0 | 2 | 0 | 0 | 0 | 58 | 4 | 6 | 13 | 20 | 20 | 2 | 5 | 12 | 0 | 0 | 0 |
| OTU271 | 8 | 19 | 5 | 1 | 8 | 13 | 13 | 21 | 18 | 10 | 11 | 18 | 2 | 7 | 21 | 0 | 1 | 2 |
| OTU272 | 0 | 0 | 0 | 0 | 0 | 0 | 47 | 7 | 22 | 22 | 10 | 50 | 2 | 7 | 22 | 0 | 0 | 0 |
| OTU273 | 0 | 0 | 0 | 0 | 0 | 0 | 0 | 0 | 0 | 0 | 0 | 0 | 5 | 0 | 0 | 0 | 101 | 5 |
| OTU274 | 0 | 0 | 0 | 0 | 0 | 0 | 1 | 0 | 0 | 0 | 0 | 0 | 1 | 0 | 0 | 0 | 178 | 0 |
| OTU275 | 1 | 0 | 0 | 0 | 0 | 0 | 0 | 0 | 0 | 0 | 0 | 0 | 1 | 1 | 0 | 1 | 346 | 0 |
| OTU276 | 75 | 1 | 4 | 3 | 1 | 22 | 0 | 1 | 0 | 0 | 1 | 41 | 0 | 1 | 0 | 0 | 0 | 0 |
| OTU277 | 22 | 12 | 53 | 15 | 2 | 14 | 4 | 2 | 6 | 2 | 0 | 3 | 0 | 3 | 4 | 0 | 0 | 1 |
| OTU278 | 5 | 16 | 35 | 0 | 5 | 33 | 0 | 0 | 0 | 0 | 0 | 0 | 0 | 0 | 0 | 0 | 0 | 0 |
| OTU279 | 14 | 4 | 19 | 8 | 12 | 2 | 9 | 6 | 0 | 26 | 16 | 17 | 0 | 0 | 0 | 0 | 1 | 0 |
| OTU28 | 40 | 131 | 204 | 10 | 883 | 654 | 2 | 0 | 1 | 1 | 1 | 1 | 3 | 1 | 1 | 3 | 0 | 2 |
| OTU280 | 0 | 2 | 2 | 1 | 5 | 2 | 4 | 4 | 5 | 3 | 5 | 6 | 0 | 3 | 3 | 5 | 2 | 2 |
| OTU281 | 3 | 81 | 0 | 0 | 0 | 1 | 11 | 31 | 4 | 0 | 2 | 3 | 0 | 0 | 4 | 0 | 0 | 0 |
| OTU282 | 2 | 12 | 4 | 1 | 105 | 60 | 2 | 16 | 21 | 37 | 30 | 12 | 0 | 1 | 12 | 0 | 2 | 2 |
| OTU283 | 1 | 0 | 5 | 14 | 32 | 28 | 1 | 6 | 8 | 8 | 6 | 5 | 0 | 0 | 12 | 0 | 1 | 0 |
| OTU284 | 0 | 0 | 0 | 0 | 1 | 0 | 6 | 9 | 11 | 2 | 3 | 30 | 2 | 2 | 18 | 0 | 0 | 0 |
| OTU285 | 0 | 0 | 1 | 1 | 0 | 0 | 11 | 25 | 16 | 17 | 5 | 45 | 0 | 9 | 20 | 0 | 3 | 0 |
| OTU286 | 0 | 0 | 0 | 0 | 0 | 0 | 2 | 13 | 12 | 15 | 5 | 7 | 4 | 15 | 8 | 0 | 1 | 0 |
| OTU287 | 0 | 3 | 0 | 3 | 14 | 0 | 2 | 39 | 11 | 2 | 21 | 67 | 1 | 11 | 10 | 0 | 0 | 0 |
| OTU288 | 0 | 0 | 0 | 0 | 0 | 0 | 0 | 0 | 0 | 0 | 0 | 0 | 8 | 26 | 0 | 1 | 65 | 30 |
| OTU289 | 0 | 1 | 0 | 0 | 0 | 0 | 0 | 0 | 0 | 0 | 0 | 0 | 11 | 5 | 0 | 0 | 64 | 17 |
| OTU29 | 171 | 22 | 81 | 38 | 37 | 78 | 720 | 1100 | 1508 | 625 | 1043 | 1290 | 155 | 543 | 1300 | 9 | 67 | 72 |
| OTU290 | 35 | 43 | 12 | 0 | 2 | 19 | 5 | 5 | 1 | 5 | 4 | 16 | 0 | 4 | 0 | 0 | 2 | 0 |
| OTU291 | 10 | 13 | 16 | 2 | 30 | 1 | 1 | 0 | 1 | 0 | 0 | 0 | 0 | 0 | 0 | 0 | 0 | 0 |
| OTU292 | 41 | 0 | 15 | 13 | 11 | 26 | 0 | 0 | 0 | 0 | 0 | 0 | 0 | 0 | 0 | 0 | 0 | 1 |
| OTU293 | 53 | 33 | 70 | 15 | 27 | 20 | 0 | 1 | 0 | 0 | 2 | 0 | 0 | 0 | 0 | 0 | 0 | 0 |
| OTU294 | 19 | 0 | 11 | 21 | 5 | 18 | 2 | 0 | 0 | 0 | 0 | 0 | 0 | 0 | 0 | 0 | 0 | 0 |
| OTU295 | 0 | 28 | 2 | 0 | 0 | 22 | 11 | 3 | 10 | 0 | 0 | 22 | 1 | 2 | 6 | 0 | 2 | 1 |
| OTU296 | 0 | 21 | 5 | 7 | 1 | 95 | 1 | 36 | 11 | 14 | 6 | 8 | 0 | 1 | 7 | 0 | 1 | 2 |
| OTU297 | 0 | 0 | 0 | 2 | 0 | 0 | 14 | 2 | 5 | 42 | 7 | 11 | 3 | 3 | 6 | 0 | 0 | 2 |
| OTU298 | 8 | 9 | 15 | 10 | 19 | 9 | 10 | 21 | 12 | 13 | 11 | 18 | 30 | 24 | 15 | 38 | 16 | 26 |
| OTU299 | 0 | 0 | 0 | 1 | 56 | 16 | 3 | 12 | 5 | 13 | 5 | 0 | 1 | 1 | 4 | 0 | 1 | 0 |
| OTU3 | 2388 | 130 | 660 | 1393 | 693 | 779 | 1117 | 2742 | 2408 | 1755 | 731 | 979 | 11350 | 7912 | 3215 | 9927 | 4024 | 11377 |
| OTU30 | 14 | 7 | 22 | 14 | 13 | 18 | 959 | 279 | 387 | 349 | 682 | 344 | 27 | 93 | 564 | 0 | 2 | 5 |
| OTU300 | 1 | 3 | 1 | 0 | 0 | 1 | 62 | 12 | 19 | 51 | 104 | 12 | 2 | 5 | 40 | 0 | 0 | 3 |
| OTU301 | 1 | 0 | 0 | 0 | 1 | 7 | 11 | 26 | 3 | 3 | 35 | 5 | 0 | 3 | 2 | 0 | 0 | 0 |
| OTU302 | 0 | 0 | 4 | 0 | 8 | 0 | 2 | 8 | 5 | 11 | 32 | 2 | 0 | 1 | 2 | 0 | 1 | 1 |
| OTU303 | 0 | 18 | 2 | 0 | 2 | 85 | 6 | 21 | 67 | 9 | 59 | 66 | 0 | 1 | 71 | 0 | 2 | 3 |
| OTU304 | 0 | 2 | 5 | 0 | 1 | 2 | 10 | 105 | 61 | 18 | 7 | 30 | 2 | 4 | 42 | 0 | 3 | 5 |
| OTU305 | 0 | 0 | 0 | 0 | 0 | 0 | 2 | 39 | 35 | 0 | 2 | 75 | 6 | 10 | 32 | 0 | 1 | 0 |
| OTU306 | 0 | 0 | 0 | 0 | 0 | 0 | 0 | 0 | 0 | 0 | 0 | 0 | 6 | 10 | 0 | 4 | 27 | 151 |
| OTU307 | 0 | 0 | 0 | 0 | 0 | 0 | 0 | 0 | 0 | 0 | 0 | 0 | 8 | 40 | 0 | 1 | 56 | 35 |
| OTU308 | 0 | 1 | 0 | 0 | 0 | 0 | 0 | 0 | 0 | 0 | 0 | 0 | 0 | 0 | 0 | 0 | 94 | 0 |
| OTU309 | 1 | 8 | 11 | 0 | 0 | 28 | 1 | 30 | 4 | 15 | 25 | 6 | 3 | 4 | 4 | 0 | 1 | 0 |
| OTU31 | 72 | 178 | 95 | 664 | 499 | 1012 | 79 | 22 | 15 | 74 | 32 | 11 | 4 | 11 | 10 | 0 | 7 | 5 |
| OTU310 | 9 | 1 | 4 | 0 | 0 | 0 | 24 | 27 | 21 | 4 | 6 | 18 | 1 | 10 | 23 | 0 | 2 | 1 |
| OTU311 | 8 | 0 | 1 | 0 | 0 | 0 | 3 | 1 | 0 | 45 | 0 | 0 | 0 | 0 | 0 | 1 | 0 | 0 |
| OTU312 | 8 | 4 | 7 | 2 | 6 | 3 | 2 | 8 | 11 | 5 | 3 | 5 | 1 | 3 | 11 | 0 | 1 | 0 |
| OTU313 | 15 | 4 | 6 | 1 | 8 | 5 | 0 | 1 | 0 | 0 | 0 | 0 | 0 | 0 | 0 | 0 | 0 | 0 |
| OTU314 | 2 | 18 | 28 | 13 | 21 | 101 | 7 | 101 | 60 | 56 | 62 | 36 | 4 | 24 | 25 | 0 | 8 | 9 |
| OTU315 | 0 | 1 | 1 | 0 | 0 | 0 | 27 | 10 | 12 | 12 | 24 | 33 | 2 | 4 | 20 | 0 | 0 | 0 |
| OTU316 | 8 | 10 | 7 | 1 | 0 | 2 | 3 | 13 | 9 | 11 | 7 | 8 | 1 | 8 | 14 | 0 | 2 | 0 |
| OTU317 | 114 | 26 | 34 | 0 | 128 | 14 | 38 | 11 | 14 | 153 | 24 | 51 | 7 | 24 | 18 | 0 | 7 | 8 |
| OTU318 | 15 | 1 | 10 | 36 | 10 | 41 | 0 | 0 | 0 | 0 | 0 | 0 | 0 | 0 | 0 | 0 | 0 | 0 |
| OTU319 | 1 | 0 | 0 | 0 | 0 | 1 | 0 | 71 | 42 | 1 | 2 | 29 | 0 | 1 | 25 | 0 | 3 | 7 |
| OTU32 | 244 | 156 | 271 | 339 | 240 | 641 | 3 | 3 | 0 | 1 | 1 | 1 | 1 | 0 | 1 | 1 | 0 | 1 |
| OTU320 | 7 | 6 | 12 | 5 | 5 | 14 | 6 | 11 | 12 | 11 | 11 | 10 | 2 | 8 | 4 | 2 | 23 | 7 |
| OTU321 | 4 | 12 | 18 | 3 | 10 | 11 | 5 | 11 | 10 | 11 | 12 | 13 | 6 | 15 | 12 | 4 | 20 | 4 |
| OTU322 | 9 | 148 | 23 | 1 | 19 | 0 | 0 | 0 | 0 | 0 | 0 | 0 | 0 | 0 | 0 | 0 | 0 | 0 |
| OTU323 | 7 | 12 | 18 | 2 | 1 | 2 | 7 | 92 | 19 | 37 | 20 | 24 | 0 | 3 | 29 | 0 | 2 | 1 |
| OTU324 | 0 | 91 | 4 | 0 | 1 | 4 | 0 | 0 | 0 | 1 | 0 | 0 | 1 | 0 | 0 | 0 | 0 | 0 |
| OTU325 | 1 | 34 | 8 | 11 | 40 | 3 | 0 | 0 | 0 | 0 | 1 | 0 | 0 | 0 | 1 | 0 | 0 | 0 |
| OTU326 | 38 | 15 | 11 | 0 | 0 | 2 | 130 | 35 | 10 | 1 | 0 | 5 | 0 | 3 | 7 | 1 | 1 | 1 |
| OTU327 | 0 | 0 | 1 | 0 | 1 | 5 | 0 | 0 | 0 | 3 | 0 | 0 | 8 | 3 | 0 | 1 | 50 | 4 |
| OTU328 | 0 | 0 | 0 | 0 | 0 | 0 | 7 | 0 | 3 | 0 | 9 | 9 | 0 | 3 | 0 | 0 | 0 | 0 |
| OTU329 | 6 | 6 | 4 | 5 | 3 | 2 | 6 | 5 | 9 | 10 | 3 | 9 | 19 | 13 | 6 | 18 | 12 | 8 |
| OTU33 | 4 | 4 | 276 | 1 | 2 | 175 | 0 | 68 | 7 | 441 | 1063 | 12 | 3 | 7 | 7 | 0 | 3 | 0 |
| OTU330 | 1 | 1 | 0 | 0 | 0 | 0 | 0 | 2 | 0 | 38 | 17 | 0 | 0 | 0 | 0 | 0 | 2 | 0 |
| OTU331 | 0 | 1 | 0 | 0 | 0 | 0 | 0 | 0 | 0 | 0 | 0 | 0 | 0 | 0 | 1 | 0 | 58 | 0 |
| OTU332 | 0 | 0 | 0 | 0 | 0 | 0 | 0 | 0 | 0 | 0 | 0 | 0 | 3 | 13 | 0 | 0 | 108 | 28 |
| OTU333 | 6 | 9 | 8 | 10 | 28 | 1 | 1 | 0 | 0 | 1 | 0 | 0 | 0 | 1 | 0 | 0 | 0 | 0 |
| OTU334 | 1 | 0 | 2 | 5 | 2 | 1 | 8 | 1 | 7 | 17 | 2 | 11 | 2 | 2 | 4 | 0 | 0 | 2 |
| OTU335 | 4 | 3 | 11 | 0 | 3 | 9 | 0 | 4 | 3 | 3 | 7 | 5 | 0 | 0 | 0 | 0 | 0 | 0 |
| OTU336 | 33 | 11 | 26 | 6 | 2 | 24 | 6 | 0 | 1 | 1 | 4 | 0 | 0 | 0 | 2 | 0 | 0 | 1 |
| OTU337 | 23 | 28 | 21 | 11 | 22 | 30 | 10 | 3 | 2 | 7 | 6 | 3 | 0 | 0 | 6 | 0 | 0 | 1 |
| OTU338 | 0 | 6 | 0 | 4 | 29 | 2 | 0 | 0 | 0 | 0 | 1 | 0 | 0 | 0 | 0 | 0 | 0 | 0 |
| OTU339 | 3 | 3 | 5 | 2 | 5 | 5 | 2 | 0 | 4 | 2 | 3 | 2 | 1 | 1 | 1 | 1 | 0 | 0 |
| OTU34 | 226 | 34 | 258 | 109 | 885 | 30 | 6 | 0 | 3 | 4 | 5 | 0 | 0 | 1 | 0 | 0 | 0 | 3 |
| OTU340 | 2 | 1 | 12 | 2 | 0 | 4 | 0 | 2 | 14 | 4 | 0 | 10 | 1 | 6 | 10 | 0 | 0 | 0 |
| OTU341 | 0 | 12 | 0 | 18 | 0 | 35 | 0 | 0 | 1 | 0 | 1 | 0 | 0 | 0 | 0 | 0 | 0 | 0 |
| OTU342 | 1 | 2 | 3 | 3 | 3 | 59 | 1 | 9 | 5 | 26 | 4 | 9 | 0 | 0 | 2 | 0 | 1 | 0 |
| OTU343 | 5 | 8 | 10 | 0 | 9 | 3 | 0 | 7 | 0 | 3 | 0 | 2 | 2 | 2 | 1 | 0 | 0 | 0 |
| OTU344 | 6 | 5 | 8 | 3 | 7 | 17 | 5 | 5 | 4 | 4 | 8 | 10 | 1 | 5 | 14 | 0 | 2 | 0 |
| OTU345 | 15 | 4 | 5 | 25 | 7 | 15 | 0 | 0 | 0 | 0 | 0 | 0 | 0 | 0 | 0 | 0 | 0 | 0 |
| OTU346 | 0 | 0 | 0 | 1 | 1 | 0 | 0 | 0 | 0 | 0 | 1 | 0 | 4 | 4 | 0 | 1 | 33 | 10 |
| OTU347 | 4 | 4 | 7 | 1 | 8 | 23 | 33 | 23 | 8 | 32 | 33 | 15 | 2 | 8 | 7 | 0 | 3 | 1 |
| OTU348 | 0 | 2 | 5 | 0 | 1 | 80 | 0 | 11 | 3 | 19 | 3 | 6 | 1 | 0 | 3 | 0 | 2 | 1 |
| OTU349 | 0 | 0 | 2 | 0 | 0 | 2 | 8 | 16 | 2 | 25 | 2 | 10 | 2 | 9 | 14 | 0 | 1 | 0 |
| OTU35 | 286 | 339 | 403 | 427 | 496 | 226 | 6 | 2 | 4 | 8 | 5 | 7 | 2 | 0 | 1 | 0 | 2 | 3 |
| OTU350 | 0 | 0 | 5 | 1 | 3 | 0 | 13 | 11 | 3 | 4 | 3 | 13 | 2 | 4 | 4 | 0 | 1 | 0 |
| OTU351 | 0 | 1 | 0 | 1 | 0 | 0 | 14 | 0 | 5 | 17 | 8 | 9 | 0 | 4 | 5 | 0 | 0 | 0 |
| OTU352 | 0 | 0 | 0 | 0 | 0 | 0 | 15 | 0 | 12 | 8 | 4 | 21 | 1 | 2 | 16 | 0 | 0 | 0 |
| OTU353 | 2 | 15 | 1 | 4 | 0 | 0 | 6 | 8 | 1 | 18 | 13 | 2 | 1 | 1 | 2 | 0 | 1 | 0 |
| OTU354 | 0 | 0 | 0 | 0 | 0 | 0 | 9 | 6 | 2 | 24 | 2 | 5 | 0 | 0 | 6 | 0 | 2 | 0 |
| OTU355 | 4 | 0 | 16 | 0 | 0 | 0 | 2 | 13 | 1 | 23 | 39 | 0 | 0 | 0 | 5 | 0 | 0 | 0 |
| OTU356 | 0 | 0 | 0 | 0 | 0 | 0 | 0 | 0 | 0 | 4 | 1 | 3 | 5 | 7 | 0 | 6 | 60 | 9 |
| OTU357 | 0 | 0 | 0 | 0 | 0 | 0 | 0 | 3 | 13 | 1 | 8 | 21 | 0 | 3 | 8 | 0 | 2 | 0 |
| OTU358 | 0 | 0 | 1 | 0 | 0 | 0 | 0 | 0 | 0 | 0 | 0 | 0 | 2 | 19 | 0 | 2 | 46 | 66 |
| OTU359 | 0 | 0 | 0 | 0 | 0 | 0 | 0 | 1 | 0 | 0 | 0 | 0 | 11 | 1 | 0 | 8 | 134 | 204 |
| OTU36 | 0 | 3 | 8 | 1 | 32 | 58 | 46 | 506 | 16 | 308 | 358 | 369 | 30 | 6 | 270 | 1 | 26 | 19 |
| OTU360 | 3 | 0 | 2 | 14 | 0 | 41 | 0 | 5 | 6 | 3 | 1 | 3 | 0 | 0 | 8 | 0 | 1 | 0 |
| OTU361 | 5 | 2 | 2 | 6 | 13 | 12 | 2 | 7 | 9 | 8 | 10 | 6 | 0 | 1 | 4 | 0 | 0 | 0 |
| OTU362 | 18 | 6 | 7 | 9 | 13 | 14 | 1 | 5 | 8 | 5 | 1 | 3 | 1 | 2 | 12 | 0 | 0 | 0 |
| OTU363 | 2 | 6 | 8 | 2 | 11 | 57 | 0 | 2 | 2 | 15 | 4 | 1 | 0 | 0 | 2 | 0 | 0 | 1 |
| OTU364 | 60 | 22 | 12 | 5 | 17 | 20 | 1 | 0 | 1 | 10 | 8 | 4 | 2 | 1 | 0 | 0 | 0 | 0 |
| OTU365 | 5 | 19 | 5 | 3 | 21 | 2 | 0 | 0 | 0 | 0 | 0 | 0 | 0 | 0 | 0 | 0 | 0 | 0 |
| OTU366 | 3 | 1 | 0 | 7 | 5 | 1 | 0 | 2 | 2 | 3 | 1 | 7 | 1 | 8 | 2 | 0 | 2 | 2 |
| OTU367 | 7 | 0 | 1 | 1 | 3 | 5 | 1 | 8 | 2 | 0 | 2 | 2 | 0 | 0 | 0 | 0 | 0 | 0 |
| OTU368 | 52 | 9 | 1 | 3 | 6 | 2 | 37 | 0 | 6 | 0 | 2 | 25 | 4 | 8 | 7 | 0 | 0 | 0 |
| OTU369 | 58 | 1 | 8 | 0 | 0 | 1 | 0 | 0 | 0 | 0 | 0 | 0 | 1 | 0 | 0 | 0 | 0 | 0 |
| OTU37 | 8 | 6 | 9 | 12 | 7 | 8 | 991 | 313 | 637 | 286 | 608 | 76 | 12 | 15 | 754 | 0 | 11 | 12 |
| OTU370 | 16 | 0 | 2 | 0 | 15 | 0 | 0 | 0 | 0 | 0 | 0 | 0 | 0 | 0 | 0 | 0 | 0 | 0 |
| OTU371 | 2 | 31 | 0 | 1 | 13 | 12 | 0 | 0 | 1 | 1 | 0 | 2 | 0 | 0 | 0 | 0 | 0 | 0 |
| OTU372 | 0 | 8 | 3 | 0 | 0 | 1 | 5 | 10 | 8 | 4 | 0 | 10 | 0 | 1 | 9 | 1 | 2 | 0 |
| OTU373 | 3 | 16 | 3 | 18 | 15 | 1 | 1 | 1 | 0 | 5 | 20 | 4 | 0 | 0 | 1 | 0 | 1 | 0 |
| OTU374 | 4 | 7 | 10 | 2 | 3 | 13 | 5 | 4 | 7 | 7 | 16 | 12 | 2 | 4 | 7 | 5 | 6 | 1 |
| OTU375 | 0 | 87 | 0 | 0 | 1 | 1 | 0 | 0 | 0 | 0 | 0 | 0 | 0 | 0 | 0 | 0 | 0 | 0 |
| OTU376 | 5 | 16 | 48 | 0 | 1 | 3 | 0 | 0 | 0 | 0 | 0 | 0 | 0 | 0 | 0 | 0 | 0 | 0 |
| OTU377 | 0 | 2 | 0 | 0 | 31 | 1 | 2 | 16 | 8 | 9 | 11 | 2 | 1 | 0 | 7 | 0 | 2 | 1 |
| OTU378 | 0 | 1 | 4 | 2 | 68 | 22 | 0 | 0 | 0 | 0 | 0 | 6 | 0 | 0 | 2 | 0 | 0 | 0 |
| OTU379 | 4 | 1 | 8 | 1 | 15 | 4 | 9 | 2 | 29 | 13 | 18 | 38 | 2 | 7 | 55 | 0 | 4 | 0 |
| OTU38 | 3 | 95 | 6 | 3 | 4 | 160 | 1 | 273 | 324 | 1008 | 45 | 70 | 4 | 11 | 198 | 0 | 13 | 13 |
| OTU380 | 0 | 1 | 3 | 4 | 6 | 4 | 1 | 93 | 27 | 30 | 6 | 23 | 0 | 1 | 9 | 0 | 3 | 1 |
| OTU381 | 0 | 2 | 1 | 67 | 0 | 1 | 0 | 0 | 0 | 0 | 0 | 1 | 0 | 0 | 0 | 0 | 1 | 0 |
| OTU382 | 2 | 2 | 2 | 0 | 2 | 6 | 4 | 11 | 19 | 6 | 9 | 16 | 11 | 13 | 9 | 0 | 2 | 3 |
| OTU383 | 1 | 4 | 0 | 0 | 2 | 0 | 5 | 1 | 1 | 1 | 10 | 2 | 1 | 2 | 0 | 0 | 0 | 1 |
| OTU384 | 32 | 1 | 2 | 0 | 1 | 0 | 19 | 2 | 4 | 1 | 1 | 9 | 0 | 12 | 5 | 0 | 0 | 0 |
| OTU385 | 0 | 0 | 0 | 0 | 0 | 0 | 2 | 10 | 2 | 31 | 13 | 7 | 0 | 0 | 5 | 0 | 0 | 0 |
| OTU386 | 0 | 0 | 1 | 0 | 0 | 0 | 2 | 30 | 11 | 2 | 4 | 11 | 0 | 3 | 0 | 0 | 0 | 0 |
| OTU387 | 1 | 1 | 0 | 7 | 9 | 6 | 2 | 6 | 13 | 18 | 8 | 32 | 0 | 1 | 1 | 0 | 0 | 0 |
| OTU388 | 0 | 0 | 0 | 0 | 0 | 0 | 3 | 1 | 2 | 2 | 1 | 0 | 4 | 21 | 1 | 0 | 22 | 62 |
| OTU389 | 0 | 8 | 3 | 4 | 3 | 7 | 23 | 13 | 14 | 22 | 18 | 55 | 7 | 22 | 17 | 0 | 3 | 1 |
| OTU39 | 115 | 924 | 270 | 0 | 0 | 9 | 19 | 80 | 2 | 1 | 3 | 4 | 2 | 0 | 1 | 1 | 0 | 0 |
| OTU390 | 0 | 0 | 0 | 0 | 0 | 0 | 0 | 0 | 0 | 0 | 0 | 0 | 2 | 5 | 0 | 0 | 35 | 6 |
| OTU391 | 0 | 1 | 0 | 0 | 0 | 0 | 0 | 0 | 0 | 0 | 1 | 0 | 2 | 1 | 0 | 1 | 42 | 16 |
| OTU392 | 9 | 7 | 6 | 2 | 0 | 1 | 2 | 2 | 2 | 0 | 0 | 1 | 0 | 3 | 2 | 0 | 0 | 1 |
| OTU393 | 5 | 14 | 2 | 3 | 7 | 1 | 0 | 0 | 0 | 1 | 0 | 0 | 0 | 0 | 0 | 0 | 0 | 0 |
| OTU394 | 11 | 11 | 17 | 1 | 48 | 2 | 0 | 47 | 54 | 11 | 15 | 28 | 2 | 3 | 29 | 0 | 3 | 1 |
| OTU395 | 13 | 10 | 10 | 12 | 9 | 0 | 1 | 0 | 0 | 0 | 0 | 0 | 0 | 0 | 0 | 0 | 0 | 0 |
| OTU396 | 10 | 4 | 1 | 5 | 6 | 11 | 3 | 4 | 5 | 2 | 7 | 3 | 0 | 0 | 12 | 0 | 0 | 1 |
| OTU397 | 20 | 9 | 25 | 18 | 3 | 22 | 1 | 0 | 0 | 1 | 0 | 0 | 0 | 0 | 0 | 0 | 0 | 0 |
| OTU398 | 3 | 3 | 2 | 1 | 0 | 2 | 1 | 1 | 2 | 5 | 4 | 3 | 1 | 1 | 2 | 1 | 3 | 0 |
| OTU399 | 6 | 1 | 4 | 1 | 12 | 2 | 0 | 13 | 10 | 11 | 4 | 1 | 0 | 0 | 5 | 0 | 0 | 0 |
| OTU4 | 28 | 16 | 39 | 15 | 34 | 24 | 3 | 31 | 14 | 20 | 16 | 21 | 2503 | 1992 | 20 | 273 | 12142 | 7471 |
| OTU40 | 0 | 2 | 2 | 0 | 4 | 2 | 2 | 750 | 338 | 582 | 1203 | 765 | 6 | 6 | 66 | 1 | 8 | 2 |
| OTU400 | 5 | 2 | 2 | 8 | 14 | 8 | 1 | 0 | 1 | 11 | 2 | 0 | 0 | 0 | 5 | 0 | 0 | 0 |
| OTU401 | 10 | 8 | 9 | 1 | 9 | 12 | 0 | 0 | 0 | 0 | 0 | 0 | 1 | 0 | 0 | 0 | 0 | 0 |
| OTU402 | 9 | 9 | 1 | 0 | 21 | 28 | 0 | 1 | 0 | 2 | 5 | 0 | 1 | 0 | 1 | 0 | 0 | 0 |
| OTU403 | 10 | 31 | 12 | 3 | 7 | 12 | 2 | 1 | 1 | 0 | 0 | 0 | 1 | 0 | 0 | 0 | 0 | 0 |
| OTU404 | 4 | 14 | 2 | 18 | 70 | 47 | 0 | 22 | 15 | 8 | 9 | 13 | 1 | 5 | 6 | 0 | 1 | 1 |
| OTU405 | 14 | 16 | 10 | 19 | 8 | 3 | 6 | 0 | 0 | 0 | 0 | 1 | 0 | 0 | 0 | 0 | 0 | 0 |
| OTU406 | 0 | 1 | 1 | 8 | 7 | 73 | 0 | 1 | 0 | 1 | 0 | 0 | 0 | 0 | 1 | 0 | 2 | 0 |
| OTU407 | 0 | 1 | 4 | 0 | 14 | 17 | 0 | 0 | 0 | 0 | 0 | 0 | 0 | 0 | 0 | 0 | 0 | 0 |
| OTU408 | 1 | 2 | 3 | 13 | 7 | 5 | 1 | 5 | 17 | 17 | 7 | 2 | 0 | 0 | 20 | 0 | 0 | 0 |
| OTU409 | 4 | 1 | 8 | 14 | 0 | 4 | 2 | 1 | 1 | 0 | 3 | 2 | 0 | 0 | 2 | 0 | 0 | 0 |
| OTU41 | 14 | 17 | 27 | 20 | 55 | 85 | 6 | 120 | 64 | 1089 | 1067 | 165 | 9 | 22 | 46 | 1 | 13 | 3 |
| OTU410 | 13 | 12 | 11 | 1 | 50 | 69 | 5 | 5 | 7 | 0 | 1 | 6 | 0 | 2 | 4 | 0 | 0 | 0 |
| OTU411 | 1 | 4 | 4 | 2 | 3 | 12 | 2 | 4 | 6 | 4 | 7 | 2 | 0 | 1 | 1 | 0 | 0 | 0 |
| OTU412 | 9 | 2 | 9 | 23 | 17 | 28 | 0 | 0 | 0 | 0 | 0 | 1 | 0 | 0 | 0 | 0 | 1 | 0 |
| OTU413 | 0 | 9 | 1 | 3 | 19 | 10 | 0 | 7 | 6 | 6 | 1 | 7 | 0 | 6 | 0 | 0 | 0 | 0 |
| OTU414 | 0 | 0 | 14 | 0 | 42 | 0 | 54 | 2 | 34 | 0 | 0 | 57 | 0 | 11 | 31 | 0 | 4 | 0 |
| OTU415 | 0 | 0 | 0 | 0 | 0 | 0 | 5 | 4 | 2 | 10 | 5 | 3 | 0 | 0 | 0 | 0 | 0 | 0 |
| OTU416 | 0 | 0 | 0 | 1 | 0 | 1 | 30 | 2 | 2 | 12 | 2 | 6 | 2 | 4 | 0 | 0 | 1 | 0 |
| OTU417 | 1 | 4 | 12 | 11 | 33 | 9 | 9 | 0 | 13 | 3 | 2 | 0 | 0 | 0 | 9 | 0 | 0 | 2 |
| OTU418 | 0 | 1 | 0 | 0 | 0 | 0 | 10 | 3 | 1 | 4 | 5 | 11 | 4 | 4 | 1 | 0 | 0 | 0 |
| OTU419 | 2 | 5 | 9 | 1 | 8 | 4 | 34 | 21 | 22 | 31 | 51 | 17 | 0 | 7 | 18 | 0 | 3 | 0 |
| OTU42 | 194 | 58 | 86 | 2 | 66 | 125 | 307 | 114 | 228 | 247 | 74 | 48 | 236 | 13 | 142 | 14 | 28 | 38 |
| OTU420 | 3 | 4 | 2 | 2 | 6 | 2 | 8 | 6 | 8 | 5 | 4 | 3 | 7 | 2 | 2 | 11 | 4 | 4 |
| OTU421 | 0 | 0 | 0 | 0 | 0 | 0 | 2 | 8 | 15 | 19 | 32 | 5 | 2 | 4 | 8 | 0 | 1 | 1 |
| OTU422 | 0 | 3 | 0 | 0 | 1 | 16 | 20 | 13 | 0 | 5 | 13 | 0 | 0 | 0 | 1 | 0 | 0 | 0 |
| OTU423 | 0 | 0 | 0 | 0 | 0 | 0 | 24 | 7 | 2 | 13 | 11 | 11 | 0 | 2 | 10 | 0 | 0 | 1 |
| OTU424 | 26 | 22 | 10 | 11 | 20 | 37 | 170 | 100 | 126 | 0 | 7 | 157 | 17 | 58 | 105 | 0 | 7 | 1 |
| OTU425 | 0 | 7 | 4 | 3 | 9 | 5 | 7 | 49 | 24 | 49 | 57 | 26 | 1 | 4 | 10 | 0 | 2 | 2 |
| OTU426 | 2 | 1 | 4 | 1 | 2 | 0 | 1 | 6 | 1 | 1 | 1 | 5 | 0 | 10 | 4 | 0 | 0 | 2 |
| OTU427 | 0 | 0 | 0 | 0 | 0 | 0 | 4 | 0 | 3 | 3 | 0 | 14 | 1 | 7 | 6 | 0 | 0 | 1 |
| OTU428 | 0 | 0 | 0 | 0 | 0 | 0 | 0 | 1 | 0 | 1 | 0 | 0 | 10 | 10 | 0 | 0 | 46 | 16 |
| OTU429 | 0 | 0 | 0 | 0 | 0 | 0 | 0 | 0 | 0 | 0 | 0 | 0 | 4 | 7 | 0 | 0 | 37 | 29 |
| OTU43 | 286 | 86 | 465 | 156 | 317 | 225 | 2 | 0 | 0 | 2 | 2 | 1 | 1 | 1 | 3 | 0 | 1 | 0 |
| OTU430 | 0 | 0 | 0 | 0 | 0 | 0 | 0 | 0 | 0 | 0 | 1 | 0 | 0 | 0 | 0 | 0 | 17 | 0 |
| OTU431 | 0 | 0 | 0 | 0 | 1 | 1 | 0 | 0 | 0 | 0 | 0 | 0 | 2 | 4 | 0 | 1 | 63 | 19 |
| OTU432 | 71 | 7 | 4 | 76 | 1 | 3 | 49 | 25 | 24 | 7 | 5 | 15 | 1 | 6 | 20 | 0 | 4 | 0 |
| OTU433 | 7 | 6 | 10 | 5 | 8 | 5 | 7 | 10 | 11 | 13 | 9 | 10 | 4 | 2 | 9 | 5 | 14 | 6 |
| OTU434 | 6 | 13 | 3 | 3 | 9 | 11 | 0 | 0 | 0 | 0 | 0 | 0 | 0 | 0 | 1 | 0 | 0 | 0 |
| OTU435 | 6 | 1 | 2 | 3 | 4 | 4 | 1 | 4 | 0 | 0 | 0 | 1 | 0 | 1 | 1 | 0 | 0 | 0 |
| OTU436 | 6 | 35 | 10 | 0 | 0 | 7 | 0 | 1 | 0 | 0 | 0 | 0 | 0 | 0 | 0 | 0 | 0 | 0 |
| OTU437 | 28 | 1 | 1 | 36 | 7 | 63 | 4 | 28 | 6 | 8 | 6 | 6 | 1 | 0 | 5 | 0 | 0 | 0 |
| OTU438 | 2 | 8 | 6 | 2 | 8 | 6 | 0 | 0 | 0 | 1 | 1 | 0 | 0 | 0 | 0 | 0 | 0 | 0 |
| OTU439 | 7 | 3 | 4 | 0 | 1 | 8 | 7 | 7 | 0 | 0 | 0 | 6 | 0 | 0 | 2 | 0 | 0 | 0 |
| OTU44 | 0 | 1 | 1 | 0 | 1 | 0 | 1 | 2 | 3 | 2 | 4 | 2 | 3 | 4 | 1 | 1 | 1037 | 4 |
| OTU440 | 0 | 105 | 1 | 0 | 0 | 0 | 0 | 0 | 0 | 1 | 0 | 1 | 0 | 0 | 0 | 0 | 0 | 0 |
| OTU441 | 0 | 12 | 1 | 4 | 0 | 2 | 1 | 34 | 9 | 16 | 13 | 6 | 0 | 0 | 9 | 0 | 1 | 2 |
| OTU442 | 1 | 1 | 13 | 7 | 14 | 6 | 0 | 0 | 0 | 1 | 0 | 0 | 0 | 0 | 0 | 0 | 0 | 0 |
| OTU443 | 10 | 28 | 21 | 3 | 4 | 6 | 1 | 0 | 0 | 0 | 0 | 0 | 0 | 0 | 0 | 0 | 1 | 0 |
| OTU444 | 0 | 0 | 12 | 0 | 0 | 0 | 2 | 5 | 7 | 0 | 0 | 2 | 0 | 0 | 4 | 0 | 0 | 0 |
| OTU445 | 4 | 0 | 1 | 3 | 0 | 20 | 0 | 0 | 0 | 1 | 2 | 3 | 0 | 0 | 0 | 0 | 0 | 0 |
| OTU446 | 0 | 2 | 16 | 1 | 0 | 1 | 7 | 0 | 2 | 0 | 0 | 1 | 0 | 0 | 1 | 0 | 0 | 0 |
| OTU447 | 1 | 0 | 34 | 0 | 0 | 2 | 0 | 0 | 0 | 0 | 0 | 0 | 0 | 0 | 0 | 0 | 1 | 0 |
| OTU448 | 1 | 0 | 6 | 0 | 0 | 3 | 1 | 0 | 1 | 0 | 0 | 4 | 0 | 0 | 1 | 0 | 0 | 0 |
| OTU449 | 0 | 0 | 0 | 3 | 32 | 4 | 1 | 0 | 2 | 10 | 8 | 10 | 0 | 1 | 1 | 0 | 1 | 0 |
| OTU45 | 329 | 69 | 397 | 926 | 682 | 1049 | 94 | 18 | 14 | 29 | 66 | 25 | 2 | 25 | 17 | 0 | 9 | 7 |
| OTU450 | 2 | 6 | 3 | 2 | 2 | 5 | 4 | 3 | 1 | 4 | 6 | 5 | 2 | 5 | 2 | 2 | 10 | 1 |
| OTU451 | 9 | 12 | 9 | 8 | 8 | 21 | 8 | 21 | 11 | 23 | 23 | 22 | 4 | 12 | 13 | 3 | 30 | 8 |
| OTU452 | 0 | 4 | 0 | 4 | 5 | 11 | 1 | 0 | 0 | 1 | 2 | 9 | 0 | 3 | 0 | 0 | 0 | 0 |
| OTU453 | 4 | 1 | 0 | 3 | 11 | 12 | 0 | 14 | 11 | 22 | 19 | 12 | 1 | 0 | 7 | 0 | 1 | 0 |
| OTU454 | 1 | 12 | 0 | 0 | 11 | 3 | 4 | 1 | 8 | 4 | 5 | 5 | 1 | 1 | 5 | 0 | 0 | 0 |
| OTU455 | 1 | 6 | 5 | 0 | 4 | 7 | 0 | 0 | 2 | 5 | 3 | 2 | 1 | 0 | 1 | 0 | 0 | 0 |
| OTU456 | 0 | 0 | 0 | 0 | 1 | 1 | 22 | 5 | 32 | 4 | 8 | 10 | 0 | 0 | 21 | 0 | 1 | 0 |
| OTU457 | 0 | 0 | 0 | 0 | 14 | 0 | 0 | 0 | 0 | 0 | 0 | 0 | 0 | 0 | 0 | 0 | 0 | 0 |
| OTU458 | 0 | 2 | 0 | 0 | 0 | 5 | 3 | 15 | 4 | 14 | 7 | 0 | 0 | 0 | 3 | 0 | 0 | 1 |
| OTU459 | 4 | 7 | 4 | 2 | 6 | 7 | 5 | 5 | 7 | 9 | 10 | 4 | 1 | 9 | 3 | 8 | 5 | 4 |
| OTU46 | 3 | 82 | 2 | 118 | 1 | 223 | 10 | 33 | 265 | 9 | 240 | 169 | 8 | 10 | 198 | 0 | 7 | 14 |
| OTU460 | 0 | 0 | 0 | 1 | 0 | 1 | 3 | 62 | 1 | 0 | 5 | 4 | 0 | 6 | 2 | 0 | 0 | 0 |
| OTU461 | 1 | 20 | 12 | 0 | 1 | 5 | 11 | 11 | 7 | 13 | 8 | 16 | 0 | 5 | 6 | 0 | 1 | 1 |
| OTU462 | 0 | 0 | 0 | 0 | 0 | 0 | 3 | 3 | 3 | 12 | 8 | 2 | 2 | 2 | 2 | 0 | 1 | 1 |
| OTU463 | 2 | 0 | 0 | 1 | 2 | 3 | 4 | 3 | 11 | 0 | 4 | 3 | 1 | 1 | 6 | 0 | 0 | 0 |
| OTU464 | 2 | 0 | 0 | 0 | 0 | 0 | 3 | 6 | 0 | 33 | 58 | 0 | 0 | 0 | 0 | 0 | 0 | 1 |
| OTU465 | 0 | 0 | 0 | 0 | 0 | 1 | 6 | 18 | 8 | 25 | 12 | 2 | 1 | 1 | 15 | 0 | 1 | 0 |
| OTU466 | 0 | 0 | 0 | 0 | 0 | 0 | 0 | 2 | 0 | 1 | 6 | 26 | 0 | 0 | 0 | 0 | 0 | 1 |
| OTU467 | 0 | 3 | 2 | 1 | 4 | 6 | 0 | 10 | 3 | 19 | 14 | 0 | 0 | 0 | 1 | 0 | 0 | 0 |
| OTU468 | 0 | 0 | 0 | 0 | 0 | 0 | 6 | 7 | 13 | 19 | 13 | 30 | 5 | 5 | 8 | 0 | 0 | 2 |
| OTU469 | 0 | 0 | 0 | 1 | 0 | 2 | 0 | 29 | 0 | 2 | 1 | 1 | 1 | 0 | 1 | 0 | 1 | 0 |
| OTU47 | 215 | 145 | 255 | 444 | 289 | 418 | 168 | 58 | 65 | 153 | 124 | 145 | 13 | 36 | 63 | 0 | 5 | 7 |
| OTU470 | 0 | 0 | 4 | 1 | 0 | 2 | 2 | 10 | 7 | 4 | 6 | 4 | 1 | 1 | 8 | 0 | 0 | 0 |
| OTU471 | 0 | 5 | 5 | 0 | 1 | 0 | 0 | 6 | 5 | 2 | 0 | 5 | 3 | 11 | 1 | 0 | 1 | 1 |
| OTU472 | 1 | 0 | 0 | 0 | 0 | 0 | 2 | 4 | 4 | 1 | 0 | 12 | 3 | 7 | 8 | 0 | 0 | 1 |
| OTU473 | 0 | 1 | 0 | 1 | 1 | 0 | 2 | 6 | 0 | 3 | 4 | 4 | 0 | 1 | 1 | 0 | 1 | 0 |
| OTU474 | 0 | 0 | 0 | 0 | 0 | 0 | 0 | 1 | 2 | 7 | 2 | 8 | 0 | 0 | 5 | 0 | 0 | 0 |
| OTU475 | 1 | 7 | 5 | 3 | 3 | 2 | 1 | 4 | 6 | 4 | 5 | 4 | 3 | 6 | 1 | 6 | 4 | 2 |
| OTU476 | 11 | 28 | 48 | 37 | 106 | 103 | 26 | 517 | 360 | 490 | 338 | 576 | 13 | 47 | 216 | 0 | 30 | 15 |
| OTU477 | 0 | 0 | 0 | 0 | 0 | 0 | 5 | 3 | 6 | 7 | 15 | 15 | 3 | 8 | 13 | 0 | 2 | 0 |
| OTU478 | 0 | 0 | 0 | 0 | 0 | 0 | 0 | 0 | 0 | 0 | 0 | 0 | 8 | 1 | 0 | 0 | 1 | 6 |
| OTU479 | 0 | 0 | 0 | 0 | 0 | 0 | 0 | 0 | 0 | 0 | 0 | 0 | 10 | 2 | 0 | 0 | 29 | 8 |
| OTU48 | 164 | 271 | 263 | 458 | 326 | 131 | 3 | 0 | 1 | 3 | 2 | 2 | 0 | 1 | 1 | 0 | 2 | 1 |
| OTU480 | 0 | 0 | 0 | 0 | 0 | 0 | 0 | 0 | 0 | 0 | 0 | 0 | 15 | 2 | 0 | 0 | 17 | 7 |
| OTU481 | 0 | 0 | 0 | 0 | 0 | 0 | 0 | 0 | 1 | 1 | 0 | 0 | 1 | 16 | 0 | 0 | 21 | 2 |
| OTU482 | 0 | 0 | 0 | 0 | 0 | 0 | 0 | 1 | 1 | 0 | 0 | 0 | 0 | 1 | 0 | 0 | 80 | 0 |
| OTU483 | 0 | 0 | 0 | 0 | 0 | 0 | 0 | 0 | 0 | 0 | 0 | 0 | 0 | 0 | 0 | 0 | 86 | 0 |
| OTU484 | 0 | 0 | 1 | 0 | 0 | 0 | 0 | 0 | 0 | 0 | 0 | 0 | 0 | 0 | 0 | 0 | 42 | 0 |
| OTU485 | 2 | 0 | 0 | 0 | 0 | 0 | 1 | 0 | 0 | 0 | 0 | 0 | 0 | 0 | 0 | 0 | 58 | 0 |
| OTU486 | 0 | 0 | 0 | 0 | 0 | 0 | 0 | 0 | 0 | 0 | 0 | 0 | 0 | 0 | 0 | 1 | 66 | 2 |
| OTU487 | 1 | 0 | 0 | 0 | 0 | 1 | 0 | 0 | 1 | 1 | 0 | 0 | 3 | 4 | 3 | 2 | 27 | 10 |
| OTU488 | 11 | 0 | 6 | 11 | 0 | 20 | 0 | 0 | 0 | 0 | 0 | 0 | 0 | 0 | 0 | 0 | 0 | 0 |
| OTU489 | 11 | 4 | 5 | 4 | 4 | 4 | 0 | 1 | 5 | 1 | 2 | 6 | 0 | 1 | 4 | 0 | 0 | 0 |
| OTU49 | 296 | 359 | 474 | 438 | 377 | 249 | 72 | 45 | 20 | 77 | 20 | 35 | 3 | 7 | 10 | 0 | 6 | 4 |
| OTU490 | 10 | 13 | 23 | 6 | 2 | 16 | 2 | 26 | 5 | 12 | 13 | 12 | 0 | 1 | 6 | 0 | 4 | 0 |
| OTU491 | 4 | 4 | 6 | 4 | 10 | 40 | 1 | 0 | 4 | 1 | 1 | 5 | 0 | 0 | 1 | 0 | 0 | 0 |
| OTU492 | 11 | 4 | 0 | 1 | 1 | 28 | 0 | 0 | 1 | 0 | 1 | 0 | 0 | 0 | 0 | 0 | 0 | 0 |
| OTU493 | 3 | 3 | 5 | 1 | 3 | 1 | 2 | 2 | 1 | 0 | 1 | 3 | 0 | 1 | 0 | 0 | 0 | 0 |
| OTU494 | 7 | 2 | 2 | 4 | 9 | 0 | 3 | 3 | 2 | 4 | 6 | 5 | 1 | 2 | 8 | 0 | 0 | 0 |
| OTU495 | 6 | 12 | 10 | 0 | 0 | 0 | 0 | 0 | 0 | 0 | 0 | 0 | 0 | 0 | 0 | 0 | 0 | 0 |
| OTU496 | 9 | 0 | 14 | 9 | 7 | 7 | 0 | 0 | 0 | 0 | 0 | 0 | 0 | 0 | 0 | 0 | 0 | 0 |
| OTU497 | 3 | 3 | 0 | 3 | 11 | 3 | 0 | 0 | 0 | 0 | 0 | 0 | 0 | 0 | 0 | 0 | 0 | 0 |
| OTU498 | 3 | 4 | 3 | 2 | 1 | 4 | 0 | 4 | 3 | 1 | 7 | 6 | 4 | 7 | 3 | 3 | 6 | 3 |
| OTU499 | 3 | 1 | 6 | 0 | 0 | 2 | 0 | 0 | 0 | 0 | 0 | 0 | 0 | 0 | 0 | 0 | 3 | 1 |
| OTU5 | 3663 | 12707 | 1740 | 1318 | 1441 | 1321 | 0 | 7 | 4 | 7 | 7 | 10 | 6 | 6 | 1 | 3 | 14 | 3 |
| OTU50 | 6 | 55 | 1515 | 8 | 7 | 10 | 1 | 1 | 0 | 0 | 3 | 1 | 0 | 0 | 1 | 0 | 2 | 0 |
| OTU500 | 11 | 1 | 6 | 0 | 1 | 6 | 2 | 1 | 5 | 0 | 0 | 4 | 0 | 0 | 0 | 0 | 3 | 1 |
| OTU501 | 9 | 11 | 4 | 0 | 2 | 1 | 1 | 0 | 1 | 1 | 1 | 3 | 0 | 0 | 0 | 0 | 0 | 0 |
| OTU502 | 38 | 1 | 0 | 0 | 0 | 8 | 0 | 0 | 0 | 0 | 0 | 0 | 0 | 0 | 0 | 0 | 0 | 0 |
| OTU503 | 44 | 1 | 4 | 3 | 0 | 4 | 3 | 0 | 3 | 0 | 5 | 1 | 0 | 1 | 5 | 0 | 1 | 0 |
| OTU504 | 7 | 1 | 3 | 9 | 2 | 14 | 0 | 0 | 0 | 0 | 0 | 0 | 0 | 0 | 0 | 0 | 0 | 0 |
| OTU505 | 4 | 11 | 8 | 7 | 3 | 10 | 1 | 0 | 0 | 0 | 0 | 0 | 0 | 0 | 0 | 0 | 0 | 0 |
| OTU506 | 1 | 4 | 0 | 3 | 7 | 1 | 2 | 2 | 1 | 0 | 1 | 0 | 0 | 0 | 1 | 0 | 0 | 1 |
| OTU507 | 0 | 1 | 0 | 0 | 5 | 2 | 0 | 2 | 0 | 2 | 3 | 0 | 0 | 0 | 3 | 0 | 0 | 0 |
| OTU508 | 2 | 3 | 23 | 3 | 1 | 0 | 5 | 0 | 0 | 0 | 0 | 0 | 0 | 0 | 0 | 0 | 0 | 0 |
| OTU509 | 7 | 7 | 7 | 1 | 0 | 5 | 0 | 0 | 0 | 0 | 0 | 0 | 0 | 0 | 0 | 0 | 0 | 0 |
| OTU51 | 1 | 1 | 126 | 847 | 37 | 4 | 63 | 27 | 1 | 26 | 96 | 52 | 8 | 11 | 0 | 1 | 9 | 5 |
| OTU510 | 0 | 1 | 2 | 3 | 1 | 2 | 0 | 12 | 1 | 1 | 1 | 0 | 0 | 0 | 0 | 0 | 0 | 0 |
| OTU511 | 3 | 5 | 4 | 0 | 5 | 4 | 0 | 0 | 0 | 0 | 1 | 0 | 0 | 0 | 0 | 0 | 0 | 0 |
| OTU512 | 0 | 48 | 0 | 0 | 0 | 0 | 0 | 0 | 0 | 0 | 0 | 0 | 0 | 0 | 0 | 0 | 0 | 0 |
| OTU513 | 1 | 4 | 5 | 3 | 10 | 16 | 1 | 4 | 3 | 12 | 6 | 2 | 0 | 0 | 3 | 0 | 0 | 0 |
| OTU514 | 0 | 2 | 54 | 0 | 0 | 1 | 4 | 19 | 12 | 19 | 6 | 15 | 0 | 1 | 8 | 0 | 1 | 0 |
| OTU515 | 0 | 2 | 2 | 0 | 16 | 0 | 1 | 5 | 5 | 3 | 2 | 2 | 0 | 1 | 2 | 0 | 1 | 1 |
| OTU516 | 0 | 2 | 1 | 0 | 0 | 0 | 1 | 3 | 0 | 9 | 5 | 1 | 0 | 0 | 0 | 0 | 0 | 0 |
| OTU517 | 7 | 1 | 0 | 17 | 5 | 12 | 3 | 4 | 1 | 1 | 1 | 0 | 0 | 1 | 3 | 0 | 0 | 0 |
| OTU518 | 1 | 0 | 1 | 5 | 0 | 8 | 2 | 0 | 0 | 0 | 0 | 1 | 1 | 0 | 0 | 0 | 2 | 0 |
| OTU519 | 5 | 1 | 2 | 4 | 2 | 3 | 1 | 0 | 0 | 0 | 0 | 2 | 0 | 0 | 0 | 0 | 0 | 0 |
| OTU52 | 3 | 0 | 2 | 3 | 1 | 4 | 682 | 172 | 5 | 179 | 154 | 89 | 10 | 17 | 22 | 1 | 7 | 6 |
| OTU520 | 2 | 6 | 2 | 7 | 11 | 6 | 0 | 0 | 0 | 0 | 0 | 0 | 0 | 0 | 0 | 0 | 0 | 0 |
| OTU521 | 1 | 0 | 0 | 0 | 2 | 2 | 1 | 1 | 1 | 1 | 3 | 2 | 1 | 1 | 1 | 0 | 1 | 0 |
| OTU522 | 3 | 1 | 0 | 1 | 1 | 1 | 0 | 2 | 3 | 1 | 5 | 4 | 1 | 0 | 2 | 2 | 3 | 2 |
| OTU523 | 1 | 0 | 0 | 1 | 1 | 1 | 1 | 0 | 0 | 3 | 1 | 0 | 1 | 1 | 1 | 1 | 0 | 1 |
| OTU524 | 1 | 0 | 2 | 1 | 29 | 1 | 0 | 1 | 2 | 1 | 13 | 1 | 1 | 0 | 1 | 0 | 1 | 0 |
| OTU525 | 0 | 0 | 0 | 0 | 0 | 5 | 0 | 0 | 5 | 0 | 0 | 0 | 0 | 0 | 0 | 0 | 0 | 0 |
| OTU526 | 1 | 0 | 0 | 0 | 0 | 0 | 10 | 1 | 5 | 21 | 4 | 1 | 0 | 0 | 3 | 0 | 0 | 0 |
| OTU527 | 8 | 0 | 3 | 0 | 2 | 2 | 18 | 4 | 12 | 3 | 4 | 7 | 2 | 1 | 7 | 0 | 0 | 0 |
| OTU528 | 0 | 0 | 0 | 0 | 0 | 0 | 8 | 0 | 17 | 10 | 1 | 3 | 0 | 0 | 7 | 0 | 0 | 1 |
| OTU529 | 0 | 0 | 0 | 0 | 0 | 0 | 2 | 6 | 3 | 3 | 0 | 5 | 0 | 4 | 3 | 0 | 0 | 2 |
| OTU53 | 3 | 8 | 71 | 2 | 49 | 96 | 72 | 190 | 249 | 52 | 912 | 110 | 3 | 4 | 219 | 0 | 21 | 5 |
| OTU530 | 0 | 1 | 0 | 1 | 1 | 1 | 18 | 9 | 2 | 12 | 6 | 8 | 2 | 2 | 1 | 0 | 2 | 0 |
| OTU531 | 0 | 0 | 0 | 0 | 0 | 0 | 2 | 1 | 1 | 1 | 3 | 2 | 1 | 1 | 5 | 0 | 0 | 0 |
| OTU532 | 0 | 0 | 0 | 0 | 0 | 0 | 9 | 0 | 0 | 0 | 0 | 0 | 0 | 0 | 0 | 0 | 0 | 0 |
| OTU533 | 2 | 0 | 0 | 2 | 7 | 0 | 14 | 2 | 2 | 2 | 1 | 0 | 0 | 0 | 1 | 0 | 0 | 0 |
| OTU534 | 0 | 0 | 0 | 0 | 0 | 0 | 4 | 4 | 3 | 30 | 4 | 4 | 0 | 2 | 0 | 0 | 0 | 0 |
| OTU535 | 1 | 0 | 0 | 0 | 0 | 0 | 1 | 28 | 4 | 2 | 0 | 0 | 0 | 0 | 3 | 0 | 1 | 1 |
| OTU536 | 0 | 1 | 0 | 0 | 0 | 0 | 0 | 7 | 2 | 7 | 3 | 1 | 0 | 0 | 2 | 0 | 0 | 0 |
| OTU537 | 0 | 0 | 0 | 0 | 0 | 0 | 0 | 5 | 1 | 0 | 1 | 4 | 0 | 0 | 1 | 0 | 1 | 0 |
| OTU538 | 16 | 3 | 4 | 1 | 0 | 14 | 7 | 5 | 9 | 0 | 0 | 5 | 0 | 1 | 4 | 0 | 1 | 0 |
| OTU539 | 1 | 0 | 2 | 0 | 2 | 4 | 2 | 7 | 0 | 11 | 3 | 1 | 0 | 0 | 1 | 0 | 0 | 0 |
| OTU54 | 222 | 160 | 234 | 282 | 524 | 501 | 4 | 1 | 1 | 5 | 4 | 0 | 0 | 3 | 5 | 0 | 2 | 2 |
| OTU540 | 0 | 0 | 0 | 0 | 0 | 0 | 0 | 9 | 8 | 0 | 0 | 6 | 1 | 0 | 10 | 0 | 0 | 0 |
| OTU541 | 0 | 0 | 0 | 0 | 0 | 0 | 1 | 6 | 11 | 1 | 2 | 3 | 0 | 0 | 2 | 0 | 0 | 0 |
| OTU542 | 1 | 0 | 0 | 0 | 0 | 2 | 1 | 4 | 4 | 3 | 5 | 4 | 0 | 1 | 10 | 0 | 0 | 0 |
| OTU543 | 0 | 1 | 0 | 0 | 0 | 2 | 1 | 29 | 0 | 50 | 5 | 3 | 0 | 0 | 1 | 0 | 0 | 1 |
| OTU544 | 0 | 5 | 2 | 1 | 0 | 14 | 0 | 15 | 1 | 24 | 24 | 5 | 2 | 2 | 5 | 0 | 1 | 0 |
| OTU545 | 10 | 4 | 5 | 7 | 1 | 2 | 4 | 13 | 3 | 2 | 3 | 6 | 0 | 0 | 2 | 0 | 0 | 0 |
| OTU546 | 0 | 0 | 0 | 0 | 0 | 0 | 0 | 1 | 8 | 0 | 0 | 24 | 0 | 1 | 9 | 0 | 0 | 0 |
| OTU547 | 0 | 0 | 0 | 0 | 0 | 4 | 13 | 31 | 11 | 9 | 16 | 31 | 2 | 5 | 14 | 0 | 1 | 0 |
| OTU548 | 1 | 0 | 0 | 0 | 0 | 1 | 2 | 2 | 5 | 25 | 1 | 3 | 0 | 1 | 1 | 0 | 2 | 2 |
| OTU549 | 0 | 0 | 0 | 0 | 0 | 0 | 6 | 0 | 0 | 15 | 2 | 1 | 0 | 12 | 2 | 0 | 0 | 2 |
| OTU55 | 80 | 144 | 212 | 241 | 232 | 218 | 47 | 16 | 127 | 1896 | 1661 | 29 | 47 | 63 | 101 | 1 | 26 | 37 |
| OTU550 | 0 | 0 | 0 | 0 | 0 | 0 | 4 | 0 | 1 | 2 | 0 | 4 | 0 | 1 | 0 | 0 | 0 | 0 |
| OTU551 | 0 | 1 | 0 | 0 | 0 | 0 | 3 | 1 | 0 | 8 | 14 | 4 | 1 | 1 | 3 | 0 | 0 | 0 |
| OTU552 | 1 | 4 | 6 | 2 | 2 | 3 | 1 | 5 | 4 | 2 | 4 | 8 | 0 | 2 | 2 | 0 | 0 | 0 |
| OTU553 | 0 | 0 | 0 | 0 | 0 | 1 | 1 | 0 | 6 | 0 | 20 | 0 | 0 | 1 | 3 | 0 | 1 | 0 |
| OTU554 | 0 | 0 | 0 | 0 | 0 | 0 | 0 | 1 | 0 | 0 | 0 | 8 | 0 | 2 | 0 | 0 | 0 | 0 |
| OTU555 | 0 | 0 | 0 | 0 | 0 | 0 | 0 | 0 | 0 | 0 | 0 | 0 | 2 | 5 | 0 | 1 | 10 | 6 |
| OTU556 | 0 | 0 | 0 | 0 | 0 | 0 | 0 | 0 | 0 | 0 | 0 | 0 | 9 | 1 | 0 | 0 | 25 | 12 |
| OTU557 | 8 | 3 | 3 | 0 | 5 | 0 | 0 | 0 | 0 | 0 | 0 | 0 | 5 | 0 | 0 | 0 | 15 | 1 |
| OTU558 | 0 | 0 | 0 | 0 | 0 | 0 | 0 | 0 | 0 | 0 | 0 | 0 | 0 | 2 | 0 | 1 | 18 | 2 |
| OTU559 | 0 | 0 | 0 | 0 | 0 | 0 | 1 | 3 | 1 | 1 | 1 | 1 | 1 | 3 | 0 | 4 | 19 | 3 |
| OTU56 | 0 | 10 | 5 | 17 | 3 | 31 | 11 | 528 | 154 | 331 | 83 | 123 | 4 | 6 | 142 | 0 | 11 | 3 |
| OTU560 | 0 | 0 | 0 | 0 | 0 | 0 | 0 | 0 | 0 | 0 | 0 | 0 | 1 | 1 | 0 | 1 | 19 | 3 |
| OTU561 | 0 | 0 | 1 | 0 | 2 | 0 | 0 | 0 | 0 | 0 | 0 | 0 | 1 | 0 | 1 | 0 | 62 | 1 |
| OTU562 | 0 | 1 | 0 | 1 | 0 | 0 | 0 | 0 | 0 | 0 | 0 | 0 | 7 | 5 | 0 | 0 | 71 | 19 |
| OTU563 | 0 | 0 | 0 | 0 | 0 | 0 | 0 | 0 | 0 | 0 | 0 | 0 | 0 | 0 | 0 | 0 | 35 | 0 |
| OTU564 | 0 | 0 | 0 | 0 | 0 | 0 | 0 | 0 | 0 | 0 | 0 | 0 | 0 | 0 | 0 | 0 | 152 | 1 |
| OTU565 | 0 | 0 | 0 | 0 | 0 | 0 | 0 | 0 | 0 | 0 | 0 | 0 | 1 | 1 | 0 | 1 | 14 | 17 |
| OTU566 | 2 | 1 | 1 | 1 | 0 | 0 | 0 | 0 | 0 | 0 | 0 | 0 | 0 | 0 | 0 | 0 | 1 | 0 |
| OTU567 | 10 | 0 | 0 | 0 | 3 | 0 | 0 | 0 | 0 | 0 | 0 | 1 | 1 | 1 | 0 | 0 | 0 | 0 |
| OTU568 | 2 | 2 | 18 | 0 | 0 | 0 | 0 | 0 | 0 | 0 | 0 | 0 | 0 | 0 | 0 | 0 | 0 | 0 |
| OTU569 | 5 | 1 | 1 | 1 | 0 | 0 | 0 | 0 | 1 | 1 | 1 | 1 | 3 | 1 | 0 | 4 | 4 | 3 |
| OTU57 | 0 | 0 | 0 | 0 | 2 | 0 | 39 | 193 | 218 | 228 | 67 | 195 | 2 | 4 | 99 | 0 | 3 | 1 |
| OTU570 | 3 | 3 | 0 | 0 | 6 | 2 | 0 | 4 | 1 | 2 | 0 | 1 | 0 | 2 | 3 | 0 | 0 | 2 |
| OTU571 | 8 | 4 | 8 | 1 | 5 | 3 | 1 | 3 | 2 | 1 | 1 | 2 | 0 | 4 | 2 | 0 | 1 | 1 |
| OTU572 | 9 | 4 | 2 | 0 | 7 | 0 | 0 | 0 | 0 | 0 | 0 | 0 | 0 | 0 | 0 | 0 | 0 | 0 |
| OTU573 | 5 | 1 | 1 | 1 | 1 | 4 | 0 | 0 | 0 | 1 | 1 | 0 | 0 | 0 | 0 | 0 | 0 | 0 |
| OTU574 | 12 | 0 | 30 | 0 | 1 | 0 | 1 | 0 | 0 | 1 | 0 | 2 | 0 | 0 | 0 | 0 | 0 | 0 |
| OTU575 | 1 | 1 | 0 | 2 | 1 | 2 | 0 | 0 | 0 | 0 | 0 | 0 | 0 | 0 | 0 | 0 | 0 | 0 |
| OTU576 | 4 | 2 | 1 | 0 | 1 | 3 | 0 | 7 | 3 | 0 | 0 | 3 | 0 | 1 | 6 | 0 | 1 | 0 |
| OTU577 | 7 | 1 | 1 | 8 | 1 | 0 | 8 | 6 | 0 | 3 | 0 | 4 | 1 | 2 | 3 | 0 | 0 | 0 |
| OTU578 | 1 | 2 | 0 | 4 | 1 | 4 | 1 | 1 | 1 | 1 | 0 | 2 | 0 | 1 | 1 | 0 | 0 | 0 |
| OTU579 | 7 | 1 | 0 | 0 | 4 | 1 | 4 | 1 | 2 | 0 | 1 | 1 | 5 | 3 | 1 | 6 | 3 | 7 |
| OTU58 | 513 | 16 | 343 | 13 | 33 | 109 | 570 | 54 | 69 | 75 | 40 | 34 | 1 | 19 | 62 | 0 | 10 | 2 |
| OTU580 | 11 | 0 | 0 | 0 | 0 | 0 | 2 | 0 | 0 | 6 | 0 | 0 | 0 | 0 | 0 | 0 | 0 | 0 |
| OTU581 | 12 | 0 | 0 | 0 | 1 | 0 | 0 | 0 | 0 | 0 | 0 | 0 | 0 | 0 | 0 | 0 | 0 | 0 |
| OTU582 | 17 | 0 | 0 | 0 | 0 | 0 | 0 | 0 | 0 | 0 | 0 | 0 | 0 | 0 | 0 | 0 | 0 | 0 |
| OTU583 | 1 | 0 | 6 | 1 | 2 | 4 | 0 | 0 | 0 | 0 | 0 | 0 | 0 | 0 | 0 | 0 | 0 | 0 |
| OTU584 | 5 | 11 | 0 | 8 | 4 | 0 | 0 | 0 | 0 | 0 | 0 | 0 | 0 | 0 | 0 | 0 | 1 | 0 |
| OTU585 | 0 | 20 | 20 | 0 | 0 | 0 | 0 | 0 | 0 | 7 | 1 | 0 | 0 | 0 | 2 | 0 | 0 | 0 |
| OTU586 | 0 | 3 | 3 | 3 | 2 | 0 | 1 | 3 | 0 | 0 | 1 | 0 | 2 | 5 | 4 | 2 | 0 | 0 |
| OTU587 | 1 | 4 | 4 | 3 | 7 | 5 | 2 | 5 | 1 | 1 | 1 | 2 | 0 | 3 | 2 | 0 | 1 | 0 |
| OTU588 | 2 | 25 | 6 | 5 | 9 | 7 | 0 | 9 | 5 | 9 | 10 | 2 | 0 | 0 | 4 | 0 | 1 | 0 |
| OTU589 | 1 | 1 | 2 | 4 | 0 | 2 | 2 | 0 | 6 | 0 | 0 | 0 | 0 | 0 | 2 | 0 | 0 | 0 |
| OTU59 | 227 | 16 | 96 | 401 | 74 | 204 | 4 | 2 | 3 | 0 | 0 | 1 | 0 | 1 | 1 | 0 | 4 | 1 |
| OTU590 | 0 | 7 | 1 | 0 | 6 | 0 | 0 | 0 | 0 | 1 | 3 | 2 | 0 | 0 | 0 | 0 | 0 | 0 |
| OTU591 | 2 | 5 | 0 | 3 | 0 | 6 | 4 | 0 | 0 | 1 | 0 | 2 | 1 | 2 | 0 | 0 | 0 | 0 |
| OTU592 | 8 | 2 | 0 | 0 | 6 | 15 | 0 | 0 | 0 | 0 | 0 | 0 | 0 | 0 | 0 | 0 | 0 | 0 |
| OTU593 | 6 | 15 | 0 | 0 | 0 | 3 | 0 | 0 | 0 | 0 | 0 | 0 | 0 | 0 | 0 | 0 | 0 | 0 |
| OTU594 | 0 | 0 | 3 | 0 | 0 | 0 | 0 | 0 | 0 | 0 | 1 | 0 | 0 | 0 | 0 | 0 | 0 | 0 |
| OTU595 | 1 | 0 | 1 | 0 | 1 | 1 | 0 | 7 | 7 | 4 | 5 | 2 | 1 | 1 | 4 | 0 | 0 | 0 |
| OTU596 | 4 | 0 | 3 | 1 | 0 | 5 | 0 | 1 | 5 | 13 | 12 | 4 | 1 | 0 | 3 | 0 | 1 | 0 |
| OTU597 | 0 | 0 | 2 | 0 | 2 | 0 | 2 | 0 | 2 | 1 | 2 | 0 | 0 | 1 | 0 | 2 | 5 | 1 |
| OTU598 | 0 | 1 | 4 | 7 | 1 | 2 | 0 | 1 | 0 | 0 | 1 | 0 | 0 | 1 | 0 | 0 | 0 | 0 |
| OTU599 | 0 | 1 | 0 | 3 | 7 | 8 | 1 | 0 | 4 | 8 | 3 | 3 | 0 | 0 | 3 | 0 | 0 | 0 |
| OTU6 | 5913 | 2348 | 6104 | 7558 | 6115 | 9119 | 94 | 32 | 41 | 74 | 74 | 59 | 8 | 14 | 39 | 1 | 30 | 12 |
| OTU60 | 3 | 4 | 7 | 7 | 4 | 9 | 233 | 108 | 425 | 204 | 70 | 284 | 38 | 111 | 445 | 0 | 7 | 13 |
| OTU600 | 0 | 0 | 1 | 1 | 1 | 2 | 0 | 0 | 0 | 0 | 0 | 0 | 0 | 0 | 0 | 0 | 0 | 0 |
| OTU601 | 1 | 1 | 2 | 3 | 3 | 3 | 1 | 4 | 1 | 3 | 0 | 4 | 0 | 1 | 4 | 0 | 0 | 0 |
| OTU602 | 0 | 0 | 0 | 2 | 1 | 1 | 1 | 2 | 2 | 2 | 1 | 0 | 2 | 0 | 0 | 0 | 0 | 0 |
| OTU603 | 0 | 0 | 1 | 2 | 2 | 0 | 1 | 2 | 0 | 0 | 0 | 1 | 0 | 2 | 0 | 0 | 0 | 0 |
| OTU604 | 2 | 3 | 2 | 6 | 8 | 18 | 1 | 0 | 2 | 0 | 0 | 4 | 0 | 0 | 0 | 0 | 0 | 0 |
| OTU605 | 2 | 0 | 0 | 3 | 7 | 0 | 0 | 0 | 0 | 1 | 0 | 2 | 0 | 0 | 0 | 0 | 0 | 0 |
| OTU606 | 1 | 0 | 0 | 7 | 2 | 3 | 0 | 0 | 3 | 6 | 2 | 2 | 0 | 0 | 3 | 0 | 0 | 0 |
| OTU607 | 6 | 2 | 8 | 3 | 23 | 5 | 10 | 24 | 18 | 45 | 50 | 7 | 3 | 2 | 17 | 0 | 4 | 3 |
| OTU608 | 2 | 0 | 2 | 18 | 4 | 0 | 0 | 0 | 0 | 0 | 0 | 0 | 0 | 0 | 0 | 0 | 0 | 0 |
| OTU609 | 0 | 0 | 5 | 6 | 0 | 0 | 0 | 1 | 1 | 0 | 0 | 0 | 0 | 0 | 1 | 0 | 0 | 0 |
| OTU61 | 117 | 78 | 122 | 193 | 398 | 603 | 100 | 59 | 79 | 49 | 58 | 101 | 9 | 25 | 36 | 0 | 5 | 2 |
| OTU610 | 1 | 0 | 0 | 37 | 0 | 3 | 0 | 1 | 1 | 0 | 1 | 0 | 0 | 0 | 0 | 0 | 0 | 0 |
| OTU611 | 2 | 1 | 3 | 4 | 0 | 12 | 1 | 2 | 1 | 1 | 0 | 3 | 0 | 2 | 0 | 0 | 0 | 0 |
| OTU612 | 0 | 0 | 0 | 0 | 4 | 10 | 0 | 0 | 0 | 3 | 0 | 0 | 0 | 0 | 0 | 0 | 0 | 0 |
| OTU613 | 0 | 0 | 0 | 0 | 2 | 16 | 0 | 0 | 0 | 0 | 0 | 1 | 0 | 0 | 0 | 0 | 0 | 0 |
| OTU614 | 1 | 0 | 0 | 0 | 4 | 2 | 1 | 2 | 1 | 0 | 0 | 2 | 0 | 0 | 1 | 0 | 0 | 0 |
| OTU615 | 1 | 0 | 1 | 2 | 5 | 2 | 0 | 0 | 0 | 0 | 0 | 0 | 0 | 0 | 0 | 0 | 0 | 0 |
| OTU616 | 0 | 1 | 3 | 0 | 3 | 0 | 1 | 0 | 1 | 1 | 1 | 1 | 0 | 0 | 1 | 0 | 0 | 0 |
| OTU617 | 0 | 2 | 0 | 1 | 10 | 8 | 0 | 4 | 4 | 1 | 0 | 3 | 0 | 0 | 3 | 0 | 1 | 1 |
| OTU618 | 0 | 0 | 2 | 1 | 0 | 10 | 0 | 1 | 0 | 0 | 0 | 0 | 0 | 0 | 0 | 0 | 0 | 0 |
| OTU619 | 0 | 0 | 1 | 0 | 2 | 11 | 3 | 8 | 3 | 7 | 5 | 4 | 0 | 3 | 2 | 0 | 0 | 0 |
| OTU62 | 1 | 1 | 1 | 0 | 0 | 1 | 0 | 50 | 199 | 3 | 4 | 425 | 22 | 58 | 129 | 0 | 8 | 1 |
| OTU620 | 0 | 0 | 1 | 3 | 1 | 4 | 0 | 11 | 7 | 4 | 3 | 2 | 0 | 0 | 1 | 0 | 0 | 0 |
| OTU621 | 0 | 1 | 1 | 0 | 4 | 8 | 0 | 5 | 10 | 4 | 4 | 8 | 0 | 1 | 3 | 0 | 4 | 0 |
| OTU622 | 4 | 4 | 1 | 2 | 9 | 8 | 1 | 3 | 8 | 4 | 8 | 7 | 2 | 6 | 3 | 3 | 4 | 1 |
| OTU623 | 9 | 5 | 4 | 3 | 3 | 8 | 1 | 11 | 7 | 4 | 11 | 7 | 0 | 2 | 7 | 0 | 3 | 0 |
| OTU624 | 1 | 3 | 1 | 2 | 2 | 2 | 1 | 3 | 1 | 2 | 2 | 2 | 0 | 0 | 2 | 0 | 0 | 0 |
| OTU625 | 0 | 0 | 0 | 0 | 0 | 27 | 0 | 0 | 0 | 1 | 0 | 0 | 0 | 0 | 0 | 0 | 0 | 0 |
| OTU626 | 15 | 0 | 7 | 0 | 0 | 0 | 48 | 0 | 0 | 0 | 0 | 0 | 0 | 0 | 2 | 0 | 0 | 0 |
| OTU627 | 0 | 0 | 0 | 0 | 0 | 0 | 8 | 1 | 0 | 1 | 0 | 0 | 1 | 0 | 1 | 0 | 0 | 0 |
| OTU628 | 0 | 0 | 0 | 0 | 0 | 1 | 14 | 0 | 2 | 3 | 1 | 4 | 0 | 2 | 1 | 0 | 0 | 0 |
| OTU629 | 0 | 0 | 0 | 0 | 0 | 0 | 2 | 3 | 5 | 0 | 0 | 1 | 1 | 1 | 3 | 0 | 0 | 0 |
| OTU63 | 2 | 2 | 4 | 5 | 2 | 6 | 133 | 96 | 156 | 122 | 229 | 62 | 12 | 41 | 225 | 0 | 4 | 10 |
| OTU630 | 0 | 0 | 1 | 0 | 0 | 0 | 3 | 0 | 2 | 0 | 0 | 2 | 0 | 0 | 1 | 0 | 0 | 0 |
| OTU631 | 0 | 0 | 0 | 1 | 0 | 2 | 2 | 7 | 2 | 16 | 13 | 3 | 0 | 0 | 2 | 0 | 0 | 0 |
| OTU632 | 1 | 0 | 0 | 0 | 0 | 0 | 4 | 3 | 2 | 2 | 14 | 2 | 0 | 2 | 3 | 0 | 0 | 0 |
| OTU633 | 0 | 1 | 0 | 0 | 0 | 2 | 2 | 14 | 6 | 7 | 9 | 4 | 0 | 1 | 7 | 0 | 1 | 0 |
| OTU634 | 0 | 0 | 0 | 0 | 0 | 0 | 3 | 4 | 0 | 0 | 0 | 4 | 0 | 0 | 0 | 0 | 0 | 0 |
| OTU635 | 0 | 2 | 0 | 0 | 0 | 0 | 2 | 0 | 1 | 1 | 0 | 2 | 0 | 0 | 3 | 0 | 0 | 0 |
| OTU636 | 2 | 0 | 0 | 0 | 0 | 0 | 8 | 2 | 4 | 3 | 5 | 2 | 0 | 1 | 2 | 0 | 0 | 0 |
| OTU637 | 0 | 3 | 1 | 3 | 1 | 2 | 2 | 4 | 1 | 2 | 1 | 2 | 0 | 0 | 1 | 2 | 3 | 1 |
| OTU638 | 5 | 0 | 3 | 2 | 0 | 1 | 1 | 3 | 0 | 0 | 5 | 0 | 0 | 0 | 1 | 0 | 0 | 0 |
| OTU639 | 0 | 0 | 4 | 0 | 1 | 0 | 13 | 3 | 0 | 0 | 1 | 5 | 0 | 1 | 1 | 0 | 0 | 0 |
| OTU64 | 0 | 1 | 1 | 0 | 0 | 1 | 3 | 3 | 0 | 2 | 1 | 2 | 12 | 39 | 1 | 38 | 552 | 152 |
| OTU640 | 1 | 2 | 1 | 0 | 0 | 4 | 1 | 4 | 1 | 0 | 1 | 2 | 1 | 1 | 0 | 1 | 7 | 1 |
| OTU641 | 0 | 0 | 1 | 0 | 0 | 0 | 2 | 6 | 0 | 0 | 1 | 1 | 0 | 1 | 1 | 0 | 1 | 0 |
| OTU642 | 0 | 5 | 1 | 0 | 0 | 2 | 0 | 3 | 1 | 0 | 0 | 0 | 0 | 0 | 2 | 0 | 0 | 0 |
| OTU643 | 0 | 0 | 0 | 2 | 0 | 2 | 0 | 7 | 4 | 15 | 3 | 3 | 0 | 0 | 6 | 0 | 0 | 0 |
| OTU644 | 0 | 0 | 0 | 0 | 0 | 0 | 6 | 3 | 3 | 5 | 0 | 0 | 0 | 0 | 1 | 0 | 0 | 0 |
| OTU645 | 1 | 2 | 0 | 0 | 0 | 0 | 2 | 5 | 0 | 3 | 4 | 2 | 1 | 0 | 1 | 1 | 1 | 0 |
| OTU646 | 0 | 0 | 0 | 0 | 0 | 0 | 1 | 11 | 32 | 1 | 0 | 1 | 0 | 0 | 9 | 0 | 0 | 0 |
| OTU647 | 0 | 1 | 1 | 0 | 2 | 0 | 1 | 10 | 1 | 2 | 14 | 3 | 0 | 0 | 1 | 0 | 0 | 0 |
| OTU648 | 0 | 0 | 0 | 0 | 0 | 0 | 0 | 3 | 1 | 3 | 0 | 4 | 1 | 3 | 0 | 0 | 0 | 0 |
| OTU649 | 2 | 2 | 9 | 3 | 0 | 5 | 1 | 3 | 2 | 1 | 1 | 2 | 1 | 2 | 2 | 0 | 0 | 0 |
| OTU65 | 0 | 0 | 1 | 0 | 0 | 0 | 167 | 37 | 99 | 55 | 57 | 91 | 379 | 26 | 169 | 1 | 14 | 7 |
| OTU650 | 7 | 9 | 8 | 1 | 0 | 14 | 0 | 17 | 6 | 8 | 9 | 2 | 0 | 1 | 2 | 0 | 0 | 0 |
| OTU651 | 5 | 0 | 4 | 0 | 0 | 1 | 0 | 16 | 1 | 0 | 0 | 0 | 0 | 0 | 2 | 0 | 0 | 0 |
| OTU652 | 0 | 0 | 0 | 0 | 0 | 0 | 0 | 1 | 2 | 3 | 0 | 3 | 0 | 0 | 5 | 0 | 0 | 0 |
| OTU653 | 0 | 0 | 0 | 0 | 0 | 0 | 0 | 1 | 9 | 12 | 2 | 1 | 0 | 1 | 11 | 0 | 0 | 1 |
| OTU654 | 0 | 0 | 0 | 0 | 0 | 0 | 0 | 0 | 5 | 3 | 3 | 0 | 0 | 0 | 1 | 0 | 0 | 0 |
| OTU655 | 0 | 2 | 0 | 1 | 2 | 1 | 1 | 0 | 1 | 1 | 4 | 3 | 1 | 0 | 2 | 0 | 0 | 0 |
| OTU656 | 0 | 0 | 0 | 1 | 0 | 0 | 0 | 0 | 6 | 5 | 0 | 0 | 0 | 0 | 4 | 0 | 1 | 0 |
| OTU657 | 0 | 1 | 0 | 0 | 1 | 0 | 0 | 0 | 1 | 2 | 0 | 0 | 0 | 0 | 1 | 0 | 0 | 0 |
| OTU658 | 0 | 0 | 0 | 0 | 0 | 1 | 5 | 11 | 1 | 12 | 3 | 0 | 0 | 1 | 0 | 0 | 0 | 0 |
| OTU659 | 0 | 0 | 0 | 0 | 0 | 0 | 0 | 0 | 3 | 2 | 2 | 0 | 1 | 0 | 4 | 0 | 0 | 0 |
| OTU66 | 2 | 1015 | 160 | 6 | 77 | 26 | 5 | 10 | 10 | 36 | 45 | 22 | 1 | 3 | 14 | 0 | 1 | 1 |
| OTU660 | 0 | 0 | 0 | 0 | 0 | 0 | 1 | 1 | 1 | 1 | 2 | 15 | 0 | 0 | 1 | 0 | 0 | 0 |
| OTU661 | 0 | 0 | 0 | 0 | 0 | 0 | 0 | 0 | 0 | 2 | 17 | 2 | 0 | 0 | 0 | 0 | 0 | 0 |
| OTU662 | 0 | 0 | 0 | 0 | 0 | 1 | 3 | 1 | 1 | 2 | 24 | 1 | 1 | 0 | 0 | 0 | 0 | 0 |
| OTU663 | 0 | 0 | 0 | 0 | 0 | 0 | 0 | 3 | 6 | 1 | 4 | 0 | 0 | 1 | 5 | 0 | 0 | 0 |
| OTU664 | 11 | 5 | 11 | 0 | 2 | 1 | 1 | 0 | 1 | 0 | 0 | 4 | 0 | 2 | 0 | 0 | 0 | 0 |
| OTU665 | 0 | 0 | 0 | 0 | 0 | 0 | 0 | 0 | 0 | 0 | 0 | 0 | 1 | 7 | 0 | 0 | 1 | 6 |
| OTU666 | 1 | 0 | 0 | 0 | 0 | 0 | 0 | 1 | 2 | 1 | 1 | 0 | 1 | 0 | 0 | 4 | 0 | 0 |
| OTU667 | 1 | 1 | 3 | 1 | 1 | 1 | 2 | 6 | 3 | 1 | 2 | 0 | 2 | 5 | 2 | 2 | 2 | 0 |
| OTU668 | 0 | 0 | 0 | 0 | 0 | 0 | 0 | 0 | 0 | 0 | 0 | 0 | 2 | 4 | 0 | 0 | 3 | 6 |
| OTU669 | 0 | 0 | 0 | 0 | 0 | 0 | 0 | 0 | 0 | 0 | 0 | 0 | 0 | 6 | 0 | 0 | 9 | 14 |
| OTU67 | 1 | 157 | 70 | 1 | 176 | 302 | 0 | 1 | 0 | 0 | 0 | 0 | 0 | 0 | 0 | 0 | 1 | 0 |
| OTU670 | 0 | 1 | 1 | 0 | 0 | 0 | 0 | 0 | 1 | 0 | 2 | 0 | 4 | 3 | 0 | 0 | 13 | 8 |
| OTU671 | 0 | 0 | 0 | 0 | 0 | 0 | 0 | 0 | 0 | 0 | 0 | 0 | 1 | 0 | 0 | 0 | 2 | 3 |
| OTU672 | 0 | 0 | 0 | 0 | 0 | 0 | 0 | 0 | 0 | 0 | 0 | 0 | 0 | 0 | 0 | 0 | 19 | 5 |
| OTU673 | 0 | 0 | 0 | 0 | 0 | 0 | 0 | 0 | 0 | 0 | 0 | 1 | 3 | 0 | 0 | 0 | 17 | 11 |
| OTU674 | 0 | 0 | 0 | 0 | 1 | 1 | 0 | 0 | 0 | 0 | 1 | 0 | 0 | 0 | 0 | 0 | 15 | 0 |
| OTU675 | 0 | 0 | 0 | 0 | 0 | 0 | 0 | 0 | 0 | 0 | 0 | 0 | 0 | 0 | 0 | 0 | 5 | 2 |
| OTU676 | 0 | 0 | 0 | 0 | 0 | 0 | 0 | 0 | 0 | 0 | 0 | 0 | 0 | 0 | 0 | 0 | 24 | 0 |
| OTU677 | 0 | 0 | 0 | 0 | 0 | 0 | 1 | 0 | 0 | 0 | 0 | 0 | 1 | 0 | 0 | 1 | 11 | 4 |
| OTU678 | 3 | 0 | 0 | 0 | 0 | 0 | 0 | 0 | 0 | 0 | 0 | 0 | 0 | 0 | 0 | 0 | 41 | 0 |
| OTU679 | 0 | 1 | 0 | 0 | 0 | 0 | 0 | 0 | 0 | 0 | 0 | 0 | 0 | 0 | 0 | 0 | 27 | 0 |
| OTU68 | 0 | 0 | 0 | 0 | 0 | 0 | 2 | 2 | 2 | 2 | 1 | 5 | 52 | 126 | 4 | 38 | 387 | 270 |
| OTU680 | 0 | 0 | 0 | 0 | 0 | 0 | 0 | 0 | 0 | 0 | 0 | 0 | 0 | 0 | 0 | 0 | 52 | 0 |
| OTU681 | 0 | 0 | 0 | 0 | 0 | 0 | 0 | 1 | 0 | 0 | 0 | 0 | 0 | 0 | 0 | 0 | 26 | 0 |
| OTU682 | 0 | 0 | 0 | 0 | 0 | 0 | 0 | 0 | 0 | 0 | 0 | 0 | 2 | 2 | 0 | 2 | 22 | 8 |
| OTU683 | 0 | 1 | 1 | 2 | 0 | 1 | 1 | 0 | 0 | 1 | 0 | 1 | 2 | 1 | 0 | 0 | 23 | 2 |
| OTU684 | 7 | 0 | 0 | 0 | 0 | 0 | 0 | 11 | 2 | 6 | 15 | 0 | 0 | 0 | 0 | 0 | 0 | 0 |
| OTU685 | 2 | 0 | 1 | 0 | 0 | 2 | 0 | 0 | 0 | 0 | 0 | 0 | 0 | 0 | 0 | 0 | 0 | 0 |
| OTU686 | 3 | 3 | 11 | 0 | 0 | 1 | 0 | 0 | 0 | 0 | 0 | 0 | 0 | 0 | 0 | 0 | 0 | 0 |
| OTU687 | 2 | 0 | 0 | 0 | 0 | 0 | 0 | 0 | 0 | 0 | 0 | 0 | 0 | 0 | 0 | 0 | 0 | 0 |
| OTU688 | 2 | 0 | 1 | 0 | 0 | 0 | 0 | 0 | 4 | 0 | 0 | 0 | 0 | 0 | 1 | 0 | 2 | 0 |
| OTU689 | 13 | 0 | 0 | 0 | 0 | 0 | 0 | 0 | 0 | 0 | 0 | 0 | 0 | 0 | 0 | 0 | 0 | 0 |
| OTU69 | 35 | 564 | 56 | 12 | 176 | 135 | 77 | 1 | 101 | 11 | 8 | 3 | 0 | 2 | 170 | 0 | 3 | 1 |
| OTU690 | 1 | 1 | 1 | 0 | 1 | 2 | 1 | 0 | 2 | 4 | 1 | 0 | 1 | 1 | 0 | 0 | 0 | 0 |
| OTU691 | 76 | 112 | 13 | 0 | 0 | 1 | 16 | 3 | 4 | 0 | 3 | 89 | 8 | 18 | 7 | 0 | 0 | 0 |
| OTU692 | 2 | 0 | 0 | 0 | 0 | 0 | 0 | 0 | 0 | 0 | 0 | 0 | 0 | 0 | 0 | 0 | 0 | 0 |
| OTU693 | 1 | 1 | 2 | 0 | 2 | 1 | 0 | 1 | 0 | 0 | 1 | 0 | 1 | 0 | 0 | 0 | 0 | 1 |
| OTU694 | 2 | 1 | 2 | 0 | 3 | 3 | 0 | 0 | 0 | 0 | 0 | 0 | 0 | 0 | 0 | 0 | 0 | 0 |
| OTU695 | 2 | 0 | 0 | 0 | 3 | 8 | 0 | 1 | 0 | 1 | 2 | 2 | 0 | 0 | 0 | 0 | 0 | 0 |
| OTU696 | 1 | 0 | 1 | 0 | 0 | 1 | 4 | 4 | 1 | 4 | 2 | 0 | 0 | 0 | 1 | 0 | 0 | 0 |
| OTU697 | 5 | 3 | 1 | 1 | 3 | 2 | 1 | 6 | 1 | 0 | 1 | 1 | 0 | 0 | 0 | 0 | 0 | 0 |
| OTU698 | 4 | 0 | 4 | 0 | 0 | 0 | 0 | 1 | 0 | 1 | 0 | 0 | 0 | 0 | 1 | 0 | 0 | 0 |
| OTU699 | 4 | 0 | 3 | 3 | 0 | 1 | 0 | 1 | 0 | 0 | 0 | 0 | 0 | 0 | 0 | 0 | 0 | 0 |
| OTU7 | 4083 | 1198 | 3816 | 4193 | 2975 | 524 | 37 | 3 | 10 | 15 | 17 | 23 | 0 | 6 | 7 | 0 | 6 | 6 |
| OTU70 | 1 | 3 | 2 | 1 | 3 | 4 | 108 | 227 | 80 | 211 | 445 | 34 | 1 | 3 | 41 | 0 | 23 | 5 |
| OTU700 | 1 | 1 | 0 | 0 | 0 | 1 | 0 | 2 | 1 | 0 | 1 | 0 | 1 | 2 | 0 | 3 | 1 | 0 |
| OTU701 | 2 | 1 | 0 | 0 | 3 | 2 | 0 | 0 | 0 | 0 | 0 | 0 | 0 | 0 | 0 | 0 | 0 | 0 |
| OTU702 | 0 | 1 | 0 | 0 | 0 | 0 | 0 | 0 | 0 | 0 | 0 | 0 | 0 | 0 | 1 | 0 | 0 | 0 |
| OTU703 | 0 | 1 | 0 | 0 | 0 | 1 | 1 | 0 | 0 | 0 | 2 | 0 | 0 | 0 | 0 | 0 | 1 | 0 |
| OTU704 | 0 | 2 | 0 | 0 | 0 | 0 | 0 | 0 | 0 | 0 | 0 | 0 | 0 | 0 | 0 | 0 | 0 | 0 |
| OTU705 | 0 | 6 | 0 | 0 | 0 | 0 | 0 | 0 | 0 | 0 | 0 | 0 | 0 | 0 | 0 | 0 | 0 | 0 |
| OTU706 | 2 | 3 | 1 | 2 | 4 | 1 | 0 | 0 | 0 | 0 | 0 | 0 | 0 | 0 | 0 | 0 | 0 | 0 |
| OTU707 | 2 | 2 | 0 | 1 | 0 | 1 | 0 | 0 | 0 | 0 | 0 | 0 | 0 | 0 | 0 | 0 | 0 | 0 |
| OTU708 | 3 | 47 | 3 | 47 | 0 | 1 | 0 | 17 | 58 | 1 | 0 | 6 | 2 | 1 | 33 | 0 | 7 | 2 |
| OTU709 | 0 | 2 | 2 | 0 | 0 | 2 | 0 | 0 | 2 | 5 | 2 | 0 | 0 | 1 | 0 | 1 | 1 | 0 |
| OTU71 | 73 | 106 | 126 | 35 | 71 | 299 | 3 | 0 | 0 | 0 | 0 | 0 | 0 | 0 | 0 | 0 | 0 | 1 |
| OTU710 | 0 | 1 | 1 | 2 | 4 | 6 | 2 | 7 | 4 | 2 | 4 | 2 | 0 | 0 | 1 | 2 | 3 | 0 |
| OTU711 | 0 | 1 | 0 | 0 | 0 | 0 | 0 | 0 | 0 | 0 | 0 | 0 | 0 | 0 | 0 | 3 | 0 | 0 |
| OTU712 | 0 | 4 | 1 | 0 | 0 | 0 | 0 | 0 | 0 | 0 | 0 | 2 | 0 | 1 | 0 | 0 | 0 | 0 |
| OTU713 | 4 | 1 | 0 | 1 | 0 | 0 | 0 | 2 | 0 | 4 | 1 | 0 | 1 | 1 | 1 | 0 | 4 | 2 |
| OTU714 | 1 | 2 | 2 | 2 | 1 | 0 | 0 | 2 | 0 | 0 | 1 | 3 | 0 | 0 | 1 | 0 | 0 | 0 |
| OTU715 | 2 | 6 | 3 | 1 | 3 | 6 | 2 | 3 | 1 | 5 | 10 | 6 | 1 | 0 | 5 | 0 | 7 | 2 |
| OTU716 | 3 | 7 | 1 | 3 | 4 | 4 | 0 | 0 | 0 | 0 | 0 | 0 | 0 | 0 | 0 | 0 | 0 | 0 |
| OTU717 | 1 | 4 | 9 | 0 | 0 | 5 | 2 | 1 | 1 | 3 | 5 | 1 | 1 | 1 | 2 | 0 | 0 | 0 |
| OTU718 | 1 | 21 | 1 | 0 | 0 | 11 | 0 | 0 | 1 | 1 | 1 | 0 | 0 | 0 | 0 | 0 | 0 | 0 |
| OTU719 | 0 | 6 | 0 | 0 | 5 | 15 | 0 | 0 | 3 | 0 | 0 | 0 | 0 | 0 | 3 | 0 | 0 | 1 |
| OTU72 | 6 | 4 | 1 | 4 | 3 | 7 | 437 | 129 | 243 | 108 | 301 | 2 | 16 | 24 | 219 | 1 | 5 | 10 |
| OTU720 | 0 | 2 | 1 | 0 | 0 | 1 | 0 | 0 | 0 | 0 | 0 | 0 | 0 | 0 | 0 | 0 | 0 | 0 |
| OTU721 | 0 | 1 | 1 | 1 | 3 | 1 | 0 | 0 | 0 | 0 | 0 | 0 | 0 | 0 | 0 | 0 | 0 | 0 |
| OTU722 | 0 | 2 | 1 | 2 | 4 | 0 | 4 | 0 | 0 | 2 | 1 | 0 | 0 | 1 | 2 | 0 | 1 | 1 |
| OTU723 | 0 | 7 | 0 | 1 | 0 | 0 | 0 | 0 | 0 | 0 | 0 | 0 | 0 | 0 | 0 | 0 | 0 | 0 |
| OTU724 | 0 | 2 | 7 | 7 | 8 | 6 | 0 | 0 | 0 | 0 | 0 | 0 | 0 | 0 | 0 | 0 | 0 | 0 |
| OTU725 | 4 | 0 | 13 | 0 | 0 | 0 | 0 | 0 | 1 | 0 | 0 | 1 | 0 | 0 | 2 | 0 | 0 | 0 |
| OTU726 | 0 | 1 | 12 | 0 | 8 | 1 | 0 | 0 | 0 | 0 | 0 | 0 | 0 | 0 | 0 | 1 | 1 | 1 |
| OTU727 | 1 | 0 | 5 | 3 | 3 | 0 | 1 | 2 | 0 | 3 | 1 | 1 | 0 | 1 | 0 | 0 | 1 | 1 |
| OTU728 | 0 | 0 | 2 | 0 | 0 | 0 | 1 | 1 | 0 | 0 | 0 | 9 | 0 | 3 | 0 | 0 | 0 | 0 |
| OTU729 | 0 | 0 | 3 | 1 | 2 | 2 | 1 | 0 | 0 | 1 | 2 | 0 | 0 | 0 | 0 | 0 | 0 | 0 |
| OTU73 | 6 | 36 | 65 | 4 | 306 | 17 | 2 | 194 | 449 | 4 | 170 | 138 | 1 | 3 | 462 | 0 | 23 | 10 |
| OTU730 | 0 | 0 | 4 | 2 | 14 | 0 | 2 | 0 | 0 | 0 | 0 | 0 | 0 | 0 | 0 | 0 | 0 | 0 |
| OTU731 | 0 | 0 | 14 | 0 | 3 | 0 | 0 | 4 | 5 | 1 | 2 | 0 | 1 | 0 | 2 | 0 | 1 | 0 |
| OTU732 | 5 | 3 | 5 | 3 | 4 | 0 | 0 | 1 | 1 | 1 | 1 | 4 | 1 | 3 | 2 | 0 | 0 | 0 |
| OTU733 | 0 | 0 | 2 | 0 | 0 | 0 | 0 | 0 | 0 | 0 | 0 | 0 | 0 | 0 | 0 | 0 | 0 | 0 |
| OTU734 | 0 | 6 | 7 | 0 | 0 | 0 | 0 | 0 | 1 | 1 | 0 | 0 | 0 | 0 | 0 | 0 | 0 | 0 |
| OTU735 | 2 | 0 | 1 | 4 | 1 | 4 | 4 | 0 | 1 | 0 | 0 | 5 | 1 | 6 | 1 | 0 | 0 | 1 |
| OTU736 | 1 | 1 | 5 | 1 | 0 | 0 | 0 | 0 | 4 | 0 | 0 | 1 | 0 | 0 | 1 | 0 | 1 | 0 |
| OTU737 | 0 | 0 | 3 | 0 | 0 | 3 | 0 | 0 | 0 | 0 | 0 | 0 | 0 | 0 | 0 | 0 | 0 | 0 |
| OTU738 | 24 | 11 | 32 | 42 | 27 | 45 | 2 | 0 | 1 | 1 | 0 | 1 | 0 | 0 | 0 | 0 | 0 | 0 |
| OTU739 | 0 | 0 | 1 | 0 | 1 | 0 | 0 | 0 | 0 | 0 | 0 | 0 | 0 | 0 | 1 | 0 | 0 | 0 |
| OTU74 | 65 | 20 | 638 | 0 | 3 | 25 | 3 | 0 | 0 | 0 | 0 | 0 | 0 | 0 | 0 | 0 | 0 | 0 |
| OTU740 | 1 | 0 | 3 | 3 | 0 | 2 | 0 | 0 | 0 | 0 | 0 | 0 | 0 | 0 | 0 | 0 | 0 | 0 |
| OTU741 | 0 | 0 | 1 | 0 | 2 | 0 | 0 | 0 | 0 | 0 | 0 | 0 | 0 | 0 | 0 | 0 | 0 | 0 |
| OTU742 | 3 | 2 | 8 | 7 | 3 | 8 | 0 | 0 | 0 | 0 | 0 | 0 | 0 | 0 | 0 | 0 | 0 | 0 |
| OTU743 | 1 | 0 | 2 | 0 | 0 | 0 | 1 | 5 | 1 | 1 | 8 | 3 | 1 | 2 | 2 | 0 | 0 | 1 |
| OTU744 | 1 | 0 | 7 | 0 | 0 | 2 | 0 | 0 | 0 | 0 | 0 | 0 | 0 | 0 | 0 | 0 | 0 | 0 |
| OTU745 | 0 | 0 | 1 | 4 | 1 | 1 | 0 | 0 | 0 | 1 | 0 | 1 | 0 | 0 | 0 | 0 | 0 | 0 |
| OTU746 | 7 | 2 | 6 | 6 | 17 | 16 | 3 | 38 | 13 | 23 | 29 | 17 | 1 | 1 | 11 | 0 | 0 | 0 |
| OTU747 | 0 | 0 | 1 | 7 | 2 | 1 | 0 | 0 | 0 | 0 | 0 | 0 | 0 | 0 | 0 | 0 | 0 | 0 |
| OTU748 | 15 | 29 | 66 | 58 | 51 | 41 | 2 | 0 | 0 | 17 | 18 | 0 | 0 | 1 | 0 | 0 | 0 | 1 |
| OTU749 | 3 | 1 | 4 | 3 | 0 | 8 | 0 | 0 | 0 | 0 | 0 | 0 | 0 | 0 | 0 | 0 | 0 | 0 |
| OTU75 | 7 | 2 | 11 | 0 | 1 | 2 | 4 | 0 | 1 | 1 | 2 | 2 | 2 | 4 | 2 | 22 | 639 | 300 |
| OTU750 | 1 | 2 | 6 | 6 | 3 | 7 | 0 | 0 | 0 | 0 | 0 | 0 | 0 | 0 | 0 | 0 | 0 | 0 |
| OTU751 | 0 | 0 | 0 | 1 | 0 | 0 | 0 | 1 | 0 | 2 | 0 | 2 | 0 | 1 | 0 | 0 | 0 | 0 |
| OTU752 | 1 | 5 | 5 | 1 | 0 | 0 | 1 | 0 | 0 | 1 | 0 | 0 | 0 | 0 | 1 | 0 | 0 | 0 |
| OTU753 | 0 | 0 | 1 | 0 | 8 | 12 | 0 | 0 | 0 | 0 | 0 | 0 | 0 | 0 | 0 | 0 | 0 | 0 |
| OTU754 | 1 | 0 | 0 | 0 | 3 | 0 | 1 | 0 | 0 | 0 | 0 | 0 | 0 | 0 | 0 | 0 | 0 | 0 |
| OTU755 | 0 | 0 | 0 | 2 | 3 | 16 | 0 | 1 | 0 | 1 | 2 | 0 | 0 | 0 | 0 | 0 | 0 | 0 |
| OTU756 | 0 | 1 | 2 | 0 | 2 | 2 | 5 | 0 | 1 | 0 | 0 | 3 | 0 | 0 | 1 | 0 | 6 | 1 |
| OTU757 | 0 | 0 | 0 | 0 | 13 | 2 | 0 | 0 | 0 | 0 | 0 | 0 | 0 | 0 | 0 | 0 | 0 | 0 |
| OTU758 | 0 | 0 | 2 | 0 | 1 | 4 | 0 | 0 | 0 | 0 | 0 | 0 | 0 | 0 | 0 | 0 | 0 | 0 |
| OTU759 | 0 | 0 | 4 | 1 | 11 | 2 | 0 | 0 | 0 | 0 | 0 | 0 | 0 | 0 | 0 | 0 | 0 | 0 |
| OTU76 | 0 | 0 | 0 | 0 | 0 | 0 | 141 | 138 | 84 | 23 | 121 | 148 | 18 | 80 | 66 | 1 | 3 | 4 |
| OTU760 | 1 | 0 | 0 | 0 | 4 | 1 | 0 | 0 | 0 | 0 | 0 | 0 | 0 | 0 | 0 | 0 | 0 | 0 |
| OTU761 | 0 | 0 | 2 | 0 | 1 | 1 | 3 | 4 | 0 | 2 | 3 | 3 | 1 | 1 | 2 | 3 | 4 | 1 |
| OTU762 | 0 | 0 | 0 | 0 | 2 | 0 | 0 | 0 | 0 | 2 | 0 | 0 | 0 | 0 | 0 | 0 | 0 | 0 |
| OTU763 | 0 | 0 | 1 | 1 | 2 | 0 | 0 | 0 | 3 | 0 | 2 | 2 | 0 | 0 | 0 | 0 | 1 | 1 |
| OTU764 | 2 | 0 | 0 | 0 | 3 | 6 | 0 | 0 | 0 | 0 | 0 | 0 | 0 | 0 | 0 | 0 | 0 | 0 |
| OTU765 | 1 | 0 | 1 | 0 | 24 | 0 | 0 | 0 | 0 | 0 | 0 | 0 | 0 | 0 | 0 | 0 | 0 | 0 |
| OTU766 | 4 | 21 | 0 | 12 | 17 | 1 | 0 | 32 | 6 | 29 | 24 | 6 | 2 | 6 | 6 | 0 | 0 | 3 |
| OTU767 | 2 | 2 | 1 | 1 | 2 | 2 | 2 | 3 | 1 | 1 | 2 | 2 | 8 | 2 | 0 | 1 | 7 | 2 |
| OTU768 | 1 | 0 | 0 | 1 | 1 | 0 | 1 | 1 | 1 | 1 | 0 | 0 | 0 | 0 | 2 | 0 | 1 | 0 |
| OTU769 | 0 | 0 | 0 | 0 | 1 | 0 | 0 | 0 | 0 | 0 | 0 | 0 | 0 | 1 | 0 | 0 | 0 | 1 |
| OTU77 | 150 | 198 | 263 | 30 | 284 | 79 | 9 | 3 | 2 | 1 | 3 | 17 | 1 | 2 | 10 | 0 | 1 | 1 |
| OTU770 | 1 | 0 | 0 | 0 | 1 | 1 | 1 | 0 | 0 | 6 | 4 | 1 | 0 | 0 | 3 | 0 | 1 | 0 |
| OTU771 | 0 | 0 | 1 | 0 | 3 | 2 | 0 | 0 | 0 | 0 | 0 | 0 | 0 | 0 | 0 | 0 | 0 | 0 |
| OTU772 | 0 | 0 | 0 | 0 | 1 | 0 | 0 | 0 | 0 | 0 | 0 | 0 | 0 | 2 | 0 | 0 | 0 | 0 |
| OTU773 | 0 | 1 | 1 | 0 | 1 | 2 | 0 | 0 | 0 | 0 | 1 | 0 | 1 | 1 | 1 | 0 | 1 | 0 |
| OTU774 | 1 | 1 | 1 | 1 | 1 | 0 | 0 | 1 | 2 | 0 | 0 | 0 | 2 | 0 | 1 | 0 | 0 | 0 |
| OTU775 | 1 | 1 | 0 | 0 | 0 | 5 | 0 | 0 | 0 | 0 | 0 | 0 | 0 | 0 | 0 | 0 | 0 | 0 |
| OTU776 | 1 | 0 | 0 | 0 | 0 | 6 | 0 | 3 | 0 | 0 | 1 | 0 | 0 | 0 | 0 | 0 | 0 | 0 |
| OTU777 | 1 | 0 | 0 | 0 | 0 | 2 | 0 | 0 | 0 | 0 | 0 | 0 | 0 | 0 | 0 | 0 | 0 | 0 |
| OTU778 | 0 | 1 | 1 | 0 | 2 | 3 | 0 | 0 | 0 | 0 | 0 | 0 | 0 | 0 | 0 | 0 | 0 | 0 |
| OTU779 | 0 | 0 | 0 | 0 | 0 | 10 | 0 | 3 | 1 | 1 | 2 | 0 | 0 | 0 | 0 | 0 | 1 | 0 |
| OTU78 | 92 | 296 | 277 | 121 | 69 | 173 | 5 | 0 | 1 | 10 | 4 | 2 | 0 | 1 | 2 | 0 | 0 | 2 |
| OTU780 | 0 | 3 | 0 | 0 | 2 | 19 | 0 | 0 | 0 | 0 | 0 | 0 | 0 | 0 | 0 | 0 | 0 | 0 |
| OTU781 | 0 | 0 | 1 | 1 | 0 | 8 | 0 | 0 | 2 | 1 | 0 | 0 | 0 | 0 | 0 | 0 | 0 | 0 |
| OTU782 | 5 | 1 | 3 | 1 | 17 | 8 | 2 | 24 | 12 | 14 | 15 | 7 | 0 | 1 | 7 | 0 | 0 | 0 |
| OTU783 | 0 | 3 | 0 | 1 | 0 | 1 | 0 | 0 | 1 | 2 | 0 | 0 | 0 | 0 | 0 | 0 | 0 | 0 |
| OTU784 | 0 | 1 | 0 | 0 | 0 | 1 | 0 | 0 | 1 | 0 | 2 | 2 | 0 | 1 | 0 | 0 | 0 | 0 |
| OTU785 | 0 | 0 | 1 | 2 | 0 | 2 | 1 | 0 | 0 | 1 | 4 | 1 | 0 | 1 | 1 | 0 | 0 | 0 |
| OTU786 | 2 | 0 | 0 | 0 | 1 | 2 | 0 | 1 | 2 | 0 | 3 | 1 | 0 | 0 | 1 | 0 | 2 | 0 |
| OTU787 | 0 | 0 | 0 | 0 | 0 | 1 | 0 | 0 | 0 | 2 | 2 | 1 | 0 | 0 | 1 | 0 | 0 | 0 |
| OTU788 | 0 | 0 | 0 | 0 | 0 | 1 | 0 | 0 | 0 | 1 | 0 | 0 | 0 | 0 | 0 | 0 | 0 | 0 |
| OTU789 | 0 | 0 | 1 | 1 | 1 | 4 | 3 | 3 | 0 | 1 | 2 | 0 | 2 | 0 | 0 | 0 | 0 | 0 |
| OTU79 | 112 | 72 | 293 | 163 | 553 | 161 | 31 | 17 | 18 | 53 | 12 | 18 | 8 | 29 | 11 | 0 | 2 | 1 |
| OTU790 | 0 | 0 | 0 | 0 | 0 | 0 | 7 | 2 | 3 | 2 | 1 | 2 | 0 | 0 | 8 | 0 | 0 | 0 |
| OTU791 | 1 | 0 | 0 | 0 | 0 | 0 | 86 | 0 | 0 | 44 | 13 | 0 | 0 | 0 | 0 | 0 | 2 | 3 |
| OTU792 | 0 | 0 | 0 | 0 | 0 | 1 | 3 | 1 | 0 | 0 | 0 | 1 | 0 | 0 | 0 | 0 | 0 | 1 |
| OTU793 | 0 | 0 | 0 | 0 | 1 | 0 | 1 | 0 | 0 | 0 | 0 | 2 | 0 | 0 | 0 | 0 | 0 | 0 |
| OTU794 | 6 | 0 | 1 | 0 | 0 | 0 | 4 | 8 | 1 | 0 | 0 | 2 | 0 | 1 | 0 | 0 | 0 | 0 |
| OTU795 | 2 | 1 | 2 | 2 | 1 | 2 | 5 | 5 | 3 | 7 | 3 | 2 | 11 | 6 | 1 | 11 | 4 | 5 |
| OTU796 | 0 | 0 | 0 | 0 | 0 | 0 | 2 | 4 | 0 | 9 | 8 | 3 | 1 | 2 | 1 | 0 | 0 | 0 |
| OTU797 | 0 | 0 | 2 | 0 | 0 | 0 | 3 | 0 | 3 | 3 | 1 | 0 | 0 | 0 | 2 | 0 | 2 | 0 |
| OTU798 | 0 | 0 | 0 | 0 | 0 | 0 | 1 | 2 | 1 | 3 | 0 | 1 | 0 | 2 | 2 | 0 | 0 | 0 |
| OTU799 | 0 | 0 | 0 | 0 | 0 | 0 | 4 | 1 | 0 | 0 | 0 | 0 | 0 | 0 | 0 | 0 | 0 | 0 |
| OTU8 | 1737 | 42 | 416 | 325 | 379 | 66 | 6336 | 313 | 109 | 1460 | 770 | 2766 | 70 | 358 | 255 | 0 | 9 | 43 |
| OTU80 | 0 | 26 | 3 | 1 | 0 | 719 | 2 | 19 | 18 | 50 | 24 | 51 | 2 | 8 | 11 | 0 | 2 | 3 |
| OTU800 | 0 | 0 | 0 | 0 | 0 | 0 | 17 | 0 | 0 | 0 | 5 | 0 | 0 | 0 | 1 | 0 | 0 | 0 |
| OTU801 | 2 | 1 | 1 | 0 | 1 | 2 | 6 | 3 | 2 | 6 | 9 | 3 | 0 | 1 | 5 | 0 | 0 | 0 |
| OTU802 | 0 | 0 | 0 | 0 | 0 | 0 | 1 | 0 | 1 | 0 | 0 | 0 | 0 | 0 | 0 | 0 | 0 | 0 |
| OTU803 | 0 | 0 | 0 | 0 | 0 | 0 | 4 | 3 | 1 | 9 | 7 | 2 | 3 | 0 | 8 | 0 | 0 | 0 |
| OTU804 | 0 | 0 | 0 | 0 | 0 | 0 | 5 | 0 | 0 | 1 | 1 | 2 | 0 | 0 | 1 | 0 | 3 | 0 |
| OTU805 | 0 | 0 | 0 | 0 | 0 | 0 | 3 | 0 | 1 | 0 | 1 | 6 | 0 | 1 | 0 | 0 | 0 | 0 |
| OTU806 | 0 | 0 | 0 | 0 | 0 | 0 | 0 | 1 | 1 | 1 | 1 | 0 | 0 | 0 | 0 | 0 | 6 | 0 |
| OTU807 | 0 | 0 | 0 | 1 | 1 | 0 | 2 | 1 | 1 | 1 | 1 | 1 | 1 | 0 | 2 | 1 | 0 | 0 |
| OTU808 | 0 | 0 | 0 | 0 | 0 | 0 | 0 | 4 | 1 | 1 | 0 | 0 | 0 | 0 | 0 | 0 | 0 | 0 |
| OTU809 | 0 | 0 | 0 | 0 | 0 | 0 | 0 | 6 | 2 | 3 | 0 | 2 | 0 | 0 | 0 | 0 | 0 | 0 |
| OTU81 | 70 | 223 | 83 | 5 | 1 | 282 | 60 | 10 | 25 | 4 | 6 | 191 | 9 | 69 | 38 | 1 | 6 | 0 |
| OTU810 | 0 | 1 | 0 | 0 | 0 | 0 | 0 | 2 | 0 | 0 | 0 | 1 | 1 | 0 | 0 | 1 | 0 | 0 |
| OTU811 | 0 | 0 | 0 | 0 | 0 | 0 | 0 | 1 | 0 | 0 | 0 | 2 | 0 | 0 | 0 | 0 | 0 | 0 |
| OTU812 | 0 | 0 | 0 | 0 | 0 | 0 | 0 | 2 | 0 | 0 | 0 | 0 | 0 | 0 | 0 | 0 | 0 | 0 |
| OTU813 | 0 | 0 | 0 | 0 | 0 | 0 | 0 | 1 | 2 | 5 | 1 | 2 | 0 | 0 | 0 | 0 | 0 | 0 |
| OTU814 | 0 | 1 | 2 | 0 | 0 | 0 | 1 | 8 | 0 | 0 | 15 | 1 | 0 | 0 | 2 | 0 | 1 | 1 |
| OTU815 | 0 | 0 | 0 | 0 | 0 | 0 | 0 | 1 | 1 | 1 | 1 | 2 | 0 | 0 | 1 | 0 | 1 | 0 |
| OTU816 | 0 | 0 | 0 | 0 | 0 | 0 | 2 | 7 | 1 | 3 | 0 | 0 | 0 | 1 | 2 | 0 | 0 | 0 |
| OTU817 | 0 | 0 | 0 | 0 | 0 | 0 | 0 | 2 | 0 | 0 | 0 | 0 | 0 | 0 | 0 | 0 | 0 | 0 |
| OTU818 | 0 | 0 | 0 | 0 | 0 | 0 | 0 | 3 | 0 | 0 | 14 | 1 | 1 | 0 | 0 | 0 | 0 | 0 |
| OTU819 | 0 | 0 | 0 | 0 | 0 | 0 | 0 | 3 | 6 | 2 | 0 | 1 | 0 | 0 | 9 | 0 | 0 | 0 |
| OTU82 | 117 | 80 | 183 | 0 | 41 | 104 | 26 | 3 | 1 | 0 | 2 | 19 | 6 | 14 | 0 | 0 | 1 | 1 |
| OTU820 | 0 | 0 | 0 | 0 | 0 | 1 | 0 | 1 | 0 | 1 | 1 | 1 | 0 | 0 | 0 | 0 | 2 | 0 |
| OTU821 | 0 | 0 | 0 | 0 | 0 | 0 | 0 | 3 | 2 | 0 | 1 | 0 | 0 | 0 | 0 | 0 | 0 | 0 |
| OTU822 | 0 | 0 | 0 | 0 | 0 | 1 | 1 | 2 | 1 | 3 | 4 | 1 | 0 | 2 | 3 | 0 | 0 | 0 |
| OTU823 | 2 | 1 | 3 | 0 | 0 | 6 | 20 | 3 | 3 | 4 | 3 | 7 | 1 | 2 | 3 | 0 | 1 | 0 |
| OTU824 | 0 | 1 | 1 | 0 | 1 | 0 | 1 | 2 | 0 | 1 | 1 | 2 | 0 | 0 | 0 | 0 | 0 | 0 |
| OTU825 | 0 | 0 | 0 | 0 | 0 | 0 | 0 | 4 | 0 | 2 | 3 | 5 | 0 | 0 | 1 | 0 | 1 | 0 |
| OTU826 | 1 | 0 | 0 | 0 | 0 | 0 | 0 | 1 | 0 | 0 | 1 | 0 | 2 | 0 | 1 | 0 | 1 | 1 |
| OTU827 | 1 | 0 | 9 | 3 | 0 | 4 | 12 | 136 | 38 | 42 | 82 | 58 | 2 | 5 | 20 | 0 | 0 | 0 |
| OTU828 | 0 | 2 | 0 | 0 | 0 | 0 | 1 | 3 | 9 | 0 | 0 | 1 | 0 | 0 | 3 | 0 | 2 | 0 |
| OTU829 | 1 | 3 | 6 | 2 | 5 | 46 | 7 | 95 | 20 | 105 | 72 | 65 | 1 | 2 | 20 | 0 | 1 | 2 |
| OTU83 | 14 | 10 | 3 | 13 | 11 | 14 | 474 | 83 | 159 | 128 | 608 | 382 | 18 | 83 | 70 | 0 | 1 | 2 |
| OTU830 | 2 | 0 | 1 | 0 | 0 | 0 | 0 | 3 | 3 | 0 | 0 | 9 | 0 | 0 | 4 | 0 | 0 | 0 |
| OTU831 | 0 | 0 | 0 | 0 | 0 | 2 | 0 | 4 | 2 | 9 | 9 | 1 | 0 | 0 | 1 | 0 | 1 | 0 |
| OTU832 | 0 | 0 | 0 | 0 | 0 | 0 | 0 | 0 | 1 | 1 | 0 | 1 | 0 | 0 | 0 | 0 | 0 | 0 |
| OTU833 | 0 | 1 | 1 | 2 | 0 | 0 | 0 | 0 | 4 | 1 | 0 | 2 | 0 | 0 | 0 | 0 | 0 | 0 |
| OTU834 | 0 | 0 | 2 | 0 | 0 | 0 | 2 | 0 | 27 | 1 | 0 | 20 | 0 | 2 | 20 | 0 | 0 | 0 |
| OTU835 | 0 | 0 | 0 | 0 | 0 | 1 | 0 | 0 | 5 | 1 | 0 | 0 | 0 | 1 | 3 | 0 | 0 | 0 |
| OTU836 | 0 | 0 | 0 | 0 | 0 | 0 | 0 | 0 | 9 | 4 | 0 | 0 | 0 | 0 | 2 | 0 | 0 | 0 |
| OTU837 | 0 | 0 | 0 | 0 | 1 | 3 | 0 | 1 | 2 | 2 | 0 | 1 | 1 | 0 | 1 | 0 | 1 | 0 |
| OTU838 | 3 | 3 | 1 | 2 | 0 | 2 | 4 | 20 | 77 | 109 | 121 | 46 | 2 | 9 | 87 | 1 | 7 | 5 |
| OTU839 | 0 | 1 | 1 | 1 | 1 | 2 | 0 | 20 | 14 | 0 | 3 | 6 | 1 | 0 | 22 | 0 | 0 | 0 |
| OTU84 | 12 | 3 | 30 | 0 | 4 | 4 | 20 | 48 | 140 | 38 | 307 | 132 | 22 | 70 | 153 | 0 | 8 | 3 |
| OTU840 | 1 | 0 | 1 | 1 | 0 | 1 | 1 | 1 | 3 | 0 | 2 | 2 | 0 | 1 | 0 | 0 | 2 | 0 |
| OTU841 | 0 | 0 | 0 | 0 | 0 | 0 | 1 | 0 | 6 | 0 | 0 | 2 | 1 | 1 | 3 | 1 | 0 | 1 |
| OTU842 | 1 | 0 | 0 | 1 | 1 | 0 | 0 | 3 | 1 | 0 | 0 | 0 | 0 | 5 | 1 | 1 | 2 | 2 |
| OTU843 | 1 | 2 | 2 | 0 | 3 | 5 | 2 | 3 | 2 | 5 | 2 | 3 | 1 | 1 | 2 | 1 | 1 | 0 |
| OTU844 | 0 | 0 | 0 | 1 | 1 | 0 | 0 | 10 | 4 | 11 | 1 | 0 | 0 | 0 | 3 | 0 | 1 | 0 |
| OTU845 | 0 | 0 | 0 | 0 | 0 | 0 | 0 | 1 | 2 | 0 | 0 | 1 | 0 | 0 | 0 | 0 | 1 | 0 |
| OTU846 | 0 | 0 | 0 | 0 | 0 | 0 | 0 | 4 | 7 | 3 | 2 | 1 | 0 | 1 | 3 | 0 | 0 | 0 |
| OTU847 | 0 | 0 | 0 | 2 | 0 | 0 | 1 | 0 | 7 | 0 | 0 | 0 | 0 | 0 | 6 | 0 | 0 | 0 |
| OTU848 | 1 | 0 | 0 | 0 | 1 | 2 | 0 | 0 | 3 | 2 | 1 | 0 | 0 | 0 | 2 | 0 | 0 | 0 |
| OTU849 | 0 | 0 | 0 | 0 | 0 | 0 | 4 | 0 | 0 | 4 | 2 | 0 | 0 | 0 | 0 | 0 | 0 | 0 |
| OTU85 | 0 | 2 | 6 | 0 | 1 | 8 | 66 | 263 | 75 | 35 | 11 | 202 | 5 | 7 | 54 | 0 | 5 | 4 |
| OTU850 | 3 | 4 | 2 | 10 | 4 | 12 | 34 | 22 | 43 | 135 | 12 | 6 | 1 | 1 | 18 | 0 | 0 | 1 |
| OTU851 | 0 | 0 | 0 | 0 | 0 | 0 | 0 | 2 | 1 | 3 | 0 | 3 | 0 | 0 | 0 | 0 | 0 | 0 |
| OTU852 | 1 | 0 | 3 | 0 | 2 | 3 | 73 | 69 | 52 | 88 | 54 | 45 | 11 | 26 | 18 | 0 | 7 | 4 |
| OTU853 | 0 | 0 | 0 | 0 | 0 | 0 | 0 | 2 | 0 | 1 | 1 | 0 | 0 | 1 | 0 | 0 | 0 | 0 |
| OTU854 | 0 | 0 | 0 | 0 | 0 | 0 | 0 | 4 | 0 | 9 | 1 | 0 | 0 | 0 | 0 | 0 | 0 | 0 |
| OTU855 | 0 | 0 | 0 | 0 | 2 | 2 | 1 | 2 | 0 | 1 | 1 | 1 | 0 | 0 | 1 | 0 | 0 | 0 |
| OTU856 | 0 | 0 | 0 | 0 | 1 | 0 | 8 | 38 | 3 | 42 | 8 | 1 | 0 | 0 | 2 | 0 | 3 | 1 |
| OTU857 | 0 | 0 | 0 | 0 | 0 | 0 | 0 | 0 | 0 | 2 | 1 | 2 | 1 | 0 | 1 | 0 | 0 | 0 |
| OTU858 | 0 | 0 | 0 | 0 | 0 | 0 | 1 | 3 | 2 | 1 | 3 | 2 | 0 | 0 | 2 | 0 | 0 | 0 |
| OTU859 | 0 | 0 | 0 | 0 | 0 | 0 | 0 | 0 | 0 | 1 | 0 | 1 | 0 | 0 | 0 | 0 | 0 | 0 |
| OTU86 | 0 | 1 | 0 | 0 | 1 | 0 | 17 | 87 | 187 | 37 | 45 | 356 | 16 | 18 | 138 | 0 | 6 | 5 |
| OTU860 | 0 | 0 | 1 | 0 | 0 | 0 | 1 | 2 | 2 | 3 | 1 | 0 | 0 | 2 | 0 | 0 | 0 | 1 |
| OTU861 | 1 | 2 | 3 | 0 | 8 | 11 | 1 | 4 | 8 | 18 | 4 | 6 | 0 | 11 | 2 | 0 | 0 | 3 |
| OTU862 | 0 | 0 | 1 | 0 | 0 | 0 | 1 | 0 | 2 | 3 | 0 | 2 | 0 | 1 | 2 | 0 | 0 | 0 |
| OTU863 | 0 | 0 | 0 | 0 | 0 | 0 | 0 | 0 | 0 | 1 | 2 | 1 | 0 | 0 | 1 | 0 | 0 | 0 |
| OTU864 | 0 | 0 | 0 | 0 | 0 | 0 | 1 | 1 | 1 | 1 | 3 | 2 | 0 | 2 | 0 | 0 | 0 | 0 |
| OTU865 | 0 | 0 | 8 | 0 | 0 | 25 | 3 | 6 | 1 | 34 | 104 | 2 | 0 | 0 | 5 | 0 | 1 | 1 |
| OTU866 | 0 | 0 | 0 | 0 | 0 | 0 | 0 | 0 | 0 | 1 | 2 | 0 | 0 | 0 | 4 | 0 | 0 | 0 |
| OTU867 | 0 | 0 | 0 | 0 | 1 | 1 | 1 | 0 | 3 | 0 | 8 | 1 | 0 | 2 | 0 | 0 | 0 | 1 |
| OTU868 | 0 | 0 | 0 | 0 | 0 | 0 | 0 | 0 | 0 | 0 | 1 | 3 | 1 | 1 | 0 | 0 | 0 | 0 |
| OTU869 | 0 | 0 | 0 | 0 | 1 | 1 | 0 | 1 | 0 | 0 | 1 | 0 | 2 | 0 | 0 | 0 | 0 | 1 |
| OTU87 | 7 | 3 | 8 | 11 | 2 | 11 | 1 | 19 | 2 | 2 | 8 | 359 | 32 | 134 | 0 | 0 | 0 | 0 |
| OTU870 | 0 | 0 | 1 | 1 | 0 | 0 | 1 | 0 | 1 | 0 | 2 | 3 | 1 | 1 | 1 | 0 | 0 | 0 |
| OTU871 | 1 | 0 | 0 | 1 | 1 | 0 | 0 | 0 | 0 | 0 | 0 | 2 | 0 | 0 | 1 | 0 | 0 | 2 |
| OTU872 | 0 | 0 | 0 | 0 | 0 | 0 | 2 | 0 | 1 | 0 | 1 | 4 | 0 | 0 | 0 | 0 | 0 | 0 |
| OTU873 | 0 | 1 | 0 | 0 | 0 | 1 | 1 | 1 | 1 | 0 | 0 | 1 | 0 | 1 | 1 | 1 | 0 | 0 |
| OTU874 | 0 | 1 | 2 | 0 | 2 | 0 | 0 | 1 | 0 | 0 | 1 | 3 | 0 | 2 | 1 | 0 | 0 | 0 |
| OTU875 | 0 | 0 | 0 | 0 | 0 | 0 | 0 | 0 | 0 | 0 | 0 | 4 | 0 | 0 | 1 | 0 | 0 | 0 |
| OTU876 | 0 | 0 | 0 | 0 | 0 | 0 | 0 | 3 | 5 | 0 | 0 | 1 | 1 | 2 | 4 | 0 | 1 | 0 |
| OTU877 | 1 | 0 | 1 | 0 | 0 | 1 | 0 | 0 | 0 | 2 | 1 | 3 | 1 | 1 | 0 | 1 | 0 | 0 |
| OTU878 | 0 | 1 | 0 | 0 | 0 | 0 | 1 | 0 | 0 | 1 | 0 | 1 | 2 | 0 | 0 | 0 | 0 | 0 |
| OTU879 | 0 | 0 | 0 | 0 | 0 | 0 | 0 | 0 | 0 | 0 | 0 | 0 | 1 | 0 | 1 | 0 | 0 | 0 |
| OTU88 | 103 | 24 | 39 | 302 | 127 | 4 | 0 | 0 | 0 | 0 | 0 | 0 | 0 | 0 | 0 | 0 | 0 | 1 |
| OTU880 | 0 | 0 | 0 | 0 | 0 | 0 | 0 | 0 | 0 | 0 | 0 | 0 | 2 | 1 | 0 | 0 | 3 | 1 |
| OTU881 | 0 | 0 | 0 | 0 | 0 | 0 | 0 | 0 | 0 | 0 | 0 | 0 | 1 | 0 | 0 | 0 | 2 | 2 |
| OTU882 | 0 | 0 | 0 | 0 | 0 | 0 | 0 | 0 | 0 | 0 | 0 | 0 | 0 | 1 | 0 | 0 | 18 | 5 |
| OTU883 | 0 | 0 | 0 | 0 | 0 | 0 | 0 | 0 | 0 | 0 | 0 | 0 | 0 | 1 | 0 | 0 | 5 | 3 |
| OTU884 | 1 | 0 | 0 | 0 | 0 | 0 | 0 | 0 | 1 | 0 | 0 | 0 | 1 | 5 | 0 | 0 | 11 | 7 |
| OTU885 | 0 | 0 | 0 | 0 | 0 | 0 | 0 | 0 | 0 | 0 | 1 | 0 | 0 | 6 | 0 | 0 | 9 | 3 |
| OTU886 | 1 | 0 | 1 | 0 | 1 | 0 | 0 | 0 | 2 | 1 | 1 | 0 | 1 | 2 | 0 | 0 | 5 | 0 |
| OTU887 | 0 | 0 | 0 | 0 | 0 | 0 | 0 | 0 | 0 | 0 | 0 | 0 | 0 | 0 | 1 | 0 | 3 | 0 |
| OTU888 | 0 | 1 | 0 | 0 | 0 | 0 | 0 | 0 | 0 | 0 | 0 | 0 | 0 | 0 | 0 | 1 | 0 | 2 |
| OTU889 | 0 | 0 | 0 | 0 | 0 | 0 | 0 | 0 | 0 | 0 | 0 | 0 | 0 | 0 | 0 | 1 | 1 | 0 |
| OTU89 | 7 | 5 | 11 | 14 | 49 | 63 | 5 | 55 | 12 | 302 | 297 | 15 | 1 | 7 | 9 | 0 | 2 | 1 |
| OTU890 | 0 | 0 | 0 | 0 | 0 | 0 | 0 | 0 | 0 | 0 | 0 | 0 | 0 | 1 | 0 | 0 | 6 | 0 |
| OTU891 | 0 | 0 | 0 | 0 | 0 | 0 | 0 | 0 | 0 | 0 | 0 | 0 | 0 | 0 | 0 | 0 | 20 | 0 |
| OTU892 | 0 | 0 | 0 | 0 | 0 | 0 | 0 | 0 | 0 | 0 | 0 | 0 | 0 | 0 | 1 | 0 | 40 | 0 |
| OTU893 | 0 | 0 | 0 | 0 | 0 | 0 | 0 | 0 | 1 | 0 | 0 | 0 | 0 | 0 | 0 | 0 | 9 | 0 |
| OTU894 | 0 | 0 | 0 | 0 | 0 | 0 | 0 | 0 | 0 | 0 | 0 | 0 | 0 | 0 | 0 | 0 | 3 | 0 |
| OTU895 | 0 | 0 | 0 | 0 | 0 | 0 | 0 | 0 | 0 | 0 | 0 | 0 | 0 | 0 | 0 | 0 | 7 | 0 |
| OTU896 | 0 | 0 | 0 | 0 | 0 | 0 | 0 | 0 | 0 | 0 | 0 | 0 | 0 | 0 | 0 | 0 | 13 | 0 |
| OTU897 | 0 | 0 | 0 | 0 | 0 | 0 | 0 | 0 | 0 | 0 | 0 | 0 | 4 | 0 | 1 | 0 | 7 | 4 |
| OTU898 | 0 | 0 | 0 | 0 | 0 | 0 | 0 | 0 | 0 | 0 | 0 | 0 | 0 | 0 | 0 | 0 | 5 | 1 |
| OTU899 | 0 | 0 | 0 | 0 | 0 | 0 | 0 | 0 | 0 | 0 | 0 | 0 | 0 | 0 | 0 | 0 | 7 | 0 |
| OTU9 | 234 | 271 | 14 | 17 | 26 | 800 | 9 | 1991 | 610 | 1599 | 3160 | 1098 | 73 | 313 | 372 | 0 | 64 | 35 |
| OTU90 | 1 | 1 | 1 | 3 | 2 | 8 | 8 | 28 | 103 | 172 | 24 | 556 | 21 | 66 | 80 | 0 | 11 | 4 |
| OTU900 | 0 | 0 | 0 | 0 | 0 | 0 | 0 | 0 | 1 | 0 | 0 | 0 | 2 | 2 | 0 | 0 | 6 | 1 |
| OTU901 | 0 | 0 | 0 | 0 | 0 | 0 | 0 | 0 | 0 | 0 | 0 | 0 | 0 | 0 | 0 | 0 | 7 | 0 |
| OTU902 | 0 | 0 | 0 | 0 | 0 | 0 | 0 | 0 | 0 | 0 | 0 | 0 | 0 | 0 | 0 | 0 | 11 | 1 |
| OTU903 | 0 | 0 | 0 | 0 | 0 | 0 | 0 | 0 | 0 | 0 | 0 | 0 | 0 | 0 | 0 | 0 | 2 | 0 |
| OTU904 | 0 | 0 | 0 | 0 | 0 | 0 | 0 | 0 | 0 | 0 | 0 | 0 | 0 | 0 | 0 | 0 | 9 | 0 |
| OTU905 | 1 | 0 | 0 | 0 | 1 | 0 | 0 | 0 | 0 | 0 | 1 | 0 | 2 | 3 | 0 | 0 | 12 | 0 |
| OTU906 | 0 | 0 | 0 | 0 | 0 | 0 | 0 | 0 | 0 | 0 | 0 | 0 | 0 | 0 | 0 | 0 | 23 | 0 |
| OTU907 | 0 | 0 | 0 | 0 | 0 | 0 | 0 | 1 | 0 | 0 | 0 | 0 | 0 | 0 | 0 | 0 | 15 | 0 |
| OTU908 | 0 | 0 | 0 | 0 | 0 | 0 | 0 | 0 | 0 | 0 | 0 | 0 | 0 | 0 | 0 | 0 | 8 | 0 |
| OTU909 | 0 | 0 | 0 | 0 | 0 | 0 | 0 | 0 | 0 | 0 | 0 | 0 | 0 | 0 | 0 | 0 | 12 | 0 |
| OTU91 | 0 | 0 | 1 | 0 | 1 | 0 | 0 | 2 | 1 | 1 | 1 | 2 | 41 | 131 | 0 | 8 | 212 | 197 |
| OTU910 | 0 | 0 | 0 | 0 | 0 | 0 | 0 | 0 | 0 | 0 | 0 | 0 | 0 | 0 | 0 | 0 | 16 | 0 |
| OTU911 | 0 | 0 | 0 | 0 | 0 | 0 | 0 | 0 | 0 | 0 | 0 | 0 | 0 | 0 | 0 | 0 | 5 | 0 |
| OTU912 | 0 | 0 | 0 | 0 | 0 | 0 | 0 | 0 | 0 | 0 | 0 | 0 | 0 | 0 | 0 | 0 | 21 | 0 |
| OTU913 | 0 | 0 | 0 | 0 | 0 | 0 | 0 | 0 | 0 | 0 | 0 | 0 | 0 | 0 | 0 | 0 | 3 | 0 |
| OTU914 | 0 | 0 | 0 | 0 | 0 | 0 | 0 | 0 | 0 | 0 | 0 | 0 | 0 | 0 | 0 | 0 | 17 | 0 |
| OTU915 | 0 | 0 | 0 | 0 | 0 | 0 | 0 | 0 | 0 | 0 | 0 | 0 | 0 | 0 | 0 | 0 | 20 | 0 |
| OTU916 | 0 | 0 | 0 | 0 | 0 | 0 | 0 | 0 | 0 | 0 | 0 | 0 | 0 | 1 | 0 | 0 | 4 | 1 |
| OTU917 | 0 | 0 | 0 | 0 | 0 | 0 | 0 | 0 | 0 | 0 | 0 | 0 | 0 | 0 | 0 | 0 | 24 | 0 |
| OTU918 | 0 | 0 | 0 | 0 | 0 | 0 | 0 | 0 | 0 | 0 | 0 | 0 | 0 | 0 | 0 | 0 | 2 | 0 |
| OTU919 | 0 | 0 | 0 | 0 | 0 | 0 | 0 | 0 | 0 | 0 | 0 | 0 | 0 | 0 | 0 | 0 | 3 | 0 |
| OTU92 | 411 | 113 | 135 | 0 | 0 | 3 | 1 | 0 | 0 | 2 | 4 | 2 | 0 | 1 | 2 | 0 | 0 | 0 |
| OTU920 | 0 | 0 | 0 | 0 | 0 | 0 | 0 | 0 | 0 | 0 | 0 | 0 | 0 | 0 | 0 | 0 | 10 | 0 |
| OTU921 | 0 | 0 | 0 | 0 | 0 | 0 | 0 | 0 | 0 | 0 | 0 | 0 | 0 | 0 | 0 | 0 | 3 | 0 |
| OTU922 | 0 | 0 | 0 | 0 | 0 | 0 | 0 | 0 | 0 | 0 | 0 | 0 | 1 | 2 | 0 | 0 | 10 | 1 |
| OTU923 | 0 | 0 | 0 | 0 | 0 | 0 | 0 | 0 | 0 | 0 | 0 | 0 | 0 | 0 | 0 | 0 | 5 | 0 |
| OTU924 | 0 | 0 | 0 | 0 | 0 | 0 | 0 | 0 | 0 | 0 | 0 | 0 | 0 | 0 | 0 | 0 | 6 | 0 |
| OTU925 | 0 | 0 | 0 | 0 | 0 | 0 | 0 | 0 | 0 | 0 | 0 | 0 | 3 | 0 | 0 | 0 | 6 | 3 |
| OTU926 | 0 | 0 | 0 | 0 | 0 | 0 | 0 | 0 | 0 | 0 | 0 | 0 | 0 | 0 | 0 | 0 | 1 | 8 |
| OTU927 | 0 | 0 | 0 | 0 | 0 | 0 | 0 | 0 | 0 | 0 | 0 | 0 | 0 | 0 | 0 | 0 | 19 | 0 |
| OTU928 | 0 | 0 | 0 | 0 | 0 | 0 | 0 | 0 | 0 | 0 | 0 | 0 | 0 | 0 | 0 | 0 | 3 | 0 |
| OTU929 | 0 | 0 | 0 | 0 | 0 | 0 | 0 | 0 | 0 | 0 | 0 | 0 | 0 | 0 | 0 | 0 | 2 | 0 |
| OTU93 | 277 | 11 | 412 | 5 | 456 | 64 | 4 | 1 | 1 | 5 | 0 | 0 | 0 | 1 | 4 | 0 | 0 | 0 |
| OTU930 | 0 | 0 | 0 | 0 | 0 | 0 | 0 | 0 | 0 | 0 | 0 | 0 | 0 | 0 | 0 | 0 | 13 | 0 |
| OTU931 | 3 | 4 | 2 | 5 | 2 | 5 | 2 | 6 | 4 | 2 | 5 | 3 | 1 | 3 | 2 | 3 | 4 | 1 |
| OTU932 | 0 | 0 | 0 | 0 | 0 | 0 | 0 | 0 | 0 | 0 | 0 | 0 | 1 | 0 | 0 | 0 | 2 | 0 |
| OTU933 | 0 | 0 | 0 | 0 | 0 | 0 | 0 | 0 | 0 | 0 | 0 | 0 | 0 | 0 | 0 | 0 | 5 | 0 |
| OTU934 | 0 | 0 | 0 | 0 | 0 | 0 | 0 | 1 | 0 | 0 | 0 | 0 | 1 | 0 | 1 | 0 | 9 | 0 |
| OTU935 | 0 | 0 | 0 | 0 | 0 | 0 | 0 | 0 | 0 | 0 | 0 | 0 | 0 | 0 | 0 | 0 | 2 | 0 |
| OTU936 | 0 | 0 | 0 | 0 | 0 | 0 | 0 | 0 | 0 | 0 | 0 | 0 | 0 | 0 | 0 | 0 | 8 | 0 |
| OTU937 | 0 | 0 | 0 | 0 | 0 | 0 | 0 | 0 | 0 | 0 | 0 | 0 | 0 | 0 | 0 | 0 | 0 | 2 |
| OTU94 | 24 | 114 | 175 | 129 | 62 | 18 | 0 | 0 | 0 | 1 | 0 | 0 | 1 | 0 | 0 | 0 | 0 | 0 |
| OTU95 | 32 | 12 | 179 | 2 | 8 | 361 | 12 | 582 | 171 | 631 | 473 | 205 | 3 | 3 | 116 | 0 | 8 | 6 |
| OTU96 | 56 | 846 | 303 | 33 | 119 | 57 | 5 | 6 | 8 | 5 | 6 | 5 | 1 | 1 | 10 | 0 | 1 | 0 |
| OTU97 | 1 | 0 | 0 | 4 | 4 | 4 | 75 | 23 | 84 | 336 | 90 | 16 | 8 | 10 | 53 | 0 | 3 | 14 |
| OTU98 | 0 | 0 | 0 | 0 | 0 | 0 | 0 | 0 | 0 | 0 | 0 | 1 | 15 | 87 | 0 | 9 | 223 | 249 |
| OTU99 | 3 | 2 | 7 | 0 | 10 | 2 | 19 | 12 | 18 | 92 | 148 | 225 | 1 | 1 | 18 | 0 | 6 | 3 |

## genus count for Fig 7R

| genus | A1 | A2 | A3 | A4 | A5 | A6 | B1 | B2 | B3 | B4 | B5 | B6 | C1 | C2 | C3 | C4 | C5 | C6 |
| --- | --- | --- | --- | --- | --- | --- | --- | --- | --- | --- | --- | --- | --- | --- | --- | --- | --- | --- |
| g__14-2 | 13 | 30 | 30 | 10 | 413 | 29 | 5 | 64 | 21 | 172 | 156 | 44 | 1 | 8 | 19 | 0 | 10 | 1 |
| g__21-14-0-10-47-8-A | 0 | 0 | 0 | 0 | 0 | 0 | 0 | 0 | 0 | 0 | 0 | 0 | 0 | 0 | 0 | 0 | 6 | 0 |
| g__33-17 | 0 | 0 | 0 | 0 | 0 | 0 | 0 | 0 | 0 | 0 | 1 | 3 | 1 | 1 | 0 | 0 | 0 | 0 |
| g__49-20 | 4 | 7 | 4 | 2 | 6 | 7 | 5 | 5 | 7 | 9 | 10 | 4 | 1 | 9 | 3 | 8 | 5 | 4 |
| g__992a | 140 | 62 | 34 | 44 | 1396 | 1370 | 141 | 406 | 145 | 256 | 681 | 243 | 14 | 14 | 118 | 1 | 16 | 8 |
| g__ASF356 | 0 | 6 | 0 | 0 | 5 | 15 | 0 | 0 | 3 | 0 | 0 | 0 | 0 | 0 | 3 | 0 | 0 | 1 |
| g__Absiella | 68 | 1 | 8 | 0 | 3 | 1 | 7 | 5 | 23 | 8 | 12 | 12 | 8 | 8 | 17 | 0 | 2 | 1 |
| g__Acetatifactor | 0 | 0 | 0 | 0 | 0 | 0 | 0 | 4 | 1 | 1 | 0 | 0 | 0 | 0 | 0 | 0 | 0 | 0 |
| g__Acetitomaculum | 0 | 3 | 0 | 3 | 14 | 0 | 2 | 39 | 11 | 2 | 21 | 67 | 1 | 11 | 10 | 0 | 0 | 0 |
| g__Acetivibrio | 7 | 14 | 10 | 4 | 1 | 4 | 1 | 1 | 1 | 1 | 0 | 2 | 0 | 1 | 1 | 0 | 0 | 0 |
| g__Acetoanaerobium | 0 | 3 | 0 | 1 | 0 | 1 | 0 | 0 | 1 | 2 | 0 | 0 | 0 | 0 | 0 | 0 | 0 | 0 |
| g__Acetobacter | 0 | 0 | 0 | 0 | 0 | 0 | 0 | 0 | 0 | 0 | 0 | 0 | 0 | 0 | 0 | 0 | 16 | 0 |
| g__Achromobacter | 0 | 0 | 0 | 0 | 0 | 0 | 0 | 0 | 0 | 0 | 0 | 0 | 0 | 0 | 0 | 0 | 13 | 0 |
| g__Acinetobacter | 0 | 1 | 3 | 0 | 4 | 3 | 5 | 0 | 1 | 0 | 1 | 4 | 25 | 13 | 3 | 0 | 270 | 13 |
| g__Actinomadura_B | 1 | 1 | 1 | 1 | 1 | 0 | 0 | 1 | 2 | 0 | 0 | 0 | 2 | 0 | 1 | 0 | 0 | 0 |
| g__Actinomyces | 0 | 1 | 1 | 0 | 0 | 1 | 3 | 3 | 0 | 2 | 1 | 2 | 46 | 57 | 1 | 43 | 647 | 279 |
| g__Actinomyces_I | 1 | 2 | 1 | 0 | 0 | 4 | 1 | 4 | 1 | 0 | 1 | 2 | 1 | 1 | 0 | 1 | 7 | 1 |
| g__Acutalibacter | 30 | 20 | 45 | 41 | 70 | 94 | 25 | 243 | 129 | 234 | 232 | 438 | 41 | 143 | 122 | 0 | 13 | 5 |
| g__Adlercreutzia | 53 | 76 | 117 | 43 | 27 | 60 | 6 | 28 | 16 | 5 | 8 | 9 | 3 | 9 | 12 | 0 | 4 | 5 |
| g__Advenella | 0 | 0 | 0 | 0 | 0 | 0 | 0 | 0 | 0 | 0 | 0 | 0 | 0 | 0 | 0 | 0 | 297 | 0 |
| g__Agathobacter | 32 | 12 | 179 | 2 | 8 | 361 | 12 | 582 | 171 | 631 | 473 | 205 | 3 | 3 | 116 | 0 | 8 | 6 |
| g__Agathobaculum | 1 | 4 | 4 | 2 | 3 | 12 | 2 | 4 | 6 | 4 | 7 | 2 | 0 | 1 | 1 | 0 | 0 | 0 |
| g__Agrobacterium | 0 | 1 | 1 | 0 | 1 | 0 | 1 | 2 | 3 | 2 | 4 | 2 | 3 | 4 | 1 | 1 | 1037 | 4 |
| g__Alicyclobacillus_B | 0 | 1 | 0 | 0 | 0 | 1 | 1 | 1 | 1 | 0 | 0 | 1 | 0 | 1 | 1 | 1 | 0 | 0 |
| g__Alicyclobacillus_H | 0 | 1 | 0 | 0 | 0 | 0 | 0 | 2 | 0 | 0 | 0 | 1 | 1 | 0 | 0 | 1 | 0 | 0 |
| g__Alistipes | 1 | 4 | 19 | 13 | 47 | 12 | 11 | 0 | 16 | 5 | 4 | 0 | 1 | 0 | 13 | 0 | 0 | 2 |
| g__Alistipes_A | 0 | 6 | 0 | 4 | 29 | 2 | 0 | 0 | 0 | 0 | 1 | 0 | 0 | 0 | 0 | 0 | 0 | 0 |
| g__Allobaculum | 7 | 18 | 66 | 0 | 1 | 3 | 0 | 0 | 0 | 0 | 0 | 0 | 0 | 0 | 0 | 0 | 0 | 0 |
| g__Aminicenans | 0 | 1 | 0 | 0 | 0 | 1 | 0 | 0 | 1 | 0 | 2 | 2 | 0 | 1 | 0 | 0 | 0 | 0 |
| g__An181 | 9 | 148 | 23 | 1 | 19 | 0 | 0 | 0 | 0 | 0 | 0 | 0 | 0 | 0 | 0 | 0 | 0 | 0 |
| g__Anaerobiospirillum_A | 0 | 2 | 2 | 0 | 4 | 2 | 2 | 750 | 338 | 582 | 1203 | 765 | 6 | 6 | 66 | 1 | 8 | 2 |
| g__Anaerocolumna | 0 | 0 | 0 | 1 | 0 | 0 | 0 | 0 | 6 | 5 | 0 | 0 | 0 | 0 | 4 | 0 | 1 | 0 |
| g__Anaerofustis | 5 | 0 | 3 | 2 | 0 | 1 | 1 | 3 | 0 | 0 | 5 | 0 | 0 | 0 | 1 | 0 | 0 | 0 |
| g__Anaeromassilibacillus | 45 | 49 | 53 | 26 | 20 | 76 | 0 | 0 | 0 | 0 | 0 | 0 | 0 | 0 | 0 | 0 | 0 | 0 |
| g__Anaerostipes | 74 | 231 | 371 | 7 | 6 | 326 | 157 | 106 | 83 | 91 | 153 | 235 | 12 | 78 | 70 | 1 | 18 | 14 |
| g__Anaerotignum | 1 | 7 | 2 | 3 | 2 | 1 | 5 | 53 | 20 | 34 | 67 | 13 | 0 | 1 | 16 | 0 | 3 | 1 |
| g__Anaerotruncus | 5 | 26 | 13 | 52 | 120 | 96 | 3 | 6 | 3 | 2 | 4 | 8 | 0 | 0 | 5 | 0 | 0 | 1 |
| g__Anaerovibrio | 0 | 0 | 0 | 0 | 0 | 0 | 1 | 11 | 32 | 1 | 0 | 1 | 0 | 0 | 9 | 0 | 0 | 0 |
| g__Angelakisella | 5 | 2 | 2 | 6 | 13 | 12 | 2 | 7 | 9 | 8 | 10 | 6 | 0 | 1 | 4 | 0 | 0 | 0 |
| g__Aquamicrobium_A | 0 | 0 | 2 | 1 | 0 | 0 | 1 | 1 | 0 | 0 | 1 | 0 | 15 | 17 | 0 | 3 | 63 | 34 |
| g__Arboricoccus | 0 | 0 | 0 | 0 | 0 | 0 | 0 | 1 | 2 | 0 | 0 | 1 | 0 | 0 | 0 | 0 | 1 | 0 |
| g__Atopostipes | 0 | 0 | 0 | 0 | 0 | 0 | 0 | 0 | 0 | 0 | 0 | 0 | 0 | 0 | 0 | 0 | 11 | 1 |
| g__Bacillus | 0 | 1 | 0 | 1 | 0 | 0 | 0 | 0 | 0 | 0 | 0 | 0 | 7 | 5 | 0 | 0 | 71 | 19 |
| g__Bacillus_A | 0 | 0 | 0 | 0 | 0 | 0 | 0 | 0 | 0 | 0 | 0 | 0 | 0 | 0 | 0 | 1 | 66 | 2 |
| g__Bacillus_C | 1 | 0 | 0 | 0 | 0 | 1 | 0 | 0 | 1 | 1 | 0 | 0 | 3 | 4 | 3 | 2 | 27 | 10 |
| g__Bacillus_J | 0 | 0 | 0 | 0 | 0 | 0 | 0 | 0 | 0 | 0 | 0 | 0 | 1 | 4 | 0 | 1 | 28 | 3 |
| g__Bacillus_W | 5 | 1 | 1 | 1 | 0 | 0 | 0 | 0 | 1 | 1 | 1 | 1 | 3 | 1 | 0 | 4 | 4 | 3 |
| g__Bact-08 | 0 | 0 | 0 | 0 | 0 | 0 | 7 | 0 | 3 | 0 | 9 | 9 | 0 | 3 | 0 | 0 | 0 | 0 |
| g__Bacteroides | 177 | 333 | 286 | 485 | 420 | 139 | 166 | 49 | 52 | 80 | 34 | 149 | 18 | 39 | 37 | 0 | 11 | 4 |
| g__Bacteroides_B | 159 | 212 | 269 | 35 | 632 | 88 | 68 | 7 | 8 | 14 | 23 | 38 | 3 | 7 | 22 | 0 | 1 | 1 |
| g__Bacteroides_F | 76 | 27 | 1 | 8 | 1 | 408 | 0 | 52 | 202 | 4 | 5 | 427 | 22 | 58 | 129 | 0 | 10 | 1 |
| g__Beduini | 7 | 1 | 1 | 8 | 1 | 0 | 12 | 6 | 3 | 6 | 0 | 18 | 2 | 9 | 9 | 0 | 0 | 1 |
| g__Beta-01 | 0 | 0 | 0 | 0 | 0 | 0 | 0 | 0 | 0 | 0 | 0 | 0 | 1 | 7 | 0 | 0 | 1 | 6 |
| g__Bifidobacterium | 8 | 10 | 27 | 10 | 27 | 10 | 10 | 21 | 12 | 13 | 11 | 18 | 30 | 24 | 15 | 39 | 17 | 27 |
| g__Bilophila | 0 | 0 | 0 | 3 | 32 | 4 | 1 | 0 | 2 | 10 | 8 | 10 | 0 | 1 | 1 | 0 | 1 | 0 |
| g__Bittarella | 6 | 13 | 3 | 3 | 9 | 11 | 0 | 0 | 0 | 0 | 0 | 0 | 0 | 0 | 1 | 0 | 0 | 0 |
| g__Blastococcus | 6 | 55 | 1515 | 8 | 7 | 10 | 1 | 1 | 0 | 0 | 3 | 1 | 0 | 0 | 1 | 0 | 2 | 0 |
| g__Blautia_A | 56 | 851 | 313 | 39 | 120 | 57 | 5 | 13 | 14 | 7 | 6 | 10 | 4 | 12 | 12 | 0 | 2 | 1 |
| g__Bog-159 | 0 | 1 | 0 | 0 | 0 | 0 | 0 | 0 | 0 | 0 | 0 | 0 | 0 | 0 | 1 | 0 | 0 | 0 |
| g__Bosea | 0 | 0 | 0 | 0 | 0 | 0 | 0 | 0 | 0 | 0 | 0 | 0 | 0 | 1 | 0 | 0 | 18 | 5 |
| g__Brachybacterium | 0 | 0 | 0 | 0 | 0 | 0 | 0 | 1 | 0 | 1 | 0 | 0 | 10 | 10 | 0 | 0 | 46 | 16 |
| g__Brachyspira | 0 | 0 | 0 | 0 | 0 | 0 | 0 | 0 | 0 | 0 | 0 | 0 | 0 | 0 | 0 | 0 | 10 | 0 |
| g__Bradyrhizobium | 0 | 1 | 1 | 0 | 0 | 0 | 0 | 0 | 1 | 0 | 2 | 0 | 4 | 3 | 0 | 0 | 13 | 8 |
| g__Brevibacillus | 0 | 0 | 0 | 0 | 0 | 0 | 0 | 0 | 1 | 0 | 0 | 0 | 2 | 2 | 0 | 0 | 6 | 1 |
| g__Brevibacterium | 0 | 1 | 0 | 0 | 0 | 0 | 0 | 0 | 0 | 0 | 0 | 0 | 11 | 5 | 0 | 0 | 64 | 17 |
| g__Brevundimonas | 0 | 0 | 0 | 0 | 0 | 0 | 0 | 0 | 0 | 0 | 0 | 0 | 15 | 2 | 0 | 0 | 17 | 7 |
| g__Butyricicoccus | 0 | 5 | 2 | 1 | 0 | 14 | 0 | 15 | 1 | 24 | 24 | 5 | 2 | 2 | 5 | 0 | 1 | 0 |
| g__Butyricicoccus_A | 0 | 0 | 1 | 1 | 0 | 0 | 1 | 0 | 1 | 0 | 2 | 3 | 1 | 1 | 1 | 0 | 0 | 0 |
| g__Butyricimonas | 2 | 8 | 6 | 2 | 8 | 6 | 0 | 0 | 0 | 1 | 1 | 0 | 0 | 0 | 0 | 0 | 0 | 0 |
| g__Butyrivibrio | 1 | 0 | 0 | 0 | 0 | 6 | 0 | 3 | 0 | 0 | 1 | 0 | 0 | 0 | 0 | 0 | 0 | 0 |
| g__Butyrivibrio_A | 2 | 14 | 13 | 3 | 10 | 137 | 5 | 505 | 1538 | 23 | 961 | 862 | 23 | 53 | 939 | 2 | 49 | 31 |
| g__CAG-1031 | 50 | 21 | 49 | 34 | 62 | 76 | 8 | 2 | 11 | 16 | 30 | 6 | 0 | 3 | 12 | 0 | 3 | 0 |
| g__CAG-110 | 34 | 15 | 35 | 6 | 2 | 29 | 8 | 1 | 2 | 4 | 9 | 1 | 1 | 1 | 4 | 0 | 0 | 1 |
| g__CAG-194 | 0 | 4 | 2 | 0 | 9 | 13 | 10 | 65 | 95 | 31 | 58 | 21 | 2 | 4 | 67 | 0 | 8 | 4 |
| g__CAG-217 | 35 | 564 | 56 | 12 | 176 | 135 | 77 | 1 | 101 | 11 | 8 | 3 | 0 | 2 | 170 | 0 | 3 | 1 |
| g__CAG-302 | 1 | 2 | 16 | 1 | 0 | 3 | 7 | 0 | 2 | 0 | 0 | 1 | 0 | 0 | 1 | 0 | 0 | 0 |
| g__CAG-306 | 3 | 1 | 1 | 9 | 5 | 3 | 1 | 2 | 2 | 4 | 5 | 8 | 1 | 9 | 3 | 0 | 2 | 2 |
| g__CAG-353 | 5 | 0 | 22 | 0 | 0 | 1 | 12 | 141 | 3 | 217 | 126 | 21 | 1 | 0 | 8 | 0 | 6 | 2 |
| g__CAG-41 | 51 | 112 | 42 | 55 | 52 | 201 | 168 | 229 | 494 | 431 | 881 | 564 | 73 | 260 | 594 | 0 | 61 | 35 |
| g__CAG-45 | 0 | 105 | 1 | 0 | 0 | 0 | 0 | 0 | 0 | 1 | 0 | 1 | 0 | 0 | 0 | 0 | 0 | 0 |
| g__CAG-484 | 3 | 5 | 0 | 3 | 0 | 6 | 8 | 3 | 2 | 3 | 14 | 4 | 1 | 4 | 3 | 0 | 0 | 0 |
| g__CAG-495 | 2 | 4 | 1 | 2 | 7 | 0 | 26 | 4 | 2 | 3 | 1 | 2 | 1 | 1 | 2 | 0 | 0 | 0 |
| g__CAG-791 | 1 | 0 | 3 | 0 | 0 | 0 | 3 | 35 | 12 | 3 | 12 | 14 | 1 | 5 | 2 | 0 | 0 | 1 |
| g__CAG-81 | 1 | 4 | 6 | 1 | 21 | 170 | 1 | 22 | 17 | 20 | 8 | 16 | 5 | 12 | 15 | 0 | 2 | 2 |
| g__CAG-822 | 1 | 0 | 0 | 0 | 0 | 0 | 5 | 5 | 48 | 1 | 0 | 24 | 3 | 8 | 47 | 0 | 2 | 1 |
| g__CAG-878 | 21 | 0 | 17 | 0 | 1 | 1 | 0 | 16 | 2 | 0 | 0 | 1 | 0 | 0 | 4 | 0 | 0 | 0 |
| g__CAG-95 | 2 | 9 | 48 | 2 | 6 | 53 | 7 | 145 | 158 | 122 | 161 | 260 | 16 | 22 | 106 | 0 | 10 | 3 |
| g__CAG-988 | 0 | 0 | 0 | 0 | 0 | 1 | 0 | 0 | 5 | 1 | 0 | 0 | 0 | 1 | 3 | 0 | 0 | 0 |
| g__CDF | 0 | 0 | 0 | 0 | 0 | 0 | 0 | 0 | 0 | 0 | 0 | 0 | 1 | 1 | 0 | 1 | 19 | 3 |
| g__CG2-30-66-27 | 1 | 2 | 2 | 0 | 3 | 5 | 2 | 3 | 2 | 5 | 2 | 3 | 1 | 1 | 2 | 1 | 1 | 0 |
| g__CHKCI006 | 0 | 87 | 0 | 0 | 1 | 1 | 3 | 0 | 1 | 0 | 1 | 6 | 0 | 1 | 0 | 0 | 0 | 0 |
| g__COE1 | 28 | 1 | 1 | 36 | 7 | 65 | 4 | 34 | 8 | 17 | 15 | 7 | 1 | 0 | 6 | 0 | 1 | 0 |
| g__Caldilinea | 0 | 0 | 0 | 0 | 0 | 0 | 0 | 0 | 0 | 0 | 0 | 0 | 0 | 0 | 0 | 0 | 2 | 0 |
| g__Christensenella | 27 | 13 | 17 | 15 | 24 | 18 | 6 | 12 | 3 | 10 | 10 | 14 | 2 | 3 | 9 | 0 | 1 | 0 |
| g__Christensenella_A | 2 | 0 | 1 | 4 | 1 | 4 | 4 | 0 | 6 | 3 | 3 | 5 | 1 | 6 | 2 | 0 | 0 | 1 |
| g__Chryseobacterium | 141 | 1285 | 1562 | 622 | 209 | 697 | 108 | 367 | 78 | 50 | 56 | 140 | 12 | 19 | 106 | 1 | 7 | 8 |
| g__Citrobacter | 0 | 0 | 0 | 0 | 1 | 1 | 0 | 0 | 0 | 0 | 0 | 0 | 2 | 4 | 0 | 1 | 63 | 19 |
| g__Clostridium | 0 | 0 | 1 | 0 | 1 | 5 | 0 | 0 | 0 | 3 | 0 | 0 | 8 | 3 | 0 | 1 | 50 | 4 |
| g__Clostridium_A | 0 | 0 | 0 | 0 | 0 | 0 | 1 | 3 | 2 | 1 | 3 | 2 | 0 | 0 | 2 | 0 | 0 | 0 |
| g__Clostridium_AM | 0 | 0 | 0 | 0 | 0 | 0 | 0 | 1 | 0 | 0 | 0 | 0 | 0 | 0 | 0 | 0 | 26 | 0 |
| g__Clostridium_B | 0 | 0 | 0 | 0 | 0 | 0 | 0 | 0 | 0 | 0 | 0 | 0 | 0 | 0 | 0 | 0 | 86 | 0 |
| g__Clostridium_C | 0 | 1 | 0 | 0 | 0 | 0 | 0 | 0 | 0 | 0 | 0 | 0 | 0 | 0 | 0 | 0 | 27 | 0 |
| g__Clostridium_H | 0 | 0 | 0 | 0 | 0 | 0 | 0 | 0 | 0 | 0 | 0 | 0 | 0 | 0 | 0 | 0 | 5 | 0 |
| g__Clostridium_N | 3 | 47 | 3 | 47 | 0 | 1 | 0 | 17 | 58 | 1 | 0 | 6 | 2 | 1 | 33 | 0 | 7 | 2 |
| g__Clostridium_Q | 18 | 23 | 55 | 18 | 779 | 351 | 7 | 126 | 99 | 112 | 88 | 70 | 3 | 9 | 65 | 0 | 10 | 9 |
| g__Clostridium_S | 0 | 0 | 0 | 0 | 0 | 0 | 1 | 0 | 0 | 0 | 0 | 0 | 1 | 0 | 0 | 0 | 178 | 0 |
| g__Collinsella | 8 | 10 | 7 | 1 | 0 | 2 | 3 | 13 | 9 | 11 | 7 | 8 | 1 | 8 | 14 | 0 | 2 | 0 |
| g__Comamonas_D | 0 | 0 | 0 | 0 | 0 | 0 | 0 | 0 | 0 | 0 | 0 | 0 | 0 | 0 | 0 | 0 | 7 | 0 |
| g__Coprobacter | 1 | 0 | 3 | 3 | 0 | 2 | 0 | 0 | 0 | 0 | 0 | 0 | 0 | 0 | 0 | 0 | 0 | 0 |
| g__Coprococcus_B | 1 | 8 | 11 | 0 | 0 | 28 | 1 | 30 | 4 | 15 | 25 | 6 | 3 | 4 | 4 | 0 | 1 | 0 |
| g__Corynebacterium | 2 | 4 | 3 | 3 | 2 | 1 | 5 | 9 | 6 | 8 | 7 | 6 | 157 | 308 | 3 | 15 | 699 | 501 |
| g__Cronobacter | 10870 | 17262 | 10095 | 10552 | 9337 | 8871 | 5817 | 4539 | 3421 | 8329 | 7306 | 4970 | 973 | 2355 | 2984 | 1554 | 526 | 280 |
| g__Cryobacterium_A | 1 | 0 | 0 | 0 | 0 | 0 | 0 | 0 | 1 | 0 | 0 | 0 | 1 | 5 | 0 | 0 | 11 | 7 |
| g__DNF00809 | 10 | 6 | 17 | 9 | 8 | 5 | 5 | 17 | 4 | 3 | 7 | 10 | 0 | 18 | 7 | 0 | 2 | 3 |
| g__DTU053 | 9 | 4 | 2 | 0 | 7 | 0 | 0 | 0 | 0 | 0 | 0 | 0 | 0 | 0 | 0 | 0 | 0 | 0 |
| g__Dakarella | 0 | 0 | 0 | 0 | 0 | 0 | 0 | 0 | 0 | 0 | 0 | 0 | 0 | 0 | 0 | 0 | 152 | 1 |
| g__Defluviitalea | 1 | 0 | 6 | 0 | 0 | 3 | 1 | 0 | 1 | 0 | 0 | 4 | 0 | 0 | 1 | 0 | 0 | 0 |
| g__Dehalobacterium | 9 | 11 | 4 | 0 | 2 | 1 | 1 | 0 | 1 | 1 | 1 | 3 | 0 | 0 | 0 | 0 | 0 | 0 |
| g__Denitrobacterium | 0 | 0 | 0 | 0 | 0 | 0 | 19 | 14 | 18 | 5 | 7 | 20 | 0 | 9 | 13 | 0 | 3 | 0 |
| g__Desulfonispora | 2 | 3 | 23 | 3 | 1 | 0 | 5 | 0 | 0 | 0 | 0 | 0 | 0 | 0 | 0 | 0 | 0 | 0 |
| g__Desulfovibrio | 1 | 160 | 78 | 2 | 208 | 360 | 46 | 507 | 16 | 308 | 358 | 369 | 30 | 6 | 270 | 1 | 27 | 19 |
| g__Desulfuromonas_B | 0 | 0 | 0 | 0 | 0 | 0 | 0 | 0 | 0 | 0 | 0 | 0 | 0 | 1 | 0 | 0 | 4 | 1 |
| g__Devosia | 0 | 0 | 0 | 0 | 0 | 0 | 0 | 0 | 0 | 0 | 0 | 0 | 10 | 13 | 1 | 0 | 121 | 35 |
| g__Dialister | 0 | 0 | 0 | 0 | 0 | 0 | 0 | 0 | 0 | 0 | 0 | 0 | 0 | 0 | 0 | 0 | 24 | 0 |
| g__Dietzia | 0 | 0 | 0 | 0 | 0 | 0 | 0 | 0 | 0 | 0 | 0 | 0 | 8 | 40 | 0 | 1 | 56 | 35 |
| g__Dorea | 12 | 63 | 55 | 23 | 77 | 130 | 26 | 204 | 206 | 249 | 266 | 138 | 10 | 45 | 153 | 2 | 23 | 18 |
| g__Duncaniella | 540 | 366 | 528 | 877 | 600 | 491 | 158 | 144 | 126 | 443 | 190 | 130 | 23 | 48 | 84 | 0 | 15 | 26 |
| g__ER4 | 2 | 0 | 0 | 0 | 3 | 8 | 0 | 1 | 0 | 1 | 2 | 2 | 0 | 0 | 0 | 0 | 0 | 0 |
| g__Effusibacillus | 0 | 3 | 3 | 3 | 2 | 0 | 1 | 3 | 0 | 0 | 1 | 0 | 2 | 5 | 4 | 2 | 0 | 0 |
| g__Eggerthella | 0 | 0 | 0 | 0 | 0 | 0 | 2 | 13 | 12 | 15 | 5 | 7 | 4 | 15 | 8 | 0 | 1 | 0 |
| g__Eisenbergiella | 13 | 3 | 3 | 1 | 23 | 23 | 2 | 24 | 12 | 14 | 15 | 7 | 0 | 1 | 7 | 0 | 0 | 0 |
| g__Emergencia | 46 | 42 | 38 | 17 | 30 | 33 | 13 | 5 | 4 | 8 | 6 | 8 | 3 | 0 | 9 | 1 | 0 | 1 |
| g__Enterococcus | 180 | 207 | 165 | 163 | 248 | 233 | 197 | 382 | 282 | 342 | 228 | 256 | 591 | 602 | 341 | 760 | 223 | 390 |
| g__Enteroscipio | 1 | 4 | 20 | 2 | 5 | 3 | 4 | 12 | 12 | 15 | 14 | 10 | 3 | 4 | 6 | 0 | 2 | 1 |
| g__Erwinia | 4 | 0 | 1 | 3 | 0 | 20 | 0 | 0 | 0 | 1 | 2 | 3 | 0 | 0 | 0 | 0 | 0 | 0 |
| g__Erysipelatoclostridium | 0 | 28 | 2 | 0 | 0 | 22 | 11 | 3 | 10 | 0 | 0 | 22 | 1 | 2 | 6 | 0 | 2 | 1 |
| g__Escherichia | 3 | 2 | 3 | 1 | 1 | 3 | 53 | 149 | 730 | 349 | 177 | 328 | 9 | 37 | 378 | 0 | 21 | 12 |
| g__Eubacterium_A | 0 | 0 | 0 | 0 | 0 | 0 | 2 | 3 | 5 | 0 | 0 | 1 | 1 | 1 | 3 | 0 | 2 | 0 |
| g__Eubacterium_C | 0 | 1 | 0 | 0 | 0 | 2 | 2 | 14 | 6 | 7 | 9 | 4 | 0 | 1 | 7 | 0 | 1 | 0 |
| g__Eubacterium_F | 0 | 0 | 0 | 0 | 0 | 0 | 2 | 39 | 35 | 0 | 2 | 75 | 6 | 10 | 32 | 0 | 1 | 0 |
| g__Eubacterium_G | 14 | 4 | 19 | 8 | 12 | 2 | 9 | 6 | 0 | 26 | 16 | 17 | 0 | 0 | 0 | 0 | 1 | 0 |
| g__Eubacterium_I | 12 | 0 | 30 | 0 | 1 | 0 | 1 | 0 | 0 | 1 | 0 | 2 | 0 | 0 | 0 | 0 | 0 | 0 |
| g__Eubacterium_J | 0 | 8 | 9 | 68 | 0 | 9 | 0 | 4 | 10 | 5 | 2 | 2 | 0 | 1 | 3 | 0 | 1 | 0 |
| g__Eubacterium_Q | 3 | 82 | 2 | 118 | 1 | 223 | 10 | 33 | 265 | 9 | 240 | 169 | 8 | 10 | 198 | 0 | 7 | 14 |
| g__Eubacterium_R | 1836 | 245 | 885 | 1141 | 444 | 309 | 1552 | 486 | 437 | 435 | 311 | 553 | 56 | 246 | 428 | 2 | 66 | 32 |
| g__Exiguobacterium | 3 | 4 | 2 | 2 | 6 | 2 | 8 | 6 | 8 | 5 | 4 | 3 | 7 | 2 | 2 | 11 | 4 | 4 |
| g__Exiguobacterium_A | 4 | 10 | 4 | 2 | 7 | 3 | 10 | 7 | 5 | 12 | 6 | 9 | 21 | 15 | 8 | 22 | 4 | 19 |
| g__F0428 | 0 | 0 | 0 | 0 | 0 | 0 | 0 | 0 | 0 | 0 | 0 | 0 | 0 | 1 | 0 | 0 | 5 | 3 |
| g__Facklamia | 0 | 0 | 0 | 0 | 0 | 0 | 1 | 0 | 0 | 0 | 0 | 0 | 1 | 0 | 0 | 1 | 14 | 4 |
| g__Faecalibacterium | 0 | 0 | 0 | 0 | 2 | 2 | 1 | 2 | 0 | 1 | 1 | 1 | 0 | 0 | 1 | 0 | 20 | 0 |
| g__Faecalibaculum | 9 | 13 | 146 | 4 | 2 | 20 | 0 | 0 | 0 | 0 | 0 | 1 | 0 | 0 | 0 | 0 | 0 | 1 |
| g__Faecalicatena | 38 | 15 | 93 | 58 | 91 | 375 | 18 | 301 | 72 | 200 | 324 | 60 | 18 | 10 | 64 | 1 | 8 | 8 |
| g__Fermentibacter | 1 | 1 | 0 | 0 | 0 | 1 | 0 | 2 | 1 | 0 | 1 | 0 | 1 | 2 | 0 | 3 | 1 | 0 |
| g__Flavisolibacter | 0 | 0 | 0 | 0 | 2 | 0 | 0 | 0 | 0 | 2 | 0 | 0 | 0 | 0 | 0 | 0 | 0 | 0 |
| g__Flavobacterium | 0 | 0 | 0 | 0 | 0 | 0 | 0 | 0 | 0 | 0 | 0 | 0 | 0 | 0 | 0 | 0 | 7 | 0 |
| g__Flavobacterium_A | 0 | 0 | 0 | 0 | 0 | 0 | 0 | 0 | 0 | 0 | 0 | 0 | 0 | 0 | 0 | 0 | 5 | 1 |
| g__Flavonifractor | 50 | 91 | 108 | 106 | 140 | 137 | 185 | 523 | 292 | 391 | 371 | 375 | 29 | 56 | 182 | 2 | 17 | 14 |
| g__Fournierella | 30 | 43 | 33 | 14 | 107 | 38 | 13 | 115 | 30 | 57 | 8 | 15 | 2 | 4 | 18 | 0 | 3 | 0 |
| g__GCA-900066575 | 15 | 51 | 32 | 22 | 13 | 43 | 4 | 99 | 52 | 48 | 61 | 42 | 2 | 3 | 37 | 0 | 10 | 1 |
| g__GCA-900066905 | 0 | 0 | 1 | 0 | 1 | 0 | 0 | 0 | 0 | 0 | 0 | 0 | 0 | 0 | 1 | 0 | 0 | 0 |
| g__GW-Nitrospira-1 | 0 | 1 | 1 | 0 | 1 | 0 | 1 | 2 | 0 | 1 | 1 | 2 | 0 | 0 | 0 | 0 | 0 | 0 |
| g__Gemmata | 0 | 0 | 0 | 0 | 0 | 0 | 0 | 0 | 0 | 0 | 0 | 0 | 0 | 0 | 0 | 0 | 3 | 0 |
| g__Globicatella | 0 | 0 | 0 | 0 | 0 | 0 | 2 | 0 | 1 | 0 | 1 | 4 | 0 | 0 | 0 | 0 | 0 | 0 |
| g__Gordonibacter | 2 | 6 | 2 | 7 | 11 | 6 | 0 | 3 | 1 | 3 | 0 | 4 | 1 | 3 | 0 | 0 | 0 | 0 |
| g__Gracilibacter | 0 | 0 | 0 | 0 | 1 | 0 | 1 | 0 | 0 | 0 | 0 | 2 | 0 | 0 | 0 | 0 | 0 | 0 |
| g__Halomonas | 0 | 0 | 0 | 0 | 0 | 0 | 0 | 0 | 0 | 0 | 0 | 0 | 2 | 2 | 0 | 2 | 22 | 8 |
| g__Halomonas_D | 0 | 1 | 0 | 0 | 0 | 0 | 1 | 0 | 1 | 0 | 0 | 0 | 0 | 0 | 0 | 1 | 0 | 2 |
| g__Helicobacter_C | 92 | 18 | 105 | 46 | 22 | 283 | 1 | 4 | 11 | 31 | 2 | 57 | 4 | 6 | 21 | 0 | 0 | 2 |
| g__Helicobacter_D | 1 | 2 | 6 | 3 | 0 | 22 | 3 | 31 | 12 | 265 | 35 | 34 | 1 | 3 | 55 | 0 | 1 | 2 |
| g__Holdemania | 4 | 3 | 11 | 0 | 3 | 9 | 0 | 4 | 3 | 3 | 7 | 5 | 0 | 0 | 0 | 0 | 0 | 0 |
| g__Hydrogenoanaerobacterium | 3 | 3 | 5 | 1 | 3 | 1 | 2 | 2 | 1 | 0 | 1 | 3 | 0 | 1 | 0 | 0 | 0 | 0 |
| g__Hydrogenophaga | 0 | 0 | 0 | 0 | 0 | 0 | 0 | 0 | 0 | 0 | 1 | 0 | 0 | 0 | 0 | 0 | 151 | 0 |
| g__Hyphomicrobium | 2 | 0 | 0 | 0 | 1 | 2 | 0 | 1 | 2 | 0 | 3 | 1 | 0 | 0 | 1 | 0 | 2 | 0 |
| g__Intestinibacillus | 0 | 0 | 0 | 0 | 0 | 0 | 0 | 1 | 1 | 1 | 1 | 2 | 0 | 0 | 1 | 0 | 1 | 0 |
| g__Intestinimonas | 15 | 177 | 71 | 18 | 18 | 7 | 175 | 63 | 114 | 80 | 374 | 83 | 26 | 54 | 87 | 0 | 10 | 8 |
| g__JJ008 | 0 | 0 | 0 | 0 | 0 | 0 | 0 | 0 | 0 | 0 | 0 | 0 | 0 | 0 | 0 | 0 | 7 | 0 |
| g__Jeotgalicoccus | 0 | 0 | 0 | 0 | 0 | 0 | 0 | 0 | 0 | 0 | 0 | 0 | 10 | 2 | 0 | 1 | 30 | 8 |
| g__Kineothrix | 251 | 338 | 38 | 103 | 111 | 1842 | 18 | 2170 | 697 | 1723 | 3232 | 1200 | 77 | 330 | 406 | 0 | 76 | 40 |
| g__Klebsiella | 31 | 68 | 89 | 32 | 60 | 79 | 40 | 85 | 53 | 76 | 90 | 68 | 83 | 82 | 54 | 43 | 577 | 254 |
| g__LD21 | 12 | 14 | 20 | 9 | 30 | 29 | 20 | 7 | 8 | 13 | 16 | 13 | 1 | 5 | 8 | 0 | 5 | 4 |
| g__Lachnoclostridium_A | 6 | 12 | 7 | 6 | 24 | 11 | 0 | 15 | 14 | 11 | 7 | 13 | 2 | 10 | 6 | 0 | 0 | 0 |
| g__Lachnospira | 0 | 12 | 0 | 18 | 0 | 35 | 0 | 0 | 1 | 0 | 1 | 0 | 0 | 0 | 0 | 0 | 0 | 0 |
| g__Lachnotalea | 0 | 0 | 2 | 0 | 1 | 4 | 0 | 0 | 0 | 0 | 0 | 0 | 0 | 0 | 0 | 0 | 0 | 0 |
| g__Lactobacillus | 5364 | 1419 | 2460 | 4453 | 1594 | 1381 | 6343 | 6769 | 5342 | 8391 | 4350 | 5391 | 10710 | 1850 | 10093 | 7406 | 915 | 34937 |
| g__Lactobacillus_B | 381 | 3230 | 1307 | 215 | 4385 | 2353 | 184 | 675 | 492 | 406 | 103 | 144 | 6828 | 16355 | 677 | 20500 | 208 | 107 |
| g__Lactobacillus_E | 0 | 0 | 0 | 0 | 0 | 0 | 0 | 0 | 0 | 0 | 0 | 0 | 0 | 0 | 0 | 0 | 19 | 0 |
| g__Lactobacillus_F | 1 | 0 | 0 | 1 | 1 | 0 | 0 | 3 | 1 | 0 | 0 | 0 | 0 | 5 | 1 | 1 | 2 | 2 |
| g__Lactobacillus_G | 0 | 0 | 0 | 0 | 0 | 0 | 0 | 0 | 0 | 0 | 0 | 0 | 0 | 0 | 0 | 0 | 8 | 0 |
| g__Lactobacillus_H | 2388 | 130 | 660 | 1393 | 693 | 779 | 1117 | 2743 | 2409 | 1755 | 731 | 979 | 11350 | 7913 | 3215 | 9927 | 4104 | 11377 |
| g__Lawsonibacter | 44 | 89 | 104 | 154 | 265 | 309 | 112 | 758 | 525 | 1947 | 1569 | 821 | 26 | 84 | 305 | 1 | 50 | 21 |
| g__Lentimicrobium | 0 | 1 | 0 | 0 | 0 | 0 | 2 | 7 | 22 | 46 | 43 | 34 | 0 | 1 | 18 | 0 | 0 | 1 |
| g__Listeria | 0 | 0 | 0 | 0 | 0 | 0 | 0 | 0 | 0 | 0 | 0 | 0 | 0 | 0 | 0 | 0 | 1 | 8 |
| g__Longilinea | 1 | 0 | 1 | 0 | 1 | 0 | 0 | 0 | 2 | 1 | 1 | 0 | 1 | 2 | 0 | 0 | 5 | 0 |
| g__Luteimonas | 0 | 0 | 0 | 0 | 0 | 0 | 0 | 0 | 0 | 0 | 0 | 0 | 3 | 5 | 0 | 0 | 37 | 6 |
| g__Lysinibacillus_A | 0 | 0 | 0 | 0 | 0 | 0 | 0 | 0 | 1 | 1 | 0 | 0 | 1 | 16 | 0 | 0 | 21 | 2 |
| g__Lysinibacillus_D | 0 | 0 | 0 | 0 | 0 | 0 | 0 | 0 | 0 | 0 | 0 | 0 | 5 | 0 | 0 | 0 | 101 | 5 |
| g__MS4 | 0 | 2 | 0 | 1 | 2 | 1 | 7 | 27 | 14 | 22 | 11 | 45 | 3 | 8 | 8 | 0 | 1 | 1 |
| g__MYbin3 | 18 | 20 | 19 | 6 | 16 | 20 | 13 | 28 | 26 | 26 | 42 | 32 | 8 | 10 | 19 | 1 | 28 | 7 |
| g__Marseille-P3106 | 2 | 13 | 12 | 5 | 11 | 11 | 57 | 34 | 36 | 53 | 69 | 72 | 7 | 29 | 35 | 0 | 6 | 1 |
| g__Marvinbryantia | 10 | 32 | 14 | 3 | 7 | 12 | 3 | 9 | 1 | 0 | 15 | 1 | 1 | 0 | 2 | 0 | 1 | 1 |
| g__Massilia | 0 | 0 | 0 | 0 | 1 | 1 | 0 | 0 | 0 | 0 | 1 | 0 | 0 | 0 | 0 | 0 | 15 | 0 |
| g__Massilimaliae | 12 | 5 | 11 | 4 | 12 | 12 | 0 | 45 | 16 | 1 | 5 | 21 | 0 | 2 | 17 | 0 | 3 | 0 |
| g__Massilioclostridium | 5 | 0 | 10 | 2 | 13 | 2 | 4 | 71 | 68 | 36 | 41 | 28 | 0 | 5 | 50 | 0 | 6 | 5 |
| g__Megamonas | 40 | 131 | 204 | 10 | 883 | 654 | 2 | 0 | 1 | 1 | 1 | 1 | 3 | 1 | 1 | 3 | 0 | 2 |
| g__Megasphaera | 1 | 0 | 0 | 0 | 0 | 0 | 0 | 0 | 0 | 0 | 0 | 0 | 1 | 1 | 0 | 1 | 386 | 0 |
| g__Meiothermus_B | 7 | 6 | 10 | 5 | 8 | 5 | 7 | 10 | 11 | 13 | 9 | 10 | 4 | 2 | 9 | 5 | 14 | 6 |
| g__Mesotoga | 0 | 1 | 2 | 3 | 1 | 2 | 0 | 12 | 1 | 1 | 1 | 0 | 0 | 0 | 0 | 0 | 0 | 0 |
| g__Methanosphaera | 5 | 16 | 35 | 0 | 5 | 33 | 0 | 2 | 0 | 1 | 6 | 26 | 0 | 0 | 0 | 0 | 0 | 1 |
| g__Methanothrix | 0 | 0 | 0 | 0 | 1 | 1 | 0 | 1 | 0 | 0 | 1 | 0 | 2 | 0 | 0 | 0 | 0 | 1 |
| g__Methylocystis | 0 | 0 | 0 | 0 | 0 | 0 | 0 | 1 | 1 | 1 | 1 | 0 | 0 | 0 | 0 | 0 | 6 | 0 |
| g__Microcystis | 1 | 1 | 3 | 1 | 1 | 1 | 2 | 6 | 3 | 1 | 2 | 0 | 2 | 5 | 2 | 2 | 2 | 0 |
| g__Monoglobus | 1 | 1 | 2 | 5 | 2 | 1 | 33 | 33 | 47 | 48 | 75 | 41 | 13 | 24 | 64 | 0 | 4 | 3 |
| g__Morganella | 7 | 6 | 12 | 5 | 5 | 14 | 6 | 11 | 12 | 11 | 11 | 10 | 2 | 8 | 4 | 2 | 23 | 7 |
| g__Muribaculum | 3 | 4 | 8 | 7 | 4 | 9 | 234 | 108 | 427 | 207 | 70 | 286 | 38 | 112 | 447 | 0 | 7 | 13 |
| g__NAK82 | 4 | 4 | 1 | 2 | 9 | 8 | 1 | 3 | 8 | 4 | 8 | 7 | 2 | 6 | 3 | 3 | 4 | 1 |
| g__Negativibacillus | 6 | 35 | 10 | 0 | 0 | 7 | 0 | 4 | 6 | 2 | 0 | 1 | 0 | 0 | 9 | 0 | 0 | 0 |
| g__Nioella | 0 | 0 | 0 | 0 | 0 | 0 | 0 | 0 | 0 | 0 | 0 | 0 | 0 | 0 | 0 | 0 | 0 | 2 |
| g__Nocardiopsis | 0 | 0 | 0 | 0 | 0 | 0 | 0 | 0 | 0 | 0 | 0 | 0 | 0 | 0 | 0 | 0 | 5 | 2 |
| g__Nosocomiicoccus | 0 | 0 | 0 | 0 | 0 | 0 | 0 | 0 | 0 | 0 | 0 | 0 | 9 | 1 | 0 | 0 | 25 | 12 |
| g__OEMS01 | 134 | 439 | 134 | 42 | 229 | 220 | 86 | 90 | 151 | 80 | 201 | 121 | 18 | 40 | 246 | 0 | 23 | 17 |
| g__Oceanisphaera | 0 | 0 | 0 | 0 | 0 | 0 | 0 | 0 | 0 | 0 | 0 | 0 | 6 | 10 | 0 | 4 | 27 | 151 |
| g__Odoribacter | 3 | 5 | 4 | 0 | 5 | 4 | 7 | 13 | 3 | 19 | 15 | 10 | 1 | 2 | 2 | 0 | 1 | 0 |
| g__Oligella | 0 | 0 | 0 | 0 | 0 | 0 | 0 | 0 | 0 | 0 | 0 | 1 | 15 | 87 | 0 | 9 | 223 | 249 |
| g__Olsenella | 3 | 1 | 6 | 0 | 0 | 2 | 0 | 0 | 0 | 0 | 0 | 0 | 0 | 0 | 0 | 0 | 3 | 1 |
| g__Oscillibacter | 10 | 20 | 21 | 30 | 76 | 177 | 14 | 76 | 44 | 200 | 90 | 49 | 3 | 0 | 35 | 0 | 3 | 1 |
| g__Paenalcaligenes | 0 | 0 | 0 | 0 | 0 | 0 | 0 | 1 | 0 | 0 | 0 | 0 | 17 | 1 | 0 | 9 | 169 | 267 |
| g__Pannonibacter | 0 | 1 | 0 | 0 | 0 | 0 | 0 | 0 | 0 | 0 | 0 | 0 | 0 | 0 | 1 | 0 | 58 | 0 |
| g__Papillibacter | 16 | 12 | 16 | 1 | 8 | 11 | 22 | 5 | 8 | 7 | 12 | 11 | 2 | 4 | 2 | 0 | 3 | 2 |
| g__Parabacteroides | 42 | 76 | 80 | 69 | 137 | 48 | 9 | 4 | 7 | 8 | 18 | 31 | 3 | 9 | 15 | 0 | 58 | 0 |
| g__Paracoccus | 0 | 0 | 0 | 0 | 0 | 0 | 0 | 0 | 0 | 0 | 0 | 0 | 4 | 7 | 0 | 0 | 37 | 29 |
| g__Paramuribaculum | 2 | 3 | 5 | 6 | 6 | 4 | 60 | 28 | 81 | 63 | 13 | 35 | 5 | 13 | 129 | 0 | 6 | 1 |
| g__Paraprevotella | 0 | 0 | 1 | 7 | 2 | 1 | 0 | 0 | 0 | 0 | 0 | 0 | 0 | 0 | 0 | 0 | 0 | 0 |
| g__Parasutterella | 43 | 192 | 371 | 199 | 153 | 36 | 0 | 0 | 0 | 2 | 0 | 1 | 1 | 1 | 0 | 0 | 0 | 0 |
| g__Pectobacterium | 0 | 2 | 2 | 0 | 0 | 2 | 0 | 0 | 2 | 5 | 2 | 0 | 0 | 1 | 0 | 1 | 1 | 0 |
| g__Pediococcus | 0 | 0 | 0 | 0 | 0 | 0 | 0 | 0 | 0 | 0 | 0 | 0 | 0 | 0 | 0 | 0 | 19 | 5 |
| g__Pedobacter | 0 | 0 | 0 | 0 | 0 | 0 | 2 | 10 | 2 | 31 | 13 | 7 | 0 | 0 | 5 | 0 | 0 | 0 |
| g__Pelagibacterium | 0 | 0 | 0 | 0 | 0 | 0 | 0 | 0 | 0 | 0 | 0 | 0 | 2 | 1 | 0 | 0 | 3 | 1 |
| g__Phascolarctobacterium | 10 | 5 | 15 | 2 | 9 | 23 | 7 | 76 | 89 | 33 | 48 | 242 | 7 | 20 | 71 | 0 | 7 | 2 |
| g__Phycicoccus | 0 | 0 | 0 | 0 | 0 | 0 | 0 | 1 | 0 | 0 | 0 | 0 | 1 | 0 | 1 | 0 | 9 | 0 |
| g__Phyllobacterium | 0 | 1 | 1 | 2 | 0 | 1 | 1 | 0 | 0 | 1 | 0 | 1 | 2 | 1 | 0 | 0 | 23 | 2 |
| g__Prevotella | 1765 | 61 | 427 | 346 | 406 | 91 | 8932 | 699 | 603 | 2028 | 3558 | 4224 | 144 | 611 | 397 | 0 | 149 | 54 |
| g__Prevotellamassilia | 103 | 24 | 41 | 302 | 130 | 4 | 45 | 17 | 46 | 116 | 53 | 108 | 6 | 17 | 39 | 0 | 2 | 2 |
| g__Propionicicella | 0 | 0 | 0 | 0 | 0 | 1 | 0 | 1 | 0 | 1 | 1 | 1 | 0 | 0 | 0 | 0 | 2 | 0 |
| g__Proteus | 4 | 12 | 18 | 3 | 10 | 11 | 5 | 11 | 10 | 11 | 12 | 13 | 6 | 15 | 12 | 4 | 20 | 4 |
| g__Provencibacterium | 19 | 51 | 20 | 49 | 101 | 73 | 8 | 16 | 32 | 28 | 26 | 15 | 1 | 0 | 36 | 0 | 3 | 1 |
| g__Providencia | 24 | 40 | 47 | 14 | 34 | 41 | 20 | 47 | 52 | 45 | 60 | 44 | 34 | 24 | 34 | 17 | 77 | 23 |
| g__Pseudochrobactrum | 0 | 0 | 0 | 0 | 0 | 0 | 0 | 0 | 0 | 0 | 0 | 0 | 0 | 0 | 0 | 0 | 3 | 0 |
| g__Pseudoflavonifractor | 4 | 4 | 6 | 4 | 10 | 40 | 1 | 0 | 4 | 1 | 1 | 5 | 0 | 0 | 1 | 0 | 0 | 0 |
| g__Pseudomonas_A | 0 | 0 | 0 | 0 | 0 | 0 | 0 | 0 | 0 | 0 | 0 | 0 | 0 | 6 | 0 | 0 | 9 | 14 |
| g__Pseudomonas_E | 0 | 2 | 1 | 2 | 3 | 1 | 1 | 10 | 7 | 1 | 13 | 6 | 1 | 2 | 4 | 2 | 3876 | 2 |
| g__Psychrobacter | 0 | 0 | 0 | 0 | 1 | 0 | 8 | 1 | 0 | 2 | 0 | 0 | 2 | 14 | 0 | 3 | 30 | 21 |
| g__Pusillimonas | 0 | 0 | 0 | 0 | 0 | 0 | 0 | 0 | 0 | 0 | 0 | 0 | 3 | 6 | 0 | 2 | 24 | 23 |
| g__QAMH01 | 5 | 8 | 10 | 0 | 9 | 3 | 0 | 7 | 0 | 3 | 0 | 2 | 2 | 2 | 1 | 0 | 0 | 0 |
| g__QKVK01 | 0 | 0 | 1 | 1 | 2 | 0 | 0 | 0 | 3 | 0 | 2 | 2 | 0 | 0 | 0 | 0 | 1 | 1 |
| g__RC9 | 0 | 1 | 0 | 0 | 1 | 1 | 138 | 64 | 130 | 63 | 45 | 200 | 5 | 13 | 128 | 0 | 8 | 1 |
| g__RUG754 | 6 | 1 | 4 | 1 | 12 | 2 | 0 | 13 | 10 | 11 | 4 | 1 | 0 | 0 | 5 | 0 | 0 | 0 |
| g__Ralstonia | 0 | 0 | 0 | 0 | 0 | 0 | 0 | 0 | 0 | 0 | 1 | 0 | 0 | 6 | 0 | 0 | 9 | 3 |
| g__Raoultibacter | 11 | 9 | 3 | 10 | 7 | 8 | 8 | 18 | 15 | 7 | 9 | 9 | 1 | 18 | 16 | 0 | 0 | 0 |
| g__Reyranella | 1 | 0 | 0 | 0 | 1 | 0 | 0 | 0 | 0 | 0 | 1 | 0 | 2 | 3 | 0 | 0 | 12 | 0 |
| g__Rhizorhabdus | 3 | 4 | 2 | 5 | 2 | 5 | 2 | 6 | 4 | 2 | 5 | 3 | 1 | 3 | 2 | 3 | 4 | 1 |
| g__Rikenella | 15 | 5 | 5 | 25 | 8 | 15 | 17 | 87 | 187 | 37 | 45 | 356 | 16 | 18 | 138 | 0 | 6 | 5 |
| g__Robinsoniella | 0 | 0 | 0 | 1 | 56 | 16 | 3 | 12 | 5 | 13 | 5 | 0 | 1 | 1 | 4 | 0 | 1 | 0 |
| g__Rodentibacter | 0 | 0 | 5 | 1 | 3 | 0 | 13 | 11 | 3 | 4 | 3 | 13 | 2 | 4 | 4 | 0 | 1 | 0 |
| g__Romboutsia | 45 | 21 | 229 | 19 | 42 | 16 | 0 | 0 | 5 | 5 | 10 | 4 | 19 | 10 | 3 | 3 | 78 | 9 |
| g__Roseburia | 0 | 1 | 8 | 0 | 0 | 25 | 3 | 20 | 13 | 44 | 107 | 29 | 0 | 1 | 16 | 0 | 1 | 1 |
| g__Rothia | 28 | 16 | 39 | 15 | 34 | 24 | 3 | 31 | 14 | 20 | 16 | 21 | 2503 | 1992 | 20 | 273 | 12142 | 7471 |
| g__Ruminiclostridium | 1 | 1 | 5 | 1 | 0 | 0 | 0 | 0 | 4 | 0 | 0 | 1 | 0 | 0 | 1 | 0 | 1 | 0 |
| g__Ruminiclostridium_A | 111 | 1 | 4 | 0 | 1 | 10 | 665 | 24 | 16 | 2 | 1 | 111 | 5 | 51 | 34 | 0 | 0 | 0 |
| g__Ruminiclostridium_C | 7 | 20 | 25 | 19 | 26 | 26 | 39 | 29 | 29 | 46 | 67 | 36 | 5 | 10 | 22 | 0 | 0 | 3 |
| g__Ruminiclostridium_D | 0 | 0 | 0 | 0 | 0 | 0 | 0 | 0 | 0 | 0 | 0 | 0 | 0 | 0 | 0 | 0 | 10 | 0 |
| g__Ruminiclostridium_E | 25 | 44 | 138 | 6 | 370 | 113 | 74 | 384 | 698 | 56 | 1082 | 248 | 4 | 7 | 681 | 0 | 44 | 15 |
| g__Ruminococcus | 4 | 2 | 7 | 0 | 14 | 3 | 38 | 15 | 35 | 92 | 352 | 225 | 2 | 1 | 37 | 0 | 6 | 3 |
| g__Ruminococcus_A | 2 | 1015 | 160 | 6 | 77 | 26 | 5 | 10 | 10 | 36 | 45 | 22 | 1 | 3 | 14 | 0 | 1 | 1 |
| g__Ruminococcus_C | 170 | 35 | 57 | 10 | 35 | 13 | 87 | 88 | 57 | 22 | 87 | 271 | 4 | 12 | 56 | 0 | 7 | 1 |
| g__Ruminococcus_D | 75 | 19 | 6 | 3 | 3 | 107 | 6 | 22 | 67 | 11 | 77 | 109 | 0 | 2 | 71 | 0 | 2 | 3 |
| g__Ruthenibacterium | 11 | 23 | 9 | 9 | 5 | 113 | 2 | 52 | 16 | 14 | 8 | 13 | 0 | 2 | 13 | 0 | 2 | 2 |
| g__SK-Y3 | 3 | 0 | 1 | 2 | 5 | 7 | 5 | 4 | 12 | 1 | 7 | 5 | 2 | 2 | 7 | 0 | 1 | 0 |
| g__SR-FBR-E99 | 3 | 3 | 5 | 2 | 5 | 5 | 2 | 0 | 4 | 2 | 3 | 2 | 1 | 1 | 1 | 1 | 0 | 0 |
| g__SZUA-55 | 0 | 0 | 0 | 0 | 0 | 0 | 0 | 0 | 0 | 0 | 0 | 0 | 8 | 1 | 0 | 0 | 1 | 6 |
| g__Saccharimonas | 171 | 22 | 81 | 38 | 37 | 78 | 720 | 1100 | 1508 | 625 | 1043 | 1290 | 155 | 543 | 1300 | 9 | 87 | 72 |
| g__Salmonella | 2307 | 765 | 1294 | 663 | 1872 | 2016 | 944 | 1109 | 1448 | 1331 | 1271 | 1378 | 96 | 317 | 1143 | 9 | 181 | 82 |
| g__Savagella | 4 | 0 | 4 | 0 | 0 | 0 | 0 | 1 | 0 | 1 | 0 | 0 | 0 | 0 | 1 | 0 | 0 | 0 |
| g__Schwartzia | 1 | 4 | 0 | 1 | 2 | 4 | 341 | 2197 | 617 | 1461 | 1392 | 565 | 76 | 234 | 316 | 3 | 54 | 20 |
| g__Serinibacter | 3 | 1 | 0 | 1 | 1 | 1 | 0 | 2 | 3 | 1 | 5 | 4 | 1 | 0 | 2 | 2 | 3 | 2 |
| g__Solobacterium | 0 | 0 | 0 | 0 | 0 | 0 | 0 | 0 | 0 | 0 | 0 | 0 | 0 | 0 | 0 | 0 | 8 | 0 |
| g__Sphingobacterium | 1 | 4 | 5 | 3 | 8 | 6 | 24 | 10 | 33 | 5 | 9 | 12 | 2 | 22 | 23 | 2 | 48 | 66 |
| g__Sphingomonas_A | 9 | 12 | 9 | 8 | 8 | 21 | 8 | 21 | 11 | 23 | 23 | 22 | 4 | 12 | 13 | 3 | 30 | 8 |
| g__Sphingomonas_B | 3 | 3 | 2 | 1 | 0 | 2 | 1 | 1 | 2 | 5 | 4 | 3 | 1 | 1 | 2 | 1 | 3 | 0 |
| g__Sphingopyxis | 0 | 1 | 0 | 0 | 0 | 0 | 0 | 0 | 0 | 0 | 0 | 0 | 0 | 0 | 0 | 0 | 94 | 0 |
| g__Sporobacter | 9 | 22 | 10 | 3 | 12 | 13 | 14 | 23 | 19 | 11 | 13 | 22 | 2 | 7 | 23 | 0 | 1 | 2 |
| g__Sporolactobacillus | 0 | 0 | 0 | 0 | 0 | 0 | 0 | 0 | 0 | 0 | 1 | 0 | 0 | 0 | 0 | 0 | 17 | 0 |
| g__Staphylococcus | 8 | 3 | 3 | 0 | 5 | 0 | 0 | 0 | 0 | 0 | 0 | 0 | 5 | 0 | 0 | 0 | 15 | 1 |
| g__Staphylococcus_A | 1 | 0 | 0 | 1 | 0 | 3 | 0 | 0 | 0 | 0 | 0 | 0 | 38 | 5 | 0 | 1 | 88 | 10 |
| g__Steroidobacter | 0 | 1 | 2 | 0 | 2 | 0 | 0 | 1 | 0 | 0 | 1 | 3 | 0 | 2 | 1 | 0 | 0 | 0 |
| g__Stoquefichus | 15 | 7 | 6 | 1 | 10 | 24 | 0 | 1 | 0 | 0 | 0 | 0 | 0 | 0 | 0 | 0 | 0 | 0 |
| g__Streptococcus | 7 | 2 | 12 | 0 | 1 | 2 | 174 | 42 | 103 | 59 | 61 | 99 | 434 | 159 | 175 | 65 | 1059 | 580 |
| g__Sutterella | 37 | 30 | 45 | 3 | 4 | 52 | 76 | 289 | 78 | 40 | 18 | 211 | 5 | 8 | 62 | 0 | 5 | 4 |
| g__Symbiobacterium | 0 | 3 | 1 | 3 | 1 | 2 | 2 | 4 | 1 | 2 | 1 | 2 | 0 | 0 | 1 | 2 | 3 | 1 |
| g__Syntrophobacter | 2 | 6 | 3 | 2 | 2 | 5 | 4 | 3 | 1 | 4 | 6 | 5 | 2 | 5 | 2 | 2 | 10 | 1 |
| g__T78 | 4 | 1 | 0 | 1 | 0 | 0 | 0 | 2 | 0 | 4 | 1 | 0 | 1 | 1 | 1 | 0 | 4 | 2 |
| g__TF01-11 | 1 | 9 | 2 | 3 | 10 | 18 | 5 | 24 | 60 | 28 | 69 | 25 | 3 | 3 | 29 | 0 | 3 | 2 |
| g__TWA4 | 2 | 25 | 6 | 5 | 9 | 7 | 0 | 9 | 5 | 9 | 10 | 2 | 0 | 0 | 4 | 0 | 1 | 0 |
| g__Tatlockia | 0 | 0 | 0 | 0 | 1 | 0 | 0 | 0 | 0 | 0 | 0 | 1 | 1 | 0 | 0 | 0 | 236 | 1 |
| g__Thermoflavifilum | 1 | 0 | 5 | 3 | 3 | 0 | 1 | 2 | 0 | 3 | 1 | 1 | 0 | 1 | 0 | 0 | 1 | 1 |
| g__Thioalkalivibrio_A | 0 | 1 | 1 | 2 | 4 | 6 | 2 | 7 | 4 | 2 | 4 | 2 | 0 | 0 | 1 | 2 | 3 | 0 |
| g__Thiobacillus | 1 | 0 | 0 | 1 | 1 | 0 | 0 | 0 | 0 | 0 | 0 | 2 | 0 | 0 | 1 | 0 | 0 | 2 |
| g__Tissierella_A | 0 | 0 | 0 | 0 | 1 | 0 | 0 | 0 | 0 | 0 | 0 | 0 | 0 | 1 | 0 | 0 | 0 | 1 |
| g__Tolypothrix_B | 0 | 0 | 0 | 0 | 1 | 3 | 0 | 1 | 2 | 2 | 0 | 1 | 1 | 0 | 1 | 0 | 1 | 0 |
| g__Treponema_D | 5 | 9 | 22 | 16 | 598 | 1157 | 110 | 845 | 2983 | 1517 | 655 | 1359 | 15 | 45 | 1202 | 1 | 22 | 8 |
| g__Tyzzerella | 4 | 1 | 8 | 1 | 15 | 4 | 9 | 2 | 29 | 13 | 18 | 38 | 2 | 7 | 55 | 0 | 4 | 0 |
| g__UBA1033 | 2 | 3 | 1 | 2 | 4 | 1 | 0 | 0 | 0 | 0 | 0 | 0 | 0 | 0 | 0 | 0 | 0 | 0 |
| g__UBA1174 | 0 | 0 | 1 | 4 | 1 | 1 | 0 | 0 | 0 | 1 | 0 | 1 | 0 | 0 | 0 | 0 | 0 | 0 |
| g__UBA1191 | 3 | 9 | 8 | 0 | 8 | 16 | 4 | 11 | 21 | 11 | 12 | 18 | 12 | 13 | 10 | 0 | 2 | 3 |
| g__UBA12465 | 0 | 0 | 0 | 0 | 0 | 1 | 3 | 1 | 0 | 0 | 0 | 1 | 0 | 0 | 0 | 0 | 0 | 1 |
| g__UBA1394 | 41 | 90 | 1 | 0 | 2 | 0 | 11 | 47 | 76 | 51 | 16 | 64 | 1 | 0 | 127 | 0 | 4 | 2 |
| g__UBA1436 | 1 | 0 | 1 | 5 | 0 | 8 | 2 | 0 | 0 | 0 | 0 | 5 | 1 | 0 | 1 | 0 | 2 | 0 |
| g__UBA1547 | 0 | 0 | 0 | 0 | 0 | 0 | 141 | 138 | 84 | 23 | 121 | 148 | 19 | 80 | 66 | 1 | 5 | 6 |
| g__UBA1711 | 0 | 0 | 1 | 0 | 0 | 0 | 92 | 16 | 15 | 63 | 44 | 24 | 3 | 5 | 25 | 0 | 1 | 0 |
| g__UBA1777 | 0 | 1 | 1 | 0 | 1 | 0 | 96 | 61 | 41 | 69 | 67 | 54 | 7 | 18 | 46 | 0 | 1 | 0 |
| g__UBA2212 | 22 | 12 | 53 | 15 | 2 | 14 | 4 | 2 | 6 | 2 | 0 | 3 | 0 | 3 | 4 | 0 | 0 | 1 |
| g__UBA2365 | 0 | 0 | 0 | 0 | 0 | 0 | 0 | 0 | 0 | 0 | 0 | 0 | 0 | 0 | 0 | 0 | 12 | 0 |
| g__UBA2730 | 53 | 30 | 78 | 4 | 3 | 163 | 113 | 78 | 117 | 6 | 0 | 239 | 23 | 139 | 82 | 0 | 4 | 3 |
| g__UBA3006 | 2 | 2 | 1 | 1 | 2 | 2 | 2 | 3 | 1 | 1 | 2 | 2 | 8 | 2 | 0 | 1 | 7 | 2 |
| g__UBA4782 | 1 | 7 | 5 | 3 | 3 | 2 | 1 | 4 | 6 | 4 | 5 | 4 | 3 | 6 | 1 | 6 | 4 | 2 |
| g__UBA6382 | 0 | 0 | 0 | 0 | 0 | 0 | 0 | 0 | 0 | 0 | 0 | 0 | 0 | 0 | 0 | 0 | 24 | 0 |
| g__UBA6398 | 0 | 0 | 0 | 0 | 13 | 2 | 0 | 0 | 0 | 0 | 0 | 0 | 0 | 0 | 0 | 0 | 0 | 0 |
| g__UBA6659 | 1 | 1 | 2 | 0 | 2 | 1 | 0 | 1 | 0 | 0 | 1 | 0 | 1 | 0 | 0 | 0 | 0 | 1 |
| g__UBA6985 | 44 | 0 | 101 | 0 | 2 | 3 | 115 | 33 | 56 | 58 | 10 | 55 | 4 | 11 | 46 | 2 | 0 | 1 |
| g__UBA7182 | 0 | 0 | 0 | 0 | 0 | 0 | 0 | 0 | 1 | 1 | 0 | 1 | 0 | 0 | 0 | 0 | 0 | 0 |
| g__UBA8950 | 1 | 0 | 1 | 0 | 0 | 1 | 0 | 0 | 0 | 2 | 1 | 3 | 1 | 1 | 0 | 1 | 0 | 0 |
| g__UBA945 | 0 | 0 | 0 | 0 | 0 | 0 | 0 | 3 | 13 | 1 | 8 | 21 | 0 | 3 | 8 | 0 | 2 | 0 |
| g__UC5-1-2E3 | 3 | 15 | 8 | 9 | 120 | 144 | 3 | 21 | 29 | 56 | 34 | 18 | 0 | 12 | 15 | 0 | 4 | 5 |
| g__URHD0088 | 0 | 0 | 0 | 1 | 0 | 0 | 0 | 1 | 0 | 2 | 0 | 2 | 0 | 1 | 0 | 0 | 0 | 0 |
| g__UTCFX2 | 3 | 4 | 3 | 2 | 1 | 4 | 0 | 4 | 3 | 1 | 7 | 6 | 4 | 7 | 3 | 3 | 6 | 3 |
| g__Vagococcus | 7 | 1 | 0 | 0 | 4 | 1 | 4 | 1 | 2 | 0 | 1 | 1 | 5 | 3 | 1 | 6 | 3 | 7 |
| g__Vallitalea | 127 | 93 | 199 | 2 | 71 | 105 | 27 | 3 | 2 | 0 | 2 | 19 | 6 | 14 | 0 | 0 | 1 | 1 |
| g__Vallitalea_A | 5 | 14 | 2 | 3 | 7 | 1 | 0 | 0 | 0 | 1 | 0 | 0 | 0 | 0 | 0 | 0 | 0 | 0 |
| g__Veillonella | 0 | 0 | 0 | 0 | 0 | 0 | 4 | 0 | 1 | 2 | 0 | 4 | 0 | 1 | 0 | 0 | 0 | 0 |
| g__Vibrio | 10022 | 5347 | 10250 | 8790 | 8905 | 12466 | 4103 | 1338 | 1759 | 1563 | 2173 | 1783 | 167 | 432 | 1955 | 3 | 99 | 65 |
| g__Virgibacillus | 0 | 0 | 0 | 0 | 0 | 0 | 0 | 0 | 0 | 0 | 0 | 0 | 8 | 26 | 0 | 1 | 65 | 30 |
| g__Virgibacillus_G | 3 | 33 | 33 | 21 | 616 | 177 | 4 | 130 | 22 | 156 | 47 | 17 | 1 | 4 | 23 | 0 | 3 | 2 |
| g__W-Firmicutes-11 | 7 | 27 | 17 | 13 | 17 | 20 | 14 | 39 | 27 | 31 | 23 | 29 | 2 | 8 | 23 | 0 | 2 | 3 |
| g__Weissella | 6 | 6 | 4 | 5 | 3 | 2 | 6 | 5 | 9 | 10 | 3 | 9 | 19 | 13 | 6 | 18 | 12 | 8 |
| g__Yaniella | 0 | 0 | 0 | 0 | 0 | 0 | 0 | 0 | 0 | 0 | 0 | 1 | 3 | 0 | 0 | 0 | 17 | 11 |
| g__ZCTH02-B6 | 0 | 0 | 0 | 0 | 0 | 0 | 0 | 0 | 0 | 0 | 0 | 0 | 1 | 0 | 1 | 0 | 0 | 0 |
| g__Zag1 | 0 | 1 | 0 | 0 | 1 | 0 | 68 | 24 | 5 | 9 | 25 | 26 | 1 | 17 | 20 | 0 | 3 | 0 |
| g__Zag111 | 0 | 0 | 0 | 0 | 0 | 1 | 1 | 2 | 1 | 3 | 4 | 1 | 0 | 2 | 3 | 0 | 0 | 0 |
| g__unclassified | 2 | 0 | 0 | 0 | 14 | 0 | 0 | 2 | 2 | 2 | 2 | 1 | 4 | 0 | 1 | 4 | 7 | 4 |

# S8

## S8.1（Fig8A）

| phylum | WT | Uox-/--1 | Uox-/--2 | SE | WT | Uox-/--1 | Uox-/--2 | total (top10) | |  |  | T-test Uox-/--2 vs Uox-/--1 |
| --- | --- | --- | --- | --- | --- | --- | --- | --- | --- | --- | --- | --- |
| Proteobacteria | 58.74408 | 28.08089 | 11.3991 |  | 1.635226 | 1.422547 | 4.140086 | 99.74879 | 99.64764 | 99.78113 |  | 6.13E-08 |
| Firmicutes | 16.77942 | 24.03958 | 66.81323 |  | 1.607899 | 2.527577 | 11.25683 |  |  |  |  | 0.035843 |
| Firmicutes_A | 13.5732 | 23.90685 | 5.094902 |  | 1.95954 | 2.971243 | 2.963863 |  |  |  |  | 0.015743 |
| Bacteroidota | 7.168941 | 12.35573 | 1.644758 |  | 0.734736 | 3.656615 | 0.760011 |  |  |  |  | 0.194495 |
| Actinobacteriota | 1.119817 | 0.349101 | 12.21828 |  | 0.726475 | 0.053773 | 6.505155 |  |  |  |  | 0.314942 |
| Firmicutes_C | 0.838491 | 3.223519 | 0.611318 |  | 0.371461 | 0.765917 | 0.252294 |  |  |  |  | 0.018736 |
| Spirochaetota | 0.734554 | 3.519425 | 0.652139 |  | 0.467528 | 1.264475 | 0.596538 |  |  |  |  | 0.065754 |
| Desulfobacterota_A | 0.353885 | 0.729897 | 0.174103 |  | 0.139777 | 0.219614 | 0.12978 |  |  |  |  | 0.179216 |
| Campylobacterota | 0.252004 | 0.210258 | 0.046328 |  | 0.10128 | 0.109917 | 0.03685 |  |  |  |  | 0.785698 |
| Patescibacteria | 0.1844 | 3.232401 | 1.126977 |  | 0.055072 | 0.478909 | 0.64803 |  |  |  |  | 8.65E-05 |
| \| Chloroflexota \| \| --- \| | 0.046758 | 0.060426 | 0.053615 |  | 0.004218 | 0.009454 | 0.012236 |  |  |  |  | 0.216185 |
| Nitrospirota | 0.043982 | 0.079006 | 0.034802 |  | 0.005419 | 0.009639 | 0.013013 |  |  |  |  | 0.010031 |
| Euryarchaeota | 0.039878 | 0.015862 | 0.000286 |  | 0.015722 | 0.011457 | 0.000286 |  |  |  |  | 0.24523 |
| Firmicutes_B | 0.026511 | 0.006959 | 0 |  | 0.010586 | 0.003245 | 0 |  |  |  |  | 0.10786 |
| Cyanobacteria | 0.023879 | 0.111861 | 0.0411 |  | 0.004395 | 0.026387 | 0.018358 |  |  |  |  | 0.008165 |
| Deinococcota | 0.018026 | 0.027453 | 0.018351 |  | 0.002118 | 0.002238 | 0.005908 |  |  |  |  | 0.012058 |
| Elusimicrobiota | 0.009917 | 0.004127 | 0.002013 |  | 0.004748 | 0.002659 | 0.00103 |  |  |  |  | 0.312259 |
| Desulfobacterota | 0.008671 | 0.010288 | 0.010502 |  | 0.001758 | 0.001745 | 0.004447 |  |  |  |  | 0.528543 |
| unclassified | 0.006844 | 0.004101 | 0.008794 |  | 0.00589 | 0.000948 | 0.003124 |  |  |  |  | 0.655516 |
| Firmicutes_K | 0.006334 | 0.005629 | 0.008246 |  | 0.001965 | 0.002425 | 0.002877 |  |  |  |  | 0.825698 |
| MBNT15 | 0.005442 | 0.00768 | 0.002853 |  | 0.001667 | 0.001109 | 0.000787 |  |  |  |  | 0.289827 |
| Firmicutes_E | 0.004525 | 0.005514 | 0.003169 |  | 0.001435 | 0.001275 | 0.001459 |  |  |  |  | 0.617784 |
| Thermotogota | 0.004094 | 0.007004 | 0 |  | 0.001292 | 0.005414 | 0 |  |  |  |  | 0.612489 |
| Armatimonadota | 0.003005 | 0.000889 | 0.000752 |  | 0.000816 | 0.000564 | 0.000496 |  |  |  |  | 0.058682 |
| Acidobacteriota | 0.001271 | 0.002275 | 0.000953 |  | 0.000888 | 0.00107 | 0.000604 |  |  |  |  | 0.486756 |
| Fermentibacterota | 0.001253 | 0.001877 | 0.00311 |  | 0.000562 | 0.000944 | 0.001191 |  |  |  |  | 0.582721 |
| Halobacterota | 0.000821 | 0.000889 | 0.001219 |  | 0.00052 | 0.000564 | 0.00092 |  |  |  |  | 0.931089 |
| Desulfuromonadota | 0 | 0 | 0.00282 |  | 0 | 0 | 0.001992 |  |  |  |  | #DIV/0! |
| Firmicutes_G | 0 | 0 | 0.000971 |  | 0 | 0 | 0.000614 |  |  |  |  | #DIV/0! |
| Firmicutes_I | 0 | 0.000516 | 0.017331 |  | 0 | 0.000516 | 0.01225 |  |  |  |  | 0.340893 |
| Planctomycetota | 0 | 0 | 0.001564 |  | 0 | 0 | 0.001564 |  |  |  |  | #DIV/0! |
| Verrucomicrobiota | 0 | 0 | 0.006418 |  | 0 | 0 | 0.003547 |  |  |  |  | #DIV/0! |

## S8.2（Fig 8B）

| class | WT | Uox-/--1 | Uox-/--2 | SE | WT | Uox-/--1 | Uox-/--2 | total (top10) | |  |  | T-test Uox-/--2 vs Uox-/--1 |
| --- | --- | --- | --- | --- | --- | --- | --- | --- | --- | --- | --- | --- |
| Gammaproteobacteria | 58.68198 | 27.97204 | 10.46734 |  | 1.634296 | 1.421499 | 3.577141 | 99.49797 | 96.30135 | 97.70792 |  | 6E-08 |
| Bacilli | 16.77942 | 24.03958 | 66.81323 |  | 1.607899 | 2.527577 | 11.25683 |  |  |  |  | 0.035843 |
| Clostridia | 13.5732 | 23.90685 | 5.094902 |  | 1.95954 | 2.971243 | 2.963863 |  |  |  |  | 0.015743 |
| Bacteroidia | 7.168941 | 12.35573 | 1.644758 |  | 0.734736 | 3.656615 | 0.760011 |  |  |  |  | 0.194495 |
| Actinobacteria | 0.841442 | 0.130028 | 12.11287 |  | 0.683429 | 0.020169 | 6.5155 |  |  |  |  | 0.322613 |
| Negativicutes | 0.838491 | 3.223519 | 0.611318 |  | 0.371461 | 0.765917 | 0.252294 |  |  |  |  | 0.018736 |
| Spirochaetia | 0.734554 | 3.519425 | 0.646926 |  | 0.467528 | 1.264475 | 0.597525 |  |  |  |  | 0.065754 |
| Desulfovibrionia | 0.353885 | 0.729897 | 0.174103 |  | 0.139777 | 0.219614 | 0.12978 |  |  |  |  | 0.179216 |
| Coriobacteriia | 0.274049 | 0.214026 | 0.096144 |  | 0.047012 | 0.034849 | 0.043714 |  |  |  |  | 0.32922 |
| Campylobacteria | 0.252004 | 0.210258 | 0.046328 |  | 0.10128 | 0.109917 | 0.03685 |  |  |  |  | 0.785698 |
| \| Saccharimonadia \| \| --- \| | 0.184007 | 3.230053 | 1.126691 |  | 0.055071 | 0.479178 | 0.648118 |  |  |  |  | 8.73E-05 |
| Alphaproteobacteria | 0.062095 | 0.108846 | 0.931761 |  | 0.004077 | 0.004147 | 0.7915 |  |  |  |  | 1.13E-05 |
| Anaerolineae | 0.046758 | 0.060426 | 0.053615 |  | 0.004218 | 0.009454 | 0.012236 |  |  |  |  | 0.216185 |
| Thermodesulfovibrionia | 0.043982 | 0.079006 | 0.034802 |  | 0.005419 | 0.009639 | 0.013013 |  |  |  |  | 0.010031 |
| Methanobacteria | 0.039878 | 0.015862 | 0.000286 |  | 0.015722 | 0.011457 | 0.000286 |  |  |  |  | 0.24523 |
| Vampirovibrionia | 0.018735 | 0.102497 | 0.033563 |  | 0.004629 | 0.027532 | 0.017021 |  |  |  |  | 0.013339 |
| Deinococci | 0.018026 | 0.027453 | 0.018351 |  | 0.002118 | 0.002238 | 0.005908 |  |  |  |  | 0.012058 |
| Peptococcia | 0.014419 | 0.002359 | 0 |  | 0.009557 | 0.002359 | 0 |  |  |  |  | 0.248595 |
| Dehalobacteriia | 0.011663 | 0.003205 | 0 |  | 0.004794 | 0.001117 | 0 |  |  |  |  | 0.116475 |
| Elusimicrobia | 0.009917 | 0.004127 | 0.002013 |  | 0.004748 | 0.002659 | 0.00103 |  |  |  |  | 0.312259 |
| Syntrophobacteria | 0.008671 | 0.010288 | 0.010502 |  | 0.001758 | 0.001745 | 0.004447 |  |  |  |  | 0.528543 |
| unclassified | 0.006844 | 0.004101 | 0.008794 |  | 0.00589 | 0.000948 | 0.003124 |  |  |  |  | 0.655516 |
| Alicyclobacillia | 0.006334 | 0.005629 | 0.008246 |  | 0.001965 | 0.002425 | 0.002877 |  |  |  |  | 0.825698 |
| MBNT15 | 0.005442 | 0.00768 | 0.002853 |  | 0.001667 | 0.001109 | 0.000787 |  |  |  |  | 0.289827 |
| Cyanobacteriia | 0.005145 | 0.009364 | 0.007537 |  | 0.001212 | 0.002745 | 0.001876 |  |  |  |  | 0.19005 |
| Symbiobacteriia | 0.004525 | 0.005514 | 0.003169 |  | 0.001435 | 0.001275 | 0.001459 |  |  |  |  | 0.617784 |
| Acidimicrobiia | 0.004326 | 0.005047 | 0.009261 |  | 0.000464 | 0.000872 | 0.004138 |  |  |  |  | 0.482517 |
| Thermotogae | 0.004094 | 0.007004 | 0 |  | 0.001292 | 0.005414 | 0 |  |  |  |  | 0.612489 |
| Fimbriimonadia | 0.003005 | 0.000889 | 0.000752 |  | 0.000816 | 0.000564 | 0.000496 |  |  |  |  | 0.058682 |
| Fermentibacteria | 0.001253 | 0.001877 | 0.00311 |  | 0.000562 | 0.000944 | 0.001191 |  |  |  |  | 0.582721 |
| Aminicenantia | 0.000832 | 0.002275 | 0.000449 |  | 0.000527 | 0.00107 | 0.000449 |  |  |  |  | 0.254162 |
| Methanosarcinia | 0.000821 | 0.000889 | 0.001219 |  | 0.00052 | 0.000564 | 0.00092 |  |  |  |  | 0.931089 |
| Acidobacteriae | 0.000439 | 0 | 0.000504 |  | 0.000439 | 0 | 0.000504 |  |  |  |  | 0.340893 |
| Desulfitobacteriia | 0.000429 | 0.001395 | 0 |  | 0.000429 | 0.000949 | 0 |  |  |  |  | 0.375273 |
| ABY1 | 0.000392 | 0.002348 | 0.000286 |  | 0.000392 | 0.00135 | 0.000286 |  |  |  |  | 0.194464 |
| Bacilli_A | 0 | 0.000516 | 0.017331 |  | 0 | 0.000516 | 0.01225 |  |  |  |  | 0.340893 |
| Brachyspirae | 0 | 0 | 0.005213 |  | 0 | 0 | 0.005213 |  |  |  |  | #DIV/0! |
| Desulfuromonadia | 0 | 0 | 0.00282 |  | 0 | 0 | 0.001992 |  |  |  |  | #DIV/0! |
| Limnochordia | 0 | 0 | 0.000971 |  | 0 | 0 | 0.000614 |  |  |  |  | #DIV/0! |
| Planctomycetes | 0 | 0 | 0.001564 |  | 0 | 0 | 0.001564 |  |  |  |  | #DIV/0! |
| Verrucomicrobiae | 0 | 0 | 0.006418 |  | 0 | 0 | 0.003547 |  |  |  |  | #DIV/0! |

## S8.3 （Fig 8C）

| order | WT | Uox-/--1 | Uox-/--2 | SE | WT | Uox-/--1 | Uox-/--2 | total (top10) | |  |  | T-test Uox-/--2 vs Uox-/--1 |
| --- | --- | --- | --- | --- | --- | --- | --- | --- | --- | --- | --- | --- |
| Enterobacterales | 58.1384 | 27.59276 | 7.240945 |  | 1.586279 | 1.520156 | 2.742985 | 96.29423 | 91.61135 | 80.90001 |  | 7.24E-08 |
| Lactobacillales | 15.81347 | 23.25917 | 66.12538 |  | 1.673267 | 2.467608 | 11.49231 |  |  |  |  | 0.031589 |
| Lachnospirales | 7.105606 | 11.69686 | 2.075029 |  | 2.035535 | 2.356579 | 1.301405 |  |  |  |  | 0.171145 |
| Oscillospirales | 5.247111 | 9.886559 | 1.935136 |  | 0.285457 | 0.706098 | 1.228281 |  |  |  |  | 0.000117 |
| Bacteroidales | 5.144749 | 11.90582 | 1.483565 |  | 0.849277 | 3.696851 | 0.711946 |  |  |  |  | 0.105009 |
| Flavobacteriales | 2.0061 | 0.372911 | 0.080421 |  | 0.624336 | 0.13904 | 0.04878 |  |  |  |  | 0.0287 |
| Selenomonadales | 0.811037 | 2.987962 | 0.343555 |  | 0.368435 | 0.780771 | 0.15739 |  |  |  |  | 0.030308 |
| Treponematales | 0.734554 | 3.519425 | 0.646926 |  | 0.467528 | 1.264475 | 0.597525 |  |  |  |  | 0.065754 |
| Mycobacteriales | 0.721819 | 0.021363 | 0.789607 |  | 0.670395 | 0.001775 | 0.356122 |  |  |  |  | 0.320697 |
| Christensenellales | 0.571377 | 0.368515 | 0.179442 |  | 0.140131 | 0.051459 | 0.121445 |  |  |  |  | 0.204029 |
| \| Burkholderiales \| \| --- \| | 0.525399 | 0.338198 | 0.825853 |  | 0.138916 | 0.123693 | 0.519012 |  |  |  |  | 0.337939 |
| Bacillales | 0.380121 | 0.169741 | 0.213471 |  | 0.247712 | 0.068199 | 0.13392 |  |  |  |  | 0.431966 |
| Desulfovibrionales | 0.353885 | 0.729897 | 0.174103 |  | 0.139777 | 0.219614 | 0.12978 |  |  |  |  | 0.179216 |
| Peptostreptococcales | 0.330231 | 0.166012 | 0.108338 |  | 0.094778 | 0.019067 | 0.037994 |  |  |  |  | 0.120233 |
| Erysipelotrichales | 0.322489 | 0.098251 | 0.041286 |  | 0.110443 | 0.02313 | 0.016215 |  |  |  |  | 0.074965 |
| Coriobacteriales | 0.274049 | 0.214026 | 0.096144 |  | 0.047012 | 0.034849 | 0.043714 |  |  |  |  | 0.32922 |
| Campylobacterales | 0.252004 | 0.210258 | 0.046328 |  | 0.10128 | 0.109917 | 0.03685 |  |  |  |  | 0.785698 |
| RF39 | 0.231673 | 0.473402 | 0.182993 |  | 0.093664 | 0.138656 | 0.09937 |  |  |  |  | 0.17915 |
| Monoglobales | 0.224586 | 1.374627 | 0.543948 |  | 0.056949 | 0.277517 | 0.309308 |  |  |  |  | 0.002289 |
| Saccharimonadales | 0.184007 | 3.230053 | 1.126691 |  | 0.055071 | 0.479178 | 0.648118 |  |  |  |  | 8.73E-05 |
| Actinomycetales | 0.116978 | 0.105397 | 11.31761 |  | 0.017665 | 0.018263 | 6.168887 |  |  |  |  | 0.65826 |
| Acetivibrionales | 0.074602 | 0.391139 | 0.044351 |  | 0.045779 | 0.302542 | 0.025748 |  |  |  |  | 0.325278 |
| Thermodesulfovibrionales | 0.043982 | 0.079006 | 0.034802 |  | 0.005419 | 0.009639 | 0.013013 |  |  |  |  | 0.010031 |
| Sphingomonadales | 0.043413 | 0.065877 | 0.092272 |  | 0.005802 | 0.008112 | 0.063807 |  |  |  |  | 0.047983 |
| Methanobacteriales | 0.039878 | 0.015862 | 0.000286 |  | 0.015722 | 0.011457 | 0.000286 |  |  |  |  | 0.24523 |
| Anaerolineales | 0.03484 | 0.04618 | 0.043838 |  | 0.003596 | 0.006499 | 0.009738 |  |  |  |  | 0.157797 |
| Acidaminococcales | 0.027033 | 0.230478 | 0.052241 |  | 0.007199 | 0.095587 | 0.033399 |  |  |  |  | 0.059785 |
| Exiguobacterales | 0.021379 | 0.038087 | 0.049939 |  | 0.004476 | 0.003571 | 0.009826 |  |  |  |  | 0.015347 |
| Gastranaerophilales | 0.018735 | 0.102497 | 0.033563 |  | 0.004629 | 0.027532 | 0.017021 |  |  |  |  | 0.013339 |
| Deinococcales | 0.018026 | 0.027453 | 0.018351 |  | 0.002118 | 0.002238 | 0.005908 |  |  |  |  | 0.012058 |
| Peptococcales | 0.014419 | 0.002359 | 0 |  | 0.009557 | 0.002359 | 0 |  |  |  |  | 0.248595 |
| 4572-78 | 0.011919 | 0.014246 | 0.008734 |  | 0.003148 | 0.003432 | 0.002088 |  |  |  |  | 0.628047 |
| Sphingobacteriales | 0.011745 | 0.072632 | 0.069611 |  | 0.002442 | 0.009184 | 0.023801 |  |  |  |  | 7.76E-05 |
| Dehalobacteriales | 0.011663 | 0.003205 | 0 |  | 0.004794 | 0.001117 | 0 |  |  |  |  | 0.116475 |
| Staphylococcales | 0.010288 | 0 | 0.124572 |  | 0.002798 | 0 | 0.078445 |  |  |  |  | 0.004271 |
| Rhizobiales | 0.010237 | 0.018792 | 0.766884 |  | 0.00159 | 0.004281 | 0.690332 |  |  |  |  | 0.090524 |
| Elusimicrobiales | 0.009917 | 0.004127 | 0.002013 |  | 0.004748 | 0.002659 | 0.00103 |  |  |  |  | 0.312259 |
| Pseudomonadales | 0.009576 | 0.028616 | 2.247818 |  | 0.002747 | 0.004793 | 2.182414 |  |  |  |  | 0.006266 |
| Syntrophobacterales | 0.008671 | 0.010288 | 0.010502 |  | 0.001758 | 0.001745 | 0.004447 |  |  |  |  | 0.528543 |
| SK-Y3 | 0.007633 | 0.016087 | 0.00588 |  | 0.00252 | 0.004703 | 0.003217 |  |  |  |  | 0.144191 |
| RF32 | 0.007079 | 0.017769 | 0.001924 |  | 0.002618 | 0.011229 | 0.000991 |  |  |  |  | 0.375681 |
| unclassified | 0.006844 | 0.004101 | 0.008794 |  | 0.00589 | 0.000948 | 0.003124 |  |  |  |  | 0.655516 |
| Clostridiales | 0.006751 | 0.00307 | 0.20154 |  | 0.002316 | 0.001655 | 0.192447 |  |  |  |  | 0.225017 |
| Chitinophagales | 0.006347 | 0.004364 | 0.011162 |  | 0.002557 | 0.00177 | 0.01029 |  |  |  |  | 0.537921 |
| Ectothiorhodospirales | 0.005987 | 0.009731 | 0.002883 |  | 0.002182 | 0.002347 | 0.001541 |  |  |  |  | 0.26983 |
| MBNT15 | 0.005442 | 0.00768 | 0.002853 |  | 0.001667 | 0.001109 | 0.000787 |  |  |  |  | 0.289827 |
| Cyanobacteriales | 0.005145 | 0.009364 | 0.007537 |  | 0.001212 | 0.002745 | 0.001876 |  |  |  |  | 0.19005 |
| Tumebacillales | 0.005063 | 0.002304 | 0.00601 |  | 0.001694 | 0.001347 | 0.002355 |  |  |  |  | 0.231055 |
| Eubacteriales | 0.004872 | 0.003975 | 0.000504 |  | 0.002049 | 0.002178 | 0.000504 |  |  |  |  | 0.770289 |
| Symbiobacteriales | 0.004525 | 0.005514 | 0.003169 |  | 0.001435 | 0.001275 | 0.001459 |  |  |  |  | 0.617784 |
| Microtrichales | 0.004326 | 0.005047 | 0.009261 |  | 0.000464 | 0.000872 | 0.004138 |  |  |  |  | 0.482517 |
| Petrotogales | 0.004094 | 0.007004 | 0 |  | 0.001292 | 0.005414 | 0 |  |  |  |  | 0.612489 |
| Fimbriimonadales | 0.003005 | 0.000889 | 0.000752 |  | 0.000816 | 0.000564 | 0.000496 |  |  |  |  | 0.058682 |
| Streptosporangiales | 0.002252 | 0.001504 | 0.004615 |  | 0.000458 | 0.001046 | 0.002374 |  |  |  |  | 0.52722 |
| Steroidobacterales | 0.002191 | 0.002274 | 0.001402 |  | 0.001055 | 0.00132 | 0.000938 |  |  |  |  | 0.961845 |
| Alicyclobacillales | 0.001271 | 0.003325 | 0.002235 |  | 0.000888 | 0.001341 | 0.000779 |  |  |  |  | 0.230257 |
| Fermentibacterales | 0.001253 | 0.001877 | 0.00311 |  | 0.000562 | 0.000944 | 0.001191 |  |  |  |  | 0.582721 |
| Reyranellales | 0.00085 | 0.000418 | 0.008537 |  | 0.000538 | 0.000418 | 0.005968 |  |  |  |  | 0.53982 |
| Aminicenantales | 0.000832 | 0.002275 | 0.000449 |  | 0.000527 | 0.00107 | 0.000449 |  |  |  |  | 0.254162 |
| Methanotrichales | 0.000821 | 0.000889 | 0.001219 |  | 0.00052 | 0.000564 | 0.00092 |  |  |  |  | 0.931089 |
| Elsterales | 0.000516 | 0.002222 | 0.000449 |  | 0.000516 | 0.00106 | 0.000449 |  |  |  |  | 0.178314 |
| Bryobacterales | 0.000439 | 0 | 0.000504 |  | 0.000439 | 0 | 0.000504 |  |  |  |  | 0.340893 |
| Desulfitobacteriales | 0.000429 | 0.001395 | 0 |  | 0.000429 | 0.000949 | 0 |  |  |  |  | 0.375273 |
| Legionellales | 0.000429 | 0.000461 | 0.123788 |  | 0.000429 | 0.000461 | 0.122886 |  |  |  |  | 0.959415 |
| Tissierellales | 0.000429 | 0 | 0.000735 |  | 0.000429 | 0 | 0.000482 |  |  |  |  | 0.340893 |
| Veillonellales | 0.000421 | 0.005078 | 0.215522 |  | 0.000421 | 0.002086 | 0.213396 |  |  |  |  | 0.053483 |
| BM507 | 0.000392 | 0.002348 | 0.000286 |  | 0.000392 | 0.00135 | 0.000286 |  |  |  |  | 0.194464 |
| Propionibacteriales | 0.000392 | 0.001765 | 0.001043 |  | 0.000392 | 0.000561 | 0.001043 |  |  |  |  | 0.072831 |
| Acetobacterales | 0 | 0 | 0.008341 |  | 0 | 0 | 0.008341 |  |  |  |  | #DIV/0! |
| Bacillales_A | 0 | 0.00093 | 0.07559 |  | 0 | 0.000594 | 0.061546 |  |  |  |  | 0.148192 |
| Brachyspirales | 0 | 0 | 0.005213 |  | 0 | 0 | 0.005213 |  |  |  |  | #DIV/0! |
| Brevibacillales | 0 | 0.000516 | 0.005246 |  | 0 | 0.000516 | 0.00289 |  |  |  |  | 0.340893 |
| Caldilineales | 0 | 0 | 0.001043 |  | 0 | 0 | 0.001043 |  |  |  |  | #DIV/0! |
| Caulobacterales | 0 | 0 | 0.018761 |  | 0 | 0 | 0.009404 |  |  |  |  | #DIV/0! |
| Desulfuromonadales | 0 | 0 | 0.00282 |  | 0 | 0 | 0.001992 |  |  |  |  | #DIV/0! |
| Geminicoccales | 0 | 0.001966 | 0.000521 |  | 0 | 0.001015 | 0.000521 |  |  |  |  | 0.081443 |
| Gemmatales | 0 | 0 | 0.001564 |  | 0 | 0 | 0.001564 |  |  |  |  | #DIV/0! |
| Limnochordales | 0 | 0 | 0.000971 |  | 0 | 0 | 0.000614 |  |  |  |  | #DIV/0! |
| Rhodobacterales | 0 | 0 | 0.033154 |  | 0 | 0 | 0.018351 |  |  |  |  | #DIV/0! |
| Rickettsiales | 0 | 0.001802 | 0.000916 |  | 0 | 0.001364 | 0.000579 |  |  |  |  | 0.215764 |
| Thermoactinomycetales | 0 | 0 | 0.012086 |  | 0 | 0 | 0.009493 |  |  |  |  | #DIV/0! |
| Verrucomicrobiales | 0 | 0 | 0.006418 |  | 0 | 0 | 0.003547 |  |  |  |  | #DIV/0! |
| Xanthomonadales | 0 | 0 | 0.024649 |  | 0 | 0 | 0.018355 |  |  |  |  | #DIV/0! |

## S8.4 （Fig 8D）

| family | WT | Uox-/--1 | Uox-/--2 | SE | WT | Uox-/--1 | Uox-/--2 | total (top10) | |  |  | T-test Uox-/--2 vs Uox-/--1 |
| --- | --- | --- | --- | --- | --- | --- | --- | --- | --- | --- | --- | --- |
| Enterobacteriaceae | 33.73323 | 20.0927 | 5.797355 |  | 3.102258 | 1.287206 | 1.839576 | 92.21444 | 80.90852 | 76.13156 |  | 0.002282 |
| Vibrionaceae | 24.39686 | 5.85271 | 1.328305 |  | 2.245935 | 1.190791 | 0.931259 |  |  |  |  | 2.62E-05 |
| Lactobacillaceae | 15.27522 | 22.22642 | 63.73503 |  | 1.674372 | 2.405529 | 11.70953 |  |  |  |  | 0.039163 |
| Lachnospiraceae | 6.813365 | 11.58154 | 2.052473 |  | 2.029878 | 2.345947 | 1.292796 |  |  |  |  | 0.155306 |
| Bacteroidaceae | 3.083887 | 9.620592 | 0.746873 |  | 0.7093 | 3.679982 | 0.304109 |  |  |  |  | 0.111727 |
| Acutalibacteraceae | 2.862506 | 2.52417 | 0.655321 |  | 0.531632 | 0.482497 | 0.352478 |  |  |  |  | 0.647568 |
| Weeksellaceae | 2.0061 | 0.372911 | 0.073879 |  | 0.624336 | 0.13904 | 0.049778 |  |  |  |  | 0.0287 |
| Muribaculaceae | 1.679724 | 1.322329 | 0.475995 |  | 0.254451 | 0.207492 | 0.318322 |  |  |  |  | 0.301896 |
| Oscillospiraceae | 1.193018 | 4.632806 | 0.606162 |  | 0.180579 | 0.769447 | 0.342007 |  |  |  |  | 0.001439 |
| Ruminococcaceae | 1.17053 | 2.682345 | 0.66017 |  | 0.219073 | 0.567029 | 0.555569 |  |  |  |  | 0.032153 |
| Selenomonadaceae | 0.811037 | 2.987962 | 0.343555 |  | 0.368435 | 0.780771 | 0.15739 |  |  |  |  | 0.030308 |
| \| Treponemataceae \| \| --- \| | 0.734554 | 3.519425 | 0.646926 |  | 0.467528 | 1.264475 | 0.597525 |  |  |  |  | 0.065754 |
| Geodermatophilaceae | 0.715082 | 0.002658 | 0.001546 |  | 0.670106 | 0.001123 | 0.001063 |  |  |  |  | 0.312713 |
| Burkholderiaceae | 0.524034 | 0.337275 | 0.824778 |  | 0.139108 | 0.123312 | 0.519108 |  |  |  |  | 0.338758 |
| Enterococcaceae | 0.522348 | 0.773603 | 1.255618 |  | 0.029303 | 0.081941 | 0.213377 |  |  |  |  | 0.016184 |
| CAG-74 | 0.515374 | 0.333912 | 0.167147 |  | 0.143895 | 0.051509 | 0.116258 |  |  |  |  | 0.262548 |
| Amphibacillaceae | 0.374843 | 0.166583 | 0.074262 |  | 0.249001 | 0.068233 | 0.030097 |  |  |  |  | 0.438635 |
| Desulfovibrionaceae | 0.353885 | 0.729897 | 0.174103 |  | 0.139777 | 0.219614 | 0.12978 |  |  |  |  | 0.179216 |
| Vallitaleaceae | 0.256016 | 0.02479 | 0.009896 |  | 0.070573 | 0.012985 | 0.006141 |  |  |  |  | 0.009139 |
| Helicobacteraceae | 0.252004 | 0.210258 | 0.046328 |  | 0.10128 | 0.109917 | 0.03685 |  |  |  |  | 0.785698 |
| Eggerthellaceae | 0.241556 | 0.185202 | 0.079803 |  | 0.040158 | 0.029344 | 0.03726 |  |  |  |  | 0.283626 |
| Erysipelotrichaceae | 0.226934 | 0.049911 | 0.025325 |  | 0.112638 | 0.010822 | 0.00925 |  |  |  |  | 0.14879 |
| UBA1381 | 0.219004 | 1.249104 | 0.491914 |  | 0.057606 | 0.263914 | 0.27957 |  |  |  |  | 0.00341 |
| Tannerellaceae | 0.199991 | 0.034885 | 0.043238 |  | 0.036497 | 0.011172 | 0.028527 |  |  |  |  | 0.0015 |
| Saccharimonadaceae | 0.184007 | 3.230053 | 1.123563 |  | 0.055071 | 0.479178 | 0.648916 |  |  |  |  | 8.73E-05 |
| Peptostreptococcaceae | 0.164651 | 0.010676 | 0.05933 |  | 0.090785 | 0.003922 | 0.037568 |  |  |  |  | 0.121042 |
| Anaerovoracaceae | 0.163353 | 0.153992 | 0.049008 |  | 0.01524 | 0.018661 | 0.022353 |  |  |  |  | 0.705748 |
| CAG-611 | 0.1381 | 0.266273 | 0.11939 |  | 0.058571 | 0.101113 | 0.065118 |  |  |  |  | 0.298404 |
| Erysipelatoclostridiaceae | 0.095555 | 0.048341 | 0.015961 |  | 0.047596 | 0.018735 | 0.007516 |  |  |  |  | 0.377713 |
| Rikenellaceae | 0.093105 | 0.361612 | 0.097219 |  | 0.028018 | 0.150124 | 0.07228 |  |  |  |  | 0.10922 |
| Acetivibrionaceae | 0.074602 | 0.391139 | 0.044351 |  | 0.045779 | 0.302542 | 0.025748 |  |  |  |  | 0.325278 |
| Micrococcaceae | 0.067992 | 0.048373 | 10.66159 |  | 0.009987 | 0.010624 | 5.794935 |  |  |  |  | 0.208186 |
| CAG-1000 | 0.065733 | 0.152304 | 0.031084 |  | 0.044697 | 0.040909 | 0.022036 |  |  |  |  | 0.183558 |
| vadinHA17 | 0.058984 | 0.040947 | 0.01232 |  | 0.009556 | 0.005702 | 0.003741 |  |  |  |  | 0.136102 |
| Christensenellaceae | 0.055126 | 0.034603 | 0.011792 |  | 0.005543 | 0.003736 | 0.005607 |  |  |  |  | 0.011838 |
| Sphingomonadaceae | 0.043413 | 0.065877 | 0.092272 |  | 0.005802 | 0.008112 | 0.063807 |  |  |  |  | 0.047983 |
| UBA9935 | 0.042667 | 0.075836 | 0.034802 |  | 0.005102 | 0.009512 | 0.013013 |  |  |  |  | 0.011779 |
| Bifidobacteriaceae | 0.04049 | 0.039097 | 0.064817 |  | 0.009741 | 0.005247 | 0.008493 |  |  |  |  | 0.902354 |
| Methanobacteriaceae | 0.039878 | 0.015862 | 0.000286 |  | 0.015722 | 0.011457 | 0.000286 |  |  |  |  | 0.24523 |
| Acidaminococcaceae | 0.027033 | 0.230478 | 0.052241 |  | 0.007199 | 0.095587 | 0.033399 |  |  |  |  | 0.059785 |
| Anaerolineaceae | 0.026186 | 0.034008 | 0.030783 |  | 0.002959 | 0.005108 | 0.007551 |  |  |  |  | 0.214676 |
| Marinifilaceae | 0.022817 | 0.030559 | 0.002894 |  | 0.00462 | 0.005975 | 0.001048 |  |  |  |  | 0.329493 |
| Exiguobacteraceae | 0.021379 | 0.038087 | 0.049939 |  | 0.004476 | 0.003571 | 0.009826 |  |  |  |  | 0.015347 |
| Butyricicoccaceae | 0.021057 | 0.047239 | 0.00827 |  | 0.008607 | 0.011857 | 0.003653 |  |  |  |  | 0.104242 |
| Gastranaerophilaceae | 0.018735 | 0.102497 | 0.033563 |  | 0.004629 | 0.027532 | 0.017021 |  |  |  |  | 0.013339 |
| Thermaceae | 0.018026 | 0.027453 | 0.018351 |  | 0.002118 | 0.002238 | 0.005908 |  |  |  |  | 0.012058 |
| Anaerotignaceae | 0.017853 | 0.087284 | 0.012156 |  | 0.006128 | 0.024946 | 0.009163 |  |  |  |  | 0.022203 |
| CAG-822 | 0.017693 | 0.050028 | 0.032015 |  | 0.010501 | 0.024158 | 0.024625 |  |  |  |  | 0.247738 |
| QAMH01 | 0.015127 | 0.005464 | 0.002336 |  | 0.004229 | 0.003156 | 0.001108 |  |  |  |  | 0.097015 |
| Desulfonisporaceae | 0.014419 | 0.002359 | 0 |  | 0.009557 | 0.002359 | 0 |  |  |  |  | 0.248595 |
| Vallitaleaceae_A | 0.014091 | 0.000414 | 0 |  | 0.005081 | 0.000414 | 0 |  |  |  |  | 0.022985 |
| Coriobacteriaceae | 0.012194 | 0.023361 | 0.012156 |  | 0.004457 | 0.003967 | 0.00687 |  |  |  |  | 0.090791 |
| NAK82 | 0.011919 | 0.014246 | 0.008734 |  | 0.003148 | 0.003432 | 0.002088 |  |  |  |  | 0.628047 |
| Sphingobacteriaceae | 0.011745 | 0.072632 | 0.069611 |  | 0.002442 | 0.009184 | 0.023801 |  |  |  |  | 7.76E-05 |
| Dehalobacteriaceae | 0.011663 | 0.003205 | 0 |  | 0.004794 | 0.001117 | 0 |  |  |  |  | 0.116475 |
| Streptococcaceae | 0.010407 | 0.250683 | 1.106394 |  | 0.005021 | 0.058183 | 0.468408 |  |  |  |  | 0.002096 |
| Staphylococcaceae | 0.010288 | 0 | 0.079563 |  | 0.002798 | 0 | 0.051919 |  |  |  |  | 0.004271 |
| CAG-302 | 0.010146 | 0.004797 | 0.000504 |  | 0.006629 | 0.003166 | 0.000504 |  |  |  |  | 0.483188 |
| Elusimicrobiaceae | 0.009917 | 0.004127 | 0.002013 |  | 0.004748 | 0.002659 | 0.00103 |  |  |  |  | 0.312259 |
| Syntrophobacteraceae | 0.008671 | 0.010288 | 0.010502 |  | 0.001758 | 0.001745 | 0.004447 |  |  |  |  | 0.528543 |
| SK-Y3 | 0.007633 | 0.016087 | 0.00588 |  | 0.00252 | 0.004703 | 0.003217 |  |  |  |  | 0.144191 |
| UBA4823 | 0.007392 | 0.009542 | 0.011731 |  | 0.001143 | 0.002934 | 0.002384 |  |  |  |  | 0.510243 |
| CAG-239 | 0.007079 | 0.017769 | 0.001924 |  | 0.002618 | 0.011229 | 0.000991 |  |  |  |  | 0.375681 |
| unclassified | 0.006844 | 0.004101 | 0.008794 |  | 0.00589 | 0.000948 | 0.003124 |  |  |  |  | 0.655516 |
| Mycobacteriaceae | 0.006738 | 0.018705 | 0.788061 |  | 0.001255 | 0.001563 | 0.355418 |  |  |  |  | 0.000137 |
| Chitinophagaceae | 0.006347 | 0.004364 | 0.004906 |  | 0.002557 | 0.00177 | 0.00405 |  |  |  |  | 0.537921 |
| Ectothiorhodospiraceae | 0.005987 | 0.009731 | 0.002883 |  | 0.002182 | 0.002347 | 0.001541 |  |  |  |  | 0.26983 |
| Monoglobaceae | 0.005583 | 0.125523 | 0.052033 |  | 0.002053 | 0.014788 | 0.029929 |  |  |  |  | 1.14E-05 |
| Vagococcaceae | 0.005496 | 0.004271 | 0.010195 |  | 0.002889 | 0.001624 | 0.001774 |  |  |  |  | 0.719267 |
| MBNT15 | 0.005442 | 0.00768 | 0.002853 |  | 0.001667 | 0.001109 | 0.000787 |  |  |  |  | 0.289827 |
| Atopobiaceae | 0.005171 | 0 | 0.00185 |  | 0.002485 | 0 | 0.001533 |  |  |  |  | 0.064122 |
| Moraxellaceae | 0.005101 | 0.010213 | 0.193577 |  | 0.002088 | 0.005477 | 0.149479 |  |  |  |  | 0.40356 |
| Effusibacillaceae | 0.005063 | 0.002304 | 0.00601 |  | 0.001694 | 0.001347 | 0.002355 |  |  |  |  | 0.231055 |
| Rhizobiaceae | 0.005035 | 0.009094 | 0.619803 |  | 0.001724 | 0.000729 | 0.580579 |  |  |  |  | 0.055246 |
| Anaerofustaceae | 0.004872 | 0.003975 | 0.000504 |  | 0.002049 | 0.002178 | 0.000504 |  |  |  |  | 0.770289 |
| Actinomycetaceae | 0.004596 | 0.009212 | 0.487357 |  | 0.001911 | 0.002536 | 0.318511 |  |  |  |  | 0.17666 |
| Symbiobacteriaceae | 0.004525 | 0.005514 | 0.003169 |  | 0.001435 | 0.001275 | 0.001459 |  |  |  |  | 0.617784 |
| Ilumatobacteraceae | 0.004326 | 0.005047 | 0.009261 |  | 0.000464 | 0.000872 | 0.004138 |  |  |  |  | 0.482517 |
| Defluviitaleaceae | 0.004281 | 0.002834 | 0.000504 |  | 0.002616 | 0.001752 | 0.000504 |  |  |  |  | 0.655643 |
| Succinivibrionaceae | 0.004272 | 1.625579 | 0.043895 |  | 0.001582 | 0.424722 | 0.031319 |  |  |  |  | 0.003388 |
| Coprobacteraceae | 0.004095 | 0 | 0 |  | 0.001622 | 0 | 0 |  |  |  |  | 0.030132 |
| Kosmotogaceae | 0.004094 | 0.007004 | 0 |  | 0.001292 | 0.005414 | 0 |  |  |  |  | 0.612489 |
| Pasteurellaceae | 0.004037 | 0.021776 | 0.005267 |  | 0.002245 | 0.005814 | 0.002138 |  |  |  |  | 0.017367 |
| Pseudomonadaceae | 0.004035 | 0.017414 | 2.03686 |  | 0.001164 | 0.005173 | 2.023111 |  |  |  |  | 0.030221 |
| Microcystaceae | 0.003539 | 0.00657 | 0.006045 |  | 0.000907 | 0.00244 | 0.001764 |  |  |  |  | 0.271257 |
| Bacillaceae_A | 0.003509 | 0.00181 | 0.006423 |  | 0.001912 | 0.000579 | 0.001907 |  |  |  |  | 0.414879 |
| Savagellaceae | 0.003474 | 0.000885 | 0.000504 |  | 0.002199 | 0.000562 | 0.000504 |  |  |  |  | 0.280536 |
| Clostridiaceae | 0.003277 | 0.002185 | 0.201036 |  | 0.001779 | 0.001196 | 0.192552 |  |  |  |  | 0.621421 |
| Beutenbergiaceae | 0.00304 | 0.006841 | 0.004425 |  | 0.001013 | 0.002029 | 0.001299 |  |  |  |  | 0.12473 |
| Fimbriimonadaceae | 0.003005 | 0.000889 | 0.000752 |  | 0.000816 | 0.000564 | 0.000496 |  |  |  |  | 0.058682 |
| Streptosporangiaceae | 0.002252 | 0.001504 | 0.004615 |  | 0.000458 | 0.001046 | 0.002374 |  |  |  |  | 0.52722 |
| Filifactoraceae | 0.002226 | 0.001344 | 0 |  | 0.001264 | 0.000884 | 0 |  |  |  |  | 0.580188 |
| Steroidobacteraceae | 0.002191 | 0.002274 | 0.001402 |  | 0.001055 | 0.00132 | 0.000938 |  |  |  |  | 0.961845 |
| Hyphomicrobiaceae | 0.002056 | 0.003219 | 0.001546 |  | 0.000983 | 0.001271 | 0.001063 |  |  |  |  | 0.485892 |
| Anderseniellaceae | 0.00182 | 0.003308 | 0.000807 |  | 0.000882 | 0.001597 | 0.000542 |  |  |  |  | 0.433894 |
| Nostocaceae | 0.001606 | 0.002794 | 0.001492 |  | 0.00117 | 0.001031 | 0.000669 |  |  |  |  | 0.463955 |
| Hydrogenophilaceae | 0.001366 | 0.000923 | 0.001075 |  | 0.000616 | 0.000923 | 0.000682 |  |  |  |  | 0.698328 |
| UBA6898 | 0.001315 | 0.003169 | 0 |  | 0.000588 | 0.000873 | 0 |  |  |  |  | 0.108631 |
| Alicyclobacillaceae | 0.001271 | 0.003325 | 0.002235 |  | 0.000888 | 0.001341 | 0.000779 |  |  |  |  | 0.230257 |
| envOPS12 | 0.001261 | 0.00263 | 0.001324 |  | 0.000566 | 0.001396 | 0.000594 |  |  |  |  | 0.384658 |
| UBA932 | 0.00126 | 0.312355 | 0.078462 |  | 0.000565 | 0.073801 | 0.062033 |  |  |  |  | 0.001785 |
| Fermentibacteraceae | 0.001253 | 0.001877 | 0.00311 |  | 0.000562 | 0.000944 | 0.001191 |  |  |  |  | 0.582721 |
| Bacillaceae | 0.000955 | 0 | 0.047954 |  | 0.000607 | 0 | 0.035191 |  |  |  |  | 0.146655 |
| Xanthobacteraceae | 0.000886 | 0.001352 | 0.012277 |  | 0.000561 | 0.00089 | 0.006132 |  |  |  |  | 0.667429 |
| GCA-900066905 | 0.000876 | 0 | 0.000504 |  | 0.000554 | 0 | 0.000504 |  |  |  |  | 0.145048 |
| Reyranellaceae | 0.00085 | 0.000418 | 0.008537 |  | 0.000538 | 0.000418 | 0.005968 |  |  |  |  | 0.53982 |
| Aminicenantaceae | 0.000832 | 0.002275 | 0.000449 |  | 0.000527 | 0.00107 | 0.000449 |  |  |  |  | 0.254162 |
| Methanotrichaceae | 0.000821 | 0.000889 | 0.001219 |  | 0.00052 | 0.000564 | 0.00092 |  |  |  |  | 0.931089 |
| Bacillaceae_H | 0.000814 | 0.00093 | 0.022456 |  | 0.000515 | 0.000594 | 0.012509 |  |  |  |  | 0.885133 |
| URHD0088 | 0.000516 | 0.002222 | 0.000449 |  | 0.000516 | 0.00106 | 0.000449 |  |  |  |  | 0.178314 |
| P3 | 0.000447 | 0.114237 | 0.016762 |  | 0.000447 | 0.034066 | 0.011954 |  |  |  |  | 0.007491 |
| Brevibacteriaceae | 0.000439 | 0 | 0.045601 |  | 0.000439 | 0 | 0.031401 |  |  |  |  | 0.340893 |
| Bryobacteraceae | 0.000439 | 0 | 0.000504 |  | 0.000439 | 0 | 0.000504 |  |  |  |  | 0.340893 |
| Lentimicrobiaceae | 0.000439 | 0.068304 | 0.009803 |  | 0.000439 | 0.018812 | 0.008933 |  |  |  |  | 0.004796 |
| Oleiphilaceae | 0.000439 | 0.000988 | 0.000979 |  | 0.000439 | 0.000626 | 0.000632 |  |  |  |  | 0.489269 |
| Stappiaceae | 0.000439 | 0 | 0.030741 |  | 0.000439 | 0 | 0.030141 |  |  |  |  | 0.340893 |
| Legionellaceae | 0.000429 | 0.000461 | 0.123788 |  | 0.000429 | 0.000461 | 0.122886 |  |  |  |  | 0.959415 |
| Syntrophobotulaceae | 0.000429 | 0.001395 | 0 |  | 0.000429 | 0.000949 | 0 |  |  |  |  | 0.375273 |
| Tissierellaceae | 0.000429 | 0 | 0.000735 |  | 0.000429 | 0 | 0.000482 |  |  |  |  | 0.340893 |
| Megasphaeraceae | 0.000421 | 0 | 0.20256 |  | 0.000421 | 0 | 0.200973 |  |  |  |  | 0.340893 |
| Microbacteriaceae | 0.000421 | 0.000516 | 0.010447 |  | 0.000421 | 0.000516 | 0.005362 |  |  |  |  | 0.889518 |
| Propionibacteriaceae | 0.000392 | 0.001765 | 0.001043 |  | 0.000392 | 0.000561 | 0.001043 |  |  |  |  | 0.072831 |
| UBA12465 | 0.000392 | 0.002348 | 0.000286 |  | 0.000392 | 0.00135 | 0.000286 |  |  |  |  | 0.194464 |
| 33-17 | 0 | 0.001802 | 0.000916 |  | 0 | 0.001364 | 0.000579 |  |  |  |  | 0.215764 |
| Acetobacteraceae | 0 | 0 | 0.008341 |  | 0 | 0 | 0.008341 |  |  |  |  | #DIV/0! |
| Aerococcaceae | 0 | 0.004196 | 0.009316 |  | 0 | 0.001871 | 0.006971 |  |  |  |  | 0.04884 |
| Aeromonadaceae | 0 | 0 | 0.066123 |  | 0 | 0 | 0.040394 |  |  |  |  | #DIV/0! |
| Bacillaceae_G | 0 | 0 | 0.035387 |  | 0 | 0 | 0.034218 |  |  |  |  | #DIV/0! |
| Beijerinckiaceae | 0 | 0.00182 | 0.014389 |  | 0 | 0.000583 | 0.012212 |  |  |  |  | 0.01083 |
| Brachyspiraceae | 0 | 0 | 0.005213 |  | 0 | 0 | 0.005213 |  |  |  |  | #DIV/0! |
| Brevibacillaceae | 0 | 0.000516 | 0.005246 |  | 0 | 0.000516 | 0.00289 |  |  |  |  | 0.340893 |
| Caldibacillaceae | 0 | 0 | 0.018126 |  | 0 | 0 | 0.013972 |  |  |  |  | #DIV/0! |
| Caldilineaceae | 0 | 0 | 0.001043 |  | 0 | 0 | 0.001043 |  |  |  |  | #DIV/0! |
| Carnobacteriaceae | 0 | 0 | 0.00602 |  | 0 | 0 | 0.005684 |  |  |  |  | #DIV/0! |
| Caulobacteraceae | 0 | 0 | 0.018761 |  | 0 | 0 | 0.009404 |  |  |  |  | #DIV/0! |
| Dermabacteraceae | 0 | 0.000885 | 0.037711 |  | 0 | 0.000562 | 0.021935 |  |  |  |  | 0.146059 |
| Dermatophilaceae | 0 | 0.000471 | 0.005663 |  | 0 | 0.000471 | 0.004536 |  |  |  |  | 0.340893 |
| Desulfuromonadaceae_C | 0 | 0 | 0.00282 |  | 0 | 0 | 0.001992 |  |  |  |  | #DIV/0! |
| DEV007 | 0 | 0 | 0.006418 |  | 0 | 0 | 0.003547 |  |  |  |  | #DIV/0! |
| Devosiaceae | 0 | 0 | 0.087321 |  | 0 | 0 | 0.060846 |  |  |  |  | #DIV/0! |
| Dialisteraceae | 0 | 0 | 0.012512 |  | 0 | 0 | 0.012512 |  |  |  |  | #DIV/0! |
| Ethanoligenenaceae | 0 | 0 | 0.005213 |  | 0 | 0 | 0.005213 |  |  |  |  | #DIV/0! |
| Flavobacteriaceae | 0 | 0 | 0.006542 |  | 0 | 0 | 0.006205 |  |  |  |  | #DIV/0! |
| Geminicoccaceae | 0 | 0.001966 | 0.000521 |  | 0 | 0.001015 | 0.000521 |  |  |  |  | 0.081443 |
| Gemmataceae | 0 | 0 | 0.001564 |  | 0 | 0 | 0.001564 |  |  |  |  | #DIV/0! |
| Halomonadaceae | 0 | 0 | 0.016402 |  | 0 | 0 | 0.010636 |  |  |  |  | #DIV/0! |
| Listeriaceae | 0 | 0 | 0.002806 |  | 0 | 0 | 0.002239 |  |  |  |  | #DIV/0! |
| Planococcaceae | 0 | 0.00093 | 0.07559 |  | 0 | 0.000594 | 0.061546 |  |  |  |  | 0.148192 |
| Rhodobacteraceae | 0 | 0 | 0.033154 |  | 0 | 0 | 0.018351 |  |  |  |  | #DIV/0! |
| Salinicoccaceae | 0 | 0 | 0.045009 |  | 0 | 0 | 0.026783 |  |  |  |  | #DIV/0! |
| Saprospiraceae | 0 | 0 | 0.006256 |  | 0 | 0 | 0.006256 |  |  |  |  | #DIV/0! |
| Sporolactobacillaceae | 0 | 0.000418 | 0.008863 |  | 0 | 0.000418 | 0.008863 |  |  |  |  | 0.340893 |
| Thermoactinomycetaceae | 0 | 0 | 0.012086 |  | 0 | 0 | 0.009493 |  |  |  |  | #DIV/0! |
| UBA10212 | 0 | 0 | 0.003128 |  | 0 | 0 | 0.003128 |  |  |  |  | #DIV/0! |
| Veillonellaceae | 0 | 0.005078 | 0.000449 |  | 0 | 0.002086 | 0.000449 |  |  |  |  | 0.035168 |
| Xanthomonadaceae | 0 | 0 | 0.024649 |  | 0 | 0 | 0.018355 |  |  |  |  | #DIV/0! |
| ZCTH02-B6 | 0 | 0 | 0.000971 |  | 0 | 0 | 0.000614 |  |  |  |  | #DIV/0! |

## S8.5 （Fig 8E）

| genus | WT | Uox-/--1 | Uox-/--2 | SE | WT | Uox-/--1 | Uox-/--2 | total (top10) | |  |  | T-test Uox-/--2 vs Uox-/--1 |
| --- | --- | --- | --- | --- | --- | --- | --- | --- | --- | --- | --- | --- |
| Cronobacter | 29.60159 | 15.44484 | 4.003235 |  | 3.558415 | 1.526519 | 1.294993 | 80.14219 | 58.72267 | 71.08242 |  | 0.004417 |
| Vibrio | 24.39686 | 5.85271 | 1.328305 |  | 2.245935 | 1.190791 | 0.931259 |  |  |  |  | 2.62E-05 |
| Lactobacillus | 7.505048 | 16.72092 | 24.38903 |  | 2.005983 | 1.431202 | 8.576177 |  |  |  |  | 0.003847 |
| Lactobacillus_B | 5.07761 | 0.936586 | 19.37363 |  | 1.725464 | 0.271914 | 9.268771 |  |  |  |  | 0.039231 |
| Salmonella | 3.822267 | 3.433833 | 0.884485 |  | 0.643055 | 0.258176 | 0.528705 |  |  |  |  | 0.587439 |
| Lactobacillus_H | 2.679597 | 4.547605 | 19.90859 |  | 0.851359 | 1.024754 | 3.246862 |  |  |  |  | 0.191147 |
| Eubacterium_R | 2.176954 | 1.75224 | 0.396628 |  | 0.66994 | 0.539287 | 0.203282 |  |  |  |  | 0.632088 |
| Chryseobacterium | 2.0061 | 0.372911 | 0.073879 |  | 0.624336 | 0.13904 | 0.049778 |  |  |  |  | 0.0287 |
| Duncaniella | 1.5265 | 0.530262 | 0.089861 |  | 0.251642 | 0.115262 | 0.03701 |  |  |  |  | 0.004853 |
| Prevotella | 1.349656 | 9.130768 | 0.634789 |  | 0.648261 | 3.597761 | 0.266009 |  |  |  |  | 0.059172 |
| 992a | 1.260169 | 0.835412 | 0.083304 |  | 0.681855 | 0.205217 | 0.055149 |  |  |  |  | 0.564093 |
| \| Kineothrix \| \| --- \| | 1.094858 | 4.008629 | 0.439764 |  | 0.658589 | 1.167782 | 0.20368 |  |  |  |  | 0.05486 |
| Bacteroides | 0.833399 | 0.244358 | 0.051438 |  | 0.174859 | 0.064475 | 0.01954 |  |  |  |  | 0.010148 |
| Megamonas | 0.805916 | 0.002753 | 0.004148 |  | 0.367941 | 0.000737 | 0.001283 |  |  |  |  | 0.053985 |
| Treponema_D | 0.734554 | 3.519425 | 0.646926 |  | 0.467528 | 1.264475 | 0.597525 |  |  |  |  | 0.065754 |
| Blastococcus | 0.715082 | 0.002658 | 0.001546 |  | 0.670106 | 0.001123 | 0.001063 |  |  |  |  | 0.312713 |
| Blautia_A | 0.631271 | 0.025735 | 0.014631 |  | 0.341195 | 0.005016 | 0.006442 |  |  |  |  | 0.106363 |
| Bacteroides_B | 0.603874 | 0.072457 | 0.016435 |  | 0.223829 | 0.027185 | 0.010394 |  |  |  |  | 0.040166 |
| Ruminococcus_A | 0.564536 | 0.056094 | 0.009674 |  | 0.427071 | 0.015826 | 0.006621 |  |  |  |  | 0.261639 |
| Clostridium_Q | 0.523217 | 0.22925 | 0.045973 |  | 0.320668 | 0.048213 | 0.03044 |  |  |  |  | 0.385972 |
| Enterococcus | 0.522348 | 0.773603 | 1.255618 |  | 0.029303 | 0.081941 | 0.213377 |  |  |  |  | 0.016184 |
| OEMS01 | 0.515374 | 0.333912 | 0.167147 |  | 0.143895 | 0.051509 | 0.116258 |  |  |  |  | 0.262548 |
| Parasutterella | 0.450653 | 0.001289 | 0.000916 |  | 0.139153 | 0.000864 | 0.000579 |  |  |  |  | 0.009033 |
| Anaerostipes | 0.432653 | 0.376946 | 0.089695 |  | 0.169612 | 0.063933 | 0.038978 |  |  |  |  | 0.764898 |
| CAG-217 | 0.422143 | 0.09824 | 0.088391 |  | 0.221969 | 0.054396 | 0.085106 |  |  |  |  | 0.186793 |
| Lawsonibacter | 0.418403 | 2.521687 | 0.235996 |  | 0.102599 | 0.663735 | 0.141198 |  |  |  |  | 0.01066 |
| Virgibacillus_G | 0.374843 | 0.166583 | 0.015986 |  | 0.249001 | 0.068233 | 0.010838 |  |  |  |  | 0.438635 |
| Desulfovibrio | 0.337052 | 0.716295 | 0.172629 |  | 0.134763 | 0.218416 | 0.129458 |  |  |  |  | 0.170278 |
| Ruminiclostridium_E | 0.297596 | 1.16605 | 0.375311 |  | 0.141386 | 0.431681 | 0.337253 |  |  |  |  | 0.084943 |
| Prevotellamassilia | 0.285295 | 0.173008 | 0.031699 |  | 0.139097 | 0.040567 | 0.01853 |  |  |  |  | 0.456287 |
| Faecalicatena | 0.280271 | 0.433396 | 0.051999 |  | 0.124965 | 0.139522 | 0.029033 |  |  |  |  | 0.432675 |
| Flavonifractor | 0.277764 | 0.974509 | 0.144057 |  | 0.034537 | 0.124978 | 0.083852 |  |  |  |  | 0.000313 |
| Vallitalea | 0.256016 | 0.02479 | 0.009896 |  | 0.070573 | 0.012985 | 0.006141 |  |  |  |  | 0.009139 |
| Agathobacter | 0.244928 | 0.921727 | 0.067071 |  | 0.141574 | 0.263182 | 0.05681 |  |  |  |  | 0.04699 |
| Helicobacter_C | 0.237841 | 0.048012 | 0.015713 |  | 0.093783 | 0.024719 | 0.009911 |  |  |  |  | 0.078791 |
| 2-14 | 0.22563 | 0.200059 | 0.019131 |  | 0.167542 | 0.070087 | 0.009231 |  |  |  |  | 0.890825 |
| CAG-41 | 0.219004 | 1.249104 | 0.491914 |  | 0.057606 | 0.263914 | 0.27957 |  |  |  |  | 0.00341 |
| Bacteroides_F | 0.209017 | 0.32962 | 0.10681 |  | 0.153141 | 0.196605 | 0.061321 |  |  |  |  | 0.63885 |
| Parabacteroides | 0.199991 | 0.034885 | 0.043238 |  | 0.036497 | 0.011172 | 0.028527 |  |  |  |  | 0.0015 |
| Eubacterium_Q | 0.186974 | 0.339109 | 0.115623 |  | 0.090569 | 0.138463 | 0.096655 |  |  |  |  | 0.379475 |
| Saccharimonas | 0.184007 | 2.926729 | 1.04078 |  | 0.055071 | 0.457254 | 0.615412 |  |  |  |  | 0.00014 |
| Adlercreutzia | 0.165323 | 0.033856 | 0.015002 |  | 0.034196 | 0.010513 | 0.005336 |  |  |  |  | 0.004283 |
| Romboutsia | 0.164651 | 0.010676 | 0.05933 |  | 0.090785 | 0.003922 | 0.037568 |  |  |  |  | 0.121042 |
| Klebsiella | 0.155949 | 0.186751 | 0.493665 |  | 0.024196 | 0.018532 | 0.266167 |  |  |  |  | 0.336007 |
| Dorea | 0.153206 | 0.4927 | 0.119908 |  | 0.039757 | 0.093505 | 0.070536 |  |  |  |  | 0.007474 |
| Oscillibacter | 0.139901 | 0.208162 | 0.020882 |  | 0.060471 | 0.063895 | 0.017066 |  |  |  |  | 0.455747 |
| Ruminococcus_C | 0.13776 | 0.28248 | 0.039404 |  | 0.061315 | 0.098087 | 0.026452 |  |  |  |  | 0.239377 |
| UBA2730 | 0.137707 | 0.263277 | 0.117429 |  | 0.058239 | 0.100777 | 0.064134 |  |  |  |  | 0.306002 |
| Provencibacterium | 0.13656 | 0.057218 | 0.020453 |  | 0.032334 | 0.01089 | 0.01773 |  |  |  |  | 0.042376 |
| Intestinimonas | 0.135559 | 0.398819 | 0.08772 |  | 0.070851 | 0.118176 | 0.041085 |  |  |  |  | 0.085118 |
| Anaerotruncus | 0.135266 | 0.011984 | 0.002805 |  | 0.047791 | 0.002586 | 0.002478 |  |  |  |  | 0.027611 |
| Acutalibacter | 0.129584 | 0.588878 | 0.153042 |  | 0.025722 | 0.153108 | 0.072785 |  |  |  |  | 0.014329 |
| CAG-1031 | 0.126137 | 0.032325 | 0.008957 |  | 0.017681 | 0.009914 | 0.005738 |  |  |  |  | 0.000939 |
| UC5-1-2E3 | 0.124022 | 0.071985 | 0.016461 |  | 0.063454 | 0.017963 | 0.007541 |  |  |  |  | 0.448379 |
| Anaeromassilibacillus | 0.115991 | 0 | 0 |  | 0.018494 | 0 | 0 |  |  |  |  | 9.24E-05 |
| Fournierella | 0.114281 | 0.109688 | 0.013362 |  | 0.033506 | 0.046809 | 0.008416 |  |  |  |  | 0.937985 |
| Emergencia | 0.0894 | 0.020067 | 0.006628 |  | 0.00996 | 0.003669 | 0.004305 |  |  |  |  | 6.62E-05 |
| An181 | 0.087747 | 0 | 0 |  | 0.061286 | 0 | 0 |  |  |  |  | 0.182716 |
| Ruminococcus_D | 0.087462 | 0.134826 | 0.038566 |  | 0.043384 | 0.047358 | 0.035227 |  |  |  |  | 0.477787 |
| Providencia | 0.086582 | 0.122445 | 0.097423 |  | 0.012326 | 0.015093 | 0.030638 |  |  |  |  | 0.095539 |
| Faecalibaculum | 0.085548 | 0.000461 | 0.000286 |  | 0.061528 | 0.000461 | 0.000286 |  |  |  |  | 0.196807 |
| CAG-81 | 0.081098 | 0.038625 | 0.016895 |  | 0.064316 | 0.009191 | 0.007385 |  |  |  |  | 0.528034 |
| GCA-900066575 | 0.076823 | 0.14014 | 0.02642 |  | 0.015777 | 0.035473 | 0.0177 |  |  |  |  | 0.133968 |
| Sutterella | 0.07256 | 0.333799 | 0.040911 |  | 0.020955 | 0.123463 | 0.029457 |  |  |  |  | 0.063544 |
| Butyrivibrio_A | 0.072406 | 1.843429 | 0.542811 |  | 0.050323 | 0.727146 | 0.459676 |  |  |  |  | 0.035464 |
| Ruthenibacterium | 0.069896 | 0.048852 | 0.009061 |  | 0.039777 | 0.020576 | 0.006141 |  |  |  |  | 0.6485 |
| Rothia | 0.067992 | 0.047912 | 10.64818 |  | 0.009987 | 0.010525 | 5.786504 |  |  |  |  | 0.196471 |
| UBA6985 | 0.065733 | 0.152304 | 0.031084 |  | 0.044697 | 0.040909 | 0.022036 |  |  |  |  | 0.183558 |
| COE1 | 0.05976 | 0.038578 | 0.004011 |  | 0.025662 | 0.012423 | 0.002887 |  |  |  |  | 0.474612 |
| UBA1394 | 0.058117 | 0.123922 | 0.067104 |  | 0.039489 | 0.032148 | 0.063384 |  |  |  |  | 0.225312 |
| Ruminiclostridium_C | 0.054058 | 0.110696 | 0.018766 |  | 0.007595 | 0.012478 | 0.010421 |  |  |  |  | 0.003073 |
| Ruminiclostridium_A | 0.053358 | 0.385816 | 0.042373 |  | 0.045597 | 0.302915 | 0.025087 |  |  |  |  | 0.303258 |
| UBA2212 | 0.052325 | 0.008141 | 0.003648 |  | 0.019277 | 0.002594 | 0.002119 |  |  |  |  | 0.046446 |
| CAG-110 | 0.051898 | 0.011157 | 0.003217 |  | 0.01488 | 0.003777 | 0.001845 |  |  |  |  | 0.024156 |
| CAG-95 | 0.050661 | 0.390995 | 0.076822 |  | 0.024236 | 0.093797 | 0.049591 |  |  |  |  | 0.005603 |
| Christensenella | 0.049775 | 0.024815 | 0.007337 |  | 0.005192 | 0.004397 | 0.004178 |  |  |  |  | 0.004327 |
| LD21 | 0.04903 | 0.034933 | 0.010492 |  | 0.008423 | 0.005283 | 0.003676 |  |  |  |  | 0.186661 |
| Clostridium_N | 0.047879 | 0.041146 | 0.022228 |  | 0.027606 | 0.028711 | 0.015827 |  |  |  |  | 0.869135 |
| CAG-45 | 0.04657 | 0.000875 | 0 |  | 0.046036 | 0.000555 | 0 |  |  |  |  | 0.344353 |
| Eubacterium_J | 0.046134 | 0.010878 | 0.002482 |  | 0.03315 | 0.004427 | 0.001441 |  |  |  |  | 0.316605 |
| W-Firmicutes-11 | 0.04425 | 0.074756 | 0.018014 |  | 0.006966 | 0.009837 | 0.010721 |  |  |  |  | 0.029828 |
| MYbin3 | 0.042667 | 0.075836 | 0.034802 |  | 0.005102 | 0.009512 | 0.013013 |  |  |  |  | 0.011779 |
| Alistipes | 0.042232 | 0.017194 | 0.007587 |  | 0.017359 | 0.007976 | 0.006373 |  |  |  |  | 0.219292 |
| Allobaculum | 0.041971 | 0 | 0 |  | 0.027927 | 0 | 0 |  |  |  |  | 0.163773 |
| Bifidobacterium | 0.04049 | 0.039097 | 0.064817 |  | 0.009741 | 0.005247 | 0.008493 |  |  |  |  | 0.902354 |
| Methanosphaera | 0.039878 | 0.015862 | 0.000286 |  | 0.015722 | 0.011457 | 0.000286 |  |  |  |  | 0.24523 |
| CHKCI006 | 0.039037 | 0.005119 | 0.000449 |  | 0.038055 | 0.002626 | 0.000449 |  |  |  |  | 0.394789 |
| Absiella | 0.034351 | 0.0314 | 0.01722 |  | 0.027698 | 0.008484 | 0.007896 |  |  |  |  | 0.920882 |
| Marvinbryantia | 0.033787 | 0.012902 | 0.002281 |  | 0.010836 | 0.006198 | 0.000929 |  |  |  |  | 0.125257 |
| Rikenella | 0.03296 | 0.344 | 0.089632 |  | 0.009878 | 0.150707 | 0.065976 |  |  |  |  | 0.066449 |
| Robinsoniella | 0.030798 | 0.017124 | 0.003452 |  | 0.023437 | 0.00549 | 0.001822 |  |  |  |  | 0.582526 |
| Sporobacter | 0.02972 | 0.047395 | 0.016758 |  | 0.00652 | 0.006603 | 0.010889 |  |  |  |  | 0.08596 |
| Sphingomonas_A | 0.028884 | 0.048637 | 0.032955 |  | 0.004384 | 0.006601 | 0.013226 |  |  |  |  | 0.031841 |
| Lachnoclostridium_A | 0.028627 | 0.027777 | 0.008448 |  | 0.00705 | 0.006835 | 0.004685 |  |  |  |  | 0.932704 |
| Lachnospira | 0.028289 | 0.000934 | 0 |  | 0.014251 | 0.000596 | 0 |  |  |  |  | 0.084109 |
| Pseudoflavonifractor | 0.028173 | 0.005677 | 0.000504 |  | 0.013413 | 0.002397 | 0.000504 |  |  |  |  | 0.129736 |
| Eisenbergiella | 0.027538 | 0.033745 | 0.003976 |  | 0.010164 | 0.008538 | 0.003465 |  |  |  |  | 0.650107 |
| Papillibacter | 0.027429 | 0.029856 | 0.005873 |  | 0.005969 | 0.007024 | 0.001602 |  |  |  |  | 0.797661 |
| Phascolarctobacterium | 0.027033 | 0.230478 | 0.052241 |  | 0.007199 | 0.095587 | 0.033399 |  |  |  |  | 0.059785 |
| Stoquefichus | 0.0263 | 0.000471 | 0 |  | 0.007644 | 0.000471 | 0 |  |  |  |  | 0.00709 |
| Eubacterium_G | 0.026205 | 0.032369 | 0.000521 |  | 0.006901 | 0.009392 | 0.000521 |  |  |  |  | 0.608473 |
| Proteus | 0.025155 | 0.028275 | 0.028784 |  | 0.005933 | 0.003047 | 0.00879 |  |  |  |  | 0.649994 |
| Negativibacillus | 0.025121 | 0.006273 | 0.004534 |  | 0.014076 | 0.002999 | 0.004534 |  |  |  |  | 0.219624 |
| DNF00809 | 0.024482 | 0.021218 | 0.013512 |  | 0.004865 | 0.006038 | 0.007685 |  |  |  |  | 0.682701 |
| Massilimaliae | 0.024087 | 0.041665 | 0.011027 |  | 0.003596 | 0.019815 | 0.008222 |  |  |  |  | 0.403213 |
| TWA4 | 0.02369 | 0.015651 | 0.002536 |  | 0.008766 | 0.004385 | 0.001978 |  |  |  |  | 0.431235 |
| Marseille-P3106 | 0.023529 | 0.145507 | 0.037341 |  | 0.004563 | 0.016009 | 0.017959 |  |  |  |  | 2.52E-05 |
| Erysipelatoclostridium | 0.021828 | 0.021921 | 0.005716 |  | 0.013291 | 0.009712 | 0.002657 |  |  |  |  | 0.995613 |
| Raoultibacter | 0.021227 | 0.030816 | 0.016613 |  | 0.003335 | 0.005769 | 0.010074 |  |  |  |  | 0.180728 |
| Morganella | 0.021167 | 0.027977 | 0.021348 |  | 0.003665 | 0.002683 | 0.010412 |  |  |  |  | 0.164661 |
| Coprococcus_B | 0.019843 | 0.036101 | 0.005734 |  | 0.010499 | 0.013078 | 0.002203 |  |  |  |  | 0.355194 |
| Bittarella | 0.019302 | 0 | 0.000504 |  | 0.004189 | 0 | 0.000504 |  |  |  |  | 0.000968 |
| Eubacterium_I | 0.018898 | 0.001809 | 0 |  | 0.013242 | 0.000917 | 0 |  |  |  |  | 0.226939 |
| UBA1191 | 0.018503 | 0.035791 | 0.018378 |  | 0.005383 | 0.007701 | 0.006608 |  |  |  |  | 0.095626 |
| TF01-11 | 0.018167 | 0.096613 | 0.019493 |  | 0.006223 | 0.027636 | 0.013711 |  |  |  |  | 0.019814 |
| Meiothermus_B | 0.018026 | 0.027453 | 0.018351 |  | 0.002118 | 0.002238 | 0.005908 |  |  |  |  | 0.012058 |
| Alistipes_A | 0.017913 | 0.000418 | 0 |  | 0.011637 | 0.000418 | 0 |  |  |  |  | 0.163871 |
| Acetivibrio | 0.017632 | 0.002797 | 0.000953 |  | 0.005091 | 0.000719 | 0.000604 |  |  |  |  | 0.01624 |
| CAG-878 | 0.017271 | 0.009035 | 0.002015 |  | 0.0102 | 0.007311 | 0.002015 |  |  |  |  | 0.526447 |
| Angelakisella | 0.017255 | 0.019149 | 0.002464 |  | 0.004798 | 0.003166 | 0.001975 |  |  |  |  | 0.748602 |
| Bilophila | 0.016833 | 0.013602 | 0.001474 |  | 0.013226 | 0.004907 | 0.000662 |  |  |  |  | 0.82346 |
| Enteroscipio | 0.015472 | 0.030414 | 0.007548 |  | 0.007774 | 0.004078 | 0.002683 |  |  |  |  | 0.119576 |
| Muribaculum | 0.015454 | 0.628741 | 0.300602 |  | 0.002709 | 0.166621 | 0.215008 |  |  |  |  | 0.004245 |
| QAMH01 | 0.015127 | 0.005464 | 0.002336 |  | 0.004229 | 0.003156 | 0.001108 |  |  |  |  | 0.097015 |
| Gordonibacter | 0.015052 | 0.005018 | 0.001814 |  | 0.003715 | 0.001903 | 0.001335 |  |  |  |  | 0.037076 |
| Desulfonispora | 0.014419 | 0.002359 | 0 |  | 0.009557 | 0.002359 | 0 |  |  |  |  | 0.248595 |
| Tyzzerella | 0.014217 | 0.050604 | 0.033871 |  | 0.005608 | 0.015854 | 0.026645 |  |  |  |  | 0.055753 |
| Helicobacter_D | 0.014164 | 0.162246 | 0.030615 |  | 0.007873 | 0.100176 | 0.027148 |  |  |  |  | 0.171338 |
| Vallitalea_A | 0.014091 | 0.000414 | 0 |  | 0.005081 | 0.000414 | 0 |  |  |  |  | 0.022985 |
| Massilioclostridium | 0.013966 | 0.115421 | 0.031991 |  | 0.005438 | 0.031342 | 0.02402 |  |  |  |  | 0.009664 |
| Butyricimonas | 0.013855 | 0.000832 | 0 |  | 0.002821 | 0.000526 | 0 |  |  |  |  | 0.001077 |
| Roseburia | 0.013828 | 0.09386 | 0.009317 |  | 0.009637 | 0.037585 | 0.007828 |  |  |  |  | 0.066095 |
| Exiguobacterium_A | 0.013076 | 0.022228 | 0.037053 |  | 0.003154 | 0.002612 | 0.007198 |  |  |  |  | 0.049441 |
| 49-20 | 0.012899 | 0.018081 | 0.013032 |  | 0.001894 | 0.00231 | 0.003338 |  |  |  |  | 0.113494 |
| Ruminococcus | 0.012872 | 0.332067 | 0.024007 |  | 0.005275 | 0.139351 | 0.017764 |  |  |  |  | 0.045095 |
| Holdemania | 0.01274 | 0.009909 | 0 |  | 0.004361 | 0.002452 | 0 |  |  |  |  | 0.583944 |
| CAG-353 | 0.012336 | 0.225838 | 0.008196 |  | 0.009549 | 0.090903 | 0.004295 |  |  |  |  | 0.041635 |
| Collinsella | 0.012194 | 0.023361 | 0.012156 |  | 0.004457 | 0.003967 | 0.00687 |  |  |  |  | 0.090791 |
| NAK82 | 0.011919 | 0.014246 | 0.008734 |  | 0.003148 | 0.003432 | 0.002088 |  |  |  |  | 0.628047 |
| Sphingobacterium | 0.011745 | 0.044446 | 0.067092 |  | 0.002442 | 0.013886 | 0.023615 |  |  |  |  | 0.042819 |
| Dehalobacterium | 0.011663 | 0.003205 | 0 |  | 0.004794 | 0.001117 | 0 |  |  |  |  | 0.116475 |
| Paramuribaculum | 0.011632 | 0.131 | 0.076575 |  | 0.002063 | 0.031667 | 0.062886 |  |  |  |  | 0.003713 |
| CAG-194 | 0.011611 | 0.131169 | 0.041797 |  | 0.005177 | 0.040185 | 0.032328 |  |  |  |  | 0.014515 |
| Weissella | 0.011602 | 0.019382 | 0.033611 |  | 0.001904 | 0.003197 | 0.00618 |  |  |  |  | 0.063061 |
| Erwinia | 0.011529 | 0.002634 | 0 |  | 0.00734 | 0.001399 | 0 |  |  |  |  | 0.261357 |
| RUG754 | 0.0112 | 0.017978 | 0.002519 |  | 0.004411 | 0.006395 | 0.002519 |  |  |  |  | 0.403479 |
| Agathobaculum | 0.010994 | 0.011431 | 0.000953 |  | 0.003669 | 0.002302 | 0.000604 |  |  |  |  | 0.92158 |
| ASF356 | 0.010666 | 0.001549 | 0.001797 |  | 0.005717 | 0.001549 | 0.001481 |  |  |  |  | 0.154793 |
| Streptococcus | 0.010407 | 0.250683 | 1.106394 |  | 0.005021 | 0.058183 | 0.468408 |  |  |  |  | 0.002096 |
| CAG-302 | 0.010146 | 0.004797 | 0.000504 |  | 0.006629 | 0.003166 | 0.000504 |  |  |  |  | 0.483188 |
| CAG-306 | 0.010112 | 0.009884 | 0.007635 |  | 0.003868 | 0.002821 | 0.003557 |  |  |  |  | 0.962992 |
| SR-FBR-E99 | 0.009954 | 0.006014 | 0.001828 |  | 0.001261 | 0.001639 | 0.000583 |  |  |  |  | 0.085818 |
| DTU053 | 0.009444 | 0 | 0 |  | 0.003859 | 0 | 0 |  |  |  |  | 0.034402 |
| UBA4782 | 0.009349 | 0.011046 | 0.009703 |  | 0.002381 | 0.002065 | 0.002311 |  |  |  |  | 0.602045 |
| Rhizorhabdus | 0.009313 | 0.010139 | 0.006416 |  | 0.001647 | 0.001894 | 0.001593 |  |  |  |  | 0.749094 |
| Butyricicoccus | 0.009101 | 0.029857 | 0.004872 |  | 0.00517 | 0.011293 | 0.002282 |  |  |  |  | 0.125638 |
| Odoribacter | 0.008962 | 0.029727 | 0.002894 |  | 0.001988 | 0.005587 | 0.001048 |  |  |  |  | 0.005711 |
| Acetitomaculum | 0.008865 | 0.065527 | 0.010446 |  | 0.005696 | 0.028756 | 0.00618 |  |  |  |  | 0.082045 |
| Syntrophobacter | 0.008671 | 0.010288 | 0.010502 |  | 0.001758 | 0.001745 | 0.004447 |  |  |  |  | 0.528543 |
| Beduini | 0.00839 | 0.02083 | 0.009796 |  | 0.00418 | 0.007374 | 0.005122 |  |  |  |  | 0.172966 |
| Exiguobacterium | 0.008303 | 0.015859 | 0.012887 |  | 0.001657 | 0.002768 | 0.003566 |  |  |  |  | 0.041195 |
| Staphylococcus | 0.008174 | 0 | 0.010439 |  | 0.003167 | 0 | 0.00763 |  |  |  |  | 0.02739 |
| SK-Y3 | 0.007633 | 0.016087 | 0.00588 |  | 0.00252 | 0.004703 | 0.003217 |  |  |  |  | 0.144191 |
| UTCFX2 | 0.007392 | 0.009542 | 0.011731 |  | 0.001143 | 0.002934 | 0.002384 |  |  |  |  | 0.510243 |
| CAG-484 | 0.007362 | 0.015159 | 0.003775 |  | 0.002528 | 0.004671 | 0.002007 |  |  |  |  | 0.172871 |
| Anaerotignum | 0.007187 | 0.085735 | 0.010359 |  | 0.002475 | 0.025192 | 0.007732 |  |  |  |  | 0.011193 |
| CAG-495 | 0.007079 | 0.017769 | 0.001924 |  | 0.002618 | 0.011229 | 0.000991 |  |  |  |  | 0.375681 |
| Hydrogenoanaerobacterium | 0.007012 | 0.004205 | 0.000449 |  | 0.001629 | 0.001195 | 0.000449 |  |  |  |  | 0.194983 |
| unclassified | 0.006844 | 0.004101 | 0.008794 |  | 0.00589 | 0.000948 | 0.003124 |  |  |  |  | 0.655516 |
| Corynebacterium | 0.006738 | 0.018705 | 0.72676 |  | 0.001255 | 0.001563 | 0.328255 |  |  |  |  | 0.000137 |
| UBA1436 | 0.006586 | 0.003251 | 0.002013 |  | 0.003403 | 0.002312 | 0.00103 |  |  |  |  | 0.436409 |
| UBA6398 | 0.006357 | 0 | 0 |  | 0.005469 | 0 | 0 |  |  |  |  | 0.27211 |
| Thioalkalivibrio_A | 0.005987 | 0.009731 | 0.002883 |  | 0.002182 | 0.002347 | 0.001541 |  |  |  |  | 0.26983 |
| UBA1033 | 0.005746 | 0 | 0 |  | 0.00125 | 0 | 0 |  |  |  |  | 0.000983 |
| Escherichia | 0.005606 | 0.842007 | 0.225627 |  | 0.00096 | 0.307422 | 0.183971 |  |  |  |  | 0.021535 |
| Monoglobus | 0.005583 | 0.125523 | 0.052033 |  | 0.002053 | 0.014788 | 0.029929 |  |  |  |  | 1.14E-05 |
| Vagococcus | 0.005496 | 0.004271 | 0.010195 |  | 0.002889 | 0.001624 | 0.001774 |  |  |  |  | 0.719267 |
| Thermoflavifilum | 0.00549 | 0.003536 | 0.001256 |  | 0.002245 | 0.001072 | 0.000592 |  |  |  |  | 0.450359 |
| CG2-30-66-27 | 0.005442 | 0.00768 | 0.002853 |  | 0.001667 | 0.001109 | 0.000787 |  |  |  |  | 0.289827 |
| Christensenella_A | 0.005351 | 0.009789 | 0.004455 |  | 0.00191 | 0.002599 | 0.002515 |  |  |  |  | 0.198946 |
| Paraprevotella | 0.005306 | 0 | 0 |  | 0.003363 | 0 | 0 |  |  |  |  | 0.145673 |
| ER4 | 0.005268 | 0.002644 | 0 |  | 0.003019 | 0.000967 | 0 |  |  |  |  | 0.427032 |
| Olsenella | 0.005171 | 0 | 0.00185 |  | 0.002485 | 0 | 0.001533 |  |  |  |  | 0.064122 |
| Schwartzia | 0.005121 | 2.962153 | 0.334873 |  | 0.001682 | 0.781735 | 0.154096 |  |  |  |  | 0.003586 |
| Effusibacillus | 0.005063 | 0.002304 | 0.00601 |  | 0.001694 | 0.001347 | 0.002355 |  |  |  |  | 0.231055 |
| Anaerofustis | 0.004872 | 0.003975 | 0.000504 |  | 0.002049 | 0.002178 | 0.000504 |  |  |  |  | 0.770289 |
| Sphingomonas_B | 0.004777 | 0.007102 | 0.003895 |  | 0.001207 | 0.001588 | 0.00135 |  |  |  |  | 0.270755 |
| Acinetobacter | 0.004672 | 0.005139 | 0.163494 |  | 0.001793 | 0.002453 | 0.13658 |  |  |  |  | 0.880918 |
| Symbiobacterium | 0.004525 | 0.005514 | 0.003169 |  | 0.001435 | 0.001275 | 0.001459 |  |  |  |  | 0.617784 |
| UBA3006 | 0.004326 | 0.005047 | 0.009261 |  | 0.000464 | 0.000872 | 0.004138 |  |  |  |  | 0.482517 |
| Defluviitalea | 0.004281 | 0.002834 | 0.000504 |  | 0.002616 | 0.001752 | 0.000504 |  |  |  |  | 0.655643 |
| Anaerobiospirillum_A | 0.004272 | 1.625579 | 0.043895 |  | 0.001582 | 0.424722 | 0.031319 |  |  |  |  | 0.003388 |
| Coprobacter | 0.004095 | 0 | 0 |  | 0.001622 | 0 | 0 |  |  |  |  | 0.030132 |
| Mesotoga | 0.004094 | 0.007004 | 0 |  | 0.001292 | 0.005414 | 0 |  |  |  |  | 0.612489 |
| Rodentibacter | 0.004037 | 0.021776 | 0.005267 |  | 0.002245 | 0.005814 | 0.002138 |  |  |  |  | 0.017367 |
| Pseudomonas_E | 0.004035 | 0.017414 | 2.025475 |  | 0.001164 | 0.005173 | 2.019755 |  |  |  |  | 0.030221 |
| Ruminiclostridium | 0.003612 | 0.002527 | 0.001025 |  | 0.002038 | 0.002024 | 0.000648 |  |  |  |  | 0.713579 |
| Microcystis | 0.003539 | 0.00657 | 0.006045 |  | 0.000907 | 0.00244 | 0.001764 |  |  |  |  | 0.271257 |
| Bacillus_W | 0.003509 | 0.00181 | 0.006423 |  | 0.001912 | 0.000579 | 0.001907 |  |  |  |  | 0.414879 |
| Savagella | 0.003474 | 0.000885 | 0.000504 |  | 0.002199 | 0.000562 | 0.000504 |  |  |  |  | 0.280536 |
| UBA1174 | 0.003331 | 0.000875 | 0 |  | 0.001879 | 0.000555 | 0 |  |  |  |  | 0.238693 |
| Actinomyces_I | 0.003317 | 0.004214 | 0.005259 |  | 0.001462 | 0.001591 | 0.003354 |  |  |  |  | 0.686682 |
| Serinibacter | 0.00304 | 0.006841 | 0.004425 |  | 0.001013 | 0.002029 | 0.001299 |  |  |  |  | 0.12473 |
| UBA6659 | 0.003005 | 0.000889 | 0.000752 |  | 0.000816 | 0.000564 | 0.000496 |  |  |  |  | 0.058682 |
| Lachnotalea | 0.002893 | 0 | 0 |  | 0.00157 | 0 | 0 |  |  |  |  | 0.09515 |
| Clostridium | 0.002838 | 0.001242 | 0.032699 |  | 0.001863 | 0.001242 | 0.024944 |  |  |  |  | 0.492227 |
| Butyrivibrio | 0.002776 | 0.001832 | 0 |  | 0.002308 | 0.001392 | 0 |  |  |  |  | 0.733259 |
| MS4 | 0.002644 | 0.057728 | 0.009831 |  | 0.000956 | 0.015685 | 0.004294 |  |  |  |  | 0.005675 |
| T78 | 0.002641 | 0.003016 | 0.004076 |  | 0.001602 | 0.001657 | 0.001759 |  |  |  |  | 0.873724 |
| Pectobacterium | 0.002558 | 0.003938 | 0.001378 |  | 0.001147 | 0.002039 | 0.000623 |  |  |  |  | 0.568193 |
| Phyllobacterium | 0.00231 | 0.001347 | 0.013945 |  | 0.00093 | 0.000604 | 0.011633 |  |  |  |  | 0.405776 |
| Actinomadura_B | 0.002252 | 0.001504 | 0.001437 |  | 0.000458 | 0.001046 | 0.000968 |  |  |  |  | 0.52722 |
| Acetoanaerobium | 0.002226 | 0.001344 | 0 |  | 0.001264 | 0.000884 | 0 |  |  |  |  | 0.580188 |
| Steroidobacter | 0.002191 | 0.002274 | 0.001402 |  | 0.001055 | 0.00132 | 0.000938 |  |  |  |  | 0.961845 |
| Staphylococcus_A | 0.002114 | 0 | 0.069123 |  | 0.001141 | 0 | 0.044313 |  |  |  |  | 0.093519 |
| Hyphomicrobium | 0.002056 | 0.003219 | 0.001546 |  | 0.000983 | 0.001271 | 0.001063 |  |  |  |  | 0.485892 |
| QKVK01 | 0.00182 | 0.003308 | 0.000807 |  | 0.000882 | 0.001597 | 0.000542 |  |  |  |  | 0.433894 |
| CAG-791 | 0.001763 | 0.036824 | 0.004006 |  | 0.001323 | 0.013635 | 0.002103 |  |  |  |  | 0.028399 |
| Faecalibacterium | 0.001642 | 0.002708 | 0.010931 |  | 0.00104 | 0.000733 | 0.010338 |  |  |  |  | 0.42199 |
| Tolypothrix_B | 0.001606 | 0.002794 | 0.001492 |  | 0.00117 | 0.001031 | 0.000669 |  |  |  |  | 0.463955 |
| Aquamicrobium_A | 0.00141 | 0.001361 | 0.058415 |  | 0.000939 | 0.000611 | 0.029253 |  |  |  |  | 0.966045 |
| Lactobacillus_F | 0.001366 | 0.00193 | 0.004772 |  | 0.000616 | 0.001405 | 0.001924 |  |  |  |  | 0.720507 |
| Thiobacillus | 0.001366 | 0.000923 | 0.001075 |  | 0.000616 | 0.000923 | 0.000682 |  |  |  |  | 0.698328 |
| Agrobacterium | 0.001315 | 0.006386 | 0.545879 |  | 0.000588 | 0.00112 | 0.539579 |  |  |  |  | 0.002484 |
| UBA1777 | 0.001315 | 0.176698 | 0.035049 |  | 0.000588 | 0.020304 | 0.022144 |  |  |  |  | 6E-06 |
| GW-Nitrospira-1 | 0.001315 | 0.003169 | 0 |  | 0.000588 | 0.000873 | 0 |  |  |  |  | 0.108631 |
| Longilinea | 0.001297 | 0.001865 | 0.003972 |  | 0.00058 | 0.001 | 0.002496 |  |  |  |  | 0.634147 |
| Actinomyces | 0.001279 | 0.004998 | 0.482098 |  | 0.000574 | 0.001364 | 0.315237 |  |  |  |  | 0.030754 |
| UBA8950 | 0.001261 | 0.00263 | 0.001324 |  | 0.000566 | 0.001396 | 0.000594 |  |  |  |  | 0.384658 |
| RC9 | 0.00126 | 0.299589 | 0.077114 |  | 0.000565 | 0.071413 | 0.062201 |  |  |  |  | 0.001896 |
| Fermentibacter | 0.001253 | 0.001877 | 0.00311 |  | 0.000562 | 0.000944 | 0.001191 |  |  |  |  | 0.582721 |
| Eubacterium_C | 0.001224 | 0.019145 | 0.004497 |  | 0.000819 | 0.004761 | 0.003383 |  |  |  |  | 0.004044 |
| Butyricicoccus_A | 0.000963 | 0.003208 | 0.00142 |  | 0.000611 | 0.001291 | 0.000636 |  |  |  |  | 0.146979 |
| Bacillus | 0.000955 | 0 | 0.047954 |  | 0.000607 | 0 | 0.035191 |  |  |  |  | 0.146655 |
| Bradyrhizobium | 0.000886 | 0.001352 | 0.012277 |  | 0.000561 | 0.00089 | 0.006132 |  |  |  |  | 0.667429 |
| GCA-900066905 | 0.000876 | 0 | 0.000504 |  | 0.000554 | 0 | 0.000504 |  |  |  |  | 0.145048 |
| Zag1 | 0.000868 | 0.072148 | 0.019743 |  | 0.000549 | 0.026003 | 0.010819 |  |  |  |  | 0.020809 |
| Flavisolibacter | 0.000857 | 0.000828 | 0 |  | 0.000857 | 0.000828 | 0 |  |  |  |  | 0.980898 |
| Reyranella | 0.00085 | 0.000418 | 0.008537 |  | 0.000538 | 0.000418 | 0.005968 |  |  |  |  | 0.53982 |
| Alicyclobacillus_B | 0.000832 | 0.001921 | 0.001361 |  | 0.000527 | 0.000609 | 0.000613 |  |  |  |  | 0.206194 |
| Aminicenans | 0.000832 | 0.002275 | 0.000449 |  | 0.000527 | 0.00107 | 0.000449 |  |  |  |  | 0.254162 |
| Citrobacter | 0.000821 | 0 | 0.041409 |  | 0.00052 | 0 | 0.031496 |  |  |  |  | 0.145452 |
| Massilia | 0.000821 | 0.000418 | 0.00782 |  | 0.00052 | 0.000418 | 0.00782 |  |  |  |  | 0.558964 |
| Methanothrix | 0.000821 | 0.000889 | 0.001219 |  | 0.00052 | 0.000564 | 0.00092 |  |  |  |  | 0.931089 |
| Bacillus_C | 0.000814 | 0.00093 | 0.022456 |  | 0.000515 | 0.000594 | 0.012509 |  |  |  |  | 0.885133 |
| Anaerocolumna | 0.000516 | 0.005168 | 0.002536 |  | 0.000516 | 0.003364 | 0.001978 |  |  |  |  | 0.201581 |
| URHD0088 | 0.000516 | 0.002222 | 0.000449 |  | 0.000516 | 0.00106 | 0.000449 |  |  |  |  | 0.178314 |
| UBA1711 | 0.000447 | 0.114237 | 0.016762 |  | 0.000447 | 0.034066 | 0.011954 |  |  |  |  | 0.007491 |
| Sphingopyxis | 0.000439 | 0 | 0.049006 |  | 0.000439 | 0 | 0.049006 |  |  |  |  | 0.340893 |
| Brevibacterium | 0.000439 | 0 | 0.045601 |  | 0.000439 | 0 | 0.031401 |  |  |  |  | 0.340893 |
| Pannonibacter | 0.000439 | 0 | 0.030741 |  | 0.000439 | 0 | 0.030141 |  |  |  |  | 0.340893 |
| Clostridium_C | 0.000439 | 0 | 0.014076 |  | 0.000439 | 0 | 0.014076 |  |  |  |  | 0.340893 |
| Lentimicrobium | 0.000439 | 0.068304 | 0.009803 |  | 0.000439 | 0.018812 | 0.008933 |  |  |  |  | 0.004796 |
| Halomonas_D | 0.000439 | 0.000988 | 0.000979 |  | 0.000439 | 0.000626 | 0.000632 |  |  |  |  | 0.489269 |
| Alicyclobacillus_H | 0.000439 | 0.001404 | 0.000875 |  | 0.000439 | 0.000963 | 0.000555 |  |  |  |  | 0.383479 |
| Bog-159 | 0.000439 | 0 | 0.000504 |  | 0.000439 | 0 | 0.000504 |  |  |  |  | 0.340893 |
| Tatlockia | 0.000429 | 0.000461 | 0.123788 |  | 0.000429 | 0.000461 | 0.122886 |  |  |  |  | 0.959415 |
| Psychrobacter | 0.000429 | 0.005074 | 0.030083 |  | 0.000429 | 0.003611 | 0.014353 |  |  |  |  | 0.230274 |
| Tissierella_A | 0.000429 | 0 | 0.000735 |  | 0.000429 | 0 | 0.000482 |  |  |  |  | 0.340893 |
| Gracilibacter | 0.000429 | 0.001395 | 0 |  | 0.000429 | 0.000949 | 0 |  |  |  |  | 0.375273 |
| Megasphaera | 0.000421 | 0 | 0.20256 |  | 0.000421 | 0 | 0.200973 |  |  |  |  | 0.340893 |
| CAG-822 | 0.000421 | 0.040992 | 0.03 |  | 0.000421 | 0.023706 | 0.022628 |  |  |  |  | 0.117832 |
| Cryobacterium_A | 0.000421 | 0.000516 | 0.010447 |  | 0.000421 | 0.000516 | 0.005362 |  |  |  |  | 0.889518 |
| Zag111 | 0.000392 | 0.005306 | 0.00241 |  | 0.000392 | 0.001217 | 0.001596 |  |  |  |  | 0.003258 |
| CAG-988 | 0.000392 | 0.002996 | 0.001961 |  | 0.000392 | 0.002532 | 0.001488 |  |  |  |  | 0.333512 |
| Propionicicella | 0.000392 | 0.001765 | 0.001043 |  | 0.000392 | 0.000561 | 0.001043 |  |  |  |  | 0.072831 |
| UBA12465 | 0.000392 | 0.002348 | 0.000286 |  | 0.000392 | 0.00135 | 0.000286 |  |  |  |  | 0.194464 |
| Oligella | 0 | 0.000461 | 0.237122 |  | 0 | 0.000461 | 0.113797 |  |  |  |  | 0.340893 |
| Paenalcaligenes | 0 | 0.000471 | 0.176411 |  | 0 | 0.000471 | 0.100797 |  |  |  |  | 0.340893 |
| Advenella | 0 | 0 | 0.154837 |  | 0 | 0 | 0.154837 |  |  |  |  | #DIV/0! |
| Clostridium_S | 0 | 0.000472 | 0.093265 |  | 0 | 0.000472 | 0.092706 |  |  |  |  | 0.340893 |
| Devosia | 0 | 0 | 0.084088 |  | 0 | 0 | 0.059567 |  |  |  |  | #DIV/0! |
| UBA1547 | 0 | 0.303324 | 0.082784 |  | 0 | 0.054937 | 0.040151 |  |  |  |  | 0.000254 |
| Dakarella | 0 | 0 | 0.079529 |  | 0 | 0 | 0.079187 |  |  |  |  | #DIV/0! |
| Hydrogenophaga | 0 | 0.000418 | 0.078722 |  | 0 | 0.000418 | 0.078722 |  |  |  |  | 0.340893 |
| Oceanisphaera | 0 | 0 | 0.066123 |  | 0 | 0 | 0.040394 |  |  |  |  | #DIV/0! |
| Dietzia | 0 | 0 | 0.061301 |  | 0 | 0 | 0.028205 |  |  |  |  | #DIV/0! |
| Virgibacillus | 0 | 0 | 0.058276 |  | 0 | 0 | 0.031105 |  |  |  |  | #DIV/0! |
| Lysinibacillus_D | 0 | 0 | 0.056417 |  | 0 | 0 | 0.051957 |  |  |  |  | #DIV/0! |
| Clostridium_B | 0 | 0 | 0.044835 |  | 0 | 0 | 0.044835 |  |  |  |  | #DIV/0! |
| Brachybacterium | 0 | 0.000885 | 0.037711 |  | 0 | 0.000562 | 0.021935 |  |  |  |  | 0.146059 |
| Bacillus_A | 0 | 0 | 0.035387 |  | 0 | 0 | 0.034218 |  |  |  |  | #DIV/0! |
| Paracoccus | 0 | 0 | 0.032583 |  | 0 | 0 | 0.018235 |  |  |  |  | #DIV/0! |
| Luteimonas | 0 | 0 | 0.024649 |  | 0 | 0 | 0.018355 |  |  |  |  | #DIV/0! |
| Pusillimonas | 0 | 0 | 0.023992 |  | 0 | 0 | 0.011678 |  |  |  |  | #DIV/0! |
| Eubacterium_F | 0 | 0.072846 | 0.023935 |  | 0 | 0.034287 | 0.015207 |  |  |  |  | 0.059562 |
| Jeotgalicoccus | 0 | 0 | 0.023898 |  | 0 | 0 | 0.014591 |  |  |  |  | #DIV/0! |
| Nosocomiicoccus | 0 | 0 | 0.02111 |  | 0 | 0 | 0.012257 |  |  |  |  | #DIV/0! |
| Lysinibacillus_A | 0 | 0.00093 | 0.019174 |  | 0 | 0.000594 | 0.011531 |  |  |  |  | 0.148192 |
| Brevundimonas | 0 | 0 | 0.018761 |  | 0 | 0 | 0.009404 |  |  |  |  | #DIV/0! |
| Bacillus_J | 0 | 0 | 0.018126 |  | 0 | 0 | 0.013972 |  |  |  |  | #DIV/0! |
| Halomonas | 0 | 0 | 0.016402 |  | 0 | 0 | 0.010636 |  |  |  |  | #DIV/0! |
| Clostridium_AM | 0 | 0.000471 | 0.013555 |  | 0 | 0.000471 | 0.013555 |  |  |  |  | 0.340893 |
| Yaniella | 0 | 0.000461 | 0.013404 |  | 0 | 0.000461 | 0.008516 |  |  |  |  | 0.340893 |
| Eggerthella | 0 | 0.024797 | 0.013157 |  | 0 | 0.005767 | 0.006623 |  |  |  |  | 0.001561 |
| Dialister | 0 | 0 | 0.012512 |  | 0 | 0 | 0.012512 |  |  |  |  | #DIV/0! |
| UBA6382 | 0 | 0 | 0.012512 |  | 0 | 0 | 0.012512 |  |  |  |  | #DIV/0! |
| Denitrobacterium | 0 | 0.039083 | 0.012156 |  | 0 | 0.008032 | 0.006668 |  |  |  |  | 0.000655 |
| CDF | 0 | 0 | 0.012086 |  | 0 | 0 | 0.009493 |  |  |  |  | #DIV/0! |
| Pseudomonas_A | 0 | 0 | 0.011386 |  | 0 | 0 | 0.005328 |  |  |  |  | #DIV/0! |
| Pediococcus | 0 | 0 | 0.011333 |  | 0 | 0 | 0.009721 |  |  |  |  | #DIV/0! |
| Bosea | 0 | 0 | 0.011261 |  | 0 | 0 | 0.00911 |  |  |  |  | #DIV/0! |
| Lactobacillus_E | 0 | 0 | 0.009905 |  | 0 | 0 | 0.009905 |  |  |  |  | #DIV/0! |
| Facklamia | 0 | 0.000472 | 0.009316 |  | 0 | 0.000472 | 0.006971 |  |  |  |  | 0.340893 |
| Sporolactobacillus | 0 | 0.000418 | 0.008863 |  | 0 | 0.000418 | 0.008863 |  |  |  |  | 0.340893 |
| Acetobacter | 0 | 0 | 0.008341 |  | 0 | 0 | 0.008341 |  |  |  |  | #DIV/0! |
| Ralstonia | 0 | 0.000418 | 0.008244 |  | 0 | 0.000418 | 0.004735 |  |  |  |  | 0.340893 |
| Achromobacter | 0 | 0 | 0.006777 |  | 0 | 0 | 0.006777 |  |  |  |  | #DIV/0! |
| UBA945 | 0 | 0.021575 | 0.006421 |  | 0 | 0.009471 | 0.003838 |  |  |  |  | 0.045944 |
| SZUA-55 | 0 | 0 | 0.006418 |  | 0 | 0 | 0.003547 |  |  |  |  | #DIV/0! |
| UBA2365 | 0 | 0 | 0.006256 |  | 0 | 0 | 0.006256 |  |  |  |  | #DIV/0! |
| Atopostipes | 0 | 0 | 0.00602 |  | 0 | 0 | 0.005684 |  |  |  |  | #DIV/0! |
| Beta-01 | 0 | 0 | 0.005846 |  | 0 | 0 | 0.003023 |  |  |  |  | #DIV/0! |
| Phycicoccus | 0 | 0.000471 | 0.005663 |  | 0 | 0.000471 | 0.004536 |  |  |  |  | 0.340893 |
| Brevibacillus | 0 | 0.000516 | 0.005246 |  | 0 | 0.000516 | 0.00289 |  |  |  |  | 0.340893 |
| Brachyspira | 0 | 0 | 0.005213 |  | 0 | 0 | 0.005213 |  |  |  |  | #DIV/0! |
| Ruminiclostridium_D | 0 | 0 | 0.005213 |  | 0 | 0 | 0.005213 |  |  |  |  | #DIV/0! |
| Anaerovibrio | 0 | 0.023056 | 0.004534 |  | 0 | 0.015948 | 0.004534 |  |  |  |  | 0.178858 |
| Lactobacillus_G | 0 | 0 | 0.004171 |  | 0 | 0 | 0.004171 |  |  |  |  | #DIV/0! |
| Solobacterium | 0 | 0 | 0.004171 |  | 0 | 0 | 0.004171 |  |  |  |  | #DIV/0! |
| F0428 | 0 | 0 | 0.003913 |  | 0 | 0 | 0.002493 |  |  |  |  | #DIV/0! |
| Comamonas_D | 0 | 0 | 0.003649 |  | 0 | 0 | 0.003649 |  |  |  |  | #DIV/0! |
| Flavobacterium | 0 | 0 | 0.003649 |  | 0 | 0 | 0.003649 |  |  |  |  | #DIV/0! |
| JJ008 | 0 | 0 | 0.003649 |  | 0 | 0 | 0.003649 |  |  |  |  | #DIV/0! |
| Eubacterium_A | 0 | 0.005401 | 0.00347 |  | 0 | 0.002427 | 0.001463 |  |  |  |  | 0.050208 |
| Pelagibacterium | 0 | 0 | 0.003232 |  | 0 | 0 | 0.001494 |  |  |  |  | #DIV/0! |
| Nocardiopsis | 0 | 0 | 0.003178 |  | 0 | 0 | 0.002555 |  |  |  |  | #DIV/0! |
| 21-14-0-10-47-8-A | 0 | 0 | 0.003128 |  | 0 | 0 | 0.003128 |  |  |  |  | #DIV/0! |
| Methylocystis | 0 | 0.00182 | 0.003128 |  | 0 | 0.000583 | 0.003128 |  |  |  |  | 0.01083 |
| Flavobacterium_A | 0 | 0 | 0.002892 |  | 0 | 0 | 0.002565 |  |  |  |  | #DIV/0! |
| Desulfuromonas_B | 0 | 0 | 0.00282 |  | 0 | 0 | 0.001992 |  |  |  |  | #DIV/0! |
| Listeria | 0 | 0 | 0.002806 |  | 0 | 0 | 0.002239 |  |  |  |  | #DIV/0! |
| Clostridium_H | 0 | 0 | 0.002607 |  | 0 | 0 | 0.002607 |  |  |  |  | #DIV/0! |
| Pedobacter | 0 | 0.028185 | 0.002519 |  | 0 | 0.010756 | 0.002519 |  |  |  |  | 0.025581 |
| Gemmata | 0 | 0 | 0.001564 |  | 0 | 0 | 0.001564 |  |  |  |  | #DIV/0! |
| Pseudochrobactrum | 0 | 0 | 0.001564 |  | 0 | 0 | 0.001564 |  |  |  |  | #DIV/0! |
| Bact-08 | 0 | 0.012766 | 0.001348 |  | 0 | 0.004588 | 0.001348 |  |  |  |  | 0.019363 |
| Caldilinea | 0 | 0 | 0.001043 |  | 0 | 0 | 0.001043 |  |  |  |  | #DIV/0! |
| Intestinibacillus | 0 | 0.002742 | 0.001025 |  | 0 | 0.000721 | 0.000648 |  |  |  |  | 0.003462 |
| Clostridium_A | 0 | 0.005509 | 0.001008 |  | 0 | 0.000995 | 0.001008 |  |  |  |  | 0.000248 |
| ZCTH02-B6 | 0 | 0 | 0.000971 |  | 0 | 0 | 0.000614 |  |  |  |  | #DIV/0! |
| 33-17 | 0 | 0.001802 | 0.000916 |  | 0 | 0.001364 | 0.000579 |  |  |  |  | 0.215764 |
| Nioella | 0 | 0 | 0.000571 |  | 0 | 0 | 0.000571 |  |  |  |  | #DIV/0! |
| Arboricoccus | 0 | 0.001966 | 0.000521 |  | 0 | 0.001015 | 0.000521 |  |  |  |  | 0.081443 |
| Veillonella | 0 | 0.005078 | 0.000449 |  | 0 | 0.002086 | 0.000449 |  |  |  |  | 0.035168 |
| UBA7182 | 0 | 0.001392 | 0 |  | 0 | 0.000628 | 0 |  |  |  |  | 0.050858 |
| Acetatifactor | 0 | 0.002816 | 0 |  | 0 | 0.00179 | 0 |  |  |  |  | 0.146852 |
| Globicatella | 0 | 0.003724 | 0 |  | 0 | 0.001707 | 0 |  |  |  |  | 0.054104 |

## S8.6（Fig 8F）

| species | WT | Uox-/--1 | Uox-/--2 | SE | WT | Uox-/--1 | Uox-/--2 | total (top10) | |  |  | T-test Uox-/--2 vs Uox-/--1 |
| --- | --- | --- | --- | --- | --- | --- | --- | --- | --- | --- | --- | --- |
| Vibrio_parahaemolyticus | 24.39686 | 5.85271 | 1.328305 |  | 2.245935 | 1.190791 | 0.931259 | 76.51157 | 48.43611 | 70.04893 |  | 2.62E-05 |
| Cronobacter_sakazakii | 14.83901 | 11.30718 | 1.95147 |  | 2.205823 | 0.763839 | 1.088 |  |  |  |  | 0.161225 |
| Cronobacter_malonaticus | 11.31719 | 2.142303 | 1.85385 |  | 4.759817 | 0.59238 | 0.654296 |  |  |  |  | 0.08481 |
| Lactobacillus_johnsonii | 7.279722 | 16.24927 | 24.14896 |  | 1.999758 | 1.366364 | 8.564763 |  |  |  |  | 0.004086 |
| Lactobacillus_B_apodemi | 5.07761 | 0.936586 | 19.37363 |  | 1.725464 | 0.271914 | 9.268771 |  |  |  |  | 0.039231 |
| Salmonella_enterica | 3.822267 | 3.433833 | 0.884485 |  | 0.643055 | 0.258176 | 0.528705 |  |  |  |  | 0.587439 |
| Cronobacter_dublinensis | 3.445401 | 1.995354 | 0.197916 |  | 0.589697 | 0.92065 | 0.069683 |  |  |  |  | 0.214239 |
| Lactobacillus_H_vaginalis_A | 2.679597 | 4.546617 | 19.86643 |  | 0.851359 | 1.024165 | 3.265204 |  |  |  |  | 0.191224 |
| Chryseobacterium_sp002899825 | 1.995499 | 0.337858 | 0.062458 |  | 0.625846 | 0.142113 | 0.041719 |  |  |  |  | 0.027281 |
| \| Eubacterium_R_sp000436835 \| \| --- \| | 1.658415 | 1.634401 | 0.381425 |  | 0.62254 | 0.532245 | 0.20445 |  |  |  |  | 0.977187 |
| Duncaniella_muris | 1.5265 | 0.530262 | 0.089861 |  | 0.251642 | 0.115262 | 0.03701 |  |  |  |  | 0.004853 |
| Prevotella_conceptionensis | 1.292379 | 5.395989 | 0.338931 |  | 0.645786 | 2.729355 | 0.17133 |  |  |  |  | 0.174146 |
| 992a_sp001940245 | 1.260169 | 0.835412 | 0.083304 |  | 0.681855 | 0.205217 | 0.055149 |  |  |  |  | 0.564093 |
| Kineothrix_alysoides | 0.976608 | 4.002234 | 0.439242 |  | 0.569197 | 1.166431 | 0.203784 |  |  |  |  | 0.041966 |
| Megamonas_hypermegale | 0.805916 | 0.002753 | 0.004148 |  | 0.367941 | 0.000737 | 0.001283 |  |  |  |  | 0.053985 |
| Bacteroides_acidifaciens | 0.73305 | 0.004933 | 0.002281 |  | 0.159332 | 0.001253 | 0.000953 |  |  |  |  | 0.001027 |
| Treponema_D_sp900316905 | 0.731604 | 0.522849 | 0.1219 |  | 0.466959 | 0.255066 | 0.102371 |  |  |  |  | 0.703042 |
| Blastococcus_sp003075095 | 0.715082 | 0.002658 | 0.001546 |  | 0.670106 | 0.001123 | 0.001063 |  |  |  |  | 0.312713 |
| Blautia_A_sp900066165 | 0.621081 | 0.016202 | 0.006475 |  | 0.339608 | 0.001823 | 0.004785 |  |  |  |  | 0.105242 |
| Ruminococcus_A_sp003011855 | 0.564536 | 0.056094 | 0.009674 |  | 0.427071 | 0.015826 | 0.006621 |  |  |  |  | 0.261639 |
| Eubacterium_R_coprostanoligenes | 0.518538 | 0.117839 | 0.015203 |  | 0.423447 | 0.035192 | 0.005373 |  |  |  |  | 0.367891 |
| Clostridium_Q_saccharolyticum | 0.518148 | 0.210576 | 0.045469 |  | 0.320276 | 0.046066 | 0.029941 |  |  |  |  | 0.364239 |
| Enterococcus_rotai | 0.517927 | 0.762263 | 1.239286 |  | 0.029758 | 0.081287 | 0.20999 |  |  |  |  | 0.018079 |
| OEMS01_sp900199405 | 0.515374 | 0.333912 | 0.167147 |  | 0.143895 | 0.051509 | 0.116258 |  |  |  |  | 0.262548 |
| Bacteroides_B_sartorii | 0.436871 | 0.071524 | 0.016435 |  | 0.108483 | 0.026658 | 0.010394 |  |  |  |  | 0.008423 |
| Anaerostipes_hadrus | 0.423704 | 0.176292 | 0.0644 |  | 0.167902 | 0.079471 | 0.032302 |  |  |  |  | 0.212454 |
| CAG-217_sp000436335 | 0.422143 | 0.09824 | 0.088391 |  | 0.221969 | 0.054396 | 0.085106 |  |  |  |  | 0.186793 |
| Virgibacillus_G_profundi | 0.374843 | 0.166583 | 0.015986 |  | 0.249001 | 0.068233 | 0.010838 |  |  |  |  | 0.438635 |
| Desulfovibrio_fairfieldensis | 0.337052 | 0.716295 | 0.172629 |  | 0.134763 | 0.218416 | 0.129458 |  |  |  |  | 0.170278 |
| Ruminiclostridium_E_siraeum | 0.297596 | 1.16605 | 0.375311 |  | 0.141386 | 0.431681 | 0.337253 |  |  |  |  | 0.084943 |
| Prevotellamassilia_sp002933955 | 0.285295 | 0.173008 | 0.031699 |  | 0.139097 | 0.040567 | 0.01853 |  |  |  |  | 0.456287 |
| Vallitalea_guaymasensis | 0.256016 | 0.02479 | 0.009896 |  | 0.070573 | 0.012985 | 0.006141 |  |  |  |  | 0.009139 |
| Agathobacter_ruminis | 0.244928 | 0.921727 | 0.067071 |  | 0.141574 | 0.263182 | 0.05681 |  |  |  |  | 0.04699 |
| Flavonifractor_sp002161085 | 0.239366 | 0.728897 | 0.104534 |  | 0.043593 | 0.13727 | 0.074024 |  |  |  |  | 0.006783 |
| Parasutterella_sp000980495 | 0.238589 | 0.000414 | 0.000467 |  | 0.072869 | 0.000414 | 0.000467 |  |  |  |  | 0.008452 |
| Faecalicatena_sp000403295 | 0.237949 | 0.124889 | 0.020396 |  | 0.127499 | 0.048291 | 0.01131 |  |  |  |  | 0.426305 |
| Helicobacter_C_typhlonius | 0.237841 | 0.000885 | 0.001075 |  | 0.093783 | 0.000562 | 0.000682 |  |  |  |  | 0.030046 |
| Lactobacillus_intestinalis | 0.224062 | 0.471647 | 0.218685 |  | 0.066202 | 0.122766 | 0.107764 |  |  |  |  | 0.106272 |
| Parasutterella_excrementihominis | 0.212064 | 0.000875 | 0.000449 |  | 0.0718 | 0.000555 | 0.000449 |  |  |  |  | 0.014753 |
| Bacteroides_F_pectinophilus | 0.209017 | 0.32962 | 0.10681 |  | 0.153141 | 0.196605 | 0.061321 |  |  |  |  | 0.63885 |
| 14-2_sp000403255 | 0.190862 | 0.14104 | 0.0096 |  | 0.173258 | 0.067632 | 0.004502 |  |  |  |  | 0.79424 |
| Eubacterium_Q_ruminantium_A | 0.186974 | 0.339109 | 0.115623 |  | 0.090569 | 0.138463 | 0.096655 |  |  |  |  | 0.379475 |
| Saccharimonas_aalborgensis | 0.184007 | 2.926729 | 1.04078 |  | 0.055071 | 0.457254 | 0.615412 |  |  |  |  | 0.00014 |
| Bacteroides_B_dorei | 0.167003 | 0.000933 | 0 |  | 0.145638 | 0.00059 | 0 |  |  |  |  | 0.280744 |
| Romboutsia_timonensis | 0.164651 | 0.010676 | 0.05933 |  | 0.090785 | 0.003922 | 0.037568 |  |  |  |  | 0.121042 |
| Lawsonibacter_asaccharolyticus | 0.16284 | 0.340222 | 0.024871 |  | 0.044482 | 0.103256 | 0.017015 |  |  |  |  | 0.14571 |
| Parabacteroides_distasonis | 0.159535 | 0.001823 | 0.002013 |  | 0.032665 | 0.000932 | 0.001538 |  |  |  |  | 0.000696 |
| Klebsiella_pneumoniae | 0.155949 | 0.186751 | 0.493665 |  | 0.024196 | 0.018532 | 0.266167 |  |  |  |  | 0.336007 |
| Lawsonibacter_sp000492175 | 0.143333 | 1.051731 | 0.155922 |  | 0.039064 | 0.223105 | 0.101202 |  |  |  |  | 0.002476 |
| UBA2730_sp900320505 | 0.137707 | 0.263277 | 0.117429 |  | 0.058239 | 0.100777 | 0.064134 |  |  |  |  | 0.306002 |
| Provencibacterium_massiliense | 0.13656 | 0.057218 | 0.020453 |  | 0.032334 | 0.01089 | 0.01773 |  |  |  |  | 0.042376 |
| Ruminococcus_C_sp000433635 | 0.135638 | 0.12937 | 0.0375 |  | 0.060694 | 0.031741 | 0.026759 |  |  |  |  | 0.928891 |
| Intestinimonas_butyriciproducens | 0.134298 | 0.392038 | 0.087217 |  | 0.071032 | 0.118426 | 0.040656 |  |  |  |  | 0.091549 |
| CAG-41_sp001941225 | 0.131674 | 0.06116 | 0.03593 |  | 0.07106 | 0.015054 | 0.022154 |  |  |  |  | 0.354561 |
| Adlercreutzia_equolifaciens | 0.131469 | 0.027051 | 0.00968 |  | 0.027065 | 0.00829 | 0.003406 |  |  |  |  | 0.004184 |
| CAG-1031_sp000431215 | 0.126137 | 0.032325 | 0.008957 |  | 0.017681 | 0.009914 | 0.005738 |  |  |  |  | 0.000939 |
| UC5-1-2E3_sp001304875 | 0.124022 | 0.071985 | 0.016461 |  | 0.063454 | 0.017963 | 0.007541 |  |  |  |  | 0.448379 |
| Oscillibacter_sp000403435 | 0.123374 | 0.195858 | 0.019371 |  | 0.055479 | 0.06004 | 0.015563 |  |  |  |  | 0.396075 |
| Kineothrix_sp000403275 | 0.11825 | 0.006395 | 0.000521 |  | 0.093964 | 0.001843 | 0.000521 |  |  |  |  | 0.261465 |
| Anaeromassilibacillus_senegalensis | 0.115991 | 0 | 0 |  | 0.018494 | 0 | 0 |  |  |  |  | 9.24E-05 |
| Dorea_sp000403475 | 0.099801 | 0.177862 | 0.039569 |  | 0.036512 | 0.043711 | 0.015752 |  |  |  |  | 0.200486 |
| Lawsonibacter_sp002161175 | 0.092686 | 1.065234 | 0.0453 |  | 0.026371 | 0.517279 | 0.020703 |  |  |  |  | 0.089865 |
| Emergencia_timonensis | 0.0894 | 0.020067 | 0.006628 |  | 0.00996 | 0.003669 | 0.004305 |  |  |  |  | 6.62E-05 |
| An181_sp002160325 | 0.087747 | 0 | 0 |  | 0.061286 | 0 | 0 |  |  |  |  | 0.182716 |
| Fournierella_massiliensis | 0.087497 | 0.102174 | 0.012409 |  | 0.035054 | 0.043289 | 0.007961 |  |  |  |  | 0.797514 |
| CAG-41_sp900066215 | 0.087329 | 1.187943 | 0.455985 |  | 0.016363 | 0.269624 | 0.257444 |  |  |  |  | 0.002234 |
| Providencia_stuartii_B | 0.086582 | 0.122445 | 0.097423 |  | 0.012326 | 0.015093 | 0.030638 |  |  |  |  | 0.095539 |
| Acutalibacter_timonensis | 0.086062 | 0.46528 | 0.132779 |  | 0.019499 | 0.151398 | 0.065544 |  |  |  |  | 0.032307 |
| Faecalibaculum_rodentium | 0.085548 | 0.000461 | 0.000286 |  | 0.061528 | 0.000461 | 0.000286 |  |  |  |  | 0.196807 |
| Anaerotruncus_sp000403395 | 0.082248 | 0.001406 | 0 |  | 0.026923 | 0.000633 | 0 |  |  |  |  | 0.013301 |
| CAG-81_sp900066785 | 0.081098 | 0.038625 | 0.016895 |  | 0.064316 | 0.009191 | 0.007385 |  |  |  |  | 0.528034 |
| GCA-900066575_sp900066385 | 0.075105 | 0.14014 | 0.02642 |  | 0.014966 | 0.035473 | 0.0177 |  |  |  |  | 0.122074 |
| Sutterella_wadsworthensis_B | 0.07256 | 0.333799 | 0.040911 |  | 0.020955 | 0.123463 | 0.029457 |  |  |  |  | 0.063544 |
| Butyrivibrio_A_crossotus | 0.072406 | 1.843429 | 0.542811 |  | 0.050323 | 0.727146 | 0.459676 |  |  |  |  | 0.035464 |
| Ruthenibacterium_lactatiformans | 0.069896 | 0.048852 | 0.009061 |  | 0.039777 | 0.020576 | 0.006141 |  |  |  |  | 0.6485 |
| Rothia_nasimurium | 0.067992 | 0.047912 | 10.64818 |  | 0.009987 | 0.010525 | 5.786504 |  |  |  |  | 0.196471 |
| UBA6985_sp900314465 | 0.065733 | 0.152304 | 0.031084 |  | 0.044697 | 0.040909 | 0.022036 |  |  |  |  | 0.183558 |
| COE1_sp000403335 | 0.058975 | 0.02677 | 0.002986 |  | 0.025097 | 0.010552 | 0.002468 |  |  |  |  | 0.264204 |
| UBA1394_sp900066845 | 0.058117 | 0.123922 | 0.067104 |  | 0.039489 | 0.032148 | 0.063384 |  |  |  |  | 0.225312 |
| Ruminiclostridium_C_viride | 0.054058 | 0.110696 | 0.018766 |  | 0.007595 | 0.012478 | 0.010421 |  |  |  |  | 0.003073 |
| Ruminiclostridium_A_cellulolyticum | 0.052911 | 0.152212 | 0.042373 |  | 0.045683 | 0.080954 | 0.025087 |  |  |  |  | 0.310505 |
| UBA2212_sp002402585 | 0.052325 | 0.008141 | 0.003648 |  | 0.019277 | 0.002594 | 0.002119 |  |  |  |  | 0.046446 |
| CAG-110_sp900315595 | 0.051898 | 0.011157 | 0.003217 |  | 0.01488 | 0.003777 | 0.001845 |  |  |  |  | 0.024156 |
| CAG-95_sp000403495 | 0.050661 | 0.390995 | 0.076822 |  | 0.024236 | 0.093797 | 0.049591 |  |  |  |  | 0.005603 |
| LD21_sp002428385 | 0.04903 | 0.034933 | 0.010492 |  | 0.008423 | 0.005283 | 0.003676 |  |  |  |  | 0.186661 |
| Anaerotruncus_colihominis | 0.048158 | 0.005589 | 0.001797 |  | 0.029864 | 0.00237 | 0.001481 |  |  |  |  | 0.185756 |
| Clostridium_N_fimetarium | 0.047879 | 0.041146 | 0.022228 |  | 0.027606 | 0.028711 | 0.015827 |  |  |  |  | 0.869135 |
| Prevotella_copri | 0.047028 | 2.728222 | 0.15983 |  | 0.006393 | 0.898128 | 0.076076 |  |  |  |  | 0.013685 |
| CAG-45_sp900066395 | 0.04657 | 0.000875 | 0 |  | 0.046036 | 0.000555 | 0 |  |  |  |  | 0.344353 |
| Eubacterium_J_plexicaudatum | 0.046134 | 0.010878 | 0.002482 |  | 0.03315 | 0.004427 | 0.001441 |  |  |  |  | 0.316605 |
| Ruminococcus_D_sp000686125 | 0.044444 | 0.01981 | 0.000449 |  | 0.030005 | 0.018751 | 0.000449 |  |  |  |  | 0.502156 |
| W-Firmicutes-11_sp002840285 | 0.04425 | 0.074756 | 0.018014 |  | 0.006966 | 0.009837 | 0.010721 |  |  |  |  | 0.029828 |
| Acutalibacter_muris | 0.043522 | 0.123598 | 0.020263 |  | 0.012961 | 0.026975 | 0.013005 |  |  |  |  | 0.023264 |
| Ruminococcus_D_sp900119155 | 0.043018 | 0.115016 | 0.038117 |  | 0.032283 | 0.036531 | 0.035315 |  |  |  |  | 0.170506 |
| Bacteroides_eggerthii | 0.042928 | 0.000418 | 0.000504 |  | 0.017541 | 0.000418 | 0.000504 |  |  |  |  | 0.035891 |
| MYbin3_sp002753335 | 0.042667 | 0.075836 | 0.034802 |  | 0.005102 | 0.009512 | 0.013013 |  |  |  |  | 0.011779 |
| Allobaculum_stercoricanis | 0.041971 | 0 | 0 |  | 0.027927 | 0 | 0 |  |  |  |  | 0.163773 |
| Methanosphaera_cuniculi | 0.039878 | 0 | 0 |  | 0.015722 | 0 | 0 |  |  |  |  | 0.029544 |
| CHKCI006_sp900018345 | 0.039037 | 0.005119 | 0.000449 |  | 0.038055 | 0.002626 | 0.000449 |  |  |  |  | 0.394789 |
| Flavonifractor_sp900199495 | 0.038398 | 0.245612 | 0.039523 |  | 0.023979 | 0.038857 | 0.014146 |  |  |  |  | 0.001078 |
| Christensenella_massiliensis | 0.035888 | 0.014418 | 0.005862 |  | 0.004807 | 0.002085 | 0.003825 |  |  |  |  | 0.002153 |
| Absiella_dolichum | 0.034351 | 0.0314 | 0.01722 |  | 0.027698 | 0.008484 | 0.007896 |  |  |  |  | 0.920882 |
| Adlercreutzia_caecimuris | 0.033854 | 0.006805 | 0.005321 |  | 0.010596 | 0.002737 | 0.002156 |  |  |  |  | 0.033016 |
| Marvinbryantia_sp900066075 | 0.033787 | 0.012902 | 0.002281 |  | 0.010836 | 0.006198 | 0.000929 |  |  |  |  | 0.125257 |
| Rikenella_massiliensis | 0.03296 | 0.344 | 0.089632 |  | 0.009878 | 0.150707 | 0.065976 |  |  |  |  | 0.066449 |
| Alistipes_putredinis | 0.030893 | 0.01625 | 0.007587 |  | 0.011885 | 0.007697 | 0.006373 |  |  |  |  | 0.325447 |
| Bifidobacterium_asteroides | 0.030864 | 0.039097 | 0.063603 |  | 0.004724 | 0.005247 | 0.008487 |  |  |  |  | 0.270631 |
| Robinsoniella_peoriensis | 0.030798 | 0.017124 | 0.003452 |  | 0.023437 | 0.00549 | 0.001822 |  |  |  |  | 0.582526 |
| 14-2_sp001940225 | 0.030438 | 0.031717 | 0.005557 |  | 0.007856 | 0.009481 | 0.003201 |  |  |  |  | 0.919351 |
| Sporobacter_termitidis | 0.02972 | 0.047395 | 0.016758 |  | 0.00652 | 0.006603 | 0.010889 |  |  |  |  | 0.08596 |
| Sphingomonas_A_sp003097155 | 0.028884 | 0.048637 | 0.032955 |  | 0.004384 | 0.006601 | 0.013226 |  |  |  |  | 0.031841 |
| Lachnospira_rogosae | 0.028289 | 0.000934 | 0 |  | 0.014251 | 0.000596 | 0 |  |  |  |  | 0.084109 |
| Pseudoflavonifractor_capillosus | 0.028173 | 0.005677 | 0.000504 |  | 0.013413 | 0.002397 | 0.000504 |  |  |  |  | 0.129736 |
| Bacteroides_uniformis | 0.027609 | 0.000886 | 0.000449 |  | 0.009709 | 0.000562 | 0.000449 |  |  |  |  | 0.020554 |
| Eisenbergiella_tayi | 0.027538 | 0.033745 | 0.003976 |  | 0.010164 | 0.008538 | 0.003465 |  |  |  |  | 0.650107 |
| Papillibacter_cinnamivorans | 0.027429 | 0.029856 | 0.005873 |  | 0.005969 | 0.007024 | 0.001602 |  |  |  |  | 0.797661 |
| Phascolarctobacterium_faecium | 0.027033 | 0.230478 | 0.052241 |  | 0.007199 | 0.095587 | 0.033399 |  |  |  |  | 0.059785 |
| Fournierella_sp002160145 | 0.026784 | 0.007514 | 0.000953 |  | 0.004327 | 0.004241 | 0.000604 |  |  |  |  | 0.009806 |
| Stoquefichus_sp001244545 | 0.0263 | 0.000471 | 0 |  | 0.007644 | 0.000471 | 0 |  |  |  |  | 0.00709 |
| Eubacterium_G_ventriosum | 0.026205 | 0.032369 | 0.000521 |  | 0.006901 | 0.009392 | 0.000521 |  |  |  |  | 0.608473 |
| Proteus_mirabilis | 0.025155 | 0.028275 | 0.028784 |  | 0.005933 | 0.003047 | 0.00879 |  |  |  |  | 0.649994 |
| Negativibacillus_massiliensis | 0.025121 | 0.006273 | 0.004534 |  | 0.014076 | 0.002999 | 0.004534 |  |  |  |  | 0.219624 |
| Bacteroides_intestinalis | 0.024984 | 0.170096 | 0.021911 |  | 0.008705 | 0.038806 | 0.009268 |  |  |  |  | 0.004472 |
| Parabacteroides_sp900155425 | 0.024387 | 0.000472 | 0 |  | 0.005293 | 0.000472 | 0 |  |  |  |  | 0.001142 |
| Massilimaliae_massiliensis | 0.024087 | 0.041665 | 0.011027 |  | 0.003596 | 0.019815 | 0.008222 |  |  |  |  | 0.403213 |
| TWA4_sp000875945 | 0.02369 | 0.015651 | 0.002536 |  | 0.008766 | 0.004385 | 0.001978 |  |  |  |  | 0.431235 |
| Marseille-P3106_sp900169975 | 0.023529 | 0.145507 | 0.037341 |  | 0.004563 | 0.016009 | 0.017959 |  |  |  |  | 2.52E-05 |
| Erysipelatoclostridium_sp000752095 | 0.021828 | 0.021921 | 0.005716 |  | 0.013291 | 0.009712 | 0.002657 |  |  |  |  | 0.995613 |
| Faecalicatena_sp000364245 | 0.021493 | 0.160439 | 0.016771 |  | 0.005029 | 0.067252 | 0.010046 |  |  |  |  | 0.066354 |
| Raoultibacter_timonensis | 0.021227 | 0.030816 | 0.016613 |  | 0.003335 | 0.005769 | 0.010074 |  |  |  |  | 0.180728 |
| Morganella_morganii_B | 0.021167 | 0.027977 | 0.021348 |  | 0.003665 | 0.002683 | 0.010412 |  |  |  |  | 0.164661 |
| Faecalicatena_orotica | 0.020829 | 0.148069 | 0.014832 |  | 0.010934 | 0.036185 | 0.008232 |  |  |  |  | 0.007169 |
| Coprococcus_B_comes | 0.019843 | 0.036101 | 0.005734 |  | 0.010499 | 0.013078 | 0.002203 |  |  |  |  | 0.355194 |
| Lawsonibacter_sp002160305 | 0.019544 | 0.0645 | 0.009903 |  | 0.007395 | 0.010799 | 0.003858 |  |  |  |  | 0.006386 |
| Bittarella_massiliensis | 0.019302 | 0 | 0.000504 |  | 0.004189 | 0 | 0.000504 |  |  |  |  | 0.000968 |
| Eubacterium_I_sp000270305 | 0.018898 | 0.001809 | 0 |  | 0.013242 | 0.000917 | 0 |  |  |  |  | 0.226939 |
| UBA1191_sp900066305 | 0.018503 | 0.035791 | 0.018378 |  | 0.005383 | 0.007701 | 0.006608 |  |  |  |  | 0.095626 |
| TF01-11_sp001414325 | 0.018167 | 0.096613 | 0.019493 |  | 0.006223 | 0.027636 | 0.013711 |  |  |  |  | 0.019814 |
| Meiothermus_B_silvanus | 0.018026 | 0.027453 | 0.018351 |  | 0.002118 | 0.002238 | 0.005908 |  |  |  |  | 0.012058 |
| Lachnoclostridium_A_edouardi | 0.018016 | 0.01253 | 0.002695 |  | 0.007418 | 0.003649 | 0.002695 |  |  |  |  | 0.52192 |
| Alistipes_A_sp900240235 | 0.017913 | 0.000418 | 0 |  | 0.011637 | 0.000418 | 0 |  |  |  |  | 0.163871 |
| Acetivibrio_cellulolyticus | 0.017632 | 0.002797 | 0.000953 |  | 0.005091 | 0.000719 | 0.000604 |  |  |  |  | 0.01624 |
| Dorea_formicigenerans | 0.017478 | 0.112364 | 0.015349 |  | 0.00549 | 0.018932 | 0.008657 |  |  |  |  | 0.000709 |
| CAG-878_sp000432255 | 0.017271 | 0.009035 | 0.002015 |  | 0.0102 | 0.007311 | 0.002015 |  |  |  |  | 0.526447 |
| Angelakisella_massiliensis | 0.017255 | 0.019149 | 0.002464 |  | 0.004798 | 0.003166 | 0.001975 |  |  |  |  | 0.748602 |
| DNF00809_sp000814825 | 0.016985 | 0.011629 | 0.01069 |  | 0.004206 | 0.003561 | 0.006065 |  |  |  |  | 0.353983 |
| Bilophila_wadsworthia | 0.016833 | 0.013602 | 0.001474 |  | 0.013226 | 0.004907 | 0.000662 |  |  |  |  | 0.82346 |
| Oscillibacter_ruminantium | 0.016527 | 0.012304 | 0.001511 |  | 0.005246 | 0.003913 | 0.001511 |  |  |  |  | 0.533355 |
| QAMH01_sp003149935 | 0.015127 | 0.005464 | 0.002336 |  | 0.004229 | 0.003156 | 0.001108 |  |  |  |  | 0.097015 |
| Gordonibacter_massiliensis | 0.015052 | 0 | 0 |  | 0.003715 | 0 | 0 |  |  |  |  | 0.002317 |
| Muribaculum_intestinale | 0.015007 | 0.625072 | 0.299145 |  | 0.002543 | 0.165734 | 0.214023 |  |  |  |  | 0.004243 |
| Desulfonispora_thiosulfatigenes | 0.014419 | 0.002359 | 0 |  | 0.009557 | 0.002359 | 0 |  |  |  |  | 0.248595 |
| Dorea_sp002160985 | 0.01441 | 0.001348 | 0 |  | 0.009132 | 0.00061 | 0 |  |  |  |  | 0.184011 |
| Tyzzerella_nexilis | 0.014217 | 0.050604 | 0.033871 |  | 0.005608 | 0.015854 | 0.026645 |  |  |  |  | 0.055753 |
| Helicobacter_D_sp000765695 | 0.014164 | 0.162246 | 0.030615 |  | 0.007873 | 0.100176 | 0.027148 |  |  |  |  | 0.171338 |
| Vallitalea_A_okinawensis | 0.014091 | 0.000414 | 0 |  | 0.005081 | 0.000414 | 0 |  |  |  |  | 0.022985 |
| Massilioclostridium_methylpentosum | 0.013966 | 0.106551 | 0.028112 |  | 0.005438 | 0.030331 | 0.022969 |  |  |  |  | 0.013239 |
| Christensenella_minuta | 0.013888 | 0.010397 | 0.001474 |  | 0.003313 | 0.002872 | 0.000662 |  |  |  |  | 0.444409 |
| Butyricimonas_synergistica | 0.013855 | 0.000832 | 0 |  | 0.002821 | 0.000526 | 0 |  |  |  |  | 0.001077 |
| Roseburia_hominis | 0.013389 | 0.063211 | 0.003326 |  | 0.009748 | 0.041446 | 0.002414 |  |  |  |  | 0.269055 |
| Exiguobacterium_A_acetylicum | 0.013076 | 0.022228 | 0.037053 |  | 0.003154 | 0.002612 | 0.007198 |  |  |  |  | 0.049441 |
| 49-20_sp002436085 | 0.012899 | 0.018081 | 0.013032 |  | 0.001894 | 0.00231 | 0.003338 |  |  |  |  | 0.113494 |
| Holdemania_sp900120005 | 0.01274 | 0.009909 | 0 |  | 0.004361 | 0.002452 | 0 |  |  |  |  | 0.583944 |
| CAG-353_sp900066885 | 0.012336 | 0.225838 | 0.008196 |  | 0.009549 | 0.090903 | 0.004295 |  |  |  |  | 0.041635 |
| Collinsella_provencensis | 0.012194 | 0.023361 | 0.012156 |  | 0.004457 | 0.003967 | 0.00687 |  |  |  |  | 0.090791 |
| NAK82_sp003130875 | 0.011919 | 0.014246 | 0.008734 |  | 0.003148 | 0.003432 | 0.002088 |  |  |  |  | 0.628047 |
| Dehalobacterium_formicoaceticum | 0.011663 | 0.003205 | 0 |  | 0.004794 | 0.001117 | 0 |  |  |  |  | 0.116475 |
| Paramuribaculum_intestinale | 0.011632 | 0.131 | 0.076575 |  | 0.002063 | 0.031667 | 0.062886 |  |  |  |  | 0.003713 |
| CAG-194_sp000432915 | 0.011611 | 0.131169 | 0.041797 |  | 0.005177 | 0.040185 | 0.032328 |  |  |  |  | 0.014515 |
| Weissella_paramesenteroides | 0.011602 | 0.019382 | 0.033611 |  | 0.001904 | 0.003197 | 0.00618 |  |  |  |  | 0.063061 |
| Erwinia_coffeiphila | 0.011529 | 0.002634 | 0 |  | 0.00734 | 0.001399 | 0 |  |  |  |  | 0.261357 |
| RUG754_sp900315895 | 0.0112 | 0.017978 | 0.002519 |  | 0.004411 | 0.006395 | 0.002519 |  |  |  |  | 0.403479 |
| Dorea_scindens | 0.011166 | 0.017846 | 0.0082 |  | 0.005044 | 0.00911 | 0.005098 |  |  |  |  | 0.535662 |
| Agathobaculum_sp900291975 | 0.010994 | 0.011431 | 0.000953 |  | 0.003669 | 0.002302 | 0.000604 |  |  |  |  | 0.92158 |
| ASF356_sp000364165 | 0.010666 | 0.001549 | 0.001797 |  | 0.005717 | 0.001549 | 0.001481 |  |  |  |  | 0.154793 |
| Lachnoclostridium_A_sp002160755 | 0.010611 | 0.015247 | 0.005753 |  | 0.002107 | 0.003595 | 0.003044 |  |  |  |  | 0.291946 |
| Sphingobacterium_mizutaii | 0.010477 | 0.005571 | 0.002877 |  | 0.002025 | 0.001812 | 0.001438 |  |  |  |  | 0.101152 |
| Ruminococcus_flavefaciens_E | 0.010344 | 0.227675 | 0.013969 |  | 0.003998 | 0.095597 | 0.008531 |  |  |  |  | 0.046457 |
| CAG-302_sp002375205 | 0.010146 | 0.004797 | 0.000504 |  | 0.006629 | 0.003166 | 0.000504 |  |  |  |  | 0.483188 |
| CAG-306_sp000980375 | 0.010112 | 0.009884 | 0.007635 |  | 0.003868 | 0.002821 | 0.003557 |  |  |  |  | 0.962992 |
| Parabacteroides_goldsteinii | 0.010021 | 0.009631 | 0.000504 |  | 0.003889 | 0.006391 | 0.000504 |  |  |  |  | 0.959476 |
| Streptococcus_parasanguinis_D | 0.00996 | 0.004576 | 0.43152 |  | 0.004638 | 0.001572 | 0.323796 |  |  |  |  | 0.297394 |
| SR-FBR-E99_sp002403305 | 0.009954 | 0.006014 | 0.001828 |  | 0.001261 | 0.001639 | 0.000583 |  |  |  |  | 0.085818 |
| Bifidobacterium_animalis | 0.009626 | 0 | 0.001215 |  | 0.005526 | 0 | 0.000573 |  |  |  |  | 0.112132 |
| DTU053_sp001512765 | 0.009444 | 0 | 0 |  | 0.003859 | 0 | 0 |  |  |  |  | 0.034402 |
| UBA4782_sp002403095 | 0.009349 | 0.011046 | 0.009703 |  | 0.002381 | 0.002065 | 0.002311 |  |  |  |  | 0.602045 |
| Rhizorhabdus_sp001015195 | 0.009313 | 0.010139 | 0.006416 |  | 0.001647 | 0.001894 | 0.001593 |  |  |  |  | 0.749094 |
| Butyricicoccus_pullicaecorum | 0.009101 | 0.029857 | 0.004872 |  | 0.00517 | 0.011293 | 0.002282 |  |  |  |  | 0.125638 |
| Odoribacter_splanchnicus | 0.008962 | 0.000418 | 0 |  | 0.001988 | 0.000418 | 0 |  |  |  |  | 0.001809 |
| Acetitomaculum_ruminis | 0.008865 | 0.065527 | 0.010446 |  | 0.005696 | 0.028756 | 0.00618 |  |  |  |  | 0.082045 |
| Alistipes_timonensis | 0.00882 | 0.000944 | 0 |  | 0.005724 | 0.000944 | 0 |  |  |  |  | 0.204391 |
| Syntrophobacter_fumaroxidans | 0.008671 | 0.010288 | 0.010502 |  | 0.001758 | 0.001745 | 0.004447 |  |  |  |  | 0.528543 |
| Anaerostipes_sp000508985 | 0.008528 | 0.136429 | 0.023396 |  | 0.005005 | 0.040804 | 0.01197 |  |  |  |  | 0.011037 |
| Beduini_massiliensis | 0.00839 | 0.02083 | 0.009796 |  | 0.00418 | 0.007374 | 0.005122 |  |  |  |  | 0.172966 |
| Exiguobacterium_mexicanum | 0.008303 | 0.015859 | 0.012887 |  | 0.001657 | 0.002768 | 0.003566 |  |  |  |  | 0.041195 |
| Staphylococcus_cohnii | 0.008174 | 0 | 0.010439 |  | 0.003167 | 0 | 0.00763 |  |  |  |  | 0.02739 |
| Enteroscipio_sp000270285 | 0.007927 | 0.011085 | 0.001906 |  | 0.001951 | 0.002829 | 0.001208 |  |  |  |  | 0.379803 |
| SK-Y3_sp002252565 | 0.007633 | 0.016087 | 0.00588 |  | 0.00252 | 0.004703 | 0.003217 |  |  |  |  | 0.144191 |
| Enteroscipio_rubneri | 0.007545 | 0.019329 | 0.005642 |  | 0.006133 | 0.004237 | 0.001797 |  |  |  |  | 0.145004 |
| DNF00809_sp001552935 | 0.007498 | 0.00959 | 0.002822 |  | 0.003403 | 0.003022 | 0.001707 |  |  |  |  | 0.655575 |
| UTCFX2_sp002050125 | 0.007392 | 0.009542 | 0.011731 |  | 0.001143 | 0.002934 | 0.002384 |  |  |  |  | 0.510243 |
| CAG-484_sp000431315 | 0.007362 | 0.015159 | 0.003775 |  | 0.002528 | 0.004671 | 0.002007 |  |  |  |  | 0.172871 |
| Anaerotignum_lactatifermentans | 0.007187 | 0.085735 | 0.010359 |  | 0.002475 | 0.025192 | 0.007732 |  |  |  |  | 0.011193 |
| CAG-495_sp001917125 | 0.007079 | 0.017769 | 0.001924 |  | 0.002618 | 0.011229 | 0.000991 |  |  |  |  | 0.375681 |
| Hydrogenoanaerobacterium_saccharovorans | 0.007012 | 0.004205 | 0.000449 |  | 0.001629 | 0.001195 | 0.000449 |  |  |  |  | 0.194983 |
| unclassified | 0.006844 | 0.004101 | 0.008794 |  | 0.00589 | 0.000948 | 0.003124 |  |  |  |  | 0.655516 |
| UBA1436_sp002159705 | 0.006586 | 0.003251 | 0.002013 |  | 0.003403 | 0.002312 | 0.00103 |  |  |  |  | 0.436409 |
| UBA6398_sp002451695 | 0.006357 | 0 | 0 |  | 0.005469 | 0 | 0 |  |  |  |  | 0.27211 |
| Parabacteroides_merdae | 0.006047 | 0 | 0.006777 |  | 0.002386 | 0 | 0.006777 |  |  |  |  | 0.029666 |
| Thioalkalivibrio_A_denitrificans | 0.005987 | 0.009731 | 0.002883 |  | 0.002182 | 0.002347 | 0.001541 |  |  |  |  | 0.26983 |
| UBA1033_sp001695555 | 0.005746 | 0 | 0 |  | 0.00125 | 0 | 0 |  |  |  |  | 0.000983 |
| Chryseobacterium_oncorhynchi | 0.005744 | 0.002854 | 0.000504 |  | 0.002659 | 0.001023 | 0.000504 |  |  |  |  | 0.334299 |
| Monoglobus_pectinilyticus | 0.005583 | 0.125523 | 0.052033 |  | 0.002053 | 0.014788 | 0.029929 |  |  |  |  | 1.14E-05 |
| Dorea_longicatena_B | 0.005505 | 0.015295 | 0.002499 |  | 0.002109 | 0.005522 | 0.001442 |  |  |  |  | 0.128672 |
| Vagococcus_sp002140795 | 0.005496 | 0.004271 | 0.010195 |  | 0.002889 | 0.001624 | 0.001774 |  |  |  |  | 0.719267 |
| Thermoflavifilum_aggregans | 0.00549 | 0.003536 | 0.001256 |  | 0.002245 | 0.001072 | 0.000592 |  |  |  |  | 0.450359 |
| CG2-30-66-27_sp001873935 | 0.005442 | 0.00768 | 0.002853 |  | 0.001667 | 0.001109 | 0.000787 |  |  |  |  | 0.289827 |
| Christensenella_A_timonensis | 0.005351 | 0.009789 | 0.004455 |  | 0.00191 | 0.002599 | 0.002515 |  |  |  |  | 0.198946 |
| Blautia_A_sp900120195 | 0.005329 | 0.000988 | 0.000504 |  | 0.003436 | 0.000626 | 0.000504 |  |  |  |  | 0.242117 |
| Paraprevotella_clara | 0.005306 | 0 | 0 |  | 0.003363 | 0 | 0 |  |  |  |  | 0.145673 |
| ER4_sp000765235 | 0.005268 | 0.002644 | 0 |  | 0.003019 | 0.000967 | 0 |  |  |  |  | 0.427032 |
| Olsenella_profusa | 0.005171 | 0 | 0.00185 |  | 0.002485 | 0 | 0.001533 |  |  |  |  | 0.064122 |
| Schwartzia_succinivorans | 0.005121 | 2.962153 | 0.334873 |  | 0.001682 | 0.781735 | 0.154096 |  |  |  |  | 0.003586 |
| Effusibacillus_lacus | 0.005063 | 0.002304 | 0.00601 |  | 0.001694 | 0.001347 | 0.002355 |  |  |  |  | 0.231055 |
| Anaerofustis_stercorihominis | 0.004872 | 0.003975 | 0.000504 |  | 0.002049 | 0.002178 | 0.000504 |  |  |  |  | 0.770289 |
| Blautia_A_sp900066205 | 0.00486 | 0.008545 | 0.007652 |  | 0.002698 | 0.003194 | 0.004547 |  |  |  |  | 0.39888 |
| Anaerotruncus_rubiinfantis | 0.00486 | 0.004989 | 0.001008 |  | 0.000843 | 0.000832 | 0.001008 |  |  |  |  | 0.915444 |
| Dorea_sp001185345 | 0.004845 | 0.167986 | 0.054291 |  | 0.00125 | 0.04915 | 0.041893 |  |  |  |  | 0.007772 |
| Bacteroides_oleiciplenus | 0.004828 | 0 | 0 |  | 0.001822 | 0 | 0 |  |  |  |  | 0.024304 |
| Sphingomonas_B_changbaiensis | 0.004777 | 0.007102 | 0.003895 |  | 0.001207 | 0.001588 | 0.00135 |  |  |  |  | 0.270755 |
| Escherichia_flexneri | 0.004712 | 0.842007 | 0.225627 |  | 0.000921 | 0.307422 | 0.183971 |  |  |  |  | 0.021428 |
| Symbiobacterium_thermophilum | 0.004525 | 0.005514 | 0.003169 |  | 0.001435 | 0.001275 | 0.001459 |  |  |  |  | 0.617784 |
| Enterococcus_ureasiticus | 0.004421 | 0.011339 | 0.016332 |  | 0.000608 | 0.001869 | 0.004372 |  |  |  |  | 0.005538 |
| UBA3006_sp002367695 | 0.004326 | 0.005047 | 0.009261 |  | 0.000464 | 0.000872 | 0.004138 |  |  |  |  | 0.482517 |
| Defluviitalea_phaphyphila | 0.004281 | 0.002834 | 0.000504 |  | 0.002616 | 0.001752 | 0.000504 |  |  |  |  | 0.655643 |
| Anaerobiospirillum_A_thomasii | 0.004272 | 1.625579 | 0.043895 |  | 0.001582 | 0.424722 | 0.031319 |  |  |  |  | 0.003388 |
| Corynebacterium_stationis | 0.004134 | 0.004189 | 0.433387 |  | 0.001318 | 0.0013 | 0.215528 |  |  |  |  | 0.97658 |
| Coprobacter_fastidiosus | 0.004095 | 0 | 0 |  | 0.001622 | 0 | 0 |  |  |  |  | 0.030132 |
| Mesotoga_infera_B | 0.004094 | 0.007004 | 0 |  | 0.001292 | 0.005414 | 0 |  |  |  |  | 0.612489 |
| Rodentibacter_heidelbergensis | 0.004037 | 0.021776 | 0.005267 |  | 0.002245 | 0.005814 | 0.002138 |  |  |  |  | 0.017367 |
| 14-2_sp000403315 | 0.003936 | 0.015375 | 0.001492 |  | 0.002509 | 0.013348 | 0.000669 |  |  |  |  | 0.419362 |
| Clostridium_Q_symbiosum | 0.003743 | 0.010512 | 0.000504 |  | 0.001487 | 0.004366 | 0.000504 |  |  |  |  | 0.172902 |
| Ruminiclostridium_thermocellum | 0.003612 | 0.002527 | 0.001025 |  | 0.002038 | 0.002024 | 0.000648 |  |  |  |  | 0.713579 |
| Pseudomonas_E_sihuiensis | 0.003588 | 0.017414 | 2.003578 |  | 0.00134 | 0.005173 | 1.997858 |  |  |  |  | 0.027077 |
| Microcystis_wesenbergii | 0.003539 | 0.00657 | 0.006045 |  | 0.000907 | 0.00244 | 0.001764 |  |  |  |  | 0.271257 |
| Bacillus_W_sp002559145 | 0.003509 | 0.00181 | 0.006423 |  | 0.001912 | 0.000579 | 0.001907 |  |  |  |  | 0.414879 |
| Savagella_sp000283555 | 0.003474 | 0.000885 | 0.000504 |  | 0.002199 | 0.000562 | 0.000504 |  |  |  |  | 0.280536 |
| UBA1174_sp002311025 | 0.003331 | 0.000875 | 0 |  | 0.001879 | 0.000555 | 0 |  |  |  |  | 0.238693 |
| Actinomyces_I_minihominis | 0.003317 | 0.004214 | 0.005259 |  | 0.001462 | 0.001591 | 0.003354 |  |  |  |  | 0.686682 |
| Chryseobacterium_taihuense | 0.00308 | 0 | 0 |  | 0.00103 | 0 | 0 |  |  |  |  | 0.013588 |
| Serinibacter_sp003121705 | 0.00304 | 0.006841 | 0.004425 |  | 0.001013 | 0.002029 | 0.001299 |  |  |  |  | 0.12473 |
| UBA6659_sp002344135 | 0.003005 | 0.000889 | 0.000752 |  | 0.000816 | 0.000564 | 0.000496 |  |  |  |  | 0.058682 |
| Acinetobacter_sp000313935 | 0.002976 | 0.00426 | 0.003917 |  | 0.00102 | 0.002381 | 0.003012 |  |  |  |  | 0.630743 |
| Lachnotalea_glycerini | 0.002893 | 0 | 0 |  | 0.00157 | 0 | 0 |  |  |  |  | 0.09515 |
| Clostridium_saudiense | 0.002838 | 0.001242 | 0.032699 |  | 0.001863 | 0.001242 | 0.024944 |  |  |  |  | 0.492227 |
| Butyrivibrio_sp900112195 | 0.002776 | 0.001832 | 0 |  | 0.002308 | 0.001392 | 0 |  |  |  |  | 0.733259 |
| MS4_sp000752215 | 0.002644 | 0.057728 | 0.009831 |  | 0.000956 | 0.015685 | 0.004294 |  |  |  |  | 0.005675 |
| T78_sp002347705 | 0.002641 | 0.003016 | 0.004076 |  | 0.001602 | 0.001657 | 0.001759 |  |  |  |  | 0.873724 |
| Prevotella_sp002251365 | 0.002608 | 0.46442 | 0.016469 |  | 0.000937 | 0.343977 | 0.007856 |  |  |  |  | 0.209096 |
| Prevotella_pleuritidis | 0.002584 | 0.082232 | 0.001492 |  | 0.001201 | 0.053634 | 0.000669 |  |  |  |  | 0.168457 |
| Pectobacterium_carotovorum_E | 0.002558 | 0.003938 | 0.001378 |  | 0.001147 | 0.002039 | 0.000623 |  |  |  |  | 0.568193 |
| Ruminococcus_flavefaciens_G | 0.002528 | 0.104392 | 0.010039 |  | 0.001627 | 0.082035 | 0.00949 |  |  |  |  | 0.242763 |
| Alistipes_shahii | 0.002519 | 0 | 0 |  | 0.001598 | 0 | 0 |  |  |  |  | 0.146071 |
| Phyllobacterium_sp900473175 | 0.00231 | 0.001347 | 0.013945 |  | 0.00093 | 0.000604 | 0.011633 |  |  |  |  | 0.405776 |
| Actinomadura_B_rubrobrunea | 0.002252 | 0.001504 | 0.001437 |  | 0.000458 | 0.001046 | 0.000968 |  |  |  |  | 0.52722 |
| Acetoanaerobium_sticklandii | 0.002226 | 0.001344 | 0 |  | 0.001264 | 0.000884 | 0 |  |  |  |  | 0.580188 |
| Steroidobacter_denitrificans | 0.002191 | 0.002274 | 0.001402 |  | 0.001055 | 0.00132 | 0.000938 |  |  |  |  | 0.961845 |
| Ruminococcus_C_callidus | 0.002122 | 0.15311 | 0.001904 |  | 0.00078 | 0.088629 | 0.000943 |  |  |  |  | 0.11929 |
| Staphylococcus_A_lentus | 0.002114 | 0 | 0.069123 |  | 0.001141 | 0 | 0.044313 |  |  |  |  | 0.093519 |
| Treponema_D_bryantii_A | 0.002093 | 2.55065 | 0.469564 |  | 0.00074 | 0.961718 | 0.445419 |  |  |  |  | 0.024313 |
| Prevotella_sp001275135 | 0.002085 | 0.294195 | 0.051141 |  | 0.001005 | 0.157677 | 0.034049 |  |  |  |  | 0.093654 |
| Hyphomicrobium_zavarzinii | 0.002056 | 0.003219 | 0.001546 |  | 0.000983 | 0.001271 | 0.001063 |  |  |  |  | 0.485892 |
| QKVK01_sp003234965 | 0.00182 | 0.003308 | 0.000807 |  | 0.000882 | 0.001597 | 0.000542 |  |  |  |  | 0.433894 |
| Chryseobacterium_geocarposphaerae | 0.001777 | 0.004251 | 0.001492 |  | 0.000571 | 0.001288 | 0.00105 |  |  |  |  | 0.109608 |
| CAG-791_sp900317475 | 0.001763 | 0.036824 | 0.004006 |  | 0.001323 | 0.013635 | 0.002103 |  |  |  |  | 0.028399 |
| GCA-900066575_sp002160765 | 0.001718 | 0 | 0 |  | 0.000872 | 0 | 0 |  |  |  |  | 0.07713 |
| Prevotella_sp002933775 | 0.001682 | 0.160615 | 0.003703 |  | 0.000533 | 0.120267 | 0.002437 |  |  |  |  | 0.215774 |
| Faecalibacterium_prausnitzii_E | 0.001642 | 0.002708 | 0.000504 |  | 0.00104 | 0.000733 | 0.000504 |  |  |  |  | 0.42199 |
| Tolypothrix_B_campylonemoides | 0.001606 | 0.002794 | 0.001492 |  | 0.00117 | 0.001031 | 0.000669 |  |  |  |  | 0.463955 |
| Aquamicrobium_A_aerolatum | 0.00141 | 0.001361 | 0.058415 |  | 0.000939 | 0.000611 | 0.029253 |  |  |  |  | 0.966045 |
| Thiobacillus_denitrificans | 0.001366 | 0.000923 | 0.001075 |  | 0.000616 | 0.000923 | 0.000682 |  |  |  |  | 0.698328 |
| Lactobacillus_F_plantarum_A | 0.001366 | 0.00193 | 0.004772 |  | 0.000616 | 0.001405 | 0.001924 |  |  |  |  | 0.720507 |
| Clostridium_Q_sp003024715 | 0.001326 | 0.008162 | 0 |  | 0.000903 | 0.00339 | 0 |  |  |  |  | 0.07992 |
| GW-Nitrospira-1_sp002839535 | 0.001315 | 0.003169 | 0 |  | 0.000588 | 0.000873 | 0 |  |  |  |  | 0.108631 |
| Agrobacterium_nepotum | 0.001315 | 0.006386 | 0.545879 |  | 0.000588 | 0.00112 | 0.539579 |  |  |  |  | 0.002484 |
| UBA1777_sp900319835 | 0.001315 | 0.176698 | 0.035049 |  | 0.000588 | 0.020304 | 0.022144 |  |  |  |  | 6E-06 |
| Acinetobacter_kookii | 0.001304 | 0 | 0.033579 |  | 0.000884 | 0 | 0.032076 |  |  |  |  | 0.170857 |
| Corynebacterium_nuruki | 0.0013 | 0.007136 | 0.0019 |  | 0.000895 | 0.001942 | 0.000608 |  |  |  |  | 0.02122 |
| Longilinea_arvoryzae | 0.001297 | 0.001865 | 0.003972 |  | 0.00058 | 0.001 | 0.002496 |  |  |  |  | 0.634147 |
| Actinomyces_bovis | 0.001279 | 0.004998 | 0.370308 |  | 0.000574 | 0.001364 | 0.273697 |  |  |  |  | 0.030754 |
| Lactobacillus_delbrueckii | 0.001264 | 0 | 0.021375 |  | 0.001264 | 0 | 0.021375 |  |  |  |  | 0.340893 |
| UBA8950_sp001872455 | 0.001261 | 0.00263 | 0.001324 |  | 0.000566 | 0.001396 | 0.000594 |  |  |  |  | 0.384658 |
| Intestinimonas_massiliensis | 0.001261 | 0.006781 | 0.000504 |  | 0.000566 | 0.001948 | 0.000504 |  |  |  |  | 0.021495 |
| Fermentibacter_daniensis | 0.001253 | 0.001877 | 0.00311 |  | 0.000562 | 0.000944 | 0.001191 |  |  |  |  | 0.582721 |
| Eubacterium_C_oxidoreducens | 0.001224 | 0.019145 | 0.004497 |  | 0.000819 | 0.004761 | 0.003383 |  |  |  |  | 0.004044 |
| Butyricicoccus_A_porcorum | 0.000963 | 0.003208 | 0.00142 |  | 0.000611 | 0.001291 | 0.000636 |  |  |  |  | 0.146979 |
| Bacillus_haynesii | 0.000955 | 0 | 0.047954 |  | 0.000607 | 0 | 0.035191 |  |  |  |  | 0.146655 |
| Escherichia_coli_D | 0.000894 | 0 | 0 |  | 0.000894 | 0 | 0 |  |  |  |  | 0.340893 |
| Bradyrhizobium_sp000617845 | 0.000886 | 0.001352 | 0.012277 |  | 0.000561 | 0.00089 | 0.006132 |  |  |  |  | 0.667429 |
| GCA-900066905_sp900066905 | 0.000876 | 0 | 0.000504 |  | 0.000554 | 0 | 0.000504 |  |  |  |  | 0.145048 |
| Corynebacterium_freneyi | 0.000876 | 0.003214 | 0.248031 |  | 0.000554 | 0.000871 | 0.103735 |  |  |  |  | 0.046911 |
| Zag1_sp001765415 | 0.000868 | 0.072148 | 0.019743 |  | 0.000549 | 0.026003 | 0.010819 |  |  |  |  | 0.020809 |
| Flavisolibacter_ginsengisoli | 0.000857 | 0.000828 | 0 |  | 0.000857 | 0.000828 | 0 |  |  |  |  | 0.980898 |
| Treponema_D_sp002296965 | 0.000857 | 0.434312 | 0.054455 |  | 0.000857 | 0.095745 | 0.048988 |  |  |  |  | 0.001096 |
| Reyranella_sp001557035 | 0.00085 | 0.000418 | 0.008537 |  | 0.000538 | 0.000418 | 0.005968 |  |  |  |  | 0.53982 |
| Prevotella_maculosa | 0.000843 | 0.000472 | 0.030238 |  | 0.000843 | 0.000472 | 0.030238 |  |  |  |  | 0.708953 |
| Alicyclobacillus_B_ferrooxydans | 0.000832 | 0.001921 | 0.001361 |  | 0.000527 | 0.000609 | 0.000613 |  |  |  |  | 0.206194 |
| Aminicenans_sakinawicola | 0.000832 | 0.002275 | 0.000449 |  | 0.000527 | 0.00107 | 0.000449 |  |  |  |  | 0.254162 |
| RC9_sp002438635 | 0.000832 | 0.068164 | 0.019572 |  | 0.000527 | 0.012794 | 0.015547 |  |  |  |  | 0.000369 |
| Citrobacter_portucalensis | 0.000821 | 0 | 0.041409 |  | 0.00052 | 0 | 0.031496 |  |  |  |  | 0.145452 |
| Massilia_timonae_A | 0.000821 | 0.000418 | 0.00782 |  | 0.00052 | 0.000418 | 0.00782 |  |  |  |  | 0.558964 |
| Methanothrix_soehngenii | 0.000821 | 0.000889 | 0.001219 |  | 0.00052 | 0.000564 | 0.00092 |  |  |  |  | 0.931089 |
| Sphingobacterium_spiritivorum_A | 0.000821 | 0.038875 | 0.011101 |  | 0.00052 | 0.01427 | 0.010488 |  |  |  |  | 0.023701 |
| Bacillus_C_aryabhattai | 0.000814 | 0.00093 | 0.022456 |  | 0.000515 | 0.000594 | 0.012509 |  |  |  |  | 0.885133 |
| COE1_sp000403215 | 0.000785 | 0.011808 | 0.001025 |  | 0.000785 | 0.004107 | 0.000648 |  |  |  |  | 0.024902 |
| URHD0088_sp000518365 | 0.000516 | 0.002222 | 0.000449 |  | 0.000516 | 0.00106 | 0.000449 |  |  |  |  | 0.178314 |
| Anaerocolumna_jejuensis | 0.000516 | 0.005168 | 0.002536 |  | 0.000516 | 0.003364 | 0.001978 |  |  |  |  | 0.201581 |
| Sphingobacterium_sp002980525 | 0.000447 | 0 | 0.053114 |  | 0.000447 | 0 | 0.025328 |  |  |  |  | 0.340893 |
| Pseudomonas_E_anguilliseptica | 0.000447 | 0 | 0.021896 |  | 0.000447 | 0 | 0.021896 |  |  |  |  | 0.340893 |
| Muribaculum_sp003150235 | 0.000447 | 0.00367 | 0.001457 |  | 0.000447 | 0.001314 | 0.001018 |  |  |  |  | 0.042677 |
| Prevotella_sp002251385 | 0.000447 | 0.004107 | 0.001184 |  | 0.000447 | 0.00114 | 0.000887 |  |  |  |  | 0.013617 |
| UBA1711_sp001543385 | 0.000447 | 0.114237 | 0.016762 |  | 0.000447 | 0.034066 | 0.011954 |  |  |  |  | 0.007491 |
| Ruminiclostridium_A_sp000244875 | 0.000447 | 0.233603 | 0 |  | 0.000447 | 0.232984 | 0 |  |  |  |  | 0.340554 |
| Streptococcus_caballi | 0.000447 | 0.235939 | 0.283418 |  | 0.000447 | 0.0569 | 0.1744 |  |  |  |  | 0.002016 |
| Sphingopyxis_sp000756385 | 0.000439 | 0 | 0.049006 |  | 0.000439 | 0 | 0.049006 |  |  |  |  | 0.340893 |
| Pannonibacter_indicus | 0.000439 | 0 | 0.030741 |  | 0.000439 | 0 | 0.030141 |  |  |  |  | 0.340893 |
| Clostridium_C_acetireducens | 0.000439 | 0 | 0.014076 |  | 0.000439 | 0 | 0.014076 |  |  |  |  | 0.340893 |
| Brevibacterium_senegalense | 0.000439 | 0 | 0.045601 |  | 0.000439 | 0 | 0.031401 |  |  |  |  | 0.340893 |
| Bog-159_sp003166475 | 0.000439 | 0 | 0.000504 |  | 0.000439 | 0 | 0.000504 |  |  |  |  | 0.340893 |
| Halomonas_D_sp002286965 | 0.000439 | 0.000988 | 0.000979 |  | 0.000439 | 0.000626 | 0.000632 |  |  |  |  | 0.489269 |
| Alicyclobacillus_H_macrosporangiidus | 0.000439 | 0.001404 | 0.000875 |  | 0.000439 | 0.000963 | 0.000555 |  |  |  |  | 0.383479 |
| Roseburia_intestinalis | 0.000439 | 0.01497 | 0.001008 |  | 0.000439 | 0.005486 | 0.001008 |  |  |  |  | 0.024722 |
| Lentimicrobium_saccharophilum | 0.000439 | 0.068304 | 0.009803 |  | 0.000439 | 0.018812 | 0.008933 |  |  |  |  | 0.004796 |
| Tissierella_A_creatinophila | 0.000429 | 0 | 0.000735 |  | 0.000429 | 0 | 0.000482 |  |  |  |  | 0.340893 |
| Corynebacterium_urealyticum | 0.000429 | 0 | 0.000898 |  | 0.000429 | 0 | 0.000898 |  |  |  |  | 0.340893 |
| Tatlockia_lansingensis | 0.000429 | 0.000461 | 0.123788 |  | 0.000429 | 0.000461 | 0.122886 |  |  |  |  | 0.959415 |
| Gracilibacter_sp001516055 | 0.000429 | 0.001395 | 0 |  | 0.000429 | 0.000949 | 0 |  |  |  |  | 0.375273 |
| Psychrobacter_pasteurii | 0.000429 | 0.005074 | 0.030083 |  | 0.000429 | 0.003611 | 0.014353 |  |  |  |  | 0.230274 |
| RC9_sp900167895 | 0.000429 | 0.028679 | 0.0109 |  | 0.000429 | 0.011754 | 0.008771 |  |  |  |  | 0.03719 |
| Megasphaera_sp000417505 | 0.000421 | 0 | 0.181707 |  | 0.000421 | 0 | 0.180119 |  |  |  |  | 0.340893 |
| Cryobacterium_A_mesophilum | 0.000421 | 0.000516 | 0.010447 |  | 0.000421 | 0.000516 | 0.005362 |  |  |  |  | 0.889518 |
[truncated: 11,759 more chars]
